# Comparison of characteristics of long noncoding RNA in Hanwoo according to sex

**DOI:** 10.5713/ajas.18.0533

**Published:** 2019-10-22

**Authors:** Jae-Young Choi, KyeongHye Won, Seungwoo Son, Donghyun Shin, Jae-Don Oh

**Affiliations:** 1 Subtropical Livestock Research Institute, National Institute of Animal Science, RDA, Jeju 63242, Korea; 2 Department of Animal Biotechnology, College of Agricultural and Life Sciences, Chonbuk National University, Jeonju 54896, Korea; 3 The Animal Molecular Genetics & Breeding Center, Chonbuk National University, Jeonju, 54896, Korea

**Keywords:** Hanwoo, Sex, LncRNA, Quantitative Trait Loci (QTL), Muscle

## Abstract

**Objective:**

Cattle were some of the first animals domesticated by humans for the production of milk, meat, etc. Long noncoding RNA (lncRNA) is defined as longer than 200 bp in non-protein coding transcripts. lncRNA is known to function in regulating gene expression and is currently being studied in a variety of livestock including cattle. The purpose of this study is to analyze the characteristics of lncRNA according to sex in Hanwoo cattle.

**Methods:**

This study was conducted using the skeletal muscles of 9 Hanwoo cattle include bulls, steers and cows. RNA was extracted from skeletal muscle of Hanwoo. Sequencing was conducted using Illumina HiSeq2000 and mapped to the Bovine Taurus genome. The expression levels of lncRNAs were measured by DEGseq and quantitative trait loci (QTL) data base was used to identify QTLs associated with lncRNA. The python script was used to match the nearby genes

**Results:**

In this study, the expression patterns of transcripts of bulls, steers and cows were identified. And we identified significantly differentially expressed lncRNAs in bulls, steers and cows. In addition, characteristics of lncRNA which express differentially in muscles according to the sex of Hanwoo were identified. As a result, we found differentially expressed lncRNAs according to sex were related to shear force and body weight.

**Conclusion:**

This study was classified and characterized lncRNA which differentially expressed by sex in Hanwoo cattle. We believe that the characterization of lncRNA by sex of Hanwoo will be helpful for future studies of the physiological mechanisms of Hanwoo cattle.

## INTRODUCTION

Cattle were some of the first animals to be domesticated by humans for agricultural purposes. About 10,000 years ago, the ancestors of cattle were tamed for the purpose of providing milk, meat, and leather to humans [1]. In 2006, a human genome sequencing center announced the sequence of the cattle genome [2]. Utilizing the genome-wide single nucleotide polymorphism panel for cattle allows for the use of quantitative trait loci (QTL) and prediction of the genetic merit of an animal without using phenotype and pedigree records [3]. Genetic studies using livestock have shown that most of the genetic variants associated with complex phenotypic traits are located outside the protein coding region [4].

Analysis of intracellular transcripts has revealed that 50% of the transcribed genomes were not aligned with known protein coding regions. Many of these have been proven to have protein coding potential [5]. Non-protein coding transcripts can potentially be noncoding RNA (ncRNA) [6]. Long noncoding RNA (lncRNA) is a molecule longer than 200 bp in a non-protein-coding transcript, or one longer than 2 kb with a coding potential of an amino acid sequence less than 100 bp [7]. LncRNA is a relatively new class of RNA molecules that are less well-characterized than microRNA (miRNA). It has been characterized only by some functional lncRNA and has been shown to regulate all levels of the gene-regulated expression pathway [8]. Previous studies have confirmed that lncRNA plays an important role in a variety of key biological processes, including translational control, RNA splicing, and chromatin modification [9]. In addition, functional studies of lncRNAs have shown that they play an important role in basic biological processes such as dose compensation, transcriptional regulation, and epigenetic regulation [10–12]. The number of studies on lncRNA has continued to increase and many databases have been constructed that include lncRNA data for domesticated animals [13]. At the time of the announcement, 12,103 pig lncRNAs, 8,923 chicken lncRNAs, and 8,250 cattle lncRNAs were included in the ALDB (domestic-animal lncRNA database) database [14].

This study was conducted to compare the expression of lncRNA in bulls, steers and cows as well as the characteristics of each sex. Samples from muscles tissues were used for each sex, and the expression of lncRNA by tissue was analyzed according to sex. We also analyzed QTL associated with lncRNA, which is significantly expressed.

## MATERIALS AND METHODS

### Sample preparation and RNA-seq snalysis

All analysis was conducted with data reprocessed from the law data from a previous study [15]. The animals and sample preparation were as follows: A total of nine (three bulls, three steers and three cows) Hanwoo cattle (*Bos taurus coreanae*) were used in this study. They were fed the same diet and managed at the same location, Hanwoo Experimental Station in National Institute of Animal Science, throughout the experiment. The average (±standard deviation) carcass weight of the cattle at slaughter was 430.2 (±40.66) kg. Immediately after slaughter, muscle tissues were separated and sampled. All of the tissue samples were immediately frozen using liquid nitrogen and stored at −80°C until analysis. All animal use, care, and experimental protocols for this experiment were reviewed and preapproved by the Institutional Animal Care and Use Committee of the National Institute of Animal Science (number 2010-042). Total RNAs of tissues were isolated using TRIzol (Invitrogen, Karlsruhe, Germany) and an RNeasy RNA purification kit with DNase treatment (Qiagen, Hilden, Germany). The messenger RNA (mRNA) was isolated from the total RNA using oligo-dT beads and was reverse transcribed into double-stranded cDNA fragments. Constructing and sequencing of the RNA-seq library for each sample was carried out based on the protocols of Illumina HiSeq2000 in order to generate 101 pair-end reads. The qualities of the RNA-seq reads from all of the tissues were checked using FastQC.

### Analysis of lncRNA discovery

Filtering was conducted in order to remove the low-quality sequences. The filtered sequences were mapped to Bovine Taurus genome (bosTau6) using STAR v2.4.0b [16]. Expression levels were calculated using Cufflinks v2.2.1. Bovine gene information was used to measure expression levels [17]. Multi read correction and frag bias correct options were additionally used to increase the accuracy of expression measurement, and other options were used as defaults. The bovine lncRNA analysis was used to conduct mapping with reference to annotated bos taurus ensemble ID results. We refer to the lncRNA list of cattle identified in the study of Koufariotis et al [18]. We matched the ensemble ID of the transcripts we found and identified lncRNA in the muscle of Hanwoo (ensembl.org).

### Statistical analysis

Differences in the expression level of each tissue were expressed by Heatmap using R package gplots (v3.0.1). LncRNA principal component analysis (PCA) plots were used to identify the differences between bulls, steers and cows muscle tissues using Mev (http://mev.tm4.org/). The DEGseq R package was used to identify differential expression of lncRNA between the bulls, steers and cows muscle tissues. Significant lncRNAs were identified using cut-off of: |fold difference| ≥1 and p-value ≤0.05. Sex-specific differentially expressed lncRNAs statistics analysis were using Prism 5 program (San Diego, CA, USA). The Venn diagram was used to identify the lncRNA assemblages that were extracted between the bulls, steers and cows. The Venn diagram was displayed using InteractiVenn (http://www.interactivenn.net/).

### Quantitative trait locus analysis

QTL regions for comparative analysis with lncRNAs were identified from the cattle QTL Database (http://www.animalgenome.org/cgi-bin/QTLdb/BT/index). In these cattle, the QTL associated with the quality and productivity of the meat was selected. The position of the selected QTL was compared with the lncRNA expressed in sex.

### Nearby gene analysis

The location of lncRNA was identified by ensembl biomart (ensembl.org/biomart) using transcript ID. The databases used included ensemble genes 92 cow genes (UMD3.1). We found for by nearby gene predicted to be affected by lncRNA. The position information of lncRNA in the genome and the gene position information of the bovine were matched using Python script. Transcription directions of lncRNA and nearby genes were used transcript information by ensembl (ensembl.org).

## RESULTS AND DISCUSSION

### Information of RNA sequencing

All analyses were conducted by reprocessing data from previous studies (Lee et al [15]). The RNA-seq raw data information is as follows: 34.2 Mb of raw readings were averaged on the muscle. More than 99.5% readings were retained after being filtered by quality control, and over 95.9% of these were mapped to the reference genome. The average length of the lncRNA was 866 bp, the minimum length was 257 bp, and the maximum length was 1,911 bp.

### Expression patterns of lncRNA in Hanwoo cattle by sex

**Figure 1:**
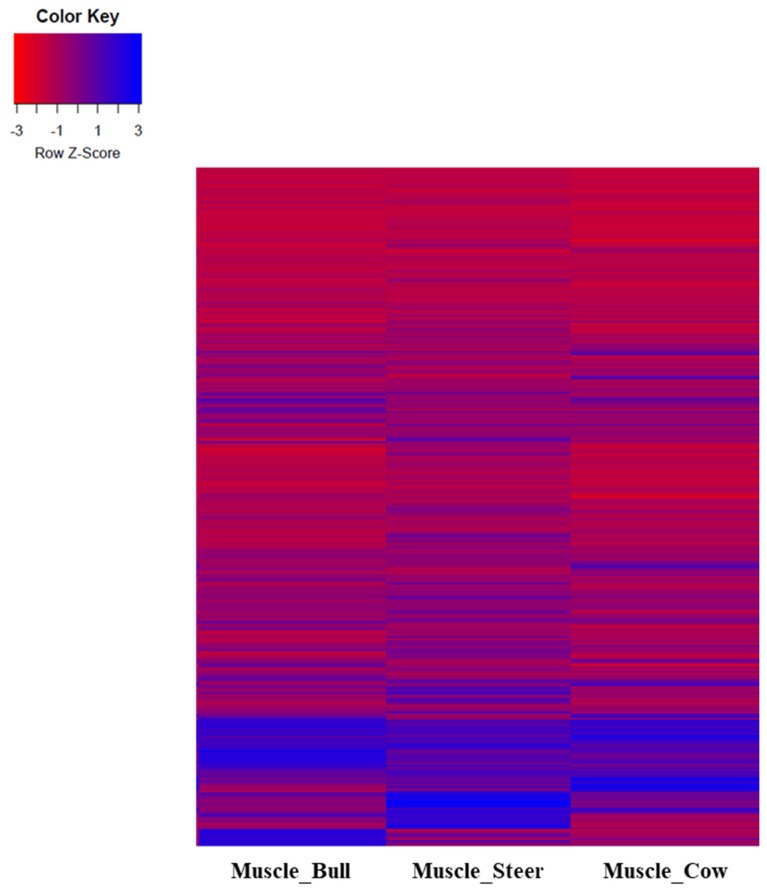
Comparison of sex transcripts expression in Hanwoo muscles. Expression of transcripts by muscle tissue of bull, steer and cow were identified. Expression of each sex was expressed as z-score, with red as negative and blue as positive.

**Figure 2:**
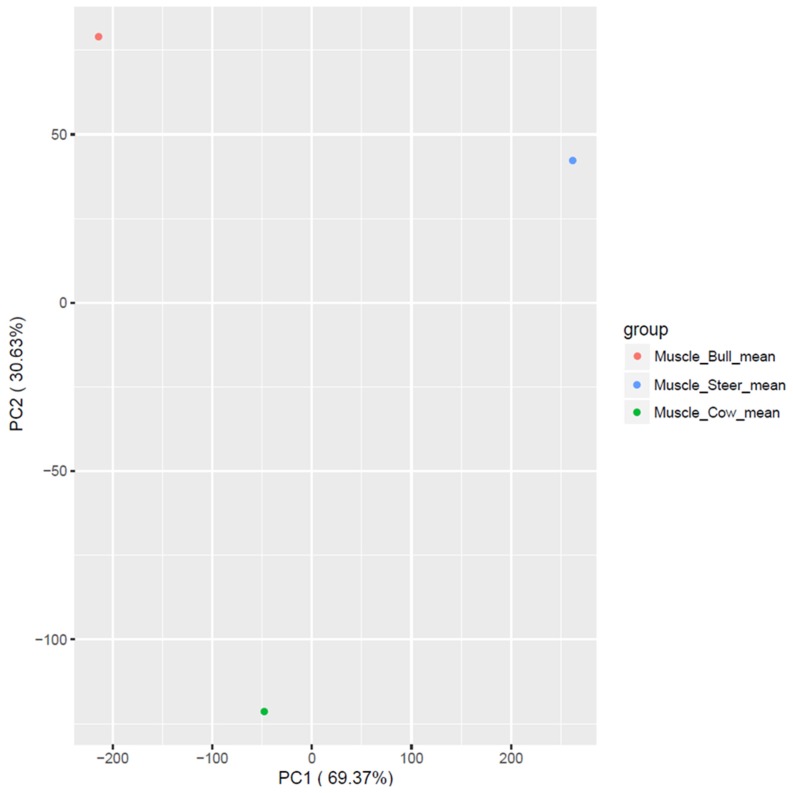
lncRNA principal component analysis plot of bulls, steers and cows in muscle tissues. Clustering analysis was conducted to determine the differences between the three sex. The distance between bull and steer is relatively close compared to cow. Red color is bull, blue color is steer and green color is cow.

**Figure 3:**
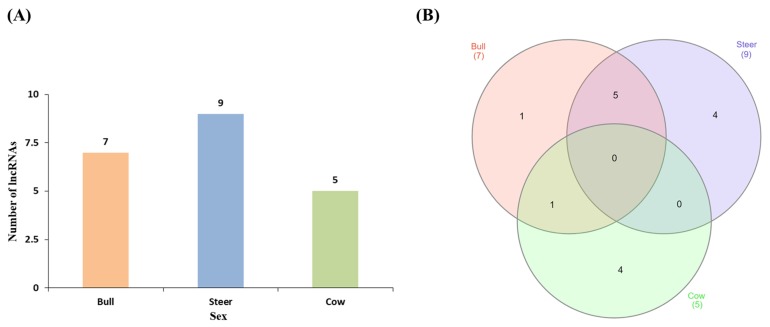
Number of lncRNA found between each sex. (A) Number of lncRNAs by sex in muscle. (B) Venn diagram showing the co-expression of lncRNAs in bulls, steers and cows. Many of the lncRNAs from Bull and Steer were found to overlap. Red color is bull, blue color is steer and green color is cow.

A hierarchical clustering analysis of the transcripts of sex transcripts in muscle tissue was performed in Hanwoo cattle. Clustering analysis of steer expressions did not show any significant differences (Figure 1). Clustering analysis using PCA analysis showed that the distances between the three groups differed by sex. The distance between the bull and the steer was relatively close to the distance to the cow (Figure 2). An analysis of the differential expression of lncRNA in each sex was conducted and found seven lncRNAs in bulls, nine lncRNAs in steers, and five lncRNAs in cows (Table 1, Figure 3A). The results of Venn diagram analysis for the found lncRNA revealed that 5 lncRNAs were found commonly in bulls and steers. We found one common lncRNA that was differentially expressed in bulls and cows. It was also identified that there was no overlapped lncRNA in the steers and cows (Figure 3B).

### LncRNA-related bovine QTL analysis

Functional traits are defined as characteristics of animals that increase efficiency by reducing the cost of breeding [19]. Livestock species have long been selected to improve their characteristics that are associated with economic benefits. These traits are generally affected by genetic and environmental factors [20]. The detection of genetic markers closely linked to loci affecting quantitative traits (QTL) will provide a tool for the application of marker-assisted selection and is a prerequisite for the detailed molecular genetic analysis of traits [21]. Previous studies have identified 7188 lncRNAs from cows and have identified 2753 loci on chromosomal regions of livestock QTL related to muscle development. The function of QTL was also related to intramuscular fat, lean meat yield, longissimus muscle area, shear force [22].

**Figure 4:**
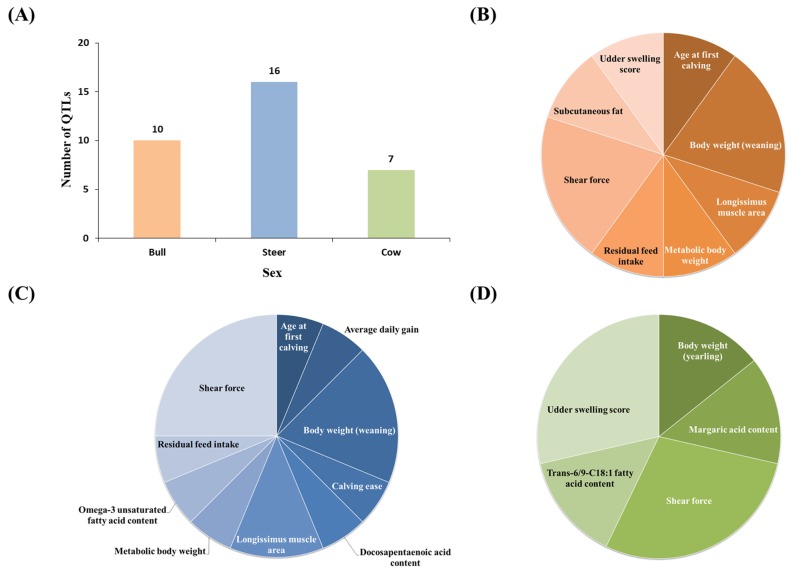
Number of quantitative trait loci (QTLs) associated with Hanwoo cattle economic traits by sex in muscle. (A) Number of QTL loci associated with lncRNA by each sex. (B) QTL locus associated with lncRNA found in bull. (C) QTL locus associated with lncRNA found in steer. (D) QTL locus associated with lncRNA found in cow. Compared to other sex, many QTL loci were identified in steer. All three sex were found to be associated with shear force. Red color is bull, blue color is steer and green color is cow.

In this study, we conducted QTL analysis according to sex in Hanwoo muscle. QTL loci related to meat quality and productivity of cattle were selected with reference to a cattle QTL database. We identified 33 QTLs with overlapping loci with lncRNAs. Ten QTLs were identified in the bulls, 16 QTLs were identified in the steers, and seven QTLs were identified in the cows. In all sexes, lncRNA was found to be mainly associated with shear force QTL (Figure 4, Table 2).

### LncRNA related genes identified

Most lncRNAs have lower conservation and expression levels than mRNA [23]. It has been reported that lncRNA can regulate the expression of adjacent protein coding genes through transcriptional activation and inhibition [24,25]. LncRNAs can be targeted by miRNAs and it has been reported that they can regulate the expression of mRNA [26,27]. The miRNAs consist of a length of about 22 nucleotides and function like silencing RNA and regulating gene expression after transcription [28,29]. In this study, three mRNAs were found to overlap with three lncRNAs. In addition, we identified and classified the transcriptional direction of lncRNA and mRNA (Table 3).

## CONCLUSION

This study profiled lncRNA, which differentially expresses the difference of sex in Hanwoo cattle. We identified the expression patterns of transcripts in each sex by comparing the bull, steer and cow strains and identified 21 lncRNAs. We identified lncRNA related to meat quality and productivity according to sex in Hanwoo through QTL analysis. Many lncRNAs were found to overlap with QTL loci associated with shear force and body weight. We also identified adjacent genes that overlap with the three lncRNAs. It will be helpful to study the genetic characteristics of Hanwoo in the future.

## Figures and Tables

**Table 1:** List of differentially expressed lncRNA of Hanwoo cattle in muscle

Sex	Transcript ID	Loci	Length	log2FC	p-value
Bull	ENSBTAT00000064621	14:20738814-20740407	1,594	−2.27	1.2E-33
	ENSBTAT00000003304	6:62506125-62506524	400	−1.63	3.4E-02
	ENSBTAT00000065672	23:34219298-34219825	528	−1.58	1.7E-44
	ENSBTAT00000009243	5:104077163-104077952	790	−1.20	1.2E-02
	ENSBTAT00000017165	8:31871390-31872132	743	−1.15	3.4E-04
	ENSBTAT00000033843	3:19259499-19260488	990	−1.13	1.4E-05
	ENSBTAT00000005333	6:71053202-71053458	257	1.41	1.7E-02
Steer	ENSBTAT00000064288	3:27428793-27430601	1,809	−1.04	4.6E-03
	ENSBTAT00000048956	8:11064112-11064777	666	−1.00	7.0E-06
	ENSBTAT00000064497	26:14093095-14093609	515	1.04	2.1E-41
	ENSBTAT00000009243	5:104077163-104077952	790	1.06	1.6E-02
	ENSBTAT00000017165	8:31871390-31872132	743	1.26	2.2E-05
	ENSBTAT00000064839	15:74915626-74916613	988	1.28	1.1E-03
	ENSBTAT00000033843	3:19259499-19260488	990	1.36	1.7E-08
	ENSBTAT00000065672	23:34219298-34219825	528	1.46	2.6E-48
	ENSBTAT00000064621	14:20738814-20740407	1,594	1.75	1.8E-29
Cow	ENSBTAT00000005333	6:71053202-71053458	257	−2.09	7.0E-03
	ENSBTAT00000010270	10:76992202-76994112	1,911	−1.48	7.9E-06
	ENSBTAT00000045699	1:84324177-84325181	1,005	−1.02	5.0E-12
	ENSBTAT00000044622	29:40214778-40215227	450	−1.07	3.2E-57
	ENSBTAT00000027478	4:58724265-58724900	636	1.19	1.3E-11

**Table 2:** List of QTLs associated with Hanwoo cattle economic traits

QTL	ID	Chr	Peak	Reference	lncRNA Transcript ID
Bull					
Age at first calving	140097	14	-	Mota RR	ENSBTAT00000064621
Body weight (weaning)	24749	3	20.34	Mahdi Saatchi	ENSBTAT00000033843
	24711	3	28.72		ENSBTAT00000033843
Longissimus muscle area	126448	8	-	de Oliveira Silva RM	ENSBTAT00000017165
Metabolic body weight	140487	14	-	Hardie LC	ENSBTAT00000064621
Residual feed intake	56461	14	31.48	Saatchi M	ENSBTAT00000064621
Shear force	20764	6	71.21	McClure MC	ENSBTAT00000005333
					ENSBTAT00000003304
Subcutaneous fat	20703	14	5.62	Veneroni-Gouveia G	ENSBTAT00000064621
Udder swelling score	106718	6	78.95	Michenet A	ENSBTAT00000005333
Steer					
Age at first calving	140097	14	-	Mota RR	ENSBTAT00000064621
Average daily gain	22798	15	97.09	Peters SO	ENSBTAT00000064839
Body weight (weaning)	24749	3	20.34	Mahdi Saatchi	ENSBTAT00000033843
	24711	3	28.72		ENSBTAT00000033843
					ENSBTAT00000064288
Calving ease	106459	26	20.05	Michenet A	ENSBTAT00000064497
Docosapentaenoic acid content	31772	3	29.66	Cesar AS	ENSBTAT00000064288
Longissimus muscle area	126448	8	-	de Oliveira Silva RM	ENSBTAT00000017165
	126451	15	-		ENSBTAT00000064839
Metabolic body weight	140487	14	-	Hardie LC	ENSBTAT00000064621
Omega-3 unsaturated fatty acid content	31782	3	29.66	Cesar AS	ENSBTAT00000064288
Residual feed intake	56461	14	31.48	Saatchi M	ENSBTAT00000064621
Shear force	20822	26	22.45	McClure MC	ENSBTAT00000064497
	20824	26	40.66		ENSBTAT00000064497
	20823	26	31.95		ENSBTAT00000064497
	20703	14	5.62	Veneroni-Gouveia G	ENSBTAT00000064621
Cow					
Body weight (yearling)	22770	1	109.62	Peters SO	ENSBTAT00000045699
Margaric acid content	19759	29	46.64	Saatchi M	ENSBTAT00000044622
Shear force	20833	29	56.05	McClure MC	ENSBTAT00000044622
	20764	6	71.21		ENSBTAT00000005333
Trans-6/9-C18:1 fatty acid content	20504	1	80.99	Saatchi M	ENSBTAT00000045699
Udder swelling score	106760	29	54.42	Michenet A	ENSBTAT00000044622
	106718	6	78.95		ENSBTAT00000005333

QTLs, quantitative trait loci.

**Table 3:** Information on lncRNAs with nearby genes

LncRNA transcript ID	Gene ID	Gene symbol	Loci	LncRNA strand	Gene strand
ENSBTAT00000033843	ENSBTAG00000017566	*TUFT1*	3:19,238,265–19,289,315	Forward	Reverse
ENSBTAT00000045699	ENSBTAG00000010394	*MCF2L2*	1:84,324,970–84,525,526	Reverse	Forward
ENSBTAT00000064288	ENSBTAG00000000664	*SLC22A15*	3:27,378,643–27,481,821	Reverse	Reverse

Three lncRNAs were identified to overlap with other genes in position. The transcription directions of the nearby genes and lncRNA were compared.

*TUFT1*, tuftelin 1; *MCF2L2*, MCF.2 cell line derived transforming sequence-like 2; *SLC22A15*, solute carrier family 22 member 15.
