# Supplementary material for: Comparison of characteristics of long noncoding RNA in Hanwoo according to sex
Source: Asian-Australas J Anim Sci. 2019 Oct 22;33(5):696–703. doi: 10.5713/ajas.18.0533 (PMC7206396; doi:10.5713/ajas.18.0533)
Supplement: Supplementary file 1 [file ajas-18-0533-suppl.pdf]

CERTIFICATE OF EDITING

HARRISCO
TOP EDITING COMPANY FOR
ACADEMIC RESEARCH PAPERS

Scientific English Research Paper Editing Service

1108, Hwanghwa B/D, 832-7, Yeoksam-Dong Kangnam-Ku

Seoul, the Republic of Korea

Tel : 82-2-557-1810~1

http://en.harrisco.net

The following manuscript was proofread and edited by
the professional English editors at HARRISCO.

Manuscript Title :

Comparison of characterization long noncoding RNA in Hanwoo according to sex

Manuscript Authors :

Jae-Don Oh

Date of Issue: :

July 03, 2018

Yours truly,

HARRISCO

Bull

GeneNames	GeneAcc	GeneName	Chr	Start	End	Strand	Type	log2(Fold_	p-value
ENSBTAT00000012797	ENSBTAG00000007090	MYH2	19	30137767	30165109	-	CODING	0.991991	0
ENSBTAT00000029890	ENSBTAG00000022158	TNNT3	29	50218484	50233948	-	CODING	-2.75753	0
ENSBTAT00000060569	ENSBTAG00000043561	COX1	MT	5687	7231	+	CODING	-1.13635	0
ENSBTAT00000060547	ENSBTAG00000043568	MT-ND3	MT	9823	10168	+	CODING	-0.53244	0
ENSBTAT00000059949	ENSBTAG00000042957	U6	5	71251611	71251717	-	CODING	13.16231	0
ENSBTAT00000059596	ENSBTAG00000042604	U6	14	83907053	83907159	-	CODING	-1.89875	0
ENSBTAT00000048981	ENSBTAG00000034580	TMSB4	GJ05	35911	36045	+	CODING	-2.13554	0
ENSBTAT00000060543	ENSBTAG00000043559	MT-ND4L	MT	10239	10535	+	CODING	-0.40979	0
ENSBTAT00000060327	ENSBTAG00000043335	U6	14	64324526	64324632	-	CODING	0.706826	0
ENSBTAT00000009327	ENSBTAG00000018204	MYH1	19	30110728	30134757	-	CODING	-1.82851	0
ENSBTAT00000006532	ENSBTAG00000046332	ACTA1	28	419154	421910	-	CODING	-0.51397	0
ENSBTAT00000008371	ENSBTAG00000022158	TNNT3	29	50218484	50233948	-	CODING	-0.11195	3.86E-289
ENSBTAT00000043649	ENSBTAG00000011392	MYBPC1	5	65737956	65840833	+	CODING	0.094735	2.17E-272
ENSBTAT00000063726	ENSBTAG00000046001	U6	19	43534127	43534233	-	CODING	-12.096	6.18E-231
ENSBTAT00000059974	ENSBTAG00000042982	U6	26	42369695	42369801	-	CODING	-12.096	6.18E-231
ENSBTAT00000059165	ENSBTAG00000042173	U6	15	54044311	54044418	-	CODING	11.54987	5.57E-229
ENSBTAT00000061306	ENSBTAG00000009703	MYH7	10	21325414	21345624	+	CODING	-0.02783	2.34E-205
ENSBTAT00000060175	ENSBTAG00000043183	U6	6	1.16E+08	1.16E+08	-	CODING	-11.8546	1.17E-203
ENSBTAT00000024749	ENSBTAG00000018598	HSPB6	18	46654243	46656762	-	CODING	-0.30638	3.10E-197
ENSBTAT00000014452	ENSBTAG00000010880	TNNI2	29	50285049	50287648	-	CODING	-1.27142	4.78E-196
ENSBTAT00000044397	ENSBTAG00000011969	HSPB1	25	34858435	34861074	+	CODING	-1.4947	3.15E-173
ENSBTAT00000013778	ENSBTAG00000010880	TNNI2	29	50285049	50287648	-	CODING	-1.63975	3.32E-170
ENSBTAT00000065799	ENSBTAG00000048229	TPT1	25	1394201	1417716	-	CODING	-1.22062	9.68E-170
ENSBTAT00000038879	ENSBTAG00000014069	PDK4	4	12754202	12767677	-	CODING	-2.10806	2.98E-161
ENSBTAT00000046179	ENSBTAG00000011424	TPM2	8	60267452	60274815	-	CODING	-0.27664	2.35E-160
ENSBTAT00000059121	ENSBTAG00000042129	U6	2	1.28E+08	1.28E+08	-	CODING	-11.374	3.34E-158
ENSBTAT00000037753	ENSBTAG00000014731	GAPDH	5	1.04E+08	1.04E+08	-	CODING	-0.45056	8.91E-155
ENSBTAT00000060571	ENSBTAG00000043558	ND1	MT	3101	4056	+	CODING	-0.60152	1.04E-142
ENSBTAT00000034732	ENSBTAG00000024929	PPP1R27	19	51658342	51659508	+	CODING	-1.69629	4.55E-140
ENSBTAT00000063025	ENSBTAG00000047231	TNNI1	16	49293852	49303508	-	CODING	-1.51196	7.42E-133
ENSBTAT00000009302	ENSBTAG00000007415	SLC7A8	10	21521555	21573888	+	CODING	-3.36389	4.86E-131
ENSBTAT00000033450	ENSBTAG00000013860	GADD45A	3	77972153	77975265	-	CODING	-3.49847	3.52E-125
ENSBTAT00000060549	ENSBTAG00000043556	COII	MT	7374	8057	+	CODING	-0.94367	7.27E-124
ENSBTAT00000015579	ENSBTAG00000011730	TCAP	19	40691138	40692346	+	CODING	-1.29285	4.61E-117
ENSBTAT00000060540	ENSBTAG00000043570	-	MT	1453	3023	+	CODING	-1.41226	2.24E-107
ENSBTAT00000007429	ENSBTAG00000005654	TMSB10	11	49933204	49934214	-	CODING	-1.83767	2.70E-96
ENSBTAT00000023600	ENSBTAG00000017743	XIRP2	2	29080500	29131828	-	CODING	0.343299	1.45E-93
ENSBTAT00000036194	ENSBTAG00000025644	CALM	10	1.03E+08	1.03E+08	+	CODING	-1.62262	1.39E-91
ENSBTAT00000047341	ENSBTAG00000033304	C27H8orf4	27	34893819	34895119	+	CODING	-2.57404	1.23E-89
ENSBTAT00000008593	ENSBTAG00000006541	ATP2A1	25	26188434	26204655	-	CODING	-0.3155	4.41E-89
ENSBTAT00000006918	ENSBTAG00000005259	UCP3	15	54213566	54224051	-	CODING	-1.89083	2.01E-83
ENSBTAT00000000556	ENSBTAG00000000434	CRYAB	15	22566929	22570256	-	CODING	-1.03938	2.91E-75
ENSBTAT00000046662	ENSBTAG00000013264	RPS24	28	33926497	33931777	+	CODING	-1.35109	1.57E-74
ENSBTAT00000000901	ENSBTAG00000000678	CSDE1	3	28695271	28723718	+	CODING	-0.05415	1.03E-73
ENSBTAT00000026932	ENSBTAG00000020223	CASQ1	3	9591852	9601502	-	CODING	-0.35081	1.83E-72
ENSBTAT00000008420	ENSBTAG00000006419	TNNT1	18	62725898	62735263	+	CODING	-1.24736	5.15E-71
ENSBTAT00000018799	ENSBTAG00000014143	ASB5	27	6692314	6736965	-	CODING	-1.53	1.14E-70
ENSBTAT00000008967	ENSBTAG00000006823	CMYA5	10	10658029	10711354	+	CODING	0.019311	1.35E-70
ENSBTAT00000011047	ENSBTAG00000008394	MYL3	22	53202766	53208551	+	CODING	-0.04685	1.08E-68
ENSBTAT00000063142	ENSBTAG00000046289	U6	27	6982216	6982322	-	CODING	0.005273	1.93E-68
ENSBTAT00000046421	ENSBTAG00000032719	TFRC	1	71260068	71280648	-	CODING	1.187228	2.03E-68
ENSBTAT00000060567	ENSBTAG00000043550	MT-CYB	MT	14514	15653	+	CODING	-0.67667	1.27E-67
ENSBTAT00000028662	ENSBTAG00000021508	LMOD3	22	32534126	32550296	+	CODING	0.014224	7.24E-66
ENSBTAT00000001838	ENSBTAG00000001398	ATP2A2	17	56458750	56512895	+	CODING	-0.17752	3.35E-63
ENSBTAT00000016359	ENSBTAG00000012330	B2M	10	1.04E+08	1.04E+08	+	CODING	-1.34358	8.85E-63
ENSBTAT00000034373	ENSBTAG00000023659	MT2	18	24125366	24126333	-	CODING	-2.70655	1.41E-62
ENSBTAT00000036043	ENSBTAG00000005974	APOBEC2	23	14985521	14998187	+	CODING	-0.03169	2.06E-62
ENSBTAT00000043764	ENSBTAG00000002094	ATP5J2	25	37494182	37499276	+	CODING	-1.52817	2.59E-62
ENSBTAT00000001373	ENSBTAG00000001032	PYGM	29	43606012	43617848	-	CODING	-0.3893	3.09E-61

ENSBTAT00000027850	ENSBTAG00000020905	RPL11	2	1.3E+08	1.3E+08	-	CODING	-1.69557	3.55E-61
ENSBTAT00000018267	ENSBTAG00000013744	SYNPO	7	64020057	64027999	+	CODING	0.189332	1.97E-60
ENSBTAT00000009604	ENSBTAG00000007300	FHL3	3	1.09E+08	1.09E+08	+	CODING	0.070694	5.44E-60
ENSBTAT00000065038	ENSBTAG00000046725	TNNC2	13	75316423	75318650	-	CODING	-0.93652	1.39E-58
ENSBTAT00000003943	ENSBTAG00000003033	GADD45G	8	90017453	90018996	-	CODING	-3.41003	3.91E-58
ENSBTAT00000008518	ENSBTAG00000006491	AGL	3	43504601	43585149	-	CODING	0.381895	5.68E-58
ENSBTAT00000008434	ENSBTAG00000006434	SYNPO2	6	7388728	7590933	-	CODING	0.335456	7.27E-58
ENSBTAT00000015883	ENSBTAG00000011969	HSPB1	25	34858438	34861035	+	CODING	1.219193	2.09E-55
ENSBTAT00000038488	ENSBTAG00000013631	GLUL	16	64948332	64958885	-	CODING	-1.47941	5.34E-55
ENSBTAT00000065039	ENSBTAG00000046623	-	13	64372559	64372993	-	CODING	-1.39344	1.10E-53
ENSBTAT00000061284	ENSBTAG00000001564	PDE4DIP	3	22916551	23063522	-	CODING	0.002463	2.72E-52
ENSBTAT00000004190	ENSBTAG00000003229	RPL23	19	40075000	40079360	-	CODING	-1.39674	4.17E-52
ENSBTAT00000059374	ENSBTAG00000042382	U6	28	41535977	41536083	+	CODING	-1.44235	3.21E-51
ENSBTAT00000043425	ENSBTAG00000019011	PGM1	3	82250292	82315999	-	CODING	-0.21574	4.66E-51
ENSBTAT00000022375	ENSBTAG00000016819	FABP3	2	1.23E+08	1.23E+08	+	CODING	-1.44257	1.07E-50
ENSBTAT00000016414	ENSBTAG00000012370	MGP	5	95456444	95459983	+	CODING	-2.31442	1.75E-50
ENSBTAT00000037900	ENSBTAG00000005085	TRIM63	2	1.28E+08	1.28E+08	+	CODING	-1.86375	1.30E-49
ENSBTAT00000044796	ENSBTAG00000005373	TPM1	10	47056204	47065846	-	CODING	-1.04299	1.75E-49
ENSBTAT00000006781	ENSBTAG00000005142	RPL37	20	33667205	33669805	+	CODING	-1.29754	2.21E-49
ENSBTAT00000018491	ENSBTAG00000013924	RPS11	18	56404237	56406668	+	CODING	-1.30585	3.76E-48
ENSBTAT00000066266	ENSBTAG00000008573	ZFP36	18	49361497	49375317	+	CODING	-1.7924	1.75E-47
ENSBTAT00000025803	ENSBTAG00000019368	IGFBP7	6	74071067	74150456	-	CODING	-1.90261	3.96E-47
ENSBTAT00000064903	ENSBTAG00000006419	TNNT1	18	62725898	62735263	+	CODING	-1.36373	4.60E-47
ENSBTAT00000015606	ENSBTAG00000011752	SYNM	21	7768429	7793372	-	CODING	0.393967	6.34E-47
ENSBTAT00000017500	ENSBTAG00000025441	HSPA1A	23	27331773	27333698	-	CODING	-1.44708	1.17E-46
ENSBTAT00000045476	ENSBTAG00000032057	-	3	28623391	28623702	-	CODING	-2.16995	1.69E-46
ENSBTAT00000065403	ENSBTAG00000018707	LDB3	28	41657195	41713873	+	CODING	-0.15417	5.53E-45
ENSBTAT00000065672	ENSBTAG00000038430	-	23	34219298	34219825	-	CODING	-2.38468	5.20E-44
ENSBTAT00000015584	ENSBTAG00000011734	ANKRD1	26	12571209	12580580	-	CODING	-1.34809	6.59E-44
ENSBTAT00000020243	ENSBTAG00000015214	CA3	14	79406494	79416487	-	CODING	-0.44805	3.05E-43
ENSBTAT00000005329	ENSBTAG00000004079	ZNF106	10	37890185	37941980	-	CODING	0.233256	4.57E-43
ENSBTAT00000009965	ENSBTAG00000013343	FTL	18	55992910	55994741	+	CODING	-2.61384	4.02E-42
ENSBTAT00000026756	ENSBTAG00000020080	MYBPC2	18	57039094	57064720	+	CODING	-0.40567	7.02E-42
ENSBTAT00000006990	ENSBTAG00000005315	RPS6	2	1.12E+08	1.12E+08	-	CODING	-1.55648	1.51E-41
ENSBTAT00000020326	ENSBTAG00000015285	RPS8	3	1.02E+08	1.02E+08	-	CODING	-1.35838	9.39E-41
ENSBTAT00000002642	ENSBTAG00000002038	RPL14	22	13336573	13339983	+	CODING	-1.38853	1.05E-40
ENSBTAT00000026259	ENSBTAG00000019701	RPL31	11	6003175	6008528	+	CODING	-1.23786	1.33E-40
ENSBTAT00000030030	ENSBTAG00000022244	ACTN3	29	45230682	45242282	+	CODING	-0.32166	7.42E-40
ENSBTAT00000004044	ENSBTAG00000003109	ITM2B	12	18114553	18139506	+	CODING	-1.44178	8.93E-40
ENSBTAT00000019583	ENSBTAG00000014718	CST6	29	44766936	44768173	+	CODING	-2.57247	1.40E-39
ENSBTAT00000019184	ENSBTAG00000014423	-	16	47992813	48000514	+	CODING	-1.57917	2.57E-39
ENSBTAT00000008422	ENSBTAG00000022158	TNNT3	29	50218484	50233948	-	CODING	-12.0695	3.22E-39
ENSBTAT00000044763	ENSBTAG00000031573	ITGB1BP3	7	21290813	21294189	-	CODING	-1.33712	1.48E-38
ENSBTAT00000048100	ENSBTAG00000025258	-	19	14514200	14548145	-	CODING	-5.81482	1.59E-38
ENSBTAT00000035803	ENSBTAG00000025462	GADD45B	7	22411968	22414079	-	CODING	-3.04446	2.51E-38
ENSBTAT00000060546	ENSBTAG00000043567	-	MT	431	1385	+	CODING	-1.17746	3.19E-38
ENSBTAT00000027713	ENSBTAG00000020795	RPS21	13	55358720	55359979	-	CODING	-1.52705	1.04E-37
ENSBTAT00000000187	ENSBTAG00000000163	DDIT4	28	28483403	28485410	+	CODING	-1.85179	6.25E-37
ENSBTAT00000012735	ENSBTAG00000009663	CSDA	5	99335508	99360895	+	CODING	-1.11459	1.17E-35
ENSBTAT00000046493	ENSBTAG00000023600	APOD	1	72670963	72684269	+	CODING	-2.05858	1.52E-35
ENSBTAT00000060250	ENSBTAG00000043258	SNORA18	29	1064655	1064785	+	CODING	-8.65279	2.12E-35
ENSBTAT00000065060	ENSBTAG00000046551	SNORA32	25	33716083	33716205	-	CODING	-8.63691	4.43E-35
ENSBTAT00000000079	ENSBTAG00000037526	FABP4	14	46833665	46838053	-	CODING	-2.51069	4.78E-35
ENSBTAT00000020323	ENSBTAG00000015283	RPL32	22	56985249	56989012	+	CODING	-1.29682	5.28E-35
ENSBTAT00000001791	ENSBTAG00000001360	RPS12	9	71974860	71978200	+	CODING	-1.25887	1.16E-34
ENSBTAT00000046011	ENSBTAG00000032436	UBC	17	53142511	53143425	+	CODING	-0.30489	1.79E-34
ENSBTAT00000014486	ENSBTAG00000010907	PPP1R1A	5	25627052	25631242	+	CODING	-1.16632	8.11E-34
ENSBTAT00000014219	ENSBTAG00000010741	KBTBD10	2	26780742	26798046	-	CODING	-0.48018	1.08E-33
ENSBTAT00000019176	ENSBTAG00000014417	-	25	33157514	33168430	+	CODING	0.156067	1.33E-33
ENSBTAT00000055075	ENSBTAG00000011765	GABARAPL1	5	1E+08	1E+08	-	CODING	-2.13828	1.45E-33
ENSBTAT00000064621	ENSBTAG00000046307	-	14	20738814	20740407	+	CODING	-3.07965	1.66E-33

ENSBTAT00000011846	ENSBTAG00000046303	-	11	98509474	98513781	+	CODING	-2.05829	3.87E-33
ENSBTAT00000024444	ENSBTAG00000018369	MYL2	17	56953813	56961603	-	CODING	-0.95449	4.18E-33
ENSBTAT00000029208	ENSBTAG00000014614	ACTA2	26	10662363	10679648	-	CODING	-1.95889	6.37E-33
ENSBTAT00000003997	ENSBTAG00000003074	SLC16A6	19	62486092	62496100	+	CODING	-3.0745	2.89E-32
ENSBTAT00000000101	ENSBTAG00000000092	HIF1AN	26	21291076	21300157	+	CODING	0.155105	3.89E-32
ENSBTAT00000017580	ENSBTAG00000013208	SLC25A4	27	14546020	14550037	+	CODING	-1.1109	1.14E-31
ENSBTAT00000019411	ENSBTAG00000014583	CALM	18	54169313	54178928	+	CODING	-0.25782	1.42E-31
ENSBTAT00000006534	ENSBTAG00000046332	ACTA1	28	419156	421250	-	CODING	-0.2286	1.56E-31
ENSBTAT00000017030	ENSBTAG00000012818	PDLIM5	6	31419270	31567339	-	CODING	-0.28926	2.24E-31
ENSBTAT00000003962	ENSBTAG00000005620	RPS3	15	55370367	55375306	+	CODING	-1.31592	4.77E-31
ENSBTAT00000022183	ENSBTAG00000016683	BIKBA	21	46065549	46068942	-	CODING	-2.43113	2.72E-30
ENSBTAT00000009469	ENSBTAG00000007196	TAGLN	15	28318411	28323666	+	CODING	-1.67694	5.23E-30
ENSBTAT00000050559	ENSBTAG00000036078	EMP1	5	97081921	97101600	-	CODING	-2.12196	6.11E-30
ENSBTAT00000025484	ENSBTAG00000019147	RPS20	14	24955079	24956324	-	CODING	-1.61718	2.23E-29
ENSBTAT00000042753	ENSBTAG00000030278	EGFL7	11	1.04E+08	1.04E+08	+	CODING	-2.0727	3.15E-29
ENSBTAT00000015924	ENSBTAG00000012003	PPIA	4	77409433	77413143	-	CODING	-2.16622	6.65E-29
ENSBTAT00000059463	ENSBTAG00000042471	SNORA25	29	1067324	1067450	+	CODING	-8.29566	7.98E-29
ENSBTAT00000001993	ENSBTAG00000001521	UQCRB	14	70329414	70334124	+	CODING	-1.18372	1.58E-28
ENSBTAT00000065455	ENSBTAG00000047155	C10orf71	28	43930428	43934735	+	CODING	0.251378	1.74E-28
ENSBTAT00000038460	ENSBTAG00000026886	MP68	21	70113045	70118400	-	CODING	-1.56811	5.70E-28
ENSBTAT00000024904	ENSBTAG00000018707	LDB3	28	41657195	41687361	+	CODING	-0.46438	6.08E-28
ENSBTAT00000021587	ENSBTAG00000016224	RPS7	8	1.13E+08	1.13E+08	-	CODING	-1.36347	6.40E-28
ENSBTAT00000024641	ENSBTAG00000018513	FHL1	X	19809635	19820952	+	CODING	-0.65197	9.70E-28
ENSBTAT00000004519	ENSBTAG00000003476	FEM1A	7	20565301	20568880	-	CODING	-0.08874	1.29E-27
ENSBTAT00000014398	ENSBTAG00000010849	ANKRD23	11	2768909	2773197	-	CODING	-0.22712	1.35E-27
ENSBTAT00000063883	ENSBTAG00000045728	SCD	26	21141592	21148318	-	CODING	0.61914	1.53E-27
ENSBTAT00000005486	ENSBTAG00000004189	MLXIP	17	55472596	55528501	-	CODING	0.622141	2.37E-27
ENSBTAT00000019758	ENSBTAG00000014835	SPARC	7	64878194	64900828	-	CODING	-1.40482	2.80E-27
ENSBTAT00000008669	ENSBTAG00000006607	CCNG1	7	77249997	77257261	+	CODING	-0.2952	5.11E-27
ENSBTAT00000064843	ENSBTAG00000047174	-	22	52425504	52428832	+	CODING	0.070138	1.68E-26
ENSBTAT00000014038	ENSBTAG00000010619	PPP3R1	11	66582636	66642276	-	CODING	0.021265	1.89E-26
ENSBTAT00000022189	ENSBTAG00000016688	-	X	66722399	66723397	-	CODING	-0.50491	2.61E-26
ENSBTAT00000023087	ENSBTAG00000017363	SAT1	X	1.26E+08	1.26E+08	-	CODING	-2.48658	3.72E-26
ENSBTAT00000000814	ENSBTAG00000000622	RPS17	21	23301663	23305104	-	CODING	-1.15738	4.92E-26
ENSBTAT00000019875	ENSBTAG00000014930	MYLK2	13	61900820	61915380	+	CODING	-0.13772	5.05E-26
ENSBTAT00000028093	ENSBTAG00000021093	RPS16	18	49393725	49396191	-	CODING	-1.36504	7.40E-26
ENSBTAT00000064624	ENSBTAG00000045783	-	16	5201143	5201319	-	CODING	0.338538	2.26E-25
ENSBTAT00000026928	ENSBTAG00000020219	MSS51	28	29578510	29588920	-	CODING	0.632354	3.45E-25
ENSBTAT00000046158	ENSBTAG00000032531	MUSTN1	22	48611898	48613043	+	CODING	-1.13068	4.52E-25
ENSBTAT00000002229	ENSBTAG00000039335	ARRDC2	7	5101428	5105930	-	CODING	-2.23093	4.62E-25
ENSBTAT00000026278	ENSBTAG00000019718	RPS15	7	45465834	45467519	+	CODING	-1.31167	1.10E-24
ENSBTAT00000020701	ENSBTAG00000015582	HMOX1	5	73980776	73987841	+	CODING	-2.15134	1.25E-24
ENSBTAT00000012544	ENSBTAG00000009535	RPS2	25	1520493	1522670	-	CODING	-1.11805	1.42E-24
ENSBTAT00000061127	ENSBTAG00000044105	FOXO1	12	21915747	22005338	+	CODING	-2.86581	1.60E-24
ENSBTAT00000009228	ENSBTAG00000006999	RYR1	18	48502352	48631056	+	CODING	-0.16391	2.72E-24
ENSBTAT00000008851	ENSBTAG00000006733	PPP1R3A	4	54866421	54906096	+	CODING	-0.211	4.20E-24
ENSBTAT00000019318	ENSBTAG00000014534	EEF1A1	9	13233554	13236949	-	CODING	-1.58127	2.35E-23
ENSBTAT00000059118	ENSBTAG00000042126	SNORA66	16	37582336	37582467	+	CODING	7.220576	3.69E-23
ENSBTAT00000006190	ENSBTAG00000025313	DYNLL2	19	9118008	9125251	+	CODING	0.116107	4.71E-23
ENSBTAT00000015235	ENSBTAG00000011465	MYBPH	16	760056	768350	-	CODING	-2.45169	5.70E-23
ENSBTAT00000024572	ENSBTAG00000018463	VIM	13	31945012	31952941	+	CODING	-1.44209	6.19E-23
ENSBTAT00000005007	ENSBTAG00000005296	RPL13A	18	56394558	56398082	+	CODING	-1.21814	6.61E-23
ENSBTAT00000001780	ENSBTAG00000001349	KIF1C	19	27014446	27043780	-	CODING	-0.1328	7.11E-23
ENSBTAT00000060548	ENSBTAG00000043571	ND2	MT	4266	5307	+	CODING	-0.70988	7.15E-23
ENSBTAT00000002790	ENSBTAG00000002157	LMOD2	4	88746219	88755270	+	CODING	-0.26928	1.21E-22
ENSBTAT00000060554	ENSBTAG00000043553	GPX3	7	64286948	64295116	+	CODING	-1.8982	1.72E-22
ENSBTAT00000010744	ENSBTAG00000008172	EGLN3	21	44805298	44834310	-	CODING	0.192894	1.81E-22
ENSBTAT00000018147	ENSBTAG00000013653	NFE2L1	19	39056971	39069122	-	CODING	-0.15394	1.91E-22
ENSBTAT00000014529	ENSBTAG00000010940	HSPB7	2	1.37E+08	1.37E+08	+	CODING	-0.31951	3.26E-22
ENSBTAT00000022510	ENSBTAG00000016924	CAP2	23	39646543	39781557	-	CODING	-0.10874	5.60E-22
ENSBTAT00000007709	ENSBTAG00000005865	MAPK6	10	58437865	58449901	-	CODING	-0.24843	5.73E-22

ENSBTAT00000016957	ENSBTAG00000012760	NDUFB3	2	90077970	90088465	+	CODING	-1.51092	5.97E-22
ENSBTAT00000015892	ENSBTAG00000011976	CYP4B1	3	99937185	99957408	-	CODING	-6.12628	6.56E-22
ENSBTAT00000062760	ENSBTAG00000045327	ACA64	25	1522971	1523097	+	CODING	-7.81064	9.80E-22
ENSBTAT00000014306	ENSBTAG00000010799	MYL6	5	57486017	57489133	-	CODING	-1.7542	1.17E-21
ENSBTAT00000013871	ENSBTAG00000010500	-	28	3323730	3333934	+	CODING	-0.02663	1.63E-21
ENSBTAT00000013713	ENSBTAG00000010389	FAM47E-STB	6	92967767	92971371	+	CODING	0.147513	1.65E-21
ENSBTAT00000059953	ENSBTAG00000042961	U6	19	57276185	57276291	-	CODING	-0.50024	2.35E-21
ENSBTAT00000023273	ENSBTAG00000017509	MYPN	28	24745815	24832856	+	CODING	0.176867	2.54E-21
ENSBTAT00000054202	ENSBTAG00000011593	QKI	9	1E+08	1E+08	+	CODING	-0.12544	3.30E-21
ENSBTAT00000013247	ENSBTAG00000027787	-	18	65621142	65621509	+	CODING	-1.92394	4.11E-21
ENSBTAT00000064506	ENSBTAG00000022158	TNNT3	29	50218484	50233948	-	CODING	12.10908	5.56E-21
ENSBTAT00000029307	ENSBTAG00000021978	PARVB	5	1.16E+08	1.16E+08	+	CODING	0.181912	8.31E-21
ENSBTAT00000064053	ENSBTAG00000047957	SCD	26	21132751	21133969	+	CODING	0.656876	9.74E-21
ENSBTAT00000064497	ENSBTAG00000046100	-	26	14093095	14093609	+	CODING	-1.57329	1.18E-20
ENSBTAT00000014744	ENSBTAG00000011104	RTN4	11	37575270	37611627	-	CODING	-0.44819	1.37E-20
ENSBTAT00000015186	ENSBTAG00000011424	TPM2	8	60268618	60274720	-	CODING	-0.88817	1.57E-20
ENSBTAT00000044290	ENSBTAG00000006712	KIAA0368	8	1.03E+08	1.03E+08	-	CODING	0.042047	2.09E-20
ENSBTAT00000005279	ENSBTAG00000004037	JUN	3	87841042	87843087	+	CODING	-1.49456	2.25E-20
ENSBTAT00000004562	ENSBTAG00000003505	DCN	5	21080013	21119087	-	CODING	-1.46925	3.13E-20
ENSBTAT00000042757	ENSBTAG00000030281	-	11	1.04E+08	1.04E+08	-	CODING	-1.39339	4.19E-20
ENSBTAT00000002556	ENSBTAG00000039682	MTUS1	27	18632852	18698056	+	CODING	-3.02069	4.52E-20
ENSBTAT00000063744	ENSBTAG00000046450	EIF1	3	60346812	60347472	+	CODING	-0.42863	6.17E-20
ENSBTAT00000059592	ENSBTAG00000042600	SNORD94	11	48600030	48600165	-	CODING	6.943464	7.44E-20
ENSBTAT00000017182	ENSBTAG00000012931	PLN	9	32823612	32834377	-	CODING	-0.32511	7.48E-20
ENSBTAT00000063728	ENSBTAG00000046024	PPP3CB	28	29592229	29602016	-	CODING	0.313092	8.03E-20
ENSBTAT00000044517	ENSBTAG00000002783	PCYOX1	11	68560573	68572075	+	CODING	0.14702	8.32E-20
ENSBTAT00000014839	ENSBTAG00000011173	FAM189A2	8	45784659	45816860	+	CODING	0.457615	8.43E-20
ENSBTAT00000019285	ENSBTAG00000014508	FBXO40	1	66712457	66717255	+	CODING	0.132214	8.50E-20
ENSBTAT00000001720	ENSBTAG00000001303	HSPB8	17	58405437	58418688	-	CODING	-1.11897	9.34E-20
ENSBTAT00000008373	ENSBTAG00000006383	-	X	1.26E+08	1.26E+08	+	CODING	-1.45472	1.07E-19
ENSBTAT00000020036	ENSBTAG00000015053	CFL2	21	45519654	45522305	-	CODING	-0.43267	1.11E-19
ENSBTAT00000040046	ENSBTAG00000027772	RPS25	15	30115928	30117641	-	CODING	-1.31329	1.25E-19
ENSBTAT00000052009	ENSBTAG00000027444	SVIL	13	34860211	34965892	-	CODING	0.25336	1.40E-19
ENSBTAT00000050064	ENSBTAG00000000223	PPM1B	11	26402682	26427596	+	CODING	-0.052	1.75E-19
ENSBTAT00000003305	ENSBTAG00000002549	PTMA	2	1.2E+08	1.2E+08	+	CODING	-1.72065	2.76E-19
ENSBTAT00000021808	ENSBTAG00000016401	OPTN	13	28078762	28117627	+	CODING	-0.24551	5.04E-19
ENSBTAT00000000506	ENSBTAG00000000393	SSPN	5	84096324	84138430	-	CODING	0.578685	5.84E-19
ENSBTAT00000027457	ENSBTAG00000020605	SMTNL2	19	25702553	25724157	-	CODING	-0.24093	6.55E-19
ENSBTAT00000044622	ENSBTAG00000031483	-	29	40214778	40215227	-	CODING	-1.48498	6.57E-19
ENSBTAT00000020867	ENSBTAG00000015720	WWP1	14	78599363	78699784	-	CODING	-0.02569	8.54E-19
ENSBTAT00000013301	ENSBTAG00000027075	-	21	14304505	14304970	+	CODING	-1.29988	8.72E-19
ENSBTAT00000016865	ENSBTAG00000001842	GSTM3	3	33768050	33770824	+	CODING	-2.23131	8.91E-19
ENSBTAT00000020719	ENSBTAG00000015598	RPS10	23	8434576	8441522	-	CODING	-1.19742	1.14E-18
ENSBTAT00000061390	ENSBTAG00000005946	USP13	1	87949654	88075267	-	CODING	0.212767	1.25E-18
ENSBTAT00000028282	ENSBTAG00000021227	ATP5H	19	57020084	57024849	+	CODING	-1.22575	1.27E-18
ENSBTAT00000000926	ENSBTAG00000000694	TAF10	15	47073272	47074686	+	CODING	-1.8243	1.41E-18
ENSBTAT00000045994	ENSBTAG00000037778	CXCL3	6	90811062	90813079	+	CODING	-3.18769	1.50E-18
ENSBTAT00000026992	ENSBTAG00000021132	SYNPO2L	28	29799037	29805747	-	CODING	0.179526	1.64E-18
ENSBTAT00000025522	ENSBTAG00000019177	BIN1	2	5350654	5407851	+	CODING	-0.28494	2.97E-18
ENSBTAT00000002666	ENSBTAG00000002060	RPL19	19	40332947	40334703	+	CODING	-1.13057	3.46E-18
ENSBTAT00000011795	ENSBTAG00000020116	JSP.1	23	28469735	28473401	-	CODING	-1.63472	3.72E-18
ENSBTAT00000000714	ENSBTAG00000000546	TOB1	19	36512064	36515184	+	CODING	-0.29085	4.51E-18
ENSBTAT00000026009	ENSBTAG00000019525	-	28	29613180	29630076	+	CODING	0.109511	8.70E-18
ENSBTAT00000008388	ENSBTAG00000006398	TOMM7	9	89692607	89692771	-	CODING	-1.74695	1.22E-17
ENSBTAT00000004952	ENSBTAG00000003798	AMFR	18	24290808	24334739	+	CODING	0.132322	1.26E-17
ENSBTAT00000049790	ENSBTAG00000013479	SLC9A3R2	25	1581874	1589619	+	CODING	-2.69001	1.48E-17
ENSBTAT00000039655	ENSBTAG00000010677	LIMCH1	6	61872553	62222095	+	CODING	0.462716	1.53E-17
ENSBTAT00000017710	ENSBTAG00000013315	ATP5B	5	57119917	57125290	+	CODING	-0.47645	1.77E-17
ENSBTAT00000014801	ENSBTAG00000011145	NDUFA4	4	18973421	18980828	-	CODING	-1.0777	2.92E-17
ENSBTAT00000007041	ENSBTAG00000005353	DES	2	1.08E+08	1.08E+08	+	CODING	-0.62714	3.16E-17
ENSBTAT00000005348	ENSBTAG00000004094	SPARCL1	6	1.04E+08	1.04E+08	-	CODING	-1.51883	5.79E-17

ENSBTAT00000065632	ENSBTAG00000046512	CMYA1	22	12549676	12558895	-	CODING	-1.65933	6.58E-17
ENSBTAT00000021880	ENSBTAG00000016457	FXR1	1	86678925	86751963	-	CODING	-0.20647	6.81E-17
ENSBTAT00000019527	ENSBTAG00000014667	PAIP2B	11	13393546	13397285	+	CODING	0.423966	7.89E-17
ENSBTAT00000006866	ENSBTAG00000005211	RPL4	10	13318875	13323526	-	CODING	-1.18865	7.93E-17
ENSBTAT00000047320	ENSBTAG00000012044	RPL13	18	14533161	14535556	+	CODING	-1.38462	8.76E-17
ENSBTAT00000059955	ENSBTAG00000042963	SNORA8	29	1065601	1065737	+	CODING	6.641945	8.80E-17
ENSBTAT00000052289	ENSBTAG00000039728	RPLP1	16	52249583	52372101	-	CODING	-1.06375	9.66E-17
ENSBTAT00000028036	ENSBTAG00000021048	ADM	15	42911044	42913326	-	CODING	-3.55477	1.03E-16
ENSBTAT00000024262	ENSBTAG00000018229	NFIX	7	13596367	13658112	-	CODING	0.324171	1.09E-16
ENSBTAT00000005327	ENSBTAG00000004077	YWHAG	25	34884283	34906639	-	CODING	-0.26767	1.23E-16
ENSBTAT00000025308	ENSBTAG00000019011	PGM1	3	82250295	82288338	-	CODING	0.229173	1.42E-16
ENSBTAT00000009575	ENSBTAG00000007281	SEMA6C	3	19717720	19728131	+	CODING	0.726613	1.60E-16
ENSBTAT00000060242	ENSBTAG00000043250	7SK	23	24977642	24977972	+	CODING	-2.4443	1.79E-16
ENSBTAT00000029092	ENSBTAG00000021823	HFE2	3	21470721	21474444	+	CODING	0.296124	2.20E-16
ENSBTAT00000017177	ENSBTAG00000012927	ALDOA	25	26470488	26475202	-	CODING	-0.89844	2.28E-16
ENSBTAT00000016154	ENSBTAG00000012178	NR1D1	19	41040926	41048228	-	CODING	0.580327	2.46E-16
ENSBTAT00000060287	ENSBTAG00000043295	SNORA13	10	1763369	1763505	-	CODING	6.579292	3.33E-16
ENSBTAT00000020452	ENSBTAG00000015388	RPL18	18	55710193	55713956	-	CODING	-1.21707	4.96E-16
ENSBTAT00000060438	ENSBTAG00000043446	SNORA51	3	15691547	15691677	-	CODING	-7.29355	5.01E-16
ENSBTAT00000024387	ENSBTAG00000038488	TMSB4	X	1.41E+08	1.41E+08	-	CODING	-1.4776	5.01E-16
ENSBTAT00000059254	ENSBTAG00000046842	U4	17	64875706	64875846	-	CODING	6.544195	6.87E-16
ENSBTAT00000012357	ENSBTAG00000009389	HNRNPH1	7	1593463	1602463	+	CODING	-1.35762	7.81E-16
ENSBTAT00000061386	ENSBTAG00000020296	UBR3	2	26361548	26565226	-	CODING	-0.16256	9.30E-16
ENSBTAT00000052050	ENSBTAG00000039555	COX7C	7	88648457	88650528	+	CODING	-1.64684	1.02E-15
ENSBTAT00000022929	ENSBTAG00000011022	ARPP19	10	57923246	57937867	+	CODING	-0.03722	1.65E-15
ENSBTAT00000060533	ENSBTAG00000043541	SNORA5	4	77227804	77227938	+	CODING	-7.23569	1.74E-15
ENSBTAT00000026293	ENSBTAG00000019730	SFRS18	9	51022633	51049299	+	CODING	-1.90053	2.57E-15
ENSBTAT00000018269	ENSBTAG00000013749	RHOQ	11	28834380	28871742	+	CODING	-0.1988	2.79E-15
ENSBTAT00000023150	ENSBTAG00000017416	KPNA4	1	1.08E+08	1.08E+08	+	CODING	-0.10181	3.22E-15
ENSBTAT00000028634	ENSBTAG00000021481	CA14	3	20420023	20427199	-	CODING	0.492512	3.32E-15
ENSBTAT00000018886	ENSBTAG00000014205	PRKAR2A	22	51658044	51727840	+	CODING	0.049225	4.09E-15
ENSBTAT00000034789	ENSBTAG00000019903	RAMP2	19	43441583	43443429	+	CODING	-1.68299	4.87E-15
ENSBTAT00000020148	ENSBTAG00000015145	S100A11	3	18768796	18770416	+	CODING	-1.71619	5.16E-15
ENSBTAT00000002525	ENSBTAG00000001941	SEMA4D	8	90136712	90262973	+	CODING	0.162257	5.88E-15
ENSBTAT00000020194	ENSBTAG00000015177	PRSS23	29	8788703	8797308	-	CODING	-0.00907	7.05E-15
ENSBTAT00000060566	ENSBTAG00000043560	COX3	MT	8970	9750	+	CODING	-0.76735	1.32E-14
ENSBTAT00000039686	ENSBTAG00000014884	HIPK3	15	64704079	64758178	+	CODING	0.08105	1.60E-14
ENSBTAT00000015261	ENSBTAG00000011483	SCARF1	19	23309862	23319829	-	CODING	-2.22961	1.75E-14
ENSBTAT00000003794	ENSBTAG00000002922	-	29	17833137	17887917	+	CODING	0.890447	1.86E-14
ENSBTAT00000037243	ENSBTAG00000020035	RCAN1	1	351708	362907	+	CODING	-1.50541	2.10E-14
ENSBTAT00000015387	ENSBTAG00000035081	SERINC2	2	1.23E+08	1.23E+08	+	CODING	0.039901	2.65E-14
ENSBTAT00000027930	ENSBTAG00000020969	IQWD1	3	587034	768209	-	CODING	-0.13955	2.79E-14
ENSBTAT00000000497	ENSBTAG00000000385	ZBTB18	16	33913963	33918059	+	CODING	0.36286	3.00E-14
ENSBTAT00000025270	ENSBTAG00000018987	RPS25	3	16507484	16507861	-	CODING	-1.24941	3.17E-14
ENSBTAT00000004658	ENSBTAG00000003581	SETD7	17	18463314	18515964	+	CODING	-0.21217	3.20E-14
ENSBTAT00000061582	ENSBTAG00000019585	MYOM1	24	37673546	37791756	-	CODING	-0.29683	4.04E-14
ENSBTAT00000012570	ENSBTAG00000009552	ATP2B1	5	19539973	19669793	-	CODING	0.23937	5.09E-14
ENSBTAT00000023981	ENSBTAG00000018016	NUPR1	25	26340079	26341408	-	CODING	-2.58972	6.15E-14
ENSBTAT00000027091	ENSBTAG00000020330	BTG1	5	22086071	22088781	-	CODING	-1.52361	6.19E-14
ENSBTAT00000010711	ENSBTAG00000008150	PKIA	14	43880469	43978277	+	CODING	-0.33266	7.23E-14
ENSBTAT00000001330	ENSBTAG00000001003	CKMT2	7	83554315	83579633	+	CODING	-1.05401	8.35E-14
ENSBTAT00000008431	ENSBTAG00000006429	ACO2	5	1.13E+08	1.13E+08	+	CODING	-0.35946	8.98E-14
ENSBTAT00000005660	ENSBTAG00000004322	FOS	10	86883739	86887169	+	CODING	-0.30145	9.93E-14
ENSBTAT00000006376	ENSBTAG00000004850	KPNA3	12	19339291	19385309	-	CODING	-0.03058	1.32E-13
ENSBTAT00000002160	ENSBTAG00000001648	RPL21	12	32852826	32859542	-	CODING	-1.33552	1.35E-13
ENSBTAT00000038022	ENSBTAG00000013235	TINAGL1	2	1.23E+08	1.23E+08	-	CODING	-2.11498	1.56E-13
ENSBTAT00000012317	ENSBTAG00000009359	-	15	38876122	38876667	-	CODING	-0.47125	1.66E-13
ENSBTAT00000018492	ENSBTAG00000013921	CKM	18	53383534	53392948	-	CODING	-0.90176	1.83E-13
ENSBTAT00000024243	ENSBTAG00000018214	SHISA2	12	33578134	33583714	+	CODING	0.710586	2.00E-13
ENSBTAT00000010213	ENSBTAG00000007767	TBX15	3	24315881	24359735	+	CODING	0.192875	2.21E-13
ENSBTAT00000023729	ENSBTAG00000017851	RXRA	11	1.06E+08	1.06E+08	+	CODING	0.536869	2.32E-13

ENSBTAT00000006476	ENSBTAG00000004922	AGPAT5	27	4679600	4727246	+	CODING	-0.13483	2.47E-13
ENSBTAT00000001429	ENSBTAG00000001078	SRL	25	3392668	3404630	-	CODING	-0.02489	2.67E-13
ENSBTAT00000045066	ENSBTAG00000031786	FAU	5	47736728	47737193	+	CODING	-1.20873	3.22E-13
ENSBTAT00000011863	ENSBTAG00000009012	PTX3	1	1.11E+08	1.11E+08	-	CODING	-5.41591	3.76E-13
ENSBTAT00000005580	ENSBTAG00000000425	RPL17	21	55609537	55610119	+	CODING	-1.14484	4.27E-13
ENSBTAT00000014505	ENSBTAG00000010919	USP47	15	41298533	41373143	-	CODING	-0.09938	4.74E-13
ENSBTAT00000065223	ENSBTAG00000018167	KLHL31	23	6783412	6787028	+	CODING	0.058063	5.58E-13
ENSBTAT00000061283	ENSBTAG00000016167	ATL2	11	20691386	20758963	-	CODING	0.004729	6.14E-13
ENSBTAT00000016346	ENSBTAG00000012317	PNP	10	26667693	26673918	-	CODING	-1.4424	6.61E-13
ENSBTAT00000059670	ENSBTAG00000042678	SNORA71	13	68003660	68003794	+	CODING	-6.92547	7.06E-13
ENSBTAT00000024514	ENSBTAG00000018423	DDX5	19	49330954	49337523	-	CODING	-1.47697	7.55E-13
ENSBTAT00000010279	ENSBTAG00000007816	-	6	87555288	87556157	+	CODING	-1.85713	8.06E-13
ENSBTAT00000036603	ENSBTAG00000012866	THBS4	10	10945425	10999244	+	CODING	-1.65677	8.15E-13
ENSBTAT00000059715	ENSBTAG00000042723	U11	2	1.25E+08	1.25E+08	-	CODING	-6.91425	8.60E-13
ENSBTAT00000040333	ENSBTAG00000027962	-	2	1.13E+08	1.13E+08	+	CODING	-0.23593	9.49E-13
ENSBTAT00000025454	ENSBTAG00000019124	EIF4EBP2	28	26764785	26786028	+	CODING	-0.09725	1.35E-12
ENSBTAT00000015829	ENSBTAG00000011931	CD63	5	57854278	57857485	+	CODING	-1.2231	1.63E-12
ENSBTAT00000017430	ENSBTAG00000013113	VDAC1	7	47247744	47273458	-	CODING	-0.37626	1.80E-12
ENSBTAT00000062708	ENSBTAG00000045275	SCARNA7	1	1.08E+08	1.08E+08	+	CODING	-2.86425	2.25E-12
ENSBTAT00000029668	ENSBTAG00000008061	RILPL1	17	54358764	54400473	+	CODING	-1.38367	2.25E-12
ENSBTAT00000031167	ENSBTAG00000002069	BOLA	23	28502524	28506312	-	CODING	-1.50238	2.30E-12
ENSBTAT00000014883	ENSBTAG00000011207	CNN1	7	17106394	17114222	+	CODING	-1.97365	2.30E-12
ENSBTAT00000059485	ENSBTAG00000042493	SNORA70	X	40366530	40366664	+	CODING	-6.85603	2.35E-12
ENSBTAT00000012351	ENSBTAG00000009387	MYOM2	27	312538	376193	+	CODING	-0.28567	2.42E-12
ENSBTAT00000048862	ENSBTAG00000034493	C6ORF106	23	8591963	8688924	-	CODING	-0.04654	2.46E-12
ENSBTAT00000031754	ENSBTAG00000023343	RPL28	18	62547220	62549950	-	CODING	-1.21951	2.51E-12
ENSBTAT00000017905	ENSBTAG00000013461	RPL24	1	46415223	46420721	-	CODING	-1.14326	2.56E-12
ENSBTAT00000002400	ENSBTAG00000001836	-	19	19937602	19947884	-	CODING	1.007637	2.62E-12
ENSBTAT00000040741	ENSBTAG00000028359	U3	19	9798918	9799131	-	CODING	-6.84634	2.77E-12
ENSBTAT00000024175	ENSBTAG00000018167	KLHL31	23	6771146	6785241	+	CODING	0.150908	3.08E-12
ENSBTAT00000022041	ENSBTAG00000016568	LUC7L	25	225533	257902	-	CODING	-1.94288	4.22E-12
ENSBTAT00000043182	ENSBTAG00000030575	BHLHE41	5	84233719	84236923	+	CODING	0.27703	4.43E-12
ENSBTAT00000022835	ENSBTAG00000017183	PDLIM3	27	14769571	14800205	-	CODING	-0.62123	5.06E-12
ENSBTAT00000002261	ENSBTAG00000001721	GYG1	1	1.2E+08	1.2E+08	-	CODING	-0.26673	5.39E-12
ENSBTAT00000004728	ENSBTAG00000024657	-	15	83248791	83286773	+	CODING	-0.0913	5.79E-12
ENSBTAT00000010176	ENSBTAG00000007737	UBA52	7	4535467	4537851	-	CODING	-1.0504	6.41E-12
ENSBTAT00000016993	ENSBTAG00000012788	COX6A1	17	64995248	64997121	+	CODING	-1.89205	7.00E-12
ENSBTAT00000029478	ENSBTAG00000009773	KREMEN1	17	70568207	70601539	+	CODING	0.554913	7.30E-12
ENSBTAT00000024128	ENSBTAG00000018127	PPM1A	10	72757582	72768778	+	CODING	-0.18811	7.35E-12
ENSBTAT00000009097	ENSBTAG00000006928	OAT	26	44378157	44397476	-	CODING	0.332633	7.46E-12
ENSBTAT00000066175	ENSBTAG00000048167	U4	17	64874908	64875048	-	CODING	-6.78515	7.64E-12
ENSBTAT00000006312	ENSBTAG00000004806	PHKB	18	15962196	16155801	+	CODING	-0.19474	7.73E-12
ENSBTAT00000045202	ENSBTAG00000031875	BANF1	29	44759486	44761429	+	CODING	-1.18612	7.86E-12
ENSBTAT00000002015	ENSBTAG00000001538	RPS16	3	1.18E+08	1.18E+08	+	CODING	-1.35115	9.88E-12
ENSBTAT00000033899	ENSBTAG00000002280	KIF5B	13	33619259	33665129	+	CODING	-0.06484	1.08E-11
ENSBTAT00000010126	ENSBTAG00000007700	PHYH	13	28254814	28275573	-	CODING	-0.46708	1.09E-11
ENSBTAT00000063623	ENSBTAG00000017509	MYPN	28	24797318	24833215	+	CODING	0.029694	1.13E-11
ENSBTAT00000025963	ENSBTAG00000019494	RPL10A	23	9391523	9394013	+	CODING	-1.34564	1.21E-11
ENSBTAT00000028880	ENSBTAG00000021672	RGS1	16	13314192	13318292	-	CODING	-2.89664	1.25E-11
ENSBTAT00000016514	ENSBTAG00000012447	PPP1CB	11	70961032	70997556	-	CODING	-0.37574	1.29E-11
ENSBTAT00000015358	ENSBTAG00000011559	RPL7A	11	1.04E+08	1.04E+08	+	CODING	-1.25323	1.30E-11
ENSBTAT00000031330	ENSBTAG00000008827	SPOCK2	28	28304694	28329730	-	CODING	-2.85947	1.34E-11
ENSBTAT00000029327	ENSBTAG00000021992	MURC	8	91916165	91928339	+	CODING	-0.14073	1.35E-11
ENSBTAT00000007115	ENSBTAG00000005408	CLK1	2	89920586	89927819	-	CODING	-1.71324	1.53E-11
ENSBTAT00000046364	ENSBTAG00000005314	MFN2	16	42561715	42581003	-	CODING	0.205808	1.55E-11
ENSBTAT00000027592	ENSBTAG00000046981	SVIL	13	34922199	34945103	-	CODING	-0.31565	1.59E-11
ENSBTAT00000046515	ENSBTAG00000003741	NEURL1	26	24330861	24403159	+	CODING	0.272987	2.35E-11
ENSBTAT00000036848	ENSBTAG00000007068	SH3BGR	1	1.41E+08	1.41E+08	+	CODING	-0.5047	2.54E-11
ENSBTAT00000030619	ENSBTAG00000015839	MAP4	22	52369974	52462511	+	CODING	0.098219	2.83E-11
ENSBTAT00000005982	ENSBTAG00000004553	TPM4	7	7923143	7948265	-	CODING	-1.68905	2.87E-11
ENSBTAT00000019803	ENSBTAG00000014872	CAPNS1	18	46987527	46994449	+	CODING	-1.24407	4.48E-11

ENSBTAT00000021199	ENSBTAG00000015942	DNAJA4	21	31177694	31192961	+	CODING	0.137002	4.84E-11
ENSBTAT00000046544	ENSBTAG00000032774	C28H10ORF1	28	41912322	41914565	+	CODING	-1.73118	5.49E-11
ENSBTAT00000020018	ENSBTAG00000015041	NDUFA2	7	53461635	53463955	-	CODING	-1.40195	5.92E-11
ENSBTAT00000001646	ENSBTAG00000001246	ATP1A1	3	27002873	27025641	-	CODING	-1.82526	6.62E-11
ENSBTAT00000056385	ENSBTAG00000016005	PPP3CA	6	24812682	25136247	+	CODING	0.069971	7.65E-11
ENSBTAT00000066053	ENSBTAG00000046786	UQCRFS1	18	1021248	1026029	-	CODING	-1.16603	8.77E-11
ENSBTAT00000047158	ENSBTAG00000033197	EPDR1	4	50028919	50064257	+	CODING	0.35149	8.94E-11
ENSBTAT00000004980	ENSBTAG00000003826	SCN1B	18	45960622	45970522	+	CODING	-0.22728	9.24E-11
ENSBTAT00000011257	ENSBTAG00000008541	MGST1	5	93926791	93950162	-	CODING	-2.15998	9.74E-11
ENSBTAT00000005592	ENSBTAG00000004269	SGK1	9	73305313	73310869	-	CODING	-2.26012	1.06E-10
ENSBTAT00000002707	ENSBTAG00000002098	CDC34	7	44789108	44794947	+	CODING	-0.45442	1.20E-10
ENSBTAT00000053250	ENSBTAG00000001274	PPM1L	1	1.07E+08	1.08E+08	-	CODING	-0.08525	1.29E-10
ENSBTAT00000019753	ENSBTAG00000014831	PPP1R3C	26	13270026	13274922	-	CODING	-0.03419	1.32E-10
ENSBTAT00000035955	ENSBTAG00000015131	SLC29A1	23	17845969	17856150	+	CODING	-1.39757	1.77E-10
ENSBTAT00000009440	ENSBTAG00000007172	GOT2	18	26533095	26556740	-	CODING	-0.49657	1.81E-10
ENSBTAT00000061385	ENSBTAG00000039682	MTUS1	27	18574170	18698056	+	CODING	-0.12445	1.92E-10
ENSBTAT00000021554	ENSBTAG00000016194	FBXO32	14	17919823	17951894	+	CODING	-0.39666	1.99E-10
ENSBTAT00000063331	ENSBTAG00000046054	RHOB	11	78464206	78466417	-	CODING	-1.54627	2.19E-10
ENSBTAT00000054745	ENSBTAG00000008997	ENG	11	98517162	98541363	-	CODING	-1.67027	2.20E-10
ENSBTAT00000028899	ENSBTAG00000021685	EEF1A2	13	54623917	54631595	+	CODING	-0.62127	2.48E-10
ENSBTAT00000042953	ENSBTAG00000004542	C9ORF59	11	1.01E+08	1.01E+08	-	CODING	0.398671	2.60E-10
ENSBTAT00000035177	ENSBTAG00000025136	MYOZ3	7	64031684	64048678	+	CODING	0.005524	2.63E-10
ENSBTAT00000065769	ENSBTAG00000047330	FABP5	14	46644609	46649827	+	CODING	-1.48498	2.69E-10
ENSBTAT00000014304	ENSBTAG00000010799	MYL6	5	57486017	57489133	-	CODING	-1.29262	2.71E-10
ENSBTAT00000009395	ENSBTAG00000007139	WSB2	17	59422963	59437371	+	CODING	0.013883	2.71E-10
ENSBTAT00000003925	ENSBTAG00000003015	SESN1	9	41666967	41690061	+	CODING	-2.99523	2.98E-10
ENSBTAT00000034186	ENSBTAG00000024561	H3F3A	16	29832591	29840787	+	CODING	-1.34928	3.13E-10
ENSBTAT00000008502	ENSBTAG00000006487	RPS9	18	63381416	63388728	-	CODING	-1.22857	4.00E-10
ENSBTAT00000038612	ENSBTAG00000026972	MYF5	5	10339425	10342660	+	CODING	-2.84271	4.10E-10
ENSBTAT00000038531	ENSBTAG00000038025	H2B	3	20771300	20773622	+	CODING	-0.46056	4.23E-10
ENSBTAT00000031908	ENSBTAG00000023416	PPP2R3A	1	1.34E+08	1.34E+08	-	CODING	0.145128	4.51E-10
ENSBTAT00000015385	ENSBTAG00000011580	DAG1	22	51187154	51200326	-	CODING	-0.09468	5.03E-10
ENSBTAT00000032663	ENSBTAG00000020791	RAPGEF1	11	1.02E+08	1.02E+08	-	CODING	0.007621	5.79E-10
ENSBTAT00000001758	ENSBTAG00000001335	GHR	20	31890736	32199996	-	CODING	-0.05728	6.02E-10
ENSBTAT00000030320	ENSBTAG00000011869	CSRP3	29	25994182	26014859	+	CODING	-0.65679	6.63E-10
ENSBTAT00000020561	ENSBTAG00000015470	SYPL2	3	34082543	34095369	-	CODING	-0.30923	6.95E-10
ENSBTAT00000019972	ENSBTAG00000014991	PARD3	13	18982015	19337608	+	CODING	0.404354	7.66E-10
ENSBTAT00000060896	ENSBTAG00000043904	SNORA57	4	43820203	43820347	+	CODING	-6.46949	8.32E-10
ENSBTAT00000021641	ENSBTAG00000016269	ME2	24	50870262	50928290	+	CODING	0.269439	9.47E-10
ENSBTAT00000017372	ENSBTAG00000013066	IGF2	29	50046626	50065230	+	CODING	-1.53554	1.11E-09
ENSBTAT00000002055	ENSBTAG00000001575	-	11	29424996	29432052	-	CODING	-1.18512	1.13E-09
ENSBTAT00000064387	ENSBTAG00000047502	FKBP5	23	9521254	9637802	-	CODING	-1.2967	1.21E-09
ENSBTAT00000053822	ENSBTAG00000015786	LRRC39	3	43251251	43272503	+	CODING	-0.36692	1.21E-09
ENSBTAT00000011066	ENSBTAG00000008409	MYC	14	13769244	13774438	-	CODING	-2.41965	1.22E-09
ENSBTAT00000018808	ENSBTAG00000014151	RCSD1	3	1058468	1130225	-	CODING	0.051231	1.26E-09
ENSBTAT00000012057	ENSBTAG00000009151	PYGO1	10	54865902	54887753	+	CODING	0.176431	1.29E-09
ENSBTAT00000023751	ENSBTAG00000017869	CAV1	4	52173110	52208687	-	CODING	-1.24138	1.38E-09
ENSBTAT00000029455	ENSBTAG00000006084	PINK1	2	1.33E+08	1.33E+08	-	CODING	-0.36255	1.54E-09
ENSBTAT00000011643	ENSBTAG00000008842	JPH1	14	39633189	39725607	-	CODING	-0.3559	1.70E-09
ENSBTAT00000000790	ENSBTAG00000000598	CST3	13	42562167	42566091	-	CODING	-0.49519	1.84E-09
ENSBTAT00000007241	ENSBTAG00000034506	SNRPG	19	12231223	12231584	+	CODING	-1.55408	1.90E-09
ENSBTAT00000010231	ENSBTAG00000007782	MYOT	7	50941047	50958425	+	CODING	-0.68219	1.93E-09
ENSBTAT00000035014	ENSBTAG00000020701	MEF2C	7	90616543	90784162	-	CODING	0.004538	1.93E-09
ENSBTAT00000003460	ENSBTAG00000002670	C28H10ORF1	28	44952355	44953778	+	CODING	-2.73767	2.03E-09
ENSBTAT00000005403	ENSBTAG00000004126	MLF1	1	1.1E+08	1.1E+08	-	CODING	-0.39752	2.09E-09
ENSBTAT00000045265	ENSBTAG00000010422	MDM2	5	45178135	45203567	-	CODING	0.206053	2.16E-09
ENSBTAT00000020651	ENSBTAG00000015543	ARHGAP35	18	54425390	54497692	+	CODING	0.442158	2.45E-09
ENSBTAT00000011740	ENSBTAG00000008920	ATP1B4	X	4919686	4940505	-	CODING	-0.00199	2.50E-09
ENSBTAT00000010698	ENSBTAG00000008137	-	5	75519790	75520044	+	CODING	-1.21002	2.78E-09
ENSBTAT00000011931	ENSBTAG00000009055	RNF144B	23	38952210	39043167	-	CODING	-0.0614	2.91E-09
ENSBTAT00000061564	ENSBTAG00000039374	RANBP6	12	79000723	79039990	+	CODING	-0.20982	2.91E-09

ENSBTAT00000016981	ENSBTAG00000012777	SRF	23	16747958	16754754	+	CODING	-0.06681	2.92E-09
ENSBTAT00000025740	ENSBTAG00000019327	NRAP	26	34339430	34414796	-	CODING	-0.49635	2.97E-09
ENSBTAT00000008072	ENSBTAG00000006135	MAP1LC3A	13	64497254	64498905	+	CODING	-1.57026	3.02E-09
ENSBTAT00000026715	ENSBTAG00000020050	MLEC	17	65184868	65194879	+	CODING	0.591851	3.31E-09
ENSBTAT00000028141	ENSBTAG00000021120	SMYD1	11	47799217	47848713	-	CODING	-0.08767	3.33E-09
ENSBTAT00000007527	ENSBTAG00000005726	HNRNPA2B1	4	70198053	70207085	+	CODING	-1.14201	3.59E-09
ENSBTAT00000008517	ENSBTAG00000006499	PIP4K2B	19	39995184	40017502	-	CODING	-0.00666	3.60E-09
ENSBTAT00000013799	ENSBTAG00000010452	PODXL	4	96032591	96039050	-	CODING	-1.7127	4.09E-09
ENSBTAT00000005143	ENSBTAG00000003937	-	13	22813811	22814335	+	CODING	-1.11701	4.10E-09
ENSBTAT00000029468	ENSBTAG00000022032	CHCHD10	17	73206771	73208629	-	CODING	-1.14808	4.14E-09
ENSBTAT00000010193	ENSBTAG00000007754	NDUFA3	18	63462789	63466137	-	CODING	-1.27732	4.30E-09
ENSBTAT00000021614	ENSBTAG00000016240	RNF157	19	56112050	56167541	+	CODING	0.282746	4.32E-09
ENSBTAT00000019129	ENSBTAG00000014387	PRKAB2	3	22635094	22646401	+	CODING	0.301313	4.63E-09
ENSBTAT00000000612	ENSBTAG00000000484	HYAL2	22	50592324	50597462	+	CODING	-3.62181	4.65E-09
ENSBTAT00000001128	ENSBTAG00000006724	PPP1R2	1	72708757	72726620	+	CODING	-0.31228	5.49E-09
ENSBTAT00000004984	ENSBTAG00000003830	PSMB3	19	39980435	39989908	+	CODING	-1.38359	5.54E-09
ENSBTAT00000015811	ENSBTAG00000011917	GPAM	26	32963414	33003349	-	CODING	-1.98421	5.64E-09
ENSBTAT00000055832	ENSBTAG00000006563	KBTBD5	22	15444466	15451791	+	CODING	-1.40408	5.89E-09
ENSBTAT00000000730	ENSBTAG00000000560	-	19	13525432	13526742	+	CODING	-1.27707	6.28E-09
ENSBTAT00000002674	ENSBTAG00000002068	TAGLN2	3	9878839	9886647	+	CODING	-1.58197	6.34E-09
ENSBTAT00000060985	ENSBTAG00000044017	MSRB3	5	48563806	48743354	-	CODING	-0.31452	6.43E-09
ENSBTAT00000004188	ENSBTAG00000003228	RPL3	5	1.11E+08	1.11E+08	-	CODING	-1.46967	6.93E-09
ENSBTAT00000028602	ENSBTAG00000021455	CFL1	29	44638896	44642280	-	CODING	-1.45142	7.22E-09
ENSBTAT00000055747	ENSBTAG00000038079	C16H1ORF21	16	66960790	67142414	+	CODING	-0.24466	7.34E-09
ENSBTAT00000018428	ENSBTAG00000013881	GJA4	3	1.11E+08	1.11E+08	-	CODING	-1.95391	7.94E-09
ENSBTAT00000037254	ENSBTAG00000026266	MYL12B	24	37834593	37910130	+	CODING	-1.95689	8.37E-09
ENSBTAT00000024391	ENSBTAG00000018331	CLEC3B	22	54791195	54797301	-	CODING	-1.67731	1.00E-08
ENSBTAT00000024376	ENSBTAG00000018320	RPLP1	10	16324341	16326492	+	CODING	-0.92833	1.02E-08
ENSBTAT00000022033	ENSBTAG00000016563	GOLGA4	22	10813998	10923960	+	CODING	-0.2303	1.05E-08
ENSBTAT00000002970	ENSBTAG00000002299	SEL1L3	6	46791165	46881531	-	CODING	0.129767	1.19E-08
ENSBTAT00000020125	ENSBTAG00000015127	SDC4	13	74393120	74412880	-	CODING	-1.77158	1.46E-08
ENSBTAT00000023192	ENSBTAG00000017450	KLHL24	1	84062791	84095207	-	CODING	0.025849	1.52E-08
ENSBTAT00000017131	ENSBTAG00000012890	SLC25A3	5	63086353	63092547	+	CODING	-0.47648	1.62E-08
ENSBTAT00000018414	ENSBTAG00000013866	RPS27	3	16505732	16507247	-	CODING	-1.07896	1.69E-08
ENSBTAT00000015408	ENSBTAG00000011596	SFRS5	10	81860044	81865204	+	CODING	-1.74629	1.71E-08
ENSBTAT00000012599	ENSBTAG00000009580	SH3BGRL3	2	1.27E+08	1.27E+08	-	CODING	-1.48643	1.84E-08
ENSBTAT00000007861	ENSBTAG00000005990	S1PR1	3	42184097	42188752	-	CODING	-1.65739	1.99E-08
ENSBTAT00000065572	ENSBTAG00000045568	-	24	13077339	13078261	+	CODING	-1.14919	2.15E-08
ENSBTAT00000015694	ENSBTAG00000011824	OGN	8	85453132	85468721	+	CODING	-1.78832	2.18E-08
ENSBTAT00000057596	ENSBTAG00000040028	MGC166429	7	2600732	2618482	-	CODING	-0.32419	2.21E-08
ENSBTAT00000005786	ENSBTAG00000004413	RHOBTB3	7	97350408	97406000	+	CODING	0.209403	2.39E-08
ENSBTAT00000056227	ENSBTAG00000002964	TXLNB	9	77937305	77996890	-	CODING	-0.16437	2.53E-08
ENSBTAT00000013663	ENSBTAG00000010347	EZR	9	96598249	96643275	-	CODING	-1.89982	2.59E-08
ENSBTAT00000015828	ENSBTAG00000011922	PLEC	14	2054917	2088261	+	CODING	-0.06855	2.74E-08
ENSBTAT00000009155	ENSBTAG00000025385	RPL12	10	83480588	83481217	-	CODING	-1.10151	2.83E-08
ENSBTAT00000005380	ENSBTAG00000004118	ALAS1	22	49242706	49256766	-	CODING	0.024664	2.88E-08
ENSBTAT00000012394	ENSBTAG00000009417	ZFAND5	8	48892499	48902995	-	CODING	-1.09683	3.07E-08
ENSBTAT00000066051	ENSBTAG00000046176	SPEG	2	1.08E+08	1.08E+08	+	CODING	0.112821	3.19E-08
ENSBTAT00000027662	ENSBTAG00000020757	PCBP2	5	26702879	26723947	-	CODING	-0.37614	3.38E-08
ENSBTAT00000007504	ENSBTAG00000005714	ACTC1	10	30361781	30367052	-	CODING	0.010375	3.57E-08
ENSBTAT00000063958	ENSBTAG00000045729	SMIM20	16	68078643	68078846	+	CODING	0.419482	3.59E-08
ENSBTAT00000015701	ENSBTAG00000011831	SPPL2A	10	59779604	59813156	+	CODING	-0.07426	3.79E-08
ENSBTAT00000016279	ENSBTAG00000012276	RPL5	13	38980088	38981117	-	CODING	-1.18226	4.06E-08
ENSBTAT00000026762	ENSBTAG00000020087	CAMK2A	7	63576976	63641028	-	CODING	0.256452	4.08E-08
ENSBTAT00000012670	ENSBTAG00000009621	-	16	41507401	41508463	+	CODING	-0.19508	4.25E-08
ENSBTAT00000023994	ENSBTAG00000018024	NR1D2	27	41866520	41883521	-	CODING	0.357164	4.34E-08
ENSBTAT00000028174	ENSBTAG00000021140	RMND5A	11	48010205	48066194	-	CODING	-0.17014	4.51E-08
ENSBTAT00000000932	ENSBTAG00000000698	MYO18B	17	67768324	68000455	+	CODING	0.214687	4.63E-08
ENSBTAT00000012786	ENSBTAG00000009696	ACTN2	28	9403202	9450916	+	CODING	-0.61502	4.64E-08
ENSBTAT00000015985	ENSBTAG00000012048	CARM1	7	16571430	16587354	+	CODING	-0.13592	4.80E-08
ENSBTAT00000028239	ENSBTAG00000021191	EHD2	18	55071102	55087454	+	CODING	-1.40714	4.90E-08

ENSBTAT00000062174	ENSBTAG00000044741	SNORD97	15	42441794	42441945	+	CODING	-2.36215	5.00E-08
ENSBTAT00000001303	ENSBTAG00000000985	RAB10	11	73335436	73400455	-	CODING	-0.33953	5.14E-08
ENSBTAT00000005025	ENSBTAG00000003851	CCNL1	1	1.11E+08	1.11E+08	+	CODING	-1.95237	5.18E-08
ENSBTAT00000010166	ENSBTAG00000027610	RPL36A	15	54468007	54468423	+	CODING	-1.09368	5.21E-08
ENSBTAT00000015810	ENSBTAG00000011918	BLOC1S1	5	57863517	57866922	-	CODING	-1.40447	5.49E-08
ENSBTAT00000005283	ENSBTAG00000004041	ANKRD10	12	89304124	89331787	-	CODING	-1.63715	5.50E-08
ENSBTAT00000045880	ENSBTAG00000009570	C19orf80	7	16878927	16880796	+	CODING	-4.60579	5.67E-08
ENSBTAT00000003236	ENSBTAG00000002490	CHPT1	5	65852754	65882216	+	CODING	-0.09509	5.89E-08
ENSBTAT00000029560	ENSBTAG00000022058	ACACB	17	66102654	66198865	-	CODING	-1.66665	6.01E-08
ENSBTAT00000015530	ENSBTAG00000038107	MAPKAPK2	16	4319154	4365926	+	CODING	-0.28541	6.02E-08
ENSBTAT00000032142	ENSBTAG00000038131	ABCC5	1	83736248	83764147	+	CODING	-2.04602	6.15E-08
ENSBTAT00000017277	ENSBTAG00000012996	RNF11	3	95599737	95601983	-	CODING	-0.3752	6.49E-08
ENSBTAT00000007124	ENSBTAG00000005414	-	1	51022816	51023423	+	CODING	-1.82356	6.50E-08
ENSBTAT00000025843	ENSBTAG00000019394	ANO5	29	22783297	22859063	-	CODING	0.295666	6.61E-08
ENSBTAT00000025582	ENSBTAG00000019210	ADCY2	20	65505171	65653865	-	CODING	-0.20098	7.46E-08
ENSBTAT00000012541	ENSBTAG00000009533	RPL3L	25	1508568	1515379	-	CODING	-1.337	7.47E-08
ENSBTAT00000020468	ENSBTAG00000015402	GREB1	11	86199420	86268193	-	CODING	2.16284	8.49E-08
ENSBTAT00000014853	ENSBTAG00000011184	FTH1	29	41172509	41175112	-	CODING	-0.57964	8.88E-08
ENSBTAT00000026323	ENSBTAG00000019754	PRKCDBP	15	47329716	47331334	+	CODING	-1.87579	9.19E-08
ENSBTAT00000029256	ENSBTAG00000021944	C10H14ORF1	10	44879976	44894546	+	CODING	-1.16913	1.01E-07
ENSBTAT00000022876	ENSBTAG00000017212	C21H15orf63	21	55974568	55975823	+	CODING	-1.28651	1.19E-07
ENSBTAT00000018239	ENSBTAG00000013724	ATG4A	X	60973684	61051097	+	CODING	0.1468	1.23E-07
ENSBTAT00000002319	ENSBTAG00000001771	DYRK1A	1	1.51E+08	1.51E+08	+	CODING	0.012757	1.24E-07
ENSBTAT00000052506	ENSBTAG00000037516	-	13	16262484	16263567	-	CODING	-0.38818	1.24E-07
ENSBTAT00000016904	ENSBTAG00000012718	XK	X	1.11E+08	1.11E+08	-	CODING	0.368257	1.25E-07
ENSBTAT00000015769	ENSBTAG00000011885	NNT	20	31171388	31258005	-	CODING	-0.28434	1.57E-07
ENSBTAT00000038527	ENSBTAG00000001083	-	2	23443013	23608298	-	CODING	0.291012	1.65E-07
ENSBTAT00000005727	ENSBTAG00000004368	NFATC3	18	35640709	35732176	+	CODING	0.061372	1.69E-07
ENSBTAT00000018411	ENSBTAG00000013863	DUSP1	20	4449109	4452189	-	CODING	-1.13052	1.73E-07
ENSBTAT00000044159	ENSBTAG00000031184	CDKN1C	29	49368787	49370785	+	CODING	-2.33858	1.76E-07
ENSBTAT00000018339	ENSBTAG00000013799	-	2	1.08E+08	1.08E+08	-	CODING	-0.42908	1.93E-07
ENSBTAT00000022579	ENSBTAG00000016977	FUNDC2	X	38815313	38838616	-	CODING	-0.41079	1.93E-07
ENSBTAT00000020105	ENSBTAG00000015109	TOB2	5	1.13E+08	1.13E+08	-	CODING	-0.22108	2.10E-07
ENSBTAT00000011734	ENSBTAG00000008915	SF3B1	2	86349373	86387060	-	CODING	-1.42678	2.31E-07
ENSBTAT00000006465	ENSBTAG00000004915	PFN1	19	27081319	27084643	+	CODING	-1.27346	2.33E-07
ENSBTAT00000019555	ENSBTAG00000014693	TMEM88	19	28156792	28157769	+	CODING	-1.87103	2.37E-07
ENSBTAT00000001447	ENSBTAG00000001093	KLHL23	2	26619585	26636478	-	CODING	0.335978	2.53E-07
ENSBTAT00000017897	ENSBTAG00000013454	TAB2	9	87759388	87798966	+	CODING	-0.09919	2.54E-07
ENSBTAT00000034976	ENSBTAG00000025046	ALKBH5	19	35025249	35041293	-	CODING	-0.15234	2.61E-07
ENSBTAT00000006383	ENSBTAG00000004855	PRDX6	16	56389804	56399714	+	CODING	-0.39584	2.66E-07
ENSBTAT00000006658	ENSBTAG00000005048	DHRS7C	19	29581010	29594334	-	CODING	-0.39704	2.92E-07
ENSBTAT00000064829	ENSBTAG00000046333	-	6	26345281	26350653	+	CODING	-0.09016	2.98E-07
ENSBTAT00000027409	ENSBTAG00000020569	CACNA2D1	4	38712468	38856748	+	CODING	0.007173	2.99E-07
ENSBTAT00000046355	ENSBTAG00000010611	OCIAD1	6	69144752	69167276	+	CODING	-1.22728	3.00E-07
ENSBTAT00000053463	ENSBTAG00000040055	CAB39	2	1.19E+08	1.19E+08	+	CODING	-0.22031	3.03E-07
ENSBTAT00000024965	ENSBTAG00000018747	PRKAA2	3	90055299	90127142	-	CODING	0.333682	3.13E-07
ENSBTAT00000020937	ENSBTAG00000015767	RNF10	17	65067805	65101419	+	CODING	-0.35691	3.20E-07
ENSBTAT00000006196	ENSBTAG00000004723	TEX2	19	49006459	49068544	-	CODING	-0.04526	3.28E-07
ENSBTAT00000018998	ENSBTAG00000014299	RHOC	3	30769966	30776469	+	CODING	-1.74034	3.44E-07
ENSBTAT00000064544	ENSBTAG00000046531	-	X	95330253	95330600	-	CODING	-1.48292	3.48E-07
ENSBTAT00000022832	ENSBTAG00000017181	MACROD1	29	42890800	43092842	-	CODING	-1.02268	3.48E-07
ENSBTAT00000024312	ENSBTAG00000018267	TRIM54	11	72327351	72348528	-	CODING	-1.10669	3.59E-07
ENSBTAT00000011972	ENSBTAG00000009080	ITGB6	2	36256325	36348759	+	CODING	0.917121	3.72E-07
ENSBTAT00000002933	ENSBTAG00000002275	PTP4A1	9	512139	516069	+	CODING	-0.22178	3.80E-07
ENSBTAT00000019817	ENSBTAG00000014885	MYOM3	2	1.29E+08	1.29E+08	+	CODING	-0.09028	3.90E-07
ENSBTAT00000006899	ENSBTAG00000005244	RASL11A	12	32842604	32844825	-	CODING	-2.62316	3.95E-07
ENSBTAT00000004418	ENSBTAG00000003407	SF3B14	11	75039681	75047800	+	CODING	-1.55494	4.00E-07
ENSBTAT00000024644	ENSBTAG00000018513	FHL1	X	19777799	19820952	+	CODING	-1.98771	4.14E-07
ENSBTAT00000049004	ENSBTAG00000017753	APP	1	9607382	9921004	+	CODING	-1.37792	4.19E-07
ENSBTAT00000064987	ENSBTAG00000046587	-	29	44770865	44771529	-	CODING	-2.15182	4.72E-07
ENSBTAT00000005324	ENSBTAG00000004072	CAPZA2	4	51781849	51836823	-	CODING	-0.36419	4.91E-07

ENSBTAT00000017995	ENSBTAG00000013533	CLIC1	23	27393342	27398934	+	CODING	-1.94968	4.98E-07
ENSBTAT00000035874	ENSBTAG00000022590	BOLA	23	27863067	27867532	+	CODING	-1.85022	5.14E-07
ENSBTAT00000047737	ENSBTAG00000033603	UQCC2	23	7796308	7820842	-	CODING	-1.58311	5.28E-07
ENSBTAT00000019939	ENSBTAG00000037605	BOLA-DQA1	23	25426330	25430097	-	CODING	0.520074	5.30E-07
ENSBTAT00000022366	ENSBTAG00000037558	GRO1	6	90822748	90824841	+	CODING	-3.65934	5.45E-07
ENSBTAT00000022534	ENSBTAG00000016943	NEK7	16	79057373	79136394	+	CODING	0.449907	5.46E-07
ENSBTAT00000016607	ENSBTAG00000012509	DYRK1B	18	49650522	49658124	-	CODING	0.138445	5.72E-07
ENSBTAT00000025142	ENSBTAG00000018887	IPO13	3	1.03E+08	1.03E+08	-	CODING	-0.24647	5.79E-07
ENSBTAT00000020850	ENSBTAG00000015704	TMCO3	12	90698790	90720746	+	CODING	-0.07239	5.80E-07
ENSBTAT00000036654	ENSBTAG00000003536	42800	20	62940001	62990698	-	CODING	-0.02225	6.47E-07
ENSBTAT00000021033	ENSBTAG00000015831	RPL18A	7	5206112	5209504	-	CODING	-1.04552	6.54E-07
ENSBTAT00000026560	ENSBTAG00000019938	N4BP1	18	17033279	17085940	-	CODING	0.043902	6.58E-07
ENSBTAT00000054925	ENSBTAG00000040018	-	28	18349473	18349742	-	CODING	-1.41017	6.72E-07
ENSBTAT00000019699	ENSBTAG00000014804	HNRPDL	6	98983515	98990087	-	CODING	-1.25928	6.93E-07
ENSBTAT00000000742	ENSBTAG00000000569	HES1	1	73974252	73976720	-	CODING	-0.10816	7.17E-07
ENSBTAT00000025863	ENSBTAG00000019414	CLK4	7	41100796	41122562	-	CODING	-1.566	7.31E-07
ENSBTAT00000038885	ENSBTAG00000032657	TEAD1	15	40303805	40482346	-	CODING	0.093505	7.78E-07
ENSBTAT00000044743	ENSBTAG00000031558	-	21	38276192	38276344	-	CODING	-1.93286	7.92E-07
ENSBTAT00000028257	ENSBTAG00000021205	PPAPDC3	11	1.02E+08	1.02E+08	+	CODING	-0.2254	8.05E-07
ENSBTAT00000020520	ENSBTAG00000015441	ACTB	11	10717554	10732398	-	CODING	-2.19316	8.06E-07
ENSBTAT00000063756	ENSBTAG00000047637	-	11	46404611	46404925	-	CODING	-1.20711	8.33E-07
ENSBTAT00000039206	ENSBTAG00000000894	PGK1	X	79282708	79305316	-	CODING	-8.28802	8.50E-07
ENSBTAT00000028364	ENSBTAG00000021288	PSMB4	3	19412241	19414600	-	CODING	-1.19958	8.66E-07
ENSBTAT00000014215	ENSBTAG00000010738	CCL14	19	14775262	14780081	+	CODING	-1.3888	8.68E-07
ENSBTAT00000017647	ENSBTAG00000025853	HOMER1	10	10350692	10484916	-	CODING	-0.06815	8.77E-07
ENSBTAT00000002787	ENSBTAG00000002151	ASB15	4	88697392	88729365	+	CODING	0.338573	8.86E-07
ENSBTAT00000027837	ENSBTAG00000020894	LAPTM4A	11	78862495	78880461	+	CODING	-1.1634	9.83E-07
ENSBTAT00000063321	ENSBTAG00000046121	-	14	79340383	79345452	+	CODING	-2.26798	9.93E-07
ENSBTAT00000059908	ENSBTAG00000042916	SNORD15	15	55374442	55374585	+	CODING	-5.83862	1.01E-06
ENSBTAT00000064895	ENSBTAG00000022808	CACUL1	26	39292197	39309978	-	CODING	0.694387	1.02E-06
ENSBTAT00000016504	ENSBTAG00000012432	FDFT1	8	7427461	7453906	-	CODING	-0.23174	1.03E-06
ENSBTAT00000023622	ENSBTAG00000031788	GSTM1	3	33782528	33800900	-	CODING	-0.30862	1.04E-06
ENSBTAT00000023487	ENSBTAG00000017662	EEF2K	25	20115008	20165902	+	CODING	-0.12767	1.04E-06
ENSBTAT00000029269	ENSBTAG00000021953	LARGE	5	72157229	72769395	-	CODING	0.13575	1.08E-06
ENSBTAT00000020150	ENSBTAG00000015147	S100A10	3	18799612	18810545	+	CODING	-1.28547	1.08E-06
ENSBTAT00000019194	ENSBTAG00000014433	UBE2G1	19	25409138	25496726	-	CODING	-0.5179	1.10E-06
ENSBTAT00000016013	ENSBTAG00000012072	NDUFS8	29	46205045	46208795	+	CODING	-1.39386	1.10E-06
ENSBTAT00000015200	ENSBTAG00000011437	-	1	1.46E+08	1.46E+08	-	CODING	-2.0223	1.11E-06
ENSBTAT00000017826	ENSBTAG00000039340	SCN4B	15	29257448	29277309	-	CODING	-0.4178	1.12E-06
ENSBTAT00000044139	ENSBTAG00000013066	IGF2	29	50058063	50062631	+	CODING	-1.98325	1.14E-06
ENSBTAT00000021068	ENSBTAG00000015848	PHKA1	X	83004459	83217747	+	CODING	-0.41711	1.17E-06
ENSBTAT00000065745	ENSBTAG00000047342	-	25	31086700	31086912	-	CODING	-2.93413	1.17E-06
ENSBTAT00000020168	ENSBTAG00000015163	TM4SF1	1	1.2E+08	1.2E+08	+	CODING	-1.58279	1.27E-06
ENSBTAT00000024555	ENSBTAG00000018451	PTMS	5	1.04E+08	1.04E+08	-	CODING	-1.28448	1.28E-06
ENSBTAT00000007616	ENSBTAG00000005793	PEA15	3	9578375	9588605	-	CODING	-1.40311	1.30E-06
ENSBTAT00000025506	ENSBTAG00000019164	RHOBTB1	28	16729036	16802909	-	CODING	0.14628	1.32E-06
ENSBTAT00000017251	ENSBTAG00000012975	VDAC2	28	31154552	31168822	+	CODING	-0.19989	1.36E-06
ENSBTAT00000034135	ENSBTAG00000024542	PIGC	16	40785953	40788409	-	CODING	-0.40607	1.37E-06
ENSBTAT00000008146	ENSBTAG00000006202	ST13	5	1.13E+08	1.13E+08	-	CODING	-0.37511	1.44E-06
ENSBTAT00000015695	ENSBTAG00000011825	C7H19orf43	7	13896310	13899366	+	CODING	-1.5288	1.45E-06
ENSBTAT00000018910	ENSBTAG00000014226	RPL34	6	17828130	17832711	-	CODING	-1.07542	1.45E-06
ENSBTAT00000021398	ENSBTAG00000016076	TRAK1	22	15084213	15160669	+	CODING	-0.11195	1.55E-06
ENSBTAT00000040033	ENSBTAG00000027766	C1QTNF5	15	30448399	30450337	-	CODING	-2.87943	1.57E-06
ENSBTAT00000020207	ENSBTAG00000015188	KLF6	13	44945068	44952151	+	CODING	-1.65645	1.59E-06
ENSBTAT00000003055	ENSBTAG00000002362	APOLD1	5	97520910	97524789	-	CODING	-1.75108	1.63E-06
ENSBTAT00000019585	ENSBTAG00000014719	TMOD1	8	63180883	63269863	+	CODING	-0.42919	1.66E-06
ENSBTAT00000005060	ENSBTAG00000003877	ZCCHC24	28	35189622	35251005	-	CODING	-0.02358	1.68E-06
ENSBTAT00000047899	ENSBTAG00000033727	RBPMS	27	25637686	25816012	+	CODING	-2.21549	1.71E-06
ENSBTAT00000010022	ENSBTAG00000007622	CATD	29	50352064	50361497	+	CODING	-1.1974	1.76E-06
ENSBTAT00000060562	ENSBTAG00000043546	MT-ND6	MT	13913	14440	-	CODING	-0.85545	1.84E-06
ENSBTAT00000027504	ENSBTAG00000020638	TIMP3	5	71751415	71809052	+	CODING	-1.22273	1.86E-06

ENSBTAT00000049486	ENSBTAG00000014766	SRSF1	19	9038200	9041927	-	CODING	-1.354	1.92E-06
ENSBTAT00000055320	ENSBTAG00000020308	EIF4G2	15	42435880	42446235	+	CODING	-0.57933	1.95E-06
ENSBTAT00000066170	ENSBTAG00000045877	TSC22D3	X	59707292	59772737	+	CODING	-0.16374	1.96E-06
ENSBTAT00000011952	ENSBTAG00000009067	DDX6	15	29887449	29918570	-	CODING	-0.11306	1.99E-06
ENSBTAT00000013507	ENSBTAG00000010232	NDUFS5	3	1.08E+08	1.08E+08	-	CODING	-1.12752	2.01E-06
ENSBTAT00000007423	ENSBTAG00000039958	GYS1	18	55996079	56012727	-	CODING	-0.45031	2.02E-06
ENSBTAT00000016717	ENSBTAG00000012594	MRPS6	1	669920	733729	-	CODING	-1.59489	2.05E-06
ENSBTAT00000014734	ENSBTAG00000025666	RPS29	10	26814899	26815069	-	CODING	4.92024	2.07E-06
ENSBTAT00000055345	ENSBTAG00000006907	NEB	2	44546002	44693638	+	CODING	-2.57792	2.09E-06
ENSBTAT00000001925	ENSBTAG00000001468	SAMD4A	10	67430991	67516426	+	CODING	0.373844	2.12E-06
ENSBTAT00000001034	ENSBTAG00000000778	-	23	17867543	17873174	+	CODING	-1.12487	2.16E-06
ENSBTAT00000020159	ENSBTAG00000015154	MCL1	3	20172325	20176960	+	CODING	-1.26324	2.18E-06
ENSBTAT00000064534	ENSBTAG00000046733	-	9	1.02E+08	1.02E+08	-	CODING	-2.7295	2.23E-06
ENSBTAT00000015260	ENSBTAG00000011482	SLC43A2	19	23261580	23301008	-	CODING	-1.54215	2.23E-06
ENSBTAT00000020564	ENSBTAG00000015474	SFRS11	3	75243969	75293883	-	CODING	-1.811	2.29E-06
ENSBTAT00000002096	ENSBTAG00000001603	YIPF7	6	64882684	64916426	-	CODING	-0.05448	2.37E-06
ENSBTAT00000001731	ENSBTAG00000001311	MORF4L2	X	57744030	57756166	-	CODING	-1.17145	2.49E-06
ENSBTAT00000000925	ENSBTAG00000000693	UBE2R2	8	76567996	76663653	+	CODING	-0.27238	2.62E-06
ENSBTAT00000019649	ENSBTAG00000014771	RBMX2	X	14270247	14282970	+	CODING	-2.43052	2.75E-06
ENSBTAT00000032665	ENSBTAG00000023806	COBL	4	4494964	4728977	+	CODING	-0.18195	2.76E-06
ENSBTAT00000014176	ENSBTAG00000010709	-	15	22665193	22697952	+	CODING	-0.10641	2.76E-06
ENSBTAT00000055368	ENSBTAG00000020796	UBE2D1	26	663449	699996	-	CODING	-0.20398	2.93E-06
ENSBTAT00000044787	ENSBTAG00000031598	GBAS	25	27919682	27946564	+	CODING	-1.05399	2.94E-06
ENSBTAT00000009492	ENSBTAG00000007215	RNPEPL1	3	1.2E+08	1.2E+08	+	CODING	-0.16633	3.05E-06
ENSBTAT00000010815	ENSBTAG00000008224	PAIP2	7	52252774	52270426	+	CODING	-1.13444	3.07E-06
ENSBTAT00000063686	ENSBTAG00000046017	POPDC3	9	45452891	45455659	+	CODING	-0.02292	3.12E-06
ENSBTAT00000028016	ENSBTAG00000021035	CTSK	3	19994998	20007861	+	CODING	-1.77217	3.13E-06
ENSBTAT00000002513	ENSBTAG00000001932	MGC142781	11	99576794	99589668	-	CODING	-0.42895	3.18E-06
ENSBTAT00000025696	ENSBTAG00000019298	STRADB	2	90432216	90456178	+	CODING	-0.27662	3.23E-06
ENSBTAT00000024225	ENSBTAG00000002960	WDR82P1	22	49218179	49227366	+	CODING	-1.30352	3.29E-06
ENSBTAT00000063319	ENSBTAG00000010204	PCMT1	9	88068718	88101696	+	CODING	-0.20496	3.39E-06
ENSBTAT00000023612	ENSBTAG00000017755	EWSR1	17	70689624	70715166	+	CODING	-1.57714	3.40E-06
ENSBTAT00000048484	ENSBTAG00000034206	EMCN	6	25578634	25700215	+	CODING	-1.64518	3.77E-06
ENSBTAT00000008727	ENSBTAG00000006642	PRKACA	7	12693768	12711139	+	CODING	-0.10861	3.78E-06
ENSBTAT00000016093	ENSBTAG00000012125	GDI1	X	40411179	40416941	+	CODING	-1.6802	3.82E-06
ENSBTAT00000018681	ENSBTAG00000014060	LSM6	17	12322226	12340189	-	CODING	-1.34801	3.89E-06
ENSBTAT00000024583	ENSBTAG00000018471	SSR3	1	1.12E+08	1.12E+08	+	CODING	-0.34346	3.97E-06
ENSBTAT00000063482	ENSBTAG00000021048	ADM	15	42911789	42913325	-	CODING	-3.02724	4.05E-06
ENSBTAT00000026358	ENSBTAG00000019782	TPI1	5	1.04E+08	1.04E+08	-	CODING	-0.66463	4.08E-06
ENSBTAT00000035335	ENSBTAG00000012849	COL4A1	12	88876125	89009422	-	CODING	-1.43402	4.21E-06
ENSBTAT00000017146	ENSBTAG00000012898	RPS27L	10	46946054	46949421	+	CODING	-1.51853	4.44E-06
ENSBTAT00000022077	ENSBTAG00000016595	CYSTM1	7	53047810	53118491	+	CODING	-1.13562	4.45E-06
ENSBTAT00000019142	ENSBTAG00000014396	TIEG1	14	63951758	63957275	+	CODING	-1.15527	4.49E-06
ENSBTAT00000027369	ENSBTAG00000020541	PIGY	6	37677099	37679897	+	CODING	-1.22357	5.02E-06
ENSBTAT00000014625	ENSBTAG00000011011	SSH2	19	21443600	21499794	-	CODING	0.241834	5.03E-06
ENSBTAT00000017243	ENSBTAG00000012970	FAM53B	26	44619642	44693891	-	CODING	0.501976	5.23E-06
ENSBTAT00000011109	ENSBTAG00000008443	LARP1	7	67988299	68007480	+	CODING	0.038462	5.29E-06
ENSBTAT00000016666	ENSBTAG00000016457	FXR1	1	86678925	86751962	-	CODING	-0.2643	5.30E-06
ENSBTAT00000015348	ENSBTAG00000011548	AMPD1	3	28756908	28768496	+	CODING	-0.43741	5.36E-06
ENSBTAT00000000791	ENSBTAG00000000599	CCNI	6	93633306	93672214	-	CODING	-0.31531	5.55E-06
ENSBTAT00000010479	ENSBTAG00000007969	CIDEC	22	16909073	16918353	+	CODING	-2.5216	5.58E-06
ENSBTAT00000003636	ENSBTAG00000002808	PSMA3	10	70797328	70822594	+	CODING	-1.20484	5.60E-06
ENSBTAT00000021458	ENSBTAG00000016125	KCNA7	18	56038883	56041594	-	CODING	0.547292	5.77E-06
ENSBTAT00000063377	ENSBTAG00000046526	ISCA1	8	80824529	80836135	-	CODING	-0.37638	5.78E-06
ENSBTAT00000021330	ENSBTAG00000016026	PCOLCE2	1	1.27E+08	1.27E+08	+	CODING	-1.80854	5.96E-06
ENSBTAT00000040082	ENSBTAG00000016648	BSG	7	44816940	44823914	+	CODING	-1.12987	5.97E-06
ENSBTAT00000057533	ENSBTAG00000007662	GRP78	11	96115572	96119306	-	CODING	-1.37676	5.98E-06
ENSBTAT00000025637	ENSBTAG00000019255	NFE2L2	2	19659540	19692048	+	CODING	-1.31903	6.08E-06
ENSBTAT00000012098	ENSBTAG00000009181	INPP5A	26	51126574	51200732	-	CODING	0.073635	6.21E-06
ENSBTAT00000008019	ENSBTAG00000006101	PSMD4	3	19598421	19607517	-	CODING	-1.63557	6.24E-06
ENSBTAT00000039835	ENSBTAG00000014463	CAMK2D	6	12965129	13272298	+	CODING	-0.16574	6.44E-06

ENSBTAT00000006730	ENSBTAG00000005102	PHTF2	4	43549368	43641516	-	CODING	0.020377	6.51E-06
ENSBTAT00000026724	ENSBTAG00000020060	TXNIP	3	21489097	21493251	+	CODING	-1.00686	6.55E-06
ENSBTAT00000023566	ENSBTAG00000017719	AKAP6	21	43132861	43630707	+	CODING	0.462894	6.61E-06
ENSBTAT00000044059	ENSBTAG00000031134	YWHAH	17	72629359	72630106	+	CODING	-1.29937	6.83E-06
ENSBTAT00000003869	ENSBTAG00000002971	CUTC	26	20550908	20583501	+	CODING	-0.29855	6.85E-06
ENSBTAT00000003801	ENSBTAG00000023039	-	28	31044244	31048603	-	CODING	-1.37794	7.27E-06
ENSBTAT00000015961	ENSBTAG00000012032	PDE4A	7	16176856	16215655	+	CODING	-0.07605	7.49E-06
ENSBTAT00000028617	ENSBTAG00000021466	COL3A1	2	7318227	7356937	-	CODING	-0.27044	7.57E-06
ENSBTAT00000048322	ENSBTAG00000034077	ASIP	13	64234645	64239783	+	CODING	0.317501	7.64E-06
ENSBTAT00000053713	ENSBTAG00000014601	SGCB	6	69526335	69553387	-	CODING	-0.31872	7.88E-06
ENSBTAT00000043635	ENSBTAG00000008185	RTN2	18	53514400	53522925	-	CODING	-0.17952	8.08E-06
ENSBTAT00000047557	ENSBTAG00000033429	FAM229B	9	38816218	38821062	-	CODING	-2.11244	8.08E-06
ENSBTAT00000020065	ENSBTAG00000015074	PTGDS	11	1.06E+08	1.06E+08	-	CODING	-2.00333	8.66E-06
ENSBTAT00000060214	ENSBTAG00000043222	SNORA12	26	21014545	21014692	-	CODING	-5.58606	8.77E-06
ENSBTAT00000023246	ENSBTAG00000017475	GNAS	13	58010287	58049012	-	CODING	-1.32748	8.78E-06
ENSBTAT00000022378	ENSBTAG00000016822	PPIB	10	45874978	45880918	+	CODING	-1.45014	8.87E-06
ENSBTAT00000022636	ENSBTAG00000017024	PPARGC1A	6	44854113	44960533	-	CODING	-2.06838	8.96E-06
ENSBTAT00000022251	ENSBTAG00000016736	HIGD1B	19	45194104	45196727	+	CODING	-3.23287	9.18E-06
ENSBTAT00000012183	ENSBTAG00000009246	DPY30	11	14683785	14696398	-	CODING	-1.26776	9.38E-06
ENSBTAT00000024750	ENSBTAG00000018596	PTPN21	10	1.01E+08	1.01E+08	-	CODING	0.106196	9.39E-06
ENSBTAT00000061391	ENSBTAG00000023002	JPH2	13	73303266	73375173	-	CODING	0.350427	9.49E-06
ENSBTAT00000011979	ENSBTAG00000009087	GNG10	8	1.03E+08	1.03E+08	+	CODING	-1.28592	9.49E-06
ENSBTAT00000025311	ENSBTAG00000019015	IFITM3	29	51367009	51368065	+	CODING	-1.45737	9.87E-06
ENSBTAT00000017010	ENSBTAG00000012805	TSPAN13	4	25213088	25251241	+	CODING	-1.35773	1.01E-05
ENSBTAT00000013078	ENSBTAG00000009914	NDUFS6	20	70986115	70991390	-	CODING	-1.24693	1.01E-05
ENSBTAT00000039828	ENSBTAG00000027654	EIF4EBP1	27	32951594	32973435	+	CODING	-1.30305	1.04E-05
ENSBTAT00000024531	ENSBTAG00000018438	RRAGD	9	61644457	61743739	+	CODING	-0.22245	1.04E-05
ENSBTAT00000023670	ENSBTAG00000017802	THRB	27	41695665	41771738	+	CODING	0.332784	1.06E-05
ENSBTAT00000021401	ENSBTAG00000016081	PTMA	2	47998614	47999416	+	CODING	-1.70908	1.06E-05
ENSBTAT00000065106	ENSBTAG00000047752	OTUD1	13	24655214	24656659	+	CODING	-1.41037	1.07E-05
ENSBTAT00000025807	ENSBTAG00000019370	RAB21	X	97425377	97426021	+	CODING	-0.35017	1.09E-05
ENSBTAT00000000398	ENSBTAG00000000310	MFAP5	5	1.02E+08	1.02E+08	+	CODING	-1.36569	1.10E-05
ENSBTAT00000016871	ENSBTAG00000012694	UHMK1	3	6975867	6995683	-	CODING	0.245613	1.12E-05
ENSBTAT00000001069	ENSBTAG00000000808	COX14	5	29952087	29956382	-	CODING	-1.28001	1.13E-05
ENSBTAT00000007216	ENSBTAG00000005488	ARIH2	22	51572989	51610467	-	CODING	-0.33983	1.18E-05
ENSBTAT00000020298	ENSBTAG00000015258	-	19	24983919	24996301	-	CODING	-0.02266	1.20E-05
ENSBTAT00000029117	ENSBTAG00000021843	UBQLN2	X	98958218	98960092	+	CODING	-0.25376	1.21E-05
ENSBTAT00000027478	ENSBTAG00000020620	-	4	58724265	58724900	-	CODING	-1.69521	1.23E-05
ENSBTAT00000031821	ENSBTAG00000030592	UBL5	1	1.54E+08	1.54E+08	+	CODING	-1.35899	1.24E-05
ENSBTAT00000026002	ENSBTAG00000019521	COX6A2	25	27737582	27738205	-	CODING	-0.99627	1.26E-05
ENSBTAT00000023750	ENSBTAG00000017866	CD36	4	40581484	40643369	-	CODING	-1.08327	1.27E-05
ENSBTAT00000065469	ENSBTAG00000011731	PNMT	19	40694079	40695765	+	CODING	-3.6547	1.29E-05
ENSBTAT00000037657	ENSBTAG00000004048	MCHR1	5	1.13E+08	1.13E+08	+	CODING	0.627235	1.34E-05
ENSBTAT00000002938	ENSBTAG00000002279	LUC7L3	19	36622184	36647084	-	CODING	-1.4286	1.36E-05
ENSBTAT00000011818	ENSBTAG00000008978	USP22	19	35728070	35746498	-	CODING	-0.22434	1.37E-05
ENSBTAT00000009545	ENSBTAG00000007256	DYRK2	5	46323523	46333943	-	CODING	0.408991	1.38E-05
ENSBTAT00000009600	ENSBTAG00000007296	-	1	82549642	82550013	+	CODING	-5.52653	1.39E-05
ENSBTAT00000015802	ENSBTAG00000011912	SLC25A25	11	98750459	98760803	+	CODING	-1.53067	1.40E-05
ENSBTAT00000002092	ENSBTAG00000038067	MT1A	18	24106722	24108521	-	CODING	-2.46617	1.46E-05
ENSBTAT00000027111	ENSBTAG00000020342	MYOC	16	39957137	39971282	-	CODING	-1.82785	1.46E-05
ENSBTAT00000014665	ENSBTAG00000011045	MRPS36	20	10390982	10399140	-	CODING	-0.23824	1.46E-05
ENSBTAT00000033843	ENSBTAG00000024407	-	3	19259499	19260488	+	CODING	-1.93105	1.48E-05
ENSBTAT00000018555	ENSBTAG00000013956	BCL2L13	5	1.1E+08	1.1E+08	+	CODING	-0.32098	1.58E-05
ENSBTAT00000029301	ENSBTAG00000021976	NDUFS1	2	94893227	94922667	-	CODING	-0.39278	1.58E-05
ENSBTAT00000016756	ENSBTAG00000012622	TRA2A	4	32250996	32269539	-	CODING	-1.6019	1.58E-05
ENSBTAT00000004161	ENSBTAG00000003205	RPL35	11	95850116	95854734	-	CODING	-1.15704	1.58E-05
ENSBTAT00000048021	ENSBTAG00000033835	MPZ	3	8229236	8234089	+	CODING	-1.56062	1.64E-05
ENSBTAT00000002326	ENSBTAG00000001777	RPLP2	29	50768654	50770791	-	CODING	-1.08805	1.68E-05
ENSBTAT00000054742	ENSBTAG00000037470	-	14	38614593	38615161	+	CODING	-1.97788	1.70E-05
ENSBTAT00000009693	ENSBTAG00000007371	SCAMP1	10	9369310	9520700	+	CODING	0.115183	1.85E-05
ENSBTAT00000009484	ENSBTAG00000007211	ASB12	X	1.01E+08	1.01E+08	+	CODING	-0.57202	1.87E-05

ENSBTAT00000028574	ENSBTAG00000021435	MAFF	5	1.1E+08	1.1E+08	+	CODING	-1.43904	1.87E-05
ENSBTAT00000045780	ENSBTAG00000008192	-	7	16982664	16990838	+	CODING	-2.56242	1.87E-05
ENSBTAT00000007053	ENSBTAG00000005359	TGFB2	16	22495736	22588280	+	CODING	0.219723	1.92E-05
ENSBTAT00000016153	ENSBTAG00000012177	SNRPD2	18	53681070	53683928	-	CODING	-1.35321	1.92E-05
ENSBTAT00000002491	ENSBTAG00000003949	RBM39	13	65567208	65592912	-	CODING	-1.36778	1.92E-05
ENSBTAT00000056211	ENSBTAG00000038241	REPIN1	4	1.14E+08	1.14E+08	+	CODING	0.168751	1.97E-05
ENSBTAT00000002932	ENSBTAG00000002271	CDADC1	12	18963010	18990944	+	CODING	-0.04334	1.97E-05
ENSBTAT00000006593	ENSBTAG00000005008	WSB1	19	19356951	19371161	+	CODING	-1.58657	2.04E-05
ENSBTAT00000012779	ENSBTAG00000009689	RWDD1	9	34577932	34595665	-	CODING	-1.15796	2.05E-05
ENSBTAT00000003229	ENSBTAG00000002485	TNS1	2	1.07E+08	1.07E+08	-	CODING	-0.17729	2.11E-05
ENSBTAT00000010762	ENSBTAG00000008182	FOSB	18	53501995	53508395	+	CODING	-0.16544	2.11E-05
ENSBTAT00000002583	ENSBTAG00000030301	MAP7D1	3	1.1E+08	1.1E+08	-	CODING	-0.16045	2.13E-05
ENSBTAT00000009803	ENSBTAG00000007454	RPL10	X	40364802	40367164	+	CODING	-1.01391	2.14E-05
ENSBTAT00000002122	ENSBTAG00000001618	ALPK3	21	22883305	22935573	+	CODING	-0.32945	2.14E-05
ENSBTAT00000065882	ENSBTAG00000014132	SNED1	3	1.21E+08	1.21E+08	+	CODING	-4.06014	2.15E-05
ENSBTAT00000014894	ENSBTAG00000011215	ACTN4	18	48668482	48741185	+	CODING	-1.52925	2.16E-05
ENSBTAT00000036888	ENSBTAG00000026199	ACTB	25	39343633	39347044	+	CODING	-1.01569	2.21E-05
ENSBTAT00000054758	ENSBTAG00000039121	PTP4A2	2	1.22E+08	1.22E+08	+	CODING	-0.65026	2.22E-05
ENSBTAT00000013537	ENSBTAG00000010244	CLIC5	23	19167358	19333272	-	CODING	-0.03174	2.24E-05
ENSBTAT00000021523	ENSBTAG00000016170	KCNJ11	15	35650715	35653362	+	CODING	-0.06773	2.24E-05
ENSBTAT00000026309	ENSBTAG00000019742	FOXJ2	5	1.02E+08	1.02E+08	+	CODING	0.221602	2.37E-05
ENSBTAT00000022970	ENSBTAG00000017279	VAPA	24	42332694	42368659	+	CODING	-0.50975	2.39E-05
ENSBTAT00000000683	ENSBTAG00000000524	CSTB	1	1.47E+08	1.47E+08	-	CODING	-1.85865	2.41E-05
ENSBTAT00000026100	ENSBTAG00000019588	BOLA-DQB	23	25855146	25863045	-	CODING	0.317222	2.42E-05
ENSBTAT00000025389	ENSBTAG00000019070	PMP22	19	33357141	33382707	-	CODING	-1.47824	2.43E-05
ENSBTAT00000001253	ENSBTAG00000000948	RAB2A	14	27864735	27937015	+	CODING	-1.13131	2.48E-05
ENSBTAT00000015797	ENSBTAG00000011909	ACVR1	2	39287889	39361498	+	CODING	0.103184	2.55E-05
ENSBTAT00000009405	ENSBTAG00000007147	CUEDC1	19	8900990	8974286	-	CODING	-0.10382	2.59E-05
ENSBTAT00000004843	ENSBTAG00000003718	HACL1	1	1.54E+08	1.54E+08	-	CODING	-1.72695	2.60E-05
ENSBTAT00000040095	ENSBTAG00000008665	DIXDC1	15	22614860	22659906	+	CODING	0.637112	2.60E-05
ENSBTAT00000022232	ENSBTAG00000016724	NPEPL1	13	58207245	58222951	-	CODING	-0.14591	2.70E-05
ENSBTAT00000021796	ENSBTAG00000016391	-	9	16325798	16326091	-	CODING	-1.05966	2.78E-05
ENSBTAT00000047298	ENSBTAG00000033284	CHCHD7	14	25052885	25058779	+	CODING	-1.33632	2.93E-05
ENSBTAT00000063866	ENSBTAG00000045504	-	9	42351250	42352038	-	CODING	-1.12814	2.93E-05
ENSBTAT00000007324	ENSBTAG00000005574	CLU	8	11043941	11061301	+	CODING	-1.28461	3.00E-05
ENSBTAT00000026197	ENSBTAG00000019658	ASB16	19	44614052	44621587	+	CODING	-0.44361	3.03E-05
ENSBTAT00000024371	ENSBTAG00000031998	CXCL16	19	27249528	27253097	+	CODING	-2.0614	3.05E-05
ENSBTAT00000010108	ENSBTAG00000007685	PSMB1	9	1.06E+08	1.06E+08	-	CODING	-1.18615	3.24E-05
ENSBTAT00000013310	ENSBTAG00000010083	HADHB	11	73215424	73246167	-	CODING	-1.21361	3.31E-05
ENSBTAT00000046700	ENSBTAG00000005339	-	23	17263646	17269998	+	CODING	-1.62405	3.32E-05
ENSBTAT00000018566	ENSBTAG00000013953	CALD1	4	99475015	99580189	+	CODING	-1.41555	3.38E-05
ENSBTAT00000000144	ENSBTAG00000000132	EIF4A1	19	27915413	27921421	+	CODING	-1.40329	3.39E-05
ENSBTAT00000038151	ENSBTAG00000021653	TRIP12	2	1.19E+08	1.19E+08	-	CODING	-0.14748	3.39E-05
ENSBTAT00000014877	ENSBTAG00000023462	RPS6	8	24927468	24930788	+	CODING	-1.40672	3.41E-05
ENSBTAT00000028499	ENSBTAG00000021378	S100A13	3	16818414	16824125	+	CODING	-1.64437	3.44E-05
ENSBTAT00000010914	ENSBTAG00000008294	KCNJ2	19	61185603	61195897	-	CODING	0.625442	3.46E-05
ENSBTAT00000011677	ENSBTAG00000008868	CAPN3	10	37828797	37885860	+	CODING	-0.51126	3.53E-05
ENSBTAT00000025416	ENSBTAG00000019091	RNPC3	3	39998006	40025400	-	CODING	-1.90496	3.70E-05
ENSBTAT00000010384	ENSBTAG00000007895	SLC20A1	11	46218716	46232124	+	CODING	-1.60684	3.86E-05
ENSBTAT00000014594	ENSBTAG00000010989	PIK3R1	20	11329104	11409885	-	CODING	-0.1555	3.88E-05
ENSBTAT00000063200	ENSBTAG00000046117	TMSB4X	11	63290422	63395507	+	CODING	-1.42868	3.90E-05
ENSBTAT00000012773	ENSBTAG00000009683	PSMA6	21	45974822	45998591	+	CODING	-1.17609	3.93E-05
ENSBTAT00000003259	ENSBTAG00000002507	ATP5A1	24	46300459	46309366	-	CODING	-0.62215	3.95E-05
ENSBTAT00000061351	ENSBTAG00000032477	HECTD1	21	42121456	42196305	-	CODING	-0.30056	3.97E-05
ENSBTAT00000017991	ENSBTAG00000013530	DDAH2	23	27399306	27402825	+	CODING	-2.18604	4.06E-05
ENSBTAT00000024373	ENSBTAG00000018317	PPP2R2A	8	74834628	74913332	+	CODING	-0.26345	4.25E-05
ENSBTAT00000053293	ENSBTAG00000037415	COX7B	17	60146304	60146762	+	CODING	-1.30725	4.28E-05
ENSBTAT00000066024	ENSBTAG00000045548	CRKL	17	74286482	74297800	+	CODING	-0.10075	4.41E-05
ENSBTAT00000017744	ENSBTAG00000013341	GYG2	X	1.4E+08	1.4E+08	-	CODING	0.409205	4.45E-05
ENSBTAT00000039242	ENSBTAG00000003512	MYH7B	13	64888626	64912758	+	CODING	-1.42364	4.59E-05
ENSBTAT00000037367	ENSBTAG00000026327	RPL8	14	1505030	1507633	-	CODING	-1.10201	4.69E-05

ENSBTAT00000043235	ENSBTAG00000010982	UBE2B	7	47528581	47541198	+	CODING	-1.01308	4.78E-05
ENSBTAT00000061240	ENSBTAG00000012882	CUL5	15	17857169	17983781	+	CODING	-0.1216	4.85E-05
ENSBTAT00000006337	ENSBTAG00000004824	REEP1	11	48389168	48525009	+	CODING	-0.09273	4.95E-05
ENSBTAT00000026036	ENSBTAG00000019543	TCEB2	25	2281945	2285931	+	CODING	-1.25794	5.09E-05
ENSBTAT00000057357	ENSBTAG00000008401	PFKFB3	13	17380743	17406594	-	CODING	-1.06835	5.10E-05
ENSBTAT00000027125	ENSBTAG00000020355	KLF4	8	98843684	98847740	-	CODING	-1.33753	5.18E-05
ENSBTAT00000064571	ENSBTAG00000046684	FOXN3	10	1.02E+08	1.02E+08	-	CODING	0.030755	5.19E-05
ENSBTAT00000006573	ENSBTAG00000004992	TNPO3	4	93722331	93800500	-	CODING	0.009379	5.24E-05
ENSBTAT00000055220	ENSBTAG00000033008	MYOZ1	28	29780787	29798613	-	CODING	-0.70652	5.30E-05
ENSBTAT00000000993	ENSBTAG00000000745	AQP1	4	65830992	65845186	-	CODING	-1.46588	5.40E-05
ENSBTAT00000017175	ENSBTAG00000012926	TM6SF1	21	25394313	25424181	-	CODING	-0.42403	5.45E-05
ENSBTAT00000063767	ENSBTAG00000045608	-	28	3273926	3274777	-	CODING	0.04116	5.64E-05
ENSBTAT00000029304	ENSBTAG00000021979	EEF1B	2	94922839	94926122	+	CODING	-1.07726	5.65E-05
ENSBTAT00000038642	ENSBTAG00000026995	PNN	21	49612519	49618556	+	CODING	-1.44662	5.88E-05
ENSBTAT00000020080	ENSBTAG00000015089	LGALS1	5	1.1E+08	1.1E+08	+	CODING	-0.55574	5.88E-05
ENSBTAT00000001560	ENSBTAG00000001176	LRRN1	22	22576210	22578360	-	CODING	0.036331	5.94E-05
ENSBTAT00000024915	ENSBTAG00000018718	DDI2	16	53135568	53168210	-	CODING	0.349891	5.96E-05
ENSBTAT00000016242	ENSBTAG00000012244	TUBA1B	5	30864529	30868119	+	CODING	-1.16459	5.97E-05
ENSBTAT00000026452	ENSBTAG00000019857	OTUD4	17	13286959	13327626	+	CODING	0.155995	6.12E-05
ENSBTAT00000006947	ENSBTAG00000005280	ADA	13	73750479	73773983	-	CODING	-1.69072	6.13E-05
ENSBTAT00000003190	ENSBTAG00000002457	SEC61B	8	64722966	64731269	+	CODING	-1.74426	6.13E-05
ENSBTAT00000013288	ENSBTAG00000010073	FLOT2	19	20811180	20815216	-	CODING	-0.20109	6.21E-05
ENSBTAT00000013399	ENSBTAG00000010155	-	19	14735718	14741999	+	CODING	-3.05123	6.24E-05
ENSBTAT00000008467	ENSBTAG00000006457	AHSP	25	27801696	27805181	+	CODING	-5.31426	6.34E-05
ENSBTAT00000024919	ENSBTAG00000018722	PLEKHM2	16	53075569	53115375	-	CODING	-0.23405	6.36E-05
ENSBTAT00000000909	ENSBTAG00000000684	ARHGEF10L	2	1.36E+08	1.36E+08	-	CODING	0.104416	6.38E-05
ENSBTAT00000026999	ENSBTAG00000020263	TIMP4	22	57602210	57609085	+	CODING	-1.56587	6.55E-05
ENSBTAT00000008894	ENSBTAG00000006759	SUCLA2	12	17884901	17930933	-	CODING	-0.49386	6.58E-05
ENSBTAT00000027191	ENSBTAG00000020405	NDUFB9	14	17132669	17140075	-	CODING	-1.11417	6.58E-05
ENSBTAT00000046614	ENSBTAG00000014874	FYTTD1	1	70954511	70984201	-	CODING	-0.28784	6.59E-05
ENSBTAT00000007057	ENSBTAG00000005354	LRRFIP1	3	1.18E+08	1.18E+08	+	CODING	-0.1402	6.60E-05
ENSBTAT00000026126	ENSBTAG00000045492	ANG	10	26429018	26445589	-	CODING	-1.83989	6.73E-05
ENSBTAT00000023595	ENSBTAG00000017745	IL6ST	20	23212633	23270316	+	CODING	-0.07906	6.74E-05
ENSBTAT00000002349	ENSBTAG00000001794	RPL36	7	19833364	19834615	-	CODING	-1.19547	6.97E-05
ENSBTAT00000024018	ENSBTAG00000018043	LCAT	18	35544370	35547611	-	CODING	-2.21352	7.00E-05
ENSBTAT00000061365	ENSBTAG00000021830	ENPP1	9	70734253	70804135	+	CODING	-1.74292	7.01E-05
ENSBTAT00000049560	ENSBTAG00000035083	ATXN7L3B	5	4048888	4052625	+	CODING	-0.10235	7.04E-05
ENSBTAT00000012977	ENSBTAG00000009844	CYR61	3	58678779	58681686	-	CODING	-1.1895	7.17E-05
ENSBTAT00000021636	ENSBTAG00000016266	SDHD	15	22722376	22733137	+	CODING	-0.43728	7.21E-05
ENSBTAT00000044086	ENSBTAG00000015273	CAND2	22	56990323	57017221	-	CODING	-0.14999	7.23E-05
ENSBTAT00000027612	ENSBTAG00000020720	MMTAG2	7	2910375	2912630	+	CODING	-1.93725	7.28E-05
ENSBTAT00000028269	ENSBTAG00000021218	MYLPF	25	26815266	26817727	+	CODING	-0.76607	7.39E-05
ENSBTAT00000044859	ENSBTAG00000031641	PSMD8	18	48451832	48457821	+	CODING	-1.35248	7.44E-05
ENSBTAT00000018777	ENSBTAG00000014129	RAB5B	5	57648526	57651738	-	CODING	-0.20873	7.59E-05
ENSBTAT00000002861	ENSBTAG00000002210	LAS1L	X	1E+08	1E+08	+	CODING	-1.6486	7.59E-05
ENSBTAT00000000843	ENSBTAG00000000641	SKAP1	19	38697518	39001101	+	CODING	0.577817	7.69E-05
ENSBTAT00000021617	ENSBTAG00000016250	AKTIP	18	21926577	21937955	-	CODING	-0.07165	7.78E-05
ENSBTAT00000006908	ENSBTAG00000005249	RNF14	7	54717280	54735688	+	CODING	-0.15792	7.81E-05
ENSBTAT00000005170	ENSBTAG00000003966	DUSP3	19	44281113	44294691	-	CODING	-0.27472	8.19E-05
ENSBTAT00000000179	ENSBTAG00000000156	LGALSL	11	62812719	62817829	+	CODING	-0.07411	8.44E-05
ENSBTAT00000012655	ENSBTAG00000009615	ANXA2	10	49860062	49904536	+	CODING	-1.28282	8.52E-05
ENSBTAT00000024301	ENSBTAG00000018255	ACTN1	10	81023526	81121590	-	CODING	-1.85943	8.52E-05
ENSBTAT00000043690	ENSBTAG00000017713	KTN1	10	68238010	68351879	+	CODING	-0.27424	8.61E-05
ENSBTAT00000010474	ENSBTAG00000007962	ATP9A	13	80167668	80262763	-	CODING	0.249859	8.62E-05
ENSBTAT00000027338	ENSBTAG00000020520	RASD1	19	35423867	35425459	+	CODING	-2.73617	8.67E-05
ENSBTAT00000005445	ENSBTAG00000004159	SIX2	11	27260475	27263699	-	CODING	0.995871	8.68E-05
ENSBTAT00000015617	ENSBTAG00000011758	-	5	1.05E+08	1.05E+08	+	CODING	-1.25345	8.72E-05
ENSBTAT00000021156	ENSBTAG00000015910	ITGB1	13	20248978	20290982	+	CODING	-1.51456	8.81E-05
ENSBTAT00000017516	ENSBTAG00000013166	SMYD2	16	70760234	70813706	-	CODING	-0.27676	8.97E-05
ENSBTAT00000040802	ENSBTAG00000028421	U1	19	15193841	15194001	-	CODING	-5.26107	8.99E-05
ENSBTAT00000001490	ENSBTAG00000001120	CORO6	19	21426533	21434772	-	CODING	-0.52794	9.05E-05

ENSBTAT00000032427	ENSBTAG00000000605	ATP5J	1	10069680	10076995	+	CODING	-1.29716	9.17E-05
ENSBTAT00000037567	ENSBTAG00000026437	ULBP3	17	39881230	39884057	+	CODING	0.094199	9.18E-05
ENSBTAT00000020755	ENSBTAG00000015625	DCTN4	7	64076578	64107651	-	CODING	-0.09264	9.32E-05
ENSBTAT00000018408	ENSBTAG00000013859	SCYL2	5	64560031	64613964	+	CODING	0.018491	9.42E-05
ENSBTAT00000065244	ENSBTAG00000013479	SLC9A3R2	25	1575649	1589619	+	CODING	-2.06064	9.45E-05
ENSBTAT00000065583	ENSBTAG00000047495	CD81	29	49842969	49848639	-	CODING	-1.23013	9.49E-05
ENSBTAT00000057198	ENSBTAG00000008001	NUCKS1	16	3301673	3329235	-	CODING	-0.32963	9.71E-05
ENSBTAT00000001098	ENSBTAG00000000833	TAX1BP3	19	24971932	24977062	-	CODING	-1.54106	9.87E-05
ENSBTAT00000021045	ENSBTAG00000015839	MAP4	22	52369974	52462511	+	CODING	-0.3669	0.000102
ENSBTAT00000008382	ENSBTAG00000006391	NDUFAB1	25	21498279	21509168	-	CODING	-1.10272	0.000103
ENSBTAT00000008847	ENSBTAG00000006729	ARID5B	28	18003736	18191994	+	CODING	-1.7543	0.000104
ENSBTAT00000028825	ENSBTAG00000021632	MSRA	8	8635550	9019576	-	CODING	-0.49425	0.000105
ENSBTAT00000010884	ENSBTAG00000008271	MEDAG	12	30021743	30039901	-	CODING	-1.92802	0.000107
ENSBTAT00000063682	ENSBTAG00000047418	SLC25A6	GJ06	1867	6353	+	CODING	-1.31245	0.000108
ENSBTAT00000061118	ENSBTAG00000015376	FBXW11	20	3584765	3624526	-	CODING	-0.04982	0.000109
ENSBTAT00000056230	ENSBTAG00000037661	DENND1B	16	78480806	78590234	-	CODING	0.28921	0.000112
ENSBTAT00000057593	ENSBTAG00000019517	ELN	25	33787889	33820672	-	CODING	-1.85087	0.000112
ENSBTAT00000017503	ENSBTAG00000003418	MSN	X	1E+08	1E+08	-	CODING	-1.37351	0.000115
ENSBTAT00000065774	ENSBTAG00000046712	PSMC2	4	44806721	44819866	+	CODING	-1.13409	0.000116
ENSBTAT00000006407	ENSBTAG00000004873	CCNL2	16	52388692	52396825	+	CODING	-1.89317	0.000116
ENSBTAT00000018663	ENSBTAG00000014041	G3BP2	6	92205907	92241546	-	CODING	-0.29502	0.000117
ENSBTAT00000021827	ENSBTAG00000016411	RNF122	27	28450185	28459083	-	CODING	0.026971	0.000119
ENSBTAT00000049039	ENSBTAG00000005742	CYYR1	1	9242710	9250037	+	CODING	-1.41035	0.000121
ENSBTAT00000040201	ENSBTAG00000027879	MPC1	9	1.03E+08	1.03E+08	-	CODING	-1.10036	0.000122
ENSBTAT00000050132	ENSBTAG00000020218	ANXA7	28	29552050	29573976	-	CODING	-0.2539	0.000124
ENSBTAT00000055318	ENSBTAG00000008732	ZC3H12C	15	20493944	20526349	+	CODING	1.008733	0.000125
ENSBTAT00000014213	ENSBTAG00000010735	HIGD2A	7	39207845	39208828	+	CODING	-0.41764	0.000125
ENSBTAT00000005102	ENSBTAG00000003907	TSPAN14	28	36077428	36109014	+	CODING	-1.55716	0.000126
ENSBTAT00000051493	ENSBTAG00000036993	U2	2	48352291	48352468	-	CODING	4.321241	0.000127
ENSBTAT00000028330	ENSBTAG00000021262	SNRPD3	17	73479566	73484880	-	CODING	-1.36402	0.000128
ENSBTAT00000012241	ENSBTAG00000009292	TMEM100	19	6237391	6239838	-	CODING	-2.292	0.000128
ENSBTAT00000065262	ENSBTAG00000047051	-	X	36171714	36172838	+	CODING	-1.18026	0.000129
ENSBTAT00000015490	ENSBTAG00000011666	THRSP	29	18084681	18090595	-	CODING	-2.63488	0.000132
ENSBTAT00000026443	ENSBTAG00000019847	CPEB2	6	1.15E+08	1.15E+08	+	CODING	0.268719	0.000132
ENSBTAT00000034879	ENSBTAG00000025005	METTL7A	5	29161683	29172757	-	CODING	-0.09554	0.000132
ENSBTAT00000056537	ENSBTAG00000037571	EBAG9	14	57006997	57020071	-	CODING	-0.33216	0.000132
ENSBTAT00000020250	ENSBTAG00000015220	AGO4	3	1.11E+08	1.11E+08	-	CODING	0.333697	0.000136
ENSBTAT00000063342	ENSBTAG00000047376	PIN4	X	83607426	83616158	-	CODING	-1.25609	0.000136
ENSBTAT00000009325	ENSBTAG00000007089	FRG1	27	18244331	18258187	+	CODING	-1.43974	0.00014
ENSBTAT00000019134	ENSBTAG00000014388	EIF3I	2	1.22E+08	1.22E+08	-	CODING	-1.19702	0.00014
ENSBTAT00000002946	ENSBTAG00000002283	FZD7	2	90987008	90989214	+	CODING	0.108022	0.00014
ENSBTAT00000013127	ENSBTAG00000009951	PGP	25	1746200	1747344	-	CODING	-0.07138	0.000141
ENSBTAT00000026228	ENSBTAG00000019680	ZYG11B	3	94123286	94197896	-	CODING	-0.13199	0.000142
ENSBTAT00000044712	ENSBTAG00000031544	DDIT3	5	56285008	56289214	+	CODING	-1.75473	0.000142
ENSBTAT00000020705	ENSBTAG00000015584	BMI1	13	23709778	23712913	+	CODING	-0.26475	0.000144
ENSBTAT00000027250	ENSBTAG00000020449	ETFB	18	57795757	57808326	-	CODING	-1.24124	0.000144
ENSBTAT00000023784	ENSBTAG00000017896	ST8SIA2	21	14879467	14949980	-	CODING	1.329618	0.000146
ENSBTAT00000064873	ENSBTAG00000000979	SMIM19	27	37055089	37070241	+	CODING	-0.32903	0.000146
ENSBTAT00000007531	ENSBTAG00000005729	FBXL4	9	51485365	51560823	+	CODING	-0.03543	0.000148
ENSBTAT00000021303	ENSBTAG00000016004	METAP1	6	26925349	26979063	-	CODING	-0.01897	0.00015
ENSBTAT00000023967	ENSBTAG00000023289	SLC26A10	5	56214540	56219323	+	CODING	-2.21405	0.000154
ENSBTAT00000027511	ENSBTAG00000020645	GNAI2	22	50670852	50691007	-	CODING	-1.51594	0.000155
ENSBTAT00000024427	ENSBTAG00000018362	TMEM109	29	37833099	37841873	+	CODING	-0.37405	0.000159
ENSBTAT00000008511	ENSBTAG00000014540	PERM1	16	52742637	52748240	+	CODING	-0.37139	0.00016
ENSBTAT00000004242	ENSBTAG00000003275	ANK1	27	36295590	36355927	-	CODING	0.439112	0.00016
ENSBTAT00000008682	ENSBTAG00000006618	HLF	19	5696798	5747138	+	CODING	0.223403	0.000161
ENSBTAT00000008572	ENSBTAG00000006526	BCL2L1	13	61766806	61817383	-	CODING	-1.40146	0.000162
ENSBTAT00000004198	ENSBTAG00000003238	MEOX2	4	23943520	24019359	-	CODING	-1.57944	0.000162
ENSBTAT00000007621	ENSBTAG00000005796	-	3	9538550	9576282	+	CODING	-0.34042	0.000163
ENSBTAT00000018461	ENSBTAG00000013901	PTDSS1	14	70236070	70303427	-	CODING	-0.1411	0.000164
ENSBTAT00000025024	ENSBTAG00000018800	RPS4	X	83532034	83536488	+	CODING	-1.13942	0.000165

ENSBTAT00000061638	ENSBTAG00000015361	CNTFR	8	77260361	77298847	-	CODING	-2.00959	0.000166
ENSBTAT00000026501	ENSBTAG00000019891	MRPL40	17	74708565	74711079	+	CODING	-1.33762	0.000166
ENSBTAT00000004243	ENSBTAG00000003278	HOXC10	5	26202176	26206357	-	CODING	-0.03434	0.000168
ENSBTAT00000010660	ENSBTAG00000008105	RBM38	13	59294500	59308857	-	CODING	-1.19688	0.00017
ENSBTAT00000061043	ENSBTAG00000016990	USP24	3	91711045	91866000	+	CODING	0.163817	0.00017
ENSBTAT00000022710	ENSBTAG00000017077	CTSL	8	84976255	84981470	-	CODING	-1.37763	0.000171
ENSBTAT00000004031	ENSBTAG00000003098	MTDH	14	68799572	68853740	-	CODING	-0.36496	0.000173
ENSBTAT00000012542	ENSBTAG00000009534	NDUFB10	25	1517626	1520287	+	CODING	-1.05935	0.000174
ENSBTAT00000021638	ENSBTAG00000016267	SERPING1	15	82159474	82172143	+	CODING	-1.48878	0.000179
ENSBTAT00000021887	ENSBTAG00000006884	PABPN1	10	21427137	21434016	-	CODING	-1.80795	0.000181
ENSBTAT00000005688	ENSBTAG00000004348	MRPL21	29	46913943	46923884	-	CODING	-1.49612	0.00019
ENSBTAT00000016160	ENSBTAG00000009789	GNAQ	8	53970972	54280697	-	CODING	0.209611	0.000193
ENSBTAT00000005615	ENSBTAG00000004283	PPFIBP1	5	82542192	82609403	-	CODING	-0.00278	0.000198
ENSBTAT00000012560	ENSBTAG00000009547	ZDHHC4	25	38953354	38961974	+	CODING	-0.42708	0.0002
ENSBTAT00000065587	ENSBTAG00000047374	-	5	56217962	56220534	+	CODING	-2.28086	0.0002
ENSBTAT00000002429	ENSBTAG00000001864	NR4A3	8	65341202	65373695	+	CODING	-1.74328	0.000201
ENSBTAT00000043067	ENSBTAG00000030520	PROB1	7	52290536	52293580	-	CODING	0.109858	0.000203
ENSBTAT00000023485	ENSBTAG00000017655	PALMD	3	43688103	43748131	-	CODING	-0.42166	0.000205
ENSBTAT00000057496	ENSBTAG00000039329	RAET1G	9	88232044	88402262	-	CODING	0.669208	0.000208
ENSBTAT00000006265	ENSBTAG00000004770	SCN4A	19	48794399	48825660	-	CODING	-0.18933	0.000212
ENSBTAT00000002653	ENSBTAG00000002048	PTPN11	17	64010592	64056567	-	CODING	-0.13514	0.000218
ENSBTAT00000033724	ENSBTAG00000007767	TBX15	3	24236815	24329679	+	CODING	0.019111	0.000221
ENSBTAT00000035299	ENSBTAG00000020928	ADPRHL1	12	90663887	90676882	-	CODING	-0.26482	0.000221
ENSBTAT00000019994	ENSBTAG00000015018	FSD2	21	23501906	23537791	-	CODING	-0.13817	0.000223
ENSBTAT00000008142	ENSBTAG00000006199	DRAP1	29	44694171	44696295	+	CODING	-1.10647	0.000226
ENSBTAT00000054652	ENSBTAG00000001729	DUSP10	16	25895839	25936864	-	CODING	0.063248	0.000226
ENSBTAT00000015648	ENSBTAG00000011787	ISCU	17	66685452	66691553	+	CODING	-1.1676	0.000227
ENSBTAT00000002634	ENSBTAG00000002033	UBXN2A	11	75101825	75130698	-	CODING	0.006201	0.00023
ENSBTAT00000014056	ENSBTAG00000018790	BVES	9	45484429	45522649	+	CODING	-0.0528	0.000231
ENSBTAT00000006715	ENSBTAG00000005096	COX7A2	9	15041836	15046972	-	CODING	-1.91495	0.000233
ENSBTAT00000024656	ENSBTAG00000018522	OAZ1	7	22577243	22580777	-	CODING	-1.00186	0.000235
ENSBTAT00000056395	ENSBTAG00000038630	KLHL34	X	1.29E+08	1.29E+08	+	CODING	0.209735	0.000239
ENSBTAT00000029018	ENSBTAG00000021769	CUL3	2	1.13E+08	1.13E+08	-	CODING	-0.3527	0.000241
ENSBTAT00000029284	ENSBTAG00000021965	SUB1	20	41122023	41143914	-	CODING	-0.348	0.000242
ENSBTAT00000038024	ENSBTAG00000011582	SERINC2	2	1.23E+08	1.23E+08	+	CODING	-0.35912	0.000245
ENSBTAT00000020512	ENSBTAG00000015434	DSTN	13	38259752	38284536	+	CODING	-1.27762	0.000246
ENSBTAT00000046331	ENSBTAG00000018071	TMOD4	3	19679350	19683836	+	CODING	-0.67504	0.000246
ENSBTAT00000004701	ENSBTAG00000003609	PBDC1	X	80576367	80580439	-	CODING	-1.41403	0.000249
ENSBTAT00000007369	ENSBTAG00000023487	COX6B1	5	94363786	94364264	+	CODING	-0.5775	0.000254
ENSBTAT00000005267	ENSBTAG00000004034	SESN3	15	15502203	15523079	+	CODING	0.31558	0.000255
ENSBTAT00000021569	ENSBTAG00000016208	TGM2	13	67663138	67697607	-	CODING	-1.5739	0.000255
ENSBTAT00000016712	ENSBTAG00000012589	HSPE1	2	86449525	86451564	+	CODING	-1.0384	0.000257
ENSBTAT00000004401	ENSBTAG00000003395	ZRANB1	26	44841835	44885237	+	CODING	-0.184	0.000258
ENSBTAT00000002103	ENSBTAG00000001604	-	7	2586101	2598115	-	CODING	-0.42437	0.000258
ENSBTAT00000061037	ENSBTAG00000044126	SNTB1	14	84253919	84504093	-	CODING	-0.23482	0.00026
ENSBTAT00000050390	ENSBTAG00000006242	USP9X	X	1.08E+08	1.08E+08	-	CODING	-0.20348	0.000262
ENSBTAT00000037140	ENSBTAG00000020439	BUD31	25	37524461	37532400	-	CODING	-1.31123	0.000264
ENSBTAT00000016768	ENSBTAG00000012634	NDUFB7	7	12325362	12329750	+	CODING	-1.12427	0.000265
ENSBTAT00000004594	ENSBTAG00000023823	CYCS	11	98339924	98340241	-	CODING	-0.59041	0.000266
ENSBTAT00000027115	ENSBTAG00000020345	CNN3	3	48763975	48794136	+	CODING	-1.73055	0.000267
ENSBTAT00000023909	ENSBTAG00000017969	CA4	19	13109655	13117426	-	CODING	-2.07681	0.000269
ENSBTAT00000036270	ENSBTAG00000016874	DNAJB1	7	12366856	12370535	+	CODING	-1.4287	0.00027
ENSBTAT00000014642	ENSBTAG00000011022	ARPP19	10	57932635	57938671	+	CODING	-0.48343	0.000272
ENSBTAT00000063501	ENSBTAG00000014861	SLC20A2	27	36938387	37017424	-	CODING	-2.86473	0.000274
ENSBTAT00000011658	ENSBTAG00000008853	HNRNPF	28	13902311	13920905	-	CODING	-1.1491	0.000275
ENSBTAT00000029418	ENSBTAG00000022020	CLDN5	17	74749250	74750524	-	CODING	-1.50554	0.000275
ENSBTAT00000022698	ENSBTAG00000017071	C1QTNF3	20	39760050	39792323	+	CODING	-1.65904	0.000277
ENSBTAT00000048673	ENSBTAG00000012307	DTNA	24	22521988	22766995	-	CODING	-0.35801	0.000278
ENSBTAT00000019913	ENSBTAG00000014956	JSRP1	7	22700027	22703913	-	CODING	-0.55388	0.00028
ENSBTAT00000020433	ENSBTAG00000015375	SH2D3C	11	98435180	98467957	-	CODING	-2.22839	0.00028
ENSBTAT00000055956	ENSBTAG00000001137	CLTA	8	60849478	60866939	+	CODING	-1.40316	0.000283

ENSBTAT00000004127	ENSBTAG00000003177	SLC25A33	16	44801770	44831904	-	CODING	-2.25721	0.000283
ENSBTAT00000055279	ENSBTAG00000020630	KCMF1	11	49779932	49811675	-	CODING	-0.50608	0.000289
ENSBTAT00000035337	ENSBTAG00000032954	ATP5E	22	35335190	35335581	+	CODING	-0.97291	0.00029
ENSBTAT00000012812	ENSBTAG00000009711	RPA1	19	23476634	23527009	+	CODING	0.00766	0.0003
ENSBTAT00000024157	ENSBTAG00000018152	MYADM	18	62018419	62024004	+	CODING	-1.12402	0.000302
ENSBTAT00000052276	ENSBTAG00000038379	-	X	39983832	39984337	+	CODING	-1.10371	0.000303
ENSBTAT00000005600	ENSBTAG00000004279	RHOA	22	51277867	51323093	+	CODING	-1.08758	0.000303
ENSBTAT00000015056	ENSBTAG00000011327	OLFML3	3	29506172	29509176	+	CODING	-1.96393	0.000305
ENSBTAT00000010949	ENSBTAG00000008314	SDHB	2	1.36E+08	1.36E+08	+	CODING	-0.98496	0.000305
ENSBTAT00000039850	ENSBTAG00000008310	TMEM9B	15	44215628	44234314	+	CODING	-0.43285	0.000306
ENSBTAT00000053382	ENSBTAG00000005542	EPS15	3	95389092	95528688	+	CODING	-0.31047	0.000307
ENSBTAT00000043792	ENSBTAG00000030942	MRPL3	1	1.4E+08	1.4E+08	+	CODING	-1.48441	0.000312
ENSBTAT00000024703	ENSBTAG00000018562	TMEM159	25	19185676	19201990	+	CODING	-0.29822	0.000312
ENSBTAT00000014805	ENSBTAG00000011150	PFN2	1	1.19E+08	1.19E+08	+	CODING	-0.51464	0.000315
ENSBTAT00000001702	ENSBTAG00000001294	PPP1R15A	18	55925891	55929294	+	CODING	-1.48157	0.000318
ENSBTAT00000007204	ENSBTAG00000005477	LAPTM5	2	1.23E+08	1.23E+08	+	CODING	-1.70085	0.000321
ENSBTAT00000022120	ENSBTAG00000016629	ADCY9	25	3242404	3344129	-	CODING	0.199625	0.000322
ENSBTAT00000023247	ENSBTAG00000017492	PCMTD1	14	22669363	22717576	-	CODING	-0.44322	0.000327
ENSBTAT00000024299	ENSBTAG00000018258	SRSF2	19	55666050	55669890	+	CODING	-1.37504	0.000328
ENSBTAT00000044250	ENSBTAG00000031249	PSMG4	23	50327386	50331704	-	CODING	-1.66578	0.00033
ENSBTAT00000019910	ENSBTAG00000014328	-	18	61608937	61623944	-	CODING	-2.85245	0.000333
ENSBTAT00000055483	ENSBTAG00000039662	RSC1A1	16	53131205	53133055	-	CODING	-0.01566	0.000334
ENSBTAT00000012308	ENSBTAG00000009351	ATXN10	5	1.17E+08	1.17E+08	+	CODING	-0.35107	0.000337
ENSBTAT00000016502	ENSBTAG00000012433	DENND1B	16	78402085	78468912	-	CODING	0.174803	0.00034
ENSBTAT00000017165	ENSBTAG00000012916	-	8	31871390	31872132	+	CODING	-1.95943	0.000344
ENSBTAT00000003959	ENSBTAG00000003043	GNG2	10	44711724	44842190	+	CODING	-2.2584	0.000345
ENSBTAT00000009303	ENSBTAG00000007077	ABHD1	11	72497319	72504279	-	CODING	-3.41161	0.000347
ENSBTAT00000001187	ENSBTAG00000000894	PGK1	X	79282708	79305386	-	CODING	-0.62919	0.000357
ENSBTAT00000024364	ENSBTAG00000018310	SETD3	21	66064391	66125305	-	CODING	-0.20497	0.000357
ENSBTAT00000054074	ENSBTAG00000038652	-	5	74935713	74959662	+	CODING	-1.26846	0.000358
ENSBTAT00000019970	ENSBTAG00000015000	VCP	8	59732258	59746989	-	CODING	-0.56445	0.000359
ENSBTAT00000044356	ENSBTAG00000002953	TXN	8	1.01E+08	1.01E+08	-	CODING	-1.34244	0.000361
ENSBTAT00000015345	ENSBTAG00000011547	KBTBD12	22	60249057	60288275	-	CODING	-0.20583	0.000365
ENSBTAT00000000950	ENSBTAG00000000711	NDRG1	14	9109762	9165926	+	CODING	-1.67968	0.00037
ENSBTAT00000000233	ENSBTAG00000000199	PDP1	14	72678594	72686976	-	CODING	6.712095	0.000373
ENSBTAT00000017759	ENSBTAG00000013347	DMPK	18	53760689	53769611	-	CODING	-0.34986	0.000377
ENSBTAT00000029290	ENSBTAG00000021970	EYA4	9	72661136	72736460	+	CODING	-0.12299	0.000377
ENSBTAT00000054840	ENSBTAG00000038896	RPS26	5	57603811	57606599	-	CODING	-0.25926	0.000377
ENSBTAT00000017386	ENSBTAG00000013076	SNX19	29	37526201	37555971	-	CODING	0.196291	0.000381
ENSBTAT00000004272	ENSBTAG00000003300	MFGE8	21	20889913	20904968	-	CODING	-1.22557	0.000382
ENSBTAT00000020055	ENSBTAG00000015066	LANCL1	2	98617457	98666531	-	CODING	-0.19384	0.000383
ENSBTAT00000034441	ENSBTAG00000009748	MFRP	15	30450602	30455935	-	CODING	-2.94502	0.000392
ENSBTAT00000003377	ENSBTAG00000002610	FKBP3	21	55325021	55335301	-	CODING	-1.06707	0.000395
ENSBTAT00000015581	ENSBTAG00000006982	RAB5C	19	42898002	42903081	-	CODING	-1.31788	0.000396
ENSBTAT00000001785	ENSBTAG00000001356	USP38	17	14889392	14921767	-	CODING	-0.25728	0.000397
ENSBTAT00000004568	ENSBTAG00000003514	HSF4	18	34922999	34927749	+	CODING	-1.99633	0.000401
ENSBTAT00000012120	ENSBTAG00000009200	PAM16	25	3499875	3505894	-	CODING	-1.42293	0.000401
ENSBTAT00000054038	ENSBTAG00000006305	AK1	11	98552024	98561576	-	CODING	-0.96722	0.000407
ENSBTAT00000001374	ENSBTAG00000001036	HIBADH	4	68926598	69034386	+	CODING	-0.42134	0.000408
ENSBTAT00000049347	ENSBTAG00000034885	MGC148992	12	11625778	11641053	+	CODING	-1.63943	0.000409
ENSBTAT00000030224	ENSBTAG00000009210	ZBTB44	29	36988682	37011135	-	CODING	0.01157	0.00041
ENSBTAT00000008076	ENSBTAG00000006138	SEMA3C	4	40140494	40345588	+	CODING	-0.01188	0.000411
ENSBTAT00000025896	ENSBTAG00000019443	CTDNEP1	19	27589896	27596032	-	CODING	-0.4231	0.000414
ENSBTAT00000054334	ENSBTAG00000013300	KCNMA1	28	32827041	33587986	-	CODING	3.194484	0.000414
ENSBTAT00000009702	ENSBTAG00000007378	CLIP4	11	70656465	70733268	-	CODING	0.011973	0.000416
ENSBTAT00000024029	ENSBTAG00000018049	MKNK2	7	22787218	22796782	+	CODING	-0.42351	0.000423
ENSBTAT00000023515	ENSBTAG00000017680	NOS3	4	1.14E+08	1.14E+08	+	CODING	-2.18107	0.000426
ENSBTAT00000014101	ENSBTAG00000010663	ADAM15	3	15593459	15603312	-	CODING	-1.88539	0.000426
ENSBTAT00000007371	ENSBTAG00000005615	CEACAM1	18	51138290	51157109	+	CODING	-1.67791	0.000433
ENSBTAT00000043384	ENSBTAG00000003253	NPPC	2	1.2E+08	1.2E+08	-	CODING	-3.68824	0.000437
ENSBTAT00000008994	ENSBTAG00000006852	ACYP2	11	36683363	36862401	+	CODING	-0.50485	0.000442

ENSBTAT00000031530	ENSBTAG00000023179	TRIB1	14	16353388	16361545	-	CODING	-1.41194	0.000456
ENSBTAT00000011652	ENSBTAG00000008849	SORT1	3	34150876	34214025	+	CODING	-0.02738	0.000458
ENSBTAT00000034695	ENSBTAG00000024909	H3F3A	19	56453856	56455637	+	CODING	-1.25679	0.00047
ENSBTAT00000015875	ENSBTAG00000011963	RPS19	18	51689627	51697161	-	CODING	-1.11826	0.000473
ENSBTAT00000024202	ENSBTAG00000018185	ASB4	4	12597838	12699701	+	CODING	0.13342	0.000477
ENSBTAT00000064026	ENSBTAG00000046996	EPN1	18	62316246	62324320	-	CODING	-0.39651	0.000485
ENSBTAT00000006538	ENSBTAG00000004966	KIF1B	16	44080103	44212899	-	CODING	-0.0372	0.000486
ENSBTAT00000018284	ENSBTAG00000013761	STMN1	2	1.28E+08	1.28E+08	+	CODING	-2.00983	0.00049
ENSBTAT00000006381	ENSBTAG00000004852	PLEKHO1	3	20519726	20528784	-	CODING	-1.82476	0.000497
ENSBTAT00000000566	ENSBTAG00000000442	RBP4	26	14940551	14946750	-	CODING	-1.81477	0.000498
ENSBTAT00000052103	ENSBTAG00000013468	AHNAK	29	41576805	41605018	-	CODING	-0.36184	0.000499
ENSBTAT00000007185	ENSBTAG00000005462	FXR2	19	27932717	27940208	-	CODING	-0.31312	0.000503
ENSBTAT00000054397	ENSBTAG00000009470	CLIC4	2	1.29E+08	1.29E+08	-	CODING	-1.32335	0.000504
ENSBTAT00000017425	ENSBTAG00000013109	STIM1	15	51845122	52049569	-	CODING	-0.33558	0.000505
ENSBTAT00000020872	ENSBTAG00000015723	CRIPT	11	28903112	28914000	+	CODING	-1.36459	0.000513
ENSBTAT00000004179	ENSBTAG00000003220	ACKR1	3	10647427	10648940	-	CODING	-2.29013	0.000515
ENSBTAT00000053592	ENSBTAG00000001762	RFXANK	7	3986329	3991487	-	CODING	-2.3433	0.000518
ENSBTAT00000022761	ENSBTAG00000017125	PABPC4	3	1.07E+08	1.07E+08	+	CODING	-0.46449	0.000522
ENSBTAT00000021848	ENSBTAG00000016429	TMEM205	7	16971974	16974656	-	CODING	-1.87478	0.000524
ENSBTAT00000064257	ENSBTAG00000046184	-	10	43935668	43936221	-	CODING	-1.08728	0.000528
ENSBTAT00000026400	ENSBTAG00000023274	-	5	1.08E+08	1.08E+08	+	CODING	-0.97607	0.000531
ENSBTAT00000055739	ENSBTAG00000011689	LENG8	18	63107489	63117295	+	CODING	-1.61257	0.000532
ENSBTAT00000039717	ENSBTAG00000001027	SERPINH1	15	55514945	55525175	+	CODING	-1.38599	0.000536
ENSBTAT00000010606	ENSBTAG00000008063	PPARA	5	1.17E+08	1.17E+08	+	CODING	0.117327	0.000543
ENSBTAT00000065245	ENSBTAG00000047326	EDA2R	X	89702603	89709755	+	CODING	0.857177	0.000553
ENSBTAT00000061473	ENSBTAG00000044185	SOX6	15	36600420	37082361	+	CODING	0.279278	0.000566
ENSBTAT00000018645	ENSBTAG00000014032	EIF3H	14	49733172	49830429	+	CODING	-1.06872	0.000567
ENSBTAT00000024107	ENSBTAG00000018115	PEBP1	17	59333429	59337958	-	CODING	-0.56737	0.000568
ENSBTAT00000030532	ENSBTAG00000018313	MBNL2	12	78283460	78445751	+	CODING	-0.41174	0.000574
ENSBTAT00000012597	ENSBTAG00000009579	CEP85	2	1.27E+08	1.27E+08	-	CODING	-0.26806	0.000586
ENSBTAT00000005137	ENSBTAG00000003935	RECS1	2	1.07E+08	1.07E+08	-	CODING	-1.22862	0.000594
ENSBTAT00000025469	ENSBTAG00000019133	ZNF326	3	53385189	53419460	-	CODING	-1.69529	0.000595
ENSBTAT00000003999	ENSBTAG00000003072	ACADVL	19	27568181	27573378	+	CODING	-1.07468	0.000597
ENSBTAT00000022292	ENSBTAG00000016762	SLC25A12	2	24678582	24777204	+	CODING	-0.25647	0.000599
ENSBTAT00000008644	ENSBTAG00000006579	P4HA3	15	54418559	54459500	-	CODING	-3.02924	0.000605
ENSBTAT00000033053	ENSBTAG00000024000	ATOH8	11	48954369	48996567	-	CODING	-2.2278	0.000613
ENSBTAT00000023465	ENSBTAG00000017639	RPS6KA3	X	1.3E+08	1.3E+08	+	CODING	-1.39517	0.000616
ENSBTAT00000047359	ENSBTAG00000010526	PPAP2A	20	23658802	23793336	+	CODING	-1.15533	0.000619
ENSBTAT00000020114	ENSBTAG00000015117	GADD45GIP1	7	13721214	13723683	+	CODING	-1.38211	0.000626
ENSBTAT00000017282	ENSBTAG00000012998	NAA30	10	70014347	70031590	+	CODING	-0.02186	0.000628
ENSBTAT00000027808	ENSBTAG00000020873	MAT2A	11	49298517	49305175	-	CODING	-1.26936	0.000643
ENSBTAT00000002852	ENSBTAG00000002203	NDUFS2	3	8309350	8318876	-	CODING	-0.54938	0.000649
ENSBTAT00000017211	ENSBTAG00000012957	SERBP1	3	78243808	78257967	+	CODING	-0.54013	0.000689
ENSBTAT00000027565	ENSBTAG00000020685	-	4	14000446	14001033	+	CODING	-1.56786	0.00069
ENSBTAT00000043062	ENSBTAG00000030518	ECSCR	7	52346079	52354152	-	CODING	-1.6719	0.000695
ENSBTAT00000028159	ENSBTAG00000033008	MYOZ1	28	29780787	29798613	-	CODING	-0.608	0.000696
ENSBTAT00000020002	ENSBTAG00000015025	YAF2	5	38674059	38753871	+	CODING	-5.5112	0.000701
ENSBTAT00000028988	ENSBTAG00000021746	ANXA5	6	3542635	3575330	+	CODING	-1.3032	0.000706
ENSBTAT00000029282	ENSBTAG00000021963	SLAIN2	6	68695298	68741224	+	CODING	-0.25585	0.000715
ENSBTAT00000010169	ENSBTAG00000047834	EFR3A	14	10171320	10250757	-	CODING	-0.22664	0.000718
ENSBTAT00000003990	ENSBTAG00000003067	PSMB7	11	95401692	95458914	-	CODING	-1.1257	0.000719
ENSBTAT00000020267	ENSBTAG00000025564	RPL36A	10	42699004	42700655	-	CODING	-1.18888	0.000721
ENSBTAT00000052919	ENSBTAG00000018732	HSPA12B	13	51901885	51918849	-	CODING	-1.77728	0.000731
ENSBTAT00000018406	ENSBTAG00000013858	C25H16ORF7	25	7931600	7955194	+	CODING	-0.38671	0.000741
ENSBTAT00000025873	ENSBTAG00000019422	-	11	22366049	22367102	-	CODING	0.108401	0.000741
ENSBTAT00000063874	ENSBTAG00000045550	TSPAN6	X	50904005	50912844	+	CODING	-1.65121	0.000752
ENSBTAT00000009341	ENSBTAG00000007101	F3	3	49110156	49121034	+	CODING	-2.3653	0.000759
ENSBTAT00000037230	ENSBTAG00000026249	-	5	35174130	35174846	-	CODING	-3.44416	0.00078
ENSBTAT00000011920	ENSBTAG00000009047	YPEL3	25	26451072	26454431	+	CODING	-1.11214	0.000785
ENSBTAT00000028731	ENSBTAG00000021565	PRSS2	4	1.07E+08	1.07E+08	+	CODING	-4.88379	0.000786
ENSBTAT00000028712	ENSBTAG00000021549	PHPT1	11	1.06E+08	1.06E+08	-	CODING	-1.09837	0.000791

ENSBTAT00000019259	ENSBTAG00000014482	FAF1	3	95937279	96434760 +		CODING	-0.02708	0.0008
ENSBTAT00000015750	ENSBTAG00000011866	PCBD1	28	27226795	27231724 -		CODING	-1.96085	0.000802
ENSBTAT00000000859	ENSBTAG00000000656	NFATC1	24	1018253	1099817 -		CODING	0.191689	0.000803
ENSBTAT00000048797	ENSBTAG00000034449	GNG11	3	5454661	5455256 +		CODING	-1.4213	0.00082
ENSBTAT00000060940	ENSBTAG00000015887	FOXJ3	3	1.04E+08	1.05E+08 +		CODING	-0.23336	0.000821
ENSBTAT00000044482	ENSBTAG00000040367	-	2	92993922	92995706 -		CODING	5.410148	0.000829
ENSBTAT00000005777	ENSBTAG00000004403	CD320	7	18183237	18187953 -		CODING	-2.08502	0.000835
ENSBTAT00000027702	ENSBTAG00000020787	PIK3R4	1	1.53E+08	1.53E+08 +		CODING	-0.07557	0.000836
ENSBTAT00000001315	ENSBTAG00000000993	N4BP2L1	12	28612076	28634685 +		CODING	-1.84219	0.000837
ENSBTAT00000007327	ENSBTAG00000005577	RNASEH2C	29	44552309	44553484 -		CODING	-1.38367	0.000839
ENSBTAT00000019613	ENSBTAG00000014737	VEGFC	27	6999636	7084866 -		CODING	-2.18524	0.000856
ENSBTAT00000045971	ENSBTAG00000032402	U1	3	22861821	22861984 -		CODING	-4.86652	0.000857
ENSBTAT00000022307	ENSBTAG00000016771	PLK2	20	20854607	20860584 +		CODING	-1.72166	0.000874
ENSBTAT00000013264	ENSBTAG00000030529	FAM53C	7	51349485	51358857 +		CODING	-0.20214	0.000877
ENSBTAT00000019553	ENSBTAG00000014691	ARFGEF1	14	33343880	33480413 -		CODING	-0.15551	0.000887
ENSBTAT00000018109	ENSBTAG00000013623	TIMM13	7	22448900	22450073 +		CODING	-1.30597	0.00089
ENSBTAT00000005009	ENSBTAG00000003840	GUCY1B1	17	44506802	44569575 -		CODING	-1.77711	0.0009
ENSBTAT00000010372	ENSBTAG00000007884	SREBF1	19	35234637	35250672 +		CODING	-0.26066	0.000916
ENSBTAT00000032006	ENSBTAG00000023471	RPL36	5	28525103	28525496 +		CODING	-1.09856	0.000919
ENSBTAT00000055155	ENSBTAG00000040380	-	15	51667478	51669427 +		CODING	-0.10883	0.000924
ENSBTAT00000029119	ENSBTAG00000021845	STRN3	21	41964003	42070978 -		CODING	-0.29737	0.000932
ENSBTAT00000048357	ENSBTAG00000014262	BZW2	4	25097884	25161432 +		CODING	-0.51482	0.000934
ENSBTAT00000003072	ENSBTAG00000002377	PSMB2	3	1.11E+08	1.11E+08 +		CODING	-1.15914	0.000937
ENSBTAT00000026525	ENSBTAG00000019911	COA3	19	43468381	43469490 -		CODING	-1.18811	0.000937
ENSBTAT00000025806	ENSBTAG00000019369	TSPAN4	29	50560713	50570675 -		CODING	-1.7794	0.000939
ENSBTAT00000027461	ENSBTAG00000020608	ATG9A	2	1.08E+08	1.08E+08 -		CODING	-0.27488	0.000955
ENSBTAT00000030962	ENSBTAG00000022808	CACUL1	26	39292197	39356979 -		CODING	0.132965	0.000956
ENSBTAT00000004810	ENSBTAG00000003692	UCP2	15	54196170	54202633 -		CODING	-1.61632	0.000964
ENSBTAT00000025640	ENSBTAG00000013982	UACA	10	17516709	17559258 -		CODING	-0.33279	0.000972
ENSBTAT00000066041	ENSBTAG00000046533	RAB31	24	42256081	42305185 +		CODING	0.012108	0.000976
ENSBTAT00000009385	ENSBTAG00000007131	GADL1	22	5258463	5452369 -		CODING	-0.30634	0.000976
ENSBTAT00000000579	ENSBTAG00000000455	CREBZF	29	9952633	9958039 +		CODING	-1.60133	0.000981
ENSBTAT00000012050	ENSBTAG00000009145	SLC7A4	17	74366170	74369086 -		CODING	-1.81487	0.000985
ENSBTAT00000061429	ENSBTAG00000044046	SGCD	7	69585066	70027839 +		CODING	-0.25514	0.001002
ENSBTAT00000011762	ENSBTAG00000008935	PFDN5	5	26886091	26890186 -		CODING	-1.19882	0.001004
ENSBTAT00000001246	ENSBTAG00000000941	PLEKHB2	2	1850107	1899289 +		CODING	-0.3961	0.001014
ENSBTAT00000025941	ENSBTAG00000019472	GR-A	7	56235966	56350171 -		CODING	-0.07916	0.001017
ENSBTAT00000012144	ENSBTAG00000009214	ETS2	1	1.53E+08	1.53E+08 +		CODING	-1.30278	0.001021
ENSBTAT00000061332	ENSBTAG00000000024	DYM	24	49193606	49590504 -		CODING	-0.27021	0.001021
ENSBTAT00000056799	ENSBTAG00000038889	RILP	19	23320981	23323996 -		CODING	-1.72823	0.001032
ENSBTAT00000007436	ENSBTAG00000005660	PSMD2	1	83491699	83501041 -		CODING	-0.44935	0.001033
ENSBTAT00000011215	ENSBTAG00000023416	PPP2R3A	1	1.34E+08	1.34E+08 -		CODING	-0.12005	0.001047
ENSBTAT00000006992	ENSBTAG00000005316	GDI2	13	43302440	43331090 +		CODING	-0.51507	0.001056
ENSBTAT00000035230	ENSBTAG00000025161	AGPAT2	11	1.04E+08	1.04E+08 -		CODING	-0.45514	0.001056
ENSBTAT00000026769	ENSBTAG00000020093	CUL4A	12	90562966	90589009 +		CODING	-0.46812	0.001062
ENSBTAT00000033787	ENSBTAG00000001908	DLD	4	49230967	49257713 +		CODING	-0.53533	0.001069
ENSBTAT00000019499	ENSBTAG00000014643	EEF1D	14	2317971	2326718 +		CODING	-1.20407	0.001074
ENSBTAT00000025590	ENSBTAG00000019217	KIF13A	23	39404303	39501741 +		CODING	0.160639	0.001076
ENSBTAT00000063284	ENSBTAG00000048122	CFD	7	45030310	45032845 +		CODING	-1.8984	0.001084
ENSBTAT00000003595	ENSBTAG00000002770	TCN2	17	71711883	71727449 +		CODING	-1.43505	0.001094
ENSBTAT00000016620	ENSBTAG00000012519	XDH	11	14176298	14281717 -		CODING	-1.50006	0.001095
ENSBTAT00000004501	ENSBTAG00000038865	TCEA3	2	1.3E+08	1.3E+08 +		CODING	-0.51658	0.001096
ENSBTAT00000026118	ENSBTAG00000019603	LDHB	5	88962679	88981219 +		CODING	-1.09873	0.001097
ENSBTAT00000003926	ENSBTAG00000038409	MBD3	7	45577377	45579729 -		CODING	-0.27384	0.001101
ENSBTAT00000036447	ENSBTAG00000025778	EMC9	10	20844420	20846694 +		CODING	-1.33816	0.001105
ENSBTAT00000051159	ENSBTAG00000036659	U2	29	41846574	41846764 -		CODING	-2.47793	0.001123
ENSBTAT00000066124	ENSBTAG00000045828	PTBP1	7	45016275	45026949 +		CODING	-1.59156	0.001127
ENSBTAT00000006033	ENSBTAG00000004599	TAGLN3	1	57178554	57192433 +		CODING	-2.08372	0.00113
ENSBTAT00000018741	ENSBTAG00000014093	IDH2	21	21903847	21920153 -		CODING	-1.05948	0.001132
ENSBTAT00000007481	ENSBTAG00000005694	TMED10	10	86770121	86802293 -		CODING	-0.3728	0.001136
ENSBTAT00000044399	ENSBTAG00000013436	HAUS5	18	46528185	46538466 +		CODING	-2.33273	0.001143

ENSBTAT00000003766	ENSBTAG00000002898	UNC45B	19	15233409	15265756	-	CODING	-0.44275	0.001147
ENSBTAT00000064202	ENSBTAG00000011121	CLCN4	X	1.43E+08	1.43E+08	-	CODING	0.515979	0.001148
ENSBTAT00000052061	ENSBTAG00000038462	TMEM182	11	7449519	7492859	+	CODING	-0.44948	0.00115
ENSBTAT00000056239	ENSBTAG00000037899	DLK	21	67274800	67283089	+	CODING	-2.9239	0.001156
ENSBTAT00000020263	ENSBTAG00000015230	PLA2G12A	6	16828642	16843001	+	CODING	-0.16478	0.00116
ENSBTAT00000012106	ENSBTAG00000009187	DNAJC28	1	1308527	1310072	+	CODING	-0.10267	0.001175
ENSBTAT00000021123	ENSBTAG00000015892	NDUFB4	1	65922482	65928617	+	CODING	-1.06179	0.001192
ENSBTAT00000014035	ENSBTAG00000010617	KRTCAP2	3	15498847	15501486	+	CODING	-1.2865	0.001198
ENSBTAT00000048277	ENSBTAG00000034033	LHFP	12	22819645	23026440	+	CODING	-1.65886	0.0012
ENSBTAT00000010553	ENSBTAG00000008025	UBE3C	4	1.19E+08	1.19E+08	+	CODING	-0.21418	0.001221
ENSBTAT00000005987	ENSBTAG00000004558	MGC165862	10	65243195	65246838	-	CODING	-2.65033	0.001242
ENSBTAT00000048956	ENSBTAG00000034560	-	8	11064112	11064777	-	CODING	-0.14034	0.001245
ENSBTAT00000014152	ENSBTAG00000010696	DVL1	16	52431328	52440427	+	CODING	-0.25449	0.001249
ENSBTAT00000007165	ENSBTAG00000005446	POLR2J	25	35091300	35095676	+	CODING	-1.2369	0.001267
ENSBTAT00000001086	ENSBTAG00000000820	GNG11	4	11074737	11079756	+	CODING	-1.53582	0.001267
ENSBTAT00000025165	ENSBTAG00000018909	-	4	67767893	67899241	-	CODING	-1.7743	0.001269
ENSBTAT00000008319	ENSBTAG00000006342	ATPIF1	2	1.26E+08	1.26E+08	-	CODING	-0.96107	0.001285
ENSBTAT00000019328	ENSBTAG00000030424	CLEC1A	5	1E+08	1E+08	+	CODING	-2.09595	0.001299
ENSBTAT00000009760	ENSBTAG00000007421	CDH5	18	34260148	34274741	+	CODING	-1.40962	0.001301
ENSBTAT00000011383	ENSBTAG00000008632	-	25	26389490	26390167	+	CODING	-1.71529	0.001308
ENSBTAT00000017857	ENSBTAG00000013420	FSD1L	8	96964125	97006477	+	CODING	0.371864	0.001318
ENSBTAT00000004215	ENSBTAG00000019017	IFITM2	11	1.07E+08	1.07E+08	-	CODING	-2.16777	0.001321
ENSBTAT00000017907	ENSBTAG00000013464	CEP97	1	46431094	46452391	+	CODING	-0.00709	0.001337
ENSBTAT00000035307	ENSBTAG00000019964	GAS6	12	90821803	90850163	-	CODING	-1.54877	0.001349
ENSBTAT00000026869	ENSBTAG00000020174	HBS1L	9	74011379	74096501	-	CODING	-0.4301	0.001357
ENSBTAT00000064442	ENSBTAG00000045582	ARPC5	16	66150415	66159807	-	CODING	-1.51491	0.00137
ENSBTAT00000018675	ENSBTAG00000014051	ANGPT1	14	59137214	59436333	+	CODING	1.099704	0.001372
ENSBTAT00000000705	ENSBTAG00000000542	CCDC104	11	38066011	38095813	+	CODING	-1.40345	0.001388
ENSBTAT00000011330	ENSBTAG00000008591	CAMTA2	19	27054328	27067739	+	CODING	-0.23694	0.001391
ENSBTAT00000044652	ENSBTAG00000016598	DYNC1H1	21	68507134	68568024	+	CODING	-0.19012	0.001394
ENSBTAT00000007028	ENSBTAG00000005345	ARPC3	17	56573543	56584354	-	CODING	-1.30095	0.001407
ENSBTAT00000066191	ENSBTAG00000001546	MGAT1	7	41337278	41349616	-	CODING	-1.65958	0.001409
ENSBTAT00000002922	ENSBTAG00000002267	ANKRD40	19	36655574	36675085	+	CODING	-0.11038	0.00141
ENSBTAT00000007921	ENSBTAG00000006027	USP34	11	59791217	59987854	-	CODING	-0.20236	0.001416
ENSBTAT00000009699	ENSBTAG00000007375	MIF	17	73273379	73274171	+	CODING	-1.3333	0.001419
ENSBTAT00000015290	ENSBTAG00000011505	RABEP1	19	26759902	26870523	-	CODING	-0.28597	0.001422
ENSBTAT00000020501	ENSBTAG00000015426	PDLIM4	7	23443528	23457942	-	CODING	-2.65827	0.001433
ENSBTAT00000013354	ENSBTAG00000010123	APOE	18	53040105	53042792	+	CODING	-1.57895	0.001436
ENSBTAT00000003431	ENSBTAG00000002648	RPS18	23	7388703	7393361	+	CODING	-0.96753	0.001455
ENSBTAT00000023499	ENSBTAG00000017656	SIAT6	3	1.03E+08	1.03E+08	-	CODING	-0.48189	0.001499
ENSBTAT00000027260	ENSBTAG00000020456	AGPAT3	1	1.47E+08	1.47E+08	+	CODING	-0.11424	0.001511
ENSBTAT00000061204	ENSBTAG00000012307	DTNA	24	22445691	22767026	-	CODING	-0.15029	0.001514
ENSBTAT00000015285	ENSBTAG00000011500	CASQ2	3	27658862	27729416	+	CODING	-0.00306	0.00153
ENSBTAT00000007226	ENSBTAG00000005497	RPRD1A	24	21361884	21427671	+	CODING	0.090574	0.001534
ENSBTAT00000059593	ENSBTAG00000042601	7SK	9	39583059	39583372	+	CODING	-4.74567	0.001539
ENSBTAT00000027864	ENSBTAG00000000874	CAST	7	98445837	98581253	+	CODING	0.181266	0.00155
ENSBTAT00000002878	ENSBTAG00000002226	FBXO31	18	12974880	13009289	-	CODING	-0.27032	0.001566
ENSBTAT00000062920	ENSBTAG00000045889	-	3	28737677	28745359	-	CODING	0.142565	0.001575
ENSBTAT00000018391	ENSBTAG00000013834	-	7	18960639	18964880	+	CODING	-1.26555	0.001582
ENSBTAT00000011077	ENSBTAG00000008419	PDE2A	15	52913333	52984107	-	CODING	-1.9039	0.00159
ENSBTAT00000006650	ENSBTAG00000005040	SMEK1	21	56800878	56835145	-	CODING	-1.43662	0.001595
ENSBTAT00000021369	ENSBTAG00000016057	CSRP1	16	49332770	49353517	-	CODING	-1.28173	0.001598
ENSBTAT00000012636	ENSBTAG00000009602	TMEM120B	17	55772992	55815637	-	CODING	-1.69518	0.001602
ENSBTAT00000044005	ENSBTAG00000031088	-	3	61240417	61241633	+	CODING	-0.4136	0.001606
ENSBTAT00000049743	ENSBTAG00000002487	UBE3A	21	2346814	2410193	-	CODING	-0.46238	0.001622
ENSBTAT00000002033	ENSBTAG00000001553	HNRNPA1	5	25939737	25944246	-	CODING	-1.14291	0.001627
ENSBTAT00000014162	ENSBTAG00000010701	NACA	5	57063071	57074070	+	CODING	-1.16226	0.001639
ENSBTAT00000002488	ENSBTAG00000011571	ACIN1	10	21626432	21637359	+	CODING	-2.11685	0.001642
ENSBTAT00000001140	ENSBTAG00000000855	NXN	19	22457712	22621493	+	CODING	-0.23828	0.001655
ENSBTAT00000003427	ENSBTAG00000002643	VPS52	23	7370938	7388536	-	CODING	-1.80493	0.001657
ENSBTAT00000023496	ENSBTAG00000017666	ABCC5	1	83765087	83817531	+	CODING	-1.55711	0.00166

ENSBTAT00000009044	ENSBTAG00000006883	EIF4B	5	27091609	27115819	-	CODING	-0.55376	0.00168
ENSBTAT00000014026	ENSBTAG00000010610	DDIT4L	6	25889906	25894862	+	CODING	-0.57572	0.001688
ENSBTAT00000000802	ENSBTAG00000000609	-	1	54654216	54654795	-	CODING	-1.10829	0.001701
ENSBTAT00000046579	ENSBTAG00000032812	-	14	45039060	45039371	-	CODING	0.306255	0.001711
ENSBTAT00000044296	ENSBTAG00000017616	ADSSL1	21	70845971	70861972	+	CODING	-0.47808	0.001716
ENSBTAT00000032213	ENSBTAG00000012881	EIF4G1	1	83466847	83484338	-	CODING	-0.46016	0.001721
ENSBTAT00000039169	ENSBTAG00000027320	KCNB1	13	78194165	78304515	-	CODING	0.187685	0.001734
ENSBTAT00000015224	ENSBTAG00000011456	NT5C3L	19	42657994	42665703	-	CODING	-2.04043	0.001749
ENSBTAT00000026568	ENSBTAG00000019944	METAP2	5	25255494	25286217	+	CODING	-0.42375	0.001758
ENSBTAT00000012028	ENSBTAG00000009127	TSPYL4	9	34889676	34893768	+	CODING	-0.15668	0.00176
ENSBTAT00000008580	ENSBTAG00000006533	NSFL1C	13	60244889	60265244	+	CODING	-1.20941	0.001774
ENSBTAT00000052238	ENSBTAG00000008285	OXR1	14	60000394	60075278	-	CODING	-0.12783	0.001774
ENSBTAT00000028860	ENSBTAG00000021657	CACNG6	18	62106778	62121184	+	CODING	-0.23802	0.001776
ENSBTAT00000006646	ENSBTAG00000005039	ARAF	X	91180863	91191308	+	CODING	-0.38416	0.001778
ENSBTAT00000019747	ENSBTAG00000014827	UBQLN1	8	78223814	78279585	-	CODING	0.790226	0.001787
ENSBTAT00000007014	ENSBTAG00000005333	MB	5	74170471	74181260	-	CODING	-0.83269	0.00179
ENSBTAT00000020406	ENSBTAG00000015350	PLIN	21	21502826	21516686	-	CODING	-2.03462	0.001792
ENSBTAT00000021336	ENSBTAG00000016033	HOXD9	2	20824235	20825910	-	CODING	0.031448	0.001797
ENSBTAT00000064455	ENSBTAG00000046467	PTP4A3	14	3578346	3586015	-	CODING	-0.50071	0.001802
ENSBTAT00000002235	ENSBTAG00000001706	SLC9A2	11	7324936	7414636	+	CODING	0.20585	0.001814
ENSBTAT00000007398	ENSBTAG00000005627	PAFAH1B2	15	28260081	28286877	+	CODING	-0.28266	0.00182
ENSBTAT00000009081	ENSBTAG00000006919	SMAD4	24	50993949	51045661	+	CODING	-0.32599	0.001824
ENSBTAT00000044459	ENSBTAG00000008013	LRRC2	22	53448776	53486047	+	CODING	-0.4818	0.001826
ENSBTAT00000000086	ENSBTAG00000000078	GLIPR2	8	60797959	60816816	+	CODING	-1.71584	0.001832
ENSBTAT00000043544	ENSBTAG00000030805	SHISA4	16	49618802	49620917	+	CODING	-0.99404	0.001835
ENSBTAT00000024049	ENSBTAG00000018063	SPATA2	13	78712288	78722796	-	CODING	0.08339	0.001851
ENSBTAT00000020200	ENSBTAG00000015181	EPM2A	9	84028748	84136430	-	CODING	-0.28693	0.001852
ENSBTAT00000027614	ENSBTAG00000020721	MRPL55	7	2905629	2909407	+	CODING	-1.21011	0.001856
ENSBTAT00000020880	ENSBTAG00000015731	TXNL1	24	56404067	56446678	-	CODING	-1.03417	0.001861
ENSBTAT00000003343	ENSBTAG00000002580	MYH14	18	56874763	56947774	+	CODING	0.014085	0.00187
ENSBTAT00000039403	ENSBTAG00000008520	NFIC	7	21676708	21748545	-	CODING	-0.16848	0.001879
ENSBTAT00000001536	ENSBTAG00000001154	DGAT2	15	55940757	55973229	+	CODING	-1.57617	0.001889
ENSBTAT00000011670	ENSBTAG00000008864	MD2	14	39369425	39402263	+	CODING	-2.51668	0.00192
ENSBTAT00000063631	ENSBTAG00000040215	EIF4G3	2	1.32E+08	1.32E+08	+	CODING	-0.19562	0.001925
ENSBTAT00000036839	ENSBTAG00000010012	BTF3	20	8038617	8045160	-	CODING	-0.9853	0.001933
ENSBTAT00000029056	ENSBTAG00000021798	ERBB2	19	40722051	40745987	+	CODING	-0.06697	0.001935
ENSBTAT00000018083	ENSBTAG00000013598	RSPO2	14	58537071	58653100	+	CODING	-0.08404	0.00195
ENSBTAT00000006451	ENSBTAG00000004902	INTS8	14	71820446	71848531	-	CODING	-0.16141	0.001962
ENSBTAT00000025384	ENSBTAG00000019067	TMC8	19	54633144	54642106	-	CODING	-3.22823	0.001972
ENSBTAT00000060961	ENSBTAG00000008301	WNK2	8	85984692	86107090	+	CODING	0.496407	0.001975
ENSBTAT00000013468	ENSBTAG00000010208	ZER1	11	99255811	99284276	-	CODING	-0.03484	0.001979
ENSBTAT00000023206	ENSBTAG00000017448	EFEMP1	11	38338744	38408288	-	CODING	-1.66599	0.002005
ENSBTAT00000003307	ENSBTAG00000002551	KCNJ8	5	88852393	88860007	+	CODING	-1.77421	0.002011
ENSBTAT00000009684	ENSBTAG00000007363	LSM3	22	58693674	58701742	-	CODING	-1.41666	0.002016
ENSBTAT00000017489	ENSBTAG00000013153	NF2	17	70895108	70966968	+	CODING	-0.10705	0.002017
ENSBTAT00000010577	ENSBTAG00000008040	SPG20	12	25199123	25229866	+	CODING	-0.35209	0.002022
ENSBTAT00000039955	ENSBTAG00000027722	IPO7	15	43781463	43825036	-	CODING	-0.43239	0.002022
ENSBTAT00000028757	ENSBTAG00000021580	HNRNPA3	2	19700523	19705981	-	CODING	-1.18427	0.002026
ENSBTAT00000031470	ENSBTAG00000005718	PLIN2	8	25129168	25142901	+	CODING	-1.16208	0.002028
ENSBTAT00000008884	ENSBTAG00000006755	SMAP	15	36224848	36251371	-	CODING	-0.37407	0.002032
ENSBTAT00000063462	ENSBTAG00000046509	FAM46C	3	25703050	25705109	-	CODING	0.532313	0.002039
ENSBTAT00000050440	ENSBTAG00000016062	C14orf28	21	55153786	55162538	+	CODING	0.488574	0.002042
ENSBTAT00000004772	ENSBTAG00000031564	GNL3L	X	97432890	97461194	+	CODING	-1.40827	0.002053
ENSBTAT00000024122	ENSBTAG00000018123	FBLN5	21	57153110	57246389	-	CODING	-1.53893	0.002081
ENSBTAT00000047947	ENSBTAG00000001212	SSR2	3	14808605	14815178	+	CODING	-1.19821	0.002081
ENSBTAT00000036100	ENSBTAG00000000527	ZFAND3	23	11712802	12040961	+	CODING	-0.38785	0.002082
ENSBTAT00000001709	ENSBTAG00000001298	STAMBPL1	26	10597573	10650140	+	CODING	-1.84173	0.002085
ENSBTAT00000066011	ENSBTAG00000000605	ATP5J	1	10069815	10076995	+	CODING	-0.96657	0.00209
ENSBTAT00000046362	ENSBTAG00000002608	42980	3	1.21E+08	1.21E+08	+	CODING	-1.41379	0.002092
ENSBTAT00000002944	ENSBTAG00000002278	FBN1	10	61877808	62142171	+	CODING	-0.00242	0.002102
ENSBTAT00000024011	ENSBTAG00000018037	PSKH1	18	35521165	35534184	+	CODING	-0.09086	0.002107

ENSBTAT00000043503	ENSBTAG00000005275	PKIG	13	73669767	73749992	+	CODING	-1.75453	0.002119
ENSBTAT00000004836	ENSBTAG00000003711	EPAS1	11	28576347	28668899	+	CODING	-1.18452	0.002121
ENSBTAT00000025063	ENSBTAG00000018828	ATN1	5	1.04E+08	1.04E+08	-	CODING	-0.19174	0.002124
ENSBTAT00000065901	ENSBTAG00000047491	CACNA1S	16	81472038	81527931	-	CODING	-0.4255	0.002125
ENSBTAT00000010682	ENSBTAG00000008122	GNG5	3	59783839	59791977	+	CODING	-1.10193	0.002131
ENSBTAT00000066246	ENSBTAG00000046218	TIEG2	11	87525614	87536326	-	CODING	-0.31732	0.002138
ENSBTAT00000017147	ENSBTAG00000012899	IFNGR2	1	1376827	1397948	-	CODING	-0.24798	0.00214
ENSBTAT00000028645	ENSBTAG00000021491	NDUFV3	1	1.45E+08	1.45E+08	+	CODING	-0.49217	0.002151
ENSBTAT00000002265	ENSBTAG00000001729	DUSP10	16	25895751	25936856	-	CODING	-2.06975	0.002172
ENSBTAT00000014799	ENSBTAG00000011143	KPNA1	1	67476527	67545097	-	CODING	-0.42863	0.002193
ENSBTAT00000004760	ENSBTAG00000003652	TMEM128	6	1.07E+08	1.07E+08	+	CODING	-1.60062	0.00223
ENSBTAT00000050399	ENSBTAG00000035907	DDX3X	X	1.08E+08	1.08E+08	-	CODING	-0.53177	0.002232
ENSBTAT00000022279	ENSBTAG00000016754	KLHL38	14	17820791	17831087	+	CODING	-0.22383	0.002237
ENSBTAT00000014091	ENSBTAG00000010661	DPYSL3	7	60588659	60703487	-	CODING	0.02931	0.002241
ENSBTAT00000017600	ENSBTAG00000013227	SNAI2	14	21577309	21580910	-	CODING	-2.13116	0.002267
ENSBTAT00000005523	ENSBTAG00000004215	RARRES2	4	1.14E+08	1.14E+08	-	CODING	-1.66236	0.002276
ENSBTAT00000040247	ENSBTAG00000016221	AMOT	X	68598671	68657930	-	CODING	-0.11398	0.002285
ENSBTAT00000039551	ENSBTAG00000008577	GRSF1	6	87922395	87940543	-	CODING	-0.13719	0.002293
ENSBTAT00000026534	ENSBTAG00000019915	GSN	8	1.13E+08	1.13E+08	+	CODING	-1.02683	0.002304
ENSBTAT00000024105	ENSBTAG00000018112	TBL1X	X	1.44E+08	1.44E+08	-	CODING	-0.16022	0.002323
ENSBTAT00000015459	ENSBTAG00000011639	STK11	7	45282199	45289554	-	CODING	-0.2064	0.002328
ENSBTAT00000022350	ENSBTAG00000016801	RXRG	3	3576688	3636911	+	CODING	0.218517	0.002369
ENSBTAT00000009950	ENSBTAG00000007559	URI1	18	40683382	40745558	+	CODING	-0.19835	0.002416
ENSBTAT00000008386	ENSBTAG00000006396	GPI	18	44979578	45007642	+	CODING	-0.62117	0.002436
ENSBTAT00000000282	ENSBTAG00000000231	BMPR-IA	28	41817915	41875990	+	CODING	-0.18862	0.002437
ENSBTAT00000011240	ENSBTAG00000008527	SRSF6	13	72804660	72810323	+	CODING	-1.3035	0.002445
ENSBTAT00000054111	ENSBTAG00000039130	-	7	14670708	14672994	-	CODING	-1.21753	0.002452
ENSBTAT00000014847	ENSBTAG00000011180	ACTR8	22	47591904	47607214	+	CODING	0.106582	0.002454
ENSBTAT00000002200	ENSBTAG00000001687	STC1	8	71854905	71868618	-	CODING	-2.02957	0.002474
ENSBTAT00000010856	ENSBTAG00000008251	SNRPN	21	2566	25936	-	CODING	-0.36795	0.002478
ENSBTAT00000011408	ENSBTAG00000008652	DUSP27	3	1656685	1723930	-	CODING	-0.4582	0.002484
ENSBTAT00000019706	ENSBTAG00000014807	DPP8	10	12321152	12369941	-	CODING	-0.10199	0.002493
ENSBTAT00000013075	ENSBTAG00000009906	DDX1	11	82824017	82855964	-	CODING	-0.35732	0.002522
ENSBTAT00000019637	ENSBTAG00000014759	-	5	95536891	95549925	+	CODING	-0.479	0.00254
ENSBTAT00000008734	ENSBTAG00000006646	HRMT1L2	18	56534833	56545053	+	CODING	-1.18243	0.002559
ENSBTAT00000023727	ENSBTAG00000008520	NFIC	7	21676708	21748618	-	CODING	-0.31187	0.002568
ENSBTAT00000007761	ENSBTAG00000005907	NDUFB6	8	11372938	11389199	+	CODING	-1.06261	0.002569
ENSBTAT00000013623	ENSBTAG00000010312	MAPK1	17	74016493	74035118	+	CODING	-0.36881	0.002572
ENSBTAT00000013950	ENSBTAG00000010549	IFRD1	4	55872976	55896662	-	CODING	-1.24114	0.002579
ENSBTAT00000011559	ENSBTAG00000021039	-	7	18289395	18329947	+	CODING	-1.21839	0.002594
ENSBTAT00000031029	ENSBTAG00000011250	AFG3L2	24	43252927	43271906	-	CODING	-0.33804	0.002599
ENSBTAT00000047653	ENSBTAG00000008832	CCL1	19	16110980	16114069	+	CODING	-2.52186	0.002604
ENSBTAT00000042613	ENSBTAG00000019105	NPLOC4	19	51781191	51829647	+	CODING	-0.31397	0.002617
ENSBTAT00000053426	ENSBTAG00000003994	IGFBP3	4	76705105	76712709	+	CODING	-1.40322	0.002643
ENSBTAT00000006115	ENSBTAG00000004659	COQ10B	2	86410860	86427846	+	CODING	-1.53003	0.002655
ENSBTAT00000042695	ENSBTAG00000012066	PECAM1	19	49175892	49238414	-	CODING	-1.27674	0.002657
ENSBTAT00000038295	ENSBTAG00000008032	-	4	1.16E+08	1.16E+08	+	CODING	-0.36073	0.002676
ENSBTAT00000065287	ENSBTAG00000048156	-	21	19391574	19391762	-	CODING	-0.17192	0.00271
ENSBTAT00000023725	ENSBTAG00000017846	F11R	3	8483556	8508122	+	CODING	-1.47425	0.00275
ENSBTAT00000004009	ENSBTAG00000003081	RWDD4A	27	13404609	13412552	-	CODING	-0.38514	0.00277
ENSBTAT00000002684	ENSBTAG00000002076	STAU2	14	38927061	39070664	-	CODING	-0.3196	0.00278
ENSBTAT00000029502	ENSBTAG00000013152	NIPSNAP1	17	70869039	70884644	-	CODING	0.350426	0.002789
ENSBTAT00000021719	ENSBTAG00000016328	SFPQ	3	1.11E+08	1.11E+08	+	CODING	-1.22407	0.002804
ENSBTAT00000026183	ENSBTAG00000019648	GNB2L1	7	41761805	41766598	-	CODING	-0.93298	0.002844
ENSBTAT00000010270	ENSBTAG00000007807	-	10	76992202	76994112	+	CODING	0.005602	0.002864
ENSBTAT00000015763	ENSBTAG00000024091	MALL	11	1747909	1780103	+	CODING	-2.00006	0.002875
ENSBTAT00000027899	ENSBTAG00000020947	RNF103	11	48149255	48171946	+	CODING	-0.27487	0.002877
ENSBTAT00000061414	ENSBTAG00000007635	PLCL1	2	86718341	87086748	+	CODING	0.146981	0.002879
ENSBTAT00000018099	ENSBTAG00000013614	TMEM38A	7	6285842	6312400	-	CODING	-0.5504	0.002888
ENSBTAT00000003884	ENSBTAG00000002983	NT5C1A	3	1.07E+08	1.07E+08	+	CODING	-0.0095	0.002889
ENSBTAT00000002846	ENSBTAG00000027930	-	2	1.22E+08	1.22E+08	-	CODING	-1.45748	0.002916

ENSBTAT00000018509	ENSBTAG00000013931	FAM96B	18	34754940	34756799	-	CODING	-1.18845	0.002966
ENSBTAT00000013956	ENSBTAG00000010555	LSMEM1	4	55858087	55868557	-	CODING	-0.29875	0.002968
ENSBTAT00000065362	ENSBTAG00000048273	-	6	7145414	7150482	+	CODING	-1.25257	0.002991
ENSBTAT00000043424	ENSBTAG00000007689	LPIN1	11	86051195	86128538	-	CODING	-1.381	0.002993
ENSBTAT00000002422	ENSBTAG00000001858	-	5	70910366	70911285	+	CODING	-0.40456	0.002994
ENSBTAT00000053555	ENSBTAG00000018922	TRMT10B	8	62328443	62339566	+	CODING	-1.89104	0.003054
ENSBTAT00000025510	ENSBTAG00000019167	INPPL1	15	52609012	52623088	+	CODING	-0.24348	0.003059
ENSBTAT00000024807	ENSBTAG00000018644	PDZRN3	22	28238772	28506578	+	CODING	-0.40596	0.003097
ENSBTAT00000022098	ENSBTAG00000016612	NEK9	10	86727710	86762452	-	CODING	-0.15396	0.003098
ENSBTAT00000013376	ENSBTAG00000010135	TMEM189	13	78882751	78907460	-	CODING	-0.29393	0.003136
ENSBTAT00000057115	ENSBTAG00000019852	PDHA1	X	1.31E+08	1.31E+08	-	CODING	-0.58605	0.003138
ENSBTAT00000013889	ENSBTAG00000010508	BLVRB	18	50056571	50073609	-	CODING	-1.18193	0.003156
ENSBTAT00000063472	ENSBTAG00000032427	FHOD1	18	34977246	34993385	-	CODING	-0.33442	0.003157
ENSBTAT00000016071	ENSBTAG00000012120	TIPARP	1	1.12E+08	1.12E+08	-	CODING	-1.54525	0.003191
ENSBTAT00000005549	ENSBTAG00000004237	BTC	6	91430305	91480129	-	CODING	-0.36177	0.003204
ENSBTAT00000047985	ENSBTAG00000021064	-	21	15998170	16090486	+	CODING	0.428239	0.00321
ENSBTAT00000033431	ENSBTAG00000000074	NFIA	3	84749656	85167847	-	CODING	-0.01939	0.003275
ENSBTAT00000035681	ENSBTAG00000007494	SMARCA2	8	42652775	42830159	-	CODING	-0.22548	0.00331
ENSBTAT00000008132	ENSBTAG00000006189	ACTG1	19	51868429	51871276	+	CODING	-0.97625	0.003321
ENSBTAT00000000994	ENSBTAG00000000746	PCAF	1	44857815	44928998	+	CODING	-0.32123	0.003327
ENSBTAT00000063725	ENSBTAG00000045544	-	X	1.44E+08	1.44E+08	-	CODING	-0.27676	0.003342
ENSBTAT00000049047	ENSBTAG00000039875	-	9	88307676	88310265	-	CODING	2.5356	0.003358
ENSBTAT00000015746	ENSBTAG00000011864	RGMB	7	1E+08	1E+08	+	CODING	-0.13297	0.00336
ENSBTAT00000044557	ENSBTAG00000031441	FXYD5	18	46067593	46078935	+	CODING	-1.8656	0.003374
ENSBTAT00000026361	ENSBTAG00000019784	PPP2R5E	10	75953664	76109267	-	CODING	-0.06939	0.003387
ENSBTAT00000020612	ENSBTAG00000015511	FADS3	29	41087531	41102449	-	CODING	-1.85107	0.003389
ENSBTAT00000032276	ENSBTAG00000023607	PTPLB	1	68464487	68550787	-	CODING	-1.72722	0.003398
ENSBTAT00000053511	ENSBTAG00000038283	TMEM243	4	33347225	33368787	+	CODING	-1.68727	0.003406
ENSBTAT00000033238	ENSBTAG00000024097	-	3	1.1E+08	1.1E+08	+	CODING	-1.34198	0.003409
ENSBTAT00000015483	ENSBTAG00000011659	PIGZ	1	72024795	72028847	-	CODING	0.389951	0.003434
ENSBTAT00000019429	ENSBTAG00000014596	EFHD1	3	1.13E+08	1.13E+08	+	CODING	-1.91747	0.003459
ENSBTAT00000021328	ENSBTAG00000016024	MYL9	24	37820258	37829300	+	CODING	-0.90052	0.003467
ENSBTAT00000003575	ENSBTAG00000002758	THBD	13	42217371	42221004	-	CODING	-1.52458	0.00347
ENSBTAT00000036296	ENSBTAG00000019788	TEAD4	5	1.07E+08	1.07E+08	-	CODING	-0.01044	0.00347
ENSBTAT00000023937	ENSBTAG00000017992	CD164	9	41246268	41256989	+	CODING	-0.49757	0.003473
ENSBTAT00000011377	ENSBTAG00000024450	MAP2K2	7	21132389	21153974	+	CODING	-0.46139	0.003479
ENSBTAT00000027160	ENSBTAG00000020376	PAIP1	20	31284382	31311777	+	CODING	-0.25585	0.003487
ENSBTAT00000055617	ENSBTAG00000005225	RAD52	5	1.08E+08	1.08E+08	-	CODING	-1.3397	0.003501
ENSBTAT00000020113	ENSBTAG00000015116	RAD23A	7	13724192	13730226	-	CODING	-0.49115	0.00351
ENSBTAT00000010670	ENSBTAG00000008113	OSR1	11	79487139	79494238	+	CODING	-3.29136	0.003512
ENSBTAT00000007149	ENSBTAG00000005431	LMCD1	22	17961211	18018234	-	CODING	-0.49061	0.003519
ENSBTAT00000015465	ENSBTAG00000011645	U2AF1	1	1.45E+08	1.45E+08	-	CODING	-1.23266	0.003539
ENSBTAT00000002464	ENSBTAG00000001894	NEDD1	5	61446535	61498524	+	CODING	-0.30492	0.00354
ENSBTAT00000011234	ENSBTAG00000027442	NFIB	8	30029740	30155742	+	CODING	-0.09618	0.00357
ENSBTAT00000053074	ENSBTAG00000047537	CCAR1	28	25228951	25249032	+	CODING	-1.37744	0.003596
ENSBTAT00000000944	ENSBTAG00000000706	ADAMTS1	1	8955134	8963815	+	CODING	-1.2802	0.003602
ENSBTAT00000043199	ENSBTAG00000030584	SLC35A4	7	53424462	53426334	+	CODING	-0.21627	0.003628
ENSBTAT00000004350	ENSBTAG00000003352	-	23	25423551	25424597	+	CODING	-1.85181	0.003666
ENSBTAT00000035139	ENSBTAG00000025121	EMC6	19	24977284	24978133	+	CODING	-1.16695	0.003677
ENSBTAT00000014911	ENSBTAG00000011228	FASTK	4	1.14E+08	1.14E+08	-	CODING	0.243293	0.003736
ENSBTAT00000052142	ENSBTAG00000003758	TKT	22	48274004	48288061	+	CODING	-2.3414	0.003745
ENSBTAT00000021062	ENSBTAG00000015844	TFPI2	4	11035479	11039782	-	CODING	-1.23879	0.003765
ENSBTAT00000045437	ENSBTAG00000020855	PTPN4	2	72049607	72146322	+	CODING	0.047264	0.003784
ENSBTAT00000007833	ENSBTAG00000005967	UBR2	23	16264297	16370157	+	CODING	-0.25055	0.003788
ENSBTAT00000012256	ENSBTAG00000009304	PDCD5	18	43254168	43260903	+	CODING	-1.21337	0.0038
ENSBTAT00000022056	ENSBTAG00000016580	TEF	5	1.13E+08	1.13E+08	+	CODING	-0.01903	0.003805
ENSBTAT00000024663	ENSBTAG00000018530	TUBA8	5	1.1E+08	1.1E+08	+	CODING	-1.04205	0.003816
ENSBTAT00000001568	ENSBTAG00000001182	CDC10	4	61611420	61710127	-	CODING	-2.9485	0.003827
ENSBTAT00000033470	ENSBTAG00000024240	ACADM	3	69344157	69382504	-	CODING	-1.26037	0.003856
ENSBTAT00000028221	ENSBTAG00000021176	CRISPLD2	18	10985132	11050904	+	CODING	-1.33923	0.003866
ENSBTAT00000036705	ENSBTAG00000007665	NPR3	20	40967082	41041629	-	CODING	1.018799	0.003868

ENSBTAT00000035714	ENSBTAG00000025425	RAB24	7	40127136	40131892	-	CODING	-1.79313	0.003872
ENSBTAT00000016254	ENSBTAG00000012253	EIF2C1	3	1.1E+08	1.1E+08	-	CODING	0.214194	0.00389
ENSBTAT00000064259	ENSBTAG00000047426	LYPD6	2	46723405	46759004	-	CODING	0.943199	0.003904
ENSBTAT00000016771	ENSBTAG00000012637	C11H2orf40	11	45690020	45699417	-	CODING	-3.2418	0.003911
ENSBTAT00000013079	ENSBTAG00000009908	RPS3A	17	6703060	6707745	-	CODING	-0.94814	0.003955
ENSBTAT00000014098	ENSBTAG00000010659	CUX1	25	35243348	35515094	-	CODING	-0.10242	0.003958
ENSBTAT00000025691	ENSBTAG00000019295	MDH1	11	61970467	61994479	+	CODING	-0.61265	0.003967
ENSBTAT00000028446	ENSBTAG00000021341	BTBD1	21	25467798	25518118	+	CODING	-0.45853	0.00399
ENSBTAT00000064744	ENSBTAG00000047277	PNKD	18	46516557	46516985	+	CODING	-1.35153	0.003991
ENSBTAT00000049002	ENSBTAG00000007732	ARPP21	22	9674589	9681167	+	CODING	-2.53735	0.00402
ENSBTAT00000065817	ENSBTAG00000024929	PPP1R27	19	51658045	51659508	+	CODING	-4.21959	0.004025
ENSBTAT00000017940	ENSBTAG00000013492	PRKAG3	2	1.08E+08	1.08E+08	-	CODING	-0.35695	0.004074
ENSBTAT00000011603	ENSBTAG00000008810	-	3	1.01E+08	1.01E+08	+	CODING	-0.28914	0.004077
ENSBTAT00000012523	ENSBTAG00000009517	DBI	2	71561192	71566372	+	CODING	-1.06495	0.004102
ENSBTAT00000002578	ENSBTAG00000001987	HSPC321	15	43509670	43585976	-	CODING	-1.81392	0.004106
ENSBTAT00000004615	ENSBTAG00000003548	GSTP1	29	46087142	46090005	+	CODING	-1.04647	0.004134
ENSBTAT00000024320	ENSBTAG00000018272	RERE	16	45621457	45879645	+	CODING	-0.36875	0.004168
ENSBTAT00000031534	ENSBTAG00000002367	HCFC1R1	25	2442007	2443352	-	CODING	-0.49818	0.004188
ENSBTAT00000032005	ENSBTAG00000023472	PPP1R14A	18	48282679	48286857	-	CODING	-1.97236	0.004217
ENSBTAT00000018429	ENSBTAG00000013882	CHMP2A	18	65960799	65963496	-	CODING	-1.32698	0.004219
ENSBTAT00000030071	ENSBTAG00000013240	SLC3A2	29	41855871	41896760	+	CODING	-1.13409	0.004235
ENSBTAT00000017420	ENSBTAG00000013103	COL1A1	19	37088246	37104998	+	CODING	-0.31832	0.004255
ENSBTAT00000044085	ENSBTAG00000012103	MAP2K4	19	31284737	31363093	+	CODING	-0.15881	0.004255
ENSBTAT00000031993	ENSBTAG00000016313	ECH1	18	48811368	48820509	-	CODING	-1.17334	0.004385
ENSBTAT00000037763	ENSBTAG00000009251	SZRD1	2	1.36E+08	1.36E+08	-	CODING	0.052808	0.004401
ENSBTAT00000065691	ENSBTAG00000024958	FAM214A	10	57828793	57888673	+	CODING	-0.0266	0.004423
ENSBTAT00000057091	ENSBTAG00000001004	ESAM	29	28601863	28610589	-	CODING	-1.34585	0.004429
ENSBTAT00000002681	ENSBTAG00000002075	MME	1	1.13E+08	1.14E+08	-	CODING	-2.0202	0.004453
ENSBTAT00000027858	ENSBTAG00000020911	FNBP4	15	78675015	78717851	-	CODING	-1.39236	0.004472
ENSBTAT00000020860	ENSBTAG00000015711	BTG2	16	890072	892370	+	CODING	-0.35205	0.004475
ENSBTAT00000025832	ENSBTAG00000005146	-	23	28330539	28334072	-	CODING	-1.27172	0.00448
ENSBTAT00000008609	ENSBTAG00000039793	HDGF	3	14130196	14138913	+	CODING	-0.50987	0.004503
ENSBTAT00000022237	ENSBTAG00000016730	ANP32E	3	20453150	20465219	+	CODING	-0.36912	0.004509
ENSBTAT00000012952	ENSBTAG00000009824	COMMD6	17	29777299	29777556	+	CODING	0.648793	0.004514
ENSBTAT00000000700	ENSBTAG00000000539	LRRC28	21	7411027	7600816	-	CODING	-0.45517	0.004535
ENSBTAT00000022146	ENSBTAG00000016650	TIGAR	5	1.06E+08	1.06E+08	-	CODING	0.571316	0.00457
ENSBTAT00000015071	ENSBTAG00000011333	CLASP2	22	7809077	7982050	-	CODING	-0.24891	0.004572
ENSBTAT00000039096	ENSBTAG00000000817	SYNJ2	9	96023880	96091443	+	CODING	-3.62679	0.004598
ENSBTAT00000055646	ENSBTAG00000022825	BRI3	25	38281392	38287463	-	CODING	-1.16866	0.004617
ENSBTAT00000055244	ENSBTAG00000010336	TALDO1	29	50856122	50863474	-	CODING	-1.40384	0.004723
ENSBTAT00000035150	ENSBTAG00000005326	CSNK1A1	7	62861221	62902057	-	CODING	-0.03676	0.004741
ENSBTAT00000014476	ENSBTAG00000010899	TIMP2	19	54079297	54131052	+	CODING	-1.19736	0.004754
ENSBTAT00000045415	ENSBTAG00000004908	CHRNE	19	27118517	27123114	+	CODING	-0.00791	0.004785
ENSBTAT00000039408	ENSBTAG00000027442	NFIB	8	30010801	30128359	+	CODING	0.114785	0.004794
ENSBTAT00000022755	ENSBTAG00000017118	FAM8A1	23	39611984	39619623	-	CODING	-0.19035	0.004798
ENSBTAT00000020443	ENSBTAG00000015381	ARHGAP18	9	68713610	68777064	-	CODING	0.276999	0.004813
ENSBTAT00000009782	ENSBTAG00000007438	STT3B	22	6083636	6184904	+	CODING	-0.3339	0.004815
ENSBTAT00000009534	ENSBTAG00000007246	TMEM204	25	1194760	1207350	+	CODING	-1.77666	0.004855
ENSBTAT00000027944	ENSBTAG00000020983	RRAS	18	56497094	56501119	-	CODING	-1.34038	0.004859
ENSBTAT00000027885	ENSBTAG00000020935	HIF1A	10	74095881	74139364	+	CODING	-1.4949	0.004865
ENSBTAT00000003514	ENSBTAG00000038842	NEDD8	10	20777431	20779257	+	CODING	-0.42859	0.004881
ENSBTAT00000023267	ENSBTAG00000017504	FAIM2	5	30155763	30185025	+	CODING	-2.83502	0.004934
ENSBTAT00000019055	ENSBTAG00000014331	UBL4A	X	40458245	40460872	-	CODING	-1.85286	0.004939
ENSBTAT00000015210	ENSBTAG00000011446	SEMA7A	21	34626181	34651240	+	CODING	-1.77363	0.004945
ENSBTAT00000035445	ENSBTAG00000010943	SLC22A23	23	50234185	50323825	+	CODING	0.508542	0.004947
ENSBTAT00000066209	ENSBTAG00000045828	PTBP1	7	45016278	45025719	+	CODING	-1.95896	0.004955
ENSBTAT00000004528	ENSBTAG00000003485	MAGOH	3	93571582	93580843	+	CODING	-1.3167	0.004983
ENSBTAT00000028490	ENSBTAG00000021370	BLMH	19	21896194	21936565	-	CODING	-0.26298	0.004988
ENSBTAT00000053653	ENSBTAG00000038794	TMEM245	8	1E+08	1E+08	-	CODING	0.036473	0.004988
ENSBTAT00000054491	ENSBTAG00000013127	LPCAT3	5	1.04E+08	1.04E+08	+	CODING	-0.30418	0.004993
ENSBTAT00000064427	ENSBTAG00000022158	TNNT3	29	50218484	50233948	-	CODING	-1.08888	0.005004

ENSBTAT00000061174	ENSBTAG00000021250	RALBP1	24	42061941	42079438	+	CODING	-0.16366	0.005039
ENSBTAT00000056840	ENSBTAG00000037941	QTRT1	7	16415204	16423129	+	CODING	-2.04892	0.00505
ENSBTAT00000024791	ENSBTAG00000018631	SAP18	12	35860636	35868470	-	CODING	-0.4774	0.005128
ENSBTAT00000026470	ENSBTAG00000019867	COMMD1	11	60427663	60598437	+	CODING	-1.45838	0.005129
ENSBTAT00000010173	ENSBTAG00000007732	ARPP21	22	9683024	9791554	+	CODING	-1.69375	0.005135
ENSBTAT00000008305	ENSBTAG00000006330	RBM5	22	50789615	50813736	-	CODING	-1.27156	0.005135
ENSBTAT00000026699	ENSBTAG00000020042	KLHL28	21	55179305	55207518	-	CODING	0.306358	0.00514
ENSBTAT00000008789	ENSBTAG00000006679	MITF	22	31735990	31769463	-	CODING	-0.29136	0.005156
ENSBTAT00000021020	ENSBTAG00000015824	RNF141	15	42719433	42753584	+	CODING	-0.06528	0.005187
ENSBTAT00000004364	ENSBTAG00000003362	HSP90B1	5	67940792	67959532	+	CODING	-1.16737	0.005218
ENSBTAT00000007817	ENSBTAG00000005957	CSE1L	13	77919378	77954416	+	CODING	0.092705	0.005222
ENSBTAT00000064130	ENSBTAG00000047420	BTNL9	7	41643902	41650567	-	CODING	-1.51073	0.005242
ENSBTAT00000061327	ENSBTAG00000021073	KIAA1549	4	1.03E+08	1.03E+08	-	CODING	1.495277	0.005299
ENSBTAT00000011923	ENSBTAG00000009050	ADIPOR2	5	1.09E+08	1.09E+08	+	CODING	-1.15	0.0053
ENSBTAT00000015544	ENSBTAG00000011704	-	13	63150593	63151022	+	CODING	-1.42448	0.005386
ENSBTAT00000001747	ENSBTAG00000001324	SLCO2A1	1	1.36E+08	1.37E+08	+	CODING	-1.91766	0.005387
ENSBTAT00000024401	ENSBTAG00000018340	-	8	15978603	15979256	-	CODING	-1.28702	0.005391
ENSBTAT00000017080	ENSBTAG00000012852	DICER1	21	61513000	61555942	-	CODING	-0.04935	0.005421
ENSBTAT00000021544	ENSBTAG00000016185	ENAH	16	29238992	29442791	-	CODING	-0.11092	0.005427
ENSBTAT00000024326	ENSBTAG00000018278	ATP5O	1	922635	929992	+	CODING	-0.96081	0.005442
ENSBTAT00000012857	ENSBTAG00000009749	USP2	15	30464926	30487308	-	CODING	-1.12348	0.005443
ENSBTAT00000021305	ENSBTAG00000016005	PPP3CA	6	24812682	25136247	+	CODING	0.03494	0.005451
ENSBTAT00000018843	ENSBTAG00000014176	REM1	13	61619569	61628687	+	CODING	-1.88842	0.005482
ENSBTAT00000019577	ENSBTAG00000014712	CLPP	7	19302913	19308138	-	CODING	-1.25028	0.005496
ENSBTAT00000001191	ENSBTAG00000000898	F2RL2	10	7777600	7788321	-	CODING	-2.24956	0.005499
ENSBTAT00000064420	ENSBTAG00000047747	NDFIP1	7	55061328	55085013	+	CODING	-0.35747	0.00552
ENSBTAT00000025911	ENSBTAG00000019451	TAPT1	6	1.16E+08	1.16E+08	-	CODING	-0.09724	0.005529
ENSBTAT00000000177	ENSBTAG00000000154	IRF2BP1	X	11966614	11968272	+	CODING	-0.05181	0.005589
ENSBTAT00000029348	ENSBTAG00000022004	FLNB	22	43674720	43815706	-	CODING	-1.56063	0.005591
ENSBTAT00000038558	ENSBTAG00000007109	ASB2	21	59126042	59160261	-	CODING	-0.66877	0.005598
ENSBTAT00000013787	ENSBTAG00000010447	LSP1	29	50238211	50276209	-	CODING	-1.65321	0.005644
ENSBTAT00000015282	ENSBTAG00000006531	DIP2C	13	47041229	47177126	+	CODING	-0.0562	0.00572
ENSBTAT00000063575	ENSBTAG00000045633	AGTR1	1	1.2E+08	1.2E+08	-	CODING	-1.77198	0.005741
ENSBTAT00000014308	ENSBTAG00000010801	CMBL	20	63065548	63090743	+	CODING	-0.49591	0.005745
ENSBTAT00000008066	ENSBTAG00000006130	CLEC14A	21	48843729	48845622	-	CODING	-1.34629	0.005804
ENSBTAT00000060978	ENSBTAG00000010664	PRKCQ	13	17093614	17185063	+	CODING	-0.30857	0.005821
ENSBTAT00000017898	ENSBTAG00000013455	SPOPL	2	58798302	58846989	-	CODING	0.072555	0.005826
ENSBTAT00000001748	ENSBTAG00000001323	CENPC1	6	84904999	84998863	-	CODING	-2.53496	0.00584
ENSBTAT00000064158	ENSBTAG00000046248	ARPC1B	25	37546485	37559078	-	CODING	-1.59446	0.005846
ENSBTAT00000065271	ENSBTAG00000047561	VEGFA	23	17257357	17261031	-	CODING	-1.30504	0.005875
ENSBTAT00000007943	ENSBTAG00000006045	P4HB	19	51643765	51653754	+	CODING	-1.11465	0.005895
ENSBTAT00000045455	ENSBTAG00000010153	ANXA3	6	95065256	95136945	+	CODING	-1.19992	0.005899
ENSBTAT00000018261	ENSBTAG00000013745	ITGA5	5	25778012	25799053	+	CODING	-1.69252	0.005926
ENSBTAT00000031279	ENSBTAG00000005077	CXCL12	28	45410676	45418793	+	CODING	-1.42435	0.005939
ENSBTAT00000056481	ENSBTAG00000005497	RPRD1A	24	21361884	21398835	+	CODING	-2.28985	0.006062
ENSBTAT00000024848	ENSBTAG00000018671	DCUN1D2	12	90680190	90695973	-	CODING	-0.49714	0.006066
ENSBTAT00000015319	ENSBTAG00000011528	SMIM11	1	463572	478996	-	CODING	-1.15784	0.006199
ENSBTAT00000063057	ENSBTAG00000046335	-	29	272264	273010	+	CODING	-0.64386	0.00626
ENSBTAT00000046344	ENSBTAG00000012393	AGT	28	46147260	46156093	+	CODING	-2.14226	0.006297
ENSBTAT00000023912	ENSBTAG00000017970	ZYX	4	1.08E+08	1.08E+08	+	CODING	-1.17324	0.006302
ENSBTAT00000001698	ENSBTAG00000001288	MAOB	X	1.05E+08	1.05E+08	+	CODING	-0.07054	0.006311
ENSBTAT00000039864	ENSBTAG00000027676	IL18BP	15	52416834	52418796	+	CODING	-10.8012	0.006349
ENSBTAT00000028723	ENSBTAG00000021558	-	13	55509535	55517058	-	CODING	-0.31209	0.006351
ENSBTAT00000023198	ENSBTAG00000017451	TSPAN17	7	39472617	39481385	+	CODING	-0.21443	0.006352
ENSBTAT00000013864	ENSBTAG00000010490	PEX5	5	1.04E+08	1.04E+08	-	CODING	1.001239	0.006357
ENSBTAT00000011070	ENSBTAG00000008412	BCL7C	25	27215012	27218439	-	CODING	-1.87566	0.006412
ENSBTAT00000036795	ENSBTAG00000012442	CTSB	8	7414945	7423429	+	CODING	-1.09449	0.006431
ENSBTAT00000060552	ENSBTAG00000043577	MT-ND4	MT	10529	11906	+	CODING	-0.78537	0.006451
ENSBTAT00000018468	ENSBTAG00000025263	FAM160A1	17	6089371	6207768	-	CODING	-0.05162	0.006478
ENSBTAT00000044703	ENSBTAG00000001790	SAFB2	7	19908341	19936959	+	CODING	-1.44734	0.006506
ENSBTAT00000011270	ENSBTAG00000008548	FAM98A	11	16157514	16173332	-	CODING	-0.41893	0.006514

ENSBTAT00000001567	ENSBTAG00000001183	KLHL33	10	26728622	26738305	+	CODING	-0.36947	0.006541
ENSBTAT00000056287	ENSBTAG00000001361	NMNAT1	16	44457295	44462940	-	CODING	-0.15931	0.006579
ENSBTAT00000066329	ENSBTAG00000046620	RPL38	1	1.41E+08	1.41E+08	-	CODING	-3.11853	0.006622
ENSBTAT00000017169	ENSBTAG00000012920	TMEM70	14	39354351	39362288	+	CODING	-1.26445	0.006625
ENSBTAT00000026053	ENSBTAG00000019552	PGRMC1	X	3468827	3477212	+	CODING	-1.68019	0.006651
ENSBTAT00000001494	ENSBTAG00000001123	SNRNP48	23	47647768	47663909	-	CODING	-1.795	0.006659
ENSBTAT00000029167	ENSBTAG00000021880	ADCK3	16	30656001	30703396	+	CODING	-0.98688	0.006702
ENSBTAT00000021218	ENSBTAG00000015955	ERGIC1	20	4505275	4618413	+	CODING	-0.19914	0.006763
ENSBTAT00000047358	ENSBTAG00000033315	DNAJC1	13	23251449	23428980	-	CODING	5.296727	0.006789
ENSBTAT00000014236	ENSBTAG00000010745	THRA	19	41017271	41042023	+	CODING	-0.32739	0.006808
ENSBTAT00000012026	ENSBTAG00000009126	YBX2	19	27622773	27628780	-	CODING	-2.48082	0.006812
ENSBTAT00000020766	ENSBTAG00000015637	IMPAD1	14	25544907	25560879	-	CODING	-0.17494	0.006874
ENSBTAT00000018329	ENSBTAG00000015996	GPC1	3	1.2E+08	1.2E+08	+	CODING	-0.18055	0.006886
ENSBTAT00000008621	ENSBTAG00000006564	PSMA1	15	38745761	38758646	+	CODING	-1.09197	0.006909
ENSBTAT00000047480	ENSBTAG00000006970	OLA1	2	22564518	22590410	+	CODING	0.865119	0.006926
ENSBTAT00000000104	ENSBTAG00000000094	ATPAF1	3	1E+08	1E+08	+	CODING	-0.43385	0.006964
ENSBTAT00000026387	ENSBTAG00000019804	SNRNP25	25	113120	115274	+	CODING	-1.12284	0.006978
ENSBTAT00000024782	ENSBTAG00000037377	ABHD14B	22	49516824	49520467	+	CODING	-1.66159	0.006986
ENSBTAT00000063570	ENSBTAG00000045702	ZBED6	16	1400457	1403399	+	CODING	-0.102	0.007032
ENSBTAT00000035789	ENSBTAG00000019685	BAG6	23	27463438	27474655	+	CODING	-0.38222	0.007043
ENSBTAT00000010969	ENSBTAG00000008330	RNF19B	2	1.22E+08	1.22E+08	+	CODING	-0.09827	0.007063
ENSBTAT00000027190	ENSBTAG00000020403	ZMYM2	12	36391824	36453989	-	CODING	-0.15102	0.007082
ENSBTAT00000005059	ENSBTAG00000003876	MCU	28	28868083	29061027	+	CODING	-0.40886	0.007086
ENSBTAT00000061359	ENSBTAG00000044071	HRH2	10	5409681	5410760	-	CODING	1.023431	0.007111
ENSBTAT00000053988	ENSBTAG00000037413	TMEM164	X	62882939	63056847	+	CODING	-0.05215	0.007119
ENSBTAT00000065236	ENSBTAG00000046309	-	10	73129829	73133979	-	CODING	-0.25412	0.007126
ENSBTAT00000064703	ENSBTAG00000047621	-	16	4998445	5005553	+	CODING	-1.69726	0.007134
ENSBTAT00000008406	ENSBTAG00000006411	THAP11	18	35472716	35474389	+	CODING	-0.38228	0.007206
ENSBTAT00000030181	ENSBTAG00000033032	FAM50A	X	40417745	40423500	+	CODING	-1.45201	0.007214
ENSBTAT00000063644	ENSBTAG00000002938	CPED1	4	86250173	86572861	+	CODING	-0.0753	0.007252
ENSBTAT00000037984	ENSBTAG00000013309	SFRS4	2	1.25E+08	1.25E+08	+	CODING	-1.32761	0.007274
ENSBTAT00000009128	ENSBTAG00000006950	COPS4	6	99610792	99649394	+	CODING	-0.54978	0.007359
ENSBTAT00000016938	ENSBTAG00000012741	CCPG1	10	55001792	55028162	+	CODING	-0.47054	0.007369
ENSBTAT00000057594	ENSBTAG00000011198	PPP1CC	17	56814813	56833870	-	CODING	-0.4546	0.007403
ENSBTAT00000027322	ENSBTAG00000020504	CDK2AP1	17	54556011	54566572	+	CODING	-0.47317	0.007408
ENSBTAT00000060539	ENSBTAG00000043584	ATP6	MT	8290	8970	+	CODING	-0.81966	0.007468
ENSBTAT00000012099	ENSBTAG00000009182	CLCN1	4	1.08E+08	1.08E+08	+	CODING	-0.24573	0.007488
ENSBTAT00000043205	ENSBTAG00000030587	LASP1	19	40090995	40131373	+	CODING	-1.41197	0.007497
ENSBTAT00000011543	ENSBTAG00000008763	PRR32	X	10981501	10983415	+	CODING	-1.92621	0.007605
ENSBTAT00000001121	ENSBTAG00000000843	NDRG2	10	26134842	26142255	+	CODING	-0.64034	0.007664
ENSBTAT00000028307	ENSBTAG00000021245	SPRY1	17	34749102	34753222	-	CODING	-1.3592	0.007685
ENSBTAT00000025935	ENSBTAG00000019470	NEK6	11	95314593	95400813	+	CODING	-1.44893	0.007693
ENSBTAT00000023772	ENSBTAG00000017885	FAM173B	20	63111708	63127170	+	CODING	0.047393	0.007697
ENSBTAT00000005165	ENSBTAG00000003959	ARHGAP24	6	1.03E+08	1.03E+08	+	CODING	-0.04457	0.007701
ENSBTAT00000042610	ENSBTAG00000009667	SLC25A46	7	1.12E+08	1.12E+08	+	CODING	-0.17005	0.007706
ENSBTAT00000003373	ENSBTAG00000002603	PRPF39	21	55289715	55324776	+	CODING	-1.48537	0.007741
ENSBTAT00000053057	ENSBTAG00000032021	RALB	2	72372371	72430777	+	CODING	-1.35851	0.007838
ENSBTAT00000022718	ENSBTAG00000017082	POR	25	34672093	34726073	+	CODING	-1.52877	0.007859
ENSBTAT00000025662	ENSBTAG00000019274	ATIC	2	1.04E+08	1.04E+08	+	CODING	-0.44146	0.00787
ENSBTAT00000031716	ENSBTAG00000021204	TES	4	52477241	52532254	-	CODING	-1.69957	0.007878
ENSBTAT00000026389	ENSBTAG00000019806	YPEL2	19	10546157	10593841	+	CODING	-0.02873	0.007898
ENSBTAT00000004201	ENSBTAG00000003237	IQSEC1	22	59460406	59549010	+	CODING	0.264896	0.007962
ENSBTAT00000053143	ENSBTAG00000038351	-	5	441593	442266	-	CODING	1.143379	0.007967
ENSBTAT00000026800	ENSBTAG00000020121	RWDD2B	1	6521212	6532969	+	CODING	0.003439	0.008007
ENSBTAT00000065986	ENSBTAG00000046794	-	X	1.04E+08	1.04E+08	-	CODING	-0.28642	0.008042
ENSBTAT00000023213	ENSBTAG00000017462	ATF4	5	1.11E+08	1.11E+08	+	CODING	-0.74198	0.008054
ENSBTAT00000006875	ENSBTAG00000005221	WNK1	5	1.08E+08	1.08E+08	+	CODING	-0.25806	0.008067
ENSBTAT00000014032	ENSBTAG00000010606	PPP1R3B	27	24316623	24317477	-	CODING	0.113227	0.008068
ENSBTAT00000011076	ENSBTAG00000008417	VPS29	17	56617708	56622846	-	CODING	-1.23998	0.008076
ENSBTAT00000053957	ENSBTAG00000039764	IER5	16	63689680	63690654	+	CODING	-1.69753	0.008078
ENSBTAT00000004879	ENSBTAG00000003746	SCP2	3	93846489	93968364	-	CODING	-0.47748	0.008082

ENSBTAT00000019983	ENSBTAG00000015007	NCOA1	11	74511607	74590924	-	CODING	-0.00938	0.008085
ENSBTAT00000008676	ENSBTAG00000006614	TMCC1	22	56700645	56742873	+	CODING	-1.11465	0.008158
ENSBTAT00000019182	ENSBTAG00000014422	MTMR10	21	27947773	27984436	-	CODING	-0.13554	0.008191
ENSBTAT00000032851	ENSBTAG00000002108	YWHAQ	11	87842280	87873674	+	CODING	-1.33719	0.008211
ENSBTAT00000029211	ENSBTAG00000021910	TBL1XR1	1	90517079	90605473	+	CODING	-0.26747	0.008226
ENSBTAT00000061376	ENSBTAG00000003667	TLN2	10	47303876	47796327	-	CODING	0.042424	0.00824
ENSBTAT00000026634	ENSBTAG00000019997	TRHR	14	57479102	57524004	-	CODING	4.274829	0.008277
ENSBTAT00000015199	ENSBTAG00000011435	NPEPPS	19	39460692	39564822	-	CODING	-0.39301	0.008313
ENSBTAT00000009422	ENSBTAG00000007159	ESR1	9	89969586	90255801	+	CODING	0.218808	0.008346
ENSBTAT00000031490	ENSBTAG00000023147	LINGO1	21	33292504	33294348	-	CODING	-0.48463	0.008433
ENSBTAT00000003434	ENSBTAG00000032899	-	5	29080926	29082000	-	CODING	0.077842	0.008438
ENSBTAT00000063474	ENSBTAG00000000139	SETD8	17	54457612	54471710	-	CODING	-0.18064	0.008463
ENSBTAT00000028721	ENSBTAG00000021556	RANBP9	23	42736705	42795080	+	CODING	-0.33309	0.0085
ENSBTAT00000060994	ENSBTAG00000013108	HK2	11	9723269	9766920	-	CODING	-1.57702	0.00856
ENSBTAT00000012391	ENSBTAG00000009414	PSMC4	18	49688182	49699038	+	CODING	-1.18103	0.008568
ENSBTAT00000007177	ENSBTAG00000005455	USP7	25	7769457	7796941	-	CODING	-0.41249	0.008574
ENSBTAT00000007052	ENSBTAG00000005364	HMBS	15	30194980	30202275	+	CODING	-0.2407	0.008576
ENSBTAT00000028329	ENSBTAG00000021260	GALNT11	4	1.15E+08	1.15E+08	+	CODING	-0.12647	0.008586
ENSBTAT00000063192	ENSBTAG00000046177	IGFN1	16	81604665	81641464	+	CODING	-0.5634	0.00863
ENSBTAT00000016923	ENSBTAG00000012729	ARHGEF9	X	1.02E+08	1.02E+08	+	CODING	-2.1132	0.008649
ENSBTAT00000066178	ENSBTAG00000048280	C12orf73	5	67960359	67967214	-	CODING	-1.30572	0.008686
ENSBTAT00000052388	ENSBTAG00000005434	PLVAP	7	5685231	5704120	+	CODING	-1.90856	0.008697
ENSBTAT00000014053	ENSBTAG00000010624	DCTN2	5	56262302	56275883	+	CODING	-1.10441	0.008731
ENSBTAT00000038477	ENSBTAG00000015396	RBM24	23	39835982	39846198	-	CODING	-0.32424	0.008777
ENSBTAT00000031069	ENSBTAG00000007784	NAA50	1	58847733	58879203	-	CODING	-0.58527	0.008779
ENSBTAT00000064297	ENSBTAG00000048308	ICMT	16	47960246	47967797	-	CODING	0.090245	0.008803
ENSBTAT00000023671	ENSBTAG00000017803	RAB14	8	1.13E+08	1.13E+08	-	CODING	-0.33359	0.00881
ENSBTAT00000015166	ENSBTAG00000011412	LAMB1	4	49263419	49339822	-	CODING	-1.5964	0.008814
ENSBTAT00000016614	ENSBTAG00000012514	PODN	3	93728705	93751305	-	CODING	-1.41138	0.008877
ENSBTAT00000000114	ENSBTAG00000000105	RIT1	3	14908141	14917339	+	CODING	-1.53615	0.008895
ENSBTAT00000021559	ENSBTAG00000016199	CLINT1	7	71421947	71479026	-	CODING	-0.40709	0.008934
ENSBTAT00000000240	ENSBTAG00000000203	ATP6V1G1	8	1.05E+08	1.06E+08	+	CODING	-1.05595	0.008955
ENSBTAT00000022854	ENSBTAG00000017196	PDIA3	21	55924230	55946434	+	CODING	-1.11047	0.008979
ENSBTAT00000002344	ENSBTAG00000007875	SAFB	7	19846208	19876661	-	CODING	-1.34194	0.009
ENSBTAT00000013242	ENSBTAG00000010042	RIOK3	24	33523340	33545584	-	CODING	-0.40172	0.009022
ENSBTAT00000036427	ENSBTAG00000017077	CTSL	8	84976257	84981349	-	CODING	-2.24301	0.009023
ENSBTAT00000049076	ENSBTAG00000034645	PON3	4	12441126	12478659	-	CODING	-0.12074	0.009044
ENSBTAT00000064288	ENSBTAG00000046566	-	3	27428793	27430601	-	CODING	0.072795	0.009046
ENSBTAT00000013146	ENSBTAG00000037811	CCL2	19	16232968	16234839	-	CODING	-1.32599	0.009065
ENSBTAT00000021256	ENSBTAG00000015978	ANXA1	8	49624473	49642916	+	CODING	-1.65375	0.009104
ENSBTAT00000003758	ENSBTAG00000002890	RBM10	X	90794365	90820965	+	CODING	-1.46956	0.009123
ENSBTAT00000002998	ENSBTAG00000002323	USP28	15	24669574	24733111	-	CODING	-0.42275	0.009227
ENSBTAT00000066224	ENSBTAG00000048160	TIGD5	14	2311790	2313733	-	CODING	0.412825	0.009229
ENSBTAT00000015631	ENSBTAG00000011772	PPP1R12B	16	71062132	71090113	+	CODING	0.252173	0.009236
ENSBTAT00000026460	ENSBTAG00000034366	RGS2	16	13041921	13045220	-	CODING	-1.59374	0.00926
ENSBTAT00000004655	ENSBTAG00000003578	DPCD	26	22205907	22227992	+	CODING	-1.61019	0.009274
ENSBTAT00000014511	ENSBTAG00000010923	RAB9A	X	1.37E+08	1.37E+08	-	CODING	-1.48316	0.009274
ENSBTAT00000027226	ENSBTAG00000020429	MED29	18	49351575	49355101	+	CODING	-1.31267	0.009319
ENSBTAT00000063012	ENSBTAG00000045699	CTNNA3	28	22419203	24270401	-	CODING	0.416833	0.009325
ENSBTAT00000055090	ENSBTAG00000011476	HOXA9	4	69331792	69334928	+	CODING	-1.44037	0.009329
ENSBTAT00000008891	ENSBTAG00000009554	EHBP1L1	29	44424071	44439541	+	CODING	-0.32233	0.009391
ENSBTAT00000029017	ENSBTAG00000021768	CCNG2	6	93746807	93758039	+	CODING	-0.09365	0.00942
ENSBTAT00000007933	ENSBTAG00000006039	ARHGDIB	5	95376923	95395815	+	CODING	-1.29562	0.009452
ENSBTAT00000015705	ENSBTAG00000011834	CCDC47	19	48662435	48682292	-	CODING	-0.53057	0.009479
ENSBTAT00000016953	ENSBTAG00000012757	GCNT1	8	52848304	52850731	+	CODING	-1.49594	0.009531
ENSBTAT00000044960	ENSBTAG00000031709	PDCD10	1	1.01E+08	1.01E+08	+	CODING	-1.45346	0.009531
ENSBTAT00000006903	ENSBTAG00000005247	FRMD3	8	77785287	77828221	-	CODING	-0.44805	0.00954
ENSBTAT00000038641	ENSBTAG00000026994	C2H2orf88	2	6038403	6113008	-	CODING	1.307871	0.009544
ENSBTAT00000003340	ENSBTAG00000002579	FBL	18	49659188	49718941	-	CODING	-1.33549	0.009546
ENSBTAT00000007444	ENSBTAG00000005666	LRRC20	28	26665369	26735793	-	CODING	-0.16579	0.009547
ENSBTAT00000006285	ENSBTAG00000004787	METRNL	19	50243829	50258586	-	CODING	-1.72702	0.009652

ENSBTAT00000032193	ENSBTAG00000016341	RGS5	3	6228349	6426528	+	CODING	-1.05292	0.009679
ENSBTAT00000002051	ENSBTAG00000001568	PPIC	7	32114445	32128245	+	CODING	-1.6912	0.009685
ENSBTAT00000013200	ENSBTAG00000010009	TMCO1	3	3166689	3222619	+	CODING	-1.30218	0.00971
ENSBTAT00000029239	ENSBTAG00000021932	RNASEK	19	27436503	27438226	+	CODING	-1.03664	0.009739
ENSBTAT00000026511	ENSBTAG00000019900	LNPEP	7	98825233	98878164	+	CODING	-0.0428	0.009742
ENSBTAT00000025313	ENSBTAG00000019018	-	11	1.07E+08	1.07E+08	+	CODING	-1.72968	0.0098
ENSBTAT00000006215	ENSBTAG00000004735	CCNE1	18	40565286	40574820	+	CODING	0.348933	0.009812
ENSBTAT00000023029	ENSBTAG00000017321	SUMO3	1	1.45E+08	1.45E+08	-	CODING	-0.40856	0.009826
ENSBTAT00000035470	ENSBTAG00000011721	MED13	19	11288860	11379955	+	CODING	-0.14943	0.009846
ENSBTAT00000048910	ENSBTAG00000034529	HMGA1	23	8258750	8268920	+	CODING	-0.31452	0.009851
ENSBTAT00000031822	ENSBTAG00000023377	SH3BP5	1	1.54E+08	1.54E+08	-	CODING	-0.44386	0.009874
ENSBTAT00000017102	ENSBTAG00000012870	BMP5	23	4390362	4533900	+	CODING	0.033793	0.009881
ENSBTAT00000005076	ENSBTAG00000003889	PER1	19	28390594	28399600	-	CODING	-1.22961	0.009965
ENSBTAT00000008268	ENSBTAG00000006296	VMA21	X	34102420	34112798	+	CODING	-0.39756	0.010032
ENSBTAT00000029279	ENSBTAG00000021960	GPX8	20	23975640	23980445	-	CODING	-1.49761	0.01005
ENSBTAT00000061629	ENSBTAG00000040215	EIF4G3	2	1.32E+08	1.32E+08	+	CODING	-0.10425	0.010161
ENSBTAT00000022774	ENSBTAG00000017135	CTSS	3	20024302	20047228	+	CODING	-1.52777	0.010189
ENSBTAT00000002431	ENSBTAG00000001865	SGCG	12	34924193	34954970	-	CODING	-0.40378	0.01019
ENSBTAT00000032910	ENSBTAG00000023929	FOSL2	11	71329772	71349193	-	CODING	-1.56764	0.010204
ENSBTAT00000042547	ENSBTAG00000030164	RPL38	19	57835002	57839034	-	CODING	-0.94	0.010259
ENSBTAT00000001642	ENSBTAG00000001244	PLAT	27	36738833	36762837	-	CODING	-1.95594	0.010271
ENSBTAT00000013153	ENSBTAG00000009976	-	2	84413662	84414878	-	CODING	-0.38315	0.010276
ENSBTAT00000018986	ENSBTAG00000014289	ZFYVE9	3	94600486	94713143	-	CODING	-0.14009	0.010299
ENSBTAT00000022178	ENSBTAG00000016679	ETFDH	17	41262754	41307126	-	CODING	-0.47201	0.010328
ENSBTAT00000022677	ENSBTAG00000017053	ABCB10	28	492423	525181	-	CODING	0.075727	0.010403
ENSBTAT00000028246	ENSBTAG00000021199	APOPT1	21	69847359	69873999	+	CODING	-1.27296	0.010404
ENSBTAT00000024017	ENSBTAG00000018041	ACADSB	26	43134415	43184345	+	CODING	-0.40769	0.010426
ENSBTAT00000005730	ENSBTAG00000004371	CS	5	57366008	57389596	+	CODING	-0.60136	0.010544
ENSBTAT00000008473	ENSBTAG00000006463	DLST	10	86533853	86550983	+	CODING	-0.47126	0.010552
ENSBTAT00000001526	ENSBTAG00000001146	HIVEP2	9	81237678	81266493	-	CODING	-0.19305	0.010562
ENSBTAT00000015499	ENSBTAG00000011677	HIST1H1C	23	31636947	31637588	+	CODING	0.268819	0.010582
ENSBTAT00000063207	ENSBTAG00000047942	DESI1	5	1.13E+08	1.13E+08	-	CODING	-1.5318	0.01062
ENSBTAT00000001723	ENSBTAG00000001305	ATP2B2	22	55001027	55302080	-	CODING	0.362254	0.010678
ENSBTAT00000011856	ENSBTAG00000009005	DUSP15	13	61941884	61950327	-	CODING	-0.24974	0.010717
ENSBTAT00000008068	ENSBTAG00000006134	DYNLRB1	13	64466198	64483930	+	CODING	-1.09751	0.010752
ENSBTAT00000044026	ENSBTAG00000003809	PLCD4	2	1.07E+08	1.07E+08	+	CODING	-1.23815	0.010802
ENSBTAT00000053246	ENSBTAG00000006670	ERGIC3	13	65435895	65448951	+	CODING	-1.35629	0.010855
ENSBTAT00000003318	ENSBTAG00000002562	PRAF2	X	92231884	92234517	-	CODING	-1.42373	0.011017
ENSBTAT00000043155	ENSBTAG00000013926	FCGRT	18	56415980	56421861	+	CODING	-3.29585	0.01103
ENSBTAT00000020032	ENSBTAG00000015050	HCN2	7	44831440	44850882	+	CODING	0.469629	0.011071
ENSBTAT00000002225	ENSBTAG00000001697	TRA2B	1	81878691	81898384	+	CODING	-1.19498	0.011074
ENSBTAT00000009285	ENSBTAG00000007062	IGFBP5	2	1.05E+08	1.05E+08	-	CODING	-0.21361	0.011084
ENSBTAT00000003415	ENSBTAG00000002633	42987	19	55118592	55153750	-	CODING	-1.40936	0.011115
ENSBTAT00000027974	ENSBTAG00000021008	ZNF219	10	26053957	26056906	+	CODING	0.006322	0.011118
ENSBTAT00000030639	ENSBTAG00000014474	API5	15	74303493	74321171	+	CODING	-0.27931	0.011215
ENSBTAT00000027256	ENSBTAG00000020454	-	13	52494961	52500593	+	CODING	0.003644	0.011244
ENSBTAT00000020154	ENSBTAG00000015151	FOXN2	11	30534467	30557119	+	CODING	-0.15697	0.011252
ENSBTAT00000032947	ENSBTAG00000023947	AVPI1	26	18711832	18719926	-	CODING	-1.58243	0.011268
ENSBTAT00000013405	ENSBTAG00000010157	TOMM22	5	1.11E+08	1.11E+08	+	CODING	-1.09531	0.0113
ENSBTAT00000026074	ENSBTAG00000019569	CD151	29	50695518	50700467	-	CODING	-1.15727	0.011303
ENSBTAT00000020550	ENSBTAG00000015457	FGFR1	27	33250534	33291989	-	CODING	-1.61686	0.011336
ENSBTAT00000035742	ENSBTAG00000025434	ZFP36L1	10	80965364	80970846	-	CODING	-1.18664	0.011357
ENSBTAT00000007890	ENSBTAG00000006007	SH3GL1	7	20923762	20951051	+	CODING	-1.65817	0.011549
ENSBTAT00000015625	ENSBTAG00000011767	MPP5	10	79536643	79579325	+	CODING	-0.2269	0.011584
ENSBTAT00000021769	ENSBTAG00000016367	RBM18	11	93101473	93121906	-	CODING	-0.27316	0.011601
ENSBTAT00000015431	ENSBTAG00000011619	RASIP1	18	55810633	55824241	-	CODING	-1.42327	0.011621
ENSBTAT00000027927	ENSBTAG00000020968	MPC2	3	768307	799012	+	CODING	-1.06855	0.01163
ENSBTAT00000027695	ENSBTAG00000020782	CNOT1	18	26390076	26447574	-	CODING	-0.34858	0.011652
ENSBTAT00000044700	ENSBTAG00000013176	SMOC2	9	1.04E+08	1.04E+08	+	CODING	0.055596	0.011666
ENSBTAT00000046801	ENSBTAG00000032964	C24H18orf25	24	46409594	46453472	+	CODING	-0.26179	0.011671
ENSBTAT00000022068	ENSBTAG00000016591	RAB11FIP3	25	404964	464143	+	CODING	-0.0808	0.011686

ENSBTAT00000023319	ENSBTAG00000017542	PPARD	23	9340955	9353750	+	CODING	-1.83275	0.011698
ENSBTAT00000022592	ENSBTAG00000016988	PIN1	7	15532998	15545250	+	CODING	-1.16792	0.011811
ENSBTAT00000024617	ENSBTAG00000018497	SDPR	2	80858087	80871118	-	CODING	-1.29438	0.011819
ENSBTAT00000039221	ENSBTAG00000034436	PDPK1	25	2062819	2097196	+	CODING	-0.06644	0.011822
ENSBTAT00000009070	ENSBTAG00000006904	TENC1	5	27069113	27082523	-	CODING	-1.45187	0.011866
ENSBTAT00000042576	ENSBTAG00000018661	TMC6	19	54647278	54666987	+	CODING	-2.75054	0.011882
ENSBTAT00000024666	ENSBTAG00000018531	IGJ	6	87759438	87768834	-	CODING	-1.96465	0.011916
ENSBTAT00000061313	ENSBTAG00000043951	IMPA2	24	43189865	43209021	+	CODING	-1.86326	0.011951
ENSBTAT00000008822	ENSBTAG00000006708	ARIH1	10	19197514	19300694	+	CODING	-0.39453	0.01198
ENSBTAT00000008170	ENSBTAG00000006222	TFDP2	1	1.28E+08	1.28E+08	+	CODING	-0.28902	0.011987
ENSBTAT00000046665	ENSBTAG00000013555	ACVR1B	5	28038532	28070153	-	CODING	0.382097	0.012041
ENSBTAT00000017292	ENSBTAG00000013007	C13H20ORF1	13	60109036	60119697	-	CODING	-0.56333	0.012046
ENSBTAT00000021895	ENSBTAG00000016471	PDPR	18	1910952	1951890	-	CODING	0.013776	0.012065
ENSBTAT00000003503	ENSBTAG00000002706	GSTZ1	10	89596394	89604430	+	CODING	0.426165	0.012075
ENSBTAT00000018052	ENSBTAG00000013579	TMEM66	27	25391027	25416076	-	CODING	-0.54235	0.012168
ENSBTAT00000018578	ENSBTAG00000013981	SPTSSA	21	45302740	45321878	-	CODING	-1.1954	0.012272
ENSBTAT00000053067	ENSBTAG00000019419	NDUFS7	7	45427274	45433438	+	CODING	-2.18335	0.012289
ENSBTAT00000018177	ENSBTAG00000013678	OSGIN2	14	76149588	76178108	-	CODING	-0.13592	0.012358
ENSBTAT00000013690	ENSBTAG00000010365	SQRDL	10	65039298	65093133	-	CODING	-1.63652	0.012361
ENSBTAT00000007806	ENSBTAG00000005947	PLAU	28	29964983	29971029	+	CODING	-1.65595	0.012421
ENSBTAT00000026127	ENSBTAG00000019612	RNASE4	10	26423874	26445617	-	CODING	-1.24586	0.012423
ENSBTAT00000009243	ENSBTAG00000007024	-	5	1.04E+08	1.04E+08	-	CODING	-2.00577	0.012438
ENSBTAT00000003319	ENSBTAG00000002563	WDR45	X	92234971	92240178	-	CODING	-1.33317	0.012541
ENSBTAT00000008194	ENSBTAG00000006244	WDR83OS	7	13940092	13953200	+	CODING	-1.09657	0.012631
ENSBTAT00000013178	ENSBTAG00000015450	CPEB3	26	13613121	13763563	-	CODING	-0.35188	0.012636
ENSBTAT00000047258	ENSBTAG00000018517	VLDLR	8	42109679	42141155	-	CODING	-0.46489	0.012636
ENSBTAT00000020845	ENSBTAG00000015698	FYCO1	22	53951029	54026241	+	CODING	-0.22488	0.01265
ENSBTAT00000057062	ENSBTAG00000012341	CSNK2A1	13	61082324	61106498	+	CODING	-0.46257	0.012679
ENSBTAT00000006554	ENSBTAG00000004979	MRS2	23	33038551	33077190	-	CODING	-0.25644	0.012765
ENSBTAT00000028717	ENSBTAG00000021553	PTPN14	16	70619178	70749808	+	CODING	-0.072	0.012781
ENSBTAT00000017775	ENSBTAG00000013363	CAP1	3	1.07E+08	1.07E+08	-	CODING	-1.39171	0.012791
ENSBTAT00000026836	ENSBTAG00000020148	TEK	8	17040335	17143857	-	CODING	-1.5003	0.012811
ENSBTAT00000015172	ENSBTAG00000011419	HSPA9	7	51506219	51521515	-	CODING	-1.00761	0.012824
ENSBTAT00000020662	ENSBTAG00000015551	-	4	1E+08	1E+08	-	CODING	-1.69547	0.012844
ENSBTAT00000061484	ENSBTAG00000002728	ARID1B	9	94882344	95271355	+	CODING	0.101179	0.012915
ENSBTAT00000018863	ENSBTAG00000014182	CTNNA1	7	51688098	51880519	+	CODING	-1.21589	0.01292
ENSBTAT00000004971	ENSBTAG00000003808	HDAC9	4	27174250	27457097	+	CODING	0.2498	0.01299
ENSBTAT00000008960	ENSBTAG00000006815	-	15	48250683	48251648	-	CODING	-1.42705	0.013005
ENSBTAT00000031972	ENSBTAG00000014960	SCHIP1	1	1.08E+08	1.08E+08	-	CODING	-0.60541	0.013006
ENSBTAT00000065657	ENSBTAG00000019781	CLASP1	2	73490617	73723487	-	CODING	-0.07913	0.013043
ENSBTAT00000037876	ENSBTAG00000026604	LYRM2	9	61434665	61436740	+	CODING	-1.591	0.013046
ENSBTAT00000012511	ENSBTAG00000009508	SMDT1	5	1.14E+08	1.14E+08	+	CODING	-1.07947	0.013202
ENSBTAT00000021534	ENSBTAG00000016177	FGL1	27	18454362	18472436	+	CODING	-1.6808	0.013217
ENSBTAT00000006979	ENSBTAG00000005299	EDNRB	12	53310604	53407024	-	CODING	-1.86691	0.013218
ENSBTAT00000014217	ENSBTAG00000010739	BLOC1S2	26	21043927	21053587	-	CODING	-1.31306	0.013244
ENSBTAT00000010745	ENSBTAG00000008173	TRIM13	12	19543053	19556013	+	CODING	-0.06017	0.013338
ENSBTAT00000005577	ENSBTAG00000004259	HPCAL1	11	87191215	87307998	-	CODING	-1.7132	0.013342
ENSBTAT00000010858	ENSBTAG00000008248	DMD	X	1.17E+08	1.18E+08	+	CODING	-0.20055	0.013386
ENSBTAT00000019496	ENSBTAG00000014642	NAPRT	14	2327870	2331019	+	CODING	-2.0146	0.013411
ENSBTAT00000018604	ENSBTAG00000013999	FAM20B	16	61810681	61852109	+	CODING	0.17108	0.013457
ENSBTAT00000057166	ENSBTAG00000040323	SECTM1	19	51104746	51149867	+	CODING	-1.91345	0.013654
ENSBTAT00000053888	ENSBTAG00000039708	C9orf172	11	1.06E+08	1.06E+08	-	CODING	0.048071	0.013664
ENSBTAT00000065946	ENSBTAG00000019269	COL6A2	1	1.48E+08	1.48E+08	+	CODING	-1.29496	0.013673
ENSBTAT00000008300	ENSBTAG00000006328	RBM6	22	50817696	50892474	-	CODING	-1.53544	0.013831
ENSBTAT00000011505	ENSBTAG00000008731	PRDX3	26	39672044	39681392	-	CODING	-0.61012	0.013909
ENSBTAT00000001255	ENSBTAG00000000950	MRPS16	28	29460098	29461928	-	CODING	-1.11704	0.013968
ENSBTAT00000002323	ENSBTAG00000001773	C7orf49	4	99713916	99719088	-	CODING	-0.0801	0.01398
ENSBTAT00000046448	ENSBTAG00000032705	CALM	20	8066341	8067501	+	CODING	-1.2307	0.014019
ENSBTAT00000001464	ENSBTAG00000001107	CACYBP	16	57694739	57705495	+	CODING	-1.07062	0.014059
ENSBTAT00000011585	ENSBTAG00000008792	RNASE6	10	26402507	26404000	-	CODING	-1.71141	0.01421
ENSBTAT00000045859	ENSBTAG00000000199	PDP1	14	72678594	72686964	-	CODING	-0.37164	0.014343

ENSBTAT00000026299	ENSBTAG00000019733	ELTD1	3	65921137	66062039	+	CODING	-1.69774	0.014353
ENSBTAT00000038058	ENSBTAG00000026684	SYNC	2	1.22E+08	1.22E+08	+	CODING	-0.13832	0.014398
ENSBTAT00000024621	ENSBTAG00000018498	PQBP1	X	92092355	92098095	+	CODING	-1.3277	0.014418
ENSBTAT00000062519	ENSBTAG00000045086	7SK	7	41115463	41115731	-	CODING	1.643796	0.014433
ENSBTAT00000011713	ENSBTAG00000008895	BPGM	4	99223160	99254538	+	CODING	-0.30394	0.014445
ENSBTAT00000004156	ENSBTAG00000003200	-	20	24233178	24292919	+	CODING	0.494928	0.014508
ENSBTAT00000011533	ENSBTAG00000008755	42801	2	36682216	36729906	-	CODING	-1.13739	0.014537
ENSBTAT00000034262	ENSBTAG00000024595	HECA	9	77893557	77907314	+	CODING	0.080823	0.014539
ENSBTAT00000065567	ENSBTAG00000026422	MPG	25	141582	146961	+	CODING	-2.07785	0.014577
ENSBTAT00000018066	ENSBTAG00000013586	CCR2	22	53613211	53614323	-	CODING	-4.53699	0.014578
ENSBTAT00000005655	ENSBTAG00000004318	ARL3	26	23563111	23761992	-	CODING	-1.54179	0.014715
ENSBTAT00000000718	ENSBTAG00000000548	KLHDC10	4	94676716	94737325	+	CODING	-0.31661	0.014734
ENSBTAT00000065849	ENSBTAG00000046004	-	X	1.01E+08	1.01E+08	-	CODING	-0.44645	0.014855
ENSBTAT00000022382	ENSBTAG00000010723	PFDN6	23	7405845	7407102	+	CODING	-1.18427	0.014876
ENSBTAT00000015760	ENSBTAG00000011873	KCNE3	15	54587990	54588289	-	CODING	-2.86348	0.014939
ENSBTAT00000035541	ENSBTAG00000025329	IRF2BPL	10	89245839	89248163	-	CODING	-0.04537	0.014944
ENSBTAT00000004597	ENSBTAG00000003535	DPM3	3	15517158	15517733	+	CODING	-1.4933	0.014961
ENSBTAT00000034273	ENSBTAG00000024603	NCOR2	17	53568327	53708353	+	CODING	-0.01483	0.014962
ENSBTAT00000049019	ENSBTAG00000034674	-	2	1.37E+08	1.37E+08	-	CODING	1.493906	0.014964
ENSBTAT00000028669	ENSBTAG00000021512	ZBTB7B	3	15634915	15648928	-	CODING	-0.28765	0.014966
ENSBTAT00000048248	ENSBTAG00000006370	CCT3	3	14538814	14553305	+	CODING	-1.48706	0.015008
ENSBTAT00000036271	ENSBTAG00000012632	TECR	7	12329820	12357348	-	CODING	-0.57829	0.015011
ENSBTAT00000028798	ENSBTAG00000030258	CDC42EP1	5	1.1E+08	1.1E+08	+	CODING	-1.3989	0.01508
ENSBTAT00000042894	ENSBTAG00000021781	MTFR1L	2	1.28E+08	1.28E+08	-	CODING	-0.33992	0.015096
ENSBTAT00000012512	ENSBTAG00000009509	NDUFA6	5	1.14E+08	1.14E+08	-	CODING	-1.0314	0.015252
ENSBTAT00000045673	ENSBTAG00000006819	-	21	49665666	49738270	+	CODING	-0.30743	0.015264
ENSBTAT00000011332	ENSBTAG00000008593	INCA1	19	27047777	27052238	+	CODING	-1.77102	0.01528
ENSBTAT00000024431	ENSBTAG00000018361	R3HDM2	5	56477490	56522167	+	CODING	-0.27446	0.015331
ENSBTAT00000017141	ENSBTAG00000012896	METTL7B	5	57899029	57901211	-	CODING	-2.18544	0.015364
ENSBTAT00000000017	ENSBTAG00000000013	PRKAA1	20	33688291	33716677	+	CODING	-0.32928	0.015449
ENSBTAT00000014525	ENSBTAG00000010937	APOO	X	1.26E+08	1.26E+08	+	CODING	-0.35068	0.01549
ENSBTAT00000015944	ENSBTAG00000012012	CYB5A	24	4413368	4445873	+	CODING	-1.21097	0.015508
ENSBTAT00000021521	ENSBTAG00000016169	ID1	13	61726125	61727283	+	CODING	-1.18941	0.015516
ENSBTAT00000022714	ENSBTAG00000017081	MIEF2	19	34984928	34986841	-	CODING	-0.03707	0.015542
ENSBTAT00000047156	ENSBTAG00000006884	PABPN1	10	21427968	21432160	-	CODING	-1.59072	0.015572
ENSBTAT00000000109	ENSBTAG00000000099	CERS2	3	19835017	19843638	+	CODING	-1.31452	0.015652
ENSBTAT00000018759	ENSBTAG00000014112	EXOC4	4	97791626	98594890	+	CODING	-0.1168	0.015671
ENSBTAT00000004227	ENSBTAG00000003263	TBCA	10	8737160	8822375	-	CODING	-1.31407	0.015738
ENSBTAT00000030227	ENSBTAG00000001059	IDH3G	X	39916807	39925370	-	CODING	-1.08391	0.015785
ENSBTAT00000027067	ENSBTAG00000020309	G3BP	7	64976048	65004834	+	CODING	-0.37023	0.015842
ENSBTAT00000014770	ENSBTAG00000011121	CLCN4	X	1.43E+08	1.43E+08	-	CODING	0.371066	0.015906
ENSBTAT00000055693	ENSBTAG00000040308	RPS13	15	35943265	35945982	+	CODING	-0.49386	0.015945
ENSBTAT00000060553	ENSBTAG00000043564	MT-ATP8	MT	8129	8329	+	CODING	-0.79962	0.015988
ENSBTAT00000065478	ENSBTAG00000047265	-	29	43163337	43163913	-	CODING	0.361346	0.016019
ENSBTAT00000009410	ENSBTAG00000007152	OS9	5	56070693	56104924	-	CODING	-1.21404	0.016048
ENSBTAT00000044128	ENSBTAG00000031171	SH3GLB1	3	57411164	57457439	-	CODING	-0.50301	0.016154
ENSBTAT00000007668	ENSBTAG00000005830	STRN4	18	54263458	54287817	-	CODING	-0.174	0.016275
ENSBTAT00000011029	ENSBTAG00000000874	CAST	7	98445837	98581253	+	CODING	-0.38693	0.016318
ENSBTAT00000021658	ENSBTAG00000016281	PREP	9	45169675	45301623	+	CODING	-0.47707	0.016337
ENSBTAT00000066239	ENSBTAG00000046959	CGGBP1	1	35655412	35660398	-	CODING	-0.27658	0.016386
ENSBTAT00000006020	ENSBTAG00000004586	-	X	65143672	65149272	+	CODING	-1.29497	0.016418
ENSBTAT00000008357	ENSBTAG00000006367	CTGF	9	70873221	70876455	-	CODING	-0.43372	0.016448
ENSBTAT00000057500	ENSBTAG00000039035	HSPA6	3	8027845	8029776	-	CODING	-2.76383	0.016491
ENSBTAT00000015064	ENSBTAG00000011337	ANKRD33B	20	62725184	62820226	-	CODING	0.091443	0.01657
ENSBTAT00000005333	ENSBTAG00000004082	-	6	71053202	71053458	-	CODING	0.607379	0.016628
ENSBTAT00000024367	ENSBTAG00000018312	-	1	1.04E+08	1.04E+08	+	CODING	-1.26896	0.016735
ENSBTAT00000008130	ENSBTAG00000006187	MFAP4	19	34685357	34687892	+	CODING	-1.64373	0.016757
ENSBTAT00000052383	ENSBTAG00000001478	PPPDE1	16	33290070	33331173	-	CODING	1.22856	0.016878
ENSBTAT00000020021	ENSBTAG00000015042	WDR55	7	53478710	53483614	+	CODING	-1.23283	0.01695
ENSBTAT00000050590	ENSBTAG00000006388	FBXO45	1	71710368	71721053	+	CODING	0.612812	0.016987
ENSBTAT00000012210	ENSBTAG00000009267	UHRF1BP1	23	8777063	8824585	+	CODING	0.012439	0.016987

ENSBTAT00000002967	ENSBTAG00000002302	CD59	15	65145073	65166597	-	CODING	-0.47363	0.016995
ENSBTAT00000024203	ENSBTAG00000018186	PDXK	1	1.46E+08	1.47E+08	+	CODING	-1.85288	0.016998
ENSBTAT00000029090	ENSBTAG00000014490	DDX39B	23	27565924	27576711	+	CODING	-1.09004	0.017003
ENSBTAT00000021532	ENSBTAG00000037640	PID1	2	1.18E+08	1.18E+08	-	CODING	-1.48546	0.017065
ENSBTAT00000016684	ENSBTAG00000012565	PCNX	10	82878051	83056528	+	CODING	-0.00317	0.017138
ENSBTAT00000049814	ENSBTAG00000035323	TMX4	13	534160	593323	-	CODING	-0.31428	0.017143
ENSBTAT00000024490	ENSBTAG00000018404	PRKG1	26	6906081	8343629	-	CODING	0.263887	0.017168
ENSBTAT00000062472	ENSBTAG00000045039	SCARNA17	24	49943458	49943600	+	CODING	0.056476	0.017254
ENSBTAT00000015332	ENSBTAG00000011540	SPG21	10	11853161	11873214	-	CODING	-1.4529	0.017256
ENSBTAT00000002554	ENSBTAG00000001968	TTC14	1	87007533	87017223	-	CODING	-1.52599	0.017261
ENSBTAT00000015624	ENSBTAG00000011763	RIOK2	7	99013511	99035256	-	CODING	-1.35192	0.017298
ENSBTAT00000024412	ENSBTAG00000018347	IL33	8	38724603	38792047	-	CODING	-1.54047	0.017343
ENSBTAT00000002286	ENSBTAG00000001741	DLGAP4	13	66204671	66292988	+	CODING	-1.28689	0.017346
ENSBTAT00000019814	ENSBTAG00000014883	GABARAP	19	27586886	27588815	-	CODING	-0.93553	0.017404
ENSBTAT00000030378	ENSBTAG00000000460	SYTL2	29	9806835	9927317	+	CODING	-2.07714	0.017452
ENSBTAT00000057380	ENSBTAG00000039023	ZNF664	17	53969695	53970480	-	CODING	-0.36343	0.017569
ENSBTAT00000014212	ENSBTAG00000010734	NOP16	7	39202866	39207621	-	CODING	-1.29076	0.017651
ENSBTAT00000024584	ENSBTAG00000018469	ALDH6A1	10	85821883	85839587	-	CODING	-0.22387	0.01769
ENSBTAT00000063162	ENSBTAG00000002688	ATP1B1	16	37568416	37592799	+	CODING	-1.03009	0.017774
ENSBTAT00000039933	ENSBTAG00000027713	LYSMD3	7	92440985	92446470	-	CODING	-0.00182	0.017777
ENSBTAT00000028608	ENSBTAG00000021461	PSMD3	19	40961497	40974702	+	CODING	-1.07972	0.01787
ENSBTAT00000023183	ENSBTAG00000017441	RPL27	19	43669374	43671655	+	CODING	-0.91769	0.017998
ENSBTAT00000021580	ENSBTAG00000032369	NMI	2	44930495	44950481	+	CODING	-2.24722	0.018044
ENSBTAT00000038842	ENSBTAG00000001514	ASB11	X	1.35E+08	1.35E+08	+	CODING	-0.52489	0.018056
ENSBTAT00000023581	ENSBTAG00000017733	CA2	14	79372712	79388600	-	CODING	-1.26596	0.018083
ENSBTAT00000034126	ENSBTAG00000024539	SPSB1	16	44994737	45006429	-	CODING	-1.68294	0.01817
ENSBTAT00000015010	ENSBTAG00000011298	EYA1	14	36898122	37268909	-	CODING	0.159365	0.018182
ENSBTAT00000023241	ENSBTAG00000017482	ISYNA1	7	4669453	4672648	+	CODING	-1.6869	0.018191
ENSBTAT00000019969	ENSBTAG00000015002	POLR2I	18	46961970	46963214	-	CODING	-1.17248	0.018227
ENSBTAT00000018514	ENSBTAG00000013935	NTPCR	28	5872192	5910468	+	CODING	-0.39778	0.018273
ENSBTAT00000020094	ENSBTAG00000015100	ATP6V0E1	20	4641618	4671634	+	CODING	-1.16946	0.018295
ENSBTAT00000018092	ENSBTAG00000013607	WDR1	6	1.1E+08	1.1E+08	-	CODING	-0.40076	0.018363
ENSBTAT00000006275	ENSBTAG00000004777	S100B	1	1.48E+08	1.48E+08	-	CODING	-1.06405	0.018417
ENSBTAT00000022780	ENSBTAG00000017139	STX7	9	71381757	71455585	-	CODING	-0.43188	0.018448
ENSBTAT00000019298	ENSBTAG00000014518	RPL9	6	60210361	60215120	-	CODING	-1.0611	0.018505
ENSBTAT00000002309	ENSBTAG00000001762	RFXANK	7	3986327	3991487	-	CODING	-2.30386	0.018584
ENSBTAT00000025580	ENSBTAG00000019214	USP14	24	35529296	35564664	+	CODING	-0.42265	0.018602
ENSBTAT00000001919	ENSBTAG00000001465	P2RY1	1	1.16E+08	1.16E+08	-	CODING	-0.06487	0.01869
ENSBTAT00000044234	ENSBTAG00000048296	TMEM27	X	1.35E+08	1.35E+08	+	CODING	-1.71209	0.018704
ENSBTAT00000002324	ENSBTAG00000001774	SPRY2	12	55684001	55685894	-	CODING	-1.41474	0.018718
ENSBTAT00000037186	ENSBTAG00000026232	TP53INP1	14	71743472	71748296	+	CODING	0.299784	0.018727
ENSBTAT00000025496	ENSBTAG00000019156	CCT2	5	44100435	44116239	-	CODING	-1.09994	0.018798
ENSBTAT00000065380	ENSBTAG00000047718	ZNF70	17	73195725	73197071	-	CODING	0.466575	0.01881
ENSBTAT00000004113	ENSBTAG00000003165	ADAMTS9	22	36876910	37037695	+	CODING	-2.14036	0.018833
ENSBTAT00000042662	ENSBTAG00000009131	TTC37	7	97172634	97262280	-	CODING	-0.06537	0.018836
ENSBTAT00000029212	ENSBTAG00000021912	DUSP7	22	49379908	49385352	+	CODING	-0.19799	0.018838
ENSBTAT00000012301	ENSBTAG00000009345	AMZ2	19	62506950	62514776	-	CODING	-0.53543	0.018938
ENSBTAT00000064569	ENSBTAG00000046750	B3GNT3	7	5271365	5291295	-	CODING	-1.56842	0.019046
ENSBTAT00000036123	ENSBTAG00000013362	DNM2	7	16465942	16523297	+	CODING	-0.34807	0.019158
ENSBTAT00000022472	ENSBTAG00000016896	HERPUD1	18	25048853	25059249	+	CODING	-1.05149	0.019158
ENSBTAT00000022078	ENSBTAG00000016596	PFDN1	7	53119765	53193428	-	CODING	-0.9853	0.019175
ENSBTAT00000019865	ENSBTAG00000014921	IL6	4	31578311	31582667	+	CODING	-3.79484	0.019256
ENSBTAT00000045065	ENSBTAG00000000215	GNB1	16	52106960	52184132	+	CODING	-1.14465	0.01944
ENSBTAT00000064941	ENSBTAG00000047490	-	2	99704435	99714269	+	CODING	1.331474	0.019502
ENSBTAT00000053663	ENSBTAG00000012890	SLC25A3	5	63086353	63092547	+	CODING	-1.4508	0.019532
ENSBTAT00000023209	ENSBTAG00000017461	SLC16A3	19	51243972	51255385	-	CODING	-1.43094	0.019552
ENSBTAT00000028361	ENSBTAG00000021287	SLC16A7	5	53987909	54214799	-	CODING	-2.37415	0.019719
ENSBTAT00000008569	ENSBTAG00000006523	SOD2	9	97399159	97404522	+	CODING	-0.564	0.019859
ENSBTAT00000064972	ENSBTAG00000046386	SERTM1	12	24926157	24926477	-	CODING	-2.56434	0.019934
ENSBTAT00000043997	ENSBTAG00000008429	LRP4	15	77663792	77701236	-	CODING	-1.81396	0.019994
ENSBTAT00000012101	ENSBTAG00000009183	SHISA5	22	51944064	51969644	+	CODING	-1.4436	0.020096

ENSBTAT00000064693	ENSBTAG00000046939	C9orf69	11	1.04E+08	1.04E+08	-	CODING	-0.16547	0.020105
ENSBTAT00000063425	ENSBTAG00000018513	FHL1	X	19777799	19820952	+	CODING	0.286043	0.020131
ENSBTAT00000056899	ENSBTAG00000026394	MGC142811	25	3744167	3749205	-	CODING	-0.43986	0.020138
ENSBTAT00000025681	ENSBTAG00000019285	FBXL17	7	1.1E+08	1.1E+08	-	CODING	-0.31075	0.020148
ENSBTAT00000006709	ENSBTAG00000005090	TMEM2	8	48223036	48303993	-	CODING	-0.30716	0.020149
ENSBTAT00000012341	ENSBTAG00000047926	OVCA2	19	23644915	23646308	+	CODING	-1.41176	0.020152
ENSBTAT00000020974	ENSBTAG00000015794	NES	3	14208425	14215064	+	CODING	-1.23692	0.020174
ENSBTAT00000009630	ENSBTAG00000007321	SREK1	20	13393479	13436297	-	CODING	-1.59374	0.020286
ENSBTAT00000005698	ENSBTAG00000004356	ROBO4	29	28719989	28734705	-	CODING	-1.47488	0.020287
ENSBTAT00000002893	ENSBTAG00000002236	TRPC4AP	13	64913710	64981620	-	CODING	-0.41613	0.0203
ENSBTAT00000033863	ENSBTAG00000013472	COL1A2	4	11624470	11661163	+	CODING	-0.43519	0.020378
ENSBTAT00000001662	ENSBTAG00000001257	AGTPBP1	8	80235083	80392851	-	CODING	-0.09662	0.020406
ENSBTAT00000003370	ENSBTAG00000002606	LGR4	15	58873843	58977292	-	CODING	-0.14619	0.020456
ENSBTAT00000013888	ENSBTAG00000010513	GPS2	19	27653444	27656067	-	CODING	-1.08392	0.020572
ENSBTAT00000011751	ENSBTAG00000008926	FUBP1	3	66969542	66993523	+	CODING	-1.31313	0.020677
ENSBTAT00000063903	ENSBTAG00000047229	CRIP1	21	71390600	71392110	-	CODING	-1.25821	0.020853
ENSBTAT00000063094	ENSBTAG00000047856	PAK2	1	71867089	71952257	+	CODING	-0.39747	0.020995
ENSBTAT00000030791	ENSBTAG00000022699	CAV3	22	17832937	17846997	-	CODING	-0.46202	0.021024
ENSBTAT00000003764	ENSBTAG00000002896	KPNA6	1	1.31E+08	1.31E+08	+	CODING	-0.45971	0.021035
ENSBTAT00000019230	ENSBTAG00000014463	CAMK2D	6	12965030	13271053	+	CODING	-0.19636	0.021104
ENSBTAT00000028045	ENSBTAG00000021059	TNMD	X	50951671	50973156	-	CODING	-3.22727	0.021137
ENSBTAT00000002626	ENSBTAG00000002026	RPL5	3	50787385	50795965	-	CODING	-1.06214	0.02114
ENSBTAT00000028355	ENSBTAG00000021282	SGCE	4	11840470	11911742	-	CODING	-1.72352	0.02116
ENSBTAT00000006213	ENSBTAG00000004732	SPTB	10	77201411	77273590	-	CODING	-0.24221	0.021355
ENSBTAT00000022847	ENSBTAG00000017191	-	13	44229670	44230470	+	CODING	-1.10702	0.021468
ENSBTAT00000003419	ENSBTAG00000002639	ZDHHC7	18	11116413	11133102	-	CODING	-0.32304	0.021596
ENSBTAT00000026366	ENSBTAG00000019787	PAFAH1B3	18	51308584	51311417	+	CODING	-1.83773	0.021652
ENSBTAT00000021337	ENSBTAG00000016032	-	11	44707028	44711889	-	CODING	-0.18354	0.021745
ENSBTAT00000018085	ENSBTAG00000013600	PSMG1	1	1.41E+08	1.41E+08	-	CODING	-1.22506	0.021746
ENSBTAT00000034949	ENSBTAG00000025028	MZT1	12	47768049	47781910	-	CODING	-0.09574	0.02176
ENSBTAT00000020211	ENSBTAG00000015192	FAM193B	7	40378360	40388704	-	CODING	-2.03652	0.021888
ENSBTAT00000054271	ENSBTAG00000009707	MYL1	2	98527573	98555234	-	CODING	-0.85083	0.021923
ENSBTAT00000064594	ENSBTAG00000045794	PL-5283	4	1E+08	1E+08	+	CODING	-0.516	0.021976
ENSBTAT00000004821	ENSBTAG00000003701	VASH2	16	72529550	72531856	-	CODING	1.997712	0.0221
ENSBTAT00000001061	ENSBTAG00000000802	LYVE1	15	42678184	42692464	+	CODING	-1.84838	0.022119
ENSBTAT00000006592	ENSBTAG00000005007	-	26	10251024	10251836	+	CODING	-1.77641	0.022215
ENSBTAT00000064804	ENSBTAG00000046122	NRARP	11	1.06E+08	1.06E+08	+	CODING	0.457078	0.022305
ENSBTAT00000056365	ENSBTAG00000013952	HNRPD	6	98916663	98933795	-	CODING	-1.07265	0.022324
ENSBTAT00000008971	ENSBTAG00000006824	APPL1	22	44397100	44427704	-	CODING	-0.32874	0.022383
ENSBTAT00000039622	ENSBTAG00000018479	PTPMT1	15	78571082	78576838	+	CODING	-1.462	0.022414
ENSBTAT00000034217	ENSBTAG00000024578	-	6	27328572	27329745	-	CODING	-1.46118	0.02254
ENSBTAT00000049126	ENSBTAG00000034689	ZNRF1	18	2396335	2482363	+	CODING	-0.44264	0.02263
ENSBTAT00000011504	ENSBTAG00000008732	ZC3H12C	15	20489130	20526349	+	CODING	-2.15258	0.022711
ENSBTAT00000011196	ENSBTAG00000008493	AQP3	8	76525369	76531334	-	CODING	-1.72334	0.02278
ENSBTAT00000013481	ENSBTAG00000010217	ZNF318	23	16901808	16928392	-	CODING	0.092465	0.022782
ENSBTAT00000018888	ENSBTAG00000014208	RPL35A	1	70788697	70792140	-	CODING	-1.01673	0.022809
ENSBTAT00000019438	ENSBTAG00000014726	VAMP3	16	46455183	46464710	-	CODING	-0.5057	0.022918
ENSBTAT00000034148	ENSBTAG00000021298	METTL11B	16	38568049	38586028	+	CODING	1.006989	0.022994
ENSBTAT00000010394	ENSBTAG00000040392	-	18	61206192	61215875	-	CODING	-1.83174	0.023098
ENSBTAT00000033371	ENSBTAG00000024182	H2B	23	30730958	30731338	+	CODING	1.339559	0.023144
ENSBTAT00000015161	ENSBTAG00000011406	TESK1	8	60207472	60212368	+	CODING	-0.40995	0.023195
ENSBTAT00000019656	ENSBTAG00000014775	GUK1	7	2895658	2905568	-	CODING	-1.39668	0.023294
ENSBTAT00000018774	ENSBTAG00000014127	PGHS-2	16	69263776	69271399	-	CODING	-2.00644	0.023302
ENSBTAT00000001296	ENSBTAG00000000979	SMIM19	27	37055278	37070241	+	CODING	-0.29944	0.023326
ENSBTAT00000023383	ENSBTAG00000017584	PLCG1	13	70510824	70524389	+	CODING	-1.57775	0.023335
ENSBTAT00000035582	ENSBTAG00000025358	SNX2	7	32329451	32388793	-	CODING	-1.33042	0.023349
ENSBTAT00000052954	ENSBTAG00000032007	LRP11	9	88115145	88183314	-	CODING	0.046565	0.023421
ENSBTAT00000007278	ENSBTAG00000005534	ENO3	19	27073498	27078655	-	CODING	-0.75598	0.023423
ENSBTAT00000021435	ENSBTAG00000001586	OTUD7B	3	20656593	20712080	+	CODING	-0.2224	0.023522
ENSBTAT00000045370	ENSBTAG00000014401	SORBS3	8	70357693	70384759	+	CODING	-1.28256	0.023552
ENSBTAT00000002274	ENSBTAG00000001736	UBN1	25	3936573	3964092	+	CODING	0.028252	0.02357

ENSBTAT00000002976	ENSBTAG00000002306	UTP11L	3	1.09E+08	1.09E+08	-	CODING	-0.43835	0.02361
ENSBTAT00000027514	ENSBTAG00000020646	ZC3H6	11	65466	111276	-	CODING	0.47721	0.02369
ENSBTAT00000009930	ENSBTAG00000007547	CACNG1	19	63738188	63745798	+	CODING	-0.54132	0.023721
ENSBTAT00000005370	ENSBTAG00000004110	-	X	84590185	84590760	-	CODING	1.161373	0.023759
ENSBTAT00000032589	ENSBTAG00000012519	XDH	11	14176298	14236380	-	CODING	-1.52905	0.023794
ENSBTAT00000015910	ENSBTAG00000011988	KIAA0100	19	20619020	20645017	-	CODING	-0.02591	0.023807
ENSBTAT00000017583	ENSBTAG00000013210	ADAMTS4	3	8322938	8331146	+	CODING	-2.09742	0.023816
ENSBTAT00000061493	ENSBTAG00000000288	UPF2	13	12363666	12452723	+	CODING	-0.31617	0.023823
ENSBTAT00000028279	ENSBTAG00000021225	DCAF5	10	81194836	81259609	-	CODING	-0.26356	0.023919
ENSBTAT00000045329	ENSBTAG00000006561	RASGEF1B	6	97988424	98026691	-	CODING	-1.93142	0.024031
ENSBTAT00000065504	ENSBTAG00000045981	DIRAS1	7	22195393	22195989	+	CODING	1.020271	0.024085
ENSBTAT00000004611	ENSBTAG00000003545	TAF1D	29	1056627	1062580	+	CODING	-1.54326	0.02413
ENSBTAT00000009990	ENSBTAG00000007595	BROX	16	26908537	26927548	+	CODING	-0.19569	0.024174
ENSBTAT00000047777	ENSBTAG00000033662	NCKAP1	2	13575102	13664827	+	CODING	-0.42444	0.024186
ENSBTAT00000013796	ENSBTAG00000010451	PA2G4	5	57532245	57538950	-	CODING	-1.12492	0.02419
ENSBTAT00000011265	ENSBTAG00000008545	ATF3	16	72820026	72832974	-	CODING	-1.06828	0.024202
ENSBTAT00000026019	ENSBTAG00000019526	CMTM6	22	7001971	7024065	-	CODING	-1.3146	0.024209
ENSBTAT00000063873	ENSBTAG00000046160	SMIM13	23	45020474	45041768	-	CODING	-0.10146	0.02421
ENSBTAT00000012136	ENSBTAG00000009211	UBE2S	18	62537351	62541236	+	CODING	-1.38152	0.024249
ENSBTAT00000000163	ENSBTAG00000000141	NFU1	11	67752716	67781632	-	CODING	-0.5052	0.024255
ENSBTAT00000055517	ENSBTAG00000016060	CREM	13	18496784	18527315	-	CODING	-1.76036	0.02434
ENSBTAT00000010834	ENSBTAG00000008236	OR51E2	15	50940888	50941850	+	CODING	0.457011	0.024398
ENSBTAT00000025533	ENSBTAG00000019184	TRAPPC2L	18	14079700	14083634	+	CODING	-1.34872	0.024434
ENSBTAT00000038321	ENSBTAG00000026819	HDAC7	5	32635940	32667499	+	CODING	-1.60236	0.024514
ENSBTAT00000022238	ENSBTAG00000016728	PLEKHG5	16	47719088	47751042	+	CODING	-2.36744	0.024517
ENSBTAT00000027794	ENSBTAG00000020861	CHAMP1	12	91100867	91103281	+	CODING	-0.24237	0.024552
ENSBTAT00000013703	ENSBTAG00000010383	YTHDF1	13	54805022	54817791	+	CODING	-0.48333	0.024588
ENSBTAT00000020099	ENSBTAG00000015104	CCDC28B	2	1.22E+08	1.22E+08	-	CODING	-1.83651	0.024629
ENSBTAT00000004885	ENSBTAG00000003752	SLC25A24	3	35152469	35210446	+	CODING	-1.71707	0.024655
ENSBTAT00000021243	ENSBTAG00000015969	STK40	3	1.1E+08	1.1E+08	+	CODING	-0.34656	0.02485
ENSBTAT00000048178	ENSBTAG00000017278	ACBD5	13	18047209	18075549	-	CODING	-0.29578	0.024866
ENSBTAT00000023705	ENSBTAG00000017830	RBMS2	5	57167137	57219971	-	CODING	-1.5384	0.024966
ENSBTAT00000061251	ENSBTAG00000039684	PTRF	19	43148013	43162165	-	CODING	-0.98578	0.025204
ENSBTAT00000015248	ENSBTAG00000011473	MYL9	13	66306260	66314230	+	CODING	-1.11258	0.025336
ENSBTAT00000006406	ENSBTAG00000004872	MRPL20	16	52383424	52386462	+	CODING	-1.16445	0.025401
ENSBTAT00000049576	ENSBTAG00000012242	MAF1	14	1921784	1924818	-	CODING	-0.41132	0.025467
ENSBTAT00000023255	ENSBTAG00000017496	ATP5I	6	1.09E+08	1.09E+08	-	CODING	-1.03613	0.025654
ENSBTAT00000016190	ENSBTAG00000012192	-	5	74997804	75003221	-	CODING	-1.92646	0.025664
ENSBTAT00000033967	ENSBTAG00000035226	TOR1AIP1	16	62533723	62577521	+	CODING	-0.18187	0.025703
ENSBTAT00000032460	ENSBTAG00000001852	BREH1	18	24827700	24854528	+	CODING	-1.571	0.025873
ENSBTAT00000038518	ENSBTAG00000016836	PDK1	2	24067475	24107467	-	CODING	-0.096	0.0259
ENSBTAT00000034054	ENSBTAG00000024503	FCER1G	3	8305544	8308776	-	CODING	-1.7001	0.025997
ENSBTAT00000018525	ENSBTAG00000013943	ZNF703	27	32683065	32685824	+	CODING	-0.14939	0.026069
ENSBTAT00000022993	ENSBTAG00000017298	AAMDC	29	18250502	18284062	-	CODING	-0.60807	0.02611
ENSBTAT00000045518	ENSBTAG00000032089	CDC42EP2	29	44189278	44195090	+	CODING	0.29686	0.026232
ENSBTAT00000012866	ENSBTAG00000009757	RPSA	22	12729703	12741653	+	CODING	-0.95214	0.026236
ENSBTAT00000032311	ENSBTAG00000019883	IST1	18	39300561	39339579	-	CODING	-1.15288	0.026252
ENSBTAT00000002587	ENSBTAG00000001996	SNRNP70	18	56058668	56073102	+	CODING	-1.37557	0.026382
ENSBTAT00000000519	ENSBTAG00000000405	VKORC1	25	27457370	27459608	-	CODING	-1.45563	0.026397
ENSBTAT00000006430	ENSBTAG00000004887	DCTN1	11	10235834	10253077	+	CODING	-0.4055	0.026467
ENSBTAT00000017366	ENSBTAG00000013060	IQGAP1	21	22530902	22614701	-	CODING	-1.29504	0.026523
ENSBTAT00000005780	ENSBTAG00000004406	MSI2	19	8651763	8720657	+	CODING	-0.09844	0.026709
ENSBTAT00000007571	ENSBTAG00000005757	FUS	25	27523969	27533988	+	CODING	-1.05564	0.026789
ENSBTAT00000009496	ENSBTAG00000007217	KIAA0195	19	56679702	56697953	-	CODING	-0.23177	0.026851
ENSBTAT00000039197	ENSBTAG00000006775	SLC12A7	20	71395142	71420351	+	CODING	-1.91126	0.026905
ENSBTAT00000048868	ENSBTAG00000034496	SHFM1	22	22098224	22098436	-	CODING	-0.88468	0.02698
ENSBTAT00000064827	ENSBTAG00000046019	SDHA	20	71940749	71964107	-	CODING	-0.63967	0.026981
ENSBTAT00000018383	ENSBTAG00000013843	ACVRL1	5	28097837	28106733	-	CODING	-1.52702	0.027183
ENSBTAT00000006653	ENSBTAG00000005043	TIMP1	X	91232235	91236073	+	CODING	-1.43379	0.027198
ENSBTAT00000055067	ENSBTAG00000037942	-	13	74496558	74498471	-	CODING	-0.06484	0.027223
ENSBTAT00000026726	ENSBTAG00000020061	KCNJ12	19	35955796	35991013	+	CODING	-0.30563	0.027277

ENSBTAT00000049158	ENSBTAG00000013750	B3GALNT2	28	8232079	8297594	-	CODING	-1.41415	0.027281
ENSBTAT00000061383	ENSBTAG00000013616	AP4E1	10	59578464	59644086	-	CODING	-0.06174	0.027372
ENSBTAT00000019643	ENSBTAG00000014764	CD9	5	1.05E+08	1.05E+08	-	CODING	-1.18783	0.027509
ENSBTAT00000005684	ENSBTAG00000004344	ACSL1	27	14223449	14288333	-	CODING	-0.64705	0.027526
ENSBTAT00000008419	ENSBTAG00000006420	BMPRII	2	91395347	91461311	+	CODING	-0.32472	0.027529
ENSBTAT00000001346	ENSBTAG00000001017	SLK	26	24786360	24844579	+	CODING	-0.10829	0.027566
ENSBTAT00000061003	ENSBTAG00000021581	FHOD3	24	20649326	21172169	-	CODING	-0.10406	0.027575
ENSBTAT00000019566	ENSBTAG00000014700	DYNC1LI1	22	7045916	7098962	-	CODING	-0.32792	0.027635
ENSBTAT00000000198	ENSBTAG00000000172	PFKFB1	X	97738494	97888649	-	CODING	-1.47246	0.027762
ENSBTAT00000000316	ENSBTAG00000000252	POLE3	8	1.04E+08	1.04E+08	-	CODING	-0.50944	0.027796
ENSBTAT00000007419	ENSBTAG00000023259	OSBPL1A	24	32680524	32897080	+	CODING	-0.20403	0.027796
ENSBTAT00000014735	ENSBTAG00000011100	CTSC	29	7433482	7473315	+	CODING	-1.41745	0.027936
ENSBTAT00000063143	ENSBTAG00000047029	-	11	47050711	47051142	-	CODING	-0.52446	0.027991
ENSBTAT00000061641	ENSBTAG00000043956	UBE2E3	2	15671752	15761822	-	CODING	-0.43697	0.028004
ENSBTAT00000024092	ENSBTAG00000018101	AHCY	13	64258560	64274274	-	CODING	-0.56718	0.028098
ENSBTAT00000017987	ENSBTAG00000013528	CHMP7	8	71228581	71251787	+	CODING	-0.33529	0.028136
ENSBTAT00000061158	ENSBTAG00000010786	TACC2	26	42323743	42485654	+	CODING	-0.45447	0.028204
ENSBTAT00000017563	ENSBTAG00000013191	AGRN	16	52674207	52712338	-	CODING	-0.10334	0.028225
ENSBTAT00000031231	ENSBTAG00000022989	FAM174B	21	14690052	14729722	+	CODING	0.104206	0.028325
ENSBTAT00000061457	ENSBTAG00000012582	IARS2	16	24285717	24329372	+	CODING	-0.54526	0.028338
ENSBTAT00000002600	ENSBTAG00000002006	THBS1	10	35314025	35329297	+	CODING	-1.25857	0.028402
ENSBTAT00000013117	ENSBTAG00000009942	PLCL2	1	1.56E+08	1.56E+08	+	CODING	-0.40287	0.028414
ENSBTAT00000025572	ENSBTAG00000019211	BRIX1	20	39256327	39265218	-	CODING	-1.47356	0.028424
ENSBTAT00000025174	ENSBTAG00000018918	NDUFAF3	22	51540981	51542102	-	CODING	-1.17446	0.028506
ENSBTAT00000026753	ENSBTAG00000020079	MAP3K4	9	98162838	98232658	+	CODING	-0.23752	0.028523
ENSBTAT00000010206	ENSBTAG00000007763	SLC1A4	11	63337655	63367589	+	CODING	0.438023	0.028536
ENSBTAT00000039322	ENSBTAG00000027397	PPP1R3D	13	57120666	57121565	+	CODING	0.721232	0.0286
ENSBTAT00000064932	ENSBTAG00000039593	FAM98C	18	48476852	48479868	+	CODING	-3.62647	0.028607
ENSBTAT00000020427	ENSBTAG00000015369	MLLT11	3	19762896	19767967	-	CODING	-0.27733	0.028644
ENSBTAT00000028718	ENSBTAG00000021554	NOL7	23	42795776	42800011	-	CODING	-1.12928	0.028653
ENSBTAT00000046599	ENSBTAG00000032829	GHITM	28	39435546	39448963	+	CODING	-0.61848	0.02868
ENSBTAT00000031376	ENSBTAG00000023073	FAM89A	28	3656713	3676337	+	CODING	-1.92178	0.028681
ENSBTAT00000025716	ENSBTAG00000019314	USP25	1	20664990	20798853	-	CODING	-0.5941	0.028727
ENSBTAT00000002697	ENSBTAG00000002083	AUTS2	25	30045721	30076258	-	CODING	-0.05098	0.028751
ENSBTAT00000020351	ENSBTAG00000015303	MPP6	4	71625584	71690219	-	CODING	-0.0786	0.028756
ENSBTAT00000046975	ENSBTAG00000012094	SCAMP3	3	15432100	15436418	+	CODING	-0.52657	0.02878
ENSBTAT00000026766	ENSBTAG00000020089	-	19	12986898	13084710	+	CODING	-0.13619	0.028986
ENSBTAT00000035885	ENSBTAG00000021134	DPP9	7	20648283	20678569	+	CODING	-0.26137	0.028997
ENSBTAT00000024417	ENSBTAG00000018352	ABRA	14	59974386	59985200	+	CODING	-1.10464	0.029029
ENSBTAT00000020593	ENSBTAG00000015467	FAM184A	9	32425508	32497610	+	CODING	0.420107	0.029036
ENSBTAT00000006510	ENSBTAG00000004950	BRN	10	26288223	26289852	+	CODING	-1.40364	0.029082
ENSBTAT00000001384	ENSBTAG00000001042	MXD1	11	68232862	68255606	+	CODING	0.026841	0.029143
ENSBTAT00000005277	ENSBTAG00000004036	GJC1	19	45154679	45186611	-	CODING	-1.97082	0.029156
ENSBTAT00000011617	ENSBTAG00000001632	NBR1	19	43785335	43812243	+	CODING	-0.40773	0.029219
ENSBTAT00000011172	ENSBTAG00000022204	-	X	57664213	57664899	+	CODING	-1.18781	0.029291
ENSBTAT00000005134	ENSBTAG00000003934	PSPC1	12	36529631	36579763	+	CODING	-1.49318	0.029292
ENSBTAT00000050220	ENSBTAG00000035735	BIRC2	15	6585516	6607013	-	CODING	0.186816	0.029321
ENSBTAT00000009992	ENSBTAG00000007596	GEM	14	72386751	72399660	+	CODING	-1.73172	0.029407
ENSBTAT00000010788	ENSBTAG00000008203	SEPW1	18	55112228	55116590	+	CODING	-0.94775	0.029457
ENSBTAT00000039626	ENSBTAG00000001348	COMMD8	6	67746952	67761215	-	CODING	-0.24498	0.029501
ENSBTAT00000005375	ENSBTAG00000004112	GAMT	7	45433859	45436865	-	CODING	-1.11134	0.029514
ENSBTAT00000063552	ENSBTAG00000046672	ZDHHC18	2	1.27E+08	1.27E+08	-	CODING	-0.11593	0.029583
ENSBTAT00000009444	ENSBTAG00000007177	EMP2	25	9330475	9359612	-	CODING	-0.4403	0.029706
ENSBTAT00000019214	ENSBTAG00000014449	-	1	84033060	84033889	+	CODING	-1.99389	0.029752
ENSBTAT00000048781	ENSBTAG00000010945	SEC24B	6	16978876	17058815	-	CODING	-0.30361	0.029773
ENSBTAT00000015599	ENSBTAG00000011748	SGMS1	26	8735912	8775858	-	CODING	-1.36023	0.029855
ENSBTAT00000020877	ENSBTAG00000015727	IFI47	7	41823772	41840054	-	CODING	-1.53607	0.02989
ENSBTAT00000001948	ENSBTAG00000001489	TUBA1A	5	30821251	30825700	+	CODING	-1.39493	0.030109
ENSBTAT00000027246	ENSBTAG00000020446	THOP1	7	22128058	22141914	-	CODING	-1.32634	0.030233
ENSBTAT00000034094	ENSBTAG00000020014	CEP104	16	50498425	50533080	+	CODING	-0.10061	0.030336
ENSBTAT00000009159	ENSBTAG00000022128	-	X	38597970	38599160	+	CODING	-0.55326	0.030375

ENSBTAT00000013035	ENSBTAG00000009886	KDELR3	5	1.11E+08	1.11E+08	+	CODING	-1.43433	0.030489
ENSBTAT00000005230	ENSBTAG00000004005	WDR7	24	56467338	56817379	+	CODING	-0.14449	0.030586
ENSBTAT00000000795	ENSBTAG00000000603	JAM2	1	10083035	10165613	-	CODING	-1.27038	0.030616
ENSBTAT00000028113	ENSBTAG00000021103	SLC35F5	2	65677515	65719366	-	CODING	-0.24763	0.03067
ENSBTAT00000004985	ENSBTAG00000003825	PTPN12	4	43834354	43884954	-	CODING	-1.42049	0.030696
ENSBTAT00000012534	ENSBTAG00000009526	TMEM59	3	92746059	92771330	+	CODING	-1.18942	0.030838
ENSBTAT00000000738	ENSBTAG00000000565	RFX7	10	54281616	54422091	+	CODING	-0.13595	0.030883
ENSBTAT00000005174	ENSBTAG00000003967	OTUB1	29	42882593	42889928	+	CODING	-1.24892	0.031196
ENSBTAT00000019872	ENSBTAG00000014927	OXSR1	22	11674127	11758890	+	CODING	-0.18181	0.031248
ENSBTAT00000007711	ENSBTAG00000005868	HEBP1	5	97348105	97385488	+	CODING	-1.62336	0.031326
ENSBTAT00000047536	ENSBTAG00000033315	DNAJC1	13	23251449	23428980	-	CODING	-1.65642	0.031412
ENSBTAT00000039536	ENSBTAG00000027513	-	6	90695494	90697557	+	CODING	-4.00081	0.03147
ENSBTAT00000064557	ENSBTAG00000045954	LRRC14B	20	71984463	71989365	-	CODING	-0.04012	0.031487
ENSBTAT00000064131	ENSBTAG00000047717	FAM222B	19	20734088	20786818	-	CODING	-0.1935	0.031509
ENSBTAT00000033154	ENSBTAG00000013290	DYSF	11	12894979	13123270	-	CODING	-0.36372	0.031525
ENSBTAT00000002914	ENSBTAG00000002258	APOA1	15	27932200	27934085	-	CODING	-2.18722	0.031529
ENSBTAT00000016319	ENSBTAG00000012299	REPS1	9	77666178	77757440	-	CODING	-0.23365	0.031565
ENSBTAT00000020229	ENSBTAG00000015204	SMPX	X	1.29E+08	1.29E+08	+	CODING	-0.69249	0.031599
ENSBTAT00000003297	ENSBTAG00000002539	TRIOBP	5	1.1E+08	1.1E+08	+	CODING	-1.53362	0.031693
ENSBTAT00000029271	ENSBTAG00000021955	NPC2	10	86170653	86179237	-	CODING	-1.17059	0.031714
ENSBTAT00000062037	ENSBTAG00000044604	snoU89	10	36862418	36862608	+	CODING	-3.89927	0.031758
ENSBTAT00000013685	ENSBTAG00000010368	TPST2	17	68445522	68499477	-	CODING	-1.40335	0.031841
ENSBTAT00000014449	ENSBTAG00000010877	ARMC12	23	9683947	9694015	+	CODING	-1.63646	0.03186
ENSBTAT00000019336	ENSBTAG00000014547	PGAM2	22	370972	373326	-	CODING	-0.73759	0.031905
ENSBTAT00000036417	ENSBTAG00000008645	ESRRA	29	43221839	43228308	+	CODING	-0.51674	0.031924
ENSBTAT00000016718	ENSBTAG00000012595	HRSP12	14	68412747	68420944	+	CODING	-1.48952	0.03204
ENSBTAT00000013781	ENSBTAG00000010439	AKAP8L	7	8754284	8787049	+	CODING	-1.23381	0.032176
ENSBTAT00000028765	ENSBTAG00000021587	SMPDL3A	9	28811578	28828968	-	CODING	-1.33096	0.032179
ENSBTAT00000003484	ENSBTAG00000002690	BLZF1	16	37838604	37860891	+	CODING	-0.43058	0.032296
ENSBTAT00000018795	ENSBTAG00000014140	ATP1B3	1	1.28E+08	1.28E+08	-	CODING	-1.51081	0.032326
ENSBTAT00000023325	ENSBTAG00000017547	DBNL	22	357512	370121	+	CODING	-1.5128	0.032344
ENSBTAT00000012594	ENSBTAG00000009576	PACSIN3	15	78266386	78275225	-	CODING	-1.00463	0.032347
ENSBTAT00000064335	ENSBTAG00000017770	-	4	1.04E+08	1.04E+08	+	CODING	-1.36363	0.032488
ENSBTAT00000013887	ENSBTAG00000010515	FBXW5	11	1.06E+08	1.06E+08	+	CODING	-0.4672	0.032521
ENSBTAT00000005179	ENSBTAG00000003970	MITD1	11	4290569	4306454	-	CODING	-1.76053	0.032581
ENSBTAT00000042645	ENSBTAG00000030209	ARHGDIA	19	51633694	51637708	+	CODING	-1.11129	0.032597
ENSBTAT00000027212	ENSBTAG00000020420	AP1S2	X	1.35E+08	1.35E+08	+	CODING	-1.63619	0.032695
ENSBTAT00000008177	ENSBTAG00000006227	IDH3A	21	31063699	31079936	+	CODING	-1.03621	0.0327
ENSBTAT00000065805	ENSBTAG00000047293	NRIP1	1	21693828	21697298	+	CODING	-0.21413	0.032727
ENSBTAT00000009208	ENSBTAG00000006995	SPTBN1	11	37030009	37241384	+	CODING	-1.041	0.032755
ENSBTAT00000008096	ENSBTAG00000006155	CCRL2	22	53567797	53569696	-	CODING	-1.75899	0.032779
ENSBTAT00000005416	ENSBTAG00000004139	BACH1	1	6157625	6174155	-	CODING	-1.53379	0.032783
ENSBTAT00000019627	ENSBTAG00000014750	EPB41L4A	10	1467376	1761414	+	CODING	-2.12916	0.032832
ENSBTAT00000021830	ENSBTAG00000016413	DUSP26	27	28963365	28969954	-	CODING	-0.9838	0.032833
ENSBTAT00000048411	ENSBTAG00000015512	-	20	6721327	6753670	-	CODING	-1.84062	0.032834
ENSBTAT00000056663	ENSBTAG00000011766	C7	20	33549495	33606517	-	CODING	-0.08473	0.032844
ENSBTAT00000025350	ENSBTAG00000019044	BAIAP2	19	52198617	52263263	-	CODING	-1.89474	0.032907
ENSBTAT00000017435	ENSBTAG00000038540	-	26	25060238	25073503	+	CODING	-0.21961	0.032931
ENSBTAT00000010613	ENSBTAG00000008068	ERLEC1	11	36371087	36395686	+	CODING	-0.28627	0.032954
ENSBTAT00000027942	ENSBTAG00000020981	42799	26	13831827	13865056	+	CODING	-0.37309	0.033028
ENSBTAT00000064819	ENSBTAG00000047975	DOK5	13	82696138	82803966	+	CODING	-0.33043	0.033065
ENSBTAT00000065813	ENSBTAG00000045523	-	6	35008595	35010340	+	CODING	-0.45491	0.033153
ENSBTAT00000028220	ENSBTAG00000021174	NRD1	3	95041110	95122769	+	CODING	-0.43502	0.033191
ENSBTAT00000008635	ENSBTAG00000006574	ARL8A	16	70894434	70900751	-	CODING	-0.21791	0.033337
ENSBTAT00000061102	ENSBTAG00000027064	BTBD11	5	70923456	71257389	+	CODING	0.2069	0.033355
ENSBTAT00000018691	ENSBTAG00000014068	VDAC3	27	36923762	36932879	+	CODING	-0.61936	0.033495
ENSBTAT00000013855	ENSBTAG00000010487	MGC128424	7	64133812	64153713	+	CODING	-1.821	0.033635
ENSBTAT00000025973	ENSBTAG00000006287	NEDD9	23	44851677	44988905	+	CODING	-1.59259	0.033723
ENSBTAT00000033229	ENSBTAG00000034586	-	11	1821486	1846701	+	CODING	-1.58758	0.033762
ENSBTAT00000045136	ENSBTAG00000014295	CAPZA1	3	30810109	30854190	-	CODING	-1.19882	0.033773
ENSBTAT00000004070	ENSBTAG00000003130	CHRNA3	21	31500106	31512927	-	CODING	0.506263	0.033836

ENSBTAT00000008976	ENSBTAG00000006828	RAPGEF3	5	32697065	32718624 +	CODING	-1.74819	0.033924
ENSBTAT00000043065	ENSBTAG00000002853	HRC	18	56107442	56111943 -	CODING	-0.61742	0.033962
ENSBTAT00000003653	ENSBTAG00000002823	MPZL1	3	930876	1012734 -	CODING	-1.34907	0.033964
ENSBTAT00000010044	ENSBTAG00000007634	HOOK3	27	37278344	37358417 +	CODING	-0.28128	0.03397
ENSBTAT00000039415	ENSBTAG00000027446	RSU1	13	31347074	31554426 -	CODING	-1.28785	0.034008
ENSBTAT00000011385	ENSBTAG00000008634	WBP2	19	56394188	56401350 +	CODING	-0.51725	0.034109
ENSBTAT00000043069	ENSBTAG00000019294	ABCC9	5	88677586	88831324 +	CODING	-0.39158	0.034191
ENSBTAT00000003304	ENSBTAG00000002550	-	6	62506125	62506524 -	CODING	-2.44071	0.034196
ENSBTAT00000005628	ENSBTAG00000004295	NDUFA8	11	93011815	93029730 -	CODING	-1.00674	0.034276
ENSBTAT00000000669	ENSBTAG00000000516	OTOR	13	10760673	10763286 +	CODING	-3.67693	0.034373
ENSBTAT00000028260	ENSBTAG00000021208	RRM2B	14	64315460	64352488 +	CODING	0.174311	0.034722
ENSBTAT00000022559	ENSBTAG00000016959	LAPTM4B	14	68668786	68730708 -	CODING	-1.15623	0.034757
ENSBTAT00000023284	ENSBTAG00000017512	MAPT	19	46524375	46643750 +	CODING	0.260152	0.034785
ENSBTAT00000019137	ENSBTAG00000014390	MTMR9	2	1.22E+08	1.22E+08 +	CODING	-1.92417	0.034858
ENSBTAT00000030067	ENSBTAG00000022255	AR	X	88410764	88621166 -	CODING	0.65992	0.035058
ENSBTAT00000007341	ENSBTAG00000005589	STK19	23	27197602	27206845 -	CODING	-1.74259	0.035171
ENSBTAT00000008200	ENSBTAG00000006247	DHPS	7	13931315	13935225 +	CODING	-0.3869	0.035194
ENSBTAT00000000596	ENSBTAG00000000469	PPP2CA	7	47425980	47450747 -	CODING	-1.0006	0.035252
ENSBTAT00000022319	ENSBTAG00000016779	CLIP1	17	55245237	55360147 +	CODING	-0.58462	0.035262
ENSBTAT00000029796	ENSBTAG00000015611	TBC1D9B	7	1273635	1313897 +	CODING	-0.26685	0.035317
ENSBTAT00000027348	ENSBTAG00000020527	IDH1	2	96941049	96962496 -	CODING	-1.27038	0.035364
ENSBTAT00000007704	ENSBTAG00000005863	-	5	26580498	26671702 +	CODING	-0.17629	0.035392
ENSBTAT00000014537	ENSBTAG00000010948	CLIC2	X	38498587	38523525 +	CODING	-1.72707	0.035441
ENSBTAT00000012852	ENSBTAG00000009747	RNFT1	19	11112589	11123128 -	CODING	-2.39442	0.035441
ENSBTAT00000026332	ENSBTAG00000019759	IDE	26	13920375	14012061 -	CODING	-0.34137	0.035653
ENSBTAT00000004358	ENSBTAG00000003359	ELOVL5	23	25155743	25228997 -	CODING	-1.77476	0.035729
ENSBTAT00000022580	ENSBTAG00000016979	PCYT1A	1	71431933	71484412 -	CODING	-0.18521	0.035889
ENSBTAT00000024515	ENSBTAG00000018425	PSMD7	18	37108019	37115996 -	CODING	-0.98438	0.035904
ENSBTAT00000025800	ENSBTAG00000019366	POLR2B	6	74027219	74071144 +	CODING	-0.47742	0.036178
ENSBTAT00000002142	ENSBTAG00000001635	CUTA	23	7534224	7535613 -	CODING	-0.32155	0.036381
ENSBTAT00000008363	ENSBTAG00000006374	NHP2	7	40601549	40605473 -	CODING	-1.11943	0.036415
ENSBTAT00000023483	ENSBTAG00000017660	-	X	91260328	91261416 +	CODING	-0.14403	0.036611
ENSBTAT00000012632	ENSBTAG00000009599	LCN1	11	1.06E+08	1.06E+08 +	CODING	-3.99285	0.03663
ENSBTAT00000006594	ENSBTAG00000005009	PRELID1	7	40132356	40135909 +	CODING	-1.44933	0.036667
ENSBTAT00000044565	ENSBTAG00000005980	RREB1	23	47900595	47959504 -	CODING	0.036989	0.036677
ENSBTAT00000017663	ENSBTAG00000013282	NECAP2	2	1.36E+08	1.36E+08 -	CODING	-1.55919	0.03671
ENSBTAT00000012316	ENSBTAG00000009358	MTSS1L	18	1531382	1545901 +	CODING	-1.90679	0.036717
ENSBTAT00000018833	ENSBTAG00000014171	NAPEPLD	4	44665683	44720623 -	CODING	-0.05386	0.036762
ENSBTAT00000063991	ENSBTAG00000011824	OGN	8	85453019	85468721 +	CODING	-3.0142	0.03679
ENSBTAT00000064959	ENSBTAG00000036183	FBXW8	17	60338777	60396323 -	CODING	-2.2316	0.036811
ENSBTAT00000024333	ENSBTAG00000018285	VAMP5	11	49249763	49258304 -	CODING	-1.38263	0.037048
ENSBTAT00000010790	ENSBTAG00000008204	C9orf41	8	51344227	51383813 -	CODING	-0.21601	0.037057
ENSBTAT00000057531	ENSBTAG00000047866	-	19	48447145	48447444 -	CODING	-3.22299	0.037106
ENSBTAT00000007438	ENSBTAG00000005661	SNAP23	10	37946231	37986849 +	CODING	-1.19887	0.037159
ENSBTAT00000002520	ENSBTAG00000001936	PCK1	13	59144594	59150719 -	CODING	-2.62378	0.037306
ENSBTAT00000017860	ENSBTAG00000013423	GLRX5	21	61986090	61996011 +	CODING	-1.09792	0.03733
ENSBTAT00000003250	ENSBTAG00000002501	CUEDC2	26	22919818	22921523 -	CODING	-1.23231	0.037352
ENSBTAT00000008092	ENSBTAG00000006152	-	4	84628122	84628559 -	CODING	-0.38991	0.037398
ENSBTAT00000044261	ENSBTAG00000046337	TUBB6	24	43249500	43250564 +	CODING	-1.27797	0.037848
ENSBTAT00000005916	ENSBTAG00000025210	COL4A2	12	89112423	89165255 +	CODING	-1.2171	0.038015
ENSBTAT00000028751	ENSBTAG00000021577	ZFYVE16	7	82875305	82908670 +	CODING	-0.20899	0.038091
ENSBTAT00000025144	ENSBTAG00000018889	ATP6V0B	3	1.03E+08	1.03E+08 -	CODING	-1.26552	0.038153
ENSBTAT00000061343	ENSBTAG00000013271	NCOR1	19	33945404	34043863 +	CODING	-0.24581	0.038171
ENSBTAT00000023529	ENSBTAG00000017690	CARNS1	29	45957524	45964322 +	CODING	-0.10249	0.038233
ENSBTAT00000023743	ENSBTAG00000017863	SRGN	28	25631917	25647735 +	CODING	-1.41776	0.038259
ENSBTAT00000009785	ENSBTAG00000007441	SEC23IP	26	40346540	40380689 +	CODING	-0.32935	0.038346
ENSBTAT00000062993	ENSBTAG00000046346	PACS2	21	71291846	71316837 +	CODING	-0.10038	0.03846
ENSBTAT00000016117	ENSBTAG00000012149	HOXC8	5	26180271	26182515 -	CODING	-0.43145	0.03848
ENSBTAT00000053650	ENSBTAG00000038156	DYNLT3	X	1.11E+08	1.11E+08 +	CODING	-1.29761	0.038567
ENSBTAT00000008411	ENSBTAG00000006416	EMC4	10	28441593	28445103 -	CODING	-1.08562	0.038595
ENSBTAT00000021155	ENSBTAG00000015909	PDE8A	21	23119315	23262118 +	CODING	-0.45904	0.038653

ENSBTAT00000032051	ENSBTAG00000001055	HNRNPL	18	48823774	48835620	-	CODING	-1.03552	0.038699
ENSBTAT00000043366	ENSBTAG00000030674	AK4	3	80491324	80582219	-	CODING	-0.24982	0.038728
ENSBTAT00000009482	ENSBTAG00000007208	HDAC11	22	59089634	59103701	-	CODING	-0.05155	0.038781
ENSBTAT00000049233	ENSBTAG00000034785	DNAJC15	12	13183734	13266310	+	CODING	-1.51872	0.03883
ENSBTAT00000044870	ENSBTAG00000031648	C18H19orf33	18	48337453	48338289	+	CODING	-2.5849	0.038879
ENSBTAT00000028708	ENSBTAG00000021544	EBNA1BP2	3	1.03E+08	1.03E+08	+	CODING	-1.23492	0.038885
ENSBTAT00000010797	ENSBTAG00000008213	ATMIN	18	7755291	7768469	+	CODING	-0.30367	0.039113
ENSBTAT00000018091	ENSBTAG00000013606	FKBP2	29	43159739	43162644	+	CODING	-1.27497	0.039244
ENSBTAT00000013697	ENSBTAG00000010373	CACTIN	7	21539960	21551276	+	CODING	-1.53898	0.039292
ENSBTAT00000026033	ENSBTAG00000019538	FBXO28	16	28009798	28033718	+	CODING	-0.21034	0.039363
ENSBTAT00000064356	ENSBTAG00000048237	FGF9	12	35630411	35647612	-	CODING	0.341393	0.039389
ENSBTAT00000004402	ENSBTAG00000003396	MAFB	13	70068210	70069181	-	CODING	0.002944	0.039443
ENSBTAT00000009307	ENSBTAG00000007080	PREB	11	72493261	72497358	+	CODING	-1.18717	0.03956
ENSBTAT00000018762	ENSBTAG00000014105	SRPR	29	29986076	29991236	-	CODING	-0.54481	0.039602
ENSBTAT00000016518	ENSBTAG00000012448	CCDC91	5	81582317	81992463	-	CODING	-0.37726	0.039633
ENSBTAT00000007505	ENSBTAG00000005715	FUOM	26	25872737	25875735	+	CODING	-1.54102	0.039651
ENSBTAT00000018977	ENSBTAG00000014284	ALPK2	24	58119582	58248068	-	CODING	-0.17334	0.039663
ENSBTAT00000064839	ENSBTAG00000045822	-	15	74915626	74916613	+	CODING	-1.66029	0.039665
ENSBTAT00000032674	ENSBTAG00000021306	CHRDL2	15	54785596	54819153	-	CODING	-2.26148	0.039911
ENSBTAT00000015674	ENSBTAG00000011808	MSTN	2	6213566	6220196	+	CODING	0.255215	0.039977
ENSBTAT00000021501	ENSBTAG00000016152	DAB2	20	35018908	35079162	+	CODING	-1.54091	0.040033
ENSBTAT00000013500	ENSBTAG00000010227	CPSF2	21	57395846	57429002	+	CODING	-0.26473	0.040054
ENSBTAT00000053151	ENSBTAG00000010851	SEPHS2	25	26867626	26868981	-	CODING	-1.31171	0.040095
ENSBTAT00000012174	ENSBTAG00000009238	HOXC6	5	26161892	26163475	-	CODING	-0.43942	0.040138
ENSBTAT00000006569	ENSBTAG00000004990	NEO1	10	19795806	19959384	+	CODING	-0.18528	0.040183
ENSBTAT00000002992	ENSBTAG00000002321	AMT	22	51257611	51261937	-	CODING	-1.74768	0.040193
ENSBTAT00000055430	ENSBTAG00000001323	CENPC1	6	84905005	84998863	-	CODING	0.38866	0.040313
ENSBTAT00000022798	ENSBTAG00000027080	SLC8A3	10	82128379	82273227	-	CODING	0.087813	0.040344
ENSBTAT00000008102	ENSBTAG00000006161	C-MET	4	51912652	52042198	-	CODING	-0.19594	0.040408
ENSBTAT00000006412	ENSBTAG00000004876	RABL2B	5	1.2E+08	1.2E+08	+	CODING	-1.50222	0.040489
ENSBTAT00000064741	ENSBTAG00000018996	PARK2	9	98421510	99411209	-	CODING	1.018768	0.040532
ENSBTAT00000065868	ENSBTAG00000018227	SLC4A7	22	1746286	1877841	-	CODING	-1.76853	0.04058
ENSBTAT00000032505	ENSBTAG00000023736	CDC42EP3	11	20109202	20110829	-	CODING	-0.25448	0.040601
ENSBTAT00000002597	ENSBTAG00000002004	LLPH	5	47901612	47905608	+	CODING	-1.16579	0.040777
ENSBTAT00000000733	ENSBTAG00000000562	TOMM20	28	7926960	7941655	-	CODING	-0.5829	0.040797
ENSBTAT00000006039	ENSBTAG00000004602	PITX1	7	48063871	48069063	-	CODING	-3.037	0.04121
ENSBTAT00000023068	ENSBTAG00000017352	ZC3H11A	16	1404592	1440704	+	CODING	-0.51257	0.041352
ENSBTAT00000034656	ENSBTAG00000024889	HSBP1	18	10172302	10175455	+	CODING	-1.00912	0.041426
ENSBTAT00000002495	ENSBTAG00000001919	ZNF652	19	37919482	37932791	+	CODING	0.034915	0.041598
ENSBTAT00000032362	ENSBTAG00000005604	POPDC2	1	65002248	65014687	-	CODING	0.047311	0.041681
ENSBTAT00000024295	ENSBTAG00000018252	ARRDC4	21	8924763	8939028	-	CODING	-1.33678	0.041704
ENSBTAT00000024559	ENSBTAG00000018453	ANKRD37	27	14706021	14708632	+	CODING	0.632014	0.041775
ENSBTAT00000015208	ENSBTAG00000011444	HINT2	8	60390945	60393499	-	CODING	-1.33055	0.041809
ENSBTAT00000065501	ENSBTAG00000045937	-	4	17767925	17769016	+	CODING	-1.03437	0.041835
ENSBTAT00000013093	ENSBTAG00000009923	KIAA1737	10	89365866	89381540	+	CODING	-1.0847	0.041904
ENSBTAT00000011977	ENSBTAG00000009085	SLC35A5	1	57786942	57807544	+	CODING	-1.37387	0.041973
ENSBTAT00000019808	ENSBTAG00000014878	COX7A1	18	46994991	46996544	-	CODING	-0.92587	0.042031
ENSBTAT00000027054	ENSBTAG00000020299	OPN1LW	X	40234826	40247095	+	CODING	-5.49895	0.042114
ENSBTAT00000031316	ENSBTAG00000012667	CAMK2G	28	29878540	29930559	-	CODING	-0.1978	0.042136
ENSBTAT00000000087	ENSBTAG00000000079	CCSAP	28	281480	297049	-	CODING	-7.34875	0.042212
ENSBTAT00000000359	ENSBTAG00000000286	PFKM	5	32312957	32337525	-	CODING	-0.88812	0.042214
ENSBTAT00000028453	ENSBTAG00000021343	ARHGEF12	15	31451015	31614554	+	CODING	-0.15893	0.042276
ENSBTAT00000013805	ENSBTAG00000010457	NUAK1	5	69816616	69892512	-	CODING	0.185725	0.042557
ENSBTAT00000005011	ENSBTAG00000003842	RABGEF1	25	28509094	28534880	+	CODING	-0.39251	0.042573
ENSBTAT00000057114	ENSBTAG00000038034	ZNF628	18	62475322	62481823	-	CODING	0.268546	0.042692
ENSBTAT00000000049	ENSBTAG00000000044	MYADML2	19	51562314	51565450	+	CODING	-0.39445	0.042725
ENSBTAT00000066030	ENSBTAG00000046545	MEF2D	3	14380072	14413740	+	CODING	-0.55832	0.04276
ENSBTAT00000029340	ENSBTAG00000039995	CFH	16	6052925	6122550	+	CODING	-1.29861	0.042854
ENSBTAT00000013382	ENSBTAG00000010138	SEMA3B	22	50655648	50661989	-	CODING	-1.90522	0.042908
ENSBTAT00000010806	ENSBTAG00000008218	NPTN	10	20207483	20270508	-	CODING	-1.06613	0.042909
ENSBTAT00000013990	ENSBTAG00000010587	SH3BGRL	X	70399116	70527059	-	CODING	-1.26647	0.042929

ENSBTAT00000009126	ENSBTAG00000006951	LMO2	15	65394511	65407118	-	CODING	-1.32766	0.042989
ENSBTAT00000036062	ENSBTAG00000012384	TFEB	23	15507488	15514052	-	CODING	-0.28675	0.043006
ENSBTAT00000018722	ENSBTAG00000014083	AMIGO1	3	34054795	34060289	+	CODING	-0.07011	0.043217
ENSBTAT00000004574	ENSBTAG00000003519	NOL3	18	34928332	34931932	+	CODING	-1.07312	0.043242
ENSBTAT00000049723	ENSBTAG00000035230	TOR1AIP2	16	62493958	62528817	-	CODING	-0.07557	0.043311
ENSBTAT00000012872	ENSBTAG00000009761	ACTR2	11	63552997	63591980	+	CODING	-1.12154	0.043331
ENSBTAT00000063852	ENSBTAG00000047880	-	2	31688429	31699171	+	CODING	-1.9335	0.043693
ENSBTAT00000005930	ENSBTAG00000004514	RAF1	22	57122412	57204951	+	CODING	-0.53833	0.043746
ENSBTAT00000003291	ENSBTAG00000027182	NR3C2	17	9742222	10173282	+	CODING	0.030692	0.044033
ENSBTAT00000036489	ENSBTAG00000025803	C15orf59	10	20379755	20392155	-	CODING	-0.27785	0.044069
ENSBTAT00000014951	ENSBTAG00000011256	MYO1B	2	80166217	80369680	+	CODING	-1.45679	0.044164
ENSBTAT00000001868	ENSBTAG00000001425	FBXO11	11	29989995	30101580	-	CODING	-0.41315	0.044254
ENSBTAT00000032902	ENSBTAG00000002261	LBX1	26	21894402	21896298	-	CODING	0.250472	0.044816
ENSBTAT00000008751	ENSBTAG00000006663	CYTH1	19	54240689	54261902	+	CODING	-1.38403	0.044869
ENSBTAT00000003274	ENSBTAG00000002520	CUGBP1	15	78490888	78570865	-	CODING	-0.36581	0.044976
ENSBTAT00000005481	ENSBTAG00000032517	BCL7A	17	55551316	55573152	-	CODING	0.248886	0.044997
ENSBTAT00000010492	ENSBTAG00000007981	GRK5	26	39702550	39930993	+	CODING	-1.71645	0.045007
ENSBTAT00000002174	ENSBTAG00000007361	EHD1	29	43704113	43708218	-	CODING	-1.14258	0.045036
ENSBTAT00000056243	ENSBTAG00000040602	-	2	1.31E+08	1.31E+08	-	CODING	-1.0078	0.045038
ENSBTAT00000057477	ENSBTAG00000001030	MTMR3	17	71122533	71253675	+	CODING	-0.26306	0.045066
ENSBTAT00000025978	ENSBTAG00000019501	NUDT15	12	17960583	17967118	+	CODING	-0.19639	0.045115
ENSBTAT00000000027	ENSBTAG00000000025	-	15	53936927	54027857	-	CODING	-0.54556	0.045408
ENSBTAT00000003955	ENSBTAG00000003039	PSMB8	23	7113304	7116676	-	CODING	-1.38628	0.045607
ENSBTAT00000016972	ENSBTAG00000045658	FAM92A1	15	43705886	43706823	+	CODING	-1.59991	0.045702
ENSBTAT00000026674	ENSBTAG00000020026	-	3	16358535	16366943	+	CODING	-0.54094	0.045717
ENSBTAT00000023976	ENSBTAG00000018013	EMP3	18	55482512	55486850	+	CODING	-1.29541	0.045738
ENSBTAT00000016754	ENSBTAG00000012620	C2ORF17	2	1.08E+08	1.08E+08	+	CODING	-0.48605	0.045764
ENSBTAT00000012587	ENSBTAG00000009569	DOCK6	7	16851754	16898876	-	CODING	-1.62894	0.045913
ENSBTAT00000056220	ENSBTAG00000007731	-	14	10191721	10264168	-	CODING	-0.05852	0.045969
ENSBTAT00000005534	ENSBTAG00000004225	TSR1	19	23877562	23886659	-	CODING	-0.36807	0.046116
ENSBTAT00000001208	ENSBTAG00000000913	UQCR10	17	71038498	71040693	+	CODING	-0.70473	0.046171
ENSBTAT00000000573	ENSBTAG00000000448	BDH1	1	72572941	72608810	-	CODING	-1.74944	0.04625
ENSBTAT00000007399	ENSBTAG00000005628	CD52	2	1.27E+08	1.27E+08	-	CODING	-1.90065	0.046305
ENSBTAT00000017303	ENSBTAG00000013016	GNAI3	3	33969546	34013930	-	CODING	-1.35457	0.046587
ENSBTAT00000007108	ENSBTAG00000005397	CD46	16	77483017	77522635	-	CODING	-1.11694	0.046681
ENSBTAT00000046260	ENSBTAG00000011488	PRPF8	19	23324762	23357323	-	CODING	-0.48768	0.046716
ENSBTAT00000026319	ENSBTAG00000019750	TMEM106B	4	19893089	19915667	+	CODING	-0.39334	0.046744
ENSBTAT00000018887	ENSBTAG00000014207	ADAMTS10	7	18391616	18408987	-	CODING	-2.30871	0.046761
ENSBTAT00000020668	ENSBTAG00000024526	-	27	22570243	22576884	+	CODING	-1.7862	0.046813
ENSBTAT00000023681	ENSBTAG00000017812	ALS2CL	22	53358573	53380720	+	CODING	-1.72605	0.046816
ENSBTAT00000008304	ENSBTAG00000006325	NUDCD3	4	77598538	77670087	+	CODING	-0.4376	0.046889
ENSBTAT00000038116	ENSBTAG00000026716	IBTK	9	22263095	22343685	-	CODING	-0.23357	0.046899
ENSBTAT00000031588	ENSBTAG00000017108	OTUD3	2	1.33E+08	1.33E+08	-	CODING	-0.33223	0.047249
ENSBTAT00000001518	ENSBTAG00000001137	CLTA	8	60849478	60867215	+	CODING	-1.24092	0.047411
ENSBTAT00000056728	ENSBTAG00000000492	ASNSD1	2	6605444	6616484	-	CODING	-0.47267	0.047586
ENSBTAT00000015668	ENSBTAG00000011802	COL6A1	1	1.47E+08	1.47E+08	+	CODING	-1.07479	0.047721
ENSBTAT00000009379	ENSBTAG00000007123	ENSA	3	20141694	20146997	+	CODING	-0.44144	0.047734
ENSBTAT00000030958	ENSBTAG00000008696	FAM120A	8	86200482	86316789	+	CODING	-0.29493	0.047744
ENSBTAT00000000112	ENSBTAG00000000103	MRPL23	29	50182529	50187374	-	CODING	-2.62602	0.047784
ENSBTAT00000028639	ENSBTAG00000021487	CIART	3	20400969	20404292	-	CODING	-1.11287	0.047853
ENSBTAT00000053960	ENSBTAG00000026882	-	2	30091989	30092690	+	CODING	-1.45177	0.047896
ENSBTAT00000022205	ENSBTAG00000016703	CDC73	16	12643618	12733800	-	CODING	-0.41477	0.047932
ENSBTAT00000017944	ENSBTAG00000013491	EMAPL	21	66358950	66556801	+	CODING	0.059802	0.048007
ENSBTAT00000006683	ENSBTAG00000005069	TM7SF2	29	43981731	43986407	+	CODING	0.10089	0.048132
ENSBTAT00000017239	ENSBTAG00000012966	SCRN3	2	22400874	22432815	-	CODING	-0.18185	0.04816
ENSBTAT00000005998	ENSBTAG00000004564	MBNL1	1	1.16E+08	1.16E+08	-	CODING	-0.62524	0.048326
ENSBTAT00000009923	ENSBTAG00000007540	GLUD1	28	41941042	41979591	-	CODING	-0.5012	0.048364
ENSBTAT00000032260	ENSBTAG00000002394	TMEM44	1	73516016	73559858	+	CODING	-2.10674	0.048397
ENSBTAT00000002428	ENSBTAG00000010255	ZNF24	24	22025794	22031048	+	CODING	-0.35249	0.048502
ENSBTAT00000028884	ENSBTAG00000021675	PJA2	7	1.11E+08	1.11E+08	-	CODING	-0.30674	0.048639
ENSBTAT00000065251	ENSBTAG00000043985	DACH1	12	46417100	46430892	-	CODING	1.760131	0.048712

ENSBTAT00000001071	ENSBTAG00000000810	COPE	7	4224033	4242168	+	CODING	-1.22266	0.048785
ENSBTAT00000002506	ENSBTAG00000001926	RAD23B	8	98681864	98726100	+	CODING	-0.68471	0.048801
ENSBTAT00000045869	ENSBTAG00000039731	RND3	2	45800466	45822011	+	CODING	-1.48778	0.048822
ENSBTAT00000010692	ENSBTAG00000008132	SOX13	16	1716803	1728817	+	CODING	-1.77694	0.049013
ENSBTAT00000021751	ENSBTAG00000016350	NACC1	7	13554143	13559771	-	CODING	-0.21721	0.049131
ENSBTAT00000063138	ENSBTAG00000046956	STYX	10	11349899	11399972	-	CODING	-0.13449	0.049182
ENSBTAT00000009083	ENSBTAG00000006909	PIK3CB	1	1.31E+08	1.32E+08	+	CODING	0.191253	0.049183
ENSBTAT00000013464	ENSBTAG00000010204	PCMT1	9	88068718	88101563	+	CODING	-0.57211	0.049212
ENSBTAT00000003111	ENSBTAG00000002404	TMX2	15	82363844	82371822	+	CODING	0.173602	0.049213
ENSBTAT00000036474	ENSBTAG00000018796	RABGGTA	10	20726808	20733197	+	CODING	-7.59028	0.049287
ENSBTAT00000010192	ENSBTAG00000035254	CYHR1	14	1663923	1665533	+	CODING	1.394785	0.049491
ENSBTAT00000001760	ENSBTAG00000001333	PPARG	22	57366997	57489570	-	CODING	-2.01289	0.049531
ENSBTAT00000023664	ENSBTAG00000017798	ERH	10	81521060	81533819	-	CODING	-1.24886	0.049539
ENSBTAT00000063236	ENSBTAG00000046156	SIAT4B	18	1748745	1760788	+	CODING	-0.20899	0.049609
ENSBTAT00000010928	ENSBTAG00000008303	FKBP1A	13	60276502	60303755	+	CODING	-0.98886	0.049637
ENSBTAT00000026409	ENSBTAG00000019821	DHX9	16	65379390	65420803	+	CODING	-1.19336	0.049672
ENSBTAT00000046161	ENSBTAG00000032534	RHOF	17	55759606	55773011	+	CODING	-1.23546	0.049764
ENSBTAT00000035362	ENSBTAG00000027980	TAF9	20	10300612	10304139	+	CODING	-1.14876	0.049908
ENSBTAT00000023668	ENSBTAG00000017801	ATP6AP2	X	1.08E+08	1.08E+08	-	CODING	-1.24621	0.049939
ENSBTAT00000005016	ENSBTAG00000003845	CSRNP1	22	12520393	12532806	-	CODING	-1.39044	0.049943
ENSBTAT00000044457	ENSBTAG00000003556	ZFYVE21	21	69978023	69980890	+	CODING	-1.54799	0.049952

Steer

GeneNames	GeneAcc	GeneName	Chr	Start	End	Strand	Type	log2(Fold_	p-value
ENSBTAT00000060569	ENSBTAG00000043561	COX1	MT	5687	7231	+	CODING	-0.87314	0
ENSBTAT00000012797	ENSBTAG00000007090	MYH2	19	30137767	30165109	-	CODING	-1.9614	0
ENSBTAT00000048981	ENSBTAG00000034580	TMSB4	GJ05	35911	36045	+	CODING	1.570912	0
ENSBTAT00000059374	ENSBTAG00000042382	U6	28	41535977	41536083	+	CODING	2.05846	0
ENSBTAT00000060543	ENSBTAG00000043559	MT-ND4L	MT	10239	10535	+	CODING	-0.44854	0
ENSBTAT00000059949	ENSBTAG00000042957	U6	5	71251611	71251717	-	CODING	-12.5462	0
ENSBTAT00000062950	ENSBTAG00000045757	TNNC1	22	48988984	48991875	+	CODING	0.485601	0
ENSBTAT00000063025	ENSBTAG00000047231	TNNI1	16	49293852	49303508	-	CODING	1.171459	0
ENSBTAT00000024444	ENSBTAG00000018369	MYL2	17	56953813	56961603	-	CODING	0.50868	0
ENSBTAT00000037753	ENSBTAG00000014731	GAPDH	5	104237902	104241979	-	CODING	-0.5612	0
ENSBTAT00000060571	ENSBTAG00000043558	ND1	MT	3101	4056	+	CODING	-0.3264	0
ENSBTAT00000044397	ENSBTAG00000011969	HSPB1	25	34858435	34861074	+	CODING	0.890859	0
ENSBTAT00000006532	ENSBTAG00000046332	ACTA1	28	419154	421910	-	CODING	-0.28894	5.26E-303
ENSBTAT00000022375	ENSBTAG00000016819	FABP3	2	122723225	122783830	+	CODING	1.522975	7.32E-298
ENSBTAT00000008593	ENSBTAG00000006541	ATP2A1	25	26188434	26204655	-	CODING	-0.96896	3.24E-292
ENSBTAT00000063726	ENSBTAG00000046001	U6	19	43534127	43534233	-	CODING	12.71216	1.19E-287
ENSBTAT00000059974	ENSBTAG00000042982	U6	26	42369695	42369801	-	CODING	12.71216	1.19E-287
ENSBTAT00000015186	ENSBTAG00000011424	TPM2	8	60268618	60274720	-	CODING	0.299782	1.49E-263
ENSBTAT00000060175	ENSBTAG00000043183	U6	6	115682606	115682712	-	CODING	12.47069	7.54E-255
ENSBTAT00000064903	ENSBTAG00000006419	TNNT1	18	62725898	62735263	+	CODING	1.264489	7.56E-251
ENSBTAT00000017177	ENSBTAG00000012927	ALDOA	25	26470488	26475202	-	CODING	-0.37956	6.33E-247
ENSBTAT00000018799	ENSBTAG00000014143	ASB5	27	6692314	6736965	-	CODING	1.256427	7.50E-230
ENSBTAT00000001373	ENSBTAG00000001032	PYGM	29	43606012	43617848	-	CODING	-0.85258	1.07E-218
ENSBTAT00000060562	ENSBTAG00000043546	MT-ND6	MT	13913	14440	-	CODING	-0.31906	2.59E-210
ENSBTAT00000060553	ENSBTAG00000043564	MT-ATP8	MT	8129	8329	+	CODING	0.091644	1.13E-204
ENSBTAT00000059121	ENSBTAG00000042129	U6	2	128245274	128245380	-	CODING	11.99012	6.68E-200
ENSBTAT00000013778	ENSBTAG00000010880	TNNI2	29	50285049	50287648	-	CODING	0.840633	4.84E-198
ENSBTAT00000007429	ENSBTAG00000005654	TMSB10	11	49933204	49934214	-	CODING	1.370326	7.87E-194
ENSBTAT00000043649	ENSBTAG00000011392	MYBPC1	5	65737956	65840833	+	CODING	-0.78167	9.75E-181
ENSBTAT00000059165	ENSBTAG00000042173	U6	15	54044311	54044418	-	CODING	-10.9338	6.44E-177
ENSBTAT00000008420	ENSBTAG00000006419	TNNT1	18	62725898	62735263	+	CODING	0.674226	2.66E-176
ENSBTAT00000065799	ENSBTAG00000048229	TPT1	25	1394201	1417716	-	CODING	0.405769	8.40E-175
ENSBTAT00000046662	ENSBTAG00000013264	RPS24	28	33926497	33931777	+	CODING	0.796212	1.33E-171
ENSBTAT00000044205	ENSBTAG00000031217	MYL6B	5	57489469	57492173	-	CODING	0.887404	2.26E-170
ENSBTAT00000028269	ENSBTAG00000021218	MYLPF	25	26815266	26817727	+	CODING	-0.27891	3.80E-157
ENSBTAT00000060549	ENSBTAG00000043556	COII	MT	7374	8057	+	CODING	0.151748	1.10E-155
ENSBTAT00000015579	ENSBTAG00000011730	TCAP	19	40691138	40692346	+	CODING	0.526471	2.00E-148
ENSBTAT00000026932	ENSBTAG00000020223	CASQ1	3	9591852	9601502	-	CODING	-0.68955	1.12E-146
ENSBTAT00000026756	ENSBTAG00000020080	MYBPC2	18	57039094	57064720	+	CODING	-0.79341	1.54E-140
ENSBTAT00000029890	ENSBTAG00000022158	TNNT3	29	50218484	50233948	-	CODING	0.588492	1.29E-138
ENSBTAT00000008371	ENSBTAG00000022158	TNNT3	29	50218484	50233948	-	CODING	-0.49454	3.08E-135
ENSBTAT00000008967	ENSBTAG00000006823	CMYA5	10	10658029	10711354	+	CODING	-1.26775	2.90E-132
ENSBTAT00000060540	ENSBTAG00000043570	-	MT	1453	3023	+	CODING	0.639709	5.69E-132
ENSBTAT00000065038	ENSBTAG00000046725	TNNC2	13	75316423	75318650	-	CODING	-0.19613	3.50E-131
ENSBTAT00000006781	ENSBTAG00000005142	RPL37	20	33667205	33669805	+	CODING	0.749852	3.42E-122
ENSBTAT00000000556	ENSBTAG00000000434	CRYAB	15	22566929	22570256	-	CODING	0.288658	9.52E-120
ENSBTAT00000065039	ENSBTAG00000046623	-	13	64372559	64372993	-	CODING	0.840223	1.35E-118
ENSBTAT00000011047	ENSBTAG00000008394	MYL3	22	53202766	53208551	+	CODING	-1.09019	1.61E-116
ENSBTAT00000030030	ENSBTAG00000022244	ACTN3	29	45230682	45242282	+	CODING	-0.90436	2.35E-116
ENSBTAT00000018491	ENSBTAG00000013924	RPS11	18	56404237	56406668	+	CODING	0.743401	2.01E-113
ENSBTAT00000009327	ENSBTAG00000018204	MYH1	19	30110728	30134757	-	CODING	-0.56341	9.40E-112
ENSBTAT00000043425	ENSBTAG00000019011	PGM1	3	82250292	82315999	-	CODING	-0.94903	1.34E-110
ENSBTAT00000034373	ENSBTAG00000023659	MT2	18	24125366	24126333	-	CODING	2.280162	3.83E-110
ENSBTAT00000060539	ENSBTAG00000043584	ATP6	MT	8290	8970	+	CODING	0.104707	2.55E-107
ENSBTAT00000007014	ENSBTAG00000005333	MB	5	74170471	74181260	-	CODING	0.177957	3.24E-104
ENSBTAT00000004190	ENSBTAG00000003229	RPL23	19	40075000	40079360	-	CODING	0.800776	1.11E-103
ENSBTAT00000061306	ENSBTAG00000009703	MYH7	10	21325414	21345624	+	CODING	-0.57094	5.44E-101
ENSBTAT00000027850	ENSBTAG00000020905	RPL11	2	129791372	129795563	-	CODING	1.072514	9.61E-101
ENSBTAT00000023600	ENSBTAG00000017743	XIRP2	2	29080500	29131828	-	CODING	-1.33267	1.68E-100
ENSBTAT00000015883	ENSBTAG00000011969	HSPB1	25	34858438	34861035	+	CODING	-7.50033	4.53E-96
ENSBTAT00000060552	ENSBTAG00000043577	MT-ND4	MT	10529	11906	+	CODING	-0.16305	2.39E-93
ENSBTAT00000016414	ENSBTAG00000012370	MGP	5	95456444	95459983	+	CODING	1.877408	1.79E-92

ENSBTAT00000027713	ENSBTAG00000020795	RPS21	13	55358720	55359979	-	CODING	1.069117	9.19E-90
ENSBTAT00000026259	ENSBTAG00000019701	RPL31	11	6003175	6008528	+	CODING	0.624017	2.52E-89
ENSBTAT00000020323	ENSBTAG00000015283	RPL32	22	56985249	56989012	+	CODING	0.761107	1.14E-88
ENSBTAT00000025803	ENSBTAG00000019368	IGFBP7	6	74071067	74150456	-	CODING	1.397691	1.49E-87
ENSBTAT00000034732	ENSBTAG00000024929	PPP1R27	19	51658342	51659508	+	CODING	0.644508	4.98E-86
ENSBTAT00000020326	ENSBTAG00000015285	RPS8	3	101816844	101818956	-	CODING	0.774571	8.02E-86
ENSBTAT00000016359	ENSBTAG00000012330	B2M	10	104139090	104145312	+	CODING	0.599313	7.12E-85
ENSBTAT00000002642	ENSBTAG00000002038	RPL14	22	13336573	13339983	+	CODING	0.810413	1.04E-84
ENSBTAT00000060577	ENSBTAG00000043563	ND5	MT	12109	13929	+	CODING	-0.27668	1.81E-79
ENSBTAT00000000901	ENSBTAG00000000678	CSDE1	3	28695271	28723718	+	CODING	-0.8347	6.97E-79
ENSBTAT00000038879	ENSBTAG00000014069	PDK4	4	12754202	12767677	-	CODING	0.806627	7.52E-79
ENSBTAT00000037900	ENSBTAG00000005085	TRIM63	2	127615050	127630608	+	CODING	1.237628	1.85E-78
ENSBTAT00000001791	ENSBTAG00000001360	RPS12	9	71974860	71978200	+	CODING	0.662485	5.18E-78
ENSBTAT00000022189	ENSBTAG00000016688	-	X	66722399	66723397	-	CODING	-0.55201	1.70E-77
ENSBTAT00000060547	ENSBTAG00000043568	MT-ND3	MT	9823	10168	+	CODING	0.096116	2.44E-77
ENSBTAT00000013077	ENSBTAG00000033217	TPM3	3	16376436	16395088	+	CODING	0.437084	1.04E-76
ENSBTAT00000008518	ENSBTAG00000006491	AGL	3	43504601	43585149	-	CODING	-1.54743	8.87E-75
ENSBTAT00000061284	ENSBTAG00000001564	PDE4DIP	3	22916551	23063522	-	CODING	-1.061	1.53E-74
ENSBTAT00000019758	ENSBTAG00000014835	SPARC	7	64878194	64900828	-	CODING	0.954178	5.38E-73
ENSBTAT00000046158	ENSBTAG00000032531	MUSTN1	22	48611898	48613043	+	CODING	0.547504	1.67E-72
ENSBTAT00000006990	ENSBTAG00000005315	RPS6	2	111820296	111821112	-	CODING	0.942027	2.43E-72
ENSBTAT00000065403	ENSBTAG00000018707	LDB3	28	41657195	41713873	+	CODING	-0.8802	7.76E-70
ENSBTAT00000065632	ENSBTAG00000046512	CMYA1	22	12549676	12558895	-	CODING	1.732506	4.48E-69
ENSBTAT00000060546	ENSBTAG00000043567	-	MT	431	1385	+	CODING	0.481049	3.02E-67
ENSBTAT00000046421	ENSBTAG00000032719	TFRC	1	71260068	71280648	-	CODING	-2.42198	3.93E-67
ENSBTAT00000047341	ENSBTAG00000033304	C27H8orf4	27	34893819	34895119	+	CODING	1.299996	1.17E-66
ENSBTAT00000015606	ENSBTAG00000011752	SYNM	21	7768429	7793372	-	CODING	-1.66511	1.23E-66
ENSBTAT00000043764	ENSBTAG00000002094	ATP5J2	25	37494182	37499276	+	CODING	0.703572	1.43E-66
ENSBTAT00000055075	ENSBTAG00000011765	GABARAPL1	5	100205186	100215034	-	CODING	1.7437	6.46E-66
ENSBTAT00000007278	ENSBTAG00000005534	ENO3	19	27073498	27078655	-	CODING	-0.39266	9.68E-66
ENSBTAT00000009469	ENSBTAG00000007196	TAGLN	15	28318411	28323666	+	CODING	1.218262	2.20E-64
ENSBTAT00000008373	ENSBTAG00000006383	-	X	126048949	126049743	+	CODING	1.144865	3.24E-63
ENSBTAT00000024572	ENSBTAG00000018463	VIM	13	31945012	31952941	+	CODING	1.029266	6.23E-63
ENSBTAT00000003943	ENSBTAG00000003033	GADD45G	8	90017453	90018996	-	CODING	2.162388	2.77E-61
ENSBTAT00000008434	ENSBTAG00000006434	SYNPO2	6	7388728	7590933	-	CODING	-1.29157	1.09E-59
ENSBTAT00000004562	ENSBTAG00000003505	DCN	5	21080013	21119087	-	CODING	1.114598	2.31E-59
ENSBTAT00000037243	ENSBTAG00000020035	RCAN1	1	351708	362907	+	CODING	1.425124	5.39E-59
ENSBTAT00000028662	ENSBTAG00000021508	LMOD3	22	32534126	32550296	+	CODING	-0.82512	2.81E-58
ENSBTAT00000003330	ENSBTAG00000002574	MYOZ2	6	7251253	7289661	-	CODING	0.622254	8.98E-58
ENSBTAT00000009302	ENSBTAG00000007415	SLC7A8	10	21521555	21573888	+	CODING	1.294072	1.20E-57
ENSBTAT00000017500	ENSBTAG00000025441	HSPA1A	23	27331773	27333698	-	CODING	0.669701	2.63E-56
ENSBTAT00000005580	ENSBTAG00000000425	RPL17	21	55609537	55610119	+	CODING	0.715504	2.63E-56
ENSBTAT00000030320	ENSBTAG00000011869	CSRP3	29	25994182	26014859	+	CODING	0.37997	2.01E-55
ENSBTAT00000008851	ENSBTAG00000006733	PPP1R3A	4	54866421	54906096	+	CODING	-0.99592	6.30E-55
ENSBTAT00000025484	ENSBTAG00000019147	RPS20	14	24955079	24956324	-	CODING	1.055777	9.30E-55
ENSBTAT00000021587	ENSBTAG00000016224	RPS7	8	112896493	112901718	-	CODING	0.75615	1.16E-54
ENSBTAT00000019176	ENSBTAG00000014417	-	25	33157514	33168430	+	CODING	-1.38778	7.11E-54
ENSBTAT00000019411	ENSBTAG00000014583	CALM	18	54169313	54178928	+	CODING	-0.77159	8.06E-54
ENSBTAT00000017580	ENSBTAG00000013208	SLC25A4	27	14546020	14550037	+	CODING	0.389569	1.40E-53
ENSBTAT00000019184	ENSBTAG00000014423	-	16	47992813	48000514	+	CODING	0.846897	7.18E-53
ENSBTAT00000009604	ENSBTAG00000007300	FHL3	3	108547857	108555472	+	CODING	-0.87963	2.11E-52
ENSBTAT00000065672	ENSBTAG00000038430	-	23	34219298	34219825	-	CODING	1.513209	2.08E-51
ENSBTAT00000018267	ENSBTAG00000013744	SYNPO	7	64020057	64027999	+	CODING	-0.98615	1.00E-50
ENSBTAT00000038488	ENSBTAG00000013631	GLUL	16	64948332	64958885	-	CODING	0.605147	6.91E-50
ENSBTAT00000003962	ENSBTAG00000005620	RPS3	15	55370367	55375306	+	CODING	0.624578	9.11E-50
ENSBTAT00000019318	ENSBTAG00000014534	EEF1A1	9	13233554	13236949	-	CODING	1.08883	1.08E-49
ENSBTAT00000005007	ENSBTAG00000005296	RPL13A	18	56394558	56398082	+	CODING	0.594881	2.46E-49
ENSBTAT00000026278	ENSBTAG00000019718	RPS15	7	45465834	45467519	+	CODING	0.692009	1.13E-48
ENSBTAT00000000359	ENSBTAG00000000286	PFKM	5	32312957	32337525	-	CODING	-0.60041	2.85E-48
ENSBTAT00000060250	ENSBTAG00000043258	SNORA18	29	1064655	1064785	+	CODING	9.268902	5.67E-48
ENSBTAT00000029208	ENSBTAG00000014614	ACTA2	26	10662363	10679648	-	CODING	1.284362	8.77E-48
ENSBTAT00000050559	ENSBTAG00000036078	EMP1	5	97081921	97101600	-	CODING	1.527585	1.71E-47
ENSBTAT00000017826	ENSBTAG00000039340	SCN4B	15	29257448	29277309	-	CODING	-1.27546	5.63E-47
ENSBTAT00000013713	ENSBTAG00000010389	FAM47E-STBD1	6	92967767	92971371	+	CODING	-1.66316	2.33E-46

ENSBTAT00000014038	ENSBTAG00000010619	PPP3R1	11	66582636	66642276	-	CODING	-1.23214	3.51E-46
ENSBTAT00000015584	ENSBTAG00000011734	ANKRD1	26	12571209	12580580	-	CODING	0.529695	6.85E-46
ENSBTAT00000009228	ENSBTAG00000006999	RYR1	18	48502352	48631056	+	CODING	-0.97214	8.87E-46
ENSBTAT00000048100	ENSBTAG00000025258	-	19	14514200	14548145	-	CODING	3.506768	1.34E-45
ENSBTAT00000044796	ENSBTAG00000005373	TPM1	10	47056204	47065846	-	CODING	-0.22529	1.81E-45
ENSBTAT00000000814	ENSBTAG00000000622	RPS17	21	23301663	23305104	-	CODING	0.455492	2.22E-45
ENSBTAT00000014306	ENSBTAG00000010799	MYL6	5	57486017	57489133	-	CODING	1.312846	2.27E-45
ENSBTAT00000064843	ENSBTAG00000047174	-	22	52425504	52428832	+	CODING	-1.29749	3.25E-45
ENSBTAT00000019875	ENSBTAG00000014930	MYLK2	13	61900820	61915380	+	CODING	-0.96669	4.93E-45
ENSBTAT00000064497	ENSBTAG00000046100	-	26	14093095	14093609	+	CODING	1.093425	6.48E-45
ENSBTAT00000014398	ENSBTAG00000010849	ANKRD23	11	2768909	2773197	-	CODING	-0.79786	7.82E-45
ENSBTAT00000023087	ENSBTAG00000017363	SAT1	X	126467442	126470489	-	CODING	2.012863	9.03E-45
ENSBTAT00000004519	ENSBTAG00000003476	FEM1A	7	20565301	20568880	-	CODING	-0.99455	6.76E-44
ENSBTAT00000042753	ENSBTAG00000030278	EGFL7	11	104130864	104131233	+	CODING	1.428758	7.96E-44
ENSBTAT00000035803	ENSBTAG00000025462	GADD45B	7	22411968	22414079	-	CODING	2.013723	1.10E-43
ENSBTAT00000024904	ENSBTAG00000018707	LDB3	28	41657195	41687361	+	CODING	-0.44751	1.72E-43
ENSBTAT00000040046	ENSBTAG00000027772	RPS25	15	30115928	30117641	-	CODING	0.737248	4.58E-43
ENSBTAT00000011846	ENSBTAG00000046303	-	11	98509474	98513781	+	CODING	1.298012	1.14E-42
ENSBTAT00000018414	ENSBTAG00000013866	RPS27	3	16505732	16507247	-	CODING	0.641574	1.91E-42
ENSBTAT00000020719	ENSBTAG00000015598	RPS10	23	8434576	8441522	-	CODING	0.584128	2.10E-42
ENSBTAT00000009965	ENSBTAG00000013343	FTL	18	55992910	55994741	+	CODING	1.561538	2.93E-42
ENSBTAT00000046493	ENSBTAG00000023600	APOD	1	72670963	72684269	+	CODING	1.236618	6.67E-42
ENSBTAT00000060554	ENSBTAG00000043553	GPX3	7	64286948	64295116	+	CODING	1.405784	8.69E-42
ENSBTAT00000038558	ENSBTAG00000007109	ASB2	21	59126042	59160261	-	CODING	-0.70213	2.57E-41
ENSBTAT00000005329	ENSBTAG00000004079	ZNF106	10	37890185	37941980	-	CODING	-1.10973	5.85E-41
ENSBTAT00000004658	ENSBTAG00000003581	SETD7	17	18463314	18515964	+	CODING	-1.15143	1.02E-40
ENSBTAT00000000101	ENSBTAG00000000092	HIF1AN	26	21291076	21300157	+	CODING	-1.20409	1.12E-40
ENSBTAT00000001993	ENSBTAG00000001521	UQCRB	14	70329414	70334124	+	CODING	0.439176	1.50E-40
ENSBTAT00000024387	ENSBTAG00000038488	TMSB4	X	140973702	140975767	-	CODING	1.044913	3.19E-40
ENSBTAT00000001187	ENSBTAG00000000894	PGK1	X	79282708	79305386	-	CODING	-0.68829	7.52E-40
ENSBTAT00000060567	ENSBTAG00000043550	MT-CYB	MT	14514	15653	+	CODING	-0.09975	1.12E-39
ENSBTAT00000000079	ENSBTAG00000037526	FABP4	14	46833665	46838053	-	CODING	1.590985	1.16E-39
ENSBTAT00000059463	ENSBTAG00000042471	SNORA25	29	1067324	1067450	+	CODING	8.911773	1.66E-39
ENSBTAT00000042757	ENSBTAG00000030281	-	11	104186563	104187141	-	CODING	0.801152	1.71E-39
ENSBTAT00000047412	ENSBTAG00000001601	PKM2	10	18965984	18992445	-	CODING	-0.42041	5.61E-39
ENSBTAT00000063142	ENSBTAG00000046289	U6	27	6982216	6982322	-	CODING	0.610842	6.80E-39
ENSBTAT00000003305	ENSBTAG00000002549	PTMA	2	120227920	120232987	+	CODING	1.240848	1.17E-38
ENSBTAT00000001838	ENSBTAG00000001398	ATP2A2	17	56458750	56512895	+	CODING	-0.50174	1.51E-37
ENSBTAT00000061000	ENSBTAG00000006907	NEB	2	44547340	44754701	+	CODING	-0.65468	5.40E-37
ENSBTAT00000013301	ENSBTAG00000027075	-	21	14304505	14304970	+	CODING	0.678954	6.00E-37
ENSBTAT00000031754	ENSBTAG00000023343	RPL28	18	62547220	62549950	-	CODING	0.72064	9.13E-37
ENSBTAT00000014452	ENSBTAG00000010880	TNNI2	29	50285049	50287648	-	CODING	0.189518	1.99E-36
ENSBTAT00000012544	ENSBTAG00000009535	RPS2	25	1520493	1522670	-	CODING	0.372136	3.09E-36
ENSBTAT00000065060	ENSBTAG00000046551	SNORA32	25	33716083	33716205	-	CODING	-8.0208	3.59E-36
ENSBTAT00000005279	ENSBTAG00000004037	JUN	3	87841042	87843087	+	CODING	0.884415	3.61E-36
ENSBTAT00000008669	ENSBTAG00000006607	CCNG1	7	77249997	77257261	+	CODING	-0.62333	1.08E-35
ENSBTAT00000005348	ENSBTAG00000004094	SPARCL1	6	104149824	104202396	-	CODING	1.002067	1.76E-35
ENSBTAT00000047320	ENSBTAG00000012044	RPL13	18	14533161	14535556	+	CODING	0.823916	1.87E-35
ENSBTAT00000017905	ENSBTAG00000013461	RPL24	1	46415223	46420721	-	CODING	0.579315	1.91E-35
ENSBTAT00000007504	ENSBTAG00000005714	ACTC1	10	30361781	30367052	-	CODING	-2.32321	1.98E-35
ENSBTAT00000025308	ENSBTAG00000019011	PGM1	3	82250295	82288338	-	CODING	-1.84408	2.24E-35
ENSBTAT00000018501	ENSBTAG00000013929	RRAD	18	34746055	34749250	-	CODING	0.8913	6.90E-35
ENSBTAT00000011795	ENSBTAG00000020116	JSP.1	23	28469735	28473401	-	CODING	1.100536	1.18E-34
ENSBTAT00000065158	ENSBTAG00000014249	IVNS1ABP	16	67765816	67785616	-	CODING	-0.66941	1.59E-34
ENSBTAT00000066266	ENSBTAG00000008573	ZFP36	18	49361497	49375317	+	CODING	0.76746	1.96E-34
ENSBTAT00000006866	ENSBTAG00000005211	RPL4	10	13318875	13323526	-	CODING	0.541857	2.29E-34
ENSBTAT00000028093	ENSBTAG00000021093	RPS16	18	49393725	49396191	-	CODING	0.618756	2.93E-34
ENSBTAT00000001780	ENSBTAG00000001349	KIF1C	19	27014446	27043780	-	CODING	-0.89788	3.60E-34
ENSBTAT00000025270	ENSBTAG00000018987	RPS25	3	16507484	16507861	-	CODING	0.683147	4.79E-34
ENSBTAT00000005486	ENSBTAG00000004189	MLXIP	17	55472596	55528501	-	CODING	-1.89177	5.36E-34
ENSBTAT00000024376	ENSBTAG00000018320	RPLP1	10	16324341	16326492	+	CODING	0.253048	1.14E-33
ENSBTAT00000014883	ENSBTAG00000011207	CNN1	7	17106394	17114222	+	CODING	1.900877	1.96E-33
ENSBTAT00000022835	ENSBTAG00000017183	PDLIM3	27	14769571	14800205	-	CODING	-0.32868	6.75E-33
ENSBTAT00000021796	ENSBTAG00000016391	-	9	16325798	16326091	-	CODING	0.700967	1.01E-32

ENSBTAT00000008386	ENSBTAG00000006396	GPI	18	44979578	45007642 +	CODING	-0.76493	2.00E-32	
ENSBTAT00000022510	ENSBTAG00000016924	CAP2	23	39646543	39781557 -	CODING	-0.92193	3.92E-32	
ENSBTAT00000021328	ENSBTAG00000016024	MYL9	24	37820258	37829300 +	CODING	0.370012	1.08E-31	
ENSBTAT00000020148	ENSBTAG00000015145	S100A11	3	18768796	18770416 +	CODING	1.270154	2.67E-31	
ENSBTAT00000064621	ENSBTAG00000046307	-	14	20738814	20740407 +	CODING	1.80287	3.43E-31	
ENSBTAT00000064427	ENSBTAG00000022158	TNNT3	29	50218484	50233948 -	CODING	1.145379	3.59E-31	
ENSBTAT00000054758	ENSBTAG00000039121	PTP4A2	2	122350144	122359897 +	CODING	-0.43887	6.73E-31	
ENSBTAT00000009155	ENSBTAG00000025385	RPL12	10	83480588	83481217 -	CODING	0.596113	7.64E-31	
ENSBTAT00000016957	ENSBTAG00000012760	NDUFB3	2	90077970	90088465 +	CODING	0.792265	1.89E-30	
ENSBTAT00000006534	ENSBTAG00000046332	ACTA1	28	419156	421250 -	CODING	-0.58962	7.94E-30	
ENSBTAT00000024641	ENSBTAG00000018513	FHL1	X	19809635	19820952 +	CODING	-0.16252	1.31E-29	
ENSBTAT00000028282	ENSBTAG00000021227	ATP5H	19	57020084	57024849 +	CODING	0.518292	1.41E-29	
ENSBTAT00000045066	ENSBTAG00000031786	FAU	5	47736728	47737193 +	CODING	0.601522	1.49E-29	
ENSBTAT00000013871	ENSBTAG00000010500	-	28	3323730	3333934 +	CODING	-0.99808	3.19E-29	
ENSBTAT00000010176	ENSBTAG00000007737	UBA52	7	4535467	4537851 -	CODING	0.386438	6.82E-29	
ENSBTAT00000027091	ENSBTAG00000020330	BTG1	5	22086071	22088781 -	CODING	1.006697	9.36E-29	
ENSBTAT00000012351	ENSBTAG00000009387	MYOM2	27	312538	376193 +	CODING	-0.88768	9.79E-29	
ENSBTAT00000018910	ENSBTAG00000014226	RPL34	6	17828130	17832711 -	CODING	0.60353	1.08E-28	
ENSBTAT00000016346	ENSBTAG00000012317	PNP	10	26667693	26673918 -	CODING	0.934103	1.33E-28	
ENSBTAT00000004044	ENSBTAG00000003109	ITM2B	12	18114553	18139506 +	CODING	0.505409	1.88E-28	
ENSBTAT00000023750	ENSBTAG00000017866	CD36	4	40581484	40643369 -	CODING	0.678323	3.91E-28	
ENSBTAT00000059596	ENSBTAG00000042604	U6	14	83907053	83907159 -	CODING	-0.25991	4.36E-28	
ENSBTAT00000061386	ENSBTAG00000020296	UBR3	2	26361548	26565226 -	CODING	-0.94777	7.34E-28	
ENSBTAT00000014304	ENSBTAG00000010799	MYL6	5	57486017	57489133 -	CODING	0.808828	7.56E-28	
ENSBTAT00000036888	ENSBTAG00000026199	ACTB	25	39343633	39347044 +	CODING	0.525009	1.08E-27	
ENSBTAT00000022183	ENSBTAG00000016683	BIKBA	21	46065549	46068942 -	CODING	1.352122	1.09E-27	
ENSBTAT00000005403	ENSBTAG00000004126	MLF1	1	109763292	109793739 -	CODING	-0.7892	1.55E-27	
ENSBTAT00000061449	ENSBTAG00000026986	TTN	2	18054943	18329808 +	CODING	-0.813	2.69E-27	
ENSBTAT00000034789	ENSBTAG00000019903	RAMP2	19	43441583	43443429 +	CODING	1.12986	4.58E-27	
ENSBTAT00000014744	ENSBTAG00000011104	RTN4	11	37575270	37611627 -	CODING	-0.4289	5.86E-27	
ENSBTAT00000025522	ENSBTAG00000019177	BIN1	2	5350654	5407851 +	CODING	-0.67957	6.34E-27	
ENSBTAT00000035955	ENSBTAG00000015131	SLC29A1	23	17845969	17856150 +	CODING	0.946018	1.13E-26	
ENSBTAT00000065455	ENSBTAG00000047155	C10orf71	28	43930428	43934735 +	CODING	-1.1184	1.30E-26	
ENSBTAT00000057357	ENSBTAG00000008401	PFKFB3	13	17380743	17406594 -	CODING	-0.70562	1.55E-26	
ENSBTAT00000036603	ENSBTAG00000012866	THBS4	10	10945425	10999244 +	CODING	1.19037	1.72E-26	
ENSBTAT00000049790	ENSBTAG00000013479	SLC9A3R2	25	1581874	1589619 +	CODING	2.080417	1.88E-26	
ENSBTAT00000026534	ENSBTAG00000019915	GSN	8	112609393	112639758 +	CODING	0.750259	2.10E-26	
ENSBTAT00000020867	ENSBTAG00000015720	WWP1	14	78599363	78699784 -	CODING	-1.01844	2.71E-26	
ENSBTAT00000020561	ENSBTAG00000015470	SYPL2	3	34082543	34095369 -	CODING	-0.91999	2.98E-26	
ENSBTAT00000023273	ENSBTAG00000017509	MYPN	28	24745815	24832856 +	CODING	-1.21337	4.34E-26	
ENSBTAT00000062174	ENSBTAG00000044741	SNORD97	15	42441794	42441945 +	CODING	-7.44206	5.65E-26	
ENSBTAT00000052289	ENSBTAG00000039728	RPLP1	16	52249583	52372101 -	CODING	0.318269	6.28E-26	
ENSBTAT00000042547	ENSBTAG00000030164	RPL38	19	57835002	57839034 -	CODING	0.535019	8.43E-26	
ENSBTAT00000046179	ENSBTAG00000011424	TPM2	8	60267452	60274815 -	CODING	-0.21143	1.04E-25	
ENSBTAT00000021068	ENSBTAG00000015848	PHKA1	X	83004459	83217747 +	CODING	-0.90014	1.07E-25	
ENSBTAT00000050064	ENSBTAG00000000223	PPM1B	11	26402682	26427596 +	CODING	-0.94115	2.08E-25	
ENSBTAT00000035177	ENSBTAG00000025136	MYOZ3	7	64031684	64048678 +	CODING	-1.52985	2.60E-25	
ENSBTAT00000019461	ENSBTAG00000014614	ACTA2	26	10662420	10679648 -	CODING	0.901504	2.81E-25	
ENSBTAT00000026400	ENSBTAG00000023274	-	5	107749635	107750145 +	CODING	0.495337	4.42E-25	
ENSBTAT00000002674	ENSBTAG00000002068	TAGLN2	3	9878839	9886647 +	CODING	1.311443	5.58E-25	
ENSBTAT00000019194	ENSBTAG00000014433	UBE2G1	19	25409138	25496726 -	CODING	-0.64094	5.59E-25	
ENSBTAT00000020150	ENSBTAG00000015147	S100A10	3	18799612	18810545 +	CODING	0.973327	8.34E-25	
ENSBTAT00000027457	ENSBTAG00000020605	SMTNL2	19	25702553	25724157 -	CODING	-0.69255	8.90E-25	
ENSBTAT00000005327	ENSBTAG00000004077	YWHAG	25	34884283	34906639 -	CODING	-0.70786	9.62E-25	
ENSBTAT00000026118	ENSBTAG00000019603	LDHB	5	88962679	88981219 +	CODING	0.894225	1.11E-24	
ENSBTAT00000008422	ENSBTAG00000022158	TNNT3	29	50218484	50233948 -	CODING	1.592243	1.45E-24	
ENSBTAT00000015829	ENSBTAG00000011931	CD63	5	57854278	57857485 +	CODING	0.581609	1.50E-24	
ENSBTAT00000021033	ENSBTAG00000015831	RPL18A	7	5206112	5209504 -	CODING	0.477665	2.10E-24	
ENSBTAT00000006190	ENSBTAG00000025313	DYNLL2	19	9118008	9125251 +	CODING	-1.0359	2.23E-24	
ENSBTAT00000045476	ENSBTAG00000032057	-	3	28623391	28623702 -	CODING	0.859152	2.54E-24	
ENSBTAT00000026009	ENSBTAG00000019525	-	28	29613180	29630076 +	CODING	-1.19586	3.69E-24	
ENSBTAT00000024749	ENSBTAG00000018598	HSPB6	18	46654243	46656762 -	CODING	-0.17341	3.78E-24	
ENSBTAT00000006312	ENSBTAG00000004806	PHKB	18	15962196	16155801 +	CODING	-0.98204	3.85E-24	
ENSBTAT00000002160	ENSBTAG00000001648	RPL21	12	32852826	32859542 -	CODING	0.692615	4.10E-24	

ENSBTAT00000020452	ENSBTAG00000015388	RPL18	18	55710193	55713956	-	CODING	0.493814	4.89E-24
ENSBTAT00000052009	ENSBTAG00000027444	SVIL	13	34860211	34965892	-	CODING	-1.32029	6.30E-24
ENSBTAT00000003377	ENSBTAG00000002610	FKBP3	21	55325021	55335301	-	CODING	0.719512	9.46E-24
ENSBTAT00000017372	ENSBTAG00000013066	IGF2	29	50046626	50065230	+	CODING	1.134232	1.75E-23
ENSBTAT00000013247	ENSBTAG00000027787	-	18	65621142	65621509	+	CODING	1.080962	1.91E-23
ENSBTAT00000007709	ENSBTAG00000005865	MAPK6	10	58437865	58449901	-	CODING	-0.60732	2.10E-23
ENSBTAT00000060242	ENSBTAG00000043250	7SK	23	24977642	24977972	+	CODING	1.766464	2.20E-23
ENSBTAT00000044290	ENSBTAG00000006712	KIAA0368	8	102504818	102589796	-	CODING	-0.99025	2.48E-23
ENSBTAT00000065572	ENSBTAG00000045568	-	24	13077339	13078261	+	CODING	0.589156	2.89E-23
ENSBTAT00000044763	ENSBTAG00000031573	ITGB1BP3	7	21290813	21294189	-	CODING	0.388192	3.48E-23
ENSBTAT00000026992	ENSBTAG00000021132	SYNPO2L	28	29799037	29805747	-	CODING	-1.23212	4.56E-23
ENSBTAT00000008502	ENSBTAG00000006487	RPS9	18	63381416	63388728	-	CODING	0.64039	7.18E-23
ENSBTAT00000060438	ENSBTAG00000043446	SNORA51	3	15691547	15691677	-	CODING	7.90966	9.99E-23
ENSBTAT00000059953	ENSBTAG00000042961	U6	19	57276185	57276291	-	CODING	-0.32535	1.10E-22
ENSBTAT00000016907	ENSBTAG00000012720	ANKRD2	26	18627373	18636269	+	CODING	0.703094	1.14E-22
ENSBTAT00000004188	ENSBTAG00000003228	RPL3	5	111261438	111267705	-	CODING	1.06828	1.16E-22
ENSBTAT00000015358	ENSBTAG00000011559	RPL7A	11	104311808	104315125	+	CODING	0.621257	1.34E-22
ENSBTAT00000062760	ENSBTAG00000045327	ACA64	25	1522971	1523097	+	CODING	-7.19452	1.65E-22
ENSBTAT00000010279	ENSBTAG00000007816	-	6	87555288	87556157	+	CODING	1.325742	2.16E-22
ENSBTAT00000000398	ENSBTAG00000000310	MFAP5	5	101647629	101659377	+	CODING	1.193341	2.20E-22
ENSBTAT00000000714	ENSBTAG00000000546	TOB1	19	36512064	36515184	+	CODING	-0.60729	2.77E-22
ENSBTAT00000018269	ENSBTAG00000013749	RHOQ	11	28834380	28871742	+	CODING	-0.79499	3.50E-22
ENSBTAT00000060533	ENSBTAG00000043541	SNORA5	4	77227804	77227938	+	CODING	7.851805	5.26E-22
ENSBTAT00000015924	ENSBTAG00000012003	PPIA	4	77409433	77413143	-	CODING	1.043699	6.66E-22
ENSBTAT00000025454	ENSBTAG00000019124	EIF4EBP2	28	26764785	26786028	+	CODING	-1.04718	8.47E-22
ENSBTAT00000025963	ENSBTAG00000019494	RPL10A	23	9391523	9394013	+	CODING	0.727915	1.05E-21
ENSBTAT00000036043	ENSBTAG00000005974	APOBEC2	23	14985521	14998187	+	CODING	-0.46404	1.09E-21
ENSBTAT00000021880	ENSBTAG00000016457	FXR1	1	86678925	86751963	-	CODING	-0.72564	1.82E-21
ENSBTAT00000024514	ENSBTAG00000018423	DDX5	19	49330954	49337523	-	CODING	0.842731	1.91E-21
ENSBTAT00000005143	ENSBTAG00000003937	-	13	22813811	22814335	+	CODING	0.487633	1.93E-21
ENSBTAT00000005982	ENSBTAG00000004553	TPM4	7	7923143	7948265	-	CODING	1.182449	2.04E-21
ENSBTAT00000016717	ENSBTAG00000012594	MRPS6	1	669920	733729	-	CODING	1.505355	3.24E-21
ENSBTAT00000002556	ENSBTAG00000039682	MTUS1	27	18632852	18698056	+	CODING	1.907297	3.41E-21
ENSBTAT00000061390	ENSBTAG00000005946	USP13	1	87949654	88075267	-	CODING	-1.20884	3.53E-21
ENSBTAT00000023622	ENSBTAG00000031788	GSTM1	3	33782528	33800900	-	CODING	-1.04433	4.71E-21
ENSBTAT00000002326	ENSBTAG00000001777	RPLP2	29	50768654	50770791	-	CODING	0.597979	5.36E-21
ENSBTAT00000024262	ENSBTAG00000018229	NFIX	7	13596367	13658112	-	CODING	-1.44982	5.52E-21
ENSBTAT00000002666	ENSBTAG00000002060	RPL19	19	40332947	40334703	+	CODING	0.339604	6.99E-21
ENSBTAT00000023751	ENSBTAG00000017869	CAV1	4	52173110	52208687	-	CODING	0.64729	7.69E-21
ENSBTAT00000054202	ENSBTAG00000011593	QKI	9	100370037	100480357	+	CODING	-0.7139	9.92E-21
ENSBTAT00000002229	ENSBTAG00000039335	ARRDC2	7	5101428	5105930	-	CODING	1.139415	1.04E-20
ENSBTAT00000000926	ENSBTAG00000000694	TAF10	15	47073272	47074686	+	CODING	0.994439	1.06E-20
ENSBTAT00000016279	ENSBTAG00000012276	RPL5	13	38980088	38981117	-	CODING	0.615417	1.43E-20
ENSBTAT00000024175	ENSBTAG00000018167	KLHL31	23	6771146	6785241	+	CODING	-1.44407	1.74E-20
ENSBTAT00000019803	ENSBTAG00000014872	CAPNS1	18	46987527	46994449	+	CODING	0.592982	1.82E-20
ENSBTAT00000028602	ENSBTAG00000021455	CFL1	29	44638896	44642280	-	CODING	0.979236	2.71E-20
ENSBTAT00000024555	ENSBTAG00000018451	PTMS	5	104039252	104040499	-	CODING	0.876288	2.71E-20
ENSBTAT00000011863	ENSBTAG00000009012	PTX3	1	111027804	111033868	-	CODING	4.929257	3.28E-20
ENSBTAT00000061582	ENSBTAG00000019585	MYOM1	24	37673546	37791756	-	CODING	-0.65435	3.45E-20
ENSBTAT00000065106	ENSBTAG00000047752	OTUD1	13	24655214	24656659	+	CODING	1.212323	3.70E-20
ENSBTAT00000031167	ENSBTAG00000002069	BOLA	23	28502524	28506312	-	CODING	0.861674	4.16E-20
ENSBTAT00000020125	ENSBTAG00000015127	SDC4	13	74393120	74412880	-	CODING	1.472206	4.56E-20
ENSBTAT00000010166	ENSBTAG00000027610	RPL36A	15	54468007	54468423	+	CODING	0.47038	4.83E-20
ENSBTAT00000008431	ENSBTAG00000006429	ACO2	5	113089139	113138185	+	CODING	-0.5743	6.04E-20
ENSBTAT00000046359	ENSBTAG00000018588	TMBIM6	5	30281520	30298109	-	CODING	-0.45911	7.50E-20
ENSBTAT00000026002	ENSBTAG00000019521	COX6A2	25	27737582	27738205	-	CODING	0.387102	8.93E-20
ENSBTAT00000012599	ENSBTAG00000009580	SH3BGRL3	2	127429585	127431204	-	CODING	1.042102	1.16E-19
ENSBTAT00000063728	ENSBTAG00000046024	PPP3CB	28	29592229	29602016	-	CODING	-1.23362	1.20E-19
ENSBTAT00000038460	ENSBTAG00000026886	MP68	21	70113045	70118400	-	CODING	0.590621	1.37E-19
ENSBTAT00000010744	ENSBTAG00000008172	EGLN3	21	44805298	44834310	-	CODING	-1.00768	1.39E-19
ENSBTAT00000029468	ENSBTAG00000022032	CHCHD10	17	73206771	73208629	-	CODING	0.506396	2.42E-19
ENSBTAT00000011677	ENSBTAG00000008868	CAPN3	10	37828797	37885860	+	CODING	-0.67115	3.65E-19
ENSBTAT00000023246	ENSBTAG00000017475	GNAS	13	58010287	58049012	-	CODING	1.006301	4.25E-19
ENSBTAT00000006376	ENSBTAG00000004850	KPNA3	12	19339291	19385309	-	CODING	-1.01808	4.42E-19

ENSBTAT00000038022	ENSBTAG00000013235	TINAGL1	2	122685823	122695535	-	CODING	1.435444	4.83E-19
ENSBTAT00000024391	ENSBTAG00000018331	CLEC3B	22	54791195	54797301	-	CODING	1.275414	5.19E-19
ENSBTAT00000019336	ENSBTAG00000014547	PGAM2	22	370972	373326	-	CODING	-0.29214	5.49E-19
ENSBTAT00000063192	ENSBTAG00000046177	IGFN1	16	81604665	81641464	+	CODING	-0.87502	7.52E-19
ENSBTAT00000039686	ENSBTAG00000014884	HIPK3	15	64704079	64758178	+	CODING	-1.12473	8.24E-19
ENSBTAT00000038612	ENSBTAG00000026972	MYF5	5	10339425	10342660	+	CODING	2.635559	8.70E-19
ENSBTAT00000014505	ENSBTAG00000010919	USP47	15	41298533	41373143	-	CODING	-0.93232	1.06E-18
ENSBTAT00000030619	ENSBTAG00000015839	MAP4	22	52369974	52462511	+	CODING	-1.34781	1.06E-18
ENSBTAT00000026197	ENSBTAG00000019658	ASB16	19	44614052	44621587	+	CODING	-0.81184	1.77E-18
ENSBTAT00000002015	ENSBTAG00000001538	RPS16	3	117506247	117506811	+	CODING	0.669986	1.87E-18
ENSBTAT00000018147	ENSBTAG00000013653	NFE2L1	19	39056971	39069122	-	CODING	-0.61795	1.96E-18
ENSBTAT00000057115	ENSBTAG00000019852	PDHA1	X	130874454	130897343	-	CODING	-0.68439	2.43E-18
ENSBTAT00000064624	ENSBTAG00000045783	-	16	5201143	5201319	-	CODING	-1.04141	3.02E-18
ENSBTAT00000037254	ENSBTAG00000026266	MYL12B	24	37834593	37910130	+	CODING	1.617967	3.66E-18
ENSBTAT00000047021	ENSBTAG00000021499	PSAP	28	28123710	28157099	-	CODING	-2.7631	3.67E-18
ENSBTAT00000027930	ENSBTAG00000020969	IQWD1	3	587034	768209	-	CODING	-0.81248	4.41E-18
ENSBTAT00000000506	ENSBTAG00000000393	SSPN	5	84096324	84138430	-	CODING	-1.52892	4.65E-18
ENSBTAT00000024157	ENSBTAG00000018152	MYADM	18	62018419	62024004	+	CODING	0.738064	5.00E-18
ENSBTAT00000028174	ENSBTAG00000021140	RMND5A	11	48010205	48066194	-	CODING	-1.1007	5.92E-18
ENSBTAT00000023183	ENSBTAG00000017441	RPL27	19	43669374	43671655	+	CODING	0.39756	6.77E-18
ENSBTAT00000009440	ENSBTAG00000007172	GOT2	18	26533095	26556740	-	CODING	-0.43286	7.50E-18
ENSBTAT00000017710	ENSBTAG00000013315	ATP5B	5	57119917	57125290	+	CODING	-0.34328	8.55E-18
ENSBTAT00000045994	ENSBTAG00000037778	CXCL3	6	90811062	90813079	+	CODING	1.899996	9.13E-18
ENSBTAT00000008517	ENSBTAG00000006499	PIP4K2B	19	39995184	40017502	-	CODING	-1.29864	1.01E-17
ENSBTAT00000023981	ENSBTAG00000018016	NUPR1	25	26340079	26341408	-	CODING	1.784347	1.09E-17
ENSBTAT00000002525	ENSBTAG00000001941	SEMA4D	8	90136712	90262973	+	CODING	-1.17155	1.13E-17
ENSBTAT00000001429	ENSBTAG00000001078	SRL	25	3392668	3404630	-	CODING	-0.99378	1.15E-17
ENSBTAT00000000497	ENSBTAG00000000385	ZBTB18	16	33913963	33918059	+	CODING	-1.49552	1.21E-17
ENSBTAT00000014801	ENSBTAG00000011145	NDUFA4	4	18973421	18980828	-	CODING	0.267334	1.46E-17
ENSBTAT00000014486	ENSBTAG00000010907	PPP1R1A	5	25627052	25631242	+	CODING	0.245335	1.80E-17
ENSBTAT00000028141	ENSBTAG00000021120	SMYD1	11	47799217	47848713	-	CODING	-1.13602	1.93E-17
ENSBTAT00000044517	ENSBTAG00000002783	PCYOX1	11	68560573	68572075	+	CODING	-0.96251	1.97E-17
ENSBTAT00000007124	ENSBTAG00000005414	-	1	51022816	51023423	+	CODING	1.500479	2.02E-17
ENSBTAT00000016993	ENSBTAG00000012788	COX6A1	17	64995248	64997121	+	CODING	1.238128	2.16E-17
ENSBTAT00000010126	ENSBTAG00000007700	PHYH	13	28254814	28275573	-	CODING	-0.43885	2.27E-17
ENSBTAT00000053250	ENSBTAG00000001274	PPM1L	1	107241227	107576086	-	CODING	-1.03595	2.44E-17
ENSBTAT00000028239	ENSBTAG00000021191	EHD2	18	55071102	55087454	+	CODING	0.888613	2.58E-17
ENSBTAT00000035337	ENSBTAG00000032954	ATP5E	22	35335190	35335581	+	CODING	0.380571	2.68E-17
ENSBTAT00000065223	ENSBTAG00000018167	KLHL31	23	6783412	6787028	+	CODING	-1.11222	2.89E-17
ENSBTAT00000012655	ENSBTAG00000009615	ANXA2	10	49860062	49904536	+	CODING	0.987205	2.99E-17
ENSBTAT00000001720	ENSBTAG00000001303	HSPB8	17	58405437	58418688	-	CODING	0.282418	3.09E-17
ENSBTAT00000061385	ENSBTAG00000039682	MTUS1	27	18574170	18698056	+	CODING	-0.97821	3.65E-17
ENSBTAT00000008132	ENSBTAG00000006189	ACTG1	19	51868429	51871276	+	CODING	0.476373	3.72E-17
ENSBTAT00000066175	ENSBTAG00000048167	U4	17	64874908	64875048	-	CODING	7.401268	4.20E-17
ENSBTAT00000006275	ENSBTAG00000004777	S100B	1	148009651	148016981	-	CODING	0.893976	5.13E-17
ENSBTAT00000055832	ENSBTAG00000006563	KBTBD5	22	15444466	15451791	+	CODING	0.8192	5.57E-17
ENSBTAT00000029327	ENSBTAG00000021992	MURC	8	91916165	91928339	+	CODING	-0.88505	6.30E-17
ENSBTAT00000006476	ENSBTAG00000004922	AGPAT5	27	4679600	4727246	+	CODING	-0.81915	7.08E-17
ENSBTAT00000015892	ENSBTAG00000011976	CYP4B1	3	99937185	99957408	-	CODING	2.487247	8.38E-17
ENSBTAT00000001646	ENSBTAG00000001246	ATP1A1	3	27002873	27025641	-	CODING	1.203682	8.50E-17
ENSBTAT00000012570	ENSBTAG00000009552	ATP2B1	5	19539973	19669793	-	CODING	-1.2886	8.53E-17
ENSBTAT00000009484	ENSBTAG00000007211	ASB12	X	101491186	101492856	+	CODING	-0.47006	1.13E-16
ENSBTAT00000011066	ENSBTAG00000008409	MYC	14	13769244	13774438	-	CODING	2.000213	1.15E-16
ENSBTAT00000049004	ENSBTAG00000017753	APP	1	9607382	9921004	+	CODING	0.89087	1.52E-16
ENSBTAT00000063623	ENSBTAG00000017509	MYPN	28	24797318	24833215	+	CODING	-1.11546	1.60E-16
ENSBTAT00000014215	ENSBTAG00000010738	CCL14	19	14775262	14780081	+	CODING	0.932841	1.62E-16
ENSBTAT00000054745	ENSBTAG00000008997	ENG	11	98517162	98541363	-	CODING	1.047123	2.00E-16
ENSBTAT00000007324	ENSBTAG00000005574	CLU	8	11043941	11061301	+	CODING	0.904708	2.30E-16
ENSBTAT00000019285	ENSBTAG00000014508	FBXO40	1	66712457	66717255	+	CODING	-0.90983	2.60E-16
ENSBTAT00000021330	ENSBTAG00000016026	PCOLCE2	1	127132777	127229651	+	CODING	1.725586	2.70E-16
ENSBTAT00000008319	ENSBTAG00000006342	ATPIF1	2	125727885	125730128	-	CODING	0.383984	3.37E-16
ENSBTAT00000010193	ENSBTAG00000007754	NDUFA3	18	63462789	63466137	-	CODING	0.627922	3.53E-16
ENSBTAT00000055220	ENSBTAG00000033008	MYOZ1	28	29780787	29798613	-	CODING	-0.20505	3.62E-16
ENSBTAT00000048862	ENSBTAG00000034493	C6ORF106	23	8591963	8688924	-	CODING	-0.95179	5.12E-16

ENSBTAT00000059118	ENSBTAG00000042126	SNORA66	16	37582336	37582467	+	CODING	-6.60446	5.97E-16
ENSBTAT00000035335	ENSBTAG00000012849	COL4A1	12	88876125	89009422	-	CODING	1.052271	6.47E-16
ENSBTAT00000018998	ENSBTAG00000014299	RHOC	3	30769966	30776469	+	CODING	1.392537	6.76E-16
ENSBTAT00000018339	ENSBTAG00000013799	-	2	107981292	107982291	-	CODING	-0.61075	9.35E-16
ENSBTAT00000029307	ENSBTAG00000021978	PARVB	5	115517820	115572329	+	CODING	-0.91392	9.86E-16
ENSBTAT00000057596	ENSBTAG00000040028	MGC166429	7	2600732	2618482	-	CODING	-0.7305	1.30E-15
ENSBTAT00000028036	ENSBTAG00000021048	ADM	15	42911044	42913326	-	CODING	2.067523	1.83E-15
ENSBTAT00000009575	ENSBTAG00000007281	SEMA6C	3	19717720	19728131	+	CODING	-1.69671	1.89E-15
ENSBTAT00000011643	ENSBTAG00000008842	JPH1	14	39633189	39725607	-	CODING	-0.62255	2.38E-15
ENSBTAT00000028634	ENSBTAG00000021481	CA14	3	20420023	20427199	-	CODING	-1.47567	2.39E-15
ENSBTAT00000028880	ENSBTAG00000021672	RGS1	16	13314192	13318292	-	CODING	2.109633	2.46E-15
ENSBTAT00000064506	ENSBTAG00000022158	TNNT3	29	50218484	50233948	-	CODING	-12.0266	2.48E-15
ENSBTAT00000004161	ENSBTAG00000003205	RPL35	11	95850116	95854734	-	CODING	0.621981	2.57E-15
ENSBTAT00000065769	ENSBTAG00000047330	FABP5	14	46644609	46649827	+	CODING	0.804062	2.62E-15
ENSBTAT00000039655	ENSBTAG00000010677	LIMCH1	6	61872553	62222095	+	CODING	-1.30824	2.66E-15
ENSBTAT00000022378	ENSBTAG00000016822	PPIB	10	45874978	45880918	+	CODING	1.092414	2.80E-15
ENSBTAT00000031470	ENSBTAG00000005718	PLIN2	8	25129168	25142901	+	CODING	0.880881	3.23E-15
ENSBTAT00000056227	ENSBTAG00000002964	TXLNB	9	77937305	77996890	-	CODING	-0.98334	3.61E-15
ENSBTAT00000002707	ENSBTAG00000002098	CDC34	7	44789108	44794947	+	CODING	-0.44469	4.30E-15
ENSBTAT00000019142	ENSBTAG00000014396	TIEG1	14	63951758	63957275	+	CODING	-0.60257	4.61E-15
ENSBTAT00000029092	ENSBTAG00000021823	HFE2	3	21470721	21474444	+	CODING	-1.15302	5.59E-15
ENSBTAT00000018886	ENSBTAG00000014205	PRKAR2A	22	51658044	51727840	+	CODING	-0.91737	6.07E-15
ENSBTAT00000004952	ENSBTAG00000003798	AMFR	18	24290808	24334739	+	CODING	-0.92042	7.90E-15
ENSBTAT00000008887	ENSBTAG00000006752	PFKFB4	22	51898015	51933996	+	CODING	-0.83134	1.15E-14
ENSBTAT00000063866	ENSBTAG00000045504	-	9	42351250	42352038	-	CODING	0.576309	1.18E-14
ENSBTAT00000020512	ENSBTAG00000015434	DSTN	13	38259752	38284536	+	CODING	0.954817	1.26E-14
ENSBTAT00000004728	ENSBTAG00000024657	-	15	83248791	83286773	+	CODING	-0.85757	1.46E-14
ENSBTAT00000029304	ENSBTAG00000021979	EEF1B	2	94922839	94926122	+	CODING	0.502591	1.59E-14
ENSBTAT00000028016	ENSBTAG00000021035	CTSK	3	19994998	20007861	+	CODING	1.499341	1.69E-14
ENSBTAT00000037367	ENSBTAG00000026327	RPL8	14	1505030	1507633	-	CODING	0.540932	1.74E-14
ENSBTAT00000003460	ENSBTAG00000002670	C28H10ORF10	28	44952355	44953778	+	CODING	2.192168	1.81E-14
ENSBTAT00000013799	ENSBTAG00000010452	PODXL	4	96032591	96039050	-	CODING	1.102436	1.82E-14
ENSBTAT00000034186	ENSBTAG00000024561	H3F3A	16	29832591	29840787	+	CODING	0.630797	1.97E-14
ENSBTAT00000056385	ENSBTAG00000016005	PPP3CA	6	24812682	25136247	+	CODING	-1.13346	1.97E-14
ENSBTAT00000060896	ENSBTAG00000043904	SNORA57	4	43820203	43820347	+	CODING	7.0856	2.53E-14
ENSBTAT00000025142	ENSBTAG00000018887	IPO13	3	102682317	102701941	-	CODING	-0.91846	2.76E-14
ENSBTAT00000020520	ENSBTAG00000015441	ACTB	11	10717554	10732398	-	CODING	1.99211	2.94E-14
ENSBTAT00000046544	ENSBTAG00000032774	C28H10ORF116	28	41912322	41914565	+	CODING	0.99189	3.79E-14
ENSBTAT00000027409	ENSBTAG00000020569	CACNA2D1	4	38712468	38856748	+	CODING	-1.34937	3.94E-14
ENSBTAT00000029256	ENSBTAG00000021944	C10H14ORF166	10	44879976	44894546	+	CODING	0.498394	4.00E-14
ENSBTAT00000002970	ENSBTAG00000002299	SEL1L3	6	46791165	46881531	-	CODING	-1.40067	4.17E-14
ENSBTAT00000000730	ENSBTAG00000000560	-	19	13525432	13526742	+	CODING	0.587471	4.35E-14
ENSBTAT00000044139	ENSBTAG00000013066	IGF2	29	50058063	50062631	+	CODING	1.70633	4.45E-14
ENSBTAT00000027504	ENSBTAG00000020638	TIMP3	5	71751415	71809052	+	CODING	0.634652	4.70E-14
ENSBTAT00000063321	ENSBTAG00000046121	-	14	79340383	79345452	+	CODING	2.098061	5.00E-14
ENSBTAT00000040741	ENSBTAG00000028359	U3	19	9798918	9799131	-	CODING	3.50924	6.23E-14
ENSBTAT00000022041	ENSBTAG00000016568	LUC7L	25	225533	257902	-	CODING	1.120784	7.35E-14
ENSBTAT00000061283	ENSBTAG00000016167	ATL2	11	20691386	20758963	-	CODING	-0.909	7.43E-14
ENSBTAT00000040201	ENSBTAG00000027879	MPC1	9	102880277	102893601	-	CODING	0.555126	7.97E-14
ENSBTAT00000001758	ENSBTAG00000001335	GHR	20	31890736	32199996	-	CODING	-0.98048	8.15E-14
ENSBTAT00000063883	ENSBTAG00000045728	SCD	26	21141592	21148318	-	CODING	-1.0561	8.50E-14
ENSBTAT00000033091	ENSBTAG00000015473	RPS27A	11	37823446	37825428	+	CODING	0.406441	8.57E-14
ENSBTAT00000023729	ENSBTAG00000017851	RXRA	11	105990344	106015000	+	CODING	-1.55634	8.79E-14
ENSBTAT00000003431	ENSBTAG00000002648	RPS18	23	7388703	7393361	+	CODING	0.366819	1.37E-13
ENSBTAT00000017010	ENSBTAG00000012805	TSPAN13	4	25213088	25251241	+	CODING	0.883566	1.41E-13
ENSBTAT00000059592	ENSBTAG00000042600	SNORD94	11	48600030	48600165	-	CODING	-6.32735	1.50E-13
ENSBTAT00000014839	ENSBTAG00000011173	FAM189A2	8	45784659	45816860	+	CODING	-1.11809	1.56E-13
ENSBTAT00000007616	ENSBTAG00000005793	PEA15	3	9578375	9588605	-	CODING	0.867306	1.61E-13
ENSBTAT00000015231	ENSBTAG00000011463	MID1IP1	X	109949360	109951138	-	CODING	0.590287	1.62E-13
ENSBTAT00000044762	ENSBTAG00000000111	UGP2	11	62244354	62299938	+	CODING	-0.46047	1.69E-13
ENSBTAT00000005060	ENSBTAG00000003877	ZCCHC24	28	35189622	35251005	-	CODING	-1.35252	1.84E-13
ENSBTAT00000022832	ENSBTAG00000017181	MACROD1	29	42890800	43092842	-	CODING	0.30532	1.93E-13
ENSBTAT00000062472	ENSBTAG00000045039	SCARNA17	24	49943458	49943600	+	CODING	-6.31341	1.94E-13
ENSBTAT00000059670	ENSBTAG00000042678	SNORA71	13	68003660	68003794	+	CODING	-6.30936	2.08E-13

ENSBTAT00000025311	ENSBTAG00000019015	IFITM3	29	51367009	51368065	+	CODING	1.029368	2.15E-13
ENSBTAT00000059715	ENSBTAG00000042723	U11	2	125390299	125390431	-	CODING	-6.29813	2.56E-13
ENSBTAT00000026358	ENSBTAG00000019782	TPI1	5	103942403	103945759	-	CODING	-0.22939	2.62E-13
ENSBTAT00000013663	ENSBTAG00000010347	EZR	9	96598249	96643275	-	CODING	1.324421	2.94E-13
ENSBTAT00000017122	ENSBTAG00000012885	ACAT1	15	17999932	18028984	+	CODING	0.639498	3.92E-13
ENSBTAT00000017995	ENSBTAG00000013533	CLIC1	23	27393342	27398934	+	CODING	1.531522	4.12E-13
ENSBTAT00000064544	ENSBTAG00000046531	-	X	95330253	95330600	-	CODING	0.912625	4.14E-13
ENSBTAT00000005660	ENSBTAG00000004322	FOS	10	86883739	86887169	+	CODING	-0.51168	5.19E-13
ENSBTAT00000002092	ENSBTAG00000038067	MT1A	18	24106722	24108521	-	CODING	2.648397	5.65E-13
ENSBTAT00000012057	ENSBTAG00000009151	PYGO1	10	54865902	54887753	+	CODING	-1.30425	5.69E-13
ENSBTAT00000024965	ENSBTAG00000018747	PRKAA2	3	90055299	90127142	-	CODING	-1.9088	6.90E-13
ENSBTAT00000059485	ENSBTAG00000042493	SNORA70	X	40366530	40366664	+	CODING	-6.23991	7.20E-13
ENSBTAT00000018099	ENSBTAG00000013614	TMEM38A	7	6285842	6312400	-	CODING	-0.64608	7.34E-13
ENSBTAT00000027592	ENSBTAG00000046981	SVIL	13	34922199	34945103	-	CODING	-0.54552	8.12E-13
ENSBTAT00000022033	ENSBTAG00000016563	GOLGA4	22	10813998	10923960	+	CODING	-0.76707	8.19E-13
ENSBTAT00000061037	ENSBTAG00000044126	SNTB1	14	84253919	84504093	-	CODING	-1.23828	1.00E-12
ENSBTAT00000018411	ENSBTAG00000013863	DUSP1	20	4449109	4452189	-	CODING	0.428883	1.04E-12
ENSBTAT00000010698	ENSBTAG00000008137	-	5	75519790	75520044	+	CODING	0.466743	1.08E-12
ENSBTAT00000032663	ENSBTAG00000020791	RAPGEF1	11	101728372	101793685	-	CODING	-1.01602	1.19E-12
ENSBTAT00000029117	ENSBTAG00000021843	UBQLN2	X	98958218	98960092	+	CODING	-0.97124	1.21E-12
ENSBTAT00000023150	ENSBTAG00000017416	KPNA4	1	107774353	107834443	+	CODING	-0.67085	1.32E-12
ENSBTAT00000063682	ENSBTAG00000047418	SLC25A6	GJ06[illegible]	1867	6353	+	CODING	0.888245	1.44E-12
ENSBTAT00000000993	ENSBTAG00000000745	AQP1	4	65830992	65845186	-	CODING	1.102332	1.46E-12
ENSBTAT00000019527	ENSBTAG00000014667	PAIP2B	11	13393546	13397285	+	CODING	-1.1399	1.75E-12
ENSBTAT00000002513	ENSBTAG00000001932	MGC142781	11	99576794	99589668	-	CODING	-0.59802	1.87E-12
ENSBTAT00000022636	ENSBTAG00000017024	PPARGC1A	6	44854113	44960533	-	CODING	1.872816	2.00E-12
ENSBTAT00000018428	ENSBTAG00000013881	GJA4	3	111481702	111482589	-	CODING	1.28335	2.03E-12
ENSBTAT00000016712	ENSBTAG00000012589	HSPE1	2	86449525	86451564	+	CODING	0.434745	2.21E-12
ENSBTAT00000011257	ENSBTAG00000008541	MGST1	5	93926791	93950162	-	CODING	1.316536	2.54E-12
ENSBTAT00000015694	ENSBTAG00000011824	OGN	8	85453132	85468721	+	CODING	1.138423	2.68E-12
ENSBTAT00000019129	ENSBTAG00000014387	PRKAB2	3	22635094	22646401	+	CODING	-1.49433	3.69E-12
ENSBTAT00000004411	ENSBTAG00000003403	PADI2	2	136049644	136103406	+	CODING	-2.13793	3.92E-12
ENSBTAT00000021614	ENSBTAG00000016240	RNF157	19	56112050	56167541	+	CODING	-1.4524	4.81E-12
ENSBTAT00000063200	ENSBTAG00000046117	TMSB4X	11	63290422	63395507	+	CODING	0.994693	5.40E-12
ENSBTAT00000025843	ENSBTAG00000019394	ANO5	29	22783297	22859063	-	CODING	-1.62129	5.70E-12
ENSBTAT00000029478	ENSBTAG00000009773	KREMEN1	17	70568207	70601539	+	CODING	-1.55671	5.77E-12
ENSBTAT00000038527	ENSBTAG00000001083	-	2	23443013	23608298	-	CODING	-1.67204	6.33E-12
ENSBTAT00000026928	ENSBTAG00000020219	MSS51	28	29578510	29588920	-	CODING	-1.026	6.37E-12
ENSBTAT00000011521	ENSBTAG00000008743	ALDH2	17	64551612	64577901	-	CODING	-0.63444	6.48E-12
ENSBTAT00000011265	ENSBTAG00000008545	ATF3	16	72820026	72832974	-	CODING	0.775473	7.18E-12
ENSBTAT00000039828	ENSBTAG00000027654	EIF4EBP1	27	32951594	32973435	+	CODING	0.740207	7.25E-12
ENSBTAT00000024243	ENSBTAG00000018214	SHISA2	12	33578134	33583714	+	CODING	-1.60771	7.64E-12
ENSBTAT00000028574	ENSBTAG00000021435	MAFF	5	110475770	110484826	+	CODING	0.961433	8.46E-12
ENSBTAT00000033787	ENSBTAG00000001908	DLD	4	49230967	49257713	+	CODING	-0.59116	8.51E-12
ENSBTAT00000007861	ENSBTAG00000005990	S1PR1	3	42184097	42188752	-	CODING	0.967815	8.67E-12
ENSBTAT00000020194	ENSBTAG00000015177	PRSS23	29	8788703	8797308	-	CODING	-0.74385	8.86E-12
ENSBTAT00000019555	ENSBTAG00000014693	TMEM88	19	28156792	28157769	+	CODING	1.298527	9.89E-12
ENSBTAT00000020168	ENSBTAG00000015163	TM4SF1	1	119750363	119758888	+	CODING	1.02818	9.99E-12
ENSBTAT00000023213	ENSBTAG00000017462	ATF4	5	111462845	111464936	+	CODING	-0.16819	1.05E-11
ENSBTAT00000021862	ENSBTAG00000016444	FAM134B	20	56709603	56758641	+	CODING	0.761328	1.05E-11
ENSBTAT00000013079	ENSBTAG00000009908	RPS3A	17	6703060	6707745	-	CODING	0.327926	1.06E-11
ENSBTAT00000048021	ENSBTAG00000033835	MPZ	3	8229236	8234089	+	CODING	1.126212	1.08E-11
ENSBTAT00000033450	ENSBTAG00000013860	GADD45A	3	77972153	77975265	-	CODING	0.569945	1.36E-11
ENSBTAT00000005600	ENSBTAG00000004279	RHOA	22	51277867	51323093	+	CODING	0.511589	1.38E-11
ENSBTAT00000027927	ENSBTAG00000020968	MPC2	3	768307	799012	+	CODING	0.683355	1.39E-11
ENSBTAT00000019970	ENSBTAG00000015000	VCP	8	59732258	59746989	-	CODING	-0.47449	1.42E-11
ENSBTAT00000019808	ENSBTAG00000014878	COX7A1	18	46994991	46996544	-	CODING	0.389005	1.46E-11
ENSBTAT00000032006	ENSBTAG00000023471	RPL36	5	28525103	28525496	+	CODING	0.577725	1.52E-11
ENSBTAT00000017086	ENSBTAG00000012855	LPL	8	67481089	67511227	+	CODING	1.035821	1.60E-11
ENSBTAT00000016981	ENSBTAG00000012777	SRF	23	16747958	16754754	+	CODING	-0.90387	1.69E-11
ENSBTAT00000057593	ENSBTAG00000019517	ELN	25	33787889	33820672	-	CODING	1.721646	1.90E-11
ENSBTAT00000001121	ENSBTAG00000000843	NDRG2	10	26134842	26142255	+	CODING	-0.43108	1.92E-11
ENSBTAT00000021401	ENSBTAG00000016081	PTMA	2	47998614	47999416	+	CODING	1.290275	2.11E-11
ENSBTAT00000033899	ENSBTAG00000002280	KIF5B	13	33619259	33665129	+	CODING	-0.77983	2.12E-11

ENSBTAT00000009285	ENSBTAG00000007062	IGFBP5	2	105378991	105397646	-	CODING	-1.8564	2.13E-11
ENSBTAT00000038531	ENSBTAG00000038025	H2B	3	20771300	20773622	+	CODING	-0.38261	2.25E-11
ENSBTAT00000046515	ENSBTAG00000003741	NEURL1	26	24330861	24403159	+	CODING	-1.19039	2.40E-11
ENSBTAT00000020105	ENSBTAG00000015109	TOB2	5	113060940	113070989	-	CODING	-0.80425	2.41E-11
ENSBTAT00000059955	ENSBTAG00000042963	SNORA8	29	1065601	1065737	+	CODING	-6.02583	2.44E-11
ENSBTAT00000024312	ENSBTAG00000018267	TRIM54	11	72327351	72348528	-	CODING	0.382548	2.61E-11
ENSBTAT00000063331	ENSBTAG00000046054	RHOB	11	78464206	78466417	-	CODING	0.730688	2.70E-11
ENSBTAT00000020261	ENSBTAG00000015228	CD74	7	63748885	63756646	-	CODING	0.517896	2.74E-11
ENSBTAT00000014877	ENSBTAG00000023462	RPS6	8	24927468	24930788	+	CODING	0.918716	2.94E-11
ENSBTAT00000010213	ENSBTAG00000007767	TBX15	3	24315881	24359735	+	CODING	-0.98152	2.96E-11
ENSBTAT00000052276	ENSBTAG00000038379	-	X	39983832	39984337	+	CODING	0.531272	3.00E-11
ENSBTAT00000046364	ENSBTAG00000005314	MFN2	16	42561715	42581003	-	CODING	-1.09113	3.07E-11
ENSBTAT00000021554	ENSBTAG00000016194	FBXO32	14	17919823	17951894	+	CODING	-0.44399	3.12E-11
ENSBTAT00000040033	ENSBTAG00000027766	C1QTNF5	15	30448399	30450337	-	CODING	2.597478	3.13E-11
ENSBTAT00000018808	ENSBTAG00000014151	RCSD1	3	1058468	1130225	-	CODING	-1.02035	3.20E-11
ENSBTAT00000064053	ENSBTAG00000047957	SCD	26	21132751	21133969	+	CODING	-1.12476	3.67E-11
ENSBTAT00000026323	ENSBTAG00000019754	PRKCDBP	15	47329716	47331334	+	CODING	1.217989	4.25E-11
ENSBTAT00000001128	ENSBTAG00000006724	PPP1R2	1	72708757	72726620	+	CODING	-0.58459	4.86E-11
ENSBTAT00000028994	ENSBTAG00000021752	DNAJB4	3	66923000	66931737	-	CODING	0.553204	5.73E-11
ENSBTAT00000060287	ENSBTAG00000043295	SNORA13	10	1763369	1763505	-	CODING	-5.96318	6.31E-11
ENSBTAT00000029418	ENSBTAG00000022020	CLDN5	17	74749250	74750524	-	CODING	1.197688	6.32E-11
ENSBTAT00000039835	ENSBTAG00000014463	CAMK2D	6	12965129	13272298	+	CODING	-1.00639	6.95E-11
ENSBTAT00000021369	ENSBTAG00000016057	CSRP1	16	49332770	49353517	-	CODING	0.944699	7.36E-11
ENSBTAT00000018888	ENSBTAG00000014208	RPL35A	1	70788697	70792140	-	CODING	0.586983	7.41E-11
ENSBTAT00000005727	ENSBTAG00000004368	NFATC3	18	35640709	35732176	+	CODING	-1.19306	7.97E-11
ENSBTAT00000061564	ENSBTAG00000039374	RANBP6	12	79000723	79039990	+	CODING	-0.6917	7.98E-11
ENSBTAT00000021638	ENSBTAG00000016267	SERPING1	15	82159474	82172143	+	CODING	1.127082	8.14E-11
ENSBTAT00000032193	ENSBTAG00000016341	RGS5	3	6228349	6426528	+	CODING	0.600803	9.51E-11
ENSBTAT00000010231	ENSBTAG00000007782	MYOT	7	50941047	50958425	+	CODING	-0.13585	9.78E-11
ENSBTAT00000004980	ENSBTAG00000003826	SCN1B	18	45960622	45970522	+	CODING	-0.60782	1.03E-10
ENSBTAT00000015387	ENSBTAG00000035081	SERINC2	2	122824336	122835401	+	CODING	-0.76507	1.06E-10
ENSBTAT00000059254	ENSBTAG00000046842	U4	17	64875706	64875846	-	CODING	-5.92808	1.06E-10
ENSBTAT00000057533	ENSBTAG00000007662	GRP78	11	96115572	96119306	-	CODING	0.775995	1.08E-10
ENSBTAT00000063744	ENSBTAG00000046450	EIF1	3	60346812	60347472	+	CODING	-0.2739	1.12E-10
ENSBTAT00000000187	ENSBTAG00000000163	DDIT4	28	28483403	28485410	+	CODING	0.485895	1.14E-10
ENSBTAT00000017131	ENSBTAG00000012890	SLC25A3	5	63086353	63092547	+	CODING	-0.38895	1.14E-10
ENSBTAT00000016514	ENSBTAG00000012447	PPP1CB	11	70961032	70997556	-	CODING	-0.42509	1.18E-10
ENSBTAT00000039242	ENSBTAG00000003512	MYH7B	13	64888626	64912758	+	CODING	0.927687	1.28E-10
ENSBTAT00000064387	ENSBTAG00000047502	FKBP5	23	9521254	9637802	-	CODING	0.497457	1.29E-10
ENSBTAT00000024128	ENSBTAG00000018127	PPM1A	10	72757582	72768778	+	CODING	-0.61005	1.37E-10
ENSBTAT00000015348	ENSBTAG00000011548	AMPD1	3	28756908	28768496	+	CODING	-0.54224	1.49E-10
ENSBTAT00000015811	ENSBTAG00000011917	GPAM	26	32963414	33003349	-	CODING	1.179149	1.54E-10
ENSBTAT00000029301	ENSBTAG00000021976	NDUFS1	2	94893227	94922667	-	CODING	-0.64496	1.58E-10
ENSBTAT00000024225	ENSBTAG00000002960	WDR82P1	22	49218179	49227366	+	CODING	0.655389	1.58E-10
ENSBTAT00000006899	ENSBTAG00000005244	RASL11A	12	32842604	32844825	-	CODING	2.038162	1.60E-10
ENSBTAT00000002349	ENSBTAG00000001794	RPL36	7	19833364	19834615	-	CODING	0.603349	1.69E-10
ENSBTAT00000004244	ENSBTAG00000027629	ANK1	27	36255859	36265466	-	CODING	-0.42928	1.75E-10
ENSBTAT00000054742	ENSBTAG00000037470	-	14	38614593	38615161	+	CODING	1.613498	1.90E-10
ENSBTAT00000025716	ENSBTAG00000019314	USP25	1	20664990	20798853	-	CODING	-0.64733	1.91E-10
ENSBTAT00000047899	ENSBTAG00000033727	RBPMS	27	25637686	25816012	+	CODING	1.708622	1.99E-10
ENSBTAT00000021398	ENSBTAG00000016076	TRAK1	22	15084213	15160669	+	CODING	-0.9975	2.09E-10
ENSBTAT00000045265	ENSBTAG00000010422	MDM2	5	45178135	45203567	-	CODING	-1.18668	2.10E-10
ENSBTAT00000008388	ENSBTAG00000006398	TOMM7	9	89692607	89692771	-	CODING	0.645468	2.14E-10
ENSBTAT00000002261	ENSBTAG00000001721	GYG1	1	120090467	120127892	-	CODING	-0.51955	2.17E-10
ENSBTAT00000017277	ENSBTAG00000012996	RNF11	3	95599737	95601983	-	CODING	-0.52817	2.29E-10
ENSBTAT00000031490	ENSBTAG00000023147	LINGO1	21	33292504	33294348	-	CODING	-0.82347	2.38E-10
ENSBTAT00000031821	ENSBTAG00000030592	UBL5	1	154150028	154150249	+	CODING	0.764303	2.48E-10
ENSBTAT00000034976	ENSBTAG00000025046	ALKBH5	19	35025249	35041293	-	CODING	-0.86209	2.57E-10
ENSBTAT00000032142	ENSBTAG00000038131	ABCC5	1	83736248	83764147	+	CODING	1.314751	2.78E-10
ENSBTAT00000066051	ENSBTAG00000046176	SPEG	2	108089328	108147468	+	CODING	-1.15226	3.09E-10
ENSBTAT00000021683	ENSBTAG00000016296	GPD1	5	29958501	29964085	-	CODING	-0.37167	3.21E-10
ENSBTAT00000012779	ENSBTAG00000009689	RWDD1	9	34577932	34595665	-	CODING	0.502364	3.26E-10
ENSBTAT00000001490	ENSBTAG00000001120	CORO6	19	21426533	21434772	-	CODING	-0.46304	3.34E-10
ENSBTAT00000010884	ENSBTAG00000008271	MEDAG	12	30021743	30039901	-	CODING	1.70453	3.80E-10

ENSBTAT00000026293	ENSBTAG00000019730	SFRS18	9	51022633	51049299	+	CODING	0.787908	4.07E-10
ENSBTAT00000005025	ENSBTAG00000003851	CCNL1	1	111318106	111331103	+	CODING	1.203806	4.08E-10
ENSBTAT00000010682	ENSBTAG00000008122	GNG5	3	59783839	59791977	+	CODING	0.583845	4.17E-10
ENSBTAT00000003794	ENSBTAG00000002922	-	29	17833137	17887917	+	CODING	-1.54604	4.70E-10
ENSBTAT00000028988	ENSBTAG00000021746	ANXA5	6	3542635	3575330	+	CODING	0.87746	4.71E-10
ENSBTAT00000044159	ENSBTAG00000031184	CDKN1C	29	49368787	49370785	+	CODING	1.635076	4.78E-10
ENSBTAT00000055320	ENSBTAG00000020308	EIF4G2	15	42435880	42446235	+	CODING	-0.30465	4.80E-10
ENSBTAT00000059908	ENSBTAG00000042916	SNORD15	15	55374442	55374585	+	CODING	6.454739	4.88E-10
ENSBTAT00000026126	ENSBTAG00000045492	ANG	10	26429018	26445589	-	CODING	1.511024	5.17E-10
ENSBTAT00000016666	ENSBTAG00000016457	FXR1	1	86678925	86751962	-	CODING	-0.78683	5.64E-10
ENSBTAT00000021808	ENSBTAG00000016401	OPTN	13	28078762	28117627	+	CODING	-0.40508	5.73E-10
ENSBTAT00000027837	ENSBTAG00000020894	LAPTM4A	11	78862495	78880461	+	CODING	0.437746	5.75E-10
ENSBTAT00000048484	ENSBTAG00000034206	EMCN	6	25578634	25700215	+	CODING	1.054103	5.82E-10
ENSBTAT00000022366	ENSBTAG00000037558	GRO1	6	90822748	90824841	+	CODING	2.988706	6.03E-10
ENSBTAT00000009097	ENSBTAG00000006928	OAT	26	44378157	44397476	-	CODING	-1.12517	6.56E-10
ENSBTAT00000001034	ENSBTAG00000000778	-	23	17867543	17873174	+	CODING	0.402691	6.92E-10
ENSBTAT00000028899	ENSBTAG00000021685	EEF1A2	13	54623917	54631595	+	CODING	-0.18353	7.21E-10
ENSBTAT00000045699	ENSBTAG00000032217	-	1	84324177	84325181	-	CODING	0.805005	7.60E-10
ENSBTAT00000000144	ENSBTAG00000000132	EIF4A1	19	27915413	27921421	+	CODING	0.843744	7.76E-10
ENSBTAT00000002787	ENSBTAG00000002151	ASB15	4	88697392	88729365	+	CODING	-1.65735	7.92E-10
ENSBTAT00000018566	ENSBTAG00000013953	CALD1	4	99475015	99580189	+	CODING	0.858054	8.61E-10
ENSBTAT00000035874	ENSBTAG00000022590	BOLA	23	27863067	27867532	+	CODING	1.176241	8.99E-10
ENSBTAT00000028221	ENSBTAG00000021176	CRISPLD2	18	10985132	11050904	+	CODING	1.086602	9.28E-10
ENSBTAT00000017430	ENSBTAG00000013113	VDAC1	7	47247744	47273458	-	CODING	-0.38644	9.43E-10
ENSBTAT00000028712	ENSBTAG00000021549	PHPT1	11	106334245	106335688	-	CODING	0.516774	9.77E-10
ENSBTAT00000024417	ENSBTAG00000018352	ABRA	14	59974386	59985200	+	CODING	0.811879	9.77E-10
ENSBTAT00000046700	ENSBTAG00000005339	-	23	17263646	17269998	+	CODING	1.133532	1.00E-09
ENSBTAT00000020018	ENSBTAG00000015041	NDUFA2	7	53461635	53463955	-	CODING	0.527547	1.12E-09
ENSBTAT00000012866	ENSBTAG00000009757	RPSA	22	12729703	12741653	+	CODING	0.390832	1.17E-09
ENSBTAT00000047158	ENSBTAG00000033197	EPDR1	4	50028919	50064257	+	CODING	-1.19679	1.21E-09
ENSBTAT00000040082	ENSBTAG00000016648	BSG	7	44816940	44823914	+	CODING	0.421344	1.24E-09
ENSBTAT00000016013	ENSBTAG00000012072	NDUFS8	29	46205045	46208795	+	CODING	0.696184	1.33E-09
ENSBTAT00000048797	ENSBTAG00000034449	GNG11	3	5454661	5455256	+	CODING	1.060624	1.56E-09
ENSBTAT00000015345	ENSBTAG00000011547	KBTBD12	22	60249057	60288275	-	CODING	-1.11505	1.61E-09
ENSBTAT00000002429	ENSBTAG00000001864	NR4A3	8	65341202	65373695	+	CODING	1.432718	1.64E-09
ENSBTAT00000001303	ENSBTAG00000000985	RAB10	11	73335436	73400455	-	CODING	-0.53947	1.76E-09
ENSBTAT00000007423	ENSBTAG00000039958	GYS1	18	55996079	56012727	-	CODING	-0.46819	1.80E-09
ENSBTAT00000053713	ENSBTAG00000014601	SGCB	6	69526335	69553387	-	CODING	-0.69327	1.88E-09
ENSBTAT00000061251	ENSBTAG00000039684	PTRF	19	43148013	43162165	-	CODING	0.470303	1.94E-09
ENSBTAT00000035014	ENSBTAG00000020701	MEF2C	7	90616543	90784162	-	CODING	-0.86966	2.09E-09
ENSBTAT00000006465	ENSBTAG00000004915	PFN1	19	27081319	27084643	+	CODING	0.519572	2.14E-09
ENSBTAT00000028257	ENSBTAG00000021205	PPAPDC3	11	101502265	101517588	+	CODING	-0.74924	2.17E-09
ENSBTAT00000051159	ENSBTAG00000036659	U2	29	41846574	41846764	-	CODING	3.094043	2.18E-09
ENSBTAT00000054397	ENSBTAG00000009470	CLIC4	2	128710453	128783921	-	CODING	0.852479	2.19E-09
ENSBTAT00000009385	ENSBTAG00000007131	GADL1	22	5258463	5452369	-	CODING	-0.98203	2.20E-09
ENSBTAT00000060985	ENSBTAG00000044017	MSRB3	5	48563806	48743354	-	CODING	-0.52932	2.29E-09
ENSBTAT00000006806	ENSBTAG00000005163	S100A1	3	16812602	16816399	-	CODING	0.456049	2.36E-09
ENSBTAT00000061240	ENSBTAG00000012882	CUL5	15	17857169	17983781	+	CODING	-1.1034	2.38E-09
ENSBTAT00000007241	ENSBTAG00000034506	SNRPG	19	12231223	12231584	+	CODING	0.698457	2.41E-09
ENSBTAT00000015408	ENSBTAG00000011596	SFRS5	10	81860044	81865204	+	CODING	0.919095	2.67E-09
ENSBTAT00000043635	ENSBTAG00000008185	RTN2	18	53514400	53522925	-	CODING	-0.89982	2.67E-09
ENSBTAT00000015695	ENSBTAG00000011825	C7H19orf43	7	13896310	13899366	+	CODING	0.840707	2.78E-09
ENSBTAT00000021397	ENSBTAG00000016079	COX4I1	18	11800134	11807341	+	CODING	0.265024	3.05E-09
ENSBTAT00000009803	ENSBTAG00000007454	RPL10	X	40364802	40367164	+	CODING	0.283171	3.14E-09
ENSBTAT00000000932	ENSBTAG00000000698	MYO18B	17	67768324	68000455	+	CODING	-1.22473	3.30E-09
ENSBTAT00000023912	ENSBTAG00000017970	ZYX	4	107598856	107607834	+	CODING	0.767519	3.50E-09
ENSBTAT00000033843	ENSBTAG00000024407	-	3	19259499	19260488	+	CODING	1.410162	4.27E-09
ENSBTAT00000001069	ENSBTAG00000000808	COX14	5	29952087	29956382	-	CODING	0.608043	4.34E-09
ENSBTAT00000025024	ENSBTAG00000018800	RPS4	X	83532034	83536488	+	CODING	0.503095	4.36E-09
ENSBTAT00000039206	ENSBTAG00000000894	PGK1	X	79282708	79305316	-	CODING	8.772461	4.66E-09
ENSBTAT00000020065	ENSBTAG00000015074	PTGDS	11	106247577	106250629	-	CODING	1.449203	4.73E-09
ENSBTAT00000042953	ENSBTAG00000004542	C9ORF59	11	101477076	101489330	-	CODING	-1.23309	4.84E-09
ENSBTAT00000020701	ENSBTAG00000015582	HMOX1	5	73980776	73987841	+	CODING	0.680373	5.12E-09
ENSBTAT00000046417	ENSBTAG00000016444	FAM134B	20	56747556	56758641	+	CODING	0.78296	5.27E-09

ENSBTAT00000038885	ENSBTAG00000032657	TEAD1	15	40303805	40482346	-	CODING	-1.17167	5.59E-09
ENSBTAT00000000612	ENSBTAG00000000484	HYAL2	22	50592324	50597462	+	CODING	2.198118	5.92E-09
ENSBTAT00000022970	ENSBTAG00000017279	VAPA	24	42332694	42368659	+	CODING	-0.42238	5.99E-09
ENSBTAT00000031908	ENSBTAG00000023416	PPP2R3A	1	134223427	134372331	-	CODING	-0.9549	7.27E-09
ENSBTAT00000015248	ENSBTAG00000011473	MYL9	13	66306260	66314230	+	CODING	0.768668	7.47E-09
ENSBTAT00000024371	ENSBTAG00000031998	CXCL16	19	27249528	27253097	+	CODING	1.599213	8.08E-09
ENSBTAT00000012357	ENSBTAG00000009389	HNRNPH1	7	1593463	1602463	+	CODING	0.376327	8.85E-09
ENSBTAT00000044712	ENSBTAG00000031544	DDIT3	5	56285008	56289214	+	CODING	1.343668	9.10E-09
ENSBTAT00000012183	ENSBTAG00000009246	DPY30	11	14683785	14696398	-	CODING	0.574941	9.15E-09
ENSBTAT00000016153	ENSBTAG00000012177	SNRPD2	18	53681070	53683928	-	CODING	0.702048	9.27E-09
ENSBTAT00000000683	ENSBTAG00000000524	CSTB	1	146555393	146558844	-	CODING	1.32723	9.51E-09
ENSBTAT00000003925	ENSBTAG00000003015	SESN1	9	41666967	41690061	+	CODING	1.643897	9.63E-09
ENSBTAT00000014176	ENSBTAG00000010709	-	15	22665193	22697952	+	CODING	-0.92466	9.67E-09
ENSBTAT00000050390	ENSBTAG00000006242	USP9X	X	107783133	107862518	-	CODING	-1.03114	9.76E-09
ENSBTAT00000026056	ENSBTAG00000019554	FBP2	8	82396095	82438817	-	CODING	-0.28029	9.85E-09
ENSBTAT00000053207	ENSBTAG00000040564	COX7C	10	72298985	72299176	-	CODING	0.437461	1.02E-08
ENSBTAT00000060214	ENSBTAG00000043222	SNORA12	26	21014545	21014692	-	CODING	6.202171	1.04E-08
ENSBTAT00000018681	ENSBTAG00000014060	LSM6	17	12322226	12340189	-	CODING	0.640947	1.07E-08
ENSBTAT00000000950	ENSBTAG00000000711	NDRG1	14	9109762	9165926	+	CODING	1.322473	1.10E-08
ENSBTAT00000015668	ENSBTAG00000011802	COL6A1	1	147404396	147423644	+	CODING	0.753544	1.12E-08
ENSBTAT00000003766	ENSBTAG00000002898	UNC45B	19	15233409	15265756	-	CODING	-0.67181	1.16E-08
ENSBTAT00000065262	ENSBTAG00000047051	-	X	36171714	36172838	+	CODING	0.538551	1.18E-08
ENSBTAT00000024807	ENSBTAG00000018644	PDZRN3	22	28238772	28506578	+	CODING	-0.82284	1.19E-08
ENSBTAT00000018645	ENSBTAG00000014032	EIF3H	14	49733172	49830429	+	CODING	0.422438	1.21E-08
ENSBTAT00000024644	ENSBTAG00000018513	FHL1	X	19777799	19820952	+	CODING	1.212108	1.23E-08
ENSBTAT00000055956	ENSBTAG00000001137	CLTA	8	60849478	60866939	+	CODING	0.892685	1.23E-08
ENSBTAT00000015985	ENSBTAG00000012048	CARM1	7	16571430	16587354	+	CODING	-0.7436	1.26E-08
ENSBTAT00000044743	ENSBTAG00000031558	-	21	38276192	38276344	-	CODING	1.187671	1.34E-08
ENSBTAT00000045455	ENSBTAG00000010153	ANXA3	6	95065256	95136945	+	CODING	0.784305	1.35E-08
ENSBTAT00000054608	ENSBTAG00000046664	TSC22D1	12	14843776	14846181	-	CODING	-0.86107	1.36E-08
ENSBTAT00000034744	ENSBTAG00000015406	ZNF750	19	50475196	50483968	+	CODING	-1.63074	1.36E-08
ENSBTAT00000020229	ENSBTAG00000015204	SMPX	X	128771954	128825159	+	CODING	0.300991	1.39E-08
ENSBTAT00000001567	ENSBTAG00000001183	KLHL33	10	26728622	26738305	+	CODING	-0.98453	1.46E-08
ENSBTAT00000013507	ENSBTAG00000010232	NDUFS5	3	107558702	107564198	-	CODING	0.371889	1.46E-08
ENSBTAT00000001098	ENSBTAG00000000833	TAX1BP3	19	24971932	24977062	-	CODING	1.010091	1.49E-08
ENSBTAT00000004167	ENSBTAG00000003209	BLCAP	13	67114713	67125217	-	CODING	-0.45443	1.50E-08
ENSBTAT00000003999	ENSBTAG00000003072	ACADVL	19	27568181	27573378	+	CODING	0.430558	1.53E-08
ENSBTAT00000015875	ENSBTAG00000011963	RPS19	18	51689627	51697161	-	CODING	0.489734	1.56E-08
ENSBTAT00000021651	ENSBTAG00000016278	RPL30	14	68467563	68470952	+	CODING	0.320373	1.64E-08
ENSBTAT00000006947	ENSBTAG00000005280	ADA	13	73750479	73773983	-	CODING	1.166323	1.70E-08
ENSBTAT00000015810	ENSBTAG00000011918	BLOC1S1	5	57863517	57866922	-	CODING	0.590198	1.73E-08
ENSBTAT00000054074	ENSBTAG00000038652	-	5	74935713	74959662	+	CODING	0.70111	1.75E-08
ENSBTAT00000028499	ENSBTAG00000021378	S100A13	3	16818414	16824125	+	CODING	1.068663	1.81E-08
ENSBTAT00000005549	ENSBTAG00000004237	BTC	6	91430305	91480129	-	CODING	-0.91204	1.86E-08
ENSBTAT00000007115	ENSBTAG00000005408	CLK1	2	89920586	89927819	-	CODING	0.700869	1.88E-08
ENSBTAT00000019643	ENSBTAG00000014764	CD9	5	104505691	104518399	-	CODING	0.942346	1.92E-08
ENSBTAT00000004836	ENSBTAG00000003711	EPAS1	11	28576347	28668899	+	CODING	0.665874	2.15E-08
ENSBTAT00000014894	ENSBTAG00000011215	ACTN4	18	48668482	48741185	+	CODING	0.899076	2.20E-08
ENSBTAT00000013950	ENSBTAG00000010549	IFRD1	4	55872976	55896662	-	CODING	0.777642	2.20E-08
ENSBTAT00000026560	ENSBTAG00000019938	N4BP1	18	17033279	17085940	-	CODING	-1.04228	2.29E-08
ENSBTAT00000023192	ENSBTAG00000017450	KLHL24	1	84062791	84095207	-	CODING	-0.88322	2.38E-08
ENSBTAT00000005592	ENSBTAG00000004269	SGK1	9	73305313	73310869	-	CODING	1.10649	2.54E-08
ENSBTAT00000020036	ENSBTAG00000015053	CFL2	21	45519654	45522305	-	CODING	-0.23496	2.61E-08
ENSBTAT00000024301	ENSBTAG00000018255	ACTN1	10	81023526	81121590	-	CODING	1.386323	2.69E-08
ENSBTAT00000028280	ENSBTAG00000021226	SAR1B	7	47717606	47747828	-	CODING	-0.44636	2.75E-08
ENSBTAT00000023247	ENSBTAG00000017492	PCMTD1	14	22669363	22717576	-	CODING	-0.58742	2.83E-08
ENSBTAT00000016071	ENSBTAG00000012120	TIPARP	1	111830980	111853985	-	CODING	1.325607	3.01E-08
ENSBTAT00000017503	ENSBTAG00000003418	MSN	X	100070406	100162454	-	CODING	0.777248	3.04E-08
ENSBTAT00000006730	ENSBTAG00000005102	PHTF2	4	43549368	43641516	-	CODING	-1.11434	3.12E-08
ENSBTAT00000012523	ENSBTAG00000009517	DBI	2	71561192	71566372	+	CODING	0.484827	3.25E-08
ENSBTAT00000021636	ENSBTAG00000016266	SDHD	15	22722376	22733137	+	CODING	-0.53614	3.28E-08
ENSBTAT00000022929	ENSBTAG00000011022	ARPP19	10	57923246	57937867	+	CODING	-0.56015	3.33E-08
ENSBTAT00000063482	ENSBTAG00000021048	ADM	15	42911789	42913325	-	CODING	2.286614	3.68E-08
ENSBTAT00000025165	ENSBTAG00000018909	-	4	67767893	67899241	-	CODING	1.563616	3.76E-08

ENSBTAT00000016242	ENSBTAG00000012244	TUBA1B	5	30864529	30868119	+	CODING	0.474396	4.00E-08
ENSBTAT00000064257	ENSBTAG00000046184	-	10	43935668	43936221	-	CODING	0.43246	4.14E-08
ENSBTAT00000062708	ENSBTAG00000045275	SCARNA7	1	107819768	107820090	+	CODING	1.327196	4.24E-08
ENSBTAT00000049347	ENSBTAG00000034885	MGC148992	12	11625778	11641053	+	CODING	1.218039	4.29E-08
ENSBTAT00000005998	ENSBTAG00000004564	MBNL1	1	116238831	116394390	-	CODING	-0.51996	4.69E-08
ENSBTAT00000005324	ENSBTAG00000004072	CAPZA2	4	51781849	51836823	-	CODING	-0.50007	4.87E-08
ENSBTAT00000037397	ENSBTAG00000026344	MAFA	14	2426857	2427912	+	CODING	-1.23236	4.98E-08
ENSBTAT00000061351	ENSBTAG00000032477	HECTD1	21	42121456	42196305	-	CODING	-0.70989	5.15E-08
ENSBTAT00000005170	ENSBTAG00000003966	DUSP3	19	44281113	44294691	-	CODING	-0.78273	5.22E-08
ENSBTAT00000016871	ENSBTAG00000012694	UHMK1	3	6975867	6995683	-	CODING	-1.48261	5.25E-08
ENSBTAT00000017647	ENSBTAG00000025853	HOMER1	10	10350692	10484916	-	CODING	-0.87722	5.52E-08
ENSBTAT00000054925	ENSBTAG00000040018	-	28	18349473	18349742	-	CODING	0.627422	5.63E-08
ENSBTAT00000025740	ENSBTAG00000019327	NRAP	26	34339430	34414796	-	CODING	-0.29113	5.78E-08
ENSBTAT00000066191	ENSBTAG00000001546	MGAT1	7	41337278	41349616	-	CODING	1.373401	5.79E-08
ENSBTAT00000004364	ENSBTAG00000003362	HSP90B1	5	67940792	67959532	+	CODING	0.677915	5.88E-08
ENSBTAT00000016154	ENSBTAG00000012178	NR1D1	19	41040926	41048228	-	CODING	-0.9837	6.18E-08
ENSBTAT00000003055	ENSBTAG00000002362	APOLD1	5	97520910	97524789	-	CODING	0.987532	6.50E-08
ENSBTAT00000008072	ENSBTAG00000006135	MAP1LC3A	13	64497254	64498905	+	CODING	0.653284	6.55E-08
ENSBTAT00000015701	ENSBTAG00000011831	SPPL2A	10	59779604	59813156	+	CODING	-0.76643	6.59E-08
ENSBTAT00000027511	ENSBTAG00000020645	GNAI2	22	50670852	50691007	-	CODING	0.958471	6.62E-08
ENSBTAT00000005102	ENSBTAG00000003907	TSPAN14	28	36077428	36109014	+	CODING	0.996775	6.93E-08
ENSBTAT00000006265	ENSBTAG00000004770	SCN4A	19	48794399	48825660	-	CODING	-0.97284	6.98E-08
ENSBTAT00000019994	ENSBTAG00000015018	FSD2	21	23501906	23537791	-	CODING	-1.06537	7.17E-08
ENSBTAT00000001536	ENSBTAG00000001154	DGAT2	15	55940757	55973229	+	CODING	1.267773	7.21E-08
ENSBTAT00000005380	ENSBTAG00000004118	ALAS1	22	49242706	49256766	-	CODING	-0.86597	7.40E-08
ENSBTAT00000015828	ENSBTAG00000011922	PLEC	14	2054917	2088261	+	CODING	-0.76114	7.44E-08
ENSBTAT00000020267	ENSBTAG00000025564	RPL36A	10	42699004	42700655	-	CODING	0.587861	7.45E-08
ENSBTAT00000028615	ENSBTAG00000021467	IGFBP6	5	27044009	27047853	-	CODING	0.900699	7.61E-08
ENSBTAT00000017940	ENSBTAG00000013492	PRKAG3	2	107509452	107516981	-	CODING	-0.90128	8.18E-08
ENSBTAT00000011952	ENSBTAG00000009067	DDX6	15	29887449	29918570	-	CODING	-0.83651	8.68E-08
ENSBTAT00000013537	ENSBTAG00000010244	CLIC5	23	19167358	19333272	-	CODING	-1.06728	9.19E-08
ENSBTAT00000060548	ENSBTAG00000043571	ND2	MT	4266	5307	+	CODING	-0.05296	9.21E-08
ENSBTAT00000019972	ENSBTAG00000014991	PARD3	13	18982015	19337608	+	CODING	-1.15038	9.71E-08
ENSBTAT00000044250	ENSBTAG00000031249	PSMG4	23	50327386	50331704	-	CODING	1.199526	9.86E-08
ENSBTAT00000065271	ENSBTAG00000047561	VEGFA	23	17257357	17261031	-	CODING	0.92614	1.02E-07
ENSBTAT00000008727	ENSBTAG00000006642	PRKACA	7	12693768	12711139	+	CODING	-0.86101	1.08E-07
ENSBTAT00000026769	ENSBTAG00000020093	CUL4A	12	90562966	90589009	+	CODING	-0.57374	1.08E-07
ENSBTAT00000026999	ENSBTAG00000020263	TIMP4	22	57602210	57609085	+	CODING	0.951623	1.10E-07
ENSBTAT00000013399	ENSBTAG00000010155	-	19	14735718	14741999	+	CODING	2.648637	1.12E-07
ENSBTAT00000000742	ENSBTAG00000000569	HES1	1	73974252	73976720	-	CODING	-0.79798	1.14E-07
ENSBTAT00000001447	ENSBTAG00000001093	KLHL23	2	26619585	26636478	-	CODING	-1.30994	1.15E-07
ENSBTAT00000000596	ENSBTAG00000000469	PPP2CA	7	47425980	47450747	-	CODING	0.477317	1.18E-07
ENSBTAT00000007216	ENSBTAG00000005488	ARIH2	22	51572989	51610467	-	CODING	-0.59014	1.29E-07
ENSBTAT00000015705	ENSBTAG00000011834	CCDC47	19	48662435	48682292	-	CODING	-0.58603	1.31E-07
ENSBTAT00000032665	ENSBTAG00000023806	COBL	4	4494964	4728977	+	CODING	-0.7472	1.33E-07
ENSBTAT00000066053	ENSBTAG00000046786	UQCRFS1	18	1021248	1026029	-	CODING	0.283807	1.34E-07
ENSBTAT00000003190	ENSBTAG00000002457	SEC61B	8	64722966	64731269	+	CODING	1.149958	1.35E-07
ENSBTAT00000006196	ENSBTAG00000004723	TEX2	19	49006459	49068544	-	CODING	-0.84071	1.44E-07
ENSBTAT00000002878	ENSBTAG00000002226	FBXO31	18	12974880	13009289	-	CODING	-0.96343	1.47E-07
ENSBTAT00000042695	ENSBTAG00000012066	PECAM1	19	49175892	49238414	-	CODING	0.79039	1.49E-07
ENSBTAT00000011734	ENSBTAG00000008915	SF3B1	2	86349373	86387060	-	CODING	0.598145	1.52E-07
ENSBTAT00000026715	ENSBTAG00000020050	MLEC	17	65184868	65194879	+	CODING	-1.38364	1.55E-07
ENSBTAT00000032427	ENSBTAG00000000605	ATP5J	1	10069680	10076995	+	CODING	0.631235	1.55E-07
ENSBTAT00000045202	ENSBTAG00000031875	BANF1	29	44759486	44761429	+	CODING	0.281895	1.62E-07
ENSBTAT00000009208	ENSBTAG00000006995	SPTBN1	11	37030009	37241384	+	CODING	0.560738	1.63E-07
ENSBTAT00000022319	ENSBTAG00000016779	CLIP1	17	55245237	55360147	+	CODING	-0.57422	1.69E-07
ENSBTAT00000011740	ENSBTAG00000008920	ATP1B4	X	4919686	4940505	-	CODING	-0.75062	1.74E-07
ENSBTAT00000004031	ENSBTAG00000003098	MTDH	14	68799572	68853740	-	CODING	-0.64594	1.79E-07
ENSBTAT00000016093	ENSBTAG00000012125	GDI1	X	40411179	40416941	+	CODING	0.919352	1.84E-07
ENSBTAT00000018778	ENSBTAG00000014130	COX6C	14	66637801	66647721	+	CODING	0.316718	1.98E-07
ENSBTAT00000036839	ENSBTAG00000010012	BTF3	20	8038617	8045160	-	CODING	0.29412	1.98E-07
ENSBTAT00000022579	ENSBTAG00000016977	FUNDC2	X	38815313	38838616	-	CODING	-0.4093	1.99E-07
ENSBTAT00000020159	ENSBTAG00000015154	MCL1	3	20172325	20176960	+	CODING	0.482217	2.01E-07
ENSBTAT00000019649	ENSBTAG00000014771	RBMX2	X	14270247	14282970	+	CODING	1.586462	2.15E-07

ENSBTAT00000020207	ENSBTAG00000015188	KLF6	13	44945068	44952151	+	CODING	0.856772	2.18E-07
ENSBTAT00000024788	ENSBTAG00000018628	RPL29	22	49499798	49501972	+	CODING	0.298411	2.25E-07
ENSBTAT00000007621	ENSBTAG00000005796	-	3	9538550	9576282	+	CODING	-0.67511	2.26E-07
ENSBTAT00000023206	ENSBTAG00000017448	EFEMP1	11	38338744	38408288	-	CODING	1.361691	2.32E-07
ENSBTAT00000043065	ENSBTAG00000002853	HRC	18	56107442	56111943	-	CODING	-0.47625	2.41E-07
ENSBTAT00000030532	ENSBTAG00000018313	MBNL2	12	78283460	78445751	+	CODING	-0.62109	2.44E-07
ENSBTAT00000019230	ENSBTAG00000014463	CAMK2D	6	12965030	13271053	+	CODING	-1.56612	2.56E-07
ENSBTAT00000009699	ENSBTAG00000007375	MIF	17	73273379	73274171	+	CODING	0.812753	2.75E-07
ENSBTAT00000040802	ENSBTAG00000028421	U1	19	15193841	15194001	-	CODING	5.877187	2.95E-07
ENSBTAT00000064895	ENSBTAG00000022808	CACUL1	26	39292197	39309978	-	CODING	-1.84079	2.96E-07
ENSBTAT00000018492	ENSBTAG00000013921	CKM	18	53383534	53392948	-	CODING	-0.06592	2.97E-07
ENSBTAT00000052050	ENSBTAG00000039555	COX7C	7	88648457	88650528	+	CODING	0.500439	3.02E-07
ENSBTAT00000004418	ENSBTAG00000003407	SF3B14	11	75039681	75047800	+	CODING	0.711016	3.03E-07
ENSBTAT00000022876	ENSBTAG00000017212	C21H15orf63	21	55974568	55975823	+	CODING	0.445927	3.06E-07
ENSBTAT00000020564	ENSBTAG00000015474	SFRS11	3	75243969	75293883	-	CODING	1.003918	3.07E-07
ENSBTAT00000004198	ENSBTAG00000003238	MEOX2	4	23943520	24019359	-	CODING	0.987816	3.10E-07
ENSBTAT00000016768	ENSBTAG00000012634	NDUFB7	7	12325362	12329750	+	CODING	0.432942	3.11E-07
ENSBTAT00000016620	ENSBTAG00000012519	XDH	11	14176298	14281717	-	CODING	1.031481	3.13E-07
ENSBTAT00000000566	ENSBTAG00000000442	RBP4	26	14940551	14946750	-	CODING	1.3833	3.17E-07
ENSBTAT00000028392	ENSBTAG00000021308	IRS1	2	115790540	115794253	-	CODING	-1.65125	3.25E-07
ENSBTAT00000026127	ENSBTAG00000019612	RNASE4	10	26423874	26445617	-	CODING	0.865464	3.29E-07
ENSBTAT00000017897	ENSBTAG00000013454	TAB2	9	87759388	87798966	+	CODING	-0.74384	3.47E-07
ENSBTAT00000022854	ENSBTAG00000017196	PDIA3	21	55924230	55946434	+	CODING	0.575363	3.52E-07
ENSBTAT00000036654	ENSBTAG00000003536	42800	20	62940001	62990698	-	CODING	-0.86086	3.64E-07
ENSBTAT00000012735	ENSBTAG00000009663	CSDA	5	99335508	99360895	+	CODING	0.122859	3.72E-07
ENSBTAT00000007028	ENSBTAG00000005345	ARPC3	17	56573543	56584354	-	CODING	0.754449	3.81E-07
ENSBTAT00000032213	ENSBTAG00000012881	EIF4G1	1	83466847	83484338	-	CODING	-0.58646	3.89E-07
ENSBTAT00000019585	ENSBTAG00000014719	TMOD1	8	63180883	63269863	+	CODING	-0.41362	3.91E-07
ENSBTAT00000001086	ENSBTAG00000000820	GNG11	4	11074737	11079756	+	CODING	1.08656	4.04E-07
ENSBTAT00000027111	ENSBTAG00000020342	MYOC	16	39957137	39971282	-	CODING	1.100085	4.29E-07
ENSBTAT00000022534	ENSBTAG00000016943	NEK7	16	79057373	79136394	+	CODING	-1.42518	4.44E-07
ENSBTAT00000026501	ENSBTAG00000019891	MRPL40	17	74708565	74711079	+	CODING	0.680816	4.49E-07
ENSBTAT00000008682	ENSBTAG00000006618	HLF	19	5696798	5747138	+	CODING	-1.58245	4.61E-07
ENSBTAT00000004984	ENSBTAG00000003830	PSMB3	19	39980435	39989908	+	CODING	0.475846	4.63E-07
ENSBTAT00000015617	ENSBTAG00000011758	-	5	104740723	104741272	+	CODING	0.552542	4.63E-07
ENSBTAT00000044086	ENSBTAG00000015273	CAND2	22	56990323	57017221	-	CODING	-0.89664	4.77E-07
ENSBTAT00000044059	ENSBTAG00000031134	YWHAH	17	72629359	72630106	+	CODING	0.528763	4.86E-07
ENSBTAT00000027115	ENSBTAG00000020345	CNN3	3	48763975	48794136	+	CODING	1.189611	5.15E-07
ENSBTAT00000063756	ENSBTAG00000047637	-	11	46404611	46404925	-	CODING	0.394444	5.18E-07
ENSBTAT00000016904	ENSBTAG00000012718	XK	X	111157579	111203561	-	CODING	-1.23215	5.33E-07
ENSBTAT00000049039	ENSBTAG00000005742	CYYR1	1	9242710	9250037	+	CODING	0.748192	5.58E-07
ENSBTAT00000025389	ENSBTAG00000019070	PMP22	19	33357141	33382707	-	CODING	0.752952	5.79E-07
ENSBTAT00000003884	ENSBTAG00000002983	NT5C1A	3	106993389	107008708	+	CODING	-1.53105	5.83E-07
ENSBTAT00000053426	ENSBTAG00000003994	IGFBP3	4	76705105	76712709	+	CODING	0.946263	5.93E-07
ENSBTAT00000003259	ENSBTAG00000002507	ATP5A1	24	46300459	46309366	-	CODING	-0.22827	6.01E-07
ENSBTAT00000023994	ENSBTAG00000018024	NR1D2	27	41866520	41883521	-	CODING	-1.16507	6.04E-07
ENSBTAT00000016504	ENSBTAG00000012432	FDFT1	8	7427461	7453906	-	CODING	-0.61769	6.08E-07
ENSBTAT00000021523	ENSBTAG00000016170	KCNJ11	15	35650715	35653362	+	CODING	-0.93964	6.09E-07
ENSBTAT00000047359	ENSBTAG00000010526	PPAP2A	20	23658802	23793336	+	CODING	0.492378	6.13E-07
ENSBTAT00000063903	ENSBTAG00000047229	CRIP1	21	71390600	71392110	-	CODING	0.940254	6.20E-07
ENSBTAT00000020298	ENSBTAG00000015258	-	19	24983919	24996301	-	CODING	-0.9662	6.34E-07
ENSBTAT00000021123	ENSBTAG00000015892	NDUFB4	1	65922482	65928617	+	CODING	0.382642	6.36E-07
ENSBTAT00000011979	ENSBTAG00000009087	GNG10	8	102784501	102790524	+	CODING	0.517557	6.40E-07
ENSBTAT00000034695	ENSBTAG00000024909	H3F3A	19	56453856	56455637	+	CODING	0.616571	6.41E-07
ENSBTAT00000024848	ENSBTAG00000018671	DCUN1D2	12	90680190	90695973	-	CODING	-0.58435	7.01E-07
ENSBTAT00000004243	ENSBTAG00000003278	HOXC10	5	26202176	26206357	-	CODING	-1.11617	7.31E-07
ENSBTAT00000000086	ENSBTAG00000000078	GLIPR2	8	60797959	60816816	+	CODING	1.3616	7.33E-07
ENSBTAT00000052506	ENSBTAG00000037516	-	13	16262484	16263567	-	CODING	-0.40481	7.81E-07
ENSBTAT00000017425	ENSBTAG00000013109	STIM1	15	51845122	52049569	-	CODING	-0.70681	8.01E-07
ENSBTAT00000008894	ENSBTAG00000006759	SUCLA2	12	17884901	17930933	-	CODING	-0.39879	8.24E-07
ENSBTAT00000065901	ENSBTAG00000047491	CACNA1S	16	81472038	81527931	-	CODING	-0.64225	8.31E-07
ENSBTAT00000065946	ENSBTAG00000019269	COL6A2	1	147570799	147572432	+	CODING	0.93835	8.55E-07
ENSBTAT00000000944	ENSBTAG00000000706	ADAMTS1	1	8955134	8963815	+	CODING	0.767998	9.05E-07
ENSBTAT00000027125	ENSBTAG00000020355	KLF4	8	98843684	98847740	-	CODING	0.615883	9.18E-07

ENSBTAT00000026470	ENSBTAG00000019867	COMMD1	11	60427663	60598437	+	CODING	1.089001	9.51E-07
ENSBTAT00000002103	ENSBTAG00000001604	-	7	2586101	2598115	-	CODING	-0.5324	1.06E-06
ENSBTAT00000014219	ENSBTAG00000010741	KBTBD10	2	26780742	26798046	-	CODING	-0.13407	1.08E-06
ENSBTAT00000028159	ENSBTAG00000033008	MYOZ1	28	29780787	29798613	-	CODING	-0.29212	1.08E-06
ENSBTAT00000022251	ENSBTAG00000016736	HIGD1B	19	45194104	45196727	+	CODING	2.216675	1.14E-06
ENSBTAT00000002600	ENSBTAG00000002006	THBS1	10	35314025	35329297	+	CODING	0.968795	1.14E-06
ENSBTAT00000064703	ENSBTAG00000047621	-	16	4998445	5005553	+	CODING	1.531917	1.16E-06
ENSBTAT00000025582	ENSBTAG00000019210	ADCY2	20	65505171	65653865	-	CODING	-0.57377	1.20E-06
ENSBTAT00000061391	ENSBTAG00000023002	JPH2	13	73303266	73375173	-	CODING	-1.43125	1.21E-06
ENSBTAT00000066024	ENSBTAG00000045548	CRKL	17	74286482	74297800	+	CODING	-0.90304	1.23E-06
ENSBTAT00000027461	ENSBTAG00000020608	ATG9A	2	107892323	107901716	-	CODING	-0.8341	1.25E-06
ENSBTAT00000023566	ENSBTAG00000017719	AKAP6	21	43132861	43630707	+	CODING	-1.5527	1.35E-06
ENSBTAT00000049743	ENSBTAG00000002487	UBE3A	21	2346814	2410193	-	CODING	-0.55025	1.36E-06
ENSBTAT00000032851	ENSBTAG00000002108	YWHAQ	11	87842280	87873674	+	CODING	0.929086	1.40E-06
ENSBTAT00000021045	ENSBTAG00000015839	MAP4	22	52369974	52462511	+	CODING	-0.5713	1.41E-06
ENSBTAT00000024531	ENSBTAG00000018438	RRAGD	9	61644457	61743739	+	CODING	-0.67549	1.41E-06
ENSBTAT00000048868	ENSBTAG00000034496	SHFM1	22	22098224	22098436	-	CODING	0.168066	1.42E-06
ENSBTAT00000020651	ENSBTAG00000015543	ARHGAP35	18	54425390	54497692	+	CODING	-1.10002	1.43E-06
ENSBTAT00000009395	ENSBTAG00000007139	WSB2	17	59422963	59437371	+	CODING	-0.66216	1.44E-06
ENSBTAT00000062920	ENSBTAG00000045889	-	3	28737677	28745359	-	CODING	-1.68384	1.47E-06
ENSBTAT00000005137	ENSBTAG00000003935	RECS1	2	107023827	107041372	-	CODING	0.570495	1.47E-06
ENSBTAT00000007531	ENSBTAG00000005729	FBXL4	9	51485365	51560823	+	CODING	-1.06979	1.47E-06
ENSBTAT00000003575	ENSBTAG00000002758	THBD	13	42217371	42221004	-	CODING	1.122841	1.49E-06
ENSBTAT00000031069	ENSBTAG00000007784	NAA50	1	58847733	58879203	-	CODING	-0.41864	1.53E-06
ENSBTAT00000009702	ENSBTAG00000007378	CLIP4	11	70656465	70733268	-	CODING	-1.23795	1.55E-06
ENSBTAT00000024919	ENSBTAG00000018722	PLEKHM2	16	53075569	53115375	-	CODING	-0.72977	1.56E-06
ENSBTAT00000025506	ENSBTAG00000019164	RHOBTB1	28	16729036	16802909	-	CODING	-1.02989	1.57E-06
ENSBTAT00000018555	ENSBTAG00000013956	BCL2L13	5	109584777	109635907	+	CODING	-0.56562	1.58E-06
ENSBTAT00000061365	ENSBTAG00000021830	ENPP1	9	70734253	70804135	+	CODING	1.051086	1.58E-06
ENSBTAT00000050132	ENSBTAG00000020218	ANXA7	28	29552050	29573976	-	CODING	-0.73337	1.59E-06
ENSBTAT00000065587	ENSBTAG00000047374	-	5	56217962	56220534	+	CODING	1.718545	1.71E-06
ENSBTAT00000006593	ENSBTAG00000005008	WSB1	19	19356951	19371161	+	CODING	0.821155	1.77E-06
ENSBTAT00000045859	ENSBTAG00000000199	PDP1	14	72678594	72686964	-	CODING	-0.91048	1.82E-06
ENSBTAT00000047947	ENSBTAG00000001212	SSR2	3	14808605	14815178	+	CODING	0.585837	1.87E-06
ENSBTAT00000017243	ENSBTAG00000012970	FAM53B	26	44619642	44693891	-	CODING	-1.56128	1.88E-06
ENSBTAT00000018777	ENSBTAG00000014129	RAB5B	5	57648526	57651738	-	CODING	-0.76624	1.89E-06
ENSBTAT00000002319	ENSBTAG00000001771	DYRK1A	1	151370526	151411483	+	CODING	-0.7878	1.91E-06
ENSBTAT00000002096	ENSBTAG00000001603	YIPF7	6	64882684	64916426	-	CODING	-0.81187	1.91E-06
ENSBTAT00000003595	ENSBTAG00000002770	TCN2	17	71711883	71727449	+	CODING	0.871121	1.96E-06
ENSBTAT00000064829	ENSBTAG00000046333	-	6	26345281	26350653	+	CODING	-0.70515	1.98E-06
ENSBTAT00000008076	ENSBTAG00000006138	SEMA3C	4	40140494	40345588	+	CODING	-1.18159	1.99E-06
ENSBTAT00000004179	ENSBTAG00000003220	ACKR1	3	10647427	10648940	-	CODING	1.852061	2.05E-06
ENSBTAT00000007761	ENSBTAG00000005907	NDUFB6	8	11372938	11389199	+	CODING	0.393216	2.06E-06
ENSBTAT00000012495	ENSBTAG00000009495	BCAM	18	52965138	52976445	+	CODING	0.72925	2.08E-06
ENSBTAT00000064442	ENSBTAG00000045582	ARPC5	16	66150415	66159807	-	CODING	0.994221	2.11E-06
ENSBTAT00000043205	ENSBTAG00000030587	LASP1	19	40090995	40131373	+	CODING	1.025184	2.17E-06
ENSBTAT00000028825	ENSBTAG00000021632	MSRA	8	8635550	9019576	-	CODING	-0.39362	2.18E-06
ENSBTAT00000004701	ENSBTAG00000003609	PBDC1	X	80576367	80580439	-	CODING	0.746617	2.22E-06
ENSBTAT00000016756	ENSBTAG00000012622	TRA2A	4	32250996	32269539	-	CODING	0.816609	2.27E-06
ENSBTAT00000021062	ENSBTAG00000015844	TFPI2	4	11035479	11039782	-	CODING	0.679305	2.28E-06
ENSBTAT00000026074	ENSBTAG00000019569	CD151	29	50695518	50700467	-	CODING	0.633545	2.30E-06
ENSBTAT00000021521	ENSBTAG00000016169	ID1	13	61726125	61727283	+	CODING	0.722258	2.33E-06
ENSBTAT00000009492	ENSBTAG00000007215	RNPEPL1	3	120320961	120324657	+	CODING	-0.68478	2.40E-06
ENSBTAT00000012098	ENSBTAG00000009181	INPP5A	26	51126574	51200732	-	CODING	-0.99687	2.45E-06
ENSBTAT00000024750	ENSBTAG00000018596	PTPN21	10	101237134	101308928	-	CODING	-1.05825	2.54E-06
ENSBTAT00000026762	ENSBTAG00000020087	CAMK2A	7	63576976	63641028	-	CODING	-0.98701	2.55E-06
ENSBTAT00000010283	ENSBTAG00000007820	-	7	2568116	2568744	+	CODING	-0.57592	2.60E-06
ENSBTAT00000065236	ENSBTAG00000046309	-	10	73129829	73133979	-	CODING	-1.05141	2.60E-06
ENSBTAT00000025657	ENSBTAG00000019267	MMP2	18	23828638	23855657	+	CODING	0.936165	2.62E-06
ENSBTAT00000017907	ENSBTAG00000013464	CEP97	1	46431094	46452391	+	CODING	-1.30948	2.65E-06
ENSBTAT00000002846	ENSBTAG00000027930	-	2	122169405	122170058	-	CODING	0.976338	2.70E-06
ENSBTAT00000006510	ENSBTAG00000004950	BRN	10	26288223	26289852	+	CODING	1.235368	2.72E-06
ENSBTAT00000029018	ENSBTAG00000021769	CUL3	2	113372323	113487279	-	CODING	-0.60773	2.82E-06
ENSBTAT00000053463	ENSBTAG00000040055	CAB39	2	119340636	119443035	+	CODING	-0.56172	2.88E-06

ENSBTAT00000065691	ENSBTAG00000024958	FAM214A	10	57828793	57888673	+	CODING	-1.45541	2.93E-06
ENSBTAT00000039717	ENSBTAG00000001027	SERPINH1	15	55514945	55525175	+	CODING	0.745107	3.02E-06
ENSBTAT00000044356	ENSBTAG00000002953	TXN	8	101484812	101492588	-	CODING	0.670432	3.06E-06
ENSBTAT00000065244	ENSBTAG00000013479	SLC9A3R2	25	1575649	1589619	+	CODING	1.364454	3.09E-06
ENSBTAT00000021569	ENSBTAG00000016208	TGM2	13	67663138	67697607	-	CODING	0.920782	3.13E-06
ENSBTAT00000052919	ENSBTAG00000018732	HSPA12B	13	51901885	51918849	-	CODING	1.244787	3.40E-06
ENSBTAT00000049560	ENSBTAG00000035083	ATXN7L3B	5	4048888	4052625	+	CODING	-0.88494	3.42E-06
ENSBTAT00000018391	ENSBTAG00000013834	-	7	18960639	18964880	+	CODING	0.648555	3.44E-06
ENSBTAT00000056276	ENSBTAG00000008683	LDHA	29	26543861	26553359	-	CODING	-0.2269	3.52E-06
ENSBTAT00000002033	ENSBTAG00000001553	HNRNPA1	5	25939737	25944246	-	CODING	0.479811	3.57E-06
ENSBTAT00000025832	ENSBTAG00000005146	-	23	28330539	28334072	-	CODING	0.727537	3.76E-06
ENSBTAT00000008216	ENSBTAG00000006262	LIMS2	2	4780176	4819192	+	CODING	0.864071	3.77E-06
ENSBTAT00000012241	ENSBTAG00000009292	TMEM100	19	6237391	6239838	-	CODING	1.610127	3.86E-06
ENSBTAT00000022761	ENSBTAG00000017125	PABPC4	3	107084063	107097489	+	CODING	-0.47216	3.96E-06
ENSBTAT00000014625	ENSBTAG00000011011	SSH2	19	21443600	21499794	-	CODING	-1.16566	4.07E-06
ENSBTAT00000017991	ENSBTAG00000013530	DDAH2	23	27399306	27402825	+	CODING	1.392703	4.10E-06
ENSBTAT00000053382	ENSBTAG00000005542	EPS15	3	95389092	95528688	+	CODING	-0.66763	4.16E-06
ENSBTAT00000005916	ENSBTAG00000025210	COL4A2	12	89112423	89165255	+	CODING	0.87979	4.24E-06
ENSBTAT00000020111	ENSBTAG00000015114	CALR	7	13731958	13735788	-	CODING	0.46933	4.44E-06
ENSBTAT00000027565	ENSBTAG00000020685	-	4	14000446	14001033	+	CODING	0.969964	4.49E-06
ENSBTAT00000014850	ENSBTAG00000011182	PDLIM1	26	16605968	16646619	-	CODING	0.424615	4.51E-06
ENSBTAT00000007436	ENSBTAG00000005660	PSMD2	1	83491699	83501041	-	CODING	-0.51995	4.51E-06
ENSBTAT00000018691	ENSBTAG00000014068	VDAC3	27	36923762	36932879	+	CODING	-0.41539	4.64E-06
ENSBTAT00000060940	ENSBTAG00000015887	FOXJ3	3	104477699	104600121	+	CODING	-0.83976	4.67E-06
ENSBTAT00000054038	ENSBTAG00000006305	AK1	11	98552024	98561576	-	CODING	-0.20773	4.69E-06
ENSBTAT00000015948	ENSBTAG00000012024	SLC29A2	29	45056314	45063665	-	CODING	-0.71848	4.82E-06
ENSBTAT00000046887	ENSBTAG00000000507	NR4A1	5	27977007	27985786	-	CODING	-0.59683	4.97E-06
ENSBTAT00000015530	ENSBTAG00000038107	MAPKAPK2	16	4319154	4365926	+	CODING	-0.45707	4.98E-06
ENSBTAT00000014101	ENSBTAG00000010663	ADAM15	3	15593459	15603312	-	CODING	1.292152	5.00E-06
ENSBTAT00000009693	ENSBTAG00000007371	SCAMP1	10	9369310	9520700	+	CODING	-1.07291	5.11E-06
ENSBTAT00000063570	ENSBTAG00000045702	ZBED6	16	1400457	1403399	+	CODING	-1.33655	5.15E-06
ENSBTAT00000063319	ENSBTAG00000010204	PCMT1	9	88068718	88101696	+	CODING	-0.62096	5.29E-06
ENSBTAT00000040247	ENSBTAG00000016221	AMOT	X	68598671	68657930	-	CODING	-1.1353	5.44E-06
ENSBTAT00000021156	ENSBTAG00000015910	ITGB1	13	20248978	20290982	+	CODING	0.77387	5.61E-06
ENSBTAT00000013008	ENSBTAG00000009863	BHLHE40	22	21492896	21498539	-	CODING	0.406323	5.65E-06
ENSBTAT00000064158	ENSBTAG00000046248	ARPC1B	25	37546485	37559078	-	CODING	1.22932	5.66E-06
ENSBTAT00000019814	ENSBTAG00000014883	GABARAP	19	27586886	27588815	-	CODING	0.241916	5.81E-06
ENSBTAT00000017030	ENSBTAG00000012818	PDLIM5	6	31419270	31567339	-	CODING	-0.20654	5.84E-06
ENSBTAT00000017516	ENSBTAG00000013166	SMYD2	16	70760234	70813706	-	CODING	-0.6458	5.91E-06
ENSBTAT00000053822	ENSBTAG00000015786	LRRC39	3	43251251	43272503	+	CODING	-0.3378	6.02E-06
ENSBTAT00000046362	ENSBTAG00000002608	42980	3	120968024	120988257	+	CODING	0.85002	6.13E-06
ENSBTAT00000024915	ENSBTAG00000018718	DDI2	16	53135568	53168210	-	CODING	-1.47748	6.14E-06
ENSBTAT00000015581	ENSBTAG00000006982	RAB5C	19	42898002	42903081	-	CODING	0.624832	6.20E-06
ENSBTAT00000011818	ENSBTAG00000008978	USP22	19	35728070	35746498	-	CODING	-0.6379	6.21E-06
ENSBTAT00000011109	ENSBTAG00000008443	LARP1	7	67988299	68007480	+	CODING	-0.90372	6.23E-06
ENSBTAT00000015961	ENSBTAG00000012032	PDE4A	7	16176856	16215655	+	CODING	-0.78592	6.33E-06
ENSBTAT00000012394	ENSBTAG00000009417	ZFAND5	8	48892499	48902995	-	CODING	0.230985	6.39E-06
ENSBTAT00000043182	ENSBTAG00000030575	BHLHE41	5	84233719	84236923	+	CODING	-0.7496	6.48E-06
ENSBTAT00000022292	ENSBTAG00000016762	SLC25A12	2	24678582	24777204	+	CODING	-0.76815	6.66E-06
ENSBTAT00000063767	ENSBTAG00000045608	-	28	3273926	3274777	-	CODING	-1.03298	6.68E-06
ENSBTAT00000056537	ENSBTAG00000037571	EBAG9	14	57006997	57020071	-	CODING	-0.586	6.72E-06
ENSBTAT00000020937	ENSBTAG00000015767	RNF10	17	65067805	65101419	+	CODING	-0.41053	6.73E-06
ENSBTAT00000018863	ENSBTAG00000014182	CTNNA1	7	51688098	51880519	+	CODING	0.714519	6.90E-06
ENSBTAT00000017600	ENSBTAG00000013227	SNAI2	14	21577309	21580910	-	CODING	1.806174	7.01E-06
ENSBTAT00000029290	ENSBTAG00000021970	EYA4	9	72661136	72736460	+	CODING	-0.93281	7.01E-06
ENSBTAT00000046355	ENSBTAG00000010611	OCIAD1	6	69144752	69167276	+	CODING	0.35625	7.08E-06
ENSBTAT00000038151	ENSBTAG00000021653	TRIP12	2	118622220	118729375	-	CODING	-0.75993	7.12E-06
ENSBTAT00000018284	ENSBTAG00000013761	STMN1	2	127773927	127779431	+	CODING	1.421415	7.24E-06
ENSBTAT00000017146	ENSBTAG00000012898	RPS27L	10	46946054	46949421	+	CODING	0.656062	7.30E-06
ENSBTAT00000006381	ENSBTAG00000004852	PLEKHO1	3	20519726	20528784	-	CODING	1.215636	7.32E-06
ENSBTAT00000054479	ENSBTAG00000010954	ART3	6	92607226	92755605	+	CODING	-0.59465	7.34E-06
ENSBTAT00000034135	ENSBTAG00000024542	PIGC	16	40785953	40788409	-	CODING	-0.38434	7.38E-06
ENSBTAT00000005283	ENSBTAG00000004041	ANKRD10	12	89304124	89331787	-	CODING	0.639442	7.40E-06
ENSBTAT00000004009	ENSBTAG00000003081	RWDD4A	27	13404609	13412552	-	CODING	-0.66461	7.47E-06

ENSBTAT00000009303	ENSBTAG00000007077	ABHD1	11	72497319	72504279	-	CODING	2.807077	7.58E-06
ENSBTAT00000024011	ENSBTAG00000018037	PSKH1	18	35521165	35534184	+	CODING	-1.14567	7.79E-06
ENSBTAT00000017251	ENSBTAG00000012975	VDAC2	28	31154552	31168822	+	CODING	-0.58976	7.88E-06
ENSBTAT00000010474	ENSBTAG00000007962	ATP9A	13	80167668	80262763	-	CODING	-1.34714	7.94E-06
ENSBTAT00000045971	ENSBTAG00000032402	U1	3	22861821	22861984	-	CODING	5.482637	7.96E-06
ENSBTAT00000000799	ENSBTAG00000000607	ABCF2	4	114587369	114602056	-	CODING	-0.61566	8.06E-06
ENSBTAT00000013035	ENSBTAG00000009886	KDELR3	5	110660391	110673555	+	CODING	1.242688	8.24E-06
ENSBTAT00000027191	ENSBTAG00000020405	NDUFB9	14	17132669	17140075	-	CODING	0.334348	8.32E-06
ENSBTAT00000004227	ENSBTAG00000003263	TBCA	10	8737160	8822375	-	CODING	0.899449	8.34E-06
ENSBTAT00000013078	ENSBTAG00000009914	NDUFS6	20	70986115	70991390	-	CODING	0.428201	8.40E-06
ENSBTAT00000065882	ENSBTAG00000014132	SNED1	3	120791910	120806556	+	CODING	2.611186	8.41E-06
ENSBTAT00000044557	ENSBTAG00000031441	FXYD5	18	46067593	46078935	+	CODING	1.503153	8.62E-06
ENSBTAT00000028307	ENSBTAG00000021245	SPRY1	17	34749102	34753222	-	CODING	0.882494	8.67E-06
ENSBTAT00000035299	ENSBTAG00000020928	ADPRHL1	12	90663887	90676882	-	CODING	-0.68962	8.78E-06
ENSBTAT00000024427	ENSBTAG00000018362	TMEM109	29	37833099	37841873	+	CODING	-0.53131	8.83E-06
ENSBTAT00000004272	ENSBTAG00000003300	MFGE8	21	20889913	20904968	-	CODING	0.505218	8.95E-06
ENSBTAT00000017165	ENSBTAG00000012916	-	8	31871390	31872132	+	CODING	1.31263	9.07E-06
ENSBTAT00000023627	ENSBTAG00000017765	GSTM1	3	33805754	33816442	-	CODING	-0.52098	9.13E-06
ENSBTAT00000020755	ENSBTAG00000015625	DCTN4	7	64076578	64107651	-	CODING	-0.87077	9.17E-06
ENSBTAT00000055483	ENSBTAG00000039662	RSC1A1	16	53131205	53133055	-	CODING	-1.06902	9.22E-06
ENSBTAT00000017211	ENSBTAG00000012957	SERBP1	3	78243808	78257967	+	CODING	-0.35803	9.30E-06
ENSBTAT00000015763	ENSBTAG00000024091	MALL	11	1747909	1780103	+	CODING	1.64968	9.44E-06
ENSBTAT00000013671	ENSBTAG00000010356	RAB12	24	41532521	41541737	+	CODING	-0.26041	9.52E-06
ENSBTAT00000010928	ENSBTAG00000008303	FKBP1A	13	60276502	60303755	+	CODING	0.402556	9.53E-06
ENSBTAT00000047197	ENSBTAG00000033217	TPM3	3	16382706	16403812	+	CODING	0.600527	9.76E-06
ENSBTAT00000043062	ENSBTAG00000030518	ECSCR	7	52346079	52354152	-	CODING	1.053818	1.02E-05
ENSBTAT00000014594	ENSBTAG00000010989	PIK3R1	20	11329104	11409885	-	CODING	-0.74228	1.02E-05
ENSBTAT00000012542	ENSBTAG00000009534	NDUFB10	25	1517626	1520287	+	CODING	0.290128	1.04E-05
ENSBTAT00000026183	ENSBTAG00000019648	GNB2L1	7	41761805	41766598	-	CODING	0.183983	1.05E-05
ENSBTAT00000035307	ENSBTAG00000019964	GAS6	12	90821803	90850163	-	CODING	0.963207	1.05E-05
ENSBTAT00000002634	ENSBTAG00000002033	UBXN2A	11	75101825	75130698	-	CODING	-1.06192	1.06E-05
ENSBTAT00000007943	ENSBTAG00000006045	P4HB	19	51643765	51653754	+	CODING	0.478839	1.06E-05
ENSBTAT00000046331	ENSBTAG00000018071	TMOD4	3	19679350	19683836	+	CODING	-0.16015	1.09E-05
ENSBTAT00000021337	ENSBTAG00000016032	-	11	44707028	44711889	-	CODING	-1.33841	1.10E-05
ENSBTAT00000023967	ENSBTAG00000023289	SLC26A10	5	56214540	56219323	+	CODING	1.466899	1.17E-05
ENSBTAT00000007053	ENSBTAG00000005359	TGFB2	16	22495736	22588280	+	CODING	-1.15853	1.17E-05
ENSBTAT00000023670	ENSBTAG00000017802	THRB	27	41695665	41771738	+	CODING	-1.25549	1.20E-05
ENSBTAT00000015210	ENSBTAG00000011446	SEMA7A	21	34626181	34651240	+	CODING	1.414642	1.21E-05
ENSBTAT00000008734	ENSBTAG00000006646	HRMT1L2	18	56534833	56545053	+	CODING	0.52712	1.22E-05
ENSBTAT00000023487	ENSBTAG00000017662	EEF2K	25	20115008	20165902	+	CODING	-0.64144	1.24E-05
ENSBTAT00000029891	ENSBTAG00000022155	FSTL1	1	65742633	65802423	-	CODING	0.674099	1.25E-05
ENSBTAT00000020904	ENSBTAG00000015743	GMPR	23	40616330	40677006	-	CODING	-0.39599	1.25E-05
ENSBTAT00000026452	ENSBTAG00000019857	OTUD4	17	13286959	13327626	+	CODING	-1.15354	1.29E-05
ENSBTAT00000012670	ENSBTAG00000009621	-	16	41507401	41508463	+	CODING	-0.50873	1.29E-05
ENSBTAT00000020705	ENSBTAG00000015584	BMI1	13	23709778	23712913	+	CODING	-0.65567	1.30E-05
ENSBTAT00000011383	ENSBTAG00000008632	-	25	26389490	26390167	+	CODING	1.150254	1.35E-05
ENSBTAT00000009797	ENSBTAG00000007447	NUDT4	5	23348068	23364470	+	CODING	0.50483	1.35E-05
ENSBTAT00000022710	ENSBTAG00000017077	CTSL	8	84976255	84981470	-	CODING	0.630044	1.37E-05
ENSBTAT00000037657	ENSBTAG00000004048	MCHR1	5	112510411	112513883	+	CODING	-1.63966	1.38E-05
ENSBTAT00000016502	ENSBTAG00000012433	DENND1B	16	78402085	78468912	-	CODING	-1.3327	1.41E-05
ENSBTAT00000021482	ENSBTAG00000016139	TMEM120A	25	34726229	34731680	-	CODING	0.935875	1.41E-05
ENSBTAT00000001948	ENSBTAG00000001489	TUBA1A	5	30821251	30825700	+	CODING	1.126764	1.51E-05
ENSBTAT00000045065	ENSBTAG00000000215	GNB1	16	52106960	52184132	+	CODING	0.606538	1.52E-05
ENSBTAT00000043753	ENSBTAG00000002326	LGALS3	10	67843328	67861113	+	CODING	1.028142	1.55E-05
ENSBTAT00000008170	ENSBTAG00000006222	TFDP2	1	127977930	128027258	+	CODING	-0.95958	1.62E-05
ENSBTAT00000028263	ENSBTAG00000021211	DPT	16	37106633	37143040	-	CODING	0.706279	1.63E-05
ENSBTAT00000015056	ENSBTAG00000011327	OLFML3	3	29506172	29509176	+	CODING	1.265148	1.64E-05
ENSBTAT00000048673	ENSBTAG00000012307	DTNA	24	22521988	22766995	-	CODING	-0.556	1.65E-05
ENSBTAT00000019328	ENSBTAG00000030424	CLEC1A	5	100300781	100321208	+	CODING	1.580736	1.71E-05
ENSBTAT00000000925	ENSBTAG00000000693	UBE2R2	8	76567996	76663653	+	CODING	-0.51033	1.73E-05
ENSBTAT00000024122	ENSBTAG00000018123	FBLN5	21	57153110	57246389	-	CODING	0.964709	1.77E-05
ENSBTAT00000006810	ENSBTAG00000005165	CSGALNACT2	28	13651048	13678154	+	CODING	-0.94773	1.81E-05
ENSBTAT00000007806	ENSBTAG00000005947	PLAU	28	29964983	29971029	+	CODING	1.380115	1.83E-05
ENSBTAT00000013354	ENSBTAG00000010123	APOE	18	53040105	53042792	+	CODING	0.977404	1.85E-05

ENSBTAT00000022919	ENSBTAG00000017246	UBB	19	33853788	33855685	-	CODING	0.212852	1.93E-05
ENSBTAT00000009534	ENSBTAG00000007246	TMEM204	25	1194760	1207350	+	CODING	1.378537	1.97E-05
ENSBTAT00000065245	ENSBTAG00000047326	EDA2R	X	89702603	89709755	+	CODING	-2.65694	1.97E-05
ENSBTAT00000001925	ENSBTAG00000001468	SAMD4A	10	67430991	67516426	+	CODING	-1.1669	1.98E-05
ENSBTAT00000029560	ENSBTAG00000022058	ACACB	17	66102654	66198865	-	CODING	0.630534	1.98E-05
ENSBTAT00000025863	ENSBTAG00000019414	CLK4	7	41100796	41122562	-	CODING	0.614267	2.00E-05
ENSBTAT00000023515	ENSBTAG00000017680	NOS3	4	114375967	114394483	+	CODING	1.505838	2.00E-05
ENSBTAT00000006573	ENSBTAG00000004992	TNPO3	4	93722331	93800500	-	CODING	-0.92878	2.01E-05
ENSBTAT00000027369	ENSBTAG00000020541	PIGY	6	37677099	37679897	+	CODING	0.376069	2.03E-05
ENSBTAT00000053957	ENSBTAG00000039764	IER5	16	63689680	63690654	+	CODING	1.348554	2.14E-05
ENSBTAT00000048956	ENSBTAG00000034560	-	8	11064112	11064777	-	CODING	-0.94823	2.14E-05
ENSBTAT00000014476	ENSBTAG00000010899	TIMP2	19	54079297	54131052	+	CODING	0.568133	2.15E-05
ENSBTAT00000008146	ENSBTAG00000006202	ST13	5	112612771	112636488	-	CODING	-0.3937	2.15E-05
ENSBTAT00000026309	ENSBTAG00000019742	FOXJ2	5	102003895	102023009	+	CODING	-1.1333	2.21E-05
ENSBTAT00000061204	ENSBTAG00000012307	DTNA	24	22445691	22767026	-	CODING	-0.94866	2.22E-05
ENSBTAT00000003563	ENSBTAG00000002747	ABCA5	19	61873253	61919888	+	CODING	-1.25626	2.24E-05
ENSBTAT00000018261	ENSBTAG00000013745	ITGA5	5	25778012	25799053	+	CODING	1.282826	2.30E-05
ENSBTAT00000046532	ENSBTAG00000032764	-	6	323371	323914	-	CODING	0.290505	2.33E-05
ENSBTAT00000024029	ENSBTAG00000018049	MKNK2	7	22787218	22796782	+	CODING	-0.47755	2.35E-05
ENSBTAT00000018083	ENSBTAG00000013598	RSPO2	14	58537071	58653100	+	CODING	-1.07907	2.35E-05
ENSBTAT00000025637	ENSBTAG00000019255	NFE2L2	2	19659540	19692048	+	CODING	0.459128	2.36E-05
ENSBTAT00000000909	ENSBTAG00000000684	ARHGEF10L	2	135557345	135672091	-	CODING	-1.05028	2.39E-05
ENSBTAT00000044563	ENSBTAG00000017816	FXYD1	18	46053083	46057378	+	CODING	0.378595	2.45E-05
ENSBTAT00000042613	ENSBTAG00000019105	NPLOC4	19	51781191	51829647	+	CODING	-0.73182	2.47E-05
ENSBTAT00000036271	ENSBTAG00000012632	TECR	7	12329820	12357348	-	CODING	-0.40717	2.56E-05
ENSBTAT00000063501	ENSBTAG00000014861	SLC20A2	27	36938387	37017424	-	CODING	2.055672	2.57E-05
ENSBTAT00000044787	ENSBTAG00000031598	GBAS	25	27919682	27946564	+	CODING	0.217886	2.61E-05
ENSBTAT00000016378	ENSBTAG00000012344	RPL26	19	28590314	28595213	-	CODING	0.403371	2.65E-05
ENSBTAT00000020501	ENSBTAG00000015426	PDLIM4	7	23443528	23457942	-	CODING	2.188126	2.66E-05
ENSBTAT00000008572	ENSBTAG00000006526	BCL2L1	13	61766806	61817383	-	CODING	0.629709	2.74E-05
ENSBTAT00000019929	ENSBTAG00000014971	SEC61G	22	1445067	1451951	+	CODING	0.370396	2.76E-05
ENSBTAT00000053653	ENSBTAG00000038794	TMEM245	8	100295620	100362311	-	CODING	-1.42529	2.76E-05
ENSBTAT00000008142	ENSBTAG00000006199	DRAP1	29	44694171	44696295	+	CODING	0.331647	2.81E-05
ENSBTAT00000027250	ENSBTAG00000020449	ETFB	18	57795757	57808326	-	CODING	0.461661	2.81E-05
ENSBTAT00000042874	ENSBTAG00000008570	RPS14	7	63761742	63779251	-	CODING	0.254125	2.84E-05
ENSBTAT00000039551	ENSBTAG00000008577	GRSF1	6	87922395	87940543	-	CODING	-0.99695	2.88E-05
ENSBTAT00000028330	ENSBTAG00000021262	SNRPD3	17	73479566	73484880	-	CODING	0.580358	2.94E-05
ENSBTAT00000012541	ENSBTAG00000009533	RPL3L	25	1508568	1515379	-	CODING	0.394409	2.97E-05
ENSBTAT00000020250	ENSBTAG00000015220	AGO4	3	110530154	110558237	-	CODING	-1.40424	3.00E-05
ENSBTAT00000021268	ENSBTAG00000015989	RPS5	18	65845925	65851516	+	CODING	0.252047	3.07E-05
ENSBTAT00000063342	ENSBTAG00000047376	PIN4	X	83607426	83616158	-	CODING	0.472682	3.08E-05
ENSBTAT00000022698	ENSBTAG00000017071	C1QTNF3	20	39760050	39792323	+	CODING	0.911995	3.12E-05
ENSBTAT00000005523	ENSBTAG00000004215	RARRES2	4	113564407	113567496	-	CODING	1.094699	3.15E-05
ENSBTAT00000025126	ENSBTAG00000005349	RPL27A	15	44469327	44472127	-	CODING	0.435644	3.21E-05
ENSBTAT00000053057	ENSBTAG00000032021	RALB	2	72372371	72430777	+	CODING	0.824459	3.27E-05
ENSBTAT00000020433	ENSBTAG00000015375	SH2D3C	11	98435180	98467957	-	CODING	1.458191	3.35E-05
ENSBTAT00000049486	ENSBTAG00000014766	SRSF1	19	9038200	9041927	-	CODING	0.454762	3.52E-05
ENSBTAT00000009760	ENSBTAG00000007421	CDH5	18	34260148	34274741	+	CODING	0.737369	3.60E-05
ENSBTAT00000063874	ENSBTAG00000045550	TSPAN6	X	50904005	50912844	+	CODING	0.968338	3.65E-05
ENSBTAT00000018663	ENSBTAG00000014041	G3BP2	6	92205907	92241546	-	CODING	-0.57501	3.65E-05
ENSBTAT00000065469	ENSBTAG00000011731	PNMT	19	40694079	40695765	+	CODING	2.087282	3.73E-05
ENSBTAT00000024018	ENSBTAG00000018043	LCAT	18	35544370	35547611	-	CODING	1.302961	3.75E-05
ENSBTAT00000021719	ENSBTAG00000016328	SFPQ	3	111140755	111148491	+	CODING	0.554872	3.81E-05
ENSBTAT00000009126	ENSBTAG00000006951	LMO2	15	65394511	65407118	-	CODING	1.019954	3.82E-05
ENSBTAT00000061244	ENSBTAG00000008077	SLC37A4	15	30122001	30127150	-	CODING	-0.83387	3.83E-05
ENSBTAT00000020055	ENSBTAG00000015066	LANCL1	2	98617457	98666531	-	CODING	-0.75573	3.83E-05
ENSBTAT00000009364	ENSBTAG00000007116	ARRDC3	7	93240419	93253094	-	CODING	-0.92287	3.83E-05
ENSBTAT00000004215	ENSBTAG00000019017	IFITM2	11	107191850	107193047	-	CODING	1.588538	3.84E-05
ENSBTAT00000025806	ENSBTAG00000019369	TSPAN4	29	50560713	50570675	-	CODING	1.123468	3.87E-05
ENSBTAT00000006213	ENSBTAG00000004732	SPTB	10	77201411	77273590	-	CODING	-1.10396	3.93E-05
ENSBTAT00000006337	ENSBTAG00000004824	REEP1	11	48389168	48525009	+	CODING	-0.77079	3.95E-05
ENSBTAT00000011972	ENSBTAG00000009080	ITGB6	2	36256325	36348759	+	CODING	-1.55969	4.05E-05
ENSBTAT00000002932	ENSBTAG00000002271	CDADC1	12	18963010	18990944	+	CODING	-0.78314	4.06E-05
ENSBTAT00000008019	ENSBTAG00000006101	PSMD4	3	19598421	19607517	-	CODING	0.701478	4.23E-05

ENSBTAT00000055244	ENSBTAG00000010336	TALDO1	29	50856122	50863474	-	CODING	0.824345	4.28E-05
ENSBTAT00000007933	ENSBTAG00000006039	ARHGDIB	5	95376923	95395815	+	CODING	0.739336	4.36E-05
ENSBTAT00000009128	ENSBTAG00000006950	COPS4	6	99610792	99649394	+	CODING	-0.40373	4.38E-05
ENSBTAT00000015760	ENSBTAG00000011873	KCNE3	15	54587990	54588289	-	CODING	3.479595	4.41E-05
ENSBTAT00000021887	ENSBTAG00000006884	PABPN1	10	21427137	21434016	-	CODING	1.008621	4.41E-05
ENSBTAT00000053663	ENSBTAG00000012890	SLC25A3	5	63086353	63092547	+	CODING	1.075282	4.41E-05
ENSBTAT00000021617	ENSBTAG00000016250	AKTIP	18	21926577	21937955	-	CODING	-0.81219	4.45E-05
ENSBTAT00000002933	ENSBTAG00000002275	PTP4A1	9	512139	516069	+	CODING	-0.49051	4.49E-05
ENSBTAT00000010762	ENSBTAG00000008182	FOSB	18	53501995	53508395	+	CODING	-0.6495	4.50E-05
ENSBTAT00000012028	ENSBTAG00000009127	TSPYL4	9	34889676	34893768	+	CODING	-0.91327	4.53E-05
ENSBTAT00000043384	ENSBTAG00000003253	NPPC	2	120392634	120397261	-	CODING	2.740283	4.64E-05
ENSBTAT00000045370	ENSBTAG00000014401	SORBS3	8	70357693	70384759	+	CODING	0.823262	4.64E-05
ENSBTAT00000018607	ENSBTAG00000014003	MYF6	5	10330469	10331983	+	CODING	0.306223	4.84E-05
ENSBTAT00000002491	ENSBTAG00000003949	RBM39	13	65567208	65592912	-	CODING	0.508964	4.91E-05
ENSBTAT00000065745	ENSBTAG00000047342	-	25	31086700	31086912	-	CODING	1.463046	4.92E-05
ENSBTAT00000022755	ENSBTAG00000017118	FAM8A1	23	39611984	39619623	-	CODING	-0.95859	4.99E-05
ENSBTAT00000000104	ENSBTAG00000000094	ATPAF1	3	100065497	100090042	+	CODING	-0.58608	5.01E-05
ENSBTAT00000005969	ENSBTAG00000004542	C9ORF59	11	101477218	101489266	-	CODING	-4.23989	5.09E-05
ENSBTAT00000014056	ENSBTAG00000018790	BVES	9	45484429	45522649	+	CODING	-0.89217	5.14E-05
ENSBTAT00000065817	ENSBTAG00000024929	PPP1R27	19	51658045	51659508	+	CODING	4.834726	5.28E-05
ENSBTAT00000015290	ENSBTAG00000011505	RABEP1	19	26759902	26870523	-	CODING	-0.69791	5.29E-05
ENSBTAT00000043035	ENSBTAG00000008135	SLIRP	10	89914334	89923167	+	CODING	0.320899	5.36E-05
ENSBTAT00000002868	ENSBTAG00000002216	MYOD1	15	35331401	35334046	-	CODING	-1.21174	5.36E-05
ENSBTAT00000025807	ENSBTAG00000019370	RAB21	X	97425377	97426021	+	CODING	-0.43434	5.50E-05
ENSBTAT00000023255	ENSBTAG00000017496	ATP5I	6	108851271	108852813	-	CODING	0.405137	5.53E-05
ENSBTAT00000027885	ENSBTAG00000020935	HIF1A	10	74095881	74139364	+	CODING	0.933288	5.61E-05
ENSBTAT00000016607	ENSBTAG00000012509	DYRK1B	18	49650522	49658124	-	CODING	-0.81337	5.67E-05
ENSBTAT00000008609	ENSBTAG00000039793	HDGF	3	14130196	14138913	+	CODING	-0.43479	5.70E-05
ENSBTAT00000007527	ENSBTAG00000005726	HNRNPA2B1	4	70198053	70207085	+	CODING	0.222724	5.72E-05
ENSBTAT00000022279	ENSBTAG00000016754	KLHL38	14	17820791	17831087	+	CODING	-0.81964	5.75E-05
ENSBTAT00000053293	ENSBTAG00000037415	COX7B	17	60146304	60146762	+	CODING	0.471966	5.79E-05
ENSBTAT00000014799	ENSBTAG00000011143	KPNA1	1	67476527	67545097	-	CODING	-0.51674	5.82E-05
ENSBTAT00000002200	ENSBTAG00000001687	STC1	8	71854905	71868618	-	CODING	1.48933	6.07E-05
ENSBTAT00000026228	ENSBTAG00000019680	ZYG11B	3	94123286	94197896	-	CODING	-0.75585	6.18E-05
ENSBTAT00000003415	ENSBTAG00000002633	42987	19	55118592	55153750	-	CODING	0.906766	6.21E-05
ENSBTAT00000031330	ENSBTAG00000008827	SPOCK2	28	28304694	28329730	-	CODING	0.992837	6.31E-05
ENSBTAT00000018408	ENSBTAG00000013859	SCYL2	5	64560031	64613964	+	CODING	-0.91072	6.39E-05
ENSBTAT00000054439	ENSBTAG00000022314	EMD	X	40341365	40343613	+	CODING	-2.06743	6.62E-05
ENSBTAT00000064237	ENSBTAG00000046358	PABPC1	14	65816006	65833756	+	CODING	0.409904	6.75E-05
ENSBTAT00000031716	ENSBTAG00000021204	TES	4	52477241	52532254	-	CODING	1.257922	6.79E-05
ENSBTAT00000022307	ENSBTAG00000016771	PLK2	20	20854607	20860584	+	CODING	1.019428	6.80E-05
ENSBTAT00000000573	ENSBTAG00000000448	BDH1	1	72572941	72608810	-	CODING	1.815619	6.81E-05
ENSBTAT00000047298	ENSBTAG00000033284	CHCHD7	14	25052885	25058779	+	CODING	0.483086	6.84E-05
ENSBTAT00000004242	ENSBTAG00000003275	ANK1	27	36295590	36355927	-	CODING	-1.48896	6.86E-05
ENSBTAT00000042554	ENSBTAG00000030169	SUMO2	19	56923424	56931107	+	CODING	0.477258	6.92E-05
ENSBTAT00000047242	ENSBTAG00000005250	BGN	X	39639906	39653687	+	CODING	0.886114	6.99E-05
ENSBTAT00000055279	ENSBTAG00000020630	KCMF1	11	49779932	49811675	-	CODING	-0.33901	7.01E-05
ENSBTAT00000011585	ENSBTAG00000008792	RNASE6	10	26402507	26404000	-	CODING	1.381336	7.27E-05
ENSBTAT00000013117	ENSBTAG00000009942	PLCL2	1	155618262	155833445	+	CODING	-0.77583	7.29E-05
ENSBTAT00000015648	ENSBTAG00000011787	ISCU	17	66685452	66691553	+	CODING	0.376244	7.40E-05
ENSBTAT00000016887	ENSBTAG00000012705	DEGS1	16	28088797	28092872	-	CODING	0.765289	7.49E-05
ENSBTAT00000063958	ENSBTAG00000045729	SMIM20	16	68078643	68078846	+	CODING	-0.94731	7.58E-05
ENSBTAT00000013956	ENSBTAG00000010555	LSMEM1	4	55858087	55868557	-	CODING	-0.71639	7.58E-05
ENSBTAT00000031822	ENSBTAG00000023377	SH3BP5	1	154024457	154103515	-	CODING	-0.58143	7.66E-05
ENSBTAT00000016865	ENSBTAG00000001842	GSTM3	3	33768050	33770824	+	CODING	0.559306	7.79E-05
ENSBTAT00000063822	ENSBTAG00000046521	-	2	93684245	93684415	+	CODING	-4.57991	7.81E-05
ENSBTAT00000012815	ENSBTAG00000009717	FGL2	4	44236254	44240215	+	CODING	0.839714	7.93E-05
ENSBTAT00000022774	ENSBTAG00000017135	CTSS	3	20024302	20047228	+	CODING	1.049389	7.97E-05
ENSBTAT00000040333	ENSBTAG00000027962	-	2	112675322	112675995	+	CODING	-0.32581	8.05E-05
ENSBTAT00000020114	ENSBTAG00000015117	GADD45GIP1	7	13721214	13723683	+	CODING	0.632334	8.09E-05
ENSBTAT00000064026	ENSBTAG00000046996	EPN1	18	62316246	62324320	-	CODING	-0.48164	8.15E-05
ENSBTAT00000020200	ENSBTAG00000015181	EPM2A	9	84028748	84136430	-	CODING	-0.69544	8.18E-05
ENSBTAT00000057091	ENSBTAG00000001004	ESAM	29	28601863	28610589	-	CODING	0.713567	8.21E-05
ENSBTAT00000021458	ENSBTAG00000016125	KCNA7	18	56038883	56041594	-	CODING	-1.3026	8.23E-05

ENSBTAT00000015261	ENSBTAG00000011483	SCARF1	19	23309862	23319829	-	CODING	0.642529	8.23E-05
ENSBTAT00000029269	ENSBTAG00000021953	LARGE	5	72157229	72769395	-	CODING	-0.81238	8.39E-05
ENSBTAT00000000282	ENSBTAG00000000231	BMPR-IA	28	41817915	41875990	+	CODING	-0.85973	8.45E-05
ENSBTAT00000002506	ENSBTAG00000001926	RAD23B	8	98681864	98726100	+	CODING	-0.24757	8.52E-05
ENSBTAT00000063631	ENSBTAG00000040215	EIF4G3	2	132097562	132451412	+	CODING	-0.82663	8.75E-05
ENSBTAT00000005786	ENSBTAG00000004413	RHOBTB3	7	97350408	97406000	+	CODING	-0.75961	8.76E-05
ENSBTAT00000011713	ENSBTAG00000008895	BPGM	4	99223160	99254538	+	CODING	-0.86345	8.77E-05
ENSBTAT00000043199	ENSBTAG00000030584	SLC35A4	7	53424462	53426334	+	CODING	-0.85125	9.06E-05
ENSBTAT00000030224	ENSBTAG00000009210	ZBTB44	29	36988682	37011135	-	CODING	-0.98311	9.10E-05
ENSBTAT00000009044	ENSBTAG00000006883	EIF4B	5	27091609	27115819	-	CODING	-0.32287	9.27E-05
ENSBTAT00000012099	ENSBTAG00000009182	CLCN1	4	107518111	107554736	+	CODING	-0.87897	9.47E-05
ENSBTAT00000023743	ENSBTAG00000017863	SRGN	28	25631917	25647735	+	CODING	1.10245	9.47E-05
ENSBTAT00000025941	ENSBTAG00000019472	GR-A	7	56235966	56350171	-	CODING	-0.9331	9.53E-05
ENSBTAT00000027338	ENSBTAG00000020520	RASD1	19	35423867	35425459	+	CODING	1.632429	9.87E-05
ENSBTAT00000015385	ENSBTAG00000011580	DAG1	22	51187154	51200326	-	CODING	-0.4648	9.97E-05
ENSBTAT00000035139	ENSBTAG00000025121	EMC6	19	24977284	24978133	+	CODING	0.467244	0.000101
ENSBTAT00000053246	ENSBTAG00000006670	ERGIC3	13	65435895	65448951	+	CODING	0.80193	0.000102
ENSBTAT00000004528	ENSBTAG00000003485	MAGOH	3	93571582	93580843	+	CODING	0.675829	0.000103
ENSBTAT00000008066	ENSBTAG00000006130	CLEC14A	21	48843729	48845622	-	CODING	0.726223	0.000104
ENSBTAT00000066124	ENSBTAG00000045828	PTBP1	7	45016275	45026949	+	CODING	0.878633	0.000104
ENSBTAT00000027728	ENSBTAG00000020807	FAU	29	43990460	43991956	-	CODING	0.450974	0.000104
ENSBTAT00000002852	ENSBTAG00000002203	NDUFS2	3	8309350	8318876	-	CODING	-0.29994	0.000105
ENSBTAT00000003236	ENSBTAG00000002490	CHPT1	5	65852754	65882216	+	CODING	-0.5332	0.000105
ENSBTAT00000025896	ENSBTAG00000019443	CTDNEP1	19	27589896	27596032	-	CODING	-0.43643	0.000106
ENSBTAT00000011824	ENSBTAG00000008985	PCBP1	11	68383793	68385266	+	CODING	-0.24241	0.000108
ENSBTAT00000007371	ENSBTAG00000005615	CEACAM1	18	51138290	51157109	+	CODING	0.893601	0.000108
ENSBTAT00000005009	ENSBTAG00000003840	GUCY1B1	17	44506802	44569575	-	CODING	1.048493	0.000109
ENSBTAT00000057062	ENSBTAG00000012341	CSNK2A1	13	61082324	61106498	+	CODING	-0.55732	0.000109
ENSBTAT00000013288	ENSBTAG00000010073	FLOT2	19	20811180	20815216	-	CODING	-0.61546	0.000111
ENSBTAT00000013889	ENSBTAG00000010508	BLVRB	18	50056571	50073609	-	CODING	0.47557	0.000111
ENSBTAT00000020709	ENSBTAG00000015591	SQSTM1	7	1334566	1346051	-	CODING	-0.22636	0.000113
ENSBTAT00000023725	ENSBTAG00000017846	F11R	3	8483556	8508122	+	CODING	0.815608	0.000113
ENSBTAT00000019747	ENSBTAG00000014827	UBQLN1	8	78223814	78279585	-	CODING	-2.50055	0.000115
ENSBTAT00000012812	ENSBTAG00000009711	RPA1	19	23476634	23527009	+	CODING	-0.9375	0.000117
ENSBTAT00000044622	ENSBTAG00000031483	-	29	40214778	40215227	-	CODING	-0.28475	0.000119
ENSBTAT00000005777	ENSBTAG00000004403	CD320	7	18183237	18187953	-	CODING	1.336075	0.000119
ENSBTAT00000013284	ENSBTAG00000010069	EGR1	7	51438727	51442500	+	CODING	0.320461	0.000119
ENSBTAT00000022120	ENSBTAG00000016629	ADCY9	25	3242404	3344129	-	CODING	-1.1879	0.00012
ENSBTAT00000009341	ENSBTAG00000007101	F3	3	49110156	49121034	+	CODING	1.584171	0.00012
ENSBTAT00000021256	ENSBTAG00000015978	ANXA1	8	49624473	49642916	+	CODING	1.174035	0.000122
ENSBTAT00000003959	ENSBTAG00000003043	GNG2	10	44711724	44842190	+	CODING	1.389928	0.000123
ENSBTAT00000015449	ENSBTAG00000011632	MAP1LC3B	18	13017744	13031053	+	CODING	-0.43716	0.000123
ENSBTAT00000026525	ENSBTAG00000019911	COA3	19	43468381	43469490	-	CODING	0.428267	0.000124
ENSBTAT00000019699	ENSBTAG00000014804	HNRPDL	6	98983515	98990087	-	CODING	0.337321	0.000125
ENSBTAT00000023909	ENSBTAG00000017969	CA4	19	13109655	13117426	-	CODING	1.207991	0.000125
ENSBTAT00000020872	ENSBTAG00000015723	CRIPT	11	28903112	28914000	+	CODING	0.58759	0.000127
ENSBTAT00000007921	ENSBTAG00000006027	USP34	11	59791217	59987854	-	CODING	-0.77223	0.000128
ENSBTAT00000026699	ENSBTAG00000020042	KLHL28	21	55179305	55207518	-	CODING	-1.7901	0.00013
ENSBTAT00000020608	ENSBTAG00000015509	NAMPT	4	47597860	47635332	-	CODING	-0.3463	0.000132
ENSBTAT00000037140	ENSBTAG00000020439	BUD31	25	37524461	37532400	-	CODING	0.506821	0.000133
ENSBTAT00000024049	ENSBTAG00000018063	SPATA2	13	78712288	78722796	-	CODING	-1.21	0.000133
ENSBTAT00000042645	ENSBTAG00000030209	ARHGDIA	19	51633694	51637708	+	CODING	0.529283	0.000133
ENSBTAT00000020877	ENSBTAG00000015727	IFI47	7	41823772	41840054	-	CODING	1.219795	0.000136
ENSBTAT00000015769	ENSBTAG00000011885	NNT	20	31171388	31258005	-	CODING	-0.39354	0.000138
ENSBTAT00000061429	ENSBTAG00000044046	SGCD	7	69585066	70027839	+	CODING	-0.67494	0.000138
ENSBTAT00000001785	ENSBTAG00000001356	USP38	17	14889392	14921767	-	CODING	-0.62207	0.000138
ENSBTAT00000001747	ENSBTAG00000001324	SLCO2A1	1	136435602	136528516	+	CODING	1.408906	0.000139
ENSBTAT00000035225	ENSBTAG00000004910	SLC25A11	19	27089299	27092026	+	CODING	-0.34547	0.00014
ENSBTAT00000016614	ENSBTAG00000012514	PODN	3	93728705	93751305	-	CODING	0.8384	0.00014
ENSBTAT00000044652	ENSBTAG00000016598	DYNC1H1	21	68507134	68568024	+	CODING	-0.78305	0.00014
ENSBTAT00000014665	ENSBTAG00000011045	MRPS36	20	10390982	10399140	-	CODING	-0.522	0.000141
ENSBTAT00000013990	ENSBTAG00000010587	SH3BGRL	X	70399116	70527059	-	CODING	0.83202	0.000142
ENSBTAT00000061473	ENSBTAG00000044185	SOX6	15	36600420	37082361	+	CODING	-1.34043	0.000144
ENSBTAT00000012497	ENSBTAG00000010125	STAT5B	19	42960226	42996671	-	CODING	-0.70072	0.000144

ENSBTAT00000023094	ENSBTAG00000017368	YBX1	3	104112934	104133708	-	CODING	0.157581	0.000146
ENSBTAT00000015544	ENSBTAG00000011704	-	13	63150593	63151022	+	CODING	0.801708	0.000147
ENSBTAT00000014152	ENSBTAG00000010696	DVL1	16	52431328	52440427	+	CODING	-0.68604	0.000149
ENSBTAT00000049176	ENSBTAG00000026586	PPP1R14C	9	88384683	88500749	+	CODING	-0.75565	0.00015
ENSBTAT00000002861	ENSBTAG00000002210	LAS1L	X	100346827	100366716	+	CODING	0.752513	0.000151
ENSBTAT00000017775	ENSBTAG00000013363	CAP1	3	106638795	106667878	-	CODING	0.850005	0.000151
ENSBTAT00000034441	ENSBTAG00000009748	MFRP	15	30450602	30455935	-	CODING	1.934652	0.000151
ENSBTAT00000066011	ENSBTAG00000000605	ATP5J	1	10069815	10076995	+	CODING	0.193797	0.000152
ENSBTAT00000001702	ENSBTAG00000001294	PPP1R15A	18	55925891	55929294	+	CODING	0.671558	0.000153
ENSBTAT00000002626	ENSBTAG00000002026	RPL5	3	50787385	50795965	-	CODING	0.408803	0.000156
ENSBTAT00000021532	ENSBTAG00000037640	PID1	2	117836412	118104185	-	CODING	1.022537	0.000156
ENSBTAT00000054061	ENSBTAG00000002624	PSTPIP2	24	46196113	46290465	-	CODING	-0.42347	0.000157
ENSBTAT00000036100	ENSBTAG00000000527	ZFAND3	23	11712802	12040961	+	CODING	-0.53671	0.000157
ENSBTAT00000007204	ENSBTAG00000005477	LAPTM5	2	123350420	123376541	+	CODING	0.873515	0.000158
ENSBTAT00000032005	ENSBTAG00000023472	PPP1R14A	18	48282679	48286857	-	CODING	1.418249	0.000158
ENSBTAT00000024326	ENSBTAG00000018278	ATP5O	1	922635	929992	+	CODING	0.205635	0.000166
ENSBTAT00000043067	ENSBTAG00000030520	PROB1	7	52290536	52293580	-	CODING	-1.00913	0.000168
ENSBTAT00000029742	ENSBTAG00000012450	RAPGEF2	17	40483943	40580445	-	CODING	1.121	0.000168
ENSBTAT00000006653	ENSBTAG00000005043	TIMP1	X	91232235	91236073	+	CODING	1.018423	0.000171
ENSBTAT00000019298	ENSBTAG00000014518	RPL9	6	60210361	60215120	-	CODING	0.396161	0.000171
ENSBTAT00000013685	ENSBTAG00000010368	TPST2	17	68445522	68499477	-	CODING	0.997939	0.000174
ENSBTAT00000006167	ENSBTAG00000018137	A2M	5	101298127	101346611	+	CODING	0.609918	0.000174
ENSBTAT00000029271	ENSBTAG00000021955	NPC2	10	86170653	86179237	-	CODING	0.615161	0.000176
ENSBTAT00000015625	ENSBTAG00000011767	MPP5	10	79536643	79579325	+	CODING	-0.92876	0.000176
ENSBTAT00000065888	ENSBTAG00000047299	DGCR6L	17	74049772	74053881	+	CODING	0.602028	0.000177
ENSBTAT00000021336	ENSBTAG00000016033	HOXD9	2	20824235	20825910	-	CODING	-1.10184	0.000182
ENSBTAT00000020550	ENSBTAG00000015457	FGFR1	27	33250534	33291989	-	CODING	1.126646	0.000187
ENSBTAT00000024202	ENSBTAG00000018185	ASB4	4	12597838	12699701	+	CODING	-1.10098	0.000189
ENSBTAT00000056173	ENSBTAG00000022396	SAA3	29	26668125	26671801	-	CODING	1.656558	0.00019
ENSBTAT00000008372	ENSBTAG00000025274	TUBB4B	11	105860110	105862549	-	CODING	-0.35899	0.000191
ENSBTAT00000002051	ENSBTAG00000001568	PPIC	7	32114445	32128245	+	CODING	1.196451	0.000192
ENSBTAT00000028364	ENSBTAG00000021288	PSMB4	3	19412241	19414600	-	CODING	0.288334	0.000195
ENSBTAT00000000109	ENSBTAG00000000099	CERS2	3	19835017	19843638	+	CODING	0.749879	0.000198
ENSBTAT00000027662	ENSBTAG00000020757	PCBP2	5	26702879	26723947	-	CODING	-0.29873	0.000198
ENSBTAT00000034054	ENSBTAG00000024503	FCER1G	3	8305544	8308776	-	CODING	1.412139	0.000199
ENSBTAT00000011762	ENSBTAG00000008935	PFDN5	5	26886091	26890186	-	CODING	0.428775	0.0002
ENSBTAT00000024320	ENSBTAG00000018272	RERE	16	45621457	45879645	+	CODING	-0.59574	0.000201
ENSBTAT00000063057	ENSBTAG00000046335	-	29	272264	273010	+	CODING	0.221735	0.000201
ENSBTAT00000010858	ENSBTAG00000008248	DMD	X	117442266	117604200	+	CODING	-0.98656	0.000204
ENSBTAT00000026053	ENSBTAG00000019552	PGRMC1	X	3468827	3477212	+	CODING	1.120076	0.000204
ENSBTAT00000013075	ENSBTAG00000009906	DDX1	11	82824017	82855964	-	CODING	-0.57835	0.000206
ENSBTAT00000003307	ENSBTAG00000002551	KCNJ8	5	88852393	88860007	+	CODING	1.078623	0.000206
ENSBTAT00000010711	ENSBTAG00000008150	PKIA	14	43880469	43978277	+	CODING	-0.24122	0.000209
ENSBTAT00000012977	ENSBTAG00000009844	CYR61	3	58678779	58681686	-	CODING	0.346223	0.00021
ENSBTAT00000019407	ENSBTAG00000014581	MLIP	23	6157657	6283325	-	CODING	-0.43053	0.000216
ENSBTAT00000050399	ENSBTAG00000035907	DDX3X	X	107678777	107691829	-	CODING	-0.34144	0.00022
ENSBTAT00000023937	ENSBTAG00000017992	CD164	9	41246268	41256989	+	CODING	-0.40323	0.000222
ENSBTAT00000001246	ENSBTAG00000000941	PLEKHB2	2	1850107	1899289	+	CODING	-0.47938	0.000224
ENSBTAT00000064578	ENSBTAG00000048287	-	X	140308768	140315911	+	CODING	-4.4081	0.000224
ENSBTAT00000019637	ENSBTAG00000014759	-	5	95536891	95549925	+	CODING	-0.41397	0.000224
ENSBTAT00000011330	ENSBTAG00000008591	CAMTA2	19	27054328	27067739	+	CODING	-0.69564	0.000226
ENSBTAT00000025800	ENSBTAG00000019366	POLR2B	6	74027219	74071144	+	CODING	-0.60636	0.000227
ENSBTAT00000015166	ENSBTAG00000011412	LAMB1	4	49263419	49339822	-	CODING	1.04709	0.000228
ENSBTAT00000011670	ENSBTAG00000008864	MD2	14	39369425	39402263	+	CODING	1.799375	0.000228
ENSBTAT00000004401	ENSBTAG00000003395	ZRANB1	26	44841835	44885237	+	CODING	-0.66173	0.000237
ENSBTAT00000029279	ENSBTAG00000021960	GPX8	20	23975640	23980445	-	CODING	0.934654	0.000238
ENSBTAT00000020468	ENSBTAG00000015402	GREB1	11	86199420	86268193	-	CODING	-2.22667	0.000243
ENSBTAT00000013153	ENSBTAG00000009976	-	2	84413662	84414878	-	CODING	-0.63636	0.000244
ENSBTAT00000015071	ENSBTAG00000011333	CLASP2	22	7809077	7982050	-	CODING	-0.76766	0.000244
ENSBTAT00000020612	ENSBTAG00000015511	FADS3	29	41087531	41102449	-	CODING	1.209211	0.000244
ENSBTAT00000061158	ENSBTAG00000010786	TACC2	26	42323743	42485654	+	CODING	-0.61591	0.00025
ENSBTAT00000061414	ENSBTAG00000007635	PLCL1	2	86718341	87086748	+	CODING	-1.31005	0.00025
ENSBTAT00000026036	ENSBTAG00000019543	TCEB2	25	2281945	2285931	+	CODING	0.392917	0.000252
ENSBTAT00000026726	ENSBTAG00000020061	KCNJ12	19	35955796	35991013	+	CODING	-0.89187	0.000252

ENSBTAT00000023976	ENSBTAG00000018013	EMP3	18	55482512	55486850	+	CODING	0.860786	0.000252
ENSBTAT00000000802	ENSBTAG00000000609	-	1	54654216	54654795	-	CODING	0.342473	0.000254
ENSBTAT00000014028	ENSBTAG00000010611	OCIAD1	6	69144344	69167282	+	CODING	-0.97059	0.000254
ENSBTAT00000005240	ENSBTAG00000004014	FBLN2	22	58990282	59038424	-	CODING	0.909368	0.000254
ENSBTAT00000011505	ENSBTAG00000008731	PRDX3	26	39672044	39681392	-	CODING	-0.29891	0.000255
ENSBTAT00000053143	ENSBTAG00000038351	-	5	441593	442266	-	CODING	-4.38457	0.000256
ENSBTAT00000029119	ENSBTAG00000021845	STRN3	21	41964003	42070978	-	CODING	-0.59	0.000258
ENSBTAT00000005987	ENSBTAG00000004558	MGC165862	10	65243195	65246838	-	CODING	1.820078	0.000258
ENSBTAT00000026631	ENSBTAG00000019994	-	2	91685397	91686142	-	CODING	0.606105	0.000262
ENSBTAT00000023068	ENSBTAG00000017352	ZC3H11A	16	1404592	1440704	+	CODING	-0.54744	0.000266
ENSBTAT00000025388	ENSBTAG00000019069	SYNGR2	19	54611056	54614829	-	CODING	-0.34386	0.000266
ENSBTAT00000063708	ENSBTAG00000047694	-	21	55966632	55968362	+	CODING	0.181112	0.00027
ENSBTAT00000060978	ENSBTAG00000010664	PRKCQ	13	17093614	17185063	+	CODING	-0.69537	0.00027
ENSBTAT00000032460	ENSBTAG00000001852	BREH1	18	24827700	24854528	+	CODING	1.183253	0.000274
ENSBTAT00000066170	ENSBTAG00000045877	TSC22D3	X	59707292	59772737	+	CODING	-0.51415	0.000275
ENSBTAT00000066178	ENSBTAG00000048280	C12orf73	5	67960359	67967214	-	CODING	0.662477	0.000281
ENSBTAT00000000795	ENSBTAG00000000603	JAM2	1	10083035	10165613	-	CODING	0.749189	0.000282
ENSBTAT00000017386	ENSBTAG00000013076	SNX19	29	37526201	37555971	-	CODING	-1.12444	0.000284
ENSBTAT00000044459	ENSBTAG00000008013	LRRC2	22	53448776	53486047	+	CODING	-0.39027	0.000285
ENSBTAT00000029239	ENSBTAG00000021932	RNASEK	19	27436503	27438226	+	CODING	0.315739	0.000286
ENSBTAT00000019232	ENSBTAG00000014465	SERPINE1	25	36198560	36206859	-	CODING	0.879962	0.000288
ENSBTAT00000015064	ENSBTAG00000011337	ANKRD33B	20	62725184	62820226	-	CODING	-1.56048	0.000289
ENSBTAT00000008327	ENSBTAG00000006346	DAP	20	62630506	62697437	+	CODING	0.909118	0.00029
ENSBTAT00000026218	ENSBTAG00000019672	-	23	40609760	40611238	+	CODING	-1.11227	0.000293
ENSBTAT00000031972	ENSBTAG00000014960	SCHIP1	1	108383680	108454122	-	CODING	-0.30015	0.000294
ENSBTAT00000008268	ENSBTAG00000006296	VMA21	X	34102420	34112798	+	CODING	-0.60285	0.000295
ENSBTAT00000036795	ENSBTAG00000012442	CTSB	8	7414945	7423429	+	CODING	0.372457	0.000295
ENSBTAT00000001560	ENSBTAG00000001176	LRRN1	22	22576210	22578360	-	CODING	-0.81236	0.000297
ENSBTAT00000024401	ENSBTAG00000018340	-	8	15978603	15979256	-	CODING	0.599443	0.000299
ENSBTAT00000001642	ENSBTAG00000001244	PLAT	27	36738833	36762837	-	CODING	1.49972	0.000301
ENSBTAT00000017489	ENSBTAG00000013153	NF2	17	70895108	70966968	+	CODING	-0.87981	0.000304
ENSBTAT00000014072	ENSBTAG00000010645	BOLA-DRA	23	25587526	25592227	-	CODING	0.457409	0.000305
ENSBTAT00000001706	ENSBTAG00000001296	TMEM50A	2	128256214	128272007	-	CODING	0.574328	0.00031
ENSBTAT00000030941	ENSBTAG00000034185	EEF1A1	18	2952235	2954439	+	CODING	0.856475	0.000311
ENSBTAT00000025590	ENSBTAG00000019217	KIF13A	23	39404303	39501741	+	CODING	-1.17632	0.000313
ENSBTAT00000002779	ENSBTAG00000002144	ADRB2	7	62229186	62230442	+	CODING	0.900152	0.000319
ENSBTAT00000002488	ENSBTAG00000011571	ACIN1	10	21626432	21637359	+	CODING	1.35522	0.00032
ENSBTAT00000013963	ENSBTAG00000010562	CD34	16	77367502	77389361	+	CODING	0.512141	0.000322
ENSBTAT00000056243	ENSBTAG00000040602	-	2	131410548	131414758	-	CODING	0.353482	0.000322
ENSBTAT00000054382	ENSBTAG00000008636	PDE4B	3	79284893	79304824	-	CODING	-0.71102	0.000326
ENSBTAT00000017835	ENSBTAG00000013406	CSRP2	5	6266018	6274473	-	CODING	1.228501	0.00033
ENSBTAT00000025662	ENSBTAG00000019274	ATIC	2	103847180	103870755	+	CODING	-0.51359	0.00033
ENSBTAT00000010670	ENSBTAG00000008113	OSR1	11	79487139	79494238	+	CODING	2.625019	0.000331
ENSBTAT00000000160	ENSBTAG00000000139	SETD8	17	54457612	54476577	-	CODING	-0.75535	0.000333
ENSBTAT00000009464	ENSBTAG00000007193	CCL16	19	14782701	14786690	+	CODING	1.106889	0.000335
ENSBTAT00000003826	ENSBTAG00000002939	FURIN	21	22206517	22213761	-	CODING	-0.8056	0.000337
ENSBTAT00000018239	ENSBTAG00000013724	ATG4A	X	60973684	61051097	+	CODING	-0.68368	0.000339
ENSBTAT00000001315	ENSBTAG00000000993	N4BP2L1	12	28612076	28634685	+	CODING	1.022333	0.00034
ENSBTAT00000011931	ENSBTAG00000009055	RNF144B	23	38952210	39043167	-	CODING	-0.46928	0.000341
ENSBTAT00000056211	ENSBTAG00000038241	REPIN1	4	113602367	113604046	+	CODING	-0.87932	0.000341
ENSBTAT00000039850	ENSBTAG00000008310	TMEM9B	15	44215628	44234314	+	CODING	-0.38288	0.000343
ENSBTAT00000026869	ENSBTAG00000020174	HBS1L	9	74011379	74096501	-	CODING	-0.43578	0.000346
ENSBTAT00000055432	ENSBTAG00000031800	PPDPF	13	54608180	54609543	-	CODING	0.204454	0.000347
ENSBTAT00000024017	ENSBTAG00000018041	ACADSB	26	43134415	43184345	+	CODING	-0.58294	0.000349
ENSBTAT00000015490	ENSBTAG00000011666	THRSP	29	18084681	18090595	-	CODING	1.463551	0.000349
ENSBTAT00000025878	ENSBTAG00000019427	KIAA0232	6	118129452	118178266	+	CODING	-0.78749	0.000352
ENSBTAT00000019706	ENSBTAG00000014807	DPP8	10	12321152	12369941	-	CODING	-0.89613	0.000354
ENSBTAT00000064455	ENSBTAG00000046467	PTP4A3	14	3578346	3586015	-	CODING	-0.36045	0.000356
ENSBTAT00000047653	ENSBTAG00000008832	CCL1	19	16110980	16114069	+	CODING	1.799804	0.000358
ENSBTAT00000020833	ENSBTAG00000015690	PLIN4	7	20831528	20841683	+	CODING	-0.7019	0.000363
ENSBTAT00000001061	ENSBTAG00000000802	LYVE1	15	42678184	42692464	+	CODING	1.520556	0.000368
ENSBTAT00000018109	ENSBTAG00000013623	TIMM13	7	22448900	22450073	+	CODING	0.513276	0.000369
ENSBTAT00000011408	ENSBTAG00000008652	DUSP27	3	1656685	1723930	-	CODING	-0.42416	0.000372
ENSBTAT00000013065	ENSBTAG00000009899	CHID1	29	50539408	50558480	+	CODING	3.059027	0.000374

ENSBTAT00000008960	ENSBTAG00000006815	-	15	48250683	48251648	-	CODING	0.84511	0.000378
ENSBTAT00000034126	ENSBTAG00000024539	SPSB1	16	44994737	45006429	-	CODING	1.24023	0.000379
ENSBTAT00000002583	ENSBTAG00000030301	MAP7D1	3	110233982	110243621	-	CODING	-0.56706	0.00038
ENSBTAT00000057198	ENSBTAG00000008001	NUCKS1	16	3301673	3329235	-	CODING	-0.45179	0.000383
ENSBTAT00000010169	ENSBTAG00000047834	EFR3A	14	10171320	10250757	-	CODING	-0.64188	0.000385
ENSBTAT00000010553	ENSBTAG00000008025	UBE3C	4	119247415	119362609	+	CODING	-0.68732	0.000391
ENSBTAT00000006033	ENSBTAG00000004599	TAGLN3	1	57178554	57192433	+	CODING	1.258083	0.000391
ENSBTAT00000063284	ENSBTAG00000048122	CFD	7	45030310	45032845	+	CODING	1.084432	0.000399
ENSBTAT00000014734	ENSBTAG00000025666	RPS29	10	26814899	26815069	-	CODING	-4.30413	0.000402
ENSBTAT00000004568	ENSBTAG00000003514	HSF4	18	34922999	34927749	+	CODING	1.076801	0.000415
ENSBTAT00000066041	ENSBTAG00000046533	RAB31	24	42256081	42305185	+	CODING	-0.94782	0.000416
ENSBTAT00000022078	ENSBTAG00000016596	PFDN1	7	53119765	53193428	-	CODING	0.264323	0.000418
ENSBTAT00000052388	ENSBTAG00000005434	PLVAP	7	5685231	5704120	+	CODING	1.3702	0.000422
ENSBTAT00000008406	ENSBTAG00000006411	THAP11	18	35472716	35474389	+	CODING	-0.58302	0.000423
ENSBTAT00000005930	ENSBTAG00000004514	RAF1	22	57122412	57204951	+	CODING	-0.48606	0.000424
ENSBTAT00000028798	ENSBTAG00000030258	CDC42EP1	5	109925709	109933537	+	CODING	0.817838	0.000431
ENSBTAT00000023612	ENSBTAG00000017755	EWSR1	17	70689624	70715166	+	CODING	0.547902	0.000433
ENSBTAT00000001723	ENSBTAG00000001305	ATP2B2	22	55001027	55302080	-	CODING	-1.92313	0.000434
ENSBTAT00000025384	ENSBTAG00000019067	TMC8	19	54633144	54642106	-	CODING	2.312732	0.000438
ENSBTAT00000037914	ENSBTAG00000026624	LYRM5	5	85210770	85217660	-	CODING	0.473099	0.000443
ENSBTAT00000018406	ENSBTAG00000013858	C25H16ORF72	25	7931600	7955194	+	CODING	-0.45379	0.000446
ENSBTAT00000011388	ENSBTAG00000008635	SULT1A1	25	26381308	26385156	-	CODING	0.757077	0.000454
ENSBTAT00000018484	ENSBTAG00000013919	BOLA-DRB3	23	25458594	25476944	+	CODING	0.478981	0.000457
ENSBTAT00000014796	ENSBTAG00000011140	FAM162A	1	67422932	67443491	+	CODING	0.312893	0.000464
ENSBTAT00000013264	ENSBTAG00000030529	FAM53C	7	51349485	51358857	+	CODING	-0.67223	0.000464
ENSBTAT00000009992	ENSBTAG00000007596	GEM	14	72386751	72399660	+	CODING	1.400678	0.000468
ENSBTAT00000015431	ENSBTAG00000011619	RASIP1	18	55810633	55824241	-	CODING	0.813275	0.000469
ENSBTAT00000017366	ENSBTAG00000013060	IQGAP1	21	22530902	22614701	-	CODING	0.739168	0.00047
ENSBTAT00000003229	ENSBTAG00000002485	TNS1	2	106627006	106773157	-	CODING	-0.54197	0.000473
ENSBTAT00000007185	ENSBTAG00000005462	FXR2	19	27932717	27940208	-	CODING	-0.51747	0.000476
ENSBTAT00000000705	ENSBTAG00000000542	CCDC104	11	38066011	38095813	+	CODING	0.620195	0.000482
ENSBTAT00000004350	ENSBTAG00000003352	-	23	25423551	25424597	+	CODING	1.158653	0.000484
ENSBTAT00000007833	ENSBTAG00000005967	UBR2	23	16264297	16370157	+	CODING	-0.70915	0.000491
ENSBTAT00000014100	ENSBTAG00000010652	PDE4C	7	4927816	4939026	+	CODING	-0.98285	0.000506
ENSBTAT00000024364	ENSBTAG00000018310	SETD3	21	66064391	66125305	-	CODING	-0.61522	0.000525
ENSBTAT00000021895	ENSBTAG00000016471	PDPR	18	1910952	1951890	-	CODING	-1.26169	0.000526
ENSBTAT00000013292	ENSBTAG00000010082	COL15A1	8	64437276	64540739	+	CODING	0.698599	0.00053
ENSBTAT00000019242	ENSBTAG00000014471	-	8	101361501	101406330	+	CODING	0.673484	0.000531
ENSBTAT00000016423	ENSBTAG00000012377	ECHDC3	13	12576391	12590757	-	CODING	-0.90402	0.000531
ENSBTAT00000021641	ENSBTAG00000016269	ME2	24	50870262	50928290	+	CODING	-0.64153	0.000536
ENSBTAT00000064131	ENSBTAG00000047717	FAM222B	19	20734088	20786818	-	CODING	-1.07844	0.000539
ENSBTAT00000042889	ENSBTAG00000004556	SLC2A3	5	101896622	101909500	-	CODING	0.650911	0.000539
ENSBTAT00000008644	ENSBTAG00000006579	P4HA3	15	54418559	54459500	-	CODING	1.868391	0.00054
ENSBTAT00000006307	ENSBTAG00000004803	NTAN1	25	13809385	13823824	+	CODING	0.450606	0.00054
ENSBTAT00000021871	ENSBTAG00000016450	CDNF	13	29756747	29778409	-	CODING	-0.76273	0.000543
ENSBTAT00000044296	ENSBTAG00000017616	ADSSL1	21	70845971	70861972	+	CODING	-0.37369	0.000544
ENSBTAT00000029020	ENSBTAG00000021771	PTTG1IP	1	145057828	145075459	-	CODING	0.743459	0.000548
ENSBTAT00000007922	ENSBTAG00000006029	OGDH	4	77458516	77512436	-	CODING	-0.36096	0.000551
ENSBTAT00000025469	ENSBTAG00000019133	ZNF326	3	53385189	53419460	-	CODING	0.830827	0.000556
ENSBTAT00000016254	ENSBTAG00000012253	EIF2C1	3	110466755	110492440	-	CODING	-1.37078	0.000564
ENSBTAT00000032276	ENSBTAG00000023607	PTPLB	1	68464487	68550787	-	CODING	1.006173	0.000578
ENSBTAT00000007444	ENSBTAG00000005666	LRRC20	28	26665369	26735793	-	CODING	-0.91703	0.00058
ENSBTAT00000012120	ENSBTAG00000009200	PAM16	25	3499875	3505894	-	CODING	0.569367	0.00058
ENSBTAT00000027944	ENSBTAG00000020983	RRAS	18	56497094	56501119	-	CODING	0.623638	0.000581
ENSBTAT00000011077	ENSBTAG00000008419	PDE2A	15	52913333	52984107	-	CODING	1.095212	0.000584
ENSBTAT00000005575	ENSBTAG00000004256	ODC1	11	87178098	87182503	+	CODING	-0.49968	0.000587
ENSBTAT00000038295	ENSBTAG00000008032	-	4	115901833	115951987	+	CODING	-0.53231	0.000597
ENSBTAT00000003343	ENSBTAG00000002580	MYH14	18	56874763	56947774	+	CODING	-0.98136	0.000607
ENSBTAT00000044005	ENSBTAG00000031088	-	3	61240417	61241633	+	CODING	-0.44287	0.000609
ENSBTAT00000035930	ENSBTAG00000004347	GPR116	23	20052363	20128180	-	CODING	0.488648	0.00062
ENSBTAT00000027702	ENSBTAG00000020787	PIK3R4	1	153233567	153320510	+	CODING	-0.79979	0.000624
ENSBTAT00000004127	ENSBTAG00000003177	SLC25A33	16	44801770	44831904	-	CODING	1.210695	0.000626
ENSBTAT00000044960	ENSBTAG00000031709	PDCD10	1	100540220	100587970	+	CODING	0.809557	0.00063
ENSBTAT00000014490	ENSBTAG00000010910	MYEOV2	3	120123114	120127712	-	CODING	0.311084	0.000631

ENSBTAT00000000259	ENSBTAG00000000215	GNB1	16	52158465	52182743	+	CODING	1.130422	0.000633
ENSBTAT00000029340	ENSBTAG00000039995	CFH	16	6052925	6122550	+	CODING	0.796514	0.000639
ENSBTAT00000039403	ENSBTAG00000008520	NFIC	7	21676708	21748545	-	CODING	-0.74505	0.00064
ENSBTAT00000004810	ENSBTAG00000003692	UCP2	15	54196170	54202633	-	CODING	0.783784	0.000641
ENSBTAT00000019656	ENSBTAG00000014775	GUK1	7	2895658	2905568	-	CODING	0.846793	0.000642
ENSBTAT00000029348	ENSBTAG00000022004	FLNB	22	43674720	43815706	-	CODING	0.874548	0.000642
ENSBTAT00000052354	ENSBTAG00000008635	SULT1A1	25	26381342	26383753	-	CODING	0.827403	0.000644
ENSBTAT00000019613	ENSBTAG00000014737	VEGFC	27	6999636	7084866	-	CODING	1.261864	0.000648
ENSBTAT00000044261	ENSBTAG00000046337	TUBB6	24	43249500	43250564	+	CODING	0.743618	0.000654
ENSBTAT00000008473	ENSBTAG00000006463	DLST	10	86533853	86550983	+	CODING	-0.46317	0.000659
ENSBTAT00000015459	ENSBTAG00000011639	STK11	7	45282199	45289554	-	CODING	-0.71113	0.000666
ENSBTAT00000064839	ENSBTAG00000045822	-	15	74915626	74916613	+	CODING	1.334919	0.000672
ENSBTAT00000056230	ENSBTAG00000037661	DENND1B	16	78480806	78590234	-	CODING	-1.04998	0.000673
ENSBTAT00000029056	ENSBTAG00000021798	ERBB2	19	40722051	40745987	+	CODING	-0.87157	0.000675
ENSBTAT00000018774	ENSBTAG00000014127	PGHS-2	16	69263776	69271399	-	CODING	1.671563	0.000685
ENSBTAT00000017860	ENSBTAG00000013423	GLRX5	21	61986090	61996011	+	CODING	0.461901	0.00069
ENSBTAT00000014571	ENSBTAG00000010976	KANK1	8	44046426	44076904	-	CODING	-0.85852	0.000691
ENSBTAT00000063377	ENSBTAG00000046526	ISCA1	8	80824529	80836135	-	CODING	-0.33171	0.0007
ENSBTAT00000003022	ENSBTAG00000002341	ETS1	29	32358407	32430222	-	CODING	0.835079	0.000709
ENSBTAT00000002578	ENSBTAG00000001987	HSPC321	15	43509670	43585976	-	CODING	1.09898	0.00071
ENSBTAT00000013127	ENSBTAG00000009951	PGP	25	1746200	1747344	-	CODING	-0.69307	0.00071
ENSBTAT00000013468	ENSBTAG00000010208	ZER1	11	99255811	99284276	-	CODING	-0.90931	0.000723
ENSBTAT00000000973	ENSBTAG00000000731	WFIKKN2	19	36536565	36542420	-	CODING	-1.26667	0.000737
ENSBTAT00000016938	ENSBTAG00000012741	CCPG1	10	55001792	55028162	+	CODING	-0.43807	0.000742
ENSBTAT00000021435	ENSBTAG00000001586	OTUD7B	3	20656593	20712080	+	CODING	-0.9386	0.000747
ENSBTAT00000046614	ENSBTAG00000014874	FYTTD1	1	70954511	70984201	-	CODING	-0.45601	0.000749
ENSBTAT00000015199	ENSBTAG00000011435	NPEPPS	19	39460692	39564822	-	CODING	-0.55181	0.000752
ENSBTAT00000028765	ENSBTAG00000021587	SMPDL3A	9	28811578	28828968	-	CODING	0.790052	0.000753
ENSBTAT00000011652	ENSBTAG00000008849	SORT1	3	34150876	34214025	+	CODING	-0.80027	0.000758
ENSBTAT00000063686	ENSBTAG00000046017	POPDC3	9	45452891	45455659	+	CODING	-0.59574	0.000758
ENSBTAT00000018383	ENSBTAG00000013843	ACVRL1	5	28097837	28106733	-	CODING	1.040546	0.000759
ENSBTAT00000061043	ENSBTAG00000016990	USP24	3	91711045	91866000	+	CODING	-0.93633	0.00078
ENSBTAT00000005534	ENSBTAG00000004225	TSR1	19	23877562	23886659	-	CODING	-0.78554	0.00078
ENSBTAT00000011485	ENSBTAG00000008717	SERPINE2	2	112870323	112900274	-	CODING	1.008777	0.000787
ENSBTAT00000047557	ENSBTAG00000033429	FAM229B	9	38816218	38821062	-	CODING	0.876728	0.000787
ENSBTAT00000056239	ENSBTAG00000037899	DLK	21	67274800	67283089	+	CODING	1.844423	0.000792
ENSBTAT00000020113	ENSBTAG00000015116	RAD23A	7	13724192	13730226	-	CODING	-0.37377	0.000794
ENSBTAT00000017583	ENSBTAG00000013210	ADAMTS4	3	8322938	8331146	+	CODING	1.777378	0.000799
ENSBTAT00000000240	ENSBTAG00000000203	ATP6V1G1	8	105493587	105502576	+	CODING	0.31256	0.0008
ENSBTAT00000018066	ENSBTAG00000013586	CCR2	22	53613211	53614323	-	CODING	5.153104	0.000815
ENSBTAT00000060961	ENSBTAG00000008301	WNK2	8	85984692	86107090	+	CODING	-1.61771	0.000817
ENSBTAT00000003370	ENSBTAG00000002606	LGR4	15	58873843	58977292	-	CODING	-1.03818	0.000825
ENSBTAT00000011617	ENSBTAG00000001632	NBR1	19	43785335	43812243	+	CODING	-0.64168	0.000845
ENSBTAT00000054840	ENSBTAG00000038896	RPS26	5	57603811	57606599	-	CODING	-0.5375	0.000848
ENSBTAT00000043690	ENSBTAG00000017713	KTN1	10	68238010	68351879	+	CODING	-0.47121	0.000849
ENSBTAT00000008789	ENSBTAG00000006679	MITF	22	31735990	31769463	-	CODING	-0.64755	0.000851
ENSBTAT00000049076	ENSBTAG00000034645	PON3	4	12441126	12478659	-	CODING	-0.94688	0.000858
ENSBTAT00000017282	ENSBTAG00000012998	NAA30	10	70014347	70031590	+	CODING	-0.81834	0.000862
ENSBTAT00000007026	ENSBTAG00000005339	-	23	17255947	17269998	+	CODING	1.120414	0.000866
ENSBTAT00000002235	ENSBTAG00000001706	SLC9A2	11	7324936	7414636	+	CODING	-1.1943	0.000866
ENSBTAT00000045415	ENSBTAG00000004908	CHRNE	19	27118517	27123114	+	CODING	-1.02691	0.000867
ENSBTAT00000012704	ENSBTAG00000009641	MTHFD1	10	76858872	76919406	+	CODING	-0.65599	0.000867
ENSBTAT00000022858	ENSBTAG00000017200	RHOJ	10	75783882	75877395	+	CODING	0.759827	0.00087
ENSBTAT00000012701	ENSBTAG00000009637	SLC12A2	7	26973462	27064924	-	CODING	-1.10211	0.000877
ENSBTAT00000015200	ENSBTAG00000011437	-	1	146207250	146216697	-	CODING	0.746756	0.000884
ENSBTAT00000026511	ENSBTAG00000019900	LNPEP	7	98825233	98878164	+	CODING	-1.07327	0.000896
ENSBTAT00000003926	ENSBTAG00000038409	MBD3	7	45577377	45579729	-	CODING	-0.568	0.000904
ENSBTAT00000023595	ENSBTAG00000017745	IL6ST	20	23212633	23270316	+	CODING	-0.63946	0.000905
ENSBTAT00000002922	ENSBTAG00000002267	ANKRD40	19	36655574	36675085	+	CODING	-0.77073	0.000912
ENSBTAT00000021303	ENSBTAG00000016004	METAP1	6	26925349	26979063	-	CODING	-0.73207	0.000917
ENSBTAT00000043069	ENSBTAG00000019294	ABCC9	5	88677586	88831324	+	CODING	-0.68496	0.000919
ENSBTAT00000017292	ENSBTAG00000013007	C13H20ORF108	13	60109036	60119697	-	CODING	-0.32943	0.000924
ENSBTAT00000038642	ENSBTAG00000026995	PNN	21	49612519	49618556	+	CODING	0.500256	0.000935
ENSBTAT00000022098	ENSBTAG00000016612	NEK9	10	86727710	86762452	-	CODING	-0.77704	0.000954

ENSBTAT00000017744	ENSBTAG00000013341	GYG2	X	140120347	140138151	-	CODING	-1.06995	0.000956
ENSBTAT00000025313	ENSBTAG00000019018	-	11	107260653	107261718	+	CODING	1.100236	0.000958
ENSBTAT00000024656	ENSBTAG00000018522	OAZ1	7	22577243	22580777	-	CODING	0.172252	0.000967
ENSBTAT00000008749	ENSBTAG00000006661	RALA	4	81919125	81929351	-	CODING	0.677181	0.000978
ENSBTAT00000017080	ENSBTAG00000012852	DICER1	21	61513000	61555942	-	CODING	-0.97218	0.000987
ENSBTAT00000024617	ENSBTAG00000018497	SDPR	2	80858087	80871118	-	CODING	0.612231	0.000991
ENSBTAT00000017898	ENSBTAG00000013455	SPOPL	2	58798302	58846989	-	CODING	-1.15699	0.000992
ENSBTAT00000031279	ENSBTAG00000005077	CXCL12	28	45410676	45418793	+	CODING	0.702593	0.000994
ENSBTAT00000026407	ENSBTAG00000019822	TPPP3	18	35121243	35124820	-	CODING	0.407635	0.001005
ENSBTAT00000002684	ENSBTAG00000002076	STAU2	14	38927061	39070664	-	CODING	-0.56034	0.001013
ENSBTAT00000012106	ENSBTAG00000009187	DNAJC28	1	1308527	1310072	+	CODING	-0.75907	0.001016
ENSBTAT00000003764	ENSBTAG00000002896	KPNA6	1	130554555	130643416	+	CODING	-0.513	0.001041
ENSBTAT00000044234	ENSBTAG00000048296	TMEM27	X	135092215	135124289	+	CODING	1.182016	0.001042
ENSBTAT00000025935	ENSBTAG00000019470	NEK6	11	95314593	95400813	+	CODING	0.749027	0.001055
ENSBTAT00000026428	ENSBTAG00000019834	ARL15	20	24955390	25026808	+	CODING	1.181904	0.001065
ENSBTAT00000031316	ENSBTAG00000012667	CAMK2G	28	29878540	29930559	-	CODING	-1.07018	0.001073
ENSBTAT00000005577	ENSBTAG00000004259	HPCAL1	11	87191215	87307998	-	CODING	1.118584	0.001081
ENSBTAT00000023734	ENSBTAG00000017855	ITM2C	2	119476496	119490380	+	CODING	0.994134	0.001089
ENSBTAT00000006407	ENSBTAG00000004873	CCNL2	16	52388692	52396825	+	CODING	0.84031	0.00109
ENSBTAT00000028355	ENSBTAG00000021282	SGCE	4	11840470	11911742	-	CODING	1.216258	0.001091
ENSBTAT00000015010	ENSBTAG00000011298	EYA1	14	36898122	37268909	-	CODING	-1.52441	0.001115
ENSBTAT00000024413	ENSBTAG00000018348	-	4	49037768	49052388	+	CODING	-0.66586	0.001121
ENSBTAT00000015277	ENSBTAG00000011494	PYGL	10	43800152	43840994	-	CODING	1.127657	0.001135
ENSBTAT00000061332	ENSBTAG00000000024	DYM	24	49193606	49590504	-	CODING	-0.55673	0.001137
ENSBTAT00000023313	ENSBTAG00000017537	PTGIS	13	78319020	78368752	-	CODING	1.084485	0.001141
ENSBTAT00000011270	ENSBTAG00000008548	FAM98A	11	16157514	16173332	-	CODING	-0.48183	0.001143
ENSBTAT00000016884	ENSBTAG00000012703	GLO1	23	12483468	12509232	-	CODING	-0.26214	0.001155
ENSBTAT00000009600	ENSBTAG00000007296	-	1	82549642	82550013	+	CODING	2.049108	0.001155
ENSBTAT00000001662	ENSBTAG00000001257	AGTPBP1	8	80235083	80392851	-	CODING	-1.08897	0.001162
ENSBTAT00000033724	ENSBTAG00000007767	TBX15	3	24236815	24329679	+	CODING	-0.77399	0.001165
ENSBTAT00000006451	ENSBTAG00000004902	INTS8	14	71820446	71848531	-	CODING	-0.71807	0.001165
ENSBTAT00000015282	ENSBTAG00000006531	DIP2C	13	47041229	47177126	+	CODING	-0.95314	0.001179
ENSBTAT00000027612	ENSBTAG00000020720	MMTAG2	7	2910375	2912630	+	CODING	0.839067	0.001194
ENSBTAT00000063404	ENSBTAG00000010083	HADHB	11	73215600	73246103	-	CODING	-1.18602	0.001212
ENSBTAT00000008569	ENSBTAG00000006523	SOD2	9	97399159	97404522	+	CODING	-0.34617	0.001217
ENSBTAT00000007057	ENSBTAG00000005354	LRRFIP1	3	117796596	117869819	+	CODING	-0.56708	0.00122
ENSBTAT00000007399	ENSBTAG00000005628	CD52	2	127396373	127397972	-	CODING	1.670383	0.001222
ENSBTAT00000006020	ENSBTAG00000004586	-	X	65143672	65149272	+	CODING	0.631388	0.001225
ENSBTAT00000005377	ENSBTAG00000004115	MYLIP	23	40770315	40790973	-	CODING	0.750235	0.001229
ENSBTAT00000015944	ENSBTAG00000012012	CYB5A	24	4413368	4445873	+	CODING	0.521181	0.001232
ENSBTAT00000036296	ENSBTAG00000019788	TEAD4	5	107264250	107325273	-	CODING	-0.95239	0.001233
ENSBTAT00000032674	ENSBTAG00000021306	CHRDL2	15	54785596	54819153	-	CODING	2.142308	0.001239
ENSBTAT00000039197	ENSBTAG00000006775	SLC12A7	20	71395142	71420351	+	CODING	1.50016	0.001245
ENSBTAT00000006908	ENSBTAG00000005249	RNF14	7	54717280	54735688	+	CODING	-0.55603	0.001247
ENSBTAT00000023618	ENSBTAG00000017763	NFIL3	8	87891326	87893182	+	CODING	0.643441	0.001252
ENSBTAT00000001731	ENSBTAG00000001311	MORF4L2	X	57744030	57756166	-	CODING	0.242921	0.001253
ENSBTAT00000023810	ENSBTAG00000031752	TMEM256	19	27714471	27715619	-	CODING	0.688518	0.001261
ENSBTAT00000020845	ENSBTAG00000015698	FYCO1	22	53951029	54026241	+	CODING	-0.80271	0.001265
ENSBTAT00000000791	ENSBTAG00000000599	CCNI	6	93633306	93672214	-	CODING	-0.36053	0.001267
ENSBTAT00000007063	ENSBTAG00000040215	EIF4G3	2	132097562	132323961	+	CODING	-2.15187	0.001283
ENSBTAT00000013707	ENSBTAG00000010386	ARPC2	2	106978526	107006143	+	CODING	0.363686	0.001283
ENSBTAT00000009545	ENSBTAG00000007256	DYRK2	5	46323523	46333943	-	CODING	-0.97053	0.001287
ENSBTAT00000002122	ENSBTAG00000001618	ALPK3	21	22883305	22935573	+	CODING	-0.37383	0.001292
ENSBTAT00000013855	ENSBTAG00000010487	MGC128424	7	64133812	64153713	+	CODING	1.437963	0.001298
ENSBTAT00000025416	ENSBTAG00000019091	RNPC3	3	39998006	40025400	-	CODING	0.779201	0.001313
ENSBTAT00000001191	ENSBTAG00000000898	F2RL2	10	7777600	7788321	-	CODING	1.49855	0.00132
ENSBTAT00000018457	ENSBTAG00000013899	LARP4B	13	46779271	46834689	+	CODING	-0.7136	0.001322
ENSBTAT00000033154	ENSBTAG00000013290	DYSF	11	12894979	13123270	-	CODING	-0.69859	0.001323
ENSBTAT00000007574	ENSBTAG00000005760	TBC1D4	12	50679632	50888598	-	CODING	-0.81721	0.001325
ENSBTAT00000008744	ENSBTAG00000006656	RANBP1	17	75036894	75038127	-	CODING	0.890344	0.001326
ENSBTAT00000023241	ENSBTAG00000017482	ISYNA1	7	4669453	4672648	+	CODING	1.120355	0.001328
ENSBTAT00000021896	ENSBTAG00000037673	GSTM4	3	33874019	33880598	-	CODING	-0.83611	0.001334
ENSBTAT00000011215	ENSBTAG00000023416	PPP2R3A	1	134223431	134394973	-	CODING	-0.71271	0.001342
ENSBTAT00000002938	ENSBTAG00000002279	LUC7L3	19	36622184	36647084	-	CODING	0.435713	0.00135

ENSBTAT00000031376	ENSBTAG00000023073	FAM89A	28	3656713	3676337	+	CODING	1.520534	0.001352
ENSBTAT00000018509	ENSBTAG00000013931	FAM96B	18	34754940	34756799	-	CODING	0.398648	0.001352
ENSBTAT00000018429	ENSBTAG00000013882	CHMP2A	18	65960799	65963496	-	CODING	0.557792	0.001358
ENSBTAT00000006646	ENSBTAG00000005039	ARAF	X	91180863	91191308	+	CODING	-0.44908	0.001376
ENSBTAT00000056240	ENSBTAG00000038849	TRDN	9	28071827	28219689	+	CODING	-0.18828	0.001401
ENSBTAT00000005016	ENSBTAG00000003845	CSRNP1	22	12520393	12532806	-	CODING	0.908595	0.001403
ENSBTAT00000048357	ENSBTAG00000014262	BZW2	4	25097884	25161432	+	CODING	-0.28871	0.001406
ENSBTAT00000011196	ENSBTAG00000008493	AQP3	8	76525369	76531334	-	CODING	1.202006	0.001419
ENSBTAT00000054581	ENSBTAG00000038464	PLIN5	7	20816181	20827916	+	CODING	0.875632	0.001423
ENSBTAT00000036270	ENSBTAG00000016874	DNAJB1	7	12366856	12370535	+	CODING	0.517615	0.001425
ENSBTAT00000015325	ENSBTAG00000011532	MLLT6	19	39938325	39956916	+	CODING	-0.79997	0.001433
ENSBTAT00000018795	ENSBTAG00000014140	ATP1B3	1	128050718	128085713	-	CODING	0.993845	0.001435
ENSBTAT00000019553	ENSBTAG00000014691	ARFGEF1	14	33343880	33480413	-	CODING	-0.65961	0.001436
ENSBTAT00000029284	ENSBTAG00000021965	SUB1	20	41122023	41143914	-	CODING	-0.41262	0.001447
ENSBTAT00000030639	ENSBTAG00000014474	API5	15	74303493	74321171	+	CODING	-0.69968	0.001471
ENSBTAT00000033229	ENSBTAG00000034586	-	11	1821486	1846701	+	CODING	1.104674	0.001472
ENSBTAT00000033238	ENSBTAG00000024097	-	3	110014938	110020632	+	CODING	0.555974	0.001474
ENSBTAT00000022056	ENSBTAG00000016580	TEF	5	113028012	113037944	+	CODING	-0.93468	0.001478
ENSBTAT00000029257	ENSBTAG00000021945	NID2	10	44894657	44986659	-	CODING	0.905557	0.001479
ENSBTAT00000040095	ENSBTAG00000008665	DIXDC1	15	22614860	22659906	+	CODING	-1.19188	0.00148
ENSBTAT00000023705	ENSBTAG00000017830	RBMS2	5	57167137	57219971	-	CODING	0.980401	0.001485
ENSBTAT00000034879	ENSBTAG00000025005	METTL7A	5	29161683	29172757	-	CODING	-0.62307	0.001486
ENSBTAT00000002653	ENSBTAG00000002048	PTPN11	17	64010592	64056567	-	CODING	-0.60754	0.001488
ENSBTAT00000002784	ENSBTAG00000002147	PPP4R2	22	28775667	28823253	-	CODING	-0.68204	0.001494
ENSBTAT00000031029	ENSBTAG00000011250	AFG3L2	24	43252927	43271906	-	CODING	-0.51475	0.001522
ENSBTAT00000026836	ENSBTAG00000020148	TEK	8	17040335	17143857	-	CODING	0.835708	0.001523
ENSBTAT00000021020	ENSBTAG00000015824	RNF141	15	42719433	42753584	+	CODING	-0.90593	0.001526
ENSBTAT00000013690	ENSBTAG00000010365	SQRDL	10	65039298	65093133	-	CODING	0.985472	0.001529
ENSBTAT00000008511	ENSBTAG00000014540	PERM1	16	52742637	52748240	+	CODING	-0.3777	0.001534
ENSBTAT00000053074	ENSBTAG00000047537	CCAR1	28	25228951	25249032	+	CODING	0.592379	0.001534
ENSBTAT00000064571	ENSBTAG00000046684	FOXN3	10	102020147	102300271	-	CODING	-0.69467	0.001537
ENSBTAT00000026841	ENSBTAG00000020152	PFDN2	3	8395605	8407551	+	CODING	0.457493	0.001552
ENSBTAT00000061376	ENSBTAG00000003667	TLN2	10	47303876	47796327	-	CODING	-1.11591	0.001562
ENSBTAT00000012515	ENSBTAG00000009511	-	15	13987258	13987770	+	CODING	-0.79947	0.001567
ENSBTAT00000001019	ENSBTAG00000000770	PGM2L1	15	54484806	54537330	-	CODING	-1.01304	0.001571
ENSBTAT00000049576	ENSBTAG00000012242	MAF1	14	1921784	1924818	-	CODING	-0.58504	0.001583
ENSBTAT00000048248	ENSBTAG00000006370	CCT3	3	14538814	14553305	+	CODING	0.836667	0.001586
ENSBTAT00000016771	ENSBTAG00000012637	C11H2orf40	11	45690020	45699417	-	CODING	2.216779	0.001589
ENSBTAT00000047142	ENSBTAG00000033186	OXCT1	20	32683996	32848723	+	CODING	-0.4034	0.00159
ENSBTAT00000011800	ENSBTAG00000008964	DTD1	13	38909550	38989758	+	CODING	1.037422	0.001601
ENSBTAT00000023325	ENSBTAG00000017547	DBNL	22	357512	370121	+	CODING	0.98631	0.001604
ENSBTAT00000014871	ENSBTAG00000011196	C1QB	2	130769172	130775911	-	CODING	1.257565	0.001608
ENSBTAT00000006569	ENSBTAG00000004990	NEO1	10	19795806	19959384	+	CODING	-1.04054	0.001609
ENSBTAT00000028723	ENSBTAG00000021558	-	13	55509535	55517058	-	CODING	-0.59965	0.001622
ENSBTAT00000036194	ENSBTAG00000025644	CALM	10	103059964	103067071	+	CODING	0.119295	0.001625
ENSBTAT00000004760	ENSBTAG00000003652	TMEM128	6	107477962	107493240	+	CODING	0.766783	0.001627
ENSBTAT00000021827	ENSBTAG00000016411	RNF122	27	28450185	28459083	-	CODING	-0.72487	0.001636
ENSBTAT00000005267	ENSBTAG00000004034	SESN3	15	15502203	15523079	+	CODING	-1.0511	0.001645
ENSBTAT00000024373	ENSBTAG00000018317	PPP2R2A	8	74834628	74913332	+	CODING	-0.43415	0.001647
ENSBTAT00000026826	ENSBTAG00000020139	RPL7	14	38741347	38744856	-	CODING	0.342433	0.001661
ENSBTAT00000018331	ENSBTAG00000013793	NEDD4	10	54536537	54624769	+	CODING	-1.1903	0.001675
ENSBTAT00000030328	ENSBTAG00000022396	SAA3	29	26668047	26671801	-	CODING	1.518356	0.001675
ENSBTAT00000017239	ENSBTAG00000012966	SCRN3	2	22400874	22432815	-	CODING	-1.08665	0.001681
ENSBTAT00000003297	ENSBTAG00000002539	TRIOBP	5	110069320	110092016	+	CODING	1.005828	0.001682
ENSBTAT00000025510	ENSBTAG00000019167	INPPL1	15	52609012	52623088	+	CODING	-0.62861	0.001694
ENSBTAT00000016040	ENSBTAG00000012088	FBLN1	5	116616200	116695692	+	CODING	0.714999	0.001698
ENSBTAT00000018468	ENSBTAG00000025263	FAM160A1	17	6089371	6207768	-	CODING	-0.94035	0.001705
ENSBTAT00000002654	ENSBTAG00000002049	HADH	6	18459380	18502809	-	CODING	-0.36478	0.001708
ENSBTAT00000005522	ENSBTAG00000004211	TNFRSF1A	5	104402771	104415588	+	CODING	0.816156	0.00173
ENSBTAT00000009405	ENSBTAG00000007147	CUEDC1	19	8900990	8974286	-	CODING	-0.54859	0.001742
ENSBTAT00000044085	ENSBTAG00000012103	MAP2K4	19	31284737	31363093	+	CODING	-0.75425	0.001759
ENSBTAT00000061118	ENSBTAG00000015376	FBXW11	20	3584765	3624526	-	CODING	-0.64583	0.001773
ENSBTAT00000019499	ENSBTAG00000014643	EEF1D	14	2317971	2326718	+	CODING	0.367445	0.001776
ENSBTAT00000027347	ENSBTAG00000020528	PCOLCE	25	36490771	36495789	-	CODING	0.789757	0.001778

ENSBTAT00000055155	ENSBTAG00000040380	-	15	51667478	51669427	+	CODING	-0.69672	0.001801
ENSBTAT00000065657	ENSBTAG00000019781	CLASP1	2	73490617	73723487	-	CODING	-0.99254	0.001809
ENSBTAT00000061255	ENSBTAG00000043969	CALN1	25	28894401	29349030	+	CODING	-1.95767	0.001814
ENSBTAT00000000843	ENSBTAG00000000641	SKAP1	19	38697518	39001101	+	CODING	-1.1956	0.001822
ENSBTAT00000003318	ENSBTAG00000002562	PRAF2	X	92231884	92234517	-	CODING	0.719377	0.001825
ENSBTAT00000012341	ENSBTAG00000047926	OVCA2	19	23644915	23646308	+	CODING	0.772445	0.001835
ENSBTAT00000006538	ENSBTAG00000004966	KIF1B	16	44080103	44212899	-	CODING	-0.7297	0.00185
ENSBTAT00000052944	ENSBTAG00000038527	-	4	18950187	18950489	-	CODING	0.580686	0.001855
ENSBTAT00000019865	ENSBTAG00000014921	IL6	4	31578311	31582667	+	CODING	3.530302	0.001877
ENSBTAT00000004597	ENSBTAG00000003535	DPM3	3	15517158	15517733	+	CODING	0.82889	0.00191
ENSBTAT00000025394	ENSBTAG00000019075	MAP1D	2	24512078	24529917	-	CODING	-2.45846	0.001911
ENSBTAT00000031530	ENSBTAG00000023179	TRIB1	14	16353388	16361545	-	CODING	0.509488	0.00193
ENSBTAT00000028361	ENSBTAG00000021287	SLC16A7	5	53987909	54214799	-	CODING	1.889872	0.001932
ENSBTAT00000006102	ENSBTAG00000031861	RHEB	4	114803157	114855282	-	CODING	0.436201	0.001936
ENSBTAT00000008102	ENSBTAG00000006161	C-MET	4	51912652	52042198	-	CODING	-1.00219	0.001945
ENSBTAT00000007505	ENSBTAG00000005715	FUOM	26	25872737	25875735	+	CODING	1.046235	0.001957
ENSBTAT00000064153	ENSBTAG00000047673	THUMPD1	25	18566260	18570516	-	CODING	-0.75309	0.001975
ENSBTAT00000014236	ENSBTAG00000010745	THRA	19	41017271	41042023	+	CODING	-0.57371	0.001989
ENSBTAT00000025683	ENSBTAG00000019290	PACSIN2	5	114357560	114475163	-	CODING	0.725866	0.00199
ENSBTAT00000046344	ENSBTAG00000012393	AGT	28	46147260	46156093	+	CODING	1.364453	0.001991
ENSBTAT00000010044	ENSBTAG00000007634	HOOK3	27	37278344	37358417	+	CODING	-0.81625	0.002027
ENSBTAT00000039536	ENSBTAG00000027513	-	6	90695494	90697557	+	CODING	4.616923	0.002037
ENSBTAT00000015224	ENSBTAG00000011456	NT5C3L	19	42657994	42665703	-	CODING	1.097699	0.002064
ENSBTAT00000039955	ENSBTAG00000027722	IPO7	15	43781463	43825036	-	CODING	-0.38574	0.002072
ENSBTAT00000015750	ENSBTAG00000011866	PCBD1	28	27226795	27231724	-	CODING	0.963118	0.002075
ENSBTAT00000009081	ENSBTAG00000006919	SMAD4	24	50993949	51045661	+	CODING	-0.49426	0.0021
ENSBTAT00000008130	ENSBTAG00000006187	MFAP4	19	34685357	34687892	+	CODING	1.008696	0.002104
ENSBTAT00000027593	ENSBTAG00000020704	RAMP3	4	77171037	77193849	-	CODING	1.178419	0.002114
ENSBTAT00000012256	ENSBTAG00000009304	PDCD5	18	43254168	43260903	+	CODING	0.417268	0.002119
ENSBTAT00000002893	ENSBTAG00000002236	TRPC4AP	13	64913710	64981620	-	CODING	-0.53952	0.002119
ENSBTAT00000054801	ENSBTAG00000039190	SLC9A5	18	34995067	35013345	+	CODING	-0.89952	0.002126
ENSBTAT00000033431	ENSBTAG00000000074	NFIA	3	84749656	85167847	-	CODING	-0.88469	0.002128
ENSBTAT00000064259	ENSBTAG00000047426	LYPD6	2	46723405	46759004	-	CODING	-2.27325	0.002129
ENSBTAT00000012886	ENSBTAG00000009770	GPD2	2	39765206	39912916	-	CODING	-0.91657	0.002129
ENSBTAT00000000100	ENSBTAG00000000091	NDUFB8	26	21283406	21288806	-	CODING	0.264024	0.00213
ENSBTAT00000012526	ENSBTAG00000009519	TRIM23	20	13891748	13925788	+	CODING	-1.0131	0.00213
ENSBTAT00000012511	ENSBTAG00000009508	SMDT1	5	113570339	113574038	+	CODING	0.329878	0.002132
ENSBTAT00000031588	ENSBTAG00000017108	OTUD3	2	133387211	133416030	-	CODING	-0.78061	0.002143
ENSBTAT00000020351	ENSBTAG00000015303	MPP6	4	71625584	71690219	-	CODING	-1.12147	0.002162
ENSBTAT00000020080	ENSBTAG00000015089	LGALS1	5	110014543	110017878	+	CODING	0.192463	0.002167
ENSBTAT00000021620	ENSBTAG00000016252	LSM12	19	44509434	44529146	-	CODING	-0.44784	0.002173
ENSBTAT00000014390	ENSBTAG00000010843	PGRMC2	17	29872406	29890867	+	CODING	-0.29051	0.002186
ENSBTAT00000019182	ENSBTAG00000014422	MTMR10	21	27947773	27984436	-	CODING	-0.8324	0.002191
ENSBTAT00000006231	ENSBTAG00000004747	SPCS1	22	48710805	48712454	-	CODING	0.285624	0.002193
ENSBTAT00000019496	ENSBTAG00000014642	NAPRT	14	2327870	2331019	+	CODING	1.369471	0.002195
ENSBTAT00000064159	ENSBTAG00000047249	WBSCR1	25	33708591	33729652	-	CODING	-0.23342	0.002196
ENSBTAT00000002681	ENSBTAG00000002075	MME	1	113387231	113502393	-	CODING	1.18664	0.0022
ENSBTAT00000064362	ENSBTAG00000046319	NDUFA7	7	18191587	18198867	-	CODING	0.355244	0.0022
ENSBTAT00000019872	ENSBTAG00000014927	OXSR1	22	11674127	11758890	+	CODING	-0.96001	0.002218
ENSBTAT00000014035	ENSBTAG00000010617	KRTCAP2	3	15498847	15501486	+	CODING	0.435124	0.002242
ENSBTAT00000004113	ENSBTAG00000003165	ADAMTS9	22	36876910	37037695	+	CODING	1.58372	0.002249
ENSBTAT00000064899	ENSBTAG00000044125	-	3	48451880	48493897	-	CODING	-2.50159	0.002249
ENSBTAT00000022714	ENSBTAG00000017081	MIEF2	19	34984928	34986841	-	CODING	-1.06142	0.002252
ENSBTAT00000006383	ENSBTAG00000004855	PRDX6	16	56389804	56399714	+	CODING	-0.25026	0.002255
ENSBTAT00000003514	ENSBTAG00000038842	NEDD8	10	20777431	20779257	+	CODING	-0.42475	0.002259
ENSBTAT00000046260	ENSBTAG00000011488	PRPF8	19	23324762	23357323	-	CODING	-0.50872	0.002275
ENSBTAT00000053888	ENSBTAG00000039708	C9orf172	11	106338334	106341261	-	CODING	-1.16653	0.00228
ENSBTAT00000003743	ENSBTAG00000002880	SORBS1	26	16721039	16919269	-	CODING	-0.70537	0.002285
ENSBTAT00000028718	ENSBTAG00000021554	NOL7	23	42795776	42800011	-	CODING	0.436852	0.002285
ENSBTAT00000004358	ENSBTAG00000003359	ELOVL5	23	25155743	25228997	-	CODING	1.313676	0.002313
ENSBTAT00000012026	ENSBTAG00000009126	YBX2	19	27622773	27628780	-	CODING	1.660795	0.002334
ENSBTAT00000005230	ENSBTAG00000004005	WDR7	24	56467338	56817379	+	CODING	-1.01229	0.002356
ENSBTAT00000045869	ENSBTAG00000039731	RND3	2	45800466	45822011	+	CODING	0.998441	0.002357
ENSBTAT00000010491	ENSBTAG00000007979	TNPO1	20	8650349	8694889	-	CODING	-0.69215	0.002367

ENSBTAT00000061629	ENSBTAG00000040215	EIF4G3	2	132097562	132451412 +	CODING	-0.89389	0.002373
ENSBTAT00000002607	ENSBTAG00000002012	FAM96A	10	45936706	45952110 +	CODING	0.458937	0.002375
ENSBTAT00000037329	ENSBTAG00000026309	ZHX2	14	18386657	18389173 -	CODING	-0.81236	0.002384
ENSBTAT00000011172	ENSBTAG00000022204	-	X	57664213	57664899 +	CODING	0.513675	0.002386
ENSBTAT00000007817	ENSBTAG00000005957	CSE1L	13	77919378	77954416 +	CODING	-1.06859	0.002392
ENSBTAT00000037984	ENSBTAG00000013309	SFRS4	2	124956594	124982238 +	CODING	0.564176	0.002397
ENSBTAT00000007108	ENSBTAG00000005397	CD46	16	77483017	77522635 -	CODING	0.460146	0.002403
ENSBTAT00000039933	ENSBTAG00000027713	LYSMD3	7	92440985	92446470 -	CODING	-1.13292	0.002413
ENSBTAT00000065097	ENSBTAG00000045497	-	21	66764061	66764393 +	CODING	0.378544	0.002436
ENSBTAT00000006875	ENSBTAG00000005221	WNK1	5	108079510	108207083 +	CODING	-0.66184	0.002458
ENSBTAT00000004182	ENSBTAG00000003221	ATF7IP	5	95800988	95858963 -	CODING	-0.77586	0.002459
ENSBTAT00000035681	ENSBTAG00000007494	SMARCA2	8	42652775	42830159 -	CODING	-0.63113	0.002464
ENSBTAT00000005698	ENSBTAG00000004356	ROBO4	29	28719989	28734705 -	CODING	0.826129	0.002464
ENSBTAT00000027808	ENSBTAG00000020873	MAT2A	11	49298517	49305175 -	CODING	0.394938	0.00248
ENSBTAT00000000994	ENSBTAG00000000746	PCAF	1	44857815	44928998 +	CODING	-0.52237	0.002483
ENSBTAT00000006358	ENSBTAG00000004840	C1S	5	103768066	103779250 -	CODING	0.786598	0.002487
ENSBTAT00000024663	ENSBTAG00000018530	TUBA8	5	109876442	109894993 +	CODING	-0.24526	0.002503
ENSBTAT00000004611	ENSBTAG00000003545	TAF1D	29	1056627	1062580 +	CODING	0.931484	0.00252
ENSBTAT00000025873	ENSBTAG00000019422	-	11	22366049	22367102 -	CODING	-0.88028	0.002534
ENSBTAT00000044859	ENSBTAG00000031641	PSMD8	18	48451832	48457821 +	CODING	0.39805	0.002535
ENSBTAT00000007398	ENSBTAG00000005627	PAFAH1B2	15	28260081	28286877 +	CODING	-0.53035	0.002539
ENSBTAT00000054270	ENSBTAG00000045748	-	22	57156996	57161484 -	CODING	-0.3301	0.002552
ENSBTAT00000024782	ENSBTAG00000037377	ABHD14B	22	49516824	49520467 +	CODING	0.88865	0.00267
ENSBTAT00000026299	ENSBTAG00000019733	ELTD1	3	65921137	66062039 +	CODING	1.019375	0.002682
ENSBTAT00000021155	ENSBTAG00000015909	PDE8A	21	23119315	23262118 +	CODING	-0.52524	0.002694
ENSBTAT00000010914	ENSBTAG00000008294	KCNJ2	19	61185603	61195897 -	CODING	-1.1272	0.002695
ENSBTAT00000012940	ENSBTAG00000009813	ELOVL1	3	103233547	103238303 +	CODING	0.798149	0.002695
ENSBTAT00000003653	ENSBTAG00000002823	MPZL1	3	930876	1012734 -	CODING	0.733529	0.002709
ENSBTAT00000006666	ENSBTAG00000005057	FAM204A	26	38923063	38945878 -	CODING	0.439961	0.002711
ENSBTAT00000036848	ENSBTAG00000007068	SH3BGR	1	141029893	141100984 +	CODING	-0.13814	0.002721
ENSBTAT00000055739	ENSBTAG00000011689	LENG8	18	63107489	63117295 +	CODING	0.652362	0.002724
ENSBTAT00000028669	ENSBTAG00000021512	ZBTB7B	3	15634915	15648928 -	CODING	-0.67471	0.002738
ENSBTAT00000064534	ENSBTAG00000046733	-	9	102132404	102133397 -	CODING	1.012443	0.002747
ENSBTAT00000006979	ENSBTAG00000005299	EDNRB	12	53310604	53407024 -	CODING	1.181359	0.002761
ENSBTAT00000055329	ENSBTAG00000016806	PAFAH1B1	19	24088805	24158415 +	CODING	-0.31933	0.002766
ENSBTAT00000007391	ENSBTAG00000005622	LITAF	25	10288891	10325021 -	CODING	0.867113	0.002775
ENSBTAT00000066209	ENSBTAG00000045828	PTBP1	7	45016278	45025719 +	CODING	1.118342	0.00279
ENSBTAT00000039221	ENSBTAG00000034436	PDPK1	25	2062819	2097196 +	CODING	-0.95039	0.002806
ENSBTAT00000005481	ENSBTAG00000032517	BCL7A	17	55551316	55573152 -	CODING	-1.8693	0.002814
ENSBTAT00000053988	ENSBTAG00000037413	TMEM164	X	62882939	63056847 +	CODING	-0.90148	0.002836
ENSBTAT00000003955	ENSBTAG00000003039	PSMB8	23	7113304	7116676 -	CODING	0.825405	0.002857
ENSBTAT00000006715	ENSBTAG00000005096	COX7A2	9	15041836	15046972 -	CODING	0.817485	0.002863
ENSBTAT00000029282	ENSBTAG00000021963	SLAIN2	6	68695298	68741224 +	CODING	-0.50687	0.002866
ENSBTAT00000019626	ENSBTAG00000014751	ZIC1	1	121863151	121867967 -	CODING	-1.06401	0.002872
ENSBTAT00000045437	ENSBTAG00000020855	PTPN4	2	72049607	72146322 +	CODING	-0.95	0.002898
ENSBTAT00000000049	ENSBTAG00000000044	MYADML2	19	51562314	51565450 +	CODING	-0.63658	0.002901
ENSBTAT00000019429	ENSBTAG00000014596	EFHD1	3	113006125	113050192 +	CODING	1.033765	0.002907
ENSBTAT00000049233	ENSBTAG00000034785	DNAJC15	12	13183734	13266310 +	CODING	0.970777	0.002926
ENSBTAT00000001760	ENSBTAG00000001333	PPARG	22	57366997	57489570 -	CODING	1.702734	0.002932
ENSBTAT00000053650	ENSBTAG00000038156	DYNLT3	X	111023274	111034498 +	CODING	0.677044	0.002947
ENSBTAT00000024307	ENSBTAG00000018261	PDHX	15	66231837	66307403 +	CODING	-0.34344	0.002955
ENSBTAT00000035541	ENSBTAG00000025329	IRF2BPL	10	89245839	89248163 -	CODING	-1.01089	0.00296
ENSBTAT00000020662	ENSBTAG00000015551	-	4	100280752	100281437 -	CODING	0.989923	0.002963
ENSBTAT00000003340	ENSBTAG00000002579	FBL	18	49659188	49718941 -	CODING	0.580097	0.002965
ENSBTAT00000023383	ENSBTAG00000017584	PLCG1	13	70510824	70524389 +	CODING	0.951135	0.002967
ENSBTAT00000063308	ENSBTAG00000047278	NDUFB11	X	90791402	90793658 -	CODING	0.286403	0.002993
ENSBTAT00000002790	ENSBTAG00000002157	LMOD2	4	88746219	88755270 +	CODING	0.164831	0.003009
ENSBTAT00000022559	ENSBTAG00000016959	LAPTM4B	14	68668786	68730708 -	CODING	0.476121	0.003013
ENSBTAT00000024333	ENSBTAG00000018285	VAMP5	11	49249763	49258304 -	CODING	0.781383	0.00303
ENSBTAT00000025561	ENSBTAG00000019203	S100A4	3	16887102	16888570 +	CODING	0.52692	0.003033
ENSBTAT00000009782	ENSBTAG00000007438	STT3B	22	6083636	6184904 +	CODING	-0.51863	0.003046
ENSBTAT00000023727	ENSBTAG00000008520	NFIC	7	21676708	21748618 -	CODING	-0.50704	0.003062
ENSBTAT00000015882	ENSBTAG00000011970	FNIP2	17	41065498	41144357 -	CODING	1.499212	0.003064
ENSBTAT00000021243	ENSBTAG00000015969	STK40	3	110074348	110109946 +	CODING	-0.63861	0.003073

ENSBTAT00000009325	ENSBTAG00000007089	FRG1	27	18244331	18258187	+	CODING	0.467316	0.003076
ENSBTAT00000003373	ENSBTAG00000002603	PRPF39	21	55289715	55324776	+	CODING	0.713578	0.003077
ENSBTAT00000021501	ENSBTAG00000016152	DAB2	20	35018908	35079162	+	CODING	1.000329	0.003089
ENSBTAT00000029455	ENSBTAG00000006084	PINK1	2	132601302	132621738	-	CODING	-0.22294	0.003116
ENSBTAT00000061260	ENSBTAG00000025308	STON2	10	93690513	93817391	-	CODING	-1.70409	0.003136
ENSBTAT00000026800	ENSBTAG00000020121	RWDD2B	1	6521212	6532969	+	CODING	-0.97846	0.00314
ENSBTAT00000013864	ENSBTAG00000010490	PEX5	5	103530565	103547919	-	CODING	-2.43142	0.003143
ENSBTAT00000033845	ENSBTAG00000001942	SNX27	3	19128462	19204626	-	CODING	-0.75901	0.003144
ENSBTAT00000014052	ENSBTAG00000010630	MGC165715	17	30106834	30143868	-	CODING	0.667457	0.00315
ENSBTAT00000027794	ENSBTAG00000020861	CHAMP1	12	91100867	91103281	+	CODING	-0.78977	0.003152
ENSBTAT00000008096	ENSBTAG00000006155	CCRL2	22	53567797	53569696	-	CODING	1.229613	0.003154
ENSBTAT00000027974	ENSBTAG00000021008	ZNF219	10	26053957	26056906	+	CODING	-1.02937	0.003164
ENSBTAT00000018843	ENSBTAG00000014176	REM1	13	61619569	61628687	+	CODING	1.054033	0.003166
ENSBTAT00000008751	ENSBTAG00000006663	CYTH1	19	54240689	54261902	+	CODING	0.810241	0.003183
ENSBTAT00000064569	ENSBTAG00000046750	B3GNT3	7	5271365	5291295	-	CODING	0.901969	0.00319
ENSBTAT00000003252	ENSBTAG00000002504	IREB2	21	31352398	31401775	+	CODING	-0.69592	0.003206
ENSBTAT00000039626	ENSBTAG00000001348	COMMD8	6	67746952	67761215	-	CODING	-0.81187	0.003209
ENSBTAT00000055646	ENSBTAG00000022825	BRI3	25	38281392	38287463	-	CODING	0.365129	0.003219
ENSBTAT00000055086	ENSBTAG00000017389	RPLP0	17	64809254	64813289	-	CODING	0.143795	0.00324
ENSBTAT00000013159	ENSBTAG00000009979	HOXB2	19	38583393	38585653	+	CODING	1.436191	0.003262
ENSBTAT00000007150	ENSBTAG00000005432	CAPG	11	49423731	49438680	+	CODING	1.516424	0.003266
ENSBTAT00000036144	ENSBTAG00000011104	RTN4	11	37575270	37649696	-	CODING	1.242793	0.003272
ENSBTAT00000024678	ENSBTAG00000018542	COX5B	11	2916017	2918064	-	CODING	0.120729	0.003274
ENSBTAT00000025099	ENSBTAG00000018852	APC	10	1116669	1212429	-	CODING	-0.74183	0.003284
ENSBTAT00000030962	ENSBTAG00000022808	CACUL1	26	39292197	39356979	-	CODING	-0.89974	0.003301
ENSBTAT00000007711	ENSBTAG00000005868	HEBP1	5	97348105	97385488	+	CODING	1.047462	0.003306
ENSBTAT00000012210	ENSBTAG00000009267	UHRF1BP1	23	8777063	8824585	+	CODING	-1.10572	0.003306
ENSBTAT00000010911	ENSBTAG00000008291	PROCR	13	65052810	65106553	+	CODING	0.956634	0.00331
ENSBTAT00000026372	ENSBTAG00000019794	SYPL1	4	47423619	47453580	-	CODING	-0.25688	0.003312
ENSBTAT00000010815	ENSBTAG00000008224	PAIP2	7	52252774	52270426	+	CODING	0.201097	0.003329
ENSBTAT00000025696	ENSBTAG00000019298	STRADB	2	90432216	90456178	+	CODING	-0.34545	0.003339
ENSBTAT00000005015	ENSBTAG00000003846	RPL37A	2	105202277	105204896	+	CODING	0.148661	0.003355
ENSBTAT00000011070	ENSBTAG00000008412	BCL7C	25	27215012	27218439	-	CODING	1.055041	0.003388
ENSBTAT00000019792	ENSBTAG00000014863	GPC	2	79255849	79304396	+	CODING	0.269153	0.003412
ENSBTAT00000028260	ENSBTAG00000021208	RRM2B	14	64315460	64352488	+	CODING	-1.56011	0.003433
ENSBTAT00000016277	ENSBTAG00000012272	TMEM19	5	1303245	1321239	+	CODING	-0.89887	0.003453
ENSBTAT00000008847	ENSBTAG00000006729	ARID5B	28	18003736	18191994	+	CODING	0.659072	0.003473
ENSBTAT00000024019	ENSBTAG00000018045	SLC12A4	18	35547822	35569646	-	CODING	0.719287	0.003478
ENSBTAT00000054334	ENSBTAG00000013300	KCNMA1	28	32827041	33587986	-	CODING	-4.42918	0.003506
ENSBTAT00000020344	ENSBTAG00000015318	PVRL2	18	52984272	53007832	+	CODING	0.674508	0.003536
ENSBTAT00000014735	ENSBTAG00000011100	CTSC	29	7433482	7473315	+	CODING	0.770672	0.003558
ENSBTAT00000019913	ENSBTAG00000014956	JSRP1	7	22700027	22703913	-	CODING	-0.20668	0.003585
ENSBTAT00000004145	ENSBTAG00000003191	FSCN1	25	39292721	39302192	-	CODING	1.058445	0.003621
ENSBTAT00000065463	ENSBTAG00000014448	-	25	28153082	28157501	+	CODING	-7.7429	0.003633
ENSBTAT00000002691	ENSBTAG00000002082	MARCKS	9	37022764	37026932	-	CODING	0.613369	0.003727
ENSBTAT00000002400	ENSBTAG00000001836	-	19	19937602	19947884	-	CODING	-0.77832	0.003734
ENSBTAT00000010949	ENSBTAG00000008314	SDHB	2	136125304	136154362	+	CODING	0.14102	0.00374
ENSBTAT00000010479	ENSBTAG00000007969	CIDEC	22	16909073	16918353	+	CODING	0.933061	0.003742
ENSBTAT00000021860	ENSBTAG00000016441	ZNF622	20	56767512	56781229	+	CODING	0.694328	0.003764
ENSBTAT00000064656	ENSBTAG00000046493	PDCD6	20	71916825	71932650	-	CODING	0.756885	0.003783
ENSBTAT00000004604	ENSBTAG00000003543	KEAP1	7	16235815	16244046	-	CODING	-0.41164	0.003821
ENSBTAT00000001296	ENSBTAG00000000979	SMIM19	27	37055278	37070241	+	CODING	-0.68323	0.003825
ENSBTAT00000038384	ENSBTAG00000026848	-	3	11468021	11468512	-	CODING	0.17945	0.00383
ENSBTAT00000012308	ENSBTAG00000009351	ATXN10	5	116756235	116897163	+	CODING	-0.38019	0.003833
ENSBTAT00000034295	ENSBTAG00000015559	ERI3	3	102293112	102421170	+	CODING	1.309572	0.003855
ENSBTAT00000009785	ENSBTAG00000007441	SEC23IP	26	40346540	40380689	+	CODING	-0.7041	0.003859
ENSBTAT00000016349	ENSBTAG00000012321	ZFAND6	21	26543823	26591580	+	CODING	-0.25235	0.003864
ENSBTAT00000017857	ENSBTAG00000013420	FSD1L	8	96964125	97006477	+	CODING	-1.16392	0.003871
ENSBTAT00000026019	ENSBTAG00000019526	CMTM6	22	7001971	7024065	-	CODING	0.623455	0.003892
ENSBTAT00000014213	ENSBTAG00000010735	HIGD2A	7	39207845	39208828	+	CODING	-0.30088	0.003913
ENSBTAT00000026319	ENSBTAG00000019750	TMEM106B	4	19893089	19915667	+	CODING	-0.62895	0.003961
ENSBTAT00000036427	ENSBTAG00000017077	CTSL	8	84976257	84981349	-	CODING	1.418111	0.003967
ENSBTAT00000043916	ENSBTAG00000001700	CDC42	2	131341266	131359925	-	CODING	0.253845	0.003975
ENSBTAT00000022847	ENSBTAG00000017191	-	13	44229670	44230470	+	CODING	0.365976	0.004002

ENSBTAT00000061057	ENSBTAG00000007732	ARPP21	22	9674589	9791425	+	CODING	-0.75668	0.004022
ENSBTAT00000013608	ENSBTAG00000010303	ICAM1	7	16040938	16050247	+	CODING	1.564748	0.004025
ENSBTAT00000032643	ENSBTAG00000023792	-	9	42568764	42569201	-	CODING	0.407194	0.004025
ENSBTAT00000013703	ENSBTAG00000010383	YTHDF1	13	54805022	54817791	+	CODING	-0.42734	0.004072
ENSBTAT00000038024	ENSBTAG00000011582	SERINC2	2	122814786	122817735	+	CODING	-0.36197	0.0041
ENSBTAT00000012201	ENSBTAG00000009260	GPM6B	X	136872591	136915809	+	CODING	0.976647	0.004106
ENSBTAT00000006115	ENSBTAG00000004659	COQ10B	2	86410860	86427846	+	CODING	0.650517	0.004129
ENSBTAT00000038477	ENSBTAG00000015396	RBM24	23	39835982	39846198	-	CODING	-0.55153	0.004186
ENSBTAT00000007052	ENSBTAG00000005364	HMBS	15	30194980	30202275	+	CODING	-0.65023	0.004195
ENSBTAT00000015465	ENSBTAG00000011645	U2AF1	1	144832445	144845410	-	CODING	0.40358	0.004199
ENSBTAT00000022212	ENSBTAG00000016710	PTRH2	19	10894828	10903658	-	CODING	-0.40904	0.004202
ENSBTAT00000064202	ENSBTAG00000011121	CLCN4	X	142758548	142826322	-	CODING	-1.28957	0.004203
ENSBTAT00000025212	ENSBTAG00000018945	AMOTL1	15	15794576	15884804	-	CODING	-0.81938	0.004228
ENSBTAT00000025580	ENSBTAG00000019214	USP14	24	35529296	35564664	+	CODING	-0.48508	0.004239
ENSBTAT00000006594	ENSBTAG00000005009	PRELID1	7	40132356	40135909	+	CODING	0.834999	0.004261
ENSBTAT00000011658	ENSBTAG00000008853	HNRNPF	28	13902311	13920905	-	CODING	0.261762	0.004261
ENSBTAT00000018443	ENSBTAG00000013889	TMEM141	11	106375058	106376837	-	CODING	0.743781	0.004281
ENSBTAT00000026031	ENSBTAG00000019536	CRBN	22	23229272	23251050	+	CODING	-0.40867	0.004283
ENSBTAT00000003560	ENSBTAG00000002746	OSTF1	8	51440387	51501432	+	CODING	0.77058	0.004284
ENSBTAT00000024412	ENSBTAG00000018347	IL33	8	38724603	38792047	-	CODING	0.830304	0.00429
ENSBTAT00000028220	ENSBTAG00000021174	NRD1	3	95041110	95122769	+	CODING	-0.5188	0.004307
ENSBTAT00000002616	ENSBTAG00000002018	EIF5A	19	27648382	27653256	+	CODING	-0.1644	0.004343
ENSBTAT00000064762	ENSBTAG00000045604	TTC9	10	82634715	82666926	+	CODING	-0.31533	0.004359
ENSBTAT00000018759	ENSBTAG00000014112	EXOC4	4	97791626	98594890	+	CODING	-0.87445	0.004361
ENSBTAT00000005684	ENSBTAG00000004344	ACSL1	27	14223449	14288333	-	CODING	0.206585	0.004408
ENSBTAT00000038591	ENSBTAG00000026963	NCALD	14	64875135	64898581	+	CODING	0.879274	0.004409
ENSBTAT00000039415	ENSBTAG00000027446	RSU1	13	31347074	31554426	-	CODING	0.620272	0.004424
ENSBTAT00000023619	ENSBTAG00000017761	SMC1A	X	96218220	96252806	-	CODING	-0.85414	0.004427
ENSBTAT00000045329	ENSBTAG00000006561	RASGEF1B	6	97988424	98026691	-	CODING	1.305556	0.004443
ENSBTAT00000018986	ENSBTAG00000014289	ZFYVE9	3	94600486	94713143	-	CODING	-0.78844	0.004449
ENSBTAT00000015085	ENSBTAG00000011351	TMEM259	7	45141266	45144158	-	CODING	-0.39303	0.004453
ENSBTAT00000021209	ENSBTAG00000015950	ISOC2	18	62502067	62508467	+	CODING	0.875311	0.004456
ENSBTAT00000028638	ENSBTAG00000021485	C1orf54	3	20406684	20413546	-	CODING	1.136529	0.004482
ENSBTAT00000029211	ENSBTAG00000021910	TBL1XR1	1	90517079	90605473	+	CODING	-0.60935	0.004501
ENSBTAT00000014593	ENSBTAG00000010992	CTSH	21	25675908	25696632	-	CODING	0.832517	0.004535
ENSBTAT00000016190	ENSBTAG00000012192	-	5	74997804	75003221	-	CODING	1.311986	0.004552
ENSBTAT00000009684	ENSBTAG00000007363	LSM3	22	58693674	58701742	-	CODING	0.53296	0.004559
ENSBTAT00000043412	ENSBTAG00000019269	COL6A2	1	147542825	147570736	+	CODING	0.845004	0.004565
ENSBTAT00000048178	ENSBTAG00000017278	ACBD5	13	18047209	18075549	-	CODING	-0.682	0.004578
ENSBTAT00000018977	ENSBTAG00000014284	ALPK2	24	58119582	58248068	-	CODING	-0.94277	0.00458
ENSBTAT00000021671	ENSBTAG00000016290	MOB1B	6	87976520	88030195	+	CODING	-1.15924	0.004592
ENSBTAT00000031396	ENSBTAG00000016377	IPP	3	100935637	100972708	+	CODING	-1.09526	0.004592
ENSBTAT00000013623	ENSBTAG00000010312	MAPK1	17	74016493	74035118	+	CODING	-0.42589	0.004687
ENSBTAT00000013754	ENSBTAG00000010423	LIFR	20	35917479	35966671	+	CODING	-0.73823	0.004707
ENSBTAT00000025837	ENSBTAG00000019390	CA12	10	46749740	46768704	+	CODING	-3.52098	0.004712
ENSBTAT00000000233	ENSBTAG00000000199	PDP1	14	72678594	72686976	-	CODING	-6.88374	0.004723
ENSBTAT00000011332	ENSBTAG00000008593	INCA1	19	27047777	27052238	+	CODING	1.039949	0.004781
ENSBTAT00000064314	ENSBTAG00000011351	TMEM259	7	45141210	45144158	-	CODING	0.705709	0.00479
ENSBTAT00000043235	ENSBTAG00000010982	UBE2B	7	47528581	47541198	+	CODING	0.140842	0.004793
ENSBTAT00000012101	ENSBTAG00000009183	SHISA5	22	51944064	51969644	+	CODING	0.733201	0.004819
ENSBTAT00000011362	ENSBTAG00000008612	C1R	5	103737177	103747996	+	CODING	0.982102	0.004825
ENSBTAT00000065805	ENSBTAG00000047293	NRIP1	1	21693828	21697298	+	CODING	-0.83675	0.004836
ENSBTAT00000026816	ENSBTAG00000025280	-	23	50603365	50617511	+	CODING	0.36344	0.004867
ENSBTAT00000016923	ENSBTAG00000012729	ARHGEF9	X	101747194	102063219	+	CODING	1.263418	0.004875
ENSBTAT00000006554	ENSBTAG00000004979	MRS2	23	33038551	33077190	-	CODING	-0.65583	0.004898
ENSBTAT00000030378	ENSBTAG00000000460	SYTL2	29	9806835	9927317	+	CODING	1.36716	0.004905
ENSBTAT00000064232	ENSBTAG00000045692	-	18	34196399	34215940	-	CODING	0.638576	0.004917
ENSBTAT00000022227	ENSBTAG00000016720	RAB1A	11	63429725	63458558	-	CODING	0.390083	0.004918
ENSBTAT00000057380	ENSBTAG00000039023	ZNF664	17	53969695	53970480	-	CODING	-0.54843	0.004935
ENSBTAT00000011396	ENSBTAG00000008642	POLR1D	12	32523508	32556412	-	CODING	0.668621	0.004939
ENSBTAT00000010168	ENSBTAG00000007734	MEMO1	11	14564175	14627388	-	CODING	-0.31738	0.004943
ENSBTAT00000011559	ENSBTAG00000021039	-	7	18289395	18329947	+	CODING	0.371244	0.004954
ENSBTAT00000024034	ENSBTAG00000025848	RBM3	X	91768033	91770790	+	CODING	0.67934	0.004961
ENSBTAT00000034148	ENSBTAG00000021298	METTL11B	16	38568049	38586028	+	CODING	-3.0319	0.004963

ENSBTAT00000027670	ENSBTAG00000020764	CNN2	7	45159981	45162941	+	CODING	0.878642	0.00504
ENSBTAT00000002113	ENSBTAG00000001612	LSM1	27	33042449	33051041	-	CODING	0.585988	0.00507
ENSBTAT00000024699	ENSBTAG00000018557	YTHDF3	14	29830349	29863766	+	CODING	-0.48833	0.005073
ENSBTAT00000002279	ENSBTAG00000001745	LUM	5	21037443	21044658	-	CODING	0.506519	0.00509
ENSBTAT00000061230	ENSBTAG00000040206	ZNF770	10	30525210	30527276	-	CODING	-0.89119	0.005139
ENSBTAT00000032260	ENSBTAG00000002394	TMEM44	1	73516016	73559858	+	CODING	1.698649	0.005179
ENSBTAT00000015716	ENSBTAG00000011843	EEF1G	29	41614571	41624167	-	CODING	0.151914	0.005191
ENSBTAT00000051818	ENSBTAG00000034978	FAM168B	2	1793433	1834568	-	CODING	-0.61781	0.005196
ENSBTAT00000000433	ENSBTAG00000000328	TPPP2	10	26128308	26130477	-	CODING	-1.77792	0.005226
ENSBTAT00000008363	ENSBTAG00000006374	NHP2	7	40601549	40605473	-	CODING	0.405769	0.005243
ENSBTAT00000020406	ENSBTAG00000015350	PLIN	21	21502826	21516686	-	CODING	0.989248	0.005283
ENSBTAT00000012827	ENSBTAG00000009725	AOX1	2	89517811	89589090	+	CODING	0.548546	0.005291
ENSBTAT00000006215	ENSBTAG00000004735	CCNE1	18	40565286	40574820	+	CODING	-1.40378	0.005314
ENSBTAT00000065362	ENSBTAG00000048273	-	6	7145414	7150482	+	CODING	0.403424	0.005326
ENSBTAT00000042576	ENSBTAG00000018661	TMC6	19	54647278	54666987	+	CODING	1.860104	0.005326
ENSBTAT00000063498	ENSBTAG00000047183	-	1	20472812	20473172	-	CODING	-3.73625	0.005367
ENSBTAT00000017303	ENSBTAG00000013016	GNAI3	3	33969546	34013930	-	CODING	0.732576	0.005374
ENSBTAT00000008891	ENSBTAG00000009554	EHBP1L1	29	44424071	44439541	+	CODING	-0.54259	0.005385
ENSBTAT00000015631	ENSBTAG00000011772	PPP1R12B	16	71062132	71090113	+	CODING	-1.25711	0.005392
ENSBTAT00000010568	ENSBTAG00000008033	PPP6C	11	96044189	96085315	-	CODING	-0.4073	0.005453
ENSBTAT00000044870	ENSBTAG00000031648	C18H19orf33	18	48337453	48338289	+	CODING	2.155996	0.005528
ENSBTAT00000061126	ENSBTAG00000010392	ESRRG	16	20595768	20789340	-	CODING	1.396378	0.00553
ENSBTAT00000002045	ENSBTAG00000001565	MESDC2	21	27322045	27330423	-	CODING	0.575765	0.005541
ENSBTAT00000023581	ENSBTAG00000017733	CA2	14	79372712	79388600	-	CODING	-0.53871	0.005556
ENSBTAT00000028094	ENSBTAG00000021090	ABCC1	25	14469282	14570639	+	CODING	-0.82319	0.005586
ENSBTAT00000036029	ENSBTAG00000010106	CCND3	23	15707556	15714372	-	CODING	0.757233	0.005611
ENSBTAT00000042662	ENSBTAG00000009131	TTC37	7	97172634	97262280	-	CODING	-0.94495	0.005616
ENSBTAT00000011385	ENSBTAG00000008634	WBP2	19	56394188	56401350	+	CODING	-0.38993	0.005625
ENSBTAT00000014847	ENSBTAG00000011180	ACTR8	22	47591904	47607214	+	CODING	-0.89854	0.005638
ENSBTAT00000056512	ENSBTAG00000039028	PI3	13	74272869	74274940	+	CODING	-2.27744	0.005651
ENSBTAT00000008976	ENSBTAG00000006828	RAPGEF3	5	32697065	32718624	+	CODING	1.144661	0.005657
ENSBTAT00000010394	ENSBTAG00000040392	-	18	61206192	61215875	-	CODING	1.154567	0.005659
ENSBTAT00000025640	ENSBTAG00000013982	UACA	10	17516709	17559258	-	CODING	-0.41204	0.005671
ENSBTAT00000022524	ENSBTAG00000016933	IL27RA	7	12743318	12767182	-	CODING	1.26799	0.005673
ENSBTAT00000025007	ENSBTAG00000018784	CTSZ	13	57889707	57899205	+	CODING	0.861927	0.005684
ENSBTAT00000039259	ENSBTAG00000007718	TGIF1	24	37940878	37948732	+	CODING	1.242514	0.005699
ENSBTAT00000028639	ENSBTAG00000021487	CIART	3	20400969	20404292	-	CODING	-0.42911	0.005705
ENSBTAT00000009070	ENSBTAG00000006904	TENC1	5	27069113	27082523	-	CODING	0.67221	0.005706
ENSBTAT00000002984	ENSBTAG00000022890	MBP	24	2558226	2590450	+	CODING	0.875964	0.005719
ENSBTAT00000027001	ENSBTAG00000020262	PSME4	11	36424173	36524445	-	CODING	-0.35352	0.005721
ENSBTAT00000024584	ENSBTAG00000018469	ALDH6A1	10	85821883	85839587	-	CODING	-0.71968	0.005737
ENSBTAT00000002265	ENSBTAG00000001729	DUSP10	16	25895751	25936856	-	CODING	1.023326	0.005749
ENSBTAT00000012434	ENSBTAG00000009449	ATP5SL	18	50845592	50850432	-	CODING	-0.80213	0.005798
ENSBTAT00000028208	ENSBTAG00000021166	FAM71A	16	72811911	72814133	-	CODING	2.951888	0.005813
ENSBTAT00000013787	ENSBTAG00000010447	LSP1	29	50238211	50276209	-	CODING	0.786944	0.005823
ENSBTAT00000014270	ENSBTAG00000010778	NDUFAF1	10	36938124	36948842	-	CODING	-0.6472	0.005842
ENSBTAT00000016850	ENSBTAG00000012684	CCL19	8	77394838	77396746	-	CODING	2.597982	0.005863
ENSBTAT00000003636	ENSBTAG00000002808	PSMA3	10	70797328	70822594	+	CODING	0.234316	0.005867
ENSBTAT00000011603	ENSBTAG00000008810	-	3	101022239	101054545	+	CODING	-0.51844	0.005878
ENSBTAT00000012636	ENSBTAG00000009602	TMEM120B	17	55772992	55815637	-	CODING	0.720477	0.005882
ENSBTAT00000009496	ENSBTAG00000007217	KIAA0195	19	56679702	56697953	-	CODING	-0.76008	0.005924
ENSBTAT00000012632	ENSBTAG00000009599	LCN1	11	106427895	106430697	+	CODING	3.708969	0.005934
ENSBTAT00000061451	ENSBTAG00000021237	DST	23	3431407	3687495	+	CODING	-0.66698	0.005941
ENSBTAT00000013994	ENSBTAG00000024095	THRAP3	3	110137251	110208496	-	CODING	-0.39939	0.005943
ENSBTAT00000023671	ENSBTAG00000017803	RAB14	8	112502334	112512347	-	CODING	-0.51853	0.005947
ENSBTAT00000028708	ENSBTAG00000021544	EBNA1BP2	3	103422605	103429520	+	CODING	0.549664	0.005953
ENSBTAT00000031993	ENSBTAG00000016313	ECH1	18	48811368	48820509	-	CODING	0.343233	0.005964
ENSBTAT00000004156	ENSBTAG00000003200	-	20	24233178	24292919	+	CODING	-1.69439	0.005985
ENSBTAT00000007177	ENSBTAG00000005455	USP7	25	7769457	7796941	-	CODING	-0.42699	0.005989
ENSBTAT00000065446	ENSBTAG00000046046	MGC160046	24	40563771	40566586	+	CODING	-0.54229	0.00601
ENSBTAT00000015090	ENSBTAG00000011354	NUDT9	6	104099623	104136963	+	CODING	-0.5679	0.006014
ENSBTAT00000002914	ENSBTAG00000002258	APOA1	15	27932200	27934085	-	CODING	1.603358	0.006021
ENSBTAT00000051493	ENSBTAG00000036993	U2	2	48352291	48352468	-	CODING	-3.70513	0.006031
ENSBTAT00000020263	ENSBTAG00000015230	PLA2G12A	6	16828642	16843001	+	CODING	-0.56911	0.006072

ENSBTAT00000000991	ENSBTAG00000000750	MSL2	1	134190685	134220225	+	CODING	-0.93434	0.006133
ENSBTAT00000025198	ENSBTAG00000018921	USP19	22	51470661	51482027	+	CODING	-0.57532	0.006138
ENSBTAT00000015208	ENSBTAG00000011444	HINT2	8	60390945	60393499	-	CODING	0.675001	0.006151
ENSBTAT00000005688	ENSBTAG00000004348	MRPL21	29	46913943	46923884	-	CODING	0.478451	0.006152
ENSBTAT00000063023	ENSBTAG00000045980	ARF6	10	42902260	42902938	+	CODING	0.469188	0.006195
ENSBTAT00000019055	ENSBTAG00000014331	UBL4A	X	40458245	40460872	-	CODING	0.934497	0.006219
ENSBTAT00000020863	ENSBTAG00000015719	PIGF	11	28871452	28902847	-	CODING	-0.45593	0.006247
ENSBTAT00000009715	ENSBTAG00000007390	VAT1	19	43687403	43694870	-	CODING	0.617416	0.006255
ENSBTAT00000028246	ENSBTAG00000021199	APOPT1	21	69847359	69873999	+	CODING	0.478082	0.006305
ENSBTAT00000035230	ENSBTAG00000025161	AGPAT2	11	104134683	104146456	-	CODING	-0.30127	0.006307
ENSBTAT00000035582	ENSBTAG00000025358	SNX2	7	32329451	32388793	-	CODING	0.603662	0.006322
ENSBTAT00000026766	ENSBTAG00000020089	-	19	12986898	13084710	+	CODING	-0.90233	0.006348
ENSBTAT00000015726	ENSBTAG00000024081	ECM2	8	85540501	85579683	-	CODING	0.557638	0.006362
ENSBTAT00000030071	ENSBTAG00000013240	SLC3A2	29	41855871	41896760	+	CODING	-0.31063	0.006394
ENSBTAT00000061454	ENSBTAG00000000838	CCDC88C	21	56629747	56773304	-	CODING	-0.74156	0.006395
ENSBTAT00000002689	ENSBTAG00000002080	NOV	14	47005560	47013935	-	CODING	0.714094	0.006405
ENSBTAT00000005628	ENSBTAG00000004295	NDUFA8	11	93011815	93029730	-	CODING	0.252796	0.006417
ENSBTAT00000008305	ENSBTAG00000006330	RBM5	22	50789615	50813736	-	CODING	0.435403	0.006452
ENSBTAT00000030821	ENSBTAG00000022714	HSPB3	20	24659525	24660204	-	CODING	0.530997	0.006515
ENSBTAT00000013141	ENSBTAG00000009959	SLC6A8	X	39828310	39835611	+	CODING	-0.37584	0.006515
ENSBTAT00000035789	ENSBTAG00000019685	BAG6	23	27463438	27474655	+	CODING	-0.44452	0.006542
ENSBTAT00000024988	ENSBTAG00000018767	RTP3	22	53492964	53495676	-	CODING	-1.02723	0.006667
ENSBTAT00000048411	ENSBTAG00000015512	-	20	6721327	6753670	-	CODING	1.216503	0.006692
ENSBTAT00000014218	ENSBTAG00000010740	CLTB	7	39210710	39230761	-	CODING	0.306533	0.006694
ENSBTAT00000011803	ENSBTAG00000008967	RAP1B	5	45355308	45399175	-	CODING	0.550796	0.006703
ENSBTAT00000014529	ENSBTAG00000010940	HSPB7	2	136635160	136637353	+	CODING	-0.13926	0.00674
ENSBTAT00000011106	ENSBTAG00000008441	SOCS3	19	54458856	54459555	+	CODING	1.03498	0.006749
ENSBTAT00000011533	ENSBTAG00000008755	42801	2	36682216	36729906	-	CODING	0.356318	0.006751
ENSBTAT00000018461	ENSBTAG00000013901	PTDSS1	14	70236070	70303427	-	CODING	-0.49979	0.00677
ENSBTAT00000004563	ENSBTAG00000003510	MMRN2	28	41886524	41901491	-	CODING	1.036965	0.006772
ENSBTAT00000049099	ENSBTAG00000034662	-	9	18473883	18475244	+	CODING	-0.32076	0.006811
ENSBTAT00000061327	ENSBTAG00000021073	KIAA1549	4	103305514	103380070	-	CODING	-2.83236	0.00688
ENSBTAT00000020094	ENSBTAG00000015100	ATP6V0E1	20	4641618	4671634	+	CODING	0.402756	0.006882
ENSBTAT00000010351	ENSBTAG00000007867	STAT1	2	79888206	79927096	-	CODING	-0.52598	0.006913
ENSBTAT00000045136	ENSBTAG00000014295	CAPZA1	3	30810109	30854190	-	CODING	0.482142	0.006933
ENSBTAT00000009410	ENSBTAG00000007152	OS9	5	56070693	56104924	-	CODING	0.441202	0.006941
ENSBTAT00000013932	ENSBTAG00000045785	TOM1	5	73905663	73946203	+	CODING	-0.61484	0.006952
ENSBTAT00000014537	ENSBTAG00000010948	CLIC2	X	38498587	38523525	+	CODING	1.101411	0.006953
ENSBTAT00000049112	ENSBTAG00000009279	TNKS2	26	13383833	13446978	+	CODING	-0.80855	0.006955
ENSBTAT00000004885	ENSBTAG00000003752	SLC25A24	3	35152469	35210446	+	CODING	1.018358	0.006965
ENSBTAT00000007890	ENSBTAG00000006007	SH3GL1	7	20923762	20951051	+	CODING	0.849032	0.00697
ENSBTAT00000020443	ENSBTAG00000015381	ARHGAP18	9	68713610	68777064	-	CODING	-1.13821	0.006978
ENSBTAT00000045223	ENSBTAG00000031885	TMEM251	21	58404957	58407259	+	CODING	0.814067	0.006979
ENSBTAT00000005590	ENSBTAG00000004268	TRAPPC5	7	17750387	17752622	+	CODING	0.580467	0.007033
ENSBTAT00000020020	ENSBTAG00000015038	HADHA	11	73246485	73288803	+	CODING	0.232796	0.007035
ENSBTAT00000006992	ENSBTAG00000005316	GDI2	13	43302440	43331090	+	CODING	-0.24541	0.007051
ENSBTAT00000005655	ENSBTAG00000004318	ARL3	26	23563111	23761992	-	CODING	0.764401	0.007083
ENSBTAT00000006039	ENSBTAG00000004602	PITX1	7	48063871	48069063	-	CODING	2.614157	0.007099
ENSBTAT00000011543	ENSBTAG00000008763	PRR32	X	10981501	10983415	+	CODING	1.030668	0.007113
ENSBTAT00000015413	ENSBTAG00000011600	MAP3K8	13	35600313	35626614	+	CODING	2.164108	0.007119
ENSBTAT00000025536	ENSBTAG00000019187	ZNF462	8	98331864	98426787	+	CODING	-1.03764	0.007173
ENSBTAT00000064288	ENSBTAG00000046566	-	3	27428793	27430601	-	CODING	-0.98106	0.007249
ENSBTAT00000001140	ENSBTAG00000000855	NXN	19	22457712	22621493	+	CODING	-0.50658	0.007267
ENSBTAT00000002867	ENSBTAG00000002215	GFPT2	7	772145	817756	+	CODING	1.240052	0.007268
ENSBTAT00000018334	ENSBTAG00000005412	NEDD4L	24	57965974	58053921	+	CODING	-0.53774	0.007286
ENSBTAT00000024107	ENSBTAG00000018115	PEBP1	17	59333429	59337958	-	CODING	-0.18966	0.007329
ENSBTAT00000004985	ENSBTAG00000003825	PTPN12	4	43834354	43884954	-	CODING	0.723545	0.007392
ENSBTAT00000019738	ENSBTAG00000014822	MRPL52	10	21816260	21821165	-	CODING	0.583123	0.007422
ENSBTAT00000012920	ENSBTAG00000009795	SF3B5	9	82549526	82550198	-	CODING	0.489126	0.007445
ENSBTAT00000056287	ENSBTAG00000001361	NMNAT1	16	44457295	44462940	-	CODING	-0.67387	0.007476
ENSBTAT00000028717	ENSBTAG00000021553	PTPN14	16	70619178	70749808	+	CODING	-0.84624	0.00748
ENSBTAT00000026386	ENSBTAG00000019803	POLR3K	25	110168	112894	-	CODING	0.241448	0.0075
ENSBTAT00000001526	ENSBTAG00000001146	HIVEP2	9	81237678	81266493	-	CODING	-0.67811	0.00754
ENSBTAT00000046195	ENSBTAG00000032557	TMEM258	29	40927662	40931388	-	CODING	0.381924	0.007558

ENSBTAT00000009974	ENSBTAG00000007581	ADORA2B	19	34084460	34104983	-	CODING	1.387222	0.007583
ENSBTAT00000002822	ENSBTAG00000002178	PPL	25	3966800	4014117	-	CODING	1.342794	0.007602
ENSBTAT00000016033	ENSBTAG00000012090	GGTA1	11	92297493	92374682	-	CODING	0.540831	0.007605
ENSBTAT00000008971	ENSBTAG00000006824	APPL1	22	44397100	44427704	-	CODING	-0.58564	0.007605
ENSBTAT00000027890	ENSBTAG00000020939	PLAC9	28	35366025	35378318	-	CODING	1.261754	0.007607
ENSBTAT00000019753	ENSBTAG00000014831	PPP1R3C	26	13270026	13274922	-	CODING	-0.3322	0.007614
ENSBTAT00000025572	ENSBTAG00000019211	BRIX1	20	39256327	39265218	-	CODING	0.769334	0.007614
ENSBTAT00000025973	ENSBTAG00000006287	NEDD9	23	44851677	44988905	+	CODING	0.927555	0.007672
ENSBTAT00000028490	ENSBTAG00000021370	BLMH	19	21896194	21936565	-	CODING	-0.54022	0.007675
ENSBTAT00000021658	ENSBTAG00000016281	PREP	9	45169675	45301623	+	CODING	-0.37737	0.007681
ENSBTAT00000048277	ENSBTAG00000034033	LHFP	12	22819645	23026440	+	CODING	0.653534	0.007684
ENSBTAT00000056635	ENSBTAG00000038195	CD302	2	36656333	36681937	+	CODING	0.713586	0.007731
ENSBTAT00000034095	ENSBTAG00000017852	ALDH9A1	3	3252171	3281221	+	CODING	0.840787	0.007766
ENSBTAT00000039622	ENSBTAG00000018479	PTPMT1	15	78571082	78576838	+	CODING	0.723954	0.007785
ENSBTAT00000002225	ENSBTAG00000001697	TRA2B	1	81878691	81898384	+	CODING	0.393645	0.00779
ENSBTAT00000008198	ENSBTAG00000006246	CBX5	5	25944853	25971390	+	CODING	-0.47282	0.007815
ENSBTAT00000006430	ENSBTAG00000004887	DCTN1	11	10235834	10253077	+	CODING	-0.50039	0.007829
ENSBTAT00000018206	ENSBTAG00000013699	TBC1D1	6	58848558	59065295	+	CODING	0.962916	0.007832
ENSBTAT00000033670	ENSBTAG00000010161	CCL21	8	77421389	77422532	-	CODING	1.056934	0.007836
ENSBTAT00000001698	ENSBTAG00000001288	MAOB	X	105235855	105359152	+	CODING	-0.76332	0.007847
ENSBTAT00000003472	ENSBTAG00000002683	PFKP	13	45500599	45553520	-	CODING	1.190877	0.007853
ENSBTAT00000000734	ENSBTAG00000000561	OCLN	20	10154909	10201157	-	CODING	1.26407	0.007853
ENSBTAT00000010916	ENSBTAG00000008296	N6AMT2	12	36055182	36075245	+	CODING	0.618375	0.007859
ENSBTAT00000027067	ENSBTAG00000020309	G3BP	7	64976048	65004834	+	CODING	-0.50088	0.007866
ENSBTAT00000061383	ENSBTAG00000013616	AP4E1	10	59578464	59644086	-	CODING	-0.97188	0.007904
ENSBTAT00000037576	ENSBTAG00000032450	-	X	57583910	57584338	+	CODING	0.403302	0.007972
ENSBTAT00000063564	ENSBTAG00000021697	PDGFB	5	111180170	111200490	-	CODING	0.694642	0.007976
ENSBTAT00000011453	ENSBTAG00000008688	HEYL	3	107032468	107047976	+	CODING	0.746162	0.007983
ENSBTAT00000027614	ENSBTAG00000020721	MRPL55	7	2905629	2909407	+	CODING	0.332742	0.007986
ENSBTAT00000024078	ENSBTAG00000018088	SETBP1	24	45025456	45141793	+	CODING	-1.40078	0.008
ENSBTAT00000066104	ENSBTAG00000048172	-	X	142665000	142690152	-	CODING	1.447895	0.008046
ENSBTAT00000019452	ENSBTAG00000014608	KDM1B	23	39166819	39204690	-	CODING	-1.11781	0.008051
ENSBTAT00000043155	ENSBTAG00000013926	FCGRT	18	56415980	56421861	+	CODING	2.12546	0.008052
ENSBTAT00000019259	ENSBTAG00000014482	FAF1	3	95937279	96434760	+	CODING	-0.65063	0.008084
ENSBTAT00000009415	ENSBTAG00000007153	C1QA	2	130792855	130795743	-	CODING	1.288959	0.008098
ENSBTAT00000002959	ENSBTAG00000002293	ARL5A	2	44468905	44491772	+	CODING	-0.51839	0.008239
ENSBTAT00000007341	ENSBTAG00000005589	STK19	23	27197602	27206845	-	CODING	1.092429	0.008265
ENSBTAT00000003758	ENSBTAG00000002890	RBM10	X	90794365	90820965	+	CODING	0.635097	0.008337
ENSBTAT00000004843	ENSBTAG00000003718	HACL1	1	154337506	154376271	-	CODING	0.534395	0.008359
ENSBTAT00000000453	ENSBTAG00000000347	RHOG	15	52061683	52075146	+	CODING	0.574293	0.008365
ENSBTAT00000009005	ENSBTAG00000006846	LGALS9	19	19738612	19757359	+	CODING	0.700499	0.008384
ENSBTAT00000001890	ENSBTAG00000001441	CARHSP1	25	7734591	7746003	-	CODING	1.233265	0.008396
ENSBTAT00000006244	ENSBTAG00000004757	LTBP4	18	50173268	50202104	+	CODING	0.508492	0.008404
ENSBTAT00000053555	ENSBTAG00000018922	TRMT10B	8	62328443	62339566	+	CODING	0.880777	0.008434
ENSBTAT00000033371	ENSBTAG00000024182	H2B	23	30730958	30731338	+	CODING	-3.57654	0.008451
ENSBTAT00000002274	ENSBTAG00000001736	UBN1	25	3936573	3964092	+	CODING	-1.05853	0.008578
ENSBTAT00000013781	ENSBTAG00000010439	AKAP8L	7	8754284	8787049	+	CODING	0.505278	0.008581
ENSBTAT00000003599	ENSBTAG00000002769	AP1G1	18	39401561	39483417	+	CODING	-0.73376	0.008594
ENSBTAT00000004304	ENSBTAG00000003322	TTLL7	3	60357482	60449087	+	CODING	-0.89179	0.008595
ENSBTAT00000055249	ENSBTAG00000011613	PLS3	X	71709936	71806536	+	CODING	0.541119	0.008642
ENSBTAT00000026443	ENSBTAG00000019847	CPEB2	6	114969373	115035177	+	CODING	-0.78656	0.008644
ENSBTAT00000052954	ENSBTAG00000032007	LRP11	9	88115145	88183314	-	CODING	-1.0813	0.008669
ENSBTAT00000047009	ENSBTAG00000006835	MCAM	15	30414474	30422213	-	CODING	0.681919	0.008676
ENSBTAT00000015485	ENSBTAG00000011662	SOX18	13	54295399	54296819	+	CODING	0.944461	0.00869
ENSBTAT00000062519	ENSBTAG00000045086	7SK	7	41115463	41115731	-	CODING	-3.60304	0.0087
ENSBTAT00000061287	ENSBTAG00000038920	VPS13C	10	48088919	48268869	+	CODING	-1.09165	0.00871
ENSBTAT00000011234	ENSBTAG00000027442	NFIB	8	30029740	30155742	+	CODING	-0.67673	0.008723
ENSBTAT00000019193	ENSBTAG00000014429	KMT2D	5	30931917	30964080	+	CODING	-0.86055	0.008771
ENSBTAT00000063852	ENSBTAG00000047880	-	2	31688429	31699171	+	CODING	1.348181	0.008873
ENSBTAT00000017002	ENSBTAG00000012797	DCUN1D1	1	84656319	84689038	+	CODING	-0.27598	0.008901
ENSBTAT00000012136	ENSBTAG00000009211	UBE2S	18	62537351	62541236	+	CODING	0.635651	0.008976
ENSBTAT00000000114	ENSBTAG00000000105	RIT1	3	14908141	14917339	+	CODING	0.686223	0.008979
ENSBTAT00000018091	ENSBTAG00000013606	FKBP2	29	43159739	43162644	+	CODING	0.570387	0.008983
ENSBTAT00000063472	ENSBTAG00000032427	FHOD1	18	34977246	34993385	-	CODING	-0.43348	0.009053

ENSBTAT00000011581	ENSBTAG00000008789	-	7	21575113	21577011	+	CODING	0.724623	0.009058
ENSBTAT00000003725	ENSBTAG00000002871	COPS6	25	36893605	36896370	-	CODING	0.378537	0.009106
ENSBTAT00000010492	ENSBTAG00000007981	GRK5	26	39702550	39930993	+	CODING	1.10365	0.009187
ENSBTAT00000039462	ENSBTAG00000027477	B3GAT1	15	85014888	85023350	-	CODING	-1.18848	0.009222
ENSBTAT00000048322	ENSBTAG00000034077	ASIP	13	64234645	64239783	+	CODING	-0.69079	0.009232
ENSBTAT00000009490	ENSBTAG00000007210	RBFOX1	25	6481524	6686788	+	CODING	-0.53678	0.009252
ENSBTAT00000006412	ENSBTAG00000004876	RABL2B	5	120246487	120264932	+	CODING	0.835529	0.009263
ENSBTAT00000021556	ENSBTAG00000016198	EP300	5	112802547	112864774	+	CODING	-0.68747	0.009331
ENSBTAT00000014907	ENSBTAG00000011224	CITED2	9	78071006	78073397	-	CODING	0.482998	0.009385
ENSBTAT00000000669	ENSBTAG00000000516	OTOR	13	10760673	10763286	+	CODING	2.975168	0.009406
ENSBTAT00000049126	ENSBTAG00000034689	ZNRF1	18	2396335	2482363	+	CODING	-0.42915	0.009447
ENSBTAT00000052079	ENSBTAG00000000484	HYAL2	22	50593627	50597453	+	CODING	1.217376	0.009467
ENSBTAT00000063294	ENSBTAG00000046484	AIF1L	11	101335452	101343787	+	CODING	0.855621	0.009512
ENSBTAT00000014098	ENSBTAG00000010659	CUX1	25	35243348	35515094	-	CODING	-0.67015	0.00953
ENSBTAT00000010251	ENSBTAG00000007791	KCNQ5	9	12697727	12864358	+	CODING	-1.44485	0.009533
ENSBTAT00000063486	ENSBTAG00000001007	SLC25A43	X	3646867	3686722	+	CODING	-1.39136	0.009547
ENSBTAT00000013382	ENSBTAG00000010138	SEMA3B	22	50655648	50661989	-	CODING	1.297678	0.009557
ENSBTAT00000010790	ENSBTAG00000008204	C9orf41	8	51344227	51383813	-	CODING	-0.78219	0.009557
ENSBTAT00000063435	ENSBTAG00000045772	-	9	95165926	95166855	-	CODING	0.347303	0.009561
ENSBTAT00000006650	ENSBTAG00000005040	SMEK1	21	56800878	56835145	-	CODING	0.490891	0.009601
ENSBTAT00000035362	ENSBTAG00000027980	TAF9	20	10300612	10304139	+	CODING	0.438283	0.009616
ENSBTAT00000007519	ENSBTAG00000005718	PLIN2	8	25129168	25137851	+	CODING	1.683491	0.009662
ENSBTAT00000001094	ENSBTAG00000000828	CAPN6	X	64684214	64708869	-	CODING	1.030805	0.009668
ENSBTAT00000038842	ENSBTAG00000001514	ASB11	X	135436138	135465103	+	CODING	-0.31617	0.009748
ENSBTAT00000027051	ENSBTAG00000020297	PQLC3	11	86588329	86601250	-	CODING	0.963087	0.009773
ENSBTAT00000007165	ENSBTAG00000005446	POLR2J	25	35091300	35095676	+	CODING	0.332912	0.009815
ENSBTAT00000016376	ENSBTAG00000012342	LIMA1	5	29804689	29898282	+	CODING	0.965439	0.009817
ENSBTAT00000014162	ENSBTAG00000010701	NACA	5	57063071	57074070	+	CODING	0.2833	0.009822
ENSBTAT00000010384	ENSBTAG00000007895	SLC20A1	11	46218716	46232124	+	CODING	0.470111	0.009832
ENSBTAT00000065547	ENSBTAG00000046498	-	25	42389844	42410734	-	CODING	-1.1714	0.009847
ENSBTAT00000027260	ENSBTAG00000020456	AGPAT3	1	146705613	146735094	+	CODING	-0.59254	0.009848
ENSBTAT00000011076	ENSBTAG00000008417	VPS29	17	56617708	56622846	-	CODING	0.407258	0.009856
ENSBTAT00000015905	ENSBTAG00000011986	PLSCR4	1	123231176	123273104	+	CODING	0.845097	0.009934
ENSBTAT00000032910	ENSBTAG00000023929	FOSL2	11	71329772	71349193	-	CODING	0.717322	0.009954
ENSBTAT00000022232	ENSBTAG00000016724	NPEPL1	13	58207245	58222951	-	CODING	-0.42188	0.009972
ENSBTAT00000047200	ENSBTAG00000033221	CCDC152	20	31793082	31834092	-	CODING	0.566184	0.01001
ENSBTAT00000007361	ENSBTAG00000005606	HOXC9	5	26188849	26191440	-	CODING	0.652582	0.010074
ENSBTAT00000065501	ENSBTAG00000045937	-	4	17767925	17769016	+	CODING	0.281899	0.010078
ENSBTAT00000056716	ENSBTAG00000015910	ITGB1	13	20248945	20292114	+	CODING	0.292112	0.010165
ENSBTAT00000011028	ENSBTAG00000008378	FEM1B	10	15080222	15092340	+	CODING	-0.55499	0.010168
ENSBTAT00000033470	ENSBTAG00000024240	ACADM	3	69344157	69382504	-	CODING	0.388729	0.010181
ENSBTAT00000009609	ENSBTAG00000007304	CLK3	21	34438146	34452116	-	CODING	0.56417	0.01019
ENSBTAT00000000447	ENSBTAG00000000342	PLOD3	25	36057472	36065405	+	CODING	0.849646	0.010191
ENSBTAT00000047757	ENSBTAG00000020693	DERL1	14	18307140	18329318	+	CODING	-0.65473	0.010207
ENSBTAT00000003277	ENSBTAG00000002526	BDH2	6	23047057	23077431	+	CODING	-0.56653	0.010221
ENSBTAT00000065391	ENSBTAG00000007791	KCNQ5	9	12750806	12785144	+	CODING	-3.74979	0.010255
ENSBTAT00000021204	ENSBTAG00000015946	RBBP5	16	2694854	2733979	-	CODING	-0.62475	0.010262
ENSBTAT00000004402	ENSBTAG00000003396	MAFB	13	70068210	70069181	-	CODING	-1.10638	0.010293
ENSBTAT00000029015	ENSBTAG00000021766	HBEGF	7	53216665	53227848	-	CODING	0.571506	0.010461
ENSBTAT00000064557	ENSBTAG00000045954	LRRC14B	20	71984463	71989365	-	CODING	-0.9903	0.010469
ENSBTAT00000014449	ENSBTAG00000010877	ARMC12	23	9683947	9694015	+	CODING	0.926517	0.010487
ENSBTAT00000015235	ENSBTAG00000011465	MYBPH	16	760056	768350	-	CODING	-0.37631	0.0105
ENSBTAT00000002464	ENSBTAG00000001894	NEDD1	5	61446535	61498524	+	CODING	-0.45778	0.01053
ENSBTAT00000044482	ENSBTAG00000040367	-	2	92993922	92995706	-	CODING	-4.79403	0.010549
ENSBTAT00000026526	ENSBTAG00000019910	SDCBP	14	26412824	26447192	+	CODING	0.451197	0.010583
ENSBTAT00000011242	ENSBTAG00000008528	SLC25A1	17	74633475	74635952	-	CODING	0.493676	0.010635
ENSBTAT00000012855	ENSBTAG00000009748	MFRP	15	30450602	30455935	-	CODING	4.562018	0.010646
ENSBTAT00000003434	ENSBTAG00000032899	-	5	29080926	29082000	-	CODING	-0.92549	0.010657
ENSBTAT00000052103	ENSBTAG00000013468	AHNAK	29	41576805	41605018	-	CODING	-0.33664	0.010663
ENSBTAT00000017242	ENSBTAG00000012969	DKK2	6	19381413	19511148	+	CODING	0.953531	0.010761
ENSBTAT00000009243	ENSBTAG00000007024	-	5	104077163	104077952	-	CODING	1.110259	0.010767
ENSBTAT00000012174	ENSBTAG00000009238	HOXC6	5	26161892	26163475	-	CODING	-0.47378	0.010775
ENSBTAT00000013500	ENSBTAG00000010227	CPSF2	21	57395846	57429002	+	CODING	-0.71284	0.010781
ENSBTAT00000000579	ENSBTAG00000000455	CREBZF	29	9952633	9958039	+	CODING	0.574891	0.010895

ENSBTAT00000040084	ENSBTAG00000014713	RARRES1	1	109661962	109704520	+	CODING	1.954788	0.010956
ENSBTAT00000003832	ENSBTAG00000002944	ATP5G3	2	21702150	21705260	+	CODING	-0.19625	0.010957
ENSBTAT00000008650	ENSBTAG00000006586	KPNB1	19	39409895	39431772	-	CODING	-0.39714	0.010979
ENSBTAT00000026387	ENSBTAG00000019804	SNRNP25	25	113120	115274	+	CODING	0.290203	0.011005
ENSBTAT00000016160	ENSBTAG00000009789	GNAQ	8	53970972	54280697	-	CODING	-0.7358	0.011024
ENSBTAT00000063911	ENSBTAG00000047424	TRIP10	7	18967189	18970399	-	CODING	0.349115	0.011025
ENSBTAT00000026239	ENSBTAG00000019684	NUDT3	23	8297778	8414740	-	CODING	-0.37794	0.011058
ENSBTAT00000044457	ENSBTAG00000003556	ZFYVE21	21	69978023	69980890	+	CODING	0.904585	0.011202
ENSBTAT00000032902	ENSBTAG00000002261	LBX1	26	21894402	21896298	-	CODING	-1.52511	0.011269
ENSBTAT00000013461	ENSBTAG00000010195	PKN3	11	99232119	99246139	+	CODING	1.332469	0.011383
ENSBTAT00000023496	ENSBTAG00000017666	ABCC5	1	83765087	83817531	+	CODING	0.567361	0.011431
ENSBTAT00000037763	ENSBTAG00000009251	SZRD1	2	136317925	136337845	-	CODING	-0.8164	0.011444
ENSBTAT00000026753	ENSBTAG00000020079	MAP3K4	9	98162838	98232658	+	CODING	-0.69498	0.011501
ENSBTAT00000046341	ENSBTAG00000006101	PSMD4	3	19598474	19607504	-	CODING	-0.33632	0.011574
ENSBTAT00000018741	ENSBTAG00000014093	IDH2	21	21903847	21920153	-	CODING	0.191802	0.011708
ENSBTAT00000015643	ENSBTAG00000011786	CLUH	19	24168990	24181331	-	CODING	-0.59047	0.011814
ENSBTAT00000022411	ENSBTAG00000016846	YWHAB	13	74018886	74040819	+	CODING	0.401449	0.011859
ENSBTAT00000009422	ENSBTAG00000007159	ESR1	9	89969586	90255801	+	CODING	-1.06692	0.011913
ENSBTAT00000011448	ENSBTAG00000008686	SLC46A3	12	31463869	31481900	+	CODING	-0.93255	0.011923
ENSBTAT00000064941	ENSBTAG00000047490	-	2	99704435	99714269	+	CODING	-3.05915	0.011932
ENSBTAT00000012170	ENSBTAG00000009233	RAC1	25	38832485	38841319	+	CODING	0.209454	0.011944
ENSBTAT00000033040	ENSBTAG00000023997	VAMP8	11	49260841	49264015	-	CODING	0.7946	0.011969
ENSBTAT00000026146	ENSBTAG00000019627	THY1	15	30509504	30515399	-	CODING	0.719686	0.012112
ENSBTAT00000005730	ENSBTAG00000004371	CS	5	57366008	57389596	+	CODING	-0.20508	0.012123
ENSBTAT00000036447	ENSBTAG00000025778	EMC9	10	20844420	20846694	+	CODING	0.391466	0.012142
ENSBTAT00000003319	ENSBTAG00000002563	WDR45	X	92234971	92240178	-	CODING	0.506328	0.012146
ENSBTAT00000039408	ENSBTAG00000027442	NFIB	8	30010801	30128359	+	CODING	-0.88048	0.012166
ENSBTAT00000027322	ENSBTAG00000020504	CDK2AP1	17	54556011	54566572	+	CODING	-0.32095	0.012196
ENSBTAT00000000859	ENSBTAG00000000656	NFATC1	24	1018253	1099817	-	CODING	-0.79612	0.012238
ENSBTAT00000012128	ENSBTAG00000009206	FOXS1	13	61924322	61925629	-	CODING	0.856981	0.012242
ENSBTAT00000026289	ENSBTAG00000019725	UBE4A	15	29476822	29516720	+	CODING	-0.39028	0.01232
ENSBTAT00000049975	ENSBTAG00000033389	-	25	1167351	1168312	+	CODING	0.849767	0.012346
ENSBTAT00000032947	ENSBTAG00000023947	AVPI1	26	18711832	18719926	-	CODING	0.718559	0.012359
ENSBTAT00000044775	ENSBTAG00000031583	NUPR1L	25	28027757	28028658	-	CODING	1.243559	0.012375
ENSBTAT00000063105	ENSBTAG00000048173	SMIM10	X	18627968	18629813	+	CODING	1.148377	0.012382
ENSBTAT00000066271	ENSBTAG00000047483	-	10	27081655	27082170	-	CODING	-1.46888	0.012409
ENSBTAT00000014053	ENSBTAG00000010624	DCTN2	5	56262302	56275883	+	CODING	0.276828	0.012422
ENSBTAT00000020864	ENSBTAG00000015717	HEY1	14	45126102	45128997	-	CODING	0.981302	0.012429
ENSBTAT00000016650	ENSBTAG00000012544	IFNGR1	9	76092204	76116593	-	CODING	0.402823	0.012482
ENSBTAT00000019939	ENSBTAG00000037605	BOLA-DQA1	23	25426330	25430097	-	CODING	-0.69456	0.012508
ENSBTAT00000017739	ENSBTAG00000013340	BAX	18	55985202	55989378	+	CODING	1.187961	0.012535
ENSBTAT00000000479	ENSBTAG00000000369	EPS8	5	94673755	94749093	+	CODING	0.613805	0.012577
ENSBTAT00000061618	ENSBTAG00000044038	TEN1	19	56279922	56300266	-	CODING	0.464402	0.012605
ENSBTAT00000005174	ENSBTAG00000003967	OTUB1	29	42882593	42889928	+	CODING	0.493219	0.012609
ENSBTAT00000043956	ENSBTAG00000017805	CRACR2B	29	50710076	50739409	-	CODING	1.243193	0.012636
ENSBTAT00000046665	ENSBTAG00000013555	ACVR1B	5	28038532	28070153	-	CODING	-1.31528	0.012668
ENSBTAT00000047710	ENSBTAG00000010987	NFKBIZ	1	46543487	46554479	+	CODING	1.291591	0.012744
ENSBTAT00000007442	ENSBTAG00000005664	YWHAE	19	23086308	23121103	-	CODING	-0.12911	0.012754
ENSBTAT00000000930	ENSBTAG00000000695	UBAP2	8	76668494	76759843	-	CODING	-0.55566	0.012826
ENSBTAT00000021878	ENSBTAG00000016455	CACHD1	3	81120400	81223921	-	CODING	-1.31175	0.012832
ENSBTAT00000024345	ENSBTAG00000018292	MOSPD2	X	135804051	135892336	-	CODING	0.939242	0.012841
ENSBTAT00000029502	ENSBTAG00000013152	NIPSNAP1	17	70869039	70884644	-	CODING	-1.04622	0.012859
ENSBTAT00000064335	ENSBTAG00000017770	-	4	103715432	103787300	+	CODING	0.618621	0.012863
ENSBTAT00000062965	ENSBTAG00000000569	HES1	1	73973533	73976720	-	CODING	-2.80058	0.012882
ENSBTAT00000065380	ENSBTAG00000047718	ZNF70	17	73195725	73197071	-	CODING	-1.53353	0.012897
ENSBTAT00000014920	ENSBTAG00000011234	FOXO3	9	42008606	42118673	-	CODING	-0.56027	0.012899
ENSBTAT00000001658	ENSBTAG00000001254	ANKRD46	14	65992659	66033894	+	CODING	-0.53396	0.012991
ENSBTAT00000061313	ENSBTAG00000043951	IMPA2	24	43189865	43209021	+	CODING	0.957543	0.013005
ENSBTAT00000000824	ENSBTAG00000000625	SMAD6	10	13507839	13583291	+	CODING	1.517768	0.013047
ENSBTAT00000061515	ENSBTAG00000002528	EIF3A	26	39585658	39616179	-	CODING	-0.31194	0.013071
ENSBTAT00000036838	ENSBTAG00000006843	UTP15	20	7973680	7992443	-	CODING	-0.75835	0.013106
ENSBTAT00000024336	ENSBTAG00000018286	RNF181	11	49244485	49246356	-	CODING	0.370429	0.013127
ENSBTAT00000002628	ENSBTAG00000002028	MGC143035	3	50658412	50787190	+	CODING	-0.71654	0.013183
ENSBTAT00000026578	ENSBTAG00000019952	ARMC8	1	131883675	131990621	-	CODING	-0.59417	0.013213

ENSBTAT00000004062	ENSBTAG00000003124	HIATL1	8	30529	228065	+	CODING	0.885788	0.013222
ENSBTAT00000027864	ENSBTAG00000000874	CAST	7	98445837	98581253	+	CODING	-0.82708	0.013236
ENSBTAT00000016020	ENSBTAG00000012082	TNFRSF12A	25	2439751	2441210	+	CODING	0.747893	0.01328
ENSBTAT00000005555	ENSBTAG00000004242	ARPC1A	25	37564390	37587556	-	CODING	0.333489	0.0133
ENSBTAT00000033862	ENSBTAG00000021307	BNIP3L	8	74924184	74947549	+	CODING	0.691168	0.013305
ENSBTAT00000007982	ENSBTAG00000006075	SUCLG1	11	50400445	50453412	+	CODING	0.170703	0.013369
ENSBTAT00000007374	ENSBTAG00000005614	UXS1	11	45583095	45639673	+	CODING	-0.32714	0.013392
ENSBTAT00000036062	ENSBTAG00000012384	TFEB	23	15507488	15514052	-	CODING	-0.66904	0.013614
ENSBTAT00000021580	ENSBTAG00000032369	NMI	2	44930495	44950481	+	CODING	1.339363	0.01364
ENSBTAT00000023373	ENSBTAG00000017574	LMNA	3	14696144	14715262	-	CODING	0.397304	0.013662
ENSBTAT00000064713	ENSBTAG00000046008	-	1	120714267	120714944	+	CODING	0.69109	0.013702
ENSBTAT00000042610	ENSBTAG00000009667	SLC25A46	7	112332877	112355108	+	CODING	-0.62003	0.013748
ENSBTAT00000011696	ENSBTAG00000008884	MAPRE3	11	72598140	72653468	-	CODING	-0.25565	0.013752
ENSBTAT00000033053	ENSBTAG00000024000	ATOH8	11	48954369	48996567	-	CODING	0.901018	0.013757
ENSBTAT00000014994	ENSBTAG00000011285	TRMT1L	16	67618111	67653844	-	CODING	-0.41982	0.013762
ENSBTAT00000017663	ENSBTAG00000013282	NECAP2	2	136252384	136266397	-	CODING	0.835265	0.013763
ENSBTAT00000012064	ENSBTAG00000009156	CAMK2N1	2	132751703	132754808	+	CODING	0.851549	0.013796
ENSBTAT00000026068	ENSBTAG00000019565	NCOA4	28	44419336	44441664	-	CODING	-0.43267	0.013806
ENSBTAT00000056479	ENSBTAG00000039954	COPZ2	19	39081066	39090187	+	CODING	0.532712	0.013814
ENSBTAT00000018815	ENSBTAG00000014159	TTL	11	46073891	46107247	+	CODING	-0.83538	0.01384
ENSBTAT00000003937	ENSBTAG00000003027	EMX2	26	38163825	38170070	+	CODING	1.994149	0.01391
ENSBTAT00000028498	ENSBTAG00000021377	S100A14	3	16827337	16829392	+	CODING	0.369197	0.013912
ENSBTAT00000027212	ENSBTAG00000020420	AP1S2	X	134965647	134991291	+	CODING	0.893584	0.013947
ENSBTAT00000008169	ENSBTAG00000006222	TFDP2	1	127957104	128027239	+	CODING	-0.98977	0.013962
ENSBTAT00000000177	ENSBTAG00000000154	IRF2BP1	X	11966614	11968272	+	CODING	-0.71043	0.013978
ENSBTAT00000030644	ENSBTAG00000021853	IP6K2	22	51747919	51771674	+	CODING	0.782829	0.014042
ENSBTAT00000052061	ENSBTAG00000038462	TMEM182	11	7449519	7492859	+	CODING	-0.27694	0.014063
ENSBTAT00000012587	ENSBTAG00000009569	DOCK6	7	16851754	16898876	-	CODING	0.947688	0.014106
ENSBTAT00000002844	ENSBTAG00000002191	RRP12	26	18468764	18497554	-	CODING	-0.56607	0.014153
ENSBTAT00000008068	ENSBTAG00000006134	DYNLRB1	13	64466198	64483930	+	CODING	0.272915	0.014173
ENSBTAT00000004892	ENSBTAG00000003758	TKT	22	48264511	48288059	+	CODING	0.737043	0.01423
ENSBTAT00000027695	ENSBTAG00000020782	CNOT1	18	26390076	26447574	-	CODING	-0.46269	0.014267
ENSBTAT00000061493	ENSBTAG00000000288	UPF2	13	12363666	12452723	+	CODING	-0.55647	0.0143
ENSBTAT00000024063	ENSBTAG00000018074	VPS72	3	19666793	19678632	+	CODING	0.576757	0.01433
ENSBTAT00000008508	ENSBTAG00000006495	GNB2	25	36446227	36451406	-	CODING	0.468173	0.014398
ENSBTAT00000027256	ENSBTAG00000020454	-	13	52494961	52500593	+	CODING	-0.83804	0.014429
ENSBTAT00000064827	ENSBTAG00000046019	SDHA	20	71940749	71964107	-	CODING	-0.18766	0.014467
ENSBTAT00000064981	ENSBTAG00000046019	SDHA	20	71940743	71964081	-	CODING	2.729194	0.014518
ENSBTAT00000020277	ENSBTAG00000015240	UBR4	2	134039040	134176134	+	CODING	-0.79403	0.014522
ENSBTAT00000023908	ENSBTAG00000017967	PTGES3	5	57091367	57112468	+	CODING	-0.16786	0.014573
ENSBTAT00000039096	ENSBTAG00000000817	SYNJ2	9	96023880	96091443	+	CODING	1.858857	0.014614
ENSBTAT00000008774	ENSBTAG00000006672	SYNCRIP	9	64722282	64744803	+	CODING	-0.35506	0.014617
ENSBTAT00000003616	ENSBTAG00000002791	SEC24C	28	29812282	29832215	+	CODING	-0.54323	0.014627
ENSBTAT00000066261	ENSBTAG00000003985	CCDC15	29	28788284	28837160	+	CODING	2.941801	0.014627
ENSBTAT00000037135	ENSBTAG00000013165	ENPP2	14	83360211	83456674	-	CODING	1.048445	0.014724
ENSBTAT00000008304	ENSBTAG00000006325	NUDCD3	4	77598538	77670087	+	CODING	-0.47009	0.014759
ENSBTAT00000046801	ENSBTAG00000032964	C24H18orf25	24	46409594	46453472	+	CODING	-0.55122	0.014843
ENSBTAT00000034572	ENSBTAG00000024826	TECRL	6	81511554	81653990	-	CODING	0.802917	0.014903
ENSBTAT00000022960	ENSBTAG00000017266	ITGA6	2	24131486	24217715	-	CODING	0.636141	0.014988
ENSBTAT00000026553	ENSBTAG00000019929	ITGAV	2	9651631	9760100	-	CODING	-0.83167	0.014997
ENSBTAT00000005582	ENSBTAG00000004261	SPON2	6	109224627	109228237	-	CODING	1.095519	0.015014
ENSBTAT00000011675	ENSBTAG00000008866	GANC	10	37754445	37817713	+	CODING	-1.44664	0.01503
ENSBTAT00000044703	ENSBTAG00000001790	SAFB2	7	19908341	19936959	+	CODING	0.543162	0.015033
ENSBTAT00000048145	ENSBTAG00000027843	TCEAL8	X	57476264	57477875	-	CODING	0.507641	0.015137
ENSBTAT00000007226	ENSBTAG00000005497	RPRD1A	24	21361884	21427671	+	CODING	-0.73067	0.015166
ENSBTAT00000019134	ENSBTAG00000014388	EIF3I	2	122128559	122136316	-	CODING	0.241461	0.015184
ENSBTAT00000052340	ENSBTAG00000010464	MN1	17	69431677	69478074	-	CODING	-0.91765	0.015225
ENSBTAT00000018278	ENSBTAG00000013755	ITGB5	1	69801844	69899676	-	CODING	0.593946	0.015226
ENSBTAT00000020254	ENSBTAG00000015222	RSRP1	2	128302962	128306994	+	CODING	0.778908	0.015237
ENSBTAT00000057166	ENSBTAG00000040323	SECTM1	19	51104746	51149867	+	CODING	0.99446	0.015259
ENSBTAT00000014770	ENSBTAG00000011121	CLCN4	X	142758776	142827683	-	CODING	-1.32052	0.015352
ENSBTAT00000027348	ENSBTAG00000020527	IDH1	2	96941049	96962496	-	CODING	0.513401	0.015411
ENSBTAT00000015910	ENSBTAG00000011988	KIAA0100	19	20619020	20645017	-	CODING	-0.90106	0.015456
ENSBTAT00000015797	ENSBTAG00000011909	ACVR1	2	39287889	39361498	+	CODING	-0.54867	0.015479

ENSBTAT00000055001	ENSBTAG00000007133	CUL2	13	18592918	18664294	+	CODING	-0.58217	0.015535
ENSBTAT00000019627	ENSBTAG00000014750	EPB41L4A	10	1467376	1761414	+	CODING	1.354388	0.01558
ENSBTAT00000008822	ENSBTAG00000006708	ARIH1	10	19197514	19300694	+	CODING	-0.41063	0.015614
ENSBTAT00000021643	ENSBTAG00000016271	SNRPF	5	60556051	60564297	+	CODING	0.606823	0.015626
ENSBTAT00000002431	ENSBTAG00000001865	SGCG	12	34924193	34954970	-	CODING	-0.39179	0.015685
ENSBTAT00000003567	ENSBTAG00000002749	CRHR1	19	46456088	46483532	+	CODING	-1.76819	0.015726
ENSBTAT00000026215	ENSBTAG00000019669	CD163	5	102245937	102278444	+	CODING	1.381716	0.01574
ENSBTAT00000038128	ENSBTAG00000000053	FILIP1	9	15140841	15249645	-	CODING	-0.63847	0.015743
ENSBTAT00000005881	ENSBTAG00000004484	CEP83	5	24226912	24374720	-	CODING	-0.88049	0.015836
ENSBTAT00000063362	ENSBTAG00000047824	-	16	31136171	31137374	-	CODING	-0.34159	0.015858
ENSBTAT00000046197	ENSBTAG00000000223	PPM1B	11	26402682	26426120	+	CODING	-0.309	0.015904
ENSBTAT00000052073	ENSBTAG00000037726	PLCD1	22	11497086	11518465	-	CODING	1.035005	0.015951
ENSBTAT00000010108	ENSBTAG00000007685	PSMB1	9	105655749	105673470	-	CODING	0.213799	0.015953
ENSBTAT00000036474	ENSBTAG00000018796	RABGGTA	10	20726808	20733197	+	CODING	7.75891	0.016015
ENSBTAT00000005626	ENSBTAG00000021372	42989	6	93528980	93628589	+	CODING	-0.60248	0.016233
ENSBTAT00000061484	ENSBTAG00000002728	ARID1B	9	94882344	95271355	+	CODING	-0.9487	0.01627
ENSBTAT00000063302	ENSBTAG00000048159	TAB3	X	117948890	117980661	+	CODING	-0.93728	0.016322
ENSBTAT00000001071	ENSBTAG00000000810	COPE	7	4224033	4242168	+	CODING	0.489066	0.016367
ENSBTAT00000011251	ENSBTAG00000008535	SOCS7	19	39648073	39672758	+	CODING	-1.13653	0.016406
ENSBTAT00000046011	ENSBTAG00000032436	UBC	17	53142511	53143425	+	CODING	0.099669	0.01642
ENSBTAT00000007571	ENSBTAG00000005757	FUS	25	27523969	27533988	+	CODING	0.263877	0.01644
ENSBTAT00000066246	ENSBTAG00000046218	TIEG2	11	87525614	87536326	-	CODING	-0.39615	0.016449
ENSBTAT00000053498	ENSBTAG00000037457	MBD2	24	54044363	54093578	-	CODING	0.380636	0.016499
ENSBTAT00000044192	ENSBTAG00000031205	RPS4X	7	23580042	23580833	-	CODING	0.413014	0.01656
ENSBTAT00000028757	ENSBTAG00000021580	HNRNPA3	2	19700523	19705981	-	CODING	0.284246	0.016638
ENSBTAT00000019948	ENSBTAG00000014984	RPRD2	3	20254979	20345588	-	CODING	-0.855	0.016675
ENSBTAT00000013682	ENSBTAG00000010362	NOSTRIN	2	27317638	27380506	-	CODING	0.98916	0.016722
ENSBTAT00000003097	ENSBTAG00000002392	ANK2	6	13389038	13529927	-	CODING	-1.25722	0.016739
ENSBTAT00000064553	ENSBTAG00000014132	SNED1	3	120761890	120804404	+	CODING	2.155126	0.016859
ENSBTAT00000027661	ENSBTAG00000020755	SELP	16	38049259	38080860	-	CODING	1.450617	0.016882
ENSBTAT00000035341	ENSBTAG00000025211	ING1	12	89339068	89339790	+	CODING	0.461151	0.016925
ENSBTAT00000056220	ENSBTAG00000007731	-	14	10191721	10264168	-	CODING	-0.96935	0.017002
ENSBTAT00000023529	ENSBTAG00000017690	CARNS1	29	45957524	45964322	+	CODING	-0.87065	0.017067
ENSBTAT00000001374	ENSBTAG00000001036	HIBADH	4	68926598	69034386	+	CODING	-0.2669	0.017073
ENSBTAT00000023664	ENSBTAG00000017798	ERH	10	81521060	81533819	-	CODING	0.517034	0.017108
ENSBTAT00000066224	ENSBTAG00000048160	TIGD5	14	2311790	2313733	-	CODING	-1.23262	0.01719
ENSBTAT00000022238	ENSBTAG00000016728	PLEKHG5	16	47719088	47751042	+	CODING	1.461715	0.017201
ENSBTAT00000039169	ENSBTAG00000027320	KCNB1	13	78194165	78304515	-	CODING	-0.80745	0.017224
ENSBTAT00000006613	ENSBTAG00000005027	GLRX3	26	49742225	49773421	+	CODING	0.328722	0.017285
ENSBTAT00000060327	ENSBTAG00000043335	U6	14	64324526	64324632	-	CODING	-0.09071	0.017366
ENSBTAT00000023221	ENSBTAG00000017468	NUCB2	15	35693278	35715902	-	CODING	0.676019	0.017377
ENSBTAT00000020211	ENSBTAG00000015192	FAM193B	7	40378360	40388704	-	CODING	1.156022	0.017378
ENSBTAT00000043502	ENSBTAG00000047186	NME2	19	36214293	36217603	-	CODING	0.434294	0.017382
ENSBTAT00000035470	ENSBTAG00000011721	MED13	19	11288860	11379955	+	CODING	-0.63816	0.01751
ENSBTAT00000023441	ENSBTAG00000017626	GFPT1	11	67684390	67728959	-	CODING	-0.99873	0.017513
ENSBTAT00000015774	ENSBTAG00000011889	NOC4L	17	46007721	46012689	-	CODING	-0.81405	0.017549
ENSBTAT00000026389	ENSBTAG00000019806	YPEL2	19	10546157	10593841	+	CODING	-0.73959	0.017557
ENSBTAT00000064551	ENSBTAG00000046037	SLC25A5	X	3696978	3701262	+	CODING	1.256501	0.017596
ENSBTAT00000022580	ENSBTAG00000016979	PCYT1A	1	71431933	71484412	-	CODING	-0.7471	0.017653
ENSBTAT00000007299	ENSBTAG00000005550	SOS2	10	43096672	43177625	-	CODING	-0.70944	0.017671
ENSBTAT00000044565	ENSBTAG00000005980	RREB1	23	47900595	47959504	-	CODING	-1.04459	0.017698
ENSBTAT00000035577	ENSBTAG00000008024	UCHL3	12	50948444	50997544	+	CODING	0.321976	0.017745
ENSBTAT00000019149	ENSBTAG00000014401	SORBS3	8	70354893	70384759	+	CODING	0.70106	0.017823
ENSBTAT00000013376	ENSBTAG00000010135	TMEM189	13	78882751	78907460	-	CODING	-0.42697	0.017849
ENSBTAT00000022630	ENSBTAG00000017020	S100G	X	134101039	134104560	-	CODING	1.908554	0.017889
ENSBTAT00000063154	ENSBTAG00000047708	CD99L2	X	33639092	33646559	-	CODING	-0.94433	0.017905
ENSBTAT00000001919	ENSBTAG00000001465	P2RY1	1	115834618	115836268	-	CODING	-0.79723	0.017967
ENSBTAT00000066030	ENSBTAG00000046545	MEF2D	3	14380072	14413740	+	CODING	-0.29713	0.01797
ENSBTAT00000008038	ENSBTAG00000006121	SBK2	18	62435402	62438857	+	CODING	2.988983	0.017972
ENSBTAT00000000519	ENSBTAG00000000405	VKORC1	25	27457370	27459608	-	CODING	0.655626	0.017977
ENSBTAT00000018833	ENSBTAG00000014171	NAPEPLD	4	44665683	44720623	-	CODING	-0.91998	0.018002
ENSBTAT00000050296	ENSBTAG00000010442	PANK1	26	11280207	11335233	-	CODING	0.95502	0.018041
ENSBTAT00000028515	ENSBTAG00000021392	WDR23	10	20855458	20863354	-	CODING	-0.57491	0.018073
ENSBTAT00000011016	ENSBTAG00000008369	RBM8A	3	21553392	21555392	+	CODING	0.425121	0.018085

ENSBTAT00000010926	ENSBTAG00000008300	FN1	2	103881402	103950562	-	CODING	0.687971	0.018142
ENSBTAT00000022809	ENSBTAG00000017157	CCDC110	27	14748843	14760880	-	CODING	3.382692	0.01817
ENSBTAT00000009959	ENSBTAG00000007570	NDUFAF6	14	71606359	71642582	-	CODING	-0.59549	0.01818
ENSBTAT00000019214	ENSBTAG00000014449	-	1	84033060	84033889	+	CODING	1.174619	0.018187
ENSBTAT00000037406	ENSBTAG00000001503	TRIM72	25	27552790	27561099	+	CODING	-0.68016	0.01829
ENSBTAT00000022068	ENSBTAG00000016591	RAB11FIP3	25	404964	464143	+	CODING	-0.72105	0.018323
ENSBTAT00000023784	ENSBTAG00000017896	ST8SIA2	21	14879467	14949980	-	CODING	-1.42684	0.018336
ENSBTAT00000060992	ENSBTAG00000040338	OBSL1	2	108203750	108225840	-	CODING	-0.71152	0.018337
ENSBTAT00000039940	ENSBTAG00000027716	C7H19orf53	15	42332731	42333027	-	CODING	1.023448	0.018353
ENSBTAT00000012872	ENSBTAG00000009761	ACTR2	11	63552997	63591980	+	CODING	0.35753	0.018361
ENSBTAT00000008607	ENSBTAG00000006550	GABARAPL2	18	2907580	2917649	+	CODING	0.258365	0.018369
ENSBTAT00000027966	ENSBTAG00000021000	KIAA2018	1	58790854	58811091	-	CODING	-0.78915	0.018393
ENSBTAT00000019817	ENSBTAG00000014885	MYOM3	2	129388770	129437616	+	CODING	-0.34475	0.018418
ENSBTAT00000065275	ENSBTAG00000000895	TAF9B	X	79269814	79277533	+	CODING	-0.91229	0.018418
ENSBTAT00000029090	ENSBTAG00000014490	DDX39B	23	27565924	27576711	+	CODING	0.273172	0.018432
ENSBTAT00000022718	ENSBTAG00000017082	POR	25	34672093	34726073	+	CODING	0.60305	0.018486
ENSBTAT00000017035	ENSBTAG00000012822	MGC137036	23	15892878	15893213	+	CODING	2.798055	0.018685
ENSBTAT00000026402	ENSBTAG00000019818	CASZ1	16	43786499	43839928	+	CODING	-0.76536	0.018693
ENSBTAT00000025437	ENSBTAG00000019106	EIF1B	22	13214224	13216690	+	CODING	0.368782	0.018745
ENSBTAT00000012436	ENSBTAG00000009451	JDP2	10	87006443	87051824	+	CODING	0.998762	0.018781
ENSBTAT00000003640	ENSBTAG00000045857	FAM195A	25	578685	583777	+	CODING	0.454172	0.018907
ENSBTAT00000020154	ENSBTAG00000015151	FOXN2	11	30534467	30557119	+	CODING	-0.63433	0.018935
ENSBTAT00000005277	ENSBTAG00000004036	GJC1	19	45154679	45186611	-	CODING	1.141611	0.018954
ENSBTAT00000017141	ENSBTAG00000012896	METTL7B	5	57899029	57901211	-	CODING	1.191016	0.018977
ENSBTAT00000005512	ENSBTAG00000004204	GPR107	11	100432297	100484528	+	CODING	-0.98322	0.018978
ENSBTAT00000005445	ENSBTAG00000004159	SIX2	11	27260475	27263699	-	CODING	-1.15225	0.019017
ENSBTAT00000044692	ENSBTAG00000019675	ATXN1	23	40588548	40606204	+	CODING	-1.10574	0.019075
ENSBTAT00000023522	ENSBTAG00000017685	JUP	19	42602486	42626600	-	CODING	0.570219	0.019125
ENSBTAT00000064292	ENSBTAG00000045584	CAV2	4	52230674	52235915	-	CODING	0.597065	0.019132
ENSBTAT00000031441	ENSBTAG00000011998	DUT	10	62284071	62295576	-	CODING	0.436914	0.019187
ENSBTAT00000023485	ENSBTAG00000017655	PALMD	3	43688103	43748131	-	CODING	0.244519	0.019247
ENSBTAT00000046975	ENSBTAG00000012094	SCAMP3	3	15432100	15436418	+	CODING	-0.30734	0.019363
ENSBTAT00000005604	ENSBTAG00000004280	SRPX	X	110531879	110672966	+	CODING	1.611253	0.019367
ENSBTAT00000018869	ENSBTAG00000014191	QSOX1	16	62804447	62845817	+	CODING	0.816722	0.019379
ENSBTAT00000014758	ENSBTAG00000011115	CH25H	26	10976195	10978251	-	CODING	1.540904	0.019392
ENSBTAT00000028751	ENSBTAG00000021577	ZFYVE16	7	82875305	82908670	+	CODING	-0.71414	0.019415
ENSBTAT00000038492	ENSBTAG00000019075	MAP1D	2	24512078	24590687	-	CODING	1.588932	0.019429
ENSBTAT00000005765	ENSBTAG00000004394	PKIB	9	28880110	29001337	-	CODING	1.815962	0.01952
ENSBTAT00000005370	ENSBTAG00000004110	-	X	84590185	84590760	-	CODING	-2.53756	0.019524
ENSBTAT00000006474	ENSBTAG00000004920	COX8A	20	24442440	24442887	+	CODING	0.547667	0.019528
ENSBTAT00000027722	ENSBTAG00000020803	-	X	141098691	141104299	-	CODING	2.07276	0.019533
ENSBTAT00000010674	ENSBTAG00000008114	CD99	X	140188320	140215657	-	CODING	0.352352	0.019578
ENSBTAT00000033827	ENSBTAG00000039851	UBAC1	11	103515902	103535051	-	CODING	-0.25022	0.019581
ENSBTAT00000027160	ENSBTAG00000020376	PAIP1	20	31284382	31311777	+	CODING	-0.45802	0.019638
ENSBTAT00000065813	ENSBTAG00000045523	-	6	35008595	35010340	+	CODING	-0.39801	0.019653
ENSBTAT00000002587	ENSBTAG00000001996	SNRNP70	18	56058668	56073102	+	CODING	0.569798	0.019739
ENSBTAT00000055485	ENSBTAG00000018086	TBC1D10A	17	71459718	71470126	-	CODING	1.389959	0.019797
ENSBTAT00000027899	ENSBTAG00000020947	RNF103	11	48149255	48171946	+	CODING	-0.43198	0.019811
ENSBTAT00000020668	ENSBTAG00000024526	-	27	22570243	22576884	+	CODING	1.063182	0.019844
ENSBTAT00000003283	ENSBTAG00000002531	ARHGAP10	17	10182664	10560361	-	CODING	0.559538	0.019848
ENSBTAT00000005615	ENSBTAG00000004283	PPFIBP1	5	82542192	82609403	-	CODING	-0.52691	0.019878
ENSBTAT00000005080	ENSBTAG00000003895	CYBA	18	13931107	13938075	-	CODING	1.208118	0.019878
ENSBTAT00000020850	ENSBTAG00000015704	TMCO3	12	90698790	90720746	+	CODING	-0.35456	0.019903
ENSBTAT00000019980	ENSBTAG00000015009	LAMTOR4	25	36788650	36791965	-	CODING	0.432725	0.019977
ENSBTAT00000033124	ENSBTAG00000014891	SRSF7	11	21105343	21110797	-	CODING	0.458546	0.020069
ENSBTAT00000021868	ENSBTAG00000016445	YME1L1	13	17986323	18015111	-	CODING	-0.31818	0.020077
ENSBTAT00000045778	ENSBTAG00000011268	TMEM64	14	75870563	75904774	+	CODING	-0.90808	0.020104
ENSBTAT00000017780	ENSBTAG00000013367	CLN1	3	106622151	106638462	+	CODING	0.662747	0.020128
ENSBTAT00000028801	ENSBTAG00000003018	FSTL3	7	44896887	44903223	+	CODING	1.278176	0.020131
ENSBTAT00000061210	ENSBTAG00000021879	VCL	28	30053806	30166317	+	CODING	0.241564	0.020256
ENSBTAT00000021593	ENSBTAG00000016229	KLF9	8	46883356	46907760	-	CODING	-0.32248	0.02027
ENSBTAT00000007465	ENSBTAG00000005681	ME1	9	23401999	23590098	-	CODING	-0.69077	0.020272
ENSBTAT00000039377	ENSBTAG00000027426	-	16	64843773	64844924	-	CODING	0.546154	0.020292
ENSBTAT00000004477	ENSBTAG00000007480	CIRBP	7	45354486	45359172	+	CODING	0.596229	0.020324

ENSBTAT00000034217	ENSBTAG00000024578	-	6	27328572	27329745	-	CODING	0.6306	0.020372
ENSBTAT00000064229	ENSBTAG00000046189	RNASE13	10	26126017	26126478	+	CODING	-1.14874	0.020377
ENSBTAT00000004051	ENSBTAG00000003115	KBTBD2	4	64321838	64324408	+	CODING	-0.33116	0.020381
ENSBTAT00000025789	ENSBTAG00000019358	RNF214	15	28355288	28397142	+	CODING	-0.7204	0.020403
ENSBTAT00000021559	ENSBTAG00000016199	CLINT1	7	71421947	71479026	-	CODING	-0.36588	0.020408
ENSBTAT00000039344	ENSBTAG00000027412	-	13	51930067	51930888	+	CODING	-0.40078	0.020423
ENSBTAT00000043154	ENSBTAG00000006538	PTHLH	5	82246522	82258519	+	CODING	2.991104	0.020445
ENSBTAT00000020880	ENSBTAG00000015731	TXNL1	24	56404067	56446678	-	CODING	0.166345	0.0205
ENSBTAT00000065483	ENSBTAG00000017512	MAPT	19	46617348	46643750	+	CODING	-0.86223	0.020506
ENSBTAT00000023396	ENSBTAG00000017592	AGPAT9	6	100070895	100141603	+	CODING	1.02318	0.020509
ENSBTAT00000031313	ENSBTAG00000023039	-	28	31044242	31057065	-	CODING	-1.16628	0.020585
ENSBTAT00000046599	ENSBTAG00000032829	GHITM	28	39435546	39448963	+	CODING	-0.20262	0.020632
ENSBTAT00000010613	ENSBTAG00000008068	ERLEC1	11	36371087	36395686	+	CODING	-0.59291	0.020636
ENSBTAT00000022946	ENSBTAG00000017265	VBP1	X	38609347	38633977	-	CODING	0.29536	0.020675
ENSBTAT00000035191	ENSBTAG00000008424	ABR	19	22374054	22431563	+	CODING	2.34671	0.020809
ENSBTAT00000012317	ENSBTAG00000009359	-	15	38876122	38876667	-	CODING	-0.10695	0.020875
ENSBTAT00000006917	ENSBTAG00000005257	PEX13	11	43608812	43640302	+	CODING	-0.62606	0.020918
ENSBTAT00000022237	ENSBTAG00000016730	ANP32E	3	20453150	20465219	+	CODING	-0.36762	0.02094
ENSBTAT00000046144	ENSBTAG00000032527	ERCC6	28	44015477	44086510	-	CODING	-1.17124	0.020948
ENSBTAT00000028556	ENSBTAG00000021421	SSR4	X	39925628	39929382	+	CODING	0.371622	0.020963
ENSBTAT00000045795	ENSBTAG00000000671	PARP3	22	49538818	49544236	-	CODING	1.43811	0.021052
ENSBTAT00000000389	ENSBTAG00000000305	LMO4	3	56832933	56846708	-	CODING	0.301859	0.021126
ENSBTAT00000005179	ENSBTAG00000003970	MITD1	11	4290569	4306454	-	CODING	0.952998	0.021147
ENSBTAT00000066239	ENSBTAG00000046959	CGGBP1	1	35655412	35660398	-	CODING	-0.53238	0.021152
ENSBTAT00000015319	ENSBTAG00000011528	SMIM11	1	463572	478996	-	CODING	0.287407	0.021168
ENSBTAT00000040049	ENSBTAG00000014354	FXYD6	15	28926760	28963808	-	CODING	0.612804	0.021169
ENSBTAT00000002375	ENSBTAG00000001814	PLXND1	22	56774467	56824220	+	CODING	0.97678	0.021189
ENSBTAT00000052166	ENSBTAG00000018775	TPX2	13	61831821	61885788	+	CODING	-0.93262	0.021251
ENSBTAT00000057500	ENSBTAG00000039035	HSPA6	3	8027845	8029776	-	CODING	1.593147	0.021331
ENSBTAT00000053511	ENSBTAG00000038283	TMEM243	4	33347225	33368787	+	CODING	0.643165	0.021369
ENSBTAT00000024279	ENSBTAG00000018238	ABTB1	22	60502719	60507367	-	CODING	0.937733	0.021405
ENSBTAT00000013178	ENSBTAG00000015450	CPEB3	26	13613121	13763563	-	CODING	-0.43533	0.02145
ENSBTAT00000011678	ENSBTAG00000010230	CAPN1	29	44064429	44089990	+	CODING	-0.69211	0.021483
ENSBTAT00000003484	ENSBTAG00000002690	BLZF1	16	37838604	37860891	+	CODING	-0.41804	0.021505
ENSBTAT00000013796	ENSBTAG00000010451	PA2G4	5	57532245	57538950	-	CODING	0.315883	0.021528
ENSBTAT00000063184	ENSBTAG00000046547	-	24	43217455	43224463	+	CODING	1.344739	0.021528
ENSBTAT00000029212	ENSBTAG00000021912	DUSP7	22	49379908	49385352	+	CODING	-0.62749	0.021554
ENSBTAT00000012726	ENSBTAG00000009657	MTMR1	X	33558564	33634304	+	CODING	-0.70342	0.021644
ENSBTAT00000035885	ENSBTAG00000021134	DPP9	7	20648283	20678569	+	CODING	-0.60268	0.021653
ENSBTAT00000019910	ENSBTAG00000014328	-	18	61608937	61623944	-	CODING	1.073447	0.021669
ENSBTAT00000003881	ENSBTAG00000002980	GPT2	18	15370927	15405215	+	CODING	-0.56629	0.021717
ENSBTAT00000019505	ENSBTAG00000014649	CPT2	3	93601359	93625434	-	CODING	0.561072	0.021731
ENSBTAT00000032362	ENSBTAG00000005604	POPDC2	1	65002248	65014687	-	CODING	-1.04925	0.021822
ENSBTAT00000064631	ENSBTAG00000004093	TUBB2B	23	50354843	50357631	+	CODING	2.191259	0.021861
ENSBTAT00000021451	ENSBTAG00000019386	BOLA-NC	23	28355361	28358332	-	CODING	0.51003	0.02191
ENSBTAT00000006112	ENSBTAG00000039307	-	15	6750617	6817541	-	CODING	-0.69789	0.021931
ENSBTAT00000000631	ENSBTAG00000000494	PDE4D	20	20268721	20321935	+	CODING	-0.45921	0.021937
ENSBTAT00000018177	ENSBTAG00000013678	OSGIN2	14	76149588	76178108	-	CODING	-0.64847	0.021973
ENSBTAT00000004994	ENSBTAG00000003832	MFAP2	2	136187693	136192151	+	CODING	1.318798	0.021989
ENSBTAT00000000092	ENSBTAG00000000081	COL4A3BP	10	6725117	6853171	-	CODING	-0.54103	0.022004
ENSBTAT00000065332	ENSBTAG00000020121	RWDD2B	1	6529548	6533387	+	CODING	-0.7785	0.022021
ENSBTAT00000016579	ENSBTAG00000012496	MTMR11	3	20718768	20726741	+	CODING	1.37522	0.022121
ENSBTAT00000023950	ENSBTAG00000017999	TNRC6A	25	22701134	22744636	+	CODING	-0.75325	0.022143
ENSBTAT00000017150	ENSBTAG00000012902	ARL8B	22	21327751	21374042	-	CODING	-0.3743	0.022191
ENSBTAT00000042737	ENSBTAG00000013988	BID2	5	109639251	109656755	-	CODING	1.333309	0.022226
ENSBTAT00000065868	ENSBTAG00000018227	SLC4A7	22	1746286	1877841	-	CODING	0.994847	0.022231
ENSBTAT00000027024	ENSBTAG00000025942	HPGD	8	6642818	6680546	-	CODING	1.250921	0.022257
ENSBTAT00000001568	ENSBTAG00000001182	CDC10	4	61611420	61710127	-	CODING	1.386343	0.022262
ENSBTAT00000028045	ENSBTAG00000021059	TNMD	X	50951671	50973156	-	CODING	1.958415	0.022273
ENSBTAT00000047985	ENSBTAG00000021064	-	21	15998170	16090486	+	CODING	-1.0379	0.022324
ENSBTAT00000003493	ENSBTAG00000002698	UBE3B	17	65905227	65944588	-	CODING	-0.81533	0.022362
ENSBTAT00000024223	ENSBTAG00000018198	TLR9	22	49229610	49233939	+	CODING	2.780227	0.022368
ENSBTAT00000036093	ENSBTAG00000025595	-	22	7111728	7112287	+	CODING	0.771838	0.022381
ENSBTAT00000004052	ENSBTAG00000003116	MTMR6	12	34177965	34197920	+	CODING	-0.61771	0.022451

ENSBTAT00000010569	ENSBTAG00000008034	LMAN2	7	40185729	40206355	-	CODING	0.369704	0.022557
ENSBTAT00000002179	ENSBTAG00000001665	COG8	18	36600499	36607395	-	CODING	-0.61007	0.02256
ENSBTAT00000063575	ENSBTAG00000045633	AGTR1	1	120403009	120404088	-	CODING	0.734601	0.022686
ENSBTAT00000020239	ENSBTAG00000015212	IFNAR2	1	1593295	1627137	-	CODING	1.053986	0.022772
ENSBTAT00000011200	ENSBTAG00000008497	RGS14	7	40214182	40227956	+	CODING	-1.09918	0.022775
ENSBTAT00000019596	ENSBTAG00000014724	EIF4A2	1	81057779	81064090	-	CODING	-0.1377	0.022775
ENSBTAT00000027897	ENSBTAG00000020944	PIEZO1	18	13984761	14002517	-	CODING	0.965076	0.022797
ENSBTAT00000009444	ENSBTAG00000007177	EMP2	25	9330475	9359612	-	CODING	-0.39653	0.022804
ENSBTAT00000028721	ENSBTAG00000021556	RANBP9	23	42736705	42795080	+	CODING	-0.42502	0.022846
ENSBTAT00000045780	ENSBTAG00000008192	-	7	16982664	16990838	+	CODING	0.790153	0.022862
ENSBTAT00000032789	ENSBTAG00000023867	RC3H2	11	94010161	94058169	-	CODING	-1.01613	0.022887
ENSBTAT00000014061	ENSBTAG00000010637	ARSG	19	62368464	62401915	+	CODING	-0.85488	0.022909
ENSBTAT00000002398	ENSBTAG00000001835	GJA1	9	30127786	30140793	-	CODING	0.852881	0.022982
ENSBTAT00000005180	ENSBTAG00000003956	SGCA	19	37113477	37122359	-	CODING	-0.2204	0.022989
ENSBTAT00000063370	ENSBTAG00000046841	IRF2BP2	28	7444901	7446967	-	CODING	-0.36342	0.022989
ENSBTAT00000054636	ENSBTAG00000018986	ACSL4	X	62476952	62508324	-	CODING	-0.62092	0.02304
ENSBTAT00000002976	ENSBTAG00000002306	UTP11L	3	108529496	108546298	-	CODING	-0.38164	0.023136
ENSBTAT00000018762	ENSBTAG00000014105	SRPR	29	29986076	29991236	-	CODING	-0.29613	0.02315
ENSBTAT00000020023	ENSBTAG00000015043	MFNG	5	76266277	76282792	-	CODING	0.800314	0.023232
ENSBTAT00000027175	ENSBTAG00000020387	MARF1	25	14138542	14171367	-	CODING	-0.821	0.023235
ENSBTAT00000020032	ENSBTAG00000015050	HCN2	7	44831440	44850882	+	CODING	-1.26031	0.023293
ENSBTAT00000016194	ENSBTAG00000012208	-	23	27689369	27692407	+	CODING	0.888069	0.023424
ENSBTAT00000012560	ENSBTAG00000009547	ZDHHC4	25	38953354	38961974	+	CODING	-0.23698	0.02349
ENSBTAT00000030222	ENSBTAG00000033884	TMA7	5	11308520	11308714	+	CODING	-3.28554	0.023552
ENSBTAT00000016139	ENSBTAG00000012168	RANBP6	8	38922907	38926224	+	CODING	-0.87673	0.023572
ENSBTAT00000018196	ENSBTAG00000013688	PGF	10	86595706	86608816	-	CODING	1.720949	0.023638
ENSBTAT00000063789	ENSBTAG00000026585	TCEB3	2	129738383	129752889	-	CODING	-0.67047	0.023639
ENSBTAT00000028862	ENSBTAG00000021658	GRASP	5	28019961	28027764	-	CODING	0.950415	0.023668
ENSBTAT00000018997	ENSBTAG00000014297	MOV10	3	30776860	30804255	-	CODING	1.302787	0.023684
ENSBTAT00000009630	ENSBTAG00000007321	SREK1	20	13393479	13436297	-	CODING	0.71899	0.023709
ENSBTAT00000062037	ENSBTAG00000044604	snoU89	10	36862418	36862608	+	CODING	-3.28315	0.023712
ENSBTAT00000061219	ENSBTAG00000043975	MYCT1	9	90836602	90852725	+	CODING	0.500838	0.023764
ENSBTAT00000018085	ENSBTAG00000013600	PSMG1	1	140726628	140739236	-	CODING	0.398089	0.023768
ENSBTAT00000026361	ENSBTAG00000019784	PPP2R5E	10	75953664	76109267	-	CODING	-0.59828	0.02387
ENSBTAT00000007668	ENSBTAG00000005830	STRN4	18	54263458	54287817	-	CODING	-0.62665	0.023927
ENSBTAT00000020355	ENSBTAG00000015312	LTBR	5	104346662	104354420	-	CODING	0.757257	0.023934
ENSBTAT00000020102	ENSBTAG00000015107	SLC16A1	3	30533845	30563298	+	CODING	0.342566	0.023986
ENSBTAT00000024367	ENSBTAG00000018312	-	1	104146631	104147374	+	CODING	0.419823	0.023996
ENSBTAT00000016684	ENSBTAG00000012565	PCNX	10	82878051	83056528	+	CODING	-0.81345	0.024052
ENSBTAT00000026674	ENSBTAG00000020026	-	3	16358535	16366943	+	CODING	-0.30766	0.024094
ENSBTAT00000013737	ENSBTAG00000010402	MYH9	5	75094866	75179909	-	CODING	0.350152	0.024133
ENSBTAT00000005280	ENSBTAG00000004038	ABAT	25	7647412	7689263	+	CODING	-0.83451	0.024134
ENSBTAT00000021199	ENSBTAG00000015942	DNAJA4	21	31177694	31192961	+	CODING	-0.33474	0.024205
ENSBTAT00000003274	ENSBTAG00000002520	CUGBP1	15	78490888	78570865	-	CODING	-0.51685	0.024214
ENSBTAT00000053533	ENSBTAG00000004136	NFE2L3	4	70211380	70214288	-	CODING	-1.11148	0.024239
ENSBTAT00000018159	ENSBTAG00000013662	COL8A1	1	43541936	43717619	+	CODING	1.142057	0.024313
ENSBTAT00000044218	ENSBTAG00000031231	IRF1	7	23235653	23243697	+	CODING	0.96967	0.024318
ENSBTAT00000044780	ENSBTAG00000009048	EFHD2	16	53306776	53324889	-	CODING	0.931676	0.024337
ENSBTAT00000061513	ENSBTAG00000019625	EHHADH	1	82467817	82521151	+	CODING	-1.22637	0.024415
ENSBTAT00000061585	ENSBTAG00000015438	RRBP1	13	38289297	38317466	-	CODING	0.758718	0.024457
ENSBTAT00000023319	ENSBTAG00000017542	PPARD	23	9340955	9353750	+	CODING	0.840486	0.024494
ENSBTAT00000061378	ENSBTAG00000044048	LMBR1L	5	30879874	30891690	+	CODING	-0.9936	0.024575
ENSBTAT00000018525	ENSBTAG00000013943	ZNF703	27	32683065	32685824	+	CODING	-0.70379	0.024582
ENSBTAT00000036738	ENSBTAG00000025931	NEU3	15	55029090	55043623	+	CODING	-0.77368	0.024594
ENSBTAT00000008486	ENSBTAG00000006471	OSBPL11	1	70573620	70664027	-	CODING	0.523248	0.024655
ENSBTAT00000011923	ENSBTAG00000009050	ADIPOR2	5	108705365	108724898	+	CODING	0.268831	0.024798
ENSBTAT00000027246	ENSBTAG00000020446	THOP1	7	22128058	22141914	-	CODING	0.515488	0.024806
ENSBTAT00000055853	ENSBTAG00000046308	-	4	67781717	68200912	-	CODING	1.649867	0.024819
ENSBTAT00000002384	ENSBTAG00000001823	STC2	20	4908292	4918945	-	CODING	2.879567	0.024867
ENSBTAT00000021226	ENSBTAG00000015963	MGC127919	8	37291856	37292741	-	CODING	0.777257	0.024873
ENSBTAT00000043219	ENSBTAG00000030593	RFTN1	1	155176264	155184715	-	CODING	1.302809	0.024882
ENSBTAT00000030183	ENSBTAG00000015368	42982	19	9697827	9707120	-	CODING	0.708435	0.024955
ENSBTAT00000017658	ENSBTAG00000024387	PNPLA8	4	49867702	49907595	-	CODING	-0.64848	0.025025
ENSBTAT00000026782	ENSBTAG00000020105	-	8	76468680	76484933	-	CODING	0.31481	0.025083

ENSBTAT00000000943	ENSBTAG00000000705	COX7A2L	11	24824245	24836596	-	CODING	0.400826	0.025345
ENSBTAT00000064407	ENSBTAG00000002977	ECE1	2	132005289	132058681	+	CODING	0.615313	0.025347
ENSBTAT00000047480	ENSBTAG00000006970	OLA1	2	22564518	22590410	+	CODING	-1.55426	0.025351
ENSBTAT00000005059	ENSBTAG00000003876	MCU	28	28868083	29061027	+	CODING	-0.34037	0.025432
ENSBTAT00000005438	ENSBTAG00000004154	SKIL	1	97824479	97843625	-	CODING	-0.69361	0.025452
ENSBTAT00000024299	ENSBTAG00000018258	SRSF2	19	55666050	55669890	+	CODING	0.338023	0.025476
ENSBTAT00000010440	ENSBTAG00000007942	PAPD5	18	18718590	18730185	+	CODING	-0.64563	0.025483
ENSBTAT00000064438	ENSBTAG00000045717	ADO	28	19004887	19005699	+	CODING	-1.10967	0.025519
ENSBTAT00000029525	ENSBTAG00000011930	DDX54	17	63448841	63466323	-	CODING	-0.52345	0.025559
ENSBTAT00000002344	ENSBTAG00000007875	SAFB	7	19846208	19876661	-	CODING	0.437782	0.025635
ENSBTAT00000003483	ENSBTAG00000002689	NME7	16	37768204	37838172	-	CODING	-2.93257	0.025672
ENSBTAT00000061457	ENSBTAG00000012582	IARS2	16	24285717	24329372	+	CODING	-0.2722	0.025701
ENSBTAT00000009939	ENSBTAG00000007554	IFI6	2	126246561	126250182	+	CODING	0.928681	0.025765
ENSBTAT00000016518	ENSBTAG00000012448	CCDC91	5	81582317	81992463	-	CODING	-0.48394	0.025839
ENSBTAT00000026236	ENSBTAG00000019682	PRRC2A	23	27475781	27488753	-	CODING	-0.52471	0.025891
ENSBTAT00000065711	ENSBTAG00000009830	PLEKHB1	15	53907206	53922643	+	CODING	1.271856	0.025928
ENSBTAT00000010745	ENSBTAG00000008173	TRIM13	12	19543053	19556013	+	CODING	-0.71261	0.026011
ENSBTAT00000020790	ENSBTAG00000015654	PON1	4	12389567	12423459	-	CODING	-2.91465	0.026148
ENSBTAT00000021414	ENSBTAG00000016091	KLHL25	21	16946194	16960419	-	CODING	-1.03567	0.026213
ENSBTAT00000002169	ENSBTAG00000001652	SLCO3A1	21	15205638	15584694	-	CODING	-1.342	0.026225
ENSBTAT00000001770	ENSBTAG00000001344	METTL21E	12	83099334	83115415	+	CODING	0.573074	0.026378
ENSBTAT00000010797	ENSBTAG00000008213	ATMIN	18	7755291	7768469	+	CODING	-0.56656	0.026547
ENSBTAT00000003165	ENSBTAG00000002428	PPA2	6	20984354	21082553	+	CODING	0.445262	0.02663
ENSBTAT00000016457	ENSBTAG00000046688	-	7	34615435	34616823	-	CODING	1.822627	0.02665
ENSBTAT00000064297	ENSBTAG00000048308	ICMT	16	47960246	47967797	-	CODING	-0.81133	0.026658
ENSBTAT00000011166	ENSBTAG00000008480	CTBP1	6	109256152	109271813	-	CODING	-0.4715	0.02667
ENSBTAT00000015866	ENSBTAG00000011954	SEC11C	24	58659025	58670236	+	CODING	0.415014	0.026688
ENSBTAT00000022469	ENSBTAG00000040494	UBL5	2	23299147	23299368	-	CODING	0.519131	0.026694
ENSBTAT00000035237	ENSBTAG00000013392	PLD2	19	27177560	27189861	-	CODING	1.434957	0.026711
ENSBTAT00000002088	ENSBTAG00000001595	MT1E	18	24117441	24118787	-	CODING	3.454414	0.026714
ENSBTAT00000004772	ENSBTAG00000031564	GNL3L	X	97432890	97461194	+	CODING	0.411906	0.026741
ENSBTAT00000018722	ENSBTAG00000014083	AMIGO1	3	34054795	34060289	+	CODING	-0.86688	0.026743
ENSBTAT00000012414	ENSBTAG00000009434	PAQR6	3	14598347	14602877	+	CODING	0.846195	0.026764
ENSBTAT00000023499	ENSBTAG00000017656	SIAT6	3	102718271	102744545	-	CODING	-0.23184	0.02677
ENSBTAT00000023772	ENSBTAG00000017885	FAM173B	20	63111708	63127170	+	CODING	-0.75568	0.02677
ENSBTAT00000002055	ENSBTAG00000001575	-	11	29424996	29432052	-	CODING	0.134023	0.026784
ENSBTAT00000023628	ENSBTAG00000017770	-	4	103732131	103787511	+	CODING	-0.43553	0.026821
ENSBTAT00000016063	ENSBTAG00000012107	SLC25A28	26	20466104	20476402	-	CODING	0.516928	0.026821
ENSBTAT00000025911	ENSBTAG00000019451	TAPT1	6	116194013	116240769	-	CODING	-0.59562	0.026834
ENSBTAT00000030590	ENSBTAG00000022580	FAM212B	3	31719424	31733085	+	CODING	1.161024	0.026855
ENSBTAT00000012512	ENSBTAG00000009509	NDUFA6	5	113575149	113586140	-	CODING	0.201145	0.026868
ENSBTAT00000056087	ENSBTAG00000040490	CCDC3	13	11543628	11636251	+	CODING	0.735468	0.026875
ENSBTAT00000032386	ENSBTAG00000015010	TBC1D23	1	44199315	44254410	+	CODING	-0.4829	0.026937
ENSBTAT00000030067	ENSBTAG00000022255	AR	X	88410764	88621166	-	CODING	-1.79239	0.026956
ENSBTAT00000027838	ENSBTAG00000020895	LOXL4	26	19201799	19214350	-	CODING	1.628333	0.026987
ENSBTAT00000024060	ENSBTAG00000018073	TSPO	5	114596006	114608640	+	CODING	0.948296	0.027052
ENSBTAT00000022140	ENSBTAG00000016644	AQPEP	10	4789066	4866585	+	CODING	0.998895	0.027076
ENSBTAT00000004070	ENSBTAG00000003130	CHRNA3	21	31500106	31512927	-	CODING	-1.55873	0.027078
ENSBTAT00000063644	ENSBTAG00000002938	CPED1	4	86250173	86572861	+	CODING	-0.6353	0.027079
ENSBTAT00000061510	ENSBTAG00000044062	RBMS3	22	3899285	4695113	+	CODING	1.402973	0.027161
ENSBTAT00000006074	ENSBTAG00000004630	COMP	7	4354380	4361950	+	CODING	1.99196	0.027231
ENSBTAT00000015904	ENSBTAG00000011987	C10H14ORF1	10	87913060	87921648	-	CODING	-1.40018	0.027247
ENSBTAT00000004959	ENSBTAG00000003806	ECM1	3	20228528	20233933	-	CODING	0.894819	0.027251
ENSBTAT00000063873	ENSBTAG00000046160	SMIM13	23	45020474	45041768	-	CODING	-0.73409	0.027268
ENSBTAT00000024402	ENSBTAG00000018339	ATP5G1	19	38253561	38255681	-	CODING	0.218771	0.027365
ENSBTAT00000040292	ENSBTAG00000027937	PRNP	13	47400413	47418507	+	CODING	-0.36515	0.027393
ENSBTAT00000014818	ENSBTAG00000011156	ATP8A1	6	62893173	63128455	-	CODING	-1.06483	0.027471
ENSBTAT00000024009	ENSBTAG00000018036	CNOT7	27	19052444	19067489	+	CODING	-0.31818	0.027544
ENSBTAT00000002532	ENSBTAG00000001948	VTI1B	10	80113761	80131964	-	CODING	0.362934	0.027548
ENSBTAT00000000841	ENSBTAG00000000639	APRT	18	14057703	14060472	-	CODING	0.56256	0.027606
ENSBTAT00000016718	ENSBTAG00000012595	HRSP12	14	68412747	68420944	+	CODING	0.663305	0.027614
ENSBTAT00000002998	ENSBTAG00000002323	USP28	15	24669574	24733111	-	CODING	-0.33459	0.02764
ENSBTAT00000000301	ENSBTAG00000000241	STAU1	13	77962041	78003899	-	CODING	-0.45566	0.027687
ENSBTAT00000009806	ENSBTAG00000007455	DNASE1L1	X	40368988	40371659	-	CODING	-0.44404	0.027687

ENSBTAT00000019445	ENSBTAG00000011400	DBN1	7	40312121	40325975	-	CODING	0.705268	0.027746
ENSBTAT00000045888	ENSBTAG00000032347	-	22	48146336	48146712	+	CODING	-0.27551	0.027768
ENSBTAT00000020765	ENSBTAG00000015636	C7H19orf66	7	15876346	15880691	+	CODING	1.258319	0.027811
ENSBTAT00000061530	ENSBTAG00000038584	OLFM1	11	106675880	106712955	-	CODING	-0.92192	0.027818
ENSBTAT00000012804	ENSBTAG00000009705	SERPINF1	19	23422880	23430667	+	CODING	0.733255	0.027819
ENSBTAT00000001494	ENSBTAG00000001123	SNRNP48	23	47647768	47663909	-	CODING	0.737532	0.027819
ENSBTAT00000007925	ENSBTAG00000006033	NDUFA1	X	4068880	4072037	+	CODING	-0.16901	0.027844
ENSBTAT00000004034	ENSBTAG00000003100	SMTN	17	72041477	72064312	+	CODING	0.654626	0.027859
ENSBTAT00000007327	ENSBTAG00000005577	RNASEH2C	29	44552309	44553484	-	CODING	0.362905	0.027875
ENSBTAT00000008300	ENSBTAG00000006328	RBM6	22	50817696	50892474	-	CODING	0.613117	0.02788
ENSBTAT00000010389	ENSBTAG00000007900	FIS1	25	36034989	36038644	+	CODING	0.181551	0.027982
ENSBTAT00000024621	ENSBTAG00000018498	PQBP1	X	92092355	92098095	+	CODING	0.448397	0.027984
ENSBTAT00000000316	ENSBTAG00000000252	POLE3	8	104356462	104359086	-	CODING	-0.30455	0.028048
ENSBTAT00000021534	ENSBTAG00000016177	FGL1	27	18454362	18472436	+	CODING	0.720822	0.02805
ENSBTAT00000022254	ENSBTAG00000016739	RASAL1	17	63397791	63429841	-	CODING	1.512243	0.028119
ENSBTAT00000009098	ENSBTAG00000006927	ABCF1	23	28199129	28211340	-	CODING	-0.41536	0.028178
ENSBTAT00000065432	ENSBTAG00000047598	-	8	60381930	60386110	+	CODING	0.601657	0.028179
ENSBTAT00000064382	ENSBTAG00000046783	-	2	94202055	94202354	+	CODING	1.143065	0.028183
ENSBTAT00000000651	ENSBTAG00000000510	ATG101	5	27962235	27968039	-	CODING	0.31898	0.028208
ENSBTAT00000023111	ENSBTAG00000017380	MYO18A	19	20994380	21082308	-	CODING	-0.2933	0.02825
ENSBTAT00000000179	ENSBTAG00000000156	LGALSL	11	62812719	62817829	+	CODING	-0.42541	0.028259
ENSBTAT00000027443	ENSBTAG00000001839	OCIAD2	6	69175254	69187082	-	CODING	1.001426	0.028274
ENSBTAT00000057334	ENSBTAG00000005710	NCAM1	15	24093189	24165578	+	CODING	-1.98923	0.028397
ENSBTAT00000011978	ENSBTAG00000009086	LOXL1	21	35057678	35082119	-	CODING	1.112811	0.028438
ENSBTAT00000021198	ENSBTAG00000015938	ARAP3	7	54377030	54453900	-	CODING	1.071699	0.028446
ENSBTAT00000008884	ENSBTAG00000006755	SMAP	15	36224848	36251371	-	CODING	-0.31652	0.028451
ENSBTAT00000015317	ENSBTAG00000011527	NT5C	19	56955613	56957740	+	CODING	0.934906	0.028474
ENSBTAT00000043046	ENSBTAG00000030503	H2AFJ	5	95568130	95568806	-	CODING	0.641737	0.028537
ENSBTAT00000030194	ENSBTAG00000011190	FLNA	X	40310757	40332714	-	CODING	0.39056	0.028607
ENSBTAT00000010899	ENSBTAG00000008283	FLI1	29	32664912	32725664	+	CODING	0.810199	0.028623
ENSBTAT00000000738	ENSBTAG00000000565	RFX7	10	54281616	54422091	+	CODING	-0.72175	0.028649
ENSBTAT00000026332	ENSBTAG00000019759	IDE	26	13920375	14012061	-	CODING	-0.50522	0.028686
ENSBTAT00000061119	ENSBTAG00000009702	MYHC-FETAL	19	30031040	30059203	-	CODING	-1.94416	0.028711
ENSBTAT00000016953	ENSBTAG00000012757	GCNT1	8	52848304	52850731	+	CODING	0.549089	0.028863
ENSBTAT00000028958	ENSBTAG00000021724	PDHB	22	43461214	43466626	+	CODING	-0.1792	0.028995
ENSBTAT00000000498	ENSBTAG00000000387	NICN1	22	51262430	51266667	-	CODING	0.80039	0.029061
ENSBTAT00000061468	ENSBTAG00000010529	FZD6	14	63358656	63392777	-	CODING	0.683963	0.029152
ENSBTAT00000001917	ENSBTAG00000001463	TNKS	27	24632930	24789416	+	CODING	-0.93544	0.029207
ENSBTAT00000024516	ENSBTAG00000018426	MRRF	11	93149379	93158576	-	CODING	0.569359	0.029214
ENSBTAT00000008621	ENSBTAG00000006564	PSMA1	15	38745761	38758646	+	CODING	0.224795	0.029395
ENSBTAT00000009985	ENSBTAG00000007591	CHUK	26	20966010	21008277	-	CODING	-0.55672	0.029491
ENSBTAT00000009990	ENSBTAG00000007595	BROX	16	26908537	26927548	+	CODING	-0.62134	0.029493
ENSBTAT00000061300	ENSBTAG00000001021	CYP1A1	21	34342808	34345682	+	CODING	1.117318	0.029494
ENSBTAT00000031877	ENSBTAG00000021799	RCN3	18	56422333	56430760	+	CODING	0.904047	0.02954
ENSBTAT00000010907	ENSBTAG00000008289	ZBTB4	19	27769639	27773345	-	CODING	-0.87733	0.029744
ENSBTAT00000027907	ENSBTAG00000020954	TRIM7	7	41718720	41731788	-	CODING	-0.47056	0.029789
ENSBTAT00000009020	ENSBTAG00000040151	GCH1	10	67576390	67631089	-	CODING	1.091809	0.029791
ENSBTAT00000012952	ENSBTAG00000009824	COMMD6	17	29777299	29777556	+	CODING	-1.21888	0.029832
ENSBTAT00000008382	ENSBTAG00000006391	NDUFAB1	25	21498279	21509168	-	CODING	0.161917	0.029899
ENSBTAT00000047019	ENSBTAG00000033107	OSMR	20	35541619	35588186	-	CODING	1.494917	0.03001
ENSBTAT00000023029	ENSBTAG00000017321	SUMO3	1	145031789	145041320	-	CODING	-0.34463	0.030201
ENSBTAT00000056481	ENSBTAG00000005497	RPRD1A	24	21361884	21398835	+	CODING	1.028196	0.030207
ENSBTAT00000034200	ENSBTAG00000011785	WDR26	16	28258333	28291746	-	CODING	-0.28558	0.030217
ENSBTAT00000029555	ENSBTAG00000001332	MYO1C	19	23168435	23191280	-	CODING	0.729256	0.030271
ENSBTAT00000011612	ENSBTAG00000008817	LAMA4	9	38644310	38810902	+	CODING	0.661066	0.030288
ENSBTAT00000024095	ENSBTAG00000018105	ACVR2B	22	11864297	11894086	+	CODING	-1.85838	0.030397
ENSBTAT00000042831	ENSBTAG00000030333	-	19	43514251	43544945	+	CODING	0.511348	0.030475
ENSBTAT00000018329	ENSBTAG00000015996	GPC1	3	120383561	120476582	+	CODING	-0.52434	0.030577
ENSBTAT00000027190	ENSBTAG00000020403	ZMYM2	12	36391824	36453989	-	CODING	-0.55197	0.030621
ENSBTAT00000024024	ENSBTAG00000018046	B3GNT2	11	60654634	60687730	+	CODING	0.736726	0.030664
ENSBTAT00000014865	ENSBTAG00000011193	C1QC	2	130783987	130788357	-	CODING	1.224153	0.030676
ENSBTAT00000002650	ENSBTAG00000002045	PSMD13	11	107246518	107254957	-	CODING	-0.34788	0.030741
ENSBTAT00000031869	ENSBTAG00000006633	IRF3	18	56517723	56523302	-	CODING	0.945963	0.030883
ENSBTAT00000024709	ENSBTAG00000018566	SFRP5	26	18782564	18787376	-	CODING	0.883732	0.030981

ENSBTAT00000006954	ENSBTAG00000005285	MRPS33	4	105101880	105110529	-	CODING	0.235891	0.031006
ENSBTAT00000027054	ENSBTAG00000020299	OPN1LW	X	40234826	40247095	+	CODING	3.258796	0.031071
ENSBTAT00000012110	ENSBTAG00000009191	TUSC3	27	20419472	20636849	-	CODING	1.289863	0.031127
ENSBTAT00000052503	ENSBTAG00000038375	STOM	8	112646069	112680926	-	CODING	0.626807	0.031243
ENSBTAT00000010692	ENSBTAG00000008132	SOX13	16	1716803	1728817	+	CODING	0.981887	0.031339
ENSBTAT00000062294	ENSBTAG00000044861	SCARNA6	3	113598483	113598756	+	CODING	2.860814	0.031356
ENSBTAT00000034949	ENSBTAG00000025028	MZT1	12	47768049	47781910	-	CODING	-0.70697	0.031444
ENSBTAT00000019983	ENSBTAG00000015007	NCOA1	11	74511607	74590924	-	CODING	-0.68594	0.031453
ENSBTAT00000047251	ENSBTAG00000008642	POLR1D	12	32554786	32556401	-	CODING	0.448931	0.031519
ENSBTAT00000064667	ENSBTAG00000005932	FAM184B	6	38614370	38672306	-	CODING	-0.95942	0.031543
ENSBTAT00000024448	ENSBTAG00000018373	DPYSL2	8	75089346	75168436	+	CODING	0.426186	0.031613
ENSBTAT00000054915	ENSBTAG00000040167	-	10	92889961	92890752	-	CODING	0.537785	0.031659
ENSBTAT00000049723	ENSBTAG00000035230	TOR1AIP2	16	62493958	62528817	-	CODING	-0.83242	0.031702
ENSBTAT00000022642	ENSBTAG00000017028	USO1	6	92297159	92378158	+	CODING	-0.32151	0.031755
ENSBTAT00000002554	ENSBTAG00000001968	TTC14	1	87007533	87017223	-	CODING	0.611364	0.031781
ENSBTAT00000014575	ENSBTAG00000010981	CIB2	21	31017716	31023825	+	CODING	-0.70816	0.031875
ENSBTAT00000015483	ENSBTAG00000011659	PIGZ	1	72024795	72028847	-	CODING	-0.94374	0.032001
ENSBTAT00000043256	ENSBTAG00000030608	SWI5	11	98895393	98902043	+	CODING	0.602726	0.032002
ENSBTAT00000047798	ENSBTAG00000017189	POLE4	11	9683454	9694986	-	CODING	0.932822	0.032022
ENSBTAT00000012005	ENSBTAG00000009109	CASD1	4	11735528	11787909	+	CODING	-0.63267	0.032143
ENSBTAT00000010178	ENSBTAG00000007740	BMK	15	77249974	77251941	+	CODING	1.538074	0.032166
ENSBTAT00000022196	ENSBTAG00000016694	KIAA0319L	3	110829674	110889859	+	CODING	-0.90182	0.03232
ENSBTAT00000024015	ENSBTAG00000018040	PSMB10	18	35540357	35542906	-	CODING	0.674392	0.032328
ENSBTAT00000006774	ENSBTAG00000005138	BCL9L	15	30023047	30033583	-	CODING	-0.75778	0.03233
ENSBTAT00000036123	ENSBTAG00000013362	DNM2	7	16465942	16523297	+	CODING	-0.43492	0.032335
ENSBTAT00000013974	ENSBTAG00000010568	ZBED5	15	42381063	42386360	+	CODING	0.597874	0.032503
ENSBTAT00000006696	ENSBTAG00000005082	MED21	5	83186276	83196046	-	CODING	0.339729	0.032527
ENSBTAT00000063162	ENSBTAG00000002688	ATP1B1	16	37568416	37592799	+	CODING	0.19773	0.032539
ENSBTAT00000038056	ENSBTAG00000012946	HSF2	9	29122564	29161606	-	CODING	0.478698	0.032587
ENSBTAT00000014308	ENSBTAG00000010801	CMBL	20	63065548	63090743	+	CODING	-0.24592	0.032729
ENSBTAT00000010644	ENSBTAG00000008091	SELENBP1	3	19484700	19493477	+	CODING	0.940393	0.032762
ENSBTAT00000003800	ENSBTAG00000002924	DUPD1	28	30993339	31024892	-	CODING	-0.49279	0.03281
ENSBTAT00000000500	ENSBTAG00000000386	-	19	28422865	28423634	-	CODING	0.272337	0.032815
ENSBTAT00000019573	ENSBTAG00000014707	ISG15	16	52714627	52715665	-	CODING	1.442507	0.032827
ENSBTAT00000055368	ENSBTAG00000020796	UBE2D1	26	663449	699996	-	CODING	-0.28372	0.032848
ENSBTAT00000014406	ENSBTAG00000039962	DCTPP1	25	26851888	26855546	-	CODING	0.776385	0.032928
ENSBTAT00000018702	ENSBTAG00000014074	SNX13	4	26353998	26448522	-	CODING	-0.7636	0.033054
ENSBTAT00000025681	ENSBTAG00000019285	FBXL17	7	109976942	110095864	-	CODING	-0.47363	0.033055
ENSBTAT00000020215	ENSBTAG00000015193	BOLA3	11	10489732	10497683	+	CODING	0.449747	0.033092
ENSBTAT00000006918	ENSBTAG00000005259	UCP3	15	54213566	54224051	-	CODING	-0.11054	0.033105
ENSBTAT00000022210	ENSBTAG00000016708	CLTC	19	10848574	10889456	+	CODING	-0.34151	0.033239
ENSBTAT00000014785	ENSBTAG00000011134	USE1	7	5800191	5803107	-	CODING	0.465786	0.033277
ENSBTAT00000024512	ENSBTAG00000018424	CXCR7	3	116650278	116662501	+	CODING	0.464045	0.033348
ENSBTAT00000020060	ENSBTAG00000015072	FZD8	13	27056507	27057989	-	CODING	-1.30703	0.033356
ENSBTAT00000053553	ENSBTAG00000009391	ADPRH	1	64933574	64937122	+	CODING	-1.19296	0.03346
ENSBTAT00000025655	ENSBTAG00000019264	EDEM3	16	67209942	67278874	-	CODING	-0.78606	0.033536
ENSBTAT00000014805	ENSBTAG00000011150	PFN2	1	119151123	119157806	+	CODING	-0.17553	0.033684
ENSBTAT00000038321	ENSBTAG00000026819	HDAC7	5	32635940	32667499	+	CODING	0.703505	0.033757
ENSBTAT00000021144	ENSBTAG00000015904	RORA	10	48949618	49750993	+	CODING	-0.55807	0.033766
ENSBTAT00000017770	ENSBTAG00000013358	RPS23	7	84523780	84525639	-	CODING	0.273635	0.033767
ENSBTAT00000045545	ENSBTAG00000008837	CSNK2B	23	27447511	27451477	-	CODING	0.206979	0.033773
ENSBTAT00000039322	ENSBTAG00000027397	PPP1R3D	13	57120666	57121565	+	CODING	-1.70284	0.033801
ENSBTAT00000054501	ENSBTAG00000037735	GPR77	18	54777175	54781917	+	CODING	0.813475	0.033855
ENSBTAT00000027942	ENSBTAG00000020981	42799	26	13831827	13865056	+	CODING	-0.44798	0.033889
ENSBTAT00000013969	ENSBTAG00000010563	PTPN2	24	43539648	43607714	-	CODING	0.538711	0.034074
ENSBTAT00000061579	ENSBTAG00000043964	ARL5B	13	20592075	20613350	+	CODING	-0.99295	0.034082
ENSBTAT00000026902	ENSBTAG00000020199	F2R	10	7879496	7897150	+	CODING	1.033825	0.034093
ENSBTAT00000006418	ENSBTAG00000004879	FOXO4	X	84822740	84831551	-	CODING	-0.50975	0.034142
ENSBTAT00000063261	ENSBTAG00000047874	PRPF38B	3	34785792	34793589	-	CODING	0.493915	0.034199
ENSBTAT00000018349	ENSBTAG00000013801	PBX1	3	4228463	4527283	-	CODING	-1.03521	0.034235
ENSBTAT00000025020	ENSBTAG00000018796	RABGGTA	10	20726776	20733197	+	CODING	-0.56467	0.034246
ENSBTAT00000031526	ENSBTAG00000009523	SLC9A6	X	19608676	19659279	+	CODING	-0.65918	0.034302
ENSBTAT00000047358	ENSBTAG00000033315	DNAJC1	13	23251449	23428980	-	CODING	-5.95871	0.034314
ENSBTAT00000027449	ENSBTAG00000020602	IDO1	27	34686577	34699480	+	CODING	0.874728	0.03433

ENSBTAT00000011390	ENSBTAG00000008636	PDE4B	3	79286815	79734224	-	CODING	-0.88895	0.034367
ENSBTAT00000022460	ENSBTAG00000016886	MT3	18	24134095	24135544	-	CODING	1.621404	0.034395
ENSBTAT00000025161	ENSBTAG00000018905	MAN2A2	21	22166073	22183354	-	CODING	-0.6452	0.034442
ENSBTAT00000017493	ENSBTAG00000013159	PHF11	12	19150794	19184692	+	CODING	0.709931	0.034469
ENSBTAT00000008730	ENSBTAG00000006645	P2RY5	12	18282157	18284006	-	CODING	0.84569	0.034562
ENSBTAT00000061127	ENSBTAG00000044105	FOXO1	12	21915747	22005338	+	CODING	0.347013	0.034617
ENSBTAT00000001290	ENSBTAG00000000974	IARS	8	85268890	85350117	-	CODING	-0.57265	0.034695
ENSBTAT00000018238	ENSBTAG00000013725	ZFYVE20	22	57838728	57857029	+	CODING	-0.91907	0.034697
ENSBTAT00000003726	ENSBTAG00000002874	TMEM183	16	519685	534911	+	CODING	0.569853	0.034845
ENSBTAT00000001253	ENSBTAG00000000948	RAB2A	14	27864735	27937015	+	CODING	0.158455	0.035046
ENSBTAT00000047860	ENSBTAG00000012585	RAB3GAP2	16	24330707	24433700	-	CODING	-0.5814	0.035092
ENSBTAT00000030005	ENSBTAG00000022227	PLSCR2	1	122911096	122937932	+	CODING	0.919117	0.035098
ENSBTAT00000004739	ENSBTAG00000003634	CISD1	26	742617	760794	-	CODING	-0.28535	0.035125
ENSBTAT00000024660	ENSBTAG00000018527	HDGFRP3	21	25317912	25392905	+	CODING	0.709768	0.03514
ENSBTAT00000004378	ENSBTAG00000003376	ASB1	3	118353153	118372314	+	CODING	-1.11796	0.035276
ENSBTAT00000004922	ENSBTAG00000006876	PMPCB	4	44755331	44769491	+	CODING	-0.35403	0.035289
ENSBTAT00000048781	ENSBTAG00000010945	SEC24B	6	16978876	17058815	-	CODING	-0.50855	0.03529
ENSBTAT00000013200	ENSBTAG00000010009	TMCO1	3	3166689	3222619	+	CODING	0.387433	0.035315
ENSBTAT00000063674	ENSBTAG00000047545	RGAG4	X	83676302	83678026	+	CODING	0.932516	0.035338
ENSBTAT00000034656	ENSBTAG00000024889	HSBP1	18	10172302	10175455	+	CODING	0.205135	0.035409
ENSBTAT00000003094	ENSBTAG00000002391	TGFB1I1	25	27760811	27766954	+	CODING	0.658464	0.035423
ENSBTAT00000061310	ENSBTAG00000044040	PDAP1	25	37532740	37544696	+	CODING	0.21327	0.035457
ENSBTAT00000025577	ENSBTAG00000018419	ADCY4	10	20671131	20685956	+	CODING	0.986984	0.035463
ENSBTAT00000021043	ENSBTAG00000015840	HOXD1	2	20759890	20761196	-	CODING	-1.70943	0.035474
ENSBTAT00000047363	ENSBTAG00000010518	SKIV2L2	20	23727322	23853276	-	CODING	-0.65375	0.03556
ENSBTAT00000061249	ENSBTAG00000009299	AFTPH	11	62911414	62951512	+	CODING	-0.49716	0.035683
ENSBTAT00000017987	ENSBTAG00000013528	CHMP7	8	71228581	71251787	+	CODING	-0.46914	0.035717
ENSBTAT00000024370	ENSBTAG00000018314	MED11	19	27253471	27255303	-	CODING	0.899949	0.035772
ENSBTAT00000015135	ENSBTAG00000011390	CHRND	2	120975092	120982350	+	CODING	1.043225	0.035809
ENSBTAT00000013214	ENSBTAG00000010018	ATP7A	X	79402956	79473209	-	CODING	-1.02118	0.03582
ENSBTAT00000014217	ENSBTAG00000010739	BLOC1S2	26	21043927	21053587	-	CODING	0.411633	0.035872
ENSBTAT00000015189	ENSBTAG00000011425	PTPRA	13	52600919	52771800	-	CODING	0.764895	0.035949
ENSBTAT00000032377	ENSBTAG00000023652	PROS1	1	37803108	37866950	-	CODING	1.015384	0.035971
ENSBTAT00000033359	ENSBTAG00000031723	RPL6	17	64115683	64120590	+	CODING	0.247067	0.035984
ENSBTAT00000056899	ENSBTAG00000026394	MGC142811	25	3744167	3749205	-	CODING	-0.34093	0.036044
ENSBTAT00000012635	ENSBTAG00000009603	UQCRH	3	100391833	100401195	-	CODING	0.224939	0.036073
ENSBTAT00000000027	ENSBTAG00000000025	-	15	53936927	54027857	-	CODING	-0.28001	0.036119
ENSBTAT00000055067	ENSBTAG00000037942	-	13	74496558	74498471	-	CODING	-0.74907	0.036122
ENSBTAT00000003869	ENSBTAG00000002971	CUTC	26	20550908	20583501	+	CODING	-0.24326	0.036173
ENSBTAT00000003525	ENSBTAG00000002721	ZCCHC11	3	94414997	94512804	+	CODING	-0.51339	0.036196
ENSBTAT00000024959	ENSBTAG00000018743	C5H12orf29	5	17892374	17902271	+	CODING	-0.55209	0.036247
ENSBTAT00000016001	ENSBTAG00000012065	DNAJC3	12	76958978	77024110	+	CODING	0.386003	0.036786
ENSBTAT00000044865	ENSBTAG00000002014	SNX1	10	45899489	45936333	-	CODING	-0.85116	0.036836
ENSBTAT00000033494	ENSBTAG00000003495	KDM7A	4	104370184	104406730	-	CODING	-0.69461	0.036841
ENSBTAT00000002300	ENSBTAG00000001754	AHCYL2	4	93956616	94148560	+	CODING	-0.46264	0.036897
ENSBTAT00000046264	ENSBTAG00000032598	RP9	4	64131287	64143143	+	CODING	0.547418	0.037005
ENSBTAT00000003072	ENSBTAG00000002377	PSMB2	3	110720283	110756534	+	CODING	0.216195	0.037033
ENSBTAT00000045398	ENSBTAG00000010196	NUP43	9	88051512	88066828	-	CODING	-0.8006	0.037084
ENSBTAT00000026033	ENSBTAG00000019538	FBXO28	16	28009798	28033718	+	CODING	-0.6358	0.037154
ENSBTAT00000047156	ENSBTAG00000006884	PABPN1	10	21427968	21432160	-	CODING	0.633103	0.037194
ENSBTAT00000028017	ENSBTAG00000021036	42796	7	18269254	18283870	+	CODING	0.450018	0.037206
ENSBTAT00000000611	ENSBTAG00000000483	HYAL1	22	50600489	50601799	+	CODING	0.756669	0.037381
ENSBTAT00000043931	ENSBTAG00000019894	C22orf39	17	74711564	74715846	-	CODING	0.803634	0.037405
ENSBTAT00000001935	ENSBTAG00000001478	PPPDE1	16	33290070	33304935	-	CODING	-0.84917	0.037491
ENSBTAT00000061343	ENSBTAG00000013271	NCOR1	19	33945404	34043863	+	CODING	-0.59115	0.037498
ENSBTAT00000035878	ENSBTAG00000008959	-	23	27600055	27604052	+	CODING	1.474307	0.037577
ENSBTAT00000061016	ENSBTAG00000007236	PCM1	27	18328847	18405603	-	CODING	-0.53548	0.037611
ENSBTAT00000024292	ENSBTAG00000018249	KCNN3	3	15781068	15948350	+	CODING	-0.43141	0.037695
ENSBTAT00000005606	ENSBTAG00000004281	ACSS1	13	43029813	43073610	-	CODING	1.028729	0.037774
ENSBTAT00000019352	ENSBTAG00000014560	HLX	16	25059261	25064940	+	CODING	1.102653	0.037801
ENSBTAT00000012035	ENSBTAG00000030913	MX1	1	143176083	143204865	+	CODING	1.543933	0.037872
ENSBTAT00000029669	ENSBTAG00000039630	TMED2	17	54330421	54338333	-	CODING	0.255594	0.037928
ENSBTAT00000016796	ENSBTAG00000012658	TMA16	6	2089641	2119479	-	CODING	0.457581	0.037965
ENSBTAT00000022677	ENSBTAG00000017053	ABCB10	28	492423	525181	-	CODING	-0.7605	0.03812

ENSBTAT00000034094	ENSBTAG00000020014	CEP104	16	50498425	50533080	+	CODING	-0.71746	0.038135
ENSBTAT00000020593	ENSBTAG00000015467	FAM184A	9	32425508	32497610	+	CODING	-1.29428	0.038176
ENSBTAT00000031780	ENSBTAG00000015129	KLK10	18	57448225	57454197	-	CODING	1.634996	0.038238
ENSBTAT00000018887	ENSBTAG00000014207	ADAMTS10	7	18391616	18408987	-	CODING	1.388271	0.038274
ENSBTAT00000024296	ENSBTAG00000018247	OCRL	X	13452405	13501004	+	CODING	-0.70764	0.038339
ENSBTAT00000064741	ENSBTAG00000018996	PARK2	9	98421510	99411209	-	CODING	-2.22988	0.038391
ENSBTAT00000056365	ENSBTAG00000013952	HNRPD	6	98916663	98933795	-	CODING	0.235621	0.038498
ENSBTAT00000063094	ENSBTAG00000047856	PAK2	1	71867089	71952257	+	CODING	-0.37895	0.038615
ENSBTAT00000024834	ENSBTAG00000018663	GPRC5C	19	57642755	57655943	-	CODING	-0.45498	0.038652
ENSBTAT00000017232	ENSBTAG00000012962	SARS	3	34283445	34303242	-	CODING	0.354001	0.038659
ENSBTAT00000028884	ENSBTAG00000021675	PJA2	7	111045732	111095494	-	CODING	-0.54854	0.038662
ENSBTAT00000001868	ENSBTAG00000001425	FBXO11	11	29989995	30101580	-	CODING	-0.41872	0.038719
ENSBTAT00000010912	ENSBTAG00000008292	SNRPD1	24	34993832	35001567	-	CODING	0.470321	0.038792
ENSBTAT00000055799	ENSBTAG00000000838	CCDC88C	21	56629747	56645863	-	CODING	-0.74253	0.038873
ENSBTAT00000022798	ENSBTAG00000027080	SLC8A3	10	82128379	82273227	-	CODING	-0.97675	0.038883
ENSBTAT00000032097	ENSBTAG00000023513	DNAJC19	1	86666813	86672081	+	CODING	0.364519	0.038965
ENSBTAT00000028604	ENSBTAG00000021457	EFEMP2	29	44650628	44657896	-	CODING	1.130621	0.038999
ENSBTAT00000017179	ENSBTAG00000012929	CEP85L	9	32779814	32900894	+	CODING	-0.62878	0.039063
ENSBTAT00000026634	ENSBTAG00000019997	TRHR	14	57479102	57524004	-	CODING	-4.84694	0.039189
ENSBTAT00000018675	ENSBTAG00000014051	ANGPT1	14	59137214	59436333	+	CODING	-1.32734	0.039363
ENSBTAT00000064605	ENSBTAG00000048179	-	3	83474568	83474966	-	CODING	2.191252	0.039374
ENSBTAT00000021305	ENSBTAG00000016005	PPP3CA	6	24812682	25136247	+	CODING	-0.65948	0.039393
ENSBTAT00000018604	ENSBTAG00000013999	FAM20B	16	61810681	61852109	+	CODING	-0.87507	0.039431
ENSBTAT00000044735	ENSBTAG00000016720	RAB1A	11	63429725	63458565	-	CODING	-1.67282	0.039448
ENSBTAT00000009356	ENSBTAG00000007111	STEAP3	2	71380830	71424474	+	CODING	-0.81204	0.039525
ENSBTAT00000061485	ENSBTAG00000004664	COQ4	11	98931366	98945011	+	CODING	-0.53389	0.039578
ENSBTAT00000010432	ENSBTAG00000007935	CALCOCO2	19	38314473	38338074	-	CODING	0.232951	0.039695
ENSBTAT00000056395	ENSBTAG00000038630	KLHL34	X	128884989	128886938	+	CODING	-0.59801	0.039752
ENSBTAT00000038690	ENSBTAG00000027024	ARHGAP5	21	42937760	43000529	+	CODING	-0.67265	0.03976
ENSBTAT00000030411	ENSBTAG00000022460	-	29	37575178	37575612	+	CODING	1.250276	0.039842
ENSBTAT00000023042	ENSBTAG00000017328	PTI	13	74943621	74947760	-	CODING	0.631219	0.03995
ENSBTAT00000023638	ENSBTAG00000017776	CHD2	21	14390575	14420462	-	CODING	0.686127	0.039979
ENSBTAT00000004201	ENSBTAG00000003237	IQSEC1	22	59460406	59549010	+	CODING	-0.89033	0.040066
ENSBTAT00000020595	ENSBTAG00000031852	TBC1D10B	25	26801396	26811293	-	CODING	-0.62453	0.040108
ENSBTAT00000003191	ENSBTAG00000002455	KLHL8	6	103844033	103871753	-	CODING	-1.2161	0.040399
ENSBTAT00000022907	ENSBTAG00000017239	GABBR1	23	28775534	28803895	+	CODING	1.124215	0.040456
ENSBTAT00000002011	ENSBTAG00000001537	TSKU	15	56961320	56974713	+	CODING	0.812239	0.040459
ENSBTAT00000002508	ENSBTAG00000001928	PDIA6	11	86834898	86857648	+	CODING	0.398248	0.040473
ENSBTAT00000020395	ENSBTAG00000015327	SPTAN1	11	99131533	99179460	+	CODING	0.567474	0.040474
ENSBTAT00000063090	ENSBTAG00000046231	-	3	8770080	8772051	+	CODING	-0.91063	0.040606
ENSBTAT00000005462	ENSBTAG00000004173	UBXN8	27	26008823	26026228	+	CODING	0.650377	0.040677
ENSBTAT00000012534	ENSBTAG00000009526	TMEM59	3	92746059	92771330	+	CODING	0.351438	0.040705
ENSBTAT00000042553	ENSBTAG00000021231	KCTD2	19	57008161	57020015	-	CODING	-0.67004	0.040776
ENSBTAT00000034803	ENSBTAG00000019660	TMUB2	19	44629959	44633906	+	CODING	-7.42308	0.040796
ENSBTAT00000017428	ENSBTAG00000013111	RRM1	15	51801715	51843018	-	CODING	-0.54738	0.040872
ENSBTAT00000063314	ENSBTAG00000012638	S100A12	3	17163820	17165144	+	CODING	1.028708	0.040873
ENSBTAT00000009936	ENSBTAG00000033806	-	4	31693514	31694260	+	CODING	1.420433	0.040914
ENSBTAT00000035343	ENSBTAG00000030616	ZCCHC10	7	46188839	46202621	-	CODING	-0.60676	0.040962
ENSBTAT00000028860	ENSBTAG00000021657	CACNG6	18	62106778	62121184	+	CODING	-0.38554	0.040985
ENSBTAT00000057477	ENSBTAG00000001030	MTMR3	17	71122533	71253675	+	CODING	-0.58161	0.041025
ENSBTAT00000053590	ENSBTAG00000037991	-	15	46902643	46904084	+	CODING	1.13518	0.041094
ENSBTAT00000061257	ENSBTAG00000010229	LAMA2	9	68129102	68665593	+	CODING	0.499348	0.041117
ENSBTAT00000016117	ENSBTAG00000012149	HOXC8	5	26180271	26182515	-	CODING	-0.3823	0.04118
ENSBTAT00000011368	ENSBTAG00000008619	SAMD8	28	31096197	31119893	+	CODING	-0.55583	0.041199
ENSBTAT00000012415	ENSBTAG00000009435	SMG5	3	14569447	14597121	+	CODING	-0.4643	0.041281
ENSBTAT00000014572	ENSBTAG00000010977	FRZB	2	13724668	13761787	+	CODING	-0.44153	0.041294
ENSBTAT00000003828	ENSBTAG00000002941	FES	21	22192640	22204117	-	CODING	1.531107	0.041312
ENSBTAT00000017563	ENSBTAG00000013191	AGRN	16	52674207	52712338	-	CODING	-0.6929	0.041319
ENSBTAT00000024010	ENSBTAG00000018035	TGFBR1	8	64570093	64641796	+	CODING	-0.91631	0.041397
ENSBTAT00000002324	ENSBTAG00000001774	SPRY2	12	55684001	55685894	-	CODING	0.50185	0.041405
ENSBTAT00000047455	ENSBTAG00000007705	COLEC12	24	35630929	35816269	-	CODING	0.800806	0.041464
ENSBTAT00000002992	ENSBTAG00000002321	AMT	22	51257611	51261937	-	CODING	0.865699	0.041494
ENSBTAT00000046140	ENSBTAG00000007129	MRVI1	15	42548308	42674631	+	CODING	4.789289	0.041576
ENSBTAT00000004630	ENSBTAG00000003560	-	12	18173185	18173958	-	CODING	0.539597	0.041597

ENSBTAT00000037527	ENSBTAG00000012683	SRRM2	25	2286717	2304536	-	CODING	-0.23704	0.041615
ENSBTAT00000053197	ENSBTAG00000018749	NUDT16	1	140044681	140046241	-	CODING	1.524111	0.04171
ENSBTAT00000028645	ENSBTAG00000021491	NDUFV3	1	144690277	144701618	+	CODING	-0.21327	0.041742
ENSBTAT00000013687	ENSBTAG00000010366	HCRTR1	2	122641987	122649721	-	CODING	1.179365	0.041753
ENSBTAT00000019575	ENSBTAG00000014710	RAP1A	3	31746448	31764423	-	CODING	0.235944	0.041769
ENSBTAT00000029813	ENSBTAG00000022114	TMEM65	14	17254750	17298970	+	CODING	-0.54831	0.041862
ENSBTAT00000022350	ENSBTAG00000016801	RXRG	3	3576688	3636911	+	CODING	-0.72773	0.041971
ENSBTAT00000012216	ENSBTAG00000009272	ELMSAN1	10	85517890	85536667	-	CODING	-0.74922	0.041978
ENSBTAT00000002350	ENSBTAG00000001795	LONP1	7	19814902	19833230	+	CODING	-0.51598	0.042072
ENSBTAT00000065252	ENSBTAG00000003697	TARDBP	16	43481408	43486458	-	CODING	-0.33728	0.042127
ENSBTAT00000048910	ENSBTAG00000034529	HMGA1	23	8258750	8268920	+	CODING	-0.40146	0.042238
ENSBTAT00000026568	ENSBTAG00000019944	METAP2	5	25255494	25286217	+	CODING	-0.25476	0.042492
ENSBTAT00000033952	ENSBTAG00000012405	PEAR1	3	13986478	14007210	-	CODING	0.729038	0.042535
ENSBTAT00000001839	ENSBTAG00000001400	AKT2	18	49904012	49950072	-	CODING	-0.39722	0.042544
ENSBTAT00000023596	ENSBTAG00000017747	PLSCR3	19	27706379	27710913	-	CODING	0.871714	0.042547
ENSBTAT00000008994	ENSBTAG00000006852	ACYP2	11	36683363	36862401	+	CODING	-0.17766	0.042586
ENSBTAT00000046682	ENSBTAG00000032881	SLCO5A1	14	35583263	35727312	-	CODING	-1.42984	0.042587
ENSBTAT00000021350	ENSBTAG00000016046	GOPC	9	33628854	33665784	+	CODING	-0.75975	0.04263
ENSBTAT00000001974	ENSBTAG00000001509	ELK3	5	60823240	60891359	+	CODING	0.513479	0.042758
ENSBTAT00000006439	ENSBTAG00000004895	AFAP1L2	26	35050366	35166042	-	CODING	1.271797	0.042843
ENSBTAT00000021025	ENSBTAG00000015828	FKBP11	5	31048546	31052006	+	CODING	1.560495	0.042843
ENSBTAT00000024728	ENSBTAG00000018579	DOK1	11	10068212	10071072	-	CODING	2.700671	0.042846
ENSBTAT00000008766	ENSBTAG00000006678	GATAD2B	3	16633874	16644316	+	CODING	-0.62143	0.042874
ENSBTAT00000043540	ENSBTAG00000010264	FBXL18	25	39359958	39376730	+	CODING	-1.29141	0.042908
ENSBTAT00000015635	ENSBTAG00000011770	ZO2	8	45649371	45696886	+	CODING	1.001648	0.042922
ENSBTAT00000052616	ENSBTAG00000004294	EVI5L	7	17832596	17865095	+	CODING	-0.74905	0.042928
ENSBTAT00000049047	ENSBTAG00000039875	-	9	88307676	88310265	-	CODING	-2.51166	0.04298
ENSBTAT00000015674	ENSBTAG00000011808	MSTN	2	6213566	6220196	+	CODING	-1.15012	0.042981
ENSBTAT00000032060	ENSBTAG00000003788	RCC1	2	125468461	125475905	-	CODING	-1.11876	0.043028
ENSBTAT00000059654	ENSBTAG00000042662	SNORD17	13	38537849	38538084	-	CODING	2.299873	0.043061
ENSBTAT00000061174	ENSBTAG00000021250	RALBP1	24	42061941	42079438	+	CODING	-0.48374	0.043269
ENSBTAT00000040283	ENSBTAG00000009727	ADIPOR1	16	55522005	55527367	-	CODING	-0.28001	0.043333
ENSBTAT00000004621	ENSBTAG00000003553	ZFP36L2	11	25585493	25587785	-	CODING	0.426082	0.043381
ENSBTAT00000002891	ENSBTAG00000046814	ACOT2	10	85358158	85365337	+	CODING	0.878637	0.043648
ENSBTAT00000056838	ENSBTAG00000010520	C8G	11	106284927	106286380	-	CODING	1.568866	0.043931
ENSBTAT00000043890	ENSBTAG00000038321	HSL	18	51216019	51227395	+	CODING	0.665962	0.043936
ENSBTAT00000046091	ENSBTAG00000021242	COQ7	25	17011665	17025337	-	CODING	0.587338	0.043946
ENSBTAT00000015933	ENSBTAG00000034147	OLFML2B	3	7528022	7577290	+	CODING	1.229857	0.044052
ENSBTAT00000063012	ENSBTAG00000045699	CTNNA3	28	22419203	24270401	-	CODING	-1.0262	0.044098
ENSBTAT00000006658	ENSBTAG00000005048	DHRS7C	19	29581010	29594334	-	CODING	-0.16433	0.044128
ENSBTAT00000054111	ENSBTAG00000039130	-	7	14670708	14672994	-	CODING	0.264188	0.044297
ENSBTAT00000011856	ENSBTAG00000009005	DUSP15	13	61941884	61950327	-	CODING	-0.45699	0.044303
ENSBTAT00000021840	ENSBTAG00000016421	-	9	101442938	101443961	+	CODING	-0.61668	0.044337
ENSBTAT00000033917	ENSBTAG00000000113	ARHGEF2	3	14843522	14868193	+	CODING	0.705392	0.044348
ENSBTAT00000013464	ENSBTAG00000010204	PCMT1	9	88068718	88101563	+	CODING	-0.24485	0.044398
ENSBTAT00000014511	ENSBTAG00000010923	RAB9A	X	137004145	137023608	-	CODING	0.494412	0.044491
ENSBTAT00000006754	ENSBTAG00000005119	PSMD1	2	119675723	119756976	+	CODING	-0.27932	0.044513
ENSBTAT00000012612	ENSBTAG00000009584	SAPS3	29	46632382	46716791	+	CODING	-0.39158	0.044689
ENSBTAT00000065242	ENSBTAG00000015037	GAREML	11	73292031	73307412	-	CODING	-2.21339	0.044769
ENSBTAT00000037233	ENSBTAG00000003446	EPHB4	25	36347294	36362877	+	CODING	0.86656	0.044834
ENSBTAT00000020671	ENSBTAG00000015559	ERI3	3	102290329	102421170	+	CODING	-0.47929	0.045052
ENSBTAT00000000445	ENSBTAG00000000340	ADNP	13	79548065	79556310	-	CODING	-0.4755	0.04508
ENSBTAT00000004763	ENSBTAG00000003653	LYAR	6	107443252	107463870	+	CODING	0.933712	0.045168
ENSBTAT00000037556	ENSBTAG00000026428	ATP6V0C	25	2014229	2019603	+	CODING	0.260901	0.045518
ENSBTAT00000011595	ENSBTAG00000008802	C19H17ORF39	19	35111312	35125579	-	CODING	-0.55809	0.045556
ENSBTAT00000020766	ENSBTAG00000015637	IMPAD1	14	25544907	25560879	-	CODING	-0.48758	0.045701
ENSBTAT00000007704	ENSBTAG00000005863	-	5	26580498	26671702	+	CODING	-0.631	0.045736
ENSBTAT00000023836	ENSBTAG00000040131	CD58	3	26825753	26889208	+	CODING	0.559487	0.045755
ENSBTAT00000061073	ENSBTAG00000037508	EBF1	7	72396487	72804182	-	CODING	0.628011	0.045792
ENSBTAT00000015161	ENSBTAG00000011406	TESK1	8	60207472	60212368	+	CODING	-0.36021	0.045799
ENSBTAT00000029948	ENSBTAG00000006686	NPNT	6	20450525	20529959	-	CODING	-0.74443	0.045837
ENSBTAT00000021189	ENSBTAG00000015926	ABLIM2	6	119107715	119213224	-	CODING	-0.38764	0.045959
ENSBTAT00000025152	ENSBTAG00000018897	PSMD11	19	18035610	18064853	-	CODING	-0.27765	0.04601
ENSBTAT00000050590	ENSBTAG00000006388	FBXO45	1	71710368	71721053	+	CODING	-1.31268	0.04605

ENSBTAT00000009355	ENSBTAG00000007110	RCOR3	16	73955570	74006866	-	CODING	-0.54471	0.046158
ENSBTAT00000045907	ENSBTAG00000032360	-	6	85640347	85641522	+	CODING	0.446158	0.046186
ENSBTAT00000064443	ENSBTAG00000047788	-	8	53587372	53632760	+	CODING	-0.86725	0.046196
ENSBTAT00000034204	ENSBTAG00000025426	NSD1	7	39982435	40123961	+	CODING	-0.52545	0.046207
ENSBTAT00000006800	ENSBTAG00000005158	NAPG	24	42616054	42628182	+	CODING	-0.53941	0.046219
ENSBTAT00000005683	ENSBTAG00000004343	CCS	29	45274394	45288010	+	CODING	0.691653	0.04626
ENSBTAT00000010628	ENSBTAG00000008083	SEL1L	10	93904711	93965166	-	CODING	-0.58107	0.046372
ENSBTAT00000044026	ENSBTAG00000003809	PLCD4	2	107298620	107321320	+	CODING	0.325629	0.046426
ENSBTAT00000023208	ENSBTAG00000017460	PRORSD1	11	37859124	37861215	+	CODING	0.692762	0.046542
ENSBTAT00000014951	ENSBTAG00000011256	MYO1B	2	80166217	80369680	+	CODING	0.609464	0.046549
ENSBTAT00000055230	ENSBTAG00000002176	NSMCE1	25	25059384	25068747	+	CODING	0.302455	0.046593
ENSBTAT00000023198	ENSBTAG00000017451	TSPAN17	7	39472617	39481385	+	CODING	-0.44958	0.046611
ENSBTAT00000061398	ENSBTAG00000016316	ZZEF1	19	25230019	25322722	-	CODING	-0.94796	0.046689
ENSBTAT00000012597	ENSBTAG00000009579	CEP85	2	127433456	127463144	-	CODING	-0.3218	0.046707
ENSBTAT00000007090	ENSBTAG00000005390	GMFG	18	49309015	49313926	-	CODING	1.354695	0.046734
ENSBTAT00000065273	ENSBTAG00000047766	G0S2	16	75539496	75540404	-	CODING	1.423296	0.046775
ENSBTAT00000000085	ENSBTAG00000000077	ADSL	5	112192430	112209091	+	CODING	-0.32686	0.046816
ENSBTAT00000025677	ENSBTAG00000019284	-	11	84076854	84078206	-	CODING	-0.24057	0.047162
ENSBTAT00000063149	ENSBTAG00000018937	HMOX2	25	3621447	3649059	+	CODING	-2.186	0.047382
ENSBTAT00000001346	ENSBTAG00000001017	SLK	26	24786360	24844579	+	CODING	-0.66442	0.047384
ENSBTAT00000063462	ENSBTAG00000046509	FAM46C	3	25703050	25705109	-	CODING	-0.91985	0.047603
ENSBTAT00000053709	ENSBTAG00000039618	C10H11ORF46	10	4872818	4904741	+	CODING	1.859475	0.047698
ENSBTAT00000021315	ENSBTAG00000016013	DNAH12	22	44377845	44392469	+	CODING	-0.44979	0.047852
ENSBTAT00000004639	ENSBTAG00000003567	FXC1	15	47185421	47186287	-	CODING	1.443025	0.048044
ENSBTAT00000008137	ENSBTAG00000006195	C29H11orf68	29	44691682	44693901	-	CODING	-0.54238	0.048061
ENSBTAT00000013221	ENSBTAG00000010023	FAM107B	13	29501668	29578090	-	CODING	0.890561	0.0481
ENSBTAT00000004806	ENSBTAG00000003690	NHLRC2	26	34584585	34640028	+	CODING	-0.68633	0.048104
ENSBTAT00000015624	ENSBTAG00000011763	RIOK2	7	99013511	99035256	-	CODING	0.432661	0.04833
ENSBTAT00000011499	ENSBTAG00000008728	MKL2	25	13072437	13283317	+	CODING	-0.70569	0.048331
ENSBTAT00000025061	ENSBTAG00000018829	SPRYD7	12	19493438	19511216	-	CODING	-0.39002	0.048441
ENSBTAT00000013888	ENSBTAG00000010513	GPS2	19	27653444	27656067	-	CODING	0.230942	0.048447
ENSBTAT00000055460	ENSBTAG00000039684	PTRF	19	43154126	43162165	-	CODING	-0.41025	0.04847
ENSBTAT00000008956	ENSBTAG00000006818	TMEM185A	X	32950185	32963181	+	CODING	-0.51287	0.048518
ENSBTAT00000010311	ENSBTAG00000007836	PPA1	28	26576202	26614511	-	CODING	0.349644	0.048541
ENSBTAT00000029894	ENSBTAG00000022160	NAP1L4	29	49264642	49316917	+	CODING	-0.25467	0.048604
ENSBTAT00000065091	ENSBTAG00000011994	NDEL1	19	28637771	28672316	+	CODING	0.390098	0.04863
ENSBTAT00000063747	ENSBTAG00000047361	-	4	93662296	93662746	+	CODING	0.280718	0.048827
ENSBTAT00000047015	ENSBTAG00000006835	MCAM	15	30414474	30422213	-	CODING	1.087333	0.048846
ENSBTAT00000012551	ENSBTAG00000009541	SUCLG2	22	33977312	34251728	+	CODING	-0.20419	0.048901
ENSBTAT00000050141	ENSBTAG00000035654	-	1	137783195	137783923	+	CODING	1.191744	0.048928
ENSBTAT00000009064	ENSBTAG00000006898	EPRS	16	24171351	24236353	-	CODING	-0.37432	0.048975
ENSBTAT00000012905	ENSBTAG00000009784	RAB32	9	84937829	84969252	+	CODING	1.025984	0.049069
ENSBTAT00000059593	ENSBTAG00000042601	7SK	9	39583059	39583372	+	CODING	1.574491	0.049152
ENSBTAT00000000081	ENSBTAG00000000073	LRPAP1	6	107207883	107221544	+	CODING	0.618065	0.049232
ENSBTAT00000028372	ENSBTAG00000021294	VWA1	16	52360276	52365505	-	CODING	0.959547	0.049312
ENSBTAT00000004805	ENSBTAG00000003687	FOXK2	19	50650350	50695396	-	CODING	-0.48067	0.049392
ENSBTAT00000003276	ENSBTAG00000002525	C7H19orf53	7	12942938	12948365	+	CODING	0.489758	0.049396
ENSBTAT00000015932	ENSBTAG00000012010	PANX1	29	590732	644582	-	CODING	-0.85673	0.049553
ENSBTAT00000008854	ENSBTAG00000006735	STAC	22	10160885	10273691	+	CODING	1.326104	0.049566
ENSBTAT00000017524	ENSBTAG00000013671	UTP14A	X	13790537	13807198	+	CODING	0.372613	0.049576
ENSBTAT00000001575	ENSBTAG00000038238	-	16	60651006	60651572	+	CODING	1.648684	0.049614
ENSBTAT00000065424	ENSBTAG00000011772	PPP1R12B	16	71062132	71137463	+	CODING	-1.29108	0.049793
ENSBTAT00000040376	ENSBTAG00000006937	ABCA10	19	61959366	62008054	+	CODING	-1.59771	0.049857
ENSBTAT00000005939	ENSBTAG00000004521	LSM7	7	22533096	22537819	+	CODING	0.506798	0.049892
ENSBTAT00000027767	ENSBTAG00000020835	KIAA0513	18	11172008	11188928	+	CODING	-1.03709	0.049933
ENSBTAT00000009673	ENSBTAG00000007354	-	19	50009722	50010311	+	CODING	-0.57045	0.049944
ENSBTAT00000050440	ENSBTAG00000016062	C14orf28	21	55153786	55162538	+	CODING	-0.87885	0.049968
ENSBTAT00000014607	ENSBTAG00000010998	FLIP	2	90139395	90172951	+	CODING	0.881444	0.049988

Cow

GeneNames	GeneAcc	GeneName	Chr	Start	End	Strand	Type	log2(Fold_	p-value
ENSBTAT00000060569	ENSBTAG00000043561	COX1	MT	5687	7231	+	CODING	1.213501	0
ENSBTAT00000060547	ENSBTAG00000043568	MT-ND3	MT	9823	10168	+	CODING	-0.53247	0
ENSBTAT00000009327	ENSBTAG00000018204	MYH1	19	30110728	30134757	-	CODING	1.501437	0
ENSBTAT00000059596	ENSBTAG00000042604	U6	14	83907053	83907159	-	CODING	1.279155	0
ENSBTAT00000062950	ENSBTAG00000045757	TNNC1	22	48988984	48991875	+	CODING	-0.71754	0
ENSBTAT00000017177	ENSBTAG00000012927	ALDOA	25	26470488	26475202	-	CODING	0.543027	0
ENSBTAT00000065038	ENSBTAG00000046725	TNNC2	13	75316423	75318650	-	CODING	0.387375	0
ENSBTAT00000060327	ENSBTAG00000043335	U6	14	64324526	64324632	-	CODING	-11.8409	0
ENSBTAT00000044205	ENSBTAG00000031217	MYL6B	5	57489469	57492173	-	CODING	-1.79032	0
ENSBTAT00000060562	ENSBTAG00000043546	MT-ND6	MT	13913	14440	-	CODING	0.433537	0
ENSBTAT00000059374	ENSBTAG00000042382	U6	28	41535977	41536083	+	CODING	-11.6551	0
ENSBTAT00000060553	ENSBTAG00000043564	MT-ATP8	MT	8129	8329	+	CODING	-0.13266	0
ENSBTAT00000024444	ENSBTAG00000018369	MYL2	17	56953813	56961603	-	CODING	-0.55354	0
ENSBTAT00000059949	ENSBTAG00000042957	U6	5	71251611	71251717	-	CODING	-11.5108	0
ENSBTAT00000029890	ENSBTAG00000022158	TNNT3	29	50218484	50233948	-	CODING	0.920405	0
ENSBTAT00000063142	ENSBTAG00000046289	U6	27	6982216	6982322	-	CODING	-11.2057	0
ENSBTAT00000022375	ENSBTAG00000016819	FABP3	2	122723225	122783830	+	CODING	-1.89435	8.27E-298
ENSBTAT00000044796	ENSBTAG00000005373	TPM1	10	47056204	47065846	-	CODING	0.531134	5.23E-270
ENSBTAT00000015186	ENSBTAG00000011424	TPM2	8	60268618	60274720	-	CODING	-0.31431	7.14E-268
ENSBTAT00000063726	ENSBTAG00000046001	U6	19	43534127	43534233	-	CODING	-10.4446	2.79E-222
ENSBTAT00000059974	ENSBTAG00000042982	U6	26	42369695	42369801	-	CODING	-10.4446	2.79E-222
ENSBTAT00000064903	ENSBTAG00000006419	TNNT1	18	62725898	62735263	+	CODING	-1.36983	1.04E-215
ENSBTAT00000030320	ENSBTAG00000011869	CSRP3	29	25994182	26014859	+	CODING	-0.82053	1.47E-207
ENSBTAT00000063025	ENSBTAG00000047231	TNNI1	16	49293852	49303508	-	CODING	-0.90457	1.45E-196
ENSBTAT00000060175	ENSBTAG00000043183	U6	6	115682606	115682712	-	CODING	-10.2031	2.39E-195
ENSBTAT00000028269	ENSBTAG00000021218	MYLPF	25	26815266	26817727	+	CODING	0.289055	2.41E-181
ENSBTAT00000013077	ENSBTAG00000033217	TPM3	3	16376436	16395088	+	CODING	-0.71448	1.52E-170
ENSBTAT00000059165	ENSBTAG00000042173	U6	15	54044311	54044418	-	CODING	-9.89841	9.58E-166
ENSBTAT00000024749	ENSBTAG00000018598	HSPB6	18	46654243	46656762	-	CODING	-0.47453	1.85E-156
ENSBTAT00000059121	ENSBTAG00000042129	U6	2	128245274	128245380	-	CODING	-9.72254	1.22E-150
ENSBTAT00000048981	ENSBTAG00000034580	TMSB4	GJ05[illegible]	35911	36045	+	CODING	-0.79587	6.10E-149
ENSBTAT00000060539	ENSBTAG00000043584	ATP6	MT	8290	8970	+	CODING	-0.12423	1.11E-145
ENSBTAT00000007014	ENSBTAG00000005333	MB	5	74170471	74181260	-	CODING	-0.21036	1.18E-137
ENSBTAT00000018799	ENSBTAG00000014143	ASB5	27	6692314	6736965	-	CODING	-1.04542	1.22E-127
ENSBTAT00000006918	ENSBTAG00000005259	UCP3	15	54213566	54224051	-	CODING	1.131982	4.04E-122
ENSBTAT00000060552	ENSBTAG00000043577	MT-ND4	MT	10529	11906	+	CODING	0.17719	8.23E-115
ENSBTAT00000060577	ENSBTAG00000043563	ND5	MT	12109	13929	+	CODING	0.31695	7.66E-112
ENSBTAT00000046179	ENSBTAG00000011424	TPM2	8	60267452	60274815	-	CODING	-0.46507	2.50E-109
ENSBTAT00000003330	ENSBTAG00000002574	MYOZ2	6	7251253	7289661	-	CODING	-0.92435	1.09E-100
ENSBTAT00000046011	ENSBTAG00000032436	UBC	17	53142511	53143425	+	CODING	-0.95327	1.47E-94
ENSBTAT00000000359	ENSBTAG00000000286	PFKM	5	32312957	32337525	-	CODING	0.747977	2.32E-87
ENSBTAT00000014452	ENSBTAG00000010880	TNNI2	29	50285049	50287648	-	CODING	0.29231	2.77E-87
ENSBTAT00000012797	ENSBTAG00000007090	MYH2	19	30137767	30165109	-	CODING	-0.43038	2.09E-84
ENSBTAT00000036194	ENSBTAG00000025644	CALM	10	103059964	103067071	+	CODING	0.695262	1.45E-82
ENSBTAT00000008593	ENSBTAG00000006541	ATP2A1	25	26188434	26204655	-	CODING	0.459645	2.66E-81
ENSBTAT00000007278	ENSBTAG00000005534	ENO3	19	27073498	27078655	-	CODING	0.402186	4.37E-76
ENSBTAT00000002790	ENSBTAG00000002157	LMOD2	4	88746219	88755270	+	CODING	-1.16784	3.13E-75
ENSBTAT00000060571	ENSBTAG00000043558	ND1	MT	3101	4056	+	CODING	0.14624	6.27E-74
ENSBTAT00000044397	ENSBTAG00000011969	HSPB1	25	34858435	34861074	+	CODING	-0.43465	1.25E-70
ENSBTAT00000001373	ENSBTAG00000001032	PYGM	29	43606012	43617848	-	CODING	0.440334	1.28E-70
ENSBTAT00000065060	ENSBTAG00000046551	SNORA32	25	33716083	33716205	-	CODING	10.28838	2.04E-66
ENSBTAT00000018501	ENSBTAG00000013929	RRAD	18	34746055	34749250	-	CODING	-1.4936	3.11E-66
ENSBTAT00000057357	ENSBTAG00000008401	PFKFB3	13	17380743	17406594	-	CODING	1.014419	5.35E-65
ENSBTAT00000037753	ENSBTAG00000014731	GAPDH	5	104237902	104241979	-	CODING	0.222699	2.63E-62
ENSBTAT00000047412	ENSBTAG00000001601	PKM2	10	18965984	18992445	-	CODING	0.49411	1.35E-59
ENSBTAT00000008420	ENSBTAG00000006419	TNNT1	18	62725898	62735263	+	CODING	-0.40738	2.23E-59
ENSBTAT00000007429	ENSBTAG00000005654	TMSB10	11	49933204	49934214	-	CODING	-0.81079	3.72E-59
ENSBTAT00000065632	ENSBTAG00000046512	CMYA1	22	12549676	12558895	-	CODING	-1.96903	4.07E-58
ENSBTAT00000008371	ENSBTAG00000022158	TNNT3	29	50218484	50233948	-	CODING	-0.32272	5.72E-58
ENSBTAT00000020243	ENSBTAG00000015214	CA3	14	79406494	79416487	-	CODING	-0.44122	1.02E-57

ENSBTAT00000018492	ENSBTAG00000013921	CKM	18	53383534	53392948	-	CODING	0.197444	3.21E-55
ENSBTAT00000046662	ENSBTAG00000013264	RPS24	28	33926497	33931777	+	CODING	-0.46662	6.21E-54
ENSBTAT00000033450	ENSBTAG00000013860	GADD45A	3	77972153	77975265	-	CODING	1.225458	6.99E-54
ENSBTAT00000062174	ENSBTAG00000044741	SNORD97	15	42441794	42441945	+	CODING	4.013614	2.63E-50
ENSBTAT00000044622	ENSBTAG00000031483	-	29	40214778	40215227	-	CODING	0.994032	8.89E-48
ENSBTAT00000019142	ENSBTAG00000014396	TIEG1	14	63951758	63957275	+	CODING	1.003704	6.11E-47
ENSBTAT00000061000	ENSBTAG00000006907	NEB	2	44547340	44754701	+	CODING	0.680009	6.94E-47
ENSBTAT00000019461	ENSBTAG00000014614	ACTA2	26	10662420	10679648	-	CODING	-1.48454	3.75E-46
ENSBTAT00000037243	ENSBTAG00000020035	RCAN1	1	351708	362907	+	CODING	-1.47272	7.29E-46
ENSBTAT00000064427	ENSBTAG00000022158	TNNT3	29	50218484	50233948	-	CODING	-1.69498	1.72E-44
ENSBTAT00000026756	ENSBTAG00000020080	MYBPC2	18	57039094	57064720	+	CODING	0.403916	6.37E-44
ENSBTAT00000062760	ENSBTAG00000045327	ACA64	25	1522971	1523097	+	CODING	9.4621	7.65E-43
ENSBTAT00000006781	ENSBTAG00000005142	RPL37	20	33667205	33669805	+	CODING	-0.46245	1.04E-42
ENSBTAT00000065158	ENSBTAG00000014249	IVNS1ABP	16	67765816	67785616	-	CODING	0.678695	8.01E-42
ENSBTAT00000019583	ENSBTAG00000014718	CST6	29	44766936	44768173	+	CODING	1.402668	7.68E-40
ENSBTAT00000061306	ENSBTAG00000009703	MYH7	10	21325414	21345624	+	CODING	-0.35165	4.05E-39
ENSBTAT00000021862	ENSBTAG00000016444	FAM134B	20	56709603	56758641	+	CODING	-1.84996	5.80E-39
ENSBTAT00000038558	ENSBTAG00000007109	ASB2	21	59126042	59160261	-	CODING	0.61422	1.16E-37
ENSBTAT00000015235	ENSBTAG00000011465	MYBPH	16	760056	768350	-	CODING	1.714679	1.16E-37
ENSBTAT00000005580	ENSBTAG00000000425	RPL17	21	55609537	55610119	+	CODING	-0.62209	2.84E-37
ENSBTAT00000018491	ENSBTAG00000013924	RPS11	18	56404237	56406668	+	CODING	-0.44081	5.20E-37
ENSBTAT00000065039	ENSBTAG00000046623	-	13	64372559	64372993	-	CODING	-0.48268	3.87E-36
ENSBTAT00000034373	ENSBTAG00000023659	MT2	18	24125366	24126333	-	CODING	-1.4093	4.96E-36
ENSBTAT00000008373	ENSBTAG00000006383	-	X	126048949	126049743	+	CODING	-0.94267	1.50E-35
ENSBTAT00000021328	ENSBTAG00000016024	MYL9	24	37820258	37829300	+	CODING	-0.4059	2.11E-34
ENSBTAT00000027713	ENSBTAG00000020795	RPS21	13	55358720	55359979	-	CODING	-0.6927	1.89E-33
ENSBTAT00000016907	ENSBTAG00000012720	ANKRD2	26	18627373	18636269	+	CODING	-0.96051	8.22E-33
ENSBTAT00000020323	ENSBTAG00000015283	RPL32	22	56985249	56989012	+	CODING	-0.48192	9.34E-33
ENSBTAT00000060250	ENSBTAG00000043258	SNORA18	29	1064655	1064785	+	CODING	-7.00132	7.28E-32
ENSBTAT00000018414	ENSBTAG00000013866	RPS27	3	16505732	16507247	-	CODING	-0.59253	8.04E-32
ENSBTAT00000001187	ENSBTAG00000000894	PGK1	X	79282708	79305386	-	CODING	0.55826	3.32E-31
ENSBTAT00000019758	ENSBTAG00000014835	SPARC	7	64878194	64900828	-	CODING	-0.6613	3.78E-31
ENSBTAT00000061449	ENSBTAG00000026986	TTN	2	18054943	18329808	+	CODING	0.78516	4.69E-31
ENSBTAT00000046158	ENSBTAG00000032531	MUSTN1	22	48611898	48613043	+	CODING	-0.36328	3.92E-30
ENSBTAT00000004562	ENSBTAG00000003505	DCN	5	21080013	21119087	-	CODING	-0.8609	3.95E-30
ENSBTAT00000021796	ENSBTAG00000016391	-	9	16325798	16326091	-	CODING	-0.72916	6.71E-30
ENSBTAT00000042547	ENSBTAG00000030164	RPL38	19	57835002	57839034	-	CODING	-0.62173	9.79E-30
ENSBTAT00000030030	ENSBTAG00000022244	ACTN3	29	45230682	45242282	+	CODING	0.40864	1.13E-29
ENSBTAT00000026534	ENSBTAG00000019915	GSN	8	112609393	112639758	+	CODING	-0.87885	3.38E-29
ENSBTAT00000016414	ENSBTAG00000012370	MGP	5	95456444	95459983	+	CODING	-1.12998	3.49E-29
ENSBTAT00000024572	ENSBTAG00000018463	VIM	13	31945012	31952941	+	CODING	-0.74227	1.19E-28
ENSBTAT00000053207	ENSBTAG00000040564	COX7C	10	72298985	72299176	-	CODING	-0.92531	1.92E-27
ENSBTAT00000020229	ENSBTAG00000015204	SMPX	X	128771954	128825159	+	CODING	-0.61437	3.93E-27
ENSBTAT00000033091	ENSBTAG00000015473	RPS27A	11	37823446	37825428	+	CODING	-0.62832	9.33E-27
ENSBTAT00000017826	ENSBTAG00000039340	SCN4B	15	29257448	29277309	-	CODING	0.818896	1.02E-26
ENSBTAT00000059670	ENSBTAG00000042678	SNORA71	13	68003660	68003794	+	CODING	8.576939	1.43E-26
ENSBTAT00000059715	ENSBTAG00000042723	U11	2	125390299	125390431	-	CODING	8.565713	2.07E-26
ENSBTAT00000004190	ENSBTAG00000003229	RPL23	19	40075000	40079360	-	CODING	-0.41441	2.10E-26
ENSBTAT00000026118	ENSBTAG00000019603	LDHB	5	88962679	88981219	+	CODING	-1.04499	3.26E-26
ENSBTAT00000008386	ENSBTAG00000006396	GPI	18	44979578	45007642	+	CODING	0.620498	6.42E-26
ENSBTAT00000059463	ENSBTAG00000042471	SNORA25	29	1067324	1067450	+	CODING	-6.64419	1.08E-25
ENSBTAT00000026259	ENSBTAG00000019701	RPL31	11	6003175	6008528	+	CODING	-0.34064	1.26E-25
ENSBTAT00000059485	ENSBTAG00000042493	SNORA70	X	40366530	40366664	+	CODING	8.507494	1.38E-25
ENSBTAT00000001330	ENSBTAG00000001003	CKMT2	7	83554315	83579633	+	CODING	0.331225	2.04E-25
ENSBTAT00000003997	ENSBTAG00000003074	SLC16A6	19	62486092	62496100	+	CODING	1.454925	1.35E-24
ENSBTAT00000025803	ENSBTAG00000019368	IGFBP7	6	74071067	74150456	-	CODING	-0.77591	1.65E-24
ENSBTAT00000043649	ENSBTAG00000011392	MYBPC1	5	65737956	65840833	+	CODING	-0.27322	1.72E-24
ENSBTAT00000020326	ENSBTAG00000015285	RPS8	3	101816844	101818956	-	CODING	-0.42194	3.25E-24
ENSBTAT00000001791	ENSBTAG00000001360	RPS12	9	71974860	71978200	+	CODING	-0.37347	9.20E-24
ENSBTAT00000002642	ENSBTAG00000002038	RPL14	22	13336573	13339983	+	CODING	-0.44051	9.52E-24
ENSBTAT00000017182	ENSBTAG00000012931	PLN	9	32823612	32834377	-	CODING	-0.56773	1.78E-23
ENSBTAT00000054038	ENSBTAG00000006305	AK1	11	98552024	98561576	-	CODING	0.433212	2.06E-23

ENSBTAT00000004244	ENSBTAG00000027629	ANK1	27	36255859	36265466	-	CODING	0.625507	2.51E-23
ENSBTAT00000022189	ENSBTAG00000016688	-	X	66722399	66723397	-	CODING	0.279254	3.11E-23
ENSBTAT00000036043	ENSBTAG00000005974	APOBEC2	23	14985521	14998187	+	CODING	-0.49367	3.61E-23
ENSBTAT00000055075	ENSBTAG00000011765	GABARAPL1	5	100205186	100215034	-	CODING	-1.10544	5.51E-23
ENSBTAT00000026400	ENSBTAG00000023274	-	5	107749635	107750145	+	CODING	-0.49848	1.16E-22
ENSBTAT00000023750	ENSBTAG00000017866	CD36	4	40581484	40643369	-	CODING	-0.65058	1.74E-22
ENSBTAT00000020080	ENSBTAG00000015089	LGALS1	5	110014543	110017878	+	CODING	-0.65646	2.54E-22
ENSBTAT00000009469	ENSBTAG00000007196	TAGLN	15	28318411	28323666	+	CODING	-0.75262	4.09E-22
ENSBTAT00000046359	ENSBTAG00000018588	TMBIM6	5	30281520	30298109	-	CODING	0.456666	6.55E-22
ENSBTAT00000003377	ENSBTAG00000002610	FKBP3	21	55325021	55335301	-	CODING	-0.75155	8.43E-22
ENSBTAT00000036888	ENSBTAG00000026199	ACTB	25	39343633	39347044	+	CODING	-0.48914	1.07E-21
ENSBTAT00000017030	ENSBTAG00000012818	PDLIM5	6	31419270	31567339	-	CODING	-0.452	1.10E-21
ENSBTAT00000046417	ENSBTAG00000016444	FAM134B	20	56747556	56758641	+	CODING	-1.53264	3.38E-21
ENSBTAT00000014529	ENSBTAG00000010940	HSPB7	2	136635160	136637353	+	CODING	-0.5058	5.06E-21
ENSBTAT00000014219	ENSBTAG00000010741	KBTBD10	2	26780742	26798046	-	CODING	-0.26353	5.47E-21
ENSBTAT00000026932	ENSBTAG00000020223	CASQ1	3	9591852	9601502	-	CODING	0.234564	1.43E-20
ENSBTAT00000014883	ENSBTAG00000011207	CNN1	7	17106394	17114222	+	CODING	-1.72087	3.32E-20
ENSBTAT00000018910	ENSBTAG00000014226	RPL34	6	17828130	17832711	-	CODING	-0.53262	3.87E-20
ENSBTAT00000054758	ENSBTAG00000039121	PTP4A2	2	122350144	122359897	+	CODING	0.330811	5.93E-20
ENSBTAT00000006275	ENSBTAG00000004777	S100B	1	148009651	148016981	-	CODING	-1.11082	6.95E-20
ENSBTAT00000028617	ENSBTAG00000021466	COL3A1	2	7318227	7356937	-	CODING	-1.28364	8.75E-20
ENSBTAT00000027850	ENSBTAG00000020905	RPL11	2	129791372	129795563	-	CODING	-0.48148	1.18E-19
ENSBTAT00000006806	ENSBTAG00000005163	S100A1	3	16812602	16816399	-	CODING	-0.74024	6.90E-19
ENSBTAT00000024376	ENSBTAG00000018320	RPLP1	10	16324341	16326492	+	CODING	-0.19034	7.32E-19
ENSBTAT00000015231	ENSBTAG00000011463	MID1IP1	X	109949360	109951138	-	CODING	-0.77722	8.73E-19
ENSBTAT00000014850	ENSBTAG00000011182	PDLIM1	26	16605968	16646619	-	CODING	-0.91157	9.88E-19
ENSBTAT00000029668	ENSBTAG00000008061	RILPL1	17	54358764	54400473	+	CODING	0.665725	1.14E-18
ENSBTAT00000009155	ENSBTAG00000025385	RPL12	10	83480588	83481217	-	CODING	-0.48226	1.33E-18
ENSBTAT00000063057	ENSBTAG00000046335	-	29	272264	273010	+	CODING	-0.55901	1.39E-18
ENSBTAT00000007041	ENSBTAG00000005353	DES	2	108075460	108082534	+	CODING	-0.19133	1.99E-18
ENSBTAT00000023183	ENSBTAG00000017441	RPL27	19	43669374	43671655	+	CODING	-0.42485	2.21E-18
ENSBTAT00000008887	ENSBTAG00000006752	PFKFB4	22	51898015	51933996	+	CODING	0.843444	2.69E-18
ENSBTAT00000012735	ENSBTAG00000009663	CSDA	5	99335508	99360895	+	CODING	0.207215	3.62E-18
ENSBTAT00000031754	ENSBTAG00000023343	RPL28	18	62547220	62549950	-	CODING	-0.52178	7.45E-18
ENSBTAT00000047021	ENSBTAG00000021499	PSAP	28	28123710	28157099	-	CODING	1.928466	8.17E-18
ENSBTAT00000023485	ENSBTAG00000017655	PALMD	3	43688103	43748131	-	CODING	-1.00871	8.86E-18
ENSBTAT00000014853	ENSBTAG00000011184	FTH1	29	41172509	41175112	-	CODING	-0.38148	1.03E-17
ENSBTAT00000044762	ENSBTAG00000000111	UGP2	11	62244354	62299938	+	CODING	0.502749	1.11E-17
ENSBTAT00000004167	ENSBTAG00000003209	BLCAP	13	67114713	67125217	-	CODING	0.640531	1.14E-17
ENSBTAT00000019336	ENSBTAG00000014547	PGAM2	22	370972	373326	-	CODING	0.270879	1.19E-17
ENSBTAT00000000398	ENSBTAG00000000310	MFAP5	5	101647629	101659377	+	CODING	-1.1996	1.35E-17
ENSBTAT00000024387	ENSBTAG00000038488	TMSB4	X	140973702	140975767	-	CODING	-0.71921	3.46E-17
ENSBTAT00000020150	ENSBTAG00000015147	S100A10	3	18799612	18810545	+	CODING	-0.87935	4.69E-17
ENSBTAT00000019318	ENSBTAG00000014534	EEF1A1	9	13233554	13236949	-	CODING	-0.65326	1.28E-16
ENSBTAT00000043425	ENSBTAG00000019011	PGM1	3	82250292	82315999	-	CODING	0.322581	1.31E-16
ENSBTAT00000014306	ENSBTAG00000010799	MYL6	5	57486017	57489133	-	CODING	-0.81844	5.20E-16
ENSBTAT00000060566	ENSBTAG00000043560	COX3	MT	8970	9750	+	CODING	-0.04177	5.73E-16
ENSBTAT00000017905	ENSBTAG00000013461	RPL24	1	46415223	46420721	-	CODING	-0.39651	6.04E-16
ENSBTAT00000064497	ENSBTAG00000046100	-	26	14093095	14093609	+	CODING	-0.67205	9.94E-16
ENSBTAT00000006990	ENSBTAG00000005315	RPS6	2	111820296	111821112	-	CODING	-0.44006	1.46E-15
ENSBTAT00000017086	ENSBTAG00000012855	LPL	8	67481089	67511227	+	CODING	-1.46199	1.50E-15
ENSBTAT00000016717	ENSBTAG00000012594	MRPS6	1	669920	733729	-	CODING	-1.48835	1.80E-15
ENSBTAT00000017122	ENSBTAG00000012885	ACAT1	15	17999932	18028984	+	CODING	-0.76628	2.10E-15
ENSBTAT00000034744	ENSBTAG00000015406	ZNF750	19	50475196	50483968	+	CODING	1.858901	2.14E-15
ENSBTAT00000061127	ENSBTAG00000044105	FOXO1	12	21915747	22005338	+	CODING	1.22045	2.48E-15
ENSBTAT00000037900	ENSBTAG00000005085	TRIM63	2	127615050	127630608	+	CODING	-0.5519	2.56E-15
ENSBTAT00000020261	ENSBTAG00000015228	CD74	7	63748885	63756646	-	CODING	-0.66488	2.88E-15
ENSBTAT00000011521	ENSBTAG00000008743	ALDH2	17	64551612	64577901	-	CODING	0.66919	3.36E-15
ENSBTAT00000008132	ENSBTAG00000006189	ACTG1	19	51868429	51871276	+	CODING	-0.4669	6.40E-15
ENSBTAT00000000556	ENSBTAG00000000434	CRYAB	15	22566929	22570256	-	CODING	-0.09824	7.07E-15
ENSBTAT00000065106	ENSBTAG00000047752	OTUD1	13	24655214	24656659	+	CODING	-1.15904	1.16E-14
ENSBTAT00000025484	ENSBTAG00000019147	RPS20	14	24955079	24956324	-	CODING	-0.55028	2.15E-14

ENSBTAT00000005007	ENSBTAG00000005296	RPL13A	18	56394558	56398082	+	CODING	-0.3211	2.17E-14
ENSBTAT00000021587	ENSBTAG00000016224	RPS7	8	112896493	112901718	-	CODING	-0.38599	3.67E-14
ENSBTAT00000021033	ENSBTAG00000015831	RPL18A	7	5206112	5209504	-	CODING	-0.37015	4.75E-14
ENSBTAT00000002674	ENSBTAG00000002068	TAGLN2	3	9878839	9886647	+	CODING	-1.07327	5.13E-14
ENSBTAT00000013284	ENSBTAG00000010069	EGR1	7	51438727	51442500	+	CODING	-0.67687	5.76E-14
ENSBTAT00000023087	ENSBTAG00000017363	SAT1	X	126467442	126470489	-	CODING	-1.1713	6.50E-14
ENSBTAT00000024157	ENSBTAG00000018152	MYADM	18	62018419	62024004	+	CODING	-0.69447	6.62E-14
ENSBTAT00000060438	ENSBTAG00000043446	SNORA51	3	15691547	15691677	-	CODING	-5.64208	7.42E-14
ENSBTAT00000002326	ENSBTAG00000001777	RPLP2	29	50768654	50770791	-	CODING	-0.5048	8.58E-14
ENSBTAT00000040046	ENSBTAG00000027772	RPS25	15	30115928	30117641	-	CODING	-0.42101	8.93E-14
ENSBTAT00000020719	ENSBTAG00000015598	RPS10	23	8434576	8441522	-	CODING	-0.33151	1.20E-13
ENSBTAT00000063192	ENSBTAG00000046177	IGFN1	16	81604665	81641464	+	CODING	0.655893	1.91E-13
ENSBTAT00000045699	ENSBTAG00000032217	-	1	84324177	84325181	-	CODING	-1.09422	2.21E-13
ENSBTAT00000060533	ENSBTAG00000043541	SNORA5	4	77227804	77227938	+	CODING	-5.58423	2.31E-13
ENSBTAT00000059118	ENSBTAG00000042126	SNORA66	16	37582336	37582467	+	CODING	-5.56911	3.09E-13
ENSBTAT00000031470	ENSBTAG00000005718	PLIN2	8	25129168	25142901	+	CODING	-0.9038	3.55E-13
ENSBTAT00000028994	ENSBTAG00000021752	DNAJB4	3	66923000	66931737	-	CODING	-0.66493	3.59E-13
ENSBTAT00000021683	ENSBTAG00000016296	GPD1	5	29958501	29964085	-	CODING	0.404075	6.65E-13
ENSBTAT00000014304	ENSBTAG00000010799	MYL6	5	57486017	57489133	-	CODING	-0.56789	7.12E-13
ENSBTAT00000024663	ENSBTAG00000018530	TUBA8	5	109876442	109894993	+	CODING	0.551359	7.77E-13
ENSBTAT00000003305	ENSBTAG00000002549	PTMA	2	120227920	120232987	+	CODING	-0.73421	1.01E-12
ENSBTAT00000026278	ENSBTAG00000019718	RPS15	7	45465834	45467519	+	CODING	-0.35139	1.11E-12
ENSBTAT00000060554	ENSBTAG00000043553	GPX3	7	64286948	64295116	+	CODING	-0.79578	1.24E-12
ENSBTAT00000025270	ENSBTAG00000018987	RPS25	3	16507484	16507861	-	CODING	-0.41895	1.45E-12
ENSBTAT00000060546	ENSBTAG00000043567	-	MT	431	1385	+	CODING	-0.20149	1.87E-12
ENSBTAT00000023246	ENSBTAG00000017475	GNAS	13	58010287	58049012	-	CODING	-0.87533	2.11E-12
ENSBTAT00000012655	ENSBTAG00000009615	ANXA2	10	49860062	49904536	+	CODING	-0.91111	2.25E-12
ENSBTAT00000064506	ENSBTAG00000022158	TNNT3	29	50218484	50233948	-	CODING	-12.1514	2.74E-12
ENSBTAT00000057115	ENSBTAG00000019852	PDHA1	X	130874454	130897343	-	CODING	0.506751	2.79E-12
ENSBTAT00000000790	ENSBTAG00000000598	CST3	13	42562167	42566091	-	CODING	-0.37992	3.03E-12
ENSBTAT00000008319	ENSBTAG00000006342	ATPIF1	2	125727885	125730128	-	CODING	-0.34149	3.59E-12
ENSBTAT00000002400	ENSBTAG00000001836	-	19	19937602	19947884	-	CODING	-2.40782	5.41E-12
ENSBTAT00000035955	ENSBTAG00000015131	SLC29A1	23	17845969	17856150	+	CODING	-0.65722	5.76E-12
ENSBTAT00000005684	ENSBTAG00000004344	ACSL1	27	14223449	14288333	-	CODING	-0.52858	6.64E-12
ENSBTAT00000011265	ENSBTAG00000008545	ATF3	16	72820026	72832974	-	CODING	-0.85656	8.03E-12
ENSBTAT00000035337	ENSBTAG00000032954	ATP5E	22	35335190	35335581	+	CODING	-0.31979	8.69E-12
ENSBTAT00000005348	ENSBTAG00000004094	SPARCL1	6	104149824	104202396	-	CODING	-0.58735	9.26E-12
ENSBTAT00000015802	ENSBTAG00000011912	SLC25A25	11	98750459	98760803	+	CODING	1.011783	1.03E-11
ENSBTAT00000007504	ENSBTAG00000005714	ACTC1	10	30361781	30367052	-	CODING	0.962739	1.15E-11
ENSBTAT00000008967	ENSBTAG00000006823	CMYA5	10	10658029	10711354	+	CODING	0.313576	1.15E-11
ENSBTAT00000013008	ENSBTAG00000009863	BHLHE40	22	21492896	21498539	-	CODING	-0.65587	1.20E-11
ENSBTAT00000024555	ENSBTAG00000018451	PTMS	5	104039252	104040499	-	CODING	-0.69838	1.21E-11
ENSBTAT00000026002	ENSBTAG00000019521	COX6A2	25	27737582	27738205	-	CODING	-0.29774	1.62E-11
ENSBTAT00000015260	ENSBTAG00000011482	SLC43A2	19	23261580	23301008	-	CODING	0.934879	1.75E-11
ENSBTAT00000020148	ENSBTAG00000015145	S100A11	3	18768796	18770416	+	CODING	-0.79268	2.23E-11
ENSBTAT00000047320	ENSBTAG00000012044	RPL13	18	14533161	14535556	+	CODING	-0.46757	2.65E-11
ENSBTAT00000030071	ENSBTAG00000013240	SLC3A2	29	41855871	41896760	+	CODING	0.709173	2.98E-11
ENSBTAT00000019194	ENSBTAG00000014433	UBE2G1	19	25409138	25496726	-	CODING	0.386322	3.02E-11
ENSBTAT00000012317	ENSBTAG00000009359	-	15	38876122	38876667	-	CODING	-0.31557	3.12E-11
ENSBTAT00000054652	ENSBTAG00000001729	DUSP10	16	25895839	25936864	-	CODING	-2.06095	3.22E-11
ENSBTAT00000038879	ENSBTAG00000014069	PDK4	4	12754202	12767677	-	CODING	0.279936	3.83E-11
ENSBTAT00000059592	ENSBTAG00000042600	SNORD94	11	48600030	48600165	-	CODING	-5.292	4.03E-11
ENSBTAT00000019808	ENSBTAG00000014878	COX7A1	18	46994991	46996544	-	CODING	-0.39933	4.10E-11
ENSBTAT00000021068	ENSBTAG00000015848	PHKA1	X	83004459	83217747	+	CODING	0.5156	4.12E-11
ENSBTAT00000013778	ENSBTAG00000010880	TNNI2	29	50285049	50287648	-	CODING	-0.18948	4.61E-11
ENSBTAT00000050559	ENSBTAG00000036078	EMP1	5	97081921	97101600	-	CODING	-0.73775	5.41E-11
ENSBTAT00000028263	ENSBTAG00000021211	DPT	16	37106633	37143040	-	CODING	-1.2518	5.44E-11
ENSBTAT00000042757	ENSBTAG00000030281	-	11	104186563	104187141	-	CODING	-0.41932	6.29E-11
ENSBTAT00000004188	ENSBTAG00000003228	RPL3	5	111261438	111267705	-	CODING	-0.77331	7.79E-11
ENSBTAT00000020608	ENSBTAG00000015509	NAMPT	4	47597860	47635332	-	CODING	0.555132	8.13E-11
ENSBTAT00000007149	ENSBTAG00000005431	LMCD1	22	17961211	18018234	-	CODING	-0.77298	8.18E-11
ENSBTAT00000022835	ENSBTAG00000017183	PDLIM3	27	14769571	14800205	-	CODING	0.173239	8.69E-11

ENSBTAT00000065572	ENSBTAG00000045568	-	24	13077339	13078261	+	CODING	-0.4041	9.36E-11
ENSBTAT00000021330	ENSBTAG00000016026	PCOLCE2	1	127132777	127229651	+	CODING	-1.60703	1.03E-10
ENSBTAT00000055086	ENSBTAG00000017389	RPLP0	17	64809254	64813289	-	CODING	-0.3272	1.04E-10
ENSBTAT00000042874	ENSBTAG00000008570	RPS14	7	63761742	63779251	-	CODING	-0.41064	1.13E-10
ENSBTAT00000004658	ENSBTAG00000003581	SETD7	17	18463314	18515964	+	CODING	0.494918	1.13E-10
ENSBTAT00000017372	ENSBTAG00000013066	IGF2	29	50046626	50065230	+	CODING	-0.79862	1.18E-10
ENSBTAT00000018778	ENSBTAG00000014130	COX6C	14	66637801	66647721	+	CODING	-0.40802	1.75E-10
ENSBTAT00000016346	ENSBTAG00000012317	PNP	10	26667693	26673918	-	CODING	-0.57424	1.75E-10
ENSBTAT00000000187	ENSBTAG00000000163	DDIT4	28	28483403	28485410	+	CODING	0.467378	1.95E-10
ENSBTAT00000011795	ENSBTAG00000020116	JSP.1	23	28469735	28473401	-	CODING	-0.60317	2.96E-10
ENSBTAT00000027478	ENSBTAG00000020620	-	4	58724265	58724900	-	CODING	1.120132	3.20E-10
ENSBTAT00000021651	ENSBTAG00000016278	RPL30	14	68467563	68470952	+	CODING	-0.37323	3.22E-10
ENSBTAT00000010176	ENSBTAG00000007737	UBA52	7	4535467	4537851	-	CODING	-0.2246	3.27E-10
ENSBTAT00000021199	ENSBTAG00000015942	DNAJA4	21	31177694	31192961	+	CODING	-1.02419	3.83E-10
ENSBTAT00000020512	ENSBTAG00000015434	DSTN	13	38259752	38284536	+	CODING	-0.85611	3.83E-10
ENSBTAT00000048100	ENSBTAG00000025258	-	19	14514200	14548145	-	CODING	-1.4967	4.27E-10
ENSBTAT00000007324	ENSBTAG00000005574	CLU	8	11043941	11061301	+	CODING	-0.75017	4.52E-10
ENSBTAT00000066175	ENSBTAG00000048167	U4	17	64874908	64875048	-	CODING	-5.13369	4.60E-10
ENSBTAT00000027927	ENSBTAG00000020968	MPC2	3	768307	799012	+	CODING	-0.68272	4.90E-10
ENSBTAT00000011863	ENSBTAG00000009012	PTX3	1	111027804	111033868	-	CODING	-3.63342	5.51E-10
ENSBTAT00000013301	ENSBTAG00000027075	-	21	14304505	14304970	+	CODING	-0.34611	6.01E-10
ENSBTAT00000008851	ENSBTAG00000006733	PPP1R3A	4	54866421	54906096	+	CODING	0.359702	6.31E-10
ENSBTAT00000045066	ENSBTAG00000031786	FAU	5	47736728	47737193	+	CODING	-0.3437	6.88E-10
ENSBTAT00000020125	ENSBTAG00000015127	SDC4	13	74393120	74412880	-	CODING	-1.09976	8.71E-10
ENSBTAT00000015579	ENSBTAG00000011730	TCAP	19	40691138	40692346	+	CODING	-0.12716	9.06E-10
ENSBTAT00000018888	ENSBTAG00000014208	RPL35A	1	70788697	70792140	-	CODING	-0.59271	9.27E-10
ENSBTAT00000027091	ENSBTAG00000020330	BTG1	5	22086071	22088781	-	CODING	-0.58889	1.14E-09
ENSBTAT00000036603	ENSBTAG00000012866	THBS4	10	10945425	10999244	+	CODING	-0.73017	1.49E-09
ENSBTAT00000006866	ENSBTAG00000005211	RPL4	10	13318875	13323526	-	CODING	-0.27807	1.53E-09
ENSBTAT00000003431	ENSBTAG00000002648	RPS18	23	7388703	7393361	+	CODING	-0.30618	2.85E-09
ENSBTAT00000021397	ENSBTAG00000016079	COX4I1	18	11800134	11807341	+	CODING	-0.27464	2.86E-09
ENSBTAT00000004411	ENSBTAG00000003403	PADI2	2	136049644	136103406	+	CODING	1.404001	2.90E-09
ENSBTAT00000056240	ENSBTAG00000038849	TRDN	9	28071827	28219689	+	CODING	0.336638	3.27E-09
ENSBTAT00000023581	ENSBTAG00000017733	CA2	14	79372712	79388600	-	CODING	1.044688	3.41E-09
ENSBTAT00000059955	ENSBTAG00000042963	SNORA8	29	1065601	1065737	+	CODING	-4.99048	3.42E-09
ENSBTAT00000026100	ENSBTAG00000019588	BOLA-DQB	23	25855146	25863045	-	CODING	-2.05397	4.03E-09
ENSBTAT00000024417	ENSBTAG00000018352	ABRA	14	59974386	59985200	+	CODING	-0.86564	4.03E-09
ENSBTAT00000017580	ENSBTAG00000013208	SLC25A4	27	14546020	14550037	+	CODING	-0.1519	4.45E-09
ENSBTAT00000002092	ENSBTAG00000038067	MT1A	18	24106722	24108521	-	CODING	-2.81749	4.54E-09
ENSBTAT00000055747	ENSBTAG00000038079	C16H1ORF21	16	66960790	67142414	+	CODING	-0.61658	4.87E-09
ENSBTAT00000024788	ENSBTAG00000018628	RPL29	22	49499798	49501972	+	CODING	-0.35177	4.93E-09
ENSBTAT00000011677	ENSBTAG00000008868	CAPN3	10	37828797	37885860	+	CODING	0.408655	5.19E-09
ENSBTAT00000024583	ENSBTAG00000018471	SSR3	1	111996968	112008219	+	CODING	-0.63572	5.34E-09
ENSBTAT00000005403	ENSBTAG00000004126	MLF1	1	109763292	109793739	-	CODING	0.390299	6.28E-09
ENSBTAT00000017420	ENSBTAG00000013103	COL1A1	19	37088246	37104998	+	CODING	-1.14578	6.40E-09
ENSBTAT00000012786	ENSBTAG00000009696	ACTN2	28	9403202	9450916	+	CODING	-0.20908	6.95E-09
ENSBTAT00000042753	ENSBTAG00000030278	EGFL7	11	104130864	104131233	+	CODING	-0.62867	7.67E-09
ENSBTAT00000060287	ENSBTAG00000043295	SNORA13	10	1763369	1763505	-	CODING	-4.92783	7.81E-09
ENSBTAT00000064987	ENSBTAG00000046587	-	29	44770865	44771529	-	CODING	1.283378	8.05E-09
ENSBTAT00000026407	ENSBTAG00000019822	TPPP3	18	35121243	35124820	-	CODING	-0.78334	8.30E-09
ENSBTAT00000016279	ENSBTAG00000012276	RPL5	13	38980088	38981117	-	CODING	-0.40098	8.46E-09
ENSBTAT00000054382	ENSBTAG00000008636	PDE4B	3	79284893	79304824	-	CODING	1.024719	8.51E-09
ENSBTAT00000038612	ENSBTAG00000026972	MYF5	5	10339425	10342660	+	CODING	-1.96249	9.70E-09
ENSBTAT00000005279	ENSBTAG00000004037	JUN	3	87841042	87843087	+	CODING	-0.42449	1.02E-08
ENSBTAT00000000814	ENSBTAG00000000622	RPS17	21	23301663	23305104	-	CODING	-0.18919	1.14E-08
ENSBTAT00000059254	ENSBTAG00000046842	U4	17	64875706	64875846	-	CODING	-4.89273	1.22E-08
ENSBTAT00000010660	ENSBTAG00000008105	RBM38	13	59294500	59308857	-	CODING	0.552709	1.31E-08
ENSBTAT00000032193	ENSBTAG00000016341	RGS5	3	6228349	6426528	+	CODING	-0.56152	1.58E-08
ENSBTAT00000022919	ENSBTAG00000017246	UBB	19	33853788	33855685	-	CODING	-0.29129	1.60E-08
ENSBTAT00000012599	ENSBTAG00000009580	SH3BGRL3	2	127429585	127431204	-	CODING	-0.7016	1.64E-08
ENSBTAT00000060540	ENSBTAG00000043570	-	MT	1453	3023	+	CODING	-0.15106	1.83E-08
ENSBTAT00000065888	ENSBTAG00000047299	DGCR6L	17	74049772	74053881	+	CODING	-1.02021	1.90E-08

ENSBTAT00000028602	ENSBTAG00000021455	CFL1	29	44638896	44642280	-	CODING	-0.63968	2.08E-08
ENSBTAT00000028639	ENSBTAG00000021487	CIART	3	20400969	20404292	-	CODING	0.805184	2.09E-08
ENSBTAT00000029208	ENSBTAG00000014614	ACTA2	26	10662363	10679648	-	CODING	-0.52029	2.21E-08
ENSBTAT00000011047	ENSBTAG00000008394	MYL3	22	53202766	53208551	+	CODING	0.242443	2.22E-08
ENSBTAT00000035335	ENSBTAG00000012849	COL4A1	12	88876125	89009422	-	CODING	-0.79671	2.23E-08
ENSBTAT00000026928	ENSBTAG00000020219	MSS51	28	29578510	29588920	-	CODING	-0.85674	2.24E-08
ENSBTAT00000022378	ENSBTAG00000016822	PPIB	10	45874978	45880918	+	CODING	-0.84791	2.44E-08
ENSBTAT00000003962	ENSBTAG00000005620	RPS3	15	55370367	55375306	+	CODING	-0.24294	2.49E-08
ENSBTAT00000060548	ENSBTAG00000043571	ND2	MT	4266	5307	+	CODING	-0.05538	2.57E-08
ENSBTAT00000006810	ENSBTAG00000005165	CSGALNACT2	28	13651048	13678154	+	CODING	1.08055	2.87E-08
ENSBTAT00000025691	ENSBTAG00000019295	MDH1	11	61970467	61994479	+	CODING	-0.39184	2.89E-08
ENSBTAT00000008502	ENSBTAG00000006487	RPS9	18	63381416	63388728	-	CODING	-0.37855	2.93E-08
ENSBTAT00000060896	ENSBTAG00000043904	SNORA57	4	43820203	43820347	+	CODING	-4.81802	3.07E-08
ENSBTAT00000015668	ENSBTAG00000011802	COL6A1	1	147404396	147423644	+	CODING	-0.80307	3.16E-08
ENSBTAT00000017175	ENSBTAG00000012926	TM6SF1	21	25394313	25424181	-	CODING	-0.5634	3.16E-08
ENSBTAT00000028280	ENSBTAG00000021226	SAR1B	7	47717606	47747828	-	CODING	0.420408	3.17E-08
ENSBTAT00000010166	ENSBTAG00000027610	RPL36A	15	54468007	54468423	+	CODING	-0.29353	3.73E-08
ENSBTAT00000001838	ENSBTAG00000001398	ATP2A2	17	56458750	56512895	+	CODING	-0.21442	3.78E-08
ENSBTAT00000063883	ENSBTAG00000045728	SCD	26	21141592	21148318	-	CODING	-0.78977	3.84E-08
ENSBTAT00000013079	ENSBTAG00000009908	RPS3A	17	6703060	6707745	-	CODING	-0.27391	4.19E-08
ENSBTAT00000007124	ENSBTAG00000005414	-	1	51022816	51023423	+	CODING	-1.07719	4.29E-08
ENSBTAT00000026197	ENSBTAG00000019658	ASB16	19	44614052	44621587	+	CODING	0.465415	4.49E-08
ENSBTAT00000024391	ENSBTAG00000018331	CLEC3B	22	54791195	54797301	-	CODING	-0.8566	4.50E-08
ENSBTAT00000046532	ENSBTAG00000032764	-	6	323371	323914	-	CODING	-0.39348	4.52E-08
ENSBTAT00000016359	ENSBTAG00000012330	B2M	10	104139090	104145312	+	CODING	-0.1724	4.66E-08
ENSBTAT00000026056	ENSBTAG00000019554	FBP2	8	82396095	82438817	-	CODING	0.256743	5.54E-08
ENSBTAT00000012866	ENSBTAG00000009757	RPSA	22	12729703	12741653	+	CODING	-0.3647	5.77E-08
ENSBTAT00000005143	ENSBTAG00000003937	-	13	22813811	22814335	+	CODING	-0.28895	5.90E-08
ENSBTAT00000051159	ENSBTAG00000036659	U2	29	41846574	41846764	-	CODING	-4.75375	6.55E-08
ENSBTAT00000023213	ENSBTAG00000017462	ATF4	5	111462845	111464936	+	CODING	0.130971	6.93E-08
ENSBTAT00000054271	ENSBTAG00000009707	MYL1	2	98527573	98555234	-	CODING	0.102164	7.20E-08
ENSBTAT00000037254	ENSBTAG00000026266	MYL12B	24	37834593	37910130	+	CODING	-1.11383	7.27E-08
ENSBTAT00000004161	ENSBTAG00000003205	RPL35	11	95850116	95854734	-	CODING	-0.44623	7.92E-08
ENSBTAT00000055220	ENSBTAG00000033008	MYOZ1	28	29780787	29798613	-	CODING	0.132054	8.51E-08
ENSBTAT00000061251	ENSBTAG00000039684	PTRF	19	43148013	43162165	-	CODING	-0.4431	8.52E-08
ENSBTAT00000029304	ENSBTAG00000021979	EEF1B	2	94922839	94926122	+	CODING	-0.36554	9.70E-08
ENSBTAT00000040201	ENSBTAG00000027879	MPC1	9	102880277	102893601	-	CODING	-0.41705	9.98E-08
ENSBTAT00000028615	ENSBTAG00000021467	IGFBP6	5	27044009	27047853	-	CODING	-1.00121	1.09E-07
ENSBTAT00000054439	ENSBTAG00000022314	EMD	X	40341365	40343613	+	CODING	2.140714	1.13E-07
ENSBTAT00000014215	ENSBTAG00000010738	CCL14	19	14775262	14780081	+	CODING	-0.64652	1.13E-07
ENSBTAT00000033863	ENSBTAG00000013472	COL1A2	4	11624470	11661163	+	CODING	-0.95377	1.19E-07
ENSBTAT00000034789	ENSBTAG00000019903	RAMP2	19	43441583	43443429	+	CODING	-0.59132	1.21E-07
ENSBTAT00000037367	ENSBTAG00000026327	RPL8	14	1505030	1507633	-	CODING	-0.39208	1.22E-07
ENSBTAT00000015248	ENSBTAG00000011473	MYL9	13	66306260	66314230	+	CODING	-0.76932	1.29E-07
ENSBTAT00000020709	ENSBTAG00000015591	SQSTM1	7	1334566	1346051	-	CODING	0.298776	1.32E-07
ENSBTAT00000028016	ENSBTAG00000021035	CTSK	3	19994998	20007861	+	CODING	-1.1547	1.47E-07
ENSBTAT00000019753	ENSBTAG00000014831	PPP1R3C	26	13270026	13274922	-	CODING	-0.69223	1.47E-07
ENSBTAT00000063866	ENSBTAG00000045504	-	9	42351250	42352038	-	CODING	-0.41249	1.56E-07
ENSBTAT00000047585	ENSBTAG00000002092	PI16	23	10836838	10848743	+	CODING	-1.8081	1.64E-07
ENSBTAT00000019643	ENSBTAG00000014764	CD9	5	104505691	104518399	-	CODING	-0.98365	1.72E-07
ENSBTAT00000021369	ENSBTAG00000016057	CSRP1	16	49332770	49353517	-	CODING	-0.82996	2.02E-07
ENSBTAT00000023622	ENSBTAG00000031788	GSTM1	3	33782528	33800900	-	CODING	0.517035	2.10E-07
ENSBTAT00000018998	ENSBTAG00000014299	RHOC	3	30769966	30776469	+	CODING	-0.98844	2.20E-07
ENSBTAT00000060549	ENSBTAG00000043556	COII	MT	7374	8057	+	CODING	-0.02964	2.59E-07
ENSBTAT00000009302	ENSBTAG00000007415	SLC7A8	10	21521555	21573888	+	CODING	0.402181	2.61E-07
ENSBTAT00000012495	ENSBTAG00000009495	BCAM	18	52965138	52976445	+	CODING	-0.87843	2.64E-07
ENSBTAT00000063321	ENSBTAG00000046121	-	14	79340383	79345452	+	CODING	-1.65669	2.68E-07
ENSBTAT00000020561	ENSBTAG00000015470	SYPL2	3	34082543	34095369	-	CODING	0.408249	2.71E-07
ENSBTAT00000023751	ENSBTAG00000017869	CAV1	4	52173110	52208687	-	CODING	-0.37251	2.77E-07
ENSBTAT00000011824	ENSBTAG00000008985	PCBP1	11	68383793	68385266	+	CODING	0.309802	2.88E-07
ENSBTAT00000000973	ENSBTAG00000000731	WFIKKN2	19	36536565	36542420	-	CODING	1.628277	3.00E-07
ENSBTAT00000020520	ENSBTAG00000015441	ACTB	11	10717554	10732398	-	CODING	-1.53954	3.03E-07

ENSBTAT00000016884	ENSBTAG00000012703	GLO1	23	12483468	12509232	-	CODING	0.394816	3.20E-07
ENSBTAT00000049790	ENSBTAG00000013479	SLC9A3R2	25	1581874	1589619	+	CODING	-1.06776	3.25E-07
ENSBTAT00000018099	ENSBTAG00000013614	TMEM38A	7	6285842	6312400	-	CODING	0.429066	3.30E-07
ENSBTAT00000028221	ENSBTAG00000021176	CRISPLD2	18	10985132	11050904	+	CODING	-1.01704	3.34E-07
ENSBTAT00000017770	ENSBTAG00000013358	RPS23	7	84523780	84525639	-	CODING	-0.714	3.34E-07
ENSBTAT00000029891	ENSBTAG00000022155	FSTL1	1	65742633	65802423	-	CODING	-0.87283	3.46E-07
ENSBTAT00000049004	ENSBTAG00000017753	APP	1	9607382	9921004	+	CODING	-0.58886	3.52E-07
ENSBTAT00000030328	ENSBTAG00000022396	SAA3	29	26668047	26671801	-	CODING	-5.41572	3.76E-07
ENSBTAT00000025716	ENSBTAG00000019314	USP25	1	20664990	20798853	-	CODING	0.479969	3.81E-07
ENSBTAT00000057593	ENSBTAG00000019517	ELN	25	33787889	33820672	-	CODING	-1.5148	3.96E-07
ENSBTAT00000001121	ENSBTAG00000000843	NDRG2	10	26134842	26142255	+	CODING	0.310802	3.98E-07
ENSBTAT00000007369	ENSBTAG00000023487	COX6B1	5	94363786	94364264	+	CODING	-0.32993	4.42E-07
ENSBTAT00000005982	ENSBTAG00000004553	TPM4	7	7923143	7948265	-	CODING	-0.67339	4.66E-07
ENSBTAT00000054608	ENSBTAG00000046664	TSC22D1	12	14843776	14846181	-	CODING	0.689963	4.68E-07
ENSBTAT00000032006	ENSBTAG00000023471	RPL36	5	28525103	28525496	+	CODING	-0.45622	4.79E-07
ENSBTAT00000009484	ENSBTAG00000007211	ASB12	X	101491186	101492856	+	CODING	0.272036	5.07E-07
ENSBTAT00000010711	ENSBTAG00000008150	PKIA	14	43880469	43978277	+	CODING	-0.33295	5.52E-07
ENSBTAT00000063682	ENSBTAG00000047418	SLC25A6	GJ06	1867	6353	+	CODING	-0.67817	5.91E-07
ENSBTAT00000015829	ENSBTAG00000011931	CD63	5	57854278	57857485	+	CODING	-0.29431	6.53E-07
ENSBTAT00000060567	ENSBTAG00000043550	MT-CYB	MT	14514	15653	+	CODING	-0.03757	6.79E-07
ENSBTAT00000014390	ENSBTAG00000010843	PGRMC2	17	29872406	29890867	+	CODING	0.447487	7.29E-07
ENSBTAT00000044139	ENSBTAG00000013066	IGF2	29	50058063	50062631	+	CODING	-1.25439	8.25E-07
ENSBTAT00000000993	ENSBTAG00000000745	AQP1	4	65830992	65845186	-	CODING	-0.84268	8.37E-07
ENSBTAT00000003943	ENSBTAG00000003033	GADD45G	8	90017453	90018996	-	CODING	-0.64559	1.02E-06
ENSBTAT00000028239	ENSBTAG00000021191	EHD2	18	55071102	55087454	+	CODING	-0.54493	1.12E-06
ENSBTAT00000016712	ENSBTAG00000012589	HSPE1	2	86449525	86451564	+	CODING	-0.31324	1.19E-06
ENSBTAT00000010279	ENSBTAG00000007816	-	6	87555288	87556157	+	CODING	-0.70746	1.27E-06
ENSBTAT00000022636	ENSBTAG00000017024	PPARGC1A	6	44854113	44960533	-	CODING	-1.47873	1.29E-06
ENSBTAT00000016378	ENSBTAG00000012344	RPL26	19	28590314	28595213	-	CODING	-0.4928	1.31E-06
ENSBTAT00000046341	ENSBTAG00000006101	PSMD4	3	19598474	19607504	-	CODING	0.606086	1.32E-06
ENSBTAT00000021482	ENSBTAG00000016139	TMEM120A	25	34726229	34731680	-	CODING	-1.20238	1.33E-06
ENSBTAT00000016865	ENSBTAG00000001842	GSTM3	3	33768050	33770824	+	CODING	0.657531	1.42E-06
ENSBTAT00000006167	ENSBTAG00000018137	A2M	5	101298127	101346611	+	CODING	-0.86888	1.43E-06
ENSBTAT00000063725	ENSBTAG00000045544	-	X	144159056	144234222	-	CODING	-0.98265	1.54E-06
ENSBTAT00000047242	ENSBTAG00000005250	BGN	X	39639906	39653687	+	CODING	-1.24209	1.58E-06
ENSBTAT00000002279	ENSBTAG00000001745	LUM	5	21037443	21044658	-	CODING	-0.97341	1.66E-06
ENSBTAT00000015358	ENSBTAG00000011559	RPL7A	11	104311808	104315125	+	CODING	-0.31648	1.76E-06
ENSBTAT00000023912	ENSBTAG00000017970	ZYX	4	107598856	107607834	+	CODING	-0.67131	1.85E-06
ENSBTAT00000012351	ENSBTAG00000009387	MYOM2	27	312538	376193	+	CODING	0.35019	1.90E-06
ENSBTAT00000036848	ENSBTAG00000007068	SH3BGR	1	141029893	141100984	+	CODING	-0.22219	2.06E-06
ENSBTAT00000047197	ENSBTAG00000033217	TPM3	3	16382706	16403812	+	CODING	-0.70098	2.07E-06
ENSBTAT00000029418	ENSBTAG00000022020	CLDN5	17	74749250	74750524	-	CODING	-0.96624	2.11E-06
ENSBTAT00000025311	ENSBTAG00000019015	IFITM3	29	51367009	51368065	+	CODING	-0.72025	2.19E-06
ENSBTAT00000029468	ENSBTAG00000022032	CHCHD10	17	73206771	73208629	-	CODING	-0.27678	2.20E-06
ENSBTAT00000017010	ENSBTAG00000012805	TSPAN13	4	25213088	25251241	+	CODING	-0.60454	2.42E-06
ENSBTAT00000014642	ENSBTAG00000011022	ARPP19	10	57932635	57938671	+	CODING	-0.43857	3.03E-06
ENSBTAT00000062472	ENSBTAG00000045039	SCARNA17	24	49943458	49943600	+	CODING	1.594988	3.10E-06
ENSBTAT00000010682	ENSBTAG00000008122	GNG5	3	59783839	59791977	+	CODING	-0.46146	3.10E-06
ENSBTAT00000010270	ENSBTAG00000007807	-	10	76992202	76994112	+	CODING	-1.55297	3.18E-06
ENSBTAT00000015285	ENSBTAG00000011500	CASQ2	3	27658862	27729416	+	CODING	-1.41657	3.30E-06
ENSBTAT00000023627	ENSBTAG00000017765	GSTM1	3	33805754	33816442	-	CODING	0.510482	3.40E-06
ENSBTAT00000012144	ENSBTAG00000009214	ETS2	1	152880454	152890252	+	CODING	0.648362	3.42E-06
ENSBTAT00000019184	ENSBTAG00000014423	-	16	47992813	48000514	+	CODING	-0.26575	3.54E-06
ENSBTAT00000011066	ENSBTAG00000008409	MYC	14	13769244	13774438	-	CODING	-1.22942	3.57E-06
ENSBTAT00000016040	ENSBTAG00000012088	FBLN1	5	116616200	116695692	+	CODING	-1.22396	3.66E-06
ENSBTAT00000013292	ENSBTAG00000010082	COL15A1	8	64437276	64540739	+	CODING	-1.0601	3.70E-06
ENSBTAT00000008422	ENSBTAG00000022158	TNNT3	29	50218484	50233948	-	CODING	0.673833	3.72E-06
ENSBTAT00000013713	ENSBTAG00000010389	FAM47E-STBI	6	92967767	92971371	+	CODING	0.454036	4.02E-06
ENSBTAT00000005600	ENSBTAG00000004279	RHOA	22	51277867	51323093	+	CODING	-0.36552	4.02E-06
ENSBTAT00000043753	ENSBTAG00000002326	LGALS3	10	67843328	67861113	+	CODING	-1.2742	4.10E-06
ENSBTAT00000060543	ENSBTAG00000043559	MT-ND4L	MT	10239	10535	+	CODING	0.044013	4.15E-06
ENSBTAT00000056173	ENSBTAG00000022396	SAA3	29	26668125	26671801	-	CODING	-2.97004	4.34E-06

ENSBTAT00000013559	ENSBTAG00000010265	MGST3	3	3296282	3319165	-	CODING	-0.3949	4.42E-06
ENSBTAT00000064762	ENSBTAG00000045604	TTC9	10	82634715	82666926	+	CODING	0.481771	4.46E-06
ENSBTAT00000025963	ENSBTAG00000019494	RPL10A	23	9391523	9394013	+	CODING	-0.36424	4.82E-06
ENSBTAT00000000596	ENSBTAG00000000469	PPP2CA	7	47425980	47450747	-	CODING	-0.43304	5.23E-06
ENSBTAT00000020002	ENSBTAG00000015025	YAF2	5	38674059	38753871	+	CODING	3.547763	5.36E-06
ENSBTAT00000020036	ENSBTAG00000015053	CFL2	21	45519654	45522305	-	CODING	-0.19336	5.53E-06
ENSBTAT00000002055	ENSBTAG00000001575	-	11	29424996	29432052	-	CODING	0.269642	5.70E-06
ENSBTAT00000009797	ENSBTAG00000007447	NUDT4	5	23348068	23364470	+	CODING	-0.56143	6.09E-06
ENSBTAT00000002160	ENSBTAG00000001648	RPL21	12	32852826	32859542	-	CODING	-0.32207	6.32E-06
ENSBTAT00000037397	ENSBTAG00000026344	MAFA	14	2426857	2427912	+	CODING	0.881625	6.50E-06
ENSBTAT00000025657	ENSBTAG00000019267	MMP2	18	23828638	23855657	+	CODING	-1.0103	6.67E-06
ENSBTAT00000020904	ENSBTAG00000015743	GMPR	23	40616330	40677006	-	CODING	0.387041	7.18E-06
ENSBTAT00000009208	ENSBTAG00000006995	SPTBN1	11	37030009	37241384	+	CODING	-0.51141	7.23E-06
ENSBTAT00000021638	ENSBTAG00000016267	SERPING1	15	82159474	82172143	+	CODING	-0.85486	7.33E-06
ENSBTAT00000035803	ENSBTAG00000025462	GADD45B	7	22411968	22414079	-	CODING	-0.66673	7.39E-06
ENSBTAT00000063200	ENSBTAG00000046117	TMSB4X	11	63290422	63395507	+	CODING	-0.69922	7.60E-06
ENSBTAT00000064053	ENSBTAG00000047957	SCD	26	21132751	21133969	+	CODING	-0.76449	7.72E-06
ENSBTAT00000052276	ENSBTAG00000038379	-	X	39983832	39984337	+	CODING	-0.37444	7.94E-06
ENSBTAT00000013671	ENSBTAG00000010356	RAB12	24	41532521	41541737	+	CODING	0.253862	8.20E-06
ENSBTAT00000025663	ENSBTAG00000019269	COL6A2	1	147542825	147572424	+	CODING	-1.04114	8.29E-06
ENSBTAT00000009228	ENSBTAG00000006999	RYR1	18	48502352	48631056	+	CODING	0.280308	8.39E-06
ENSBTAT00000011846	ENSBTAG00000046303	-	11	98509474	98513781	+	CODING	-0.43696	9.62E-06
ENSBTAT00000053783	ENSBTAG00000009302	RCAN2	23	19437663	19667184	-	CODING	-1.15893	9.64E-06
ENSBTAT00000045455	ENSBTAG00000010153	ANXA3	6	95065256	95136945	+	CODING	-0.66072	9.72E-06
ENSBTAT00000005015	ENSBTAG00000003846	RPL37A	2	105202277	105204896	+	CODING	-0.22982	1.01E-05
ENSBTAT00000028988	ENSBTAG00000021746	ANXA5	6	3542635	3575330	+	CODING	-0.67256	1.02E-05
ENSBTAT00000014072	ENSBTAG00000010645	BOLA-DRA	23	25587526	25592227	-	CODING	-0.5997	1.05E-05
ENSBTAT00000016154	ENSBTAG00000012178	NR1D1	19	41040926	41048228	-	CODING	-0.82075	1.06E-05
ENSBTAT00000012827	ENSBTAG00000009725	AOX1	2	89517811	89589090	+	CODING	-0.9723	1.09E-05
ENSBTAT00000031490	ENSBTAG00000023147	LINGO1	21	33292504	33294348	-	CODING	0.52291	1.10E-05
ENSBTAT00000065672	ENSBTAG00000038430	-	23	34219298	34219825	-	CODING	-0.4531	1.23E-05
ENSBTAT00000033787	ENSBTAG00000001908	DLD	4	49230967	49257713	+	CODING	0.356265	1.23E-05
ENSBTAT00000003801	ENSBTAG00000023039	-	28	31044244	31048603	-	CODING	0.515213	1.27E-05
ENSBTAT00000046887	ENSBTAG00000000507	NR4A1	5	27977007	27985786	-	CODING	0.530487	1.28E-05
ENSBTAT00000054061	ENSBTAG00000002624	PSTPIP2	24	46196113	46290465	-	CODING	0.461131	1.32E-05
ENSBTAT00000009285	ENSBTAG00000007062	IGFBP5	2	105378991	105397646	-	CODING	0.971338	1.33E-05
ENSBTAT00000065774	ENSBTAG00000046712	PSMC2	4	44806721	44819866	+	CODING	0.351497	1.42E-05
ENSBTAT00000023209	ENSBTAG00000017461	SLC16A3	19	51243972	51255385	-	CODING	1.041588	1.46E-05
ENSBTAT00000005076	ENSBTAG00000003889	PER1	19	28390594	28399600	-	CODING	0.659276	1.52E-05
ENSBTAT00000010884	ENSBTAG00000008271	MEDAG	12	30021743	30039901	-	CODING	-1.33918	1.55E-05
ENSBTAT00000059908	ENSBTAG00000042916	SNORD15	15	55374442	55374585	+	CODING	-4.18716	1.60E-05
ENSBTAT00000048797	ENSBTAG00000034449	GNG11	3	5454661	5455256	+	CODING	-0.83179	1.62E-05
ENSBTAT00000005998	ENSBTAG00000004564	MBNL1	1	116238831	116394390	-	CODING	0.387391	1.62E-05
ENSBTAT00000019411	ENSBTAG00000014583	CALM	18	54169313	54178928	+	CODING	0.202277	1.66E-05
ENSBTAT00000054479	ENSBTAG00000010954	ART3	6	92607226	92755605	+	CODING	0.530752	1.69E-05
ENSBTAT00000065463	ENSBTAG00000014448	-	25	28153082	28157501	+	CODING	4.263681	1.69E-05
ENSBTAT00000040333	ENSBTAG00000027962	-	2	112675322	112675995	+	CODING	-0.36251	1.72E-05
ENSBTAT00000011504	ENSBTAG00000008732	ZC3H12C	15	20489130	20526349	+	CODING	2.118179	1.77E-05
ENSBTAT00000016071	ENSBTAG00000012120	TIPARP	1	111830980	111853985	-	CODING	-1.16545	1.77E-05
ENSBTAT00000019803	ENSBTAG00000014872	CAPNS1	18	46987527	46994449	+	CODING	-0.28487	1.82E-05
ENSBTAT00000027504	ENSBTAG00000020638	TIMP3	5	71751415	71809052	+	CODING	-0.37722	1.95E-05
ENSBTAT00000020860	ENSBTAG00000015711	BTG2	16	890072	892370	+	CODING	-0.74948	2.01E-05
ENSBTAT00000044563	ENSBTAG00000017816	FXYD1	18	46053083	46057378	+	CODING	-0.40063	2.12E-05
ENSBTAT00000060242	ENSBTAG00000043250	7SK	23	24977642	24977972	+	CODING	-0.79111	2.45E-05
ENSBTAT00000055832	ENSBTAG00000006563	KBTBD5	22	15444466	15451791	+	CODING	-0.43456	2.52E-05
ENSBTAT00000035177	ENSBTAG00000025136	MYOZ3	7	64031684	64048678	+	CODING	0.528982	2.62E-05
ENSBTAT00000034732	ENSBTAG00000024929	PPP1R27	19	51658342	51659508	+	CODING	0.136958	2.92E-05
ENSBTAT00000002944	ENSBTAG00000002278	FBN1	10	61877808	62142171	+	CODING	-1.28684	3.05E-05
ENSBTAT00000008216	ENSBTAG00000006262	LIMS2	2	4780176	4819192	+	CODING	-0.86205	3.07E-05
ENSBTAT00000048021	ENSBTAG00000033835	MPZ	3	8229236	8234089	+	CODING	-0.74796	3.20E-05
ENSBTAT00000028282	ENSBTAG00000021227	ATP5H	19	57020084	57024849	+	CODING	-0.19618	3.31E-05
ENSBTAT00000015716	ENSBTAG00000011843	EEF1G	29	41614571	41624167	-	CODING	-0.23173	3.32E-05

ENSBTAT00000017995	ENSBTAG00000013533	CLIC1	23	27393342	27398934	+	CODING	-0.9573	3.44E-05
ENSBTAT00000028574	ENSBTAG00000021435	MAFF	5	110475770	110484826	+	CODING	-0.62585	3.50E-05
ENSBTAT00000005180	ENSBTAG00000003956	SGCA	19	37113477	37122359	-	CODING	0.385161	3.51E-05
ENSBTAT00000002600	ENSBTAG00000002006	THBS1	10	35314025	35329297	+	CODING	-0.91508	3.57E-05
ENSBTAT00000061511	ENSBTAG00000044192	MAF	18	6551586	6552719	-	CODING	0.934908	3.61E-05
ENSBTAT00000040033	ENSBTAG00000027766	C1QTNF5	15	30448399	30450337	-	CODING	-1.82496	3.68E-05
ENSBTAT00000063903	ENSBTAG00000047229	CRIP1	21	71390600	71392110	-	CODING	-0.85949	3.72E-05
ENSBTAT00000007616	ENSBTAG00000005793	PEA15	3	9578375	9588605	-	CODING	-0.51465	3.81E-05
ENSBTAT00000014026	ENSBTAG00000010610	DDIT4L	6	25889906	25894862	+	CODING	-0.31545	3.82E-05
ENSBTAT00000001007	ENSBTAG00000000759	NENF	16	73013321	73019895	+	CODING	-0.85714	3.89E-05
ENSBTAT00000006658	ENSBTAG00000005048	DHRS7C	19	29581010	29594334	-	CODING	-0.34486	3.90E-05
ENSBTAT00000024514	ENSBTAG00000018423	DDX5	19	49330954	49337523	-	CODING	-0.38017	4.25E-05
ENSBTAT00000063425	ENSBTAG00000018513	FHL1	X	19777799	19820952	+	CODING	-2.856	4.30E-05
ENSBTAT00000055432	ENSBTAG00000031800	PPDPF	13	54608180	54609543	-	CODING	-0.24065	4.31E-05
ENSBTAT00000020701	ENSBTAG00000015582	HMOX1	5	73980776	73987841	+	CODING	0.464106	4.34E-05
ENSBTAT00000062037	ENSBTAG00000044604	snoU89	10	36862418	36862608	+	CODING	5.55073	4.38E-05
ENSBTAT00000017497	ENSBTAG00000013162	HSPA8	15	34216278	34220705	-	CODING	0.156329	4.38E-05
ENSBTAT00000016349	ENSBTAG00000012321	ZFAND6	21	26543823	26591580	+	CODING	0.343193	4.39E-05
ENSBTAT00000049397	ENSBTAG00000007399	LAMP2	X	4846778	4875136	+	CODING	0.452562	4.53E-05
ENSBTAT00000029455	ENSBTAG00000006084	PINK1	2	132601302	132621738	-	CODING	-0.31337	4.69E-05
ENSBTAT00000028712	ENSBTAG00000021549	PHPT1	11	106334245	106335688	-	CODING	-0.35893	5.00E-05
ENSBTAT00000006510	ENSBTAG00000004950	BRN	10	26288223	26289852	+	CODING	-1.22284	5.20E-05
ENSBTAT00000025431	ENSBTAG00000047739	TSC22D1	12	14722213	14725630	-	CODING	0.401392	5.32E-05
ENSBTAT00000014877	ENSBTAG00000023462	RPS6	8	24927468	24930788	+	CODING	-0.59678	5.58E-05
ENSBTAT00000020111	ENSBTAG00000015114	CALR	7	13731958	13735788	-	CODING	-0.43392	5.76E-05
ENSBTAT00000002429	ENSBTAG00000001864	NR4A3	8	65341202	65373695	+	CODING	-1.06366	5.92E-05
ENSBTAT00000010283	ENSBTAG00000007820	-	7	2568116	2568744	+	CODING	0.459116	6.21E-05
ENSBTAT00000021401	ENSBTAG00000016081	PTMA	2	47998614	47999416	+	CODING	-0.83931	6.48E-05
ENSBTAT00000019792	ENSBTAG00000014863	GPC	2	79255849	79304396	+	CODING	-0.38399	6.54E-05
ENSBTAT00000065271	ENSBTAG00000047561	VEGFA	23	17257357	17261031	-	CODING	-0.75839	6.55E-05
ENSBTAT00000019970	ENSBTAG00000015000	VCP	8	59732258	59746989	-	CODING	0.267763	6.56E-05
ENSBTAT00000026127	ENSBTAG00000019612	RNASE4	10	26423874	26445617	-	CODING	-0.73761	6.57E-05
ENSBTAT00000025165	ENSBTAG00000018909	-	4	67767893	67899241	-	CODING	-1.28888	6.97E-05
ENSBTAT00000026126	ENSBTAG00000045492	ANG	10	26429018	26445589	-	CODING	-1.07372	7.14E-05
ENSBTAT00000028392	ENSBTAG00000021308	IRS1	2	115790540	115794253	-	CODING	1.052925	7.36E-05
ENSBTAT00000042889	ENSBTAG00000004556	SLC2A3	5	101896622	101909500	-	CODING	-0.82265	7.39E-05
ENSBTAT00000049176	ENSBTAG00000026586	PPP1R14C	9	88384683	88500749	+	CODING	0.718218	7.41E-05
ENSBTAT00000014052	ENSBTAG00000010630	MGC165715	17	30106834	30143868	-	CODING	-1.00875	7.73E-05
ENSBTAT00000001993	ENSBTAG00000001521	UQCRB	14	70329414	70334124	+	CODING	-0.1331	7.81E-05
ENSBTAT00000002689	ENSBTAG00000002080	NOV	14	47005560	47013935	-	CODING	-1.19513	7.92E-05
ENSBTAT00000002654	ENSBTAG00000002049	HADH	6	18459380	18502809	-	CODING	0.435678	7.97E-05
ENSBTAT00000013950	ENSBTAG00000010549	IFRD1	4	55872976	55896662	-	CODING	-0.58797	8.03E-05
ENSBTAT00000012050	ENSBTAG00000009145	SLC7A4	17	74366170	74369086	-	CODING	1.047879	8.07E-05
ENSBTAT00000004364	ENSBTAG00000003362	HSP90B1	5	67940792	67959532	+	CODING	-0.52425	8.36E-05
ENSBTAT00000003460	ENSBTAG00000002670	C28H10ORF1	28	44952355	44953778	+	CODING	-1.21238	8.62E-05
ENSBTAT00000052944	ENSBTAG00000038527	-	4	18950187	18950489	-	CODING	-0.80623	8.70E-05
ENSBTAT00000054397	ENSBTAG00000009470	CLIC4	2	128710453	128783921	-	CODING	-0.59942	8.87E-05
ENSBTAT00000065446	ENSBTAG00000046046	MGC160046	24	40563771	40566586	+	CODING	0.713823	8.98E-05
ENSBTAT00000012544	ENSBTAG00000009535	RPS2	25	1520493	1522670	-	CODING	-0.11817	9.20E-05
ENSBTAT00000009415	ENSBTAG00000007153	C1QA	2	130792855	130795743	-	CODING	-2.70798	9.24E-05
ENSBTAT00000000651	ENSBTAG00000000510	ATG101	5	27962235	27968039	-	CODING	-0.60936	9.41E-05
ENSBTAT00000021641	ENSBTAG00000016269	ME2	24	50870262	50928290	+	CODING	-0.75647	9.42E-05
ENSBTAT00000002115	ENSBTAG00000001609	MAP2K6	19	61708769	61814973	-	CODING	0.505956	9.65E-05
ENSBTAT00000039206	ENSBTAG00000000894	PGK1	X	79282708	79305316	-	CODING	-10.0332	9.95E-05
ENSBTAT00000021544	ENSBTAG00000016185	ENAH	16	29238992	29442791	-	CODING	-1.12136	0.000104
ENSBTAT00000060214	ENSBTAG00000043222	SNORA12	26	21014545	21014692	-	CODING	-3.93459	0.000104
ENSBTAT00000061037	ENSBTAG00000044126	SNTB1	14	84253919	84504093	-	CODING	0.590202	0.000108
ENSBTAT00000000799	ENSBTAG00000000607	ABCF2	4	114587369	114602056	-	CODING	0.496779	0.000109
ENSBTAT00000012523	ENSBTAG00000009517	DBI	2	71561192	71566372	+	CODING	-0.35459	0.000112
ENSBTAT00000065403	ENSBTAG00000018707	LDB3	28	41657195	41713873	+	CODING	0.179665	0.000113
ENSBTAT00000018607	ENSBTAG00000014003	MYF6	5	10330469	10331983	+	CODING	-0.30204	0.000113
ENSBTAT00000023908	ENSBTAG00000017967	PTGES3	5	57091367	57112468	+	CODING	0.257574	0.000115

ENSBTAT00000025308	ENSBTAG00000019011	PGM1	3	82250295	82288338	-	CODING	0.474091	0.000115
ENSBTAT00000031167	ENSBTAG00000002069	BOLA	23	28502524	28506312	-	CODING	-0.3788	0.000116
ENSBTAT00000039828	ENSBTAG00000027654	EIF4EBP1	27	32951594	32973435	+	CODING	-0.43936	0.000117
ENSBTAT00000019939	ENSBTAG00000037605	BOLA-DQA1	23	25426330	25430097	-	CODING	-1.17535	0.000117
ENSBTAT00000005916	ENSBTAG00000025210	COL4A2	12	89112423	89165255	+	CODING	-0.80999	0.000117
ENSBTAT00000022319	ENSBTAG00000016779	CLIP1	17	55245237	55360147	+	CODING	0.396339	0.000123
ENSBTAT00000014100	ENSBTAG00000010652	PDE4C	7	4927816	4939026	+	CODING	0.957647	0.000123
ENSBTAT00000054742	ENSBTAG00000037470	-	14	38614593	38615161	+	CODING	-1.07488	0.000124
ENSBTAT00000019875	ENSBTAG00000014930	MYLK2	13	61900820	61915380	+	CODING	0.242523	0.000128
ENSBTAT00000011362	ENSBTAG00000008612	C1R	5	103737177	103747996	+	CODING	-1.63123	0.000128
ENSBTAT00000014486	ENSBTAG00000010907	PPP1R1A	5	25627052	25631242	+	CODING	0.109839	0.000135
ENSBTAT00000065946	ENSBTAG00000019269	COL6A2	1	147570799	147572432	+	CODING	-0.7969	0.000138
ENSBTAT00000039242	ENSBTAG00000003512	MYH7B	13	64888626	64912758	+	CODING	-0.58886	0.000138
ENSBTAT00000000733	ENSBTAG00000000562	TOMM20	28	7926960	7941655	-	CODING	-0.43987	0.000138
ENSBTAT00000037567	ENSBTAG00000026437	ULBP3	17	39881230	39884057	+	CODING	-0.99535	0.000142
ENSBTAT00000004836	ENSBTAG00000003711	EPAS1	11	28576347	28668899	+	CODING	-0.47982	0.000143
ENSBTAT00000047737	ENSBTAG00000033603	UQCC2	23	7796308	7820842	-	CODING	0.535215	0.000146
ENSBTAT00000001536	ENSBTAG00000001154	DGAT2	15	55940757	55973229	+	CODING	-0.99382	0.000148
ENSBTAT00000043065	ENSBTAG00000002853	HRC	18	56107442	56111943	-	CODING	0.332452	0.000148
ENSBTAT00000066191	ENSBTAG00000001546	MGAT1	7	41337278	41349616	-	CODING	-1.07483	0.000149
ENSBTAT00000015449	ENSBTAG00000011632	MAP1LC3B	18	13017744	13031053	+	CODING	0.408299	0.000154
ENSBTAT00000062960	ENSBTAG00000045661	Metazoa_SRP	10	42671324	42671620	+	CODING	-1.31048	0.000167
ENSBTAT00000019817	ENSBTAG00000014885	MYOM3	2	129388770	129437616	+	CODING	-0.57311	0.000172
ENSBTAT00000055345	ENSBTAG00000006907	NEB	2	44546002	44693638	+	CODING	1.109875	0.00019
ENSBTAT00000064703	ENSBTAG00000047621	-	16	4998445	5005553	+	CODING	-1.35403	0.000191
ENSBTAT00000013035	ENSBTAG00000009886	KDELR3	5	110660391	110673555	+	CODING	-1.18225	0.000201
ENSBTAT00000010022	ENSBTAG00000007622	CATD	29	50352064	50361497	+	CODING	0.289347	0.000207
ENSBTAT00000020850	ENSBTAG00000015704	TMCO3	12	90698790	90720746	+	CODING	-0.58882	0.000214
ENSBTAT00000000079	ENSBTAG00000037526	FABP4	14	46833665	46838053	-	CODING	-0.45987	0.000215
ENSBTAT00000010911	ENSBTAG00000008291	PROCR	13	65052810	65106553	+	CODING	-1.43154	0.00022
ENSBTAT00000010193	ENSBTAG00000007754	NDUFA3	18	63462789	63466137	-	CODING	-0.29602	0.000221
ENSBTAT00000006312	ENSBTAG00000004806	PHKB	18	15962196	16155801	+	CODING	0.327347	0.000223
ENSBTAT00000000950	ENSBTAG00000000711	NDRG1	14	9109762	9165926	+	CODING	-0.94253	0.000223
ENSBTAT00000034572	ENSBTAG00000024826	TECRL	6	81511554	81653990	-	CODING	-1.45114	0.000233
ENSBTAT00000002349	ENSBTAG00000001794	RPL36	7	19833364	19834615	-	CODING	-0.36411	0.000235
ENSBTAT00000063521	ENSBTAG00000047362	RCN1	15	63656459	63663593	+	CODING	-0.80576	0.000247
ENSBTAT00000016957	ENSBTAG00000012760	NDUFB3	2	90077970	90088465	+	CODING	-0.26202	0.000251
ENSBTAT00000057496	ENSBTAG00000039329	RAET1G	9	88232044	88402262	-	CODING	-1.82835	0.000253
ENSBTAT00000006244	ENSBTAG00000004757	LTBP4	18	50173268	50202104	+	CODING	-0.7728	0.000259
ENSBTAT00000021808	ENSBTAG00000016401	OPTN	13	28078762	28117627	+	CODING	-0.23952	0.00026
ENSBTAT00000022854	ENSBTAG00000017196	PDIA3	21	55924230	55946434	+	CODING	-0.43558	0.00026
ENSBTAT00000026724	ENSBTAG00000020060	TXNIP	3	21489097	21493251	+	CODING	0.158127	0.000261
ENSBTAT00000046493	ENSBTAG00000023600	APOD	1	72670963	72684269	+	CODING	-0.34385	0.000264
ENSBTAT00000064544	ENSBTAG00000046531	-	X	95330253	95330600	-	CODING	-0.48491	0.000274
ENSBTAT00000023042	ENSBTAG00000017328	PTI	13	74943621	74947760	-	CODING	-1.31325	0.000274
ENSBTAT00000011242	ENSBTAG00000008528	SLC25A1	17	74633475	74635952	-	CODING	-0.77061	0.000275
ENSBTAT00000014038	ENSBTAG00000010619	PPP3R1	11	66582636	66642276	-	CODING	0.281398	0.000282
ENSBTAT00000024600	ENSBTAG00000018483	NDUFS3	15	78582186	78586701	+	CODING	0.365511	0.000283
ENSBTAT00000024904	ENSBTAG00000018707	LDB3	28	41657195	41687361	+	CODING	0.113651	0.000288
ENSBTAT00000043182	ENSBTAG00000030575	BHLHE41	5	84233719	84236923	+	CODING	-0.60989	0.00032
ENSBTAT00000002868	ENSBTAG00000002216	MYOD1	15	35331401	35334046	-	CODING	0.932828	0.000323
ENSBTAT00000010806	ENSBTAG00000008218	NPTN	10	20207483	20270508	-	CODING	0.438343	0.000325
ENSBTAT00000003201	ENSBTAG00000002468	RPS28	7	18198992	18200124	+	CODING	-0.24548	0.000327
ENSBTAT00000048868	ENSBTAG00000034496	SHFM1	22	22098224	22098436	-	CODING	-0.12706	0.000339
ENSBTAT00000021268	ENSBTAG00000015989	RPS5	18	65845925	65851516	+	CODING	-0.2226	0.000346
ENSBTAT00000025561	ENSBTAG00000019203	S100A4	3	16887102	16888570	+	CODING	-0.68945	0.000354
ENSBTAT00000014398	ENSBTAG00000010849	ANKRD23	11	2768909	2773197	-	CODING	0.190086	0.000366
ENSBTAT00000026358	ENSBTAG00000019782	TPI1	5	103942403	103945759	-	CODING	0.109558	0.000369
ENSBTAT00000017500	ENSBTAG00000025441	HSPA1A	23	27331773	27333698	-	CODING	-0.15465	0.000373
ENSBTAT00000012857	ENSBTAG00000009749	USP2	15	30464926	30487308	-	CODING	0.385927	0.000374
ENSBTAT00000015261	ENSBTAG00000011483	SCARF1	19	23309862	23319829	-	CODING	0.561867	0.00038
ENSBTAT00000052289	ENSBTAG00000039728	RPLP1	16	52249583	52372101	-	CODING	-0.10939	0.000382

ENSBTAT00000023206	ENSBTAG00000017448	EFEMP1	11	38338744	38408288	-	CODING	-1.04086	0.000392
ENSBTAT00000001646	ENSBTAG00000001246	ATP1A1	3	27002873	27025641	-	CODING	-0.54123	0.000399
ENSBTAT00000025371	ENSBTAG00000019052	ANK3	28	15818095	15936518	-	CODING	0.567985	0.0004
ENSBTAT00000053151	ENSBTAG00000010851	SEPHS2	25	26867626	26868981	-	CODING	0.795585	0.000418
ENSBTAT00000000573	ENSBTAG00000000448	BDH1	1	72572941	72608810	-	CODING	-1.99175	0.000424
ENSBTAT00000015881	ENSBTAG00000011966	LAMC1	16	65545497	65662819	+	CODING	-0.58416	0.000424
ENSBTAT00000016887	ENSBTAG00000012705	DEGS1	16	28088797	28092872	-	CODING	-0.74335	0.000429
ENSBTAT00000023305	ENSBTAG00000017528	SNAI3	18	13958995	13964622	-	CODING	-2.59573	0.000437
ENSBTAT00000028174	ENSBTAG00000021140	RMND5A	11	48010205	48066194	-	CODING	0.402806	0.00045
ENSBTAT00000054745	ENSBTAG00000008997	ENG	11	98517162	98541363	-	CODING	-0.4708	0.000452
ENSBTAT00000038022	ENSBTAG00000013235	TINAGL1	2	122685823	122695535	-	CODING	-0.59393	0.000461
ENSBTAT00000008372	ENSBTAG00000025274	TUBB4B	11	105860110	105862549	-	CODING	0.322514	0.000467
ENSBTAT00000010928	ENSBTAG00000008303	FKBP1A	13	60276502	60303755	+	CODING	-0.33157	0.000471
ENSBTAT00000010780	ENSBTAG00000008195	PHKG1	25	28005386	28014212	-	CODING	0.512755	0.000478
ENSBTAT00000013963	ENSBTAG00000010562	CD34	16	77367502	77389361	+	CODING	-0.52989	0.000483
ENSBTAT00000015948	ENSBTAG00000012024	SLC29A2	29	45056314	45063665	-	CODING	0.506179	0.000485
ENSBTAT00000029256	ENSBTAG00000021944	C10H14ORF1	10	44879976	44894546	+	CODING	-0.23767	0.000488
ENSBTAT00000056276	ENSBTAG00000008683	LDHA	29	26543861	26553359	-	CODING	0.166362	0.000495
ENSBTAT00000006358	ENSBTAG00000004840	C1S	5	103768066	103779250	-	CODING	-1.02178	0.000506
ENSBTAT00000019626	ENSBTAG00000014751	ZIC1	1	121863151	121867967	-	CODING	1.081121	0.000512
ENSBTAT00000042695	ENSBTAG00000012066	PECAM1	19	49175892	49238414	-	CODING	-0.55888	0.000512
ENSBTAT00000011042	ENSBTAG00000008389	HTRA1	26	42659876	42716633	+	CODING	0.458747	0.000514
ENSBTAT00000020468	ENSBTAG00000015402	GREB1	11	86199420	86268193	-	CODING	-2.31277	0.000519
ENSBTAT00000021521	ENSBTAG00000016169	ID1	13	61726125	61727283	+	CODING	-0.56774	0.000527
ENSBTAT00000063744	ENSBTAG00000046450	EIF1	3	60346812	60347472	+	CODING	-0.14733	0.000532
ENSBTAT00000064065	ENSBTAG00000001945	ARG2	10	80078810	80113797	+	CODING	-3.81974	0.000547
ENSBTAT00000018339	ENSBTAG00000013799	-	2	107981292	107982291	-	CODING	0.249019	0.000548
ENSBTAT00000003563	ENSBTAG00000002747	ABCA5	19	61873253	61919888	+	CODING	0.882581	0.000556
ENSBTAT00000055368	ENSBTAG00000020796	UBE2D1	26	663449	699996	-	CODING	-0.47491	0.00056
ENSBTAT00000018278	ENSBTAG00000013755	ITGB5	1	69801844	69899676	-	CODING	-0.94545	0.000563
ENSBTAT00000022929	ENSBTAG00000011022	ARPP19	10	57923246	57937867	+	CODING	-0.35131	0.000567
ENSBTAT00000001948	ENSBTAG00000001489	TUBA1A	5	30821251	30825700	+	CODING	-1.00466	0.00057
ENSBTAT00000023082	ENSBTAG00000030499	DIAPH1	7	54285455	54437207	-	CODING	-0.6195	0.000604
ENSBTAT00000001019	ENSBTAG00000000770	PGM2L1	15	54484806	54537330	-	CODING	0.965855	0.000611
ENSBTAT00000020424	ENSBTAG00000015366	SFRP4	4	50009882	50020466	-	CODING	-1.89763	0.000615
ENSBTAT00000016317	ENSBTAG00000012302	RTN4RL1	19	23559326	23560666	-	CODING	2.288016	0.000618
ENSBTAT00000025126	ENSBTAG00000005349	RPL27A	15	44469327	44472127	-	CODING	-0.37581	0.000621
ENSBTAT00000044712	ENSBTAG00000031544	DDIT3	5	56285008	56289214	+	CODING	-0.87448	0.000623
ENSBTAT00000063474	ENSBTAG00000000139	SETD8	17	54457612	54471710	-	CODING	-0.91348	0.00063
ENSBTAT00000032851	ENSBTAG00000002108	YWHAQ	11	87842280	87873674	+	CODING	-0.71506	0.00063
ENSBTAT00000001567	ENSBTAG00000001183	KLHL33	10	26728622	26738305	+	CODING	0.535447	0.000634
ENSBTAT00000020168	ENSBTAG00000015163	TM4SF1	1	119750363	119758888	+	CODING	-0.54799	0.000646
ENSBTAT00000020452	ENSBTAG00000015388	RPL18	18	55710193	55713956	-	CODING	-0.17111	0.000647
ENSBTAT00000026470	ENSBTAG00000019867	COMMD1	11	60427663	60598437	+	CODING	-0.82884	0.000671
ENSBTAT00000017648	ENSBTAG00000017715	HOMER2	21	23577846	23668524	-	CODING	-0.42053	0.000673
ENSBTAT00000000160	ENSBTAG00000000139	SETD8	17	54457612	54476577	-	CODING	0.651628	0.000694
ENSBTAT00000035150	ENSBTAG00000005326	CSNK1A1	7	62861221	62902057	-	CODING	-1.05831	0.000702
ENSBTAT00000016993	ENSBTAG00000012788	COX6A1	17	64995248	64997121	+	CODING	-0.52047	0.000709
ENSBTAT00000002015	ENSBTAG00000001538	RPS16	3	117506247	117506811	+	CODING	-0.26812	0.000722
ENSBTAT00000026782	ENSBTAG00000020105	-	8	76468680	76484933	-	CODING	-0.50379	0.000726
ENSBTAT00000013663	ENSBTAG00000010347	EZR	9	96598249	96643275	-	CODING	-0.6533	0.000731
ENSBTAT00000046700	ENSBTAG00000005339	-	23	17263646	17269998	+	CODING	-0.67393	0.000733
ENSBTAT00000019929	ENSBTAG00000014971	SEC61G	22	1445067	1451951	+	CODING	-0.30945	0.000757
ENSBTAT00000028093	ENSBTAG00000021093	RPS16	18	49393725	49396191	-	CODING	-0.17545	0.000762
ENSBTAT00000040802	ENSBTAG00000028421	U1	19	15193841	15194001	-	CODING	-3.60961	0.000766
ENSBTAT00000022832	ENSBTAG00000017181	MACROD1	29	42890800	43092842	-	CODING	-0.14275	0.000767
ENSBTAT00000016020	ENSBTAG00000012082	TNFRSF12A	25	2439751	2441210	+	CODING	-1.16801	0.000786
ENSBTAT00000064843	ENSBTAG00000047174	-	22	52425504	52428832	+	CODING	0.27523	0.000791
ENSBTAT00000055318	ENSBTAG00000008732	ZC3H12C	15	20493944	20526349	+	CODING	-2.01359	0.000797
ENSBTAT00000013799	ENSBTAG00000010452	PODXL	4	96032591	96039050	-	CODING	-0.50947	0.000806
ENSBTAT00000013310	ENSBTAG00000010083	HADHB	11	73215424	73246167	-	CODING	0.312471	0.000806
ENSBTAT00000015883	ENSBTAG00000011969	HSPB1	25	34858438	34861035	+	CODING	0.407542	0.000844

ENSBTAT00000064159	ENSBTAG00000047249	WBSCR1	25	33708591	33729652	-	CODING	0.246523	0.000852
ENSBTAT00000055956	ENSBTAG00000001137	CLTA	8	60849478	60866939	+	CODING	-0.55721	0.000868
ENSBTAT00000022366	ENSBTAG00000037558	GRO1	6	90822748	90824841	+	CODING	-1.71294	0.00087
ENSBTAT00000020102	ENSBTAG00000015107	SLC16A1	3	30533845	30563298	+	CODING	-0.53746	0.000885
ENSBTAT00000013093	ENSBTAG00000009923	KIAA1737	10	89365866	89381540	+	CODING	0.432019	0.000886
ENSBTAT00000049347	ENSBTAG00000034885	MGC148992	12	11625778	11641053	+	CODING	-0.80412	0.000898
ENSBTAT00000002779	ENSBTAG00000002144	ADRB2	7	62229186	62230442	+	CODING	-0.92297	0.000926
ENSBTAT00000018645	ENSBTAG00000014032	EIF3H	14	49733172	49830429	+	CODING	-0.25412	0.000926
ENSBTAT00000028880	ENSBTAG00000021672	RGS1	16	13314192	13318292	-	CODING	-0.92376	0.000939
ENSBTAT00000012815	ENSBTAG00000009717	FGL2	4	44236254	44240215	+	CODING	-0.77044	0.000943
ENSBTAT00000038384	ENSBTAG00000026848	-	3	11468021	11468512	-	CODING	-0.21006	0.000966
ENSBTAT00000015705	ENSBTAG00000011834	CCDC47	19	48662435	48682292	-	CODING	0.345391	0.000968
ENSBTAT00000043205	ENSBTAG00000030587	LASP1	19	40090995	40131373	+	CODING	-0.77947	0.000976
ENSBTAT00000043035	ENSBTAG00000008135	SLIRP	10	89914334	89923167	+	CODING	-0.27023	0.001015
ENSBTAT00000009126	ENSBTAG00000006951	LMO2	15	65394511	65407118	-	CODING	-0.9018	0.001022
ENSBTAT00000003999	ENSBTAG00000003072	ACADVL	19	27568181	27573378	+	CODING	-0.25844	0.001038
ENSBTAT00000010982	ENSBTAG00000008340	ATOX1	7	64945749	64961110	-	CODING	-0.6694	0.001039
ENSBTAT00000046421	ENSBTAG00000032719	TFRC	1	71260068	71280648	-	CODING	-0.37927	0.001058
ENSBTAT00000000144	ENSBTAG00000000132	EIF4A1	19	27915413	27921421	+	CODING	-0.47549	0.001062
ENSBTAT00000026074	ENSBTAG00000019569	CD151	29	50695518	50700467	-	CODING	-0.46481	0.001063
ENSBTAT00000012497	ENSBTAG00000010125	STAT5B	19	42960226	42996671	-	CODING	0.556109	0.001065
ENSBTAT00000011931	ENSBTAG00000009055	RNF144B	23	38952210	39043167	-	CODING	-0.4374	0.001065
ENSBTAT00000021209	ENSBTAG00000015950	ISOC2	18	62502067	62508467	+	CODING	-1.15354	0.001094
ENSBTAT00000022077	ENSBTAG00000016595	CYSTM1	7	53047810	53118491	+	CODING	0.22499	0.001112
ENSBTAT00000018566	ENSBTAG00000013953	CALD1	4	99475015	99580189	+	CODING	-0.48287	0.001121
ENSBTAT00000024807	ENSBTAG00000018644	PDZRN3	22	28238772	28506578	+	CODING	0.432067	0.001145
ENSBTAT00000013065	ENSBTAG00000009899	CHID1	29	50539408	50558480	+	CODING	-6.69597	0.001153
ENSBTAT00000047899	ENSBTAG00000033727	RBPMS	27	25637686	25816012	+	CODING	-0.94277	0.001167
ENSBTAT00000032589	ENSBTAG00000012519	XDH	11	14176298	14236380	-	CODING	0.926841	0.001202
ENSBTAT00000026631	ENSBTAG00000019994	-	2	91685397	91686142	-	CODING	-0.57578	0.001218
ENSBTAT00000026776	ENSBTAG00000020099	STMN2	14	44969002	45027081	+	CODING	-2.52141	0.00122
ENSBTAT00000045935	ENSBTAG00000032374	SELK	22	47582815	47590574	+	CODING	-0.52256	0.001242
ENSBTAT00000015875	ENSBTAG00000011963	RPS19	18	51689627	51697161	-	CODING	-0.29051	0.00125
ENSBTAT00000010491	ENSBTAG00000007979	TNPO1	20	8650349	8694889	-	CODING	0.672904	0.001251
ENSBTAT00000012594	ENSBTAG00000009576	PACSIN3	15	78266386	78275225	-	CODING	0.288048	0.001255
ENSBTAT00000005969	ENSBTAG00000004542	C9ORF59	11	101477218	101489266	-	CODING	1.89455	0.001272
ENSBTAT00000054074	ENSBTAG00000038652	-	5	74935713	74959662	+	CODING	-0.42235	0.001279
ENSBTAT00000027347	ENSBTAG00000020528	PCOLCE	25	36490771	36495789	-	CODING	-0.90596	0.001287
ENSBTAT00000015385	ENSBTAG00000011580	DAG1	22	51187154	51200326	-	CODING	-0.39026	0.001291
ENSBTAT00000018479	ENSBTAG00000030190	COL6A3	3	117409071	117482330	-	CODING	-0.9308	0.001296
ENSBTAT00000014907	ENSBTAG00000011224	CITED2	9	78071006	78073397	-	CODING	-0.64588	0.0013
ENSBTAT00000064807	ENSBTAG00000014448	-	25	28101590	28157501	+	CODING	-1.54029	0.001308
ENSBTAT00000003869	ENSBTAG00000002971	CUTC	26	20550908	20583501	+	CODING	-0.38289	0.001327
ENSBTAT00000019575	ENSBTAG00000014710	RAP1A	3	31746448	31764423	-	CODING	-0.38824	0.001362
ENSBTAT00000024512	ENSBTAG00000018424	CXCR7	3	116650278	116662501	+	CODING	-0.76401	0.001377
ENSBTAT00000061386	ENSBTAG00000020296	UBR3	2	26361548	26565226	-	CODING	0.25574	0.00138
ENSBTAT00000005240	ENSBTAG00000004014	FBLN2	22	58990282	59038424	-	CODING	-0.88141	0.001382
ENSBTAT00000063362	ENSBTAG00000047824	-	16	31136171	31137374	-	CODING	0.430142	0.001407
ENSBTAT00000031821	ENSBTAG00000030592	UBL5	1	154150028	154150249	+	CODING	-0.40471	0.00144
ENSBTAT00000053426	ENSBTAG00000003994	IGFBP3	4	76705105	76712709	+	CODING	-0.64962	0.001468
ENSBTAT00000025024	ENSBTAG00000018800	RPS4	X	83532034	83536488	+	CODING	-0.28306	0.001475
ENSBTAT00000004227	ENSBTAG00000003263	TBCA	10	8737160	8822375	-	CODING	-0.69591	0.001475
ENSBTAT00000000086	ENSBTAG00000000078	GLIPR2	8	60797959	60816816	+	CODING	-0.9646	0.00149
ENSBTAT00000057533	ENSBTAG00000007662	GRP78	11	96115572	96119306	-	CODING	-0.40048	0.001526
ENSBTAT00000017839	ENSBTAG00000013411	-	16	45401695	45416462	+	CODING	-0.5337	0.001529
ENSBTAT00000019555	ENSBTAG00000014693	TMEM88	19	28156792	28157769	+	CODING	-0.64414	0.001529
ENSBTAT00000019232	ENSBTAG00000014465	SERPINE1	25	36198560	36206859	-	CODING	-0.84868	0.001549
ENSBTAT00000063708	ENSBTAG00000047694	-	21	55966632	55968362	+	CODING	-0.16066	0.001549
ENSBTAT00000009158	ENSBTAG00000006969	TUBB5	23	28101727	28105320	-	CODING	0.33123	0.001569
ENSBTAT00000010595	ENSBTAG00000008054	MGC133632	14	65337750	65345928	+	CODING	-0.52511	0.001575
ENSBTAT00000020020	ENSBTAG00000015038	HADHA	11	73246485	73288803	+	CODING	-0.28224	0.001585
ENSBTAT00000064819	ENSBTAG00000047975	DOK5	13	82696138	82803966	+	CODING	-0.77699	0.00162

ENSBTAT00000023981	ENSBTAG00000018016	NUPR1	25	26340079	26341408	-	CODING	-0.68274	0.001678
ENSBTAT00000064686	ENSBTAG00000048152	TUBG1	19	43316471	43321165	+	CODING	0.508626	0.00168
ENSBTAT00000004621	ENSBTAG00000003553	ZFP36L2	11	25585493	25587785	-	CODING	-0.72106	0.001695
ENSBTAT00000026816	ENSBTAG00000025280	-	23	50603365	50617511	+	CODING	-0.42608	0.001715
ENSBTAT00000021189	ENSBTAG00000015926	ABLIM2	6	119107715	119213224	-	CODING	0.572239	0.001722
ENSBTAT00000004519	ENSBTAG00000003476	FEM1A	7	20565301	20568880	-	CODING	0.206985	0.001724
ENSBTAT00000003167	ENSBTAG00000031363	AES	7	21977262	21983594	+	CODING	-0.28938	0.001724
ENSBTAT00000045476	ENSBTAG00000032057	-	3	28623391	28623702	-	CODING	0.261357	0.001725
ENSBTAT00000015760	ENSBTAG00000011873	KCNE3	15	54587990	54588289	-	CODING	-3.45381	0.001725
ENSBTAT00000037682	ENSBTAG00000009887	DDX17	5	110675824	110691542	-	CODING	0.368412	0.001738
ENSBTAT00000064422	ENSBTAG00000015025	YAF2	5	38674059	38753871	+	CODING	-1.05745	0.001741
ENSBTAT00000005549	ENSBTAG00000004237	BTC	6	91430305	91480129	-	CODING	0.462632	0.001755
ENSBTAT00000029117	ENSBTAG00000021843	UBQLN2	X	98958218	98960092	+	CODING	0.388802	0.001817
ENSBTAT00000007043	ENSBTAG00000005354	LRRFIP1	3	117707634	117869819	+	CODING	-0.64234	0.001828
ENSBTAT00000003575	ENSBTAG00000002758	THBD	13	42217371	42221004	-	CODING	-0.79354	0.001828
ENSBTAT00000054581	ENSBTAG00000038464	PLIN5	7	20816181	20827916	+	CODING	-0.95735	0.001838
ENSBTAT00000012779	ENSBTAG00000009689	RWDD1	9	34577932	34595665	-	CODING	-0.25792	0.00184
ENSBTAT00000006899	ENSBTAG00000005244	RASL11A	12	32842604	32844825	-	CODING	-1.06419	0.001847
ENSBTAT00000012773	ENSBTAG00000009683	PSMA6	21	45974822	45998591	+	CODING	0.267048	0.001897
ENSBTAT00000024371	ENSBTAG00000031998	CXCL16	19	27249528	27253097	+	CODING	-0.93577	0.001914
ENSBTAT00000024295	ENSBTAG00000018252	ARRDC4	21	8924763	8939028	-	CODING	0.740906	0.001919
ENSBTAT00000022554	ENSBTAG00000016957	-	8	112124219	112138177	+	CODING	1.065044	0.001925
ENSBTAT00000000944	ENSBTAG00000000706	ADAMTS1	1	8955134	8963815	+	CODING	-0.5162	0.001926
ENSBTAT00000013399	ENSBTAG00000010155	-	19	14735718	14741999	+	CODING	-1.70829	0.001968
ENSBTAT00000009385	ENSBTAG00000007131	GADL1	22	5258463	5452369	-	CODING	0.460498	0.001971
ENSBTAT00000018691	ENSBTAG00000014068	VDAC3	27	36923762	36932879	+	CODING	0.268871	0.001979
ENSBTAT00000064237	ENSBTAG00000046358	PABPC1	14	65816006	65833756	+	CODING	-0.33056	0.002059
ENSBTAT00000009699	ENSBTAG00000007375	MIF	17	73273379	73274171	+	CODING	-0.51797	0.002081
ENSBTAT00000064804	ENSBTAG00000046122	NRARP	11	105790354	105790698	+	CODING	-2.31417	0.002083
ENSBTAT00000001086	ENSBTAG00000000820	GNG11	4	11074737	11079756	+	CODING	-0.71152	0.002162
ENSBTAT00000063314	ENSBTAG00000012638	S100A12	3	17163820	17165144	+	CODING	-1.97609	0.002206
ENSBTAT00000005555	ENSBTAG00000004242	ARPC1A	25	37564390	37587556	-	CODING	-0.43409	0.002214
ENSBTAT00000019230	ENSBTAG00000014463	CAMK2D	6	12965030	13271053	+	CODING	0.78003	0.002228
ENSBTAT00000016620	ENSBTAG00000012519	XDH	11	14176298	14281717	-	CODING	-0.66369	0.002251
ENSBTAT00000032643	ENSBTAG00000023792	-	9	42568764	42569201	-	CODING	-0.45693	0.002259
ENSBTAT00000018863	ENSBTAG00000014182	CTNNA1	7	51688098	51880519	+	CODING	-0.51663	0.002262
ENSBTAT00000020267	ENSBTAG00000025564	RPL36A	10	42699004	42700655	-	CODING	-0.34858	0.002293
ENSBTAT00000005472	ENSBTAG00000004178	ACOX2	22	43379504	43410315	+	CODING	-0.70421	0.002354
ENSBTAT00000026428	ENSBTAG00000019834	ARL15	20	24955390	25026808	+	CODING	-1.26856	0.002387
ENSBTAT00000019176	ENSBTAG00000014417	-	25	33157514	33168430	+	CODING	0.241411	0.002413
ENSBTAT00000063822	ENSBTAG00000046521	-	2	93684245	93684415	+	CODING	1.894283	0.002424
ENSBTAT00000061618	ENSBTAG00000044038	TEN1	19	56279922	56300266	-	CODING	-0.6066	0.002439
ENSBTAT00000056479	ENSBTAG00000039954	COPZ2	19	39081066	39090187	+	CODING	-0.71418	0.00244
ENSBTAT00000011485	ENSBTAG00000008717	SERPINE2	2	112870323	112900274	-	CODING	-1.02542	0.002443
ENSBTAT00000007806	ENSBTAG00000005947	PLAU	28	29964983	29971029	+	CODING	-1.09448	0.002457
ENSBTAT00000002616	ENSBTAG00000002018	EIF5A	19	27648382	27653256	+	CODING	0.170761	0.002474
ENSBTAT00000018484	ENSBTAG00000013919	BOLA-DRB3	23	25458594	25476944	+	CODING	-0.4364	0.00248
ENSBTAT00000065287	ENSBTAG00000048156	-	21	19391574	19391762	-	CODING	-0.7007	0.002517
ENSBTAT00000019814	ENSBTAG00000014883	GABARAP	19	27586886	27588815	-	CODING	-0.16489	0.002519
ENSBTAT00000044250	ENSBTAG00000031249	PSMG4	23	50327386	50331704	-	CODING	-0.73428	0.002543
ENSBTAT00000036839	ENSBTAG00000010012	BTF3	20	8038617	8045160	-	CODING	-0.1748	0.00256
ENSBTAT00000001709	ENSBTAG00000001298	STAMBPL1	26	10597573	10650140	+	CODING	0.881745	0.002647
ENSBTAT00000024301	ENSBTAG00000018255	ACTN1	10	81023526	81121590	-	CODING	-0.81103	0.00266
ENSBTAT00000001098	ENSBTAG00000000833	TAX1BP3	19	24971932	24977062	-	CODING	-0.57162	0.002662
ENSBTAT00000055693	ENSBTAG00000040308	RPS13	15	35943265	35945982	+	CODING	-0.41093	0.002676
ENSBTAT00000064257	ENSBTAG00000046184	-	10	43935668	43936221	-	CODING	-0.24479	0.00268
ENSBTAT00000025044	ENSBTAG00000018813	IDH3B	13	52978729	52983681	+	CODING	0.294949	0.002691
ENSBTAT00000026289	ENSBTAG00000019725	UBE4A	15	29476822	29516720	+	CODING	0.443044	0.002719
ENSBTAT00000064873	ENSBTAG00000000979	SMIM19	27	37055089	37070241	+	CODING	-0.39783	0.002763
ENSBTAT00000008135	ENSBTAG00000006193	IRAK2	22	16694678	16750676	-	CODING	0.610469	0.00277
ENSBTAT00000052142	ENSBTAG00000003758	TKT	22	48274004	48288061	+	CODING	1.291652	0.002775
ENSBTAT00000064158	ENSBTAG00000046248	ARPC1B	25	37546485	37559078	-	CODING	-0.89053	0.002803

ENSBTAT00000025142	ENSBTAG00000018887	IPO13	3	102682317	102701941	-	CODING	0.331562	0.002826
ENSBTAT00000014839	ENSBTAG00000011173	FAM189A2	8	45784659	45816860	+	CODING	-0.43811	0.002857
ENSBTAT00000042554	ENSBTAG00000030169	SUMO2	19	56923424	56931107	+	CODING	-0.37479	0.002879
ENSBTAT00000001490	ENSBTAG00000001120	CORO6	19	21426533	21434772	-	CODING	0.210841	0.002886
ENSBTAT00000012635	ENSBTAG00000009603	UQCRH	3	100391833	100401195	-	CODING	-0.3323	0.002896
ENSBTAT00000027728	ENSBTAG00000020807	FAU	29	43990460	43991956	-	CODING	-0.36212	0.002915
ENSBTAT00000002967	ENSBTAG00000002302	CD59	15	65145073	65166597	-	CODING	-0.43948	0.002918
ENSBTAT00000018411	ENSBTAG00000013863	DUSP1	20	4449109	4452189	-	CODING	-0.18412	0.002921
ENSBTAT00000003766	ENSBTAG00000002898	UNC45B	19	15233409	15265756	-	CODING	0.328575	0.002936
ENSBTAT00000011920	ENSBTAG00000009047	YPEL3	25	26451072	26454431	+	CODING	0.259849	0.002966
ENSBTAT00000015066	ENSBTAG00000011338	NREP	10	2161055	2193663	+	CODING	-0.69118	0.00301
ENSBTAT00000021896	ENSBTAG00000037673	GSTM4	3	33874019	33880598	-	CODING	0.699002	0.003016
ENSBTAT00000045065	ENSBTAG00000000215	GNB1	16	52106960	52184132	+	CODING	-0.43837	0.003073
ENSBTAT00000007028	ENSBTAG00000005345	ARPC3	17	56573543	56584354	-	CODING	-0.46538	0.003086
ENSBTAT00000003204	ENSBTAG00000002473	ANGPTL4	7	18236517	18243581	+	CODING	-0.71588	0.003099
ENSBTAT00000002640	ENSBTAG00000002036	CACNB1	19	40298255	40310382	+	CODING	0.333116	0.003116
ENSBTAT00000033843	ENSBTAG00000024407	-	3	19259499	19260488	+	CODING	-0.76224	0.003151
ENSBTAT00000045880	ENSBTAG00000009570	C19orf80	7	16878927	16880796	+	CODING	1.222026	0.003166
ENSBTAT00000001706	ENSBTAG00000001296	TMEM50A	2	128256214	128272007	-	CODING	-0.49941	0.003173
ENSBTAT00000053355	ENSBTAG00000010727	ATG4D	7	16279919	16288392	+	CODING	-1.67649	0.003177
ENSBTAT00000064624	ENSBTAG00000045783	-	16	5201143	5201319	-	CODING	-0.3403	0.003228
ENSBTAT00000044192	ENSBTAG00000031205	RPS4X	7	23580042	23580833	-	CODING	-0.54091	0.003259
ENSBTAT00000013994	ENSBTAG00000024095	THRAP3	3	110137251	110208496	-	CODING	0.404917	0.003319
ENSBTAT00000000566	ENSBTAG00000000442	RBP4	26	14940551	14946750	-	CODING	-0.86501	0.003352
ENSBTAT00000023743	ENSBTAG00000017863	SRGN	28	25631917	25647735	+	CODING	-0.9182	0.003356
ENSBTAT00000065769	ENSBTAG00000047330	FABP5	14	46644609	46649827	+	CODING	-0.31043	0.003357
ENSBTAT00000009364	ENSBTAG00000007116	ARRDC3	7	93240419	93253094	-	CODING	0.593807	0.003362
ENSBTAT00000015325	ENSBTAG00000011532	MLLT6	19	39938325	39956916	+	CODING	0.668417	0.003369
ENSBTAT00000065849	ENSBTAG00000046004	-	X	101428040	101428600	-	CODING	-0.46011	0.003373
ENSBTAT00000023267	ENSBTAG00000017504	FAIM2	5	30155763	30185025	+	CODING	1.616116	0.003381
ENSBTAT00000000730	ENSBTAG00000000560	-	19	13525432	13526742	+	CODING	-0.23524	0.003461
ENSBTAT00000029742	ENSBTAG00000012450	RAPGEF2	17	40483943	40580445	-	CODING	-0.97124	0.003464
ENSBTAT00000053590	ENSBTAG00000037991	-	15	46902643	46904084	+	CODING	-2.12265	0.00349
ENSBTAT00000002513	ENSBTAG00000001932	MGC142781	11	99576794	99589668	-	CODING	0.235393	0.003508
ENSBTAT00000063622	ENSBTAG00000048120	7SK	3	34060769	34060962	-	CODING	-1.5004	0.003525
ENSBTAT00000017940	ENSBTAG00000013492	PRKAG3	2	107509452	107516981	-	CODING	0.447285	0.003578
ENSBTAT00000003990	ENSBTAG00000003067	PSMB7	11	95401692	95458914	-	CODING	0.263263	0.003656
ENSBTAT00000006947	ENSBTAG00000005280	ADA	13	73750479	73773983	-	CODING	-0.64341	0.003663
ENSBTAT00000008092	ENSBTAG00000006152	-	4	84628122	84628559	-	CODING	-0.63006	0.003667
ENSBTAT00000009921	ENSBTAG00000007537	PEX19	3	9517705	9523145	+	CODING	0.386071	0.003681
ENSBTAT00000064667	ENSBTAG00000005932	FAM184B	6	38614370	38672306	-	CODING	1.137038	0.00372
ENSBTAT00000020833	ENSBTAG00000015690	PLIN4	7	20831528	20841683	+	CODING	0.526851	0.003743
ENSBTAT00000047742	ENSBTAG00000033603	UQCC2	23	7758144	7783948	-	CODING	-0.4045	0.003752
ENSBTAT00000065799	ENSBTAG00000048229	TPT1	25	1394201	1417716	-	CODING	-0.04231	0.003752
ENSBTAT00000037527	ENSBTAG00000012683	SRRM2	25	2286717	2304536	-	CODING	0.324975	0.003752
ENSBTAT00000052340	ENSBTAG00000010464	MN1	17	69431677	69478074	-	CODING	0.970454	0.003798
ENSBTAT00000035577	ENSBTAG00000008024	UCHL3	12	50948444	50997544	+	CODING	-0.41282	0.003808
ENSBTAT00000065262	ENSBTAG00000047051	-	X	36171714	36172838	+	CODING	-0.28386	0.003814
ENSBTAT00000024678	ENSBTAG00000018542	COX5B	11	2916017	2918064	-	CODING	-0.12024	0.003914
ENSBTAT00000002422	ENSBTAG00000001858	-	5	70910366	70911285	+	CODING	-0.41304	0.00392
ENSBTAT00000028608	ENSBTAG00000021461	PSMD3	19	40961497	40974702	+	CODING	0.318827	0.00393
ENSBTAT00000009965	ENSBTAG00000013343	FTL	18	55992910	55994741	+	CODING	-0.33623	0.003945
ENSBTAT00000017835	ENSBTAG00000013406	CSRP2	5	6266018	6274473	-	CODING	-1.11522	0.003973
ENSBTAT00000056613	ENSBTAG00000010957	LHPP	26	44431214	44558731	+	CODING	-0.71069	0.00398
ENSBTAT00000018052	ENSBTAG00000013579	TMEM66	27	25391027	25416076	-	CODING	-0.31643	0.003993
ENSBTAT00000063143	ENSBTAG00000047029	-	11	47050711	47051142	-	CODING	-0.38807	0.00404
ENSBTAT00000012254	ENSBTAG00000009302	RCAN2	23	19437558	19459166	-	CODING	-1.41731	0.004044
ENSBTAT00000014734	ENSBTAG00000025666	RPS29	10	26814899	26815069	-	CODING	-3.26878	0.004074
ENSBTAT00000034295	ENSBTAG00000015559	ERI3	3	102293112	102421170	+	CODING	-1.55863	0.004077
ENSBTAT00000027339	ENSBTAG00000020518	PARK7	16	46249163	46265920	-	CODING	0.178753	0.004082
ENSBTAT00000013141	ENSBTAG00000009959	SLC6A8	X	39828310	39835611	+	CODING	0.377741	0.004111
ENSBTAT00000023094	ENSBTAG00000017368	YBX1	3	104112934	104133708	-	CODING	-0.12095	0.004127

ENSBTAT00000014490	ENSBTAG00000010910	MYEOV2	3	120123114	120127712	-	CODING	-0.26946	0.004223
ENSBTAT00000017009	ENSBTAG00000012804	REEP5	10	1043713	1094330	+	CODING	0.228054	0.004244
ENSBTAT00000024225	ENSBTAG00000002960	WDR82P1	22	49218179	49227366	+	CODING	-0.30502	0.004249
ENSBTAT00000011978	ENSBTAG00000009086	LOXL1	21	35057678	35082119	-	CODING	-1.81515	0.004263
ENSBTAT00000023465	ENSBTAG00000017639	RPS6KA3	X	130193741	130300294	+	CODING	0.456109	0.004289
ENSBTAT00000000683	ENSBTAG00000000524	CSTB	1	146555393	146558844	-	CODING	-0.70813	0.004295
ENSBTAT00000030194	ENSBTAG00000011190	FLNA	X	40310757	40332714	-	CODING	-0.54314	0.00432
ENSBTAT00000061284	ENSBTAG00000001564	PDE4DIP	3	22916551	23063522	-	CODING	0.152617	0.004345
ENSBTAT00000017147	ENSBTAG00000012899	IFNGR2	1	1376827	1397948	-	CODING	-0.55784	0.004425
ENSBTAT00000048484	ENSBTAG00000034206	EMCN	6	25578634	25700215	+	CODING	-0.51259	0.00443
ENSBTAT00000014796	ENSBTAG00000011140	FAM162A	1	67422932	67443491	+	CODING	-0.26291	0.004447
ENSBTAT00000018267	ENSBTAG00000013744	SYNPO	7	64020057	64027999	+	CODING	-0.17888	0.004464
ENSBTAT00000052050	ENSBTAG00000039555	COX7C	7	88648457	88650528	+	CODING	0.274473	0.004466
ENSBTAT00000044763	ENSBTAG00000031573	ITGB1BP3	7	21290813	21294189	-	CODING	0.111128	0.004525
ENSBTAT00000007691	ENSBTAG00000005847	ROCK2	11	86501577	86583652	+	CODING	-0.45799	0.004529
ENSBTAT00000027593	ENSBTAG00000020704	RAMP3	4	77171037	77193849	-	CODING	-1.25666	0.004537
ENSBTAT00000040741	ENSBTAG00000028359	U3	19	9798918	9799131	-	CODING	-1.24166	0.00455
ENSBTAT00000019505	ENSBTAG00000014649	CPT2	3	93601359	93625434	-	CODING	-0.75924	0.004566
ENSBTAT00000057596	ENSBTAG00000040028	MGC166429	7	2600732	2618482	-	CODING	0.24355	0.00458
ENSBTAT00000009440	ENSBTAG00000007172	GOT2	18	26533095	26556740	-	CODING	0.13778	0.004634
ENSBTAT00000026372	ENSBTAG00000019794	SYPL1	4	47423619	47453580	-	CODING	0.239961	0.004636
ENSBTAT00000015499	ENSBTAG00000011677	HIST1H1C	23	31636947	31637588	+	CODING	-1.42674	0.004715
ENSBTAT00000046195	ENSBTAG00000032557	TMEM258	29	40927662	40931388	-	CODING	-0.42541	0.004716
ENSBTAT00000053663	ENSBTAG00000012890	SLC25A3	5	63086353	63092547	+	CODING	-0.81448	0.004735
ENSBTAT00000002293	ENSBTAG00000001748	TRIM45	3	26213057	26227392	+	CODING	-1.39489	0.004756
ENSBTAT00000025095	ENSBTAG00000018848	FBXL3	12	52466040	52477380	-	CODING	-0.58632	0.004759
ENSBTAT00000028307	ENSBTAG00000021245	SPRY1	17	34749102	34753222	-	CODING	-0.60062	0.004778
ENSBTAT00000021062	ENSBTAG00000015844	TFPI2	4	11035479	11039782	-	CODING	-0.42706	0.004842
ENSBTAT00000005857	ENSBTAG00000004463	KDELR1	18	55525319	55534438	-	CODING	-0.52993	0.004912
ENSBTAT00000009464	ENSBTAG00000007193	CCL16	19	14782701	14786690	+	CODING	-0.96775	0.004925
ENSBTAT00000002846	ENSBTAG00000027930	-	2	122169405	122170058	-	CODING	-0.62623	0.005076
ENSBTAT00000020065	ENSBTAG00000015074	PTGDS	11	106247577	106250629	-	CODING	-0.74206	0.005098
ENSBTAT00000013942	ENSBTAG00000010543	FGFR4	7	39936164	39946911	+	CODING	1.047726	0.005114
ENSBTAT00000011388	ENSBTAG00000008635	SULT1A1	25	26381308	26385156	-	CODING	-0.65344	0.005118
ENSBTAT00000045971	ENSBTAG00000032402	U1	3	22861821	22861984	-	CODING	-3.21506	0.005126
ENSBTAT00000040084	ENSBTAG00000014713	RARRES1	1	109661962	109704520	+	CODING	-3.16425	0.00516
ENSBTAT00000045370	ENSBTAG00000014401	SORBS3	8	70357693	70384759	+	CODING	-0.60716	0.005185
ENSBTAT00000015694	ENSBTAG00000011824	OGN	8	85453132	85468721	+	CODING	-0.47868	0.005191
ENSBTAT00000001893	ENSBTAG00000001444	TNXB	23	27085415	27136954	-	CODING	-1.36676	0.005234
ENSBTAT00000003794	ENSBTAG00000002922	-	29	17833137	17887917	+	CODING	-0.65877	0.005278
ENSBTAT00000045969	ENSBTAG00000018318	DNAJB5	8	59689172	59695910	+	CODING	-1.31454	0.005338
ENSBTAT00000039940	ENSBTAG00000027716	C7H19orf53	15	42332731	42333027	-	CODING	-1.43592	0.005364
ENSBTAT00000038591	ENSBTAG00000026963	NCALD	14	64875135	64898581	+	CODING	-0.96288	0.00538
ENSBTAT00000061057	ENSBTAG00000007732	ARPP21	22	9674589	9791425	+	CODING	0.666727	0.005435
ENSBTAT00000048322	ENSBTAG00000034077	ASIP	13	64234645	64239783	+	CODING	-0.76941	0.00545
ENSBTAT00000023197	ENSBTAG00000017455	ADAM-9	27	33816038	33896288	+	CODING	-0.63069	0.005486
ENSBTAT00000025683	ENSBTAG00000019290	PACSIN2	5	114357560	114475163	-	CODING	-0.70953	0.00549
ENSBTAT00000030941	ENSBTAG00000034185	EEF1A1	18	2952235	2954439	+	CODING	-0.71652	0.005552
ENSBTAT00000005333	ENSBTAG00000004082	-	6	71053202	71053458	-	CODING	-2.16542	0.005566
ENSBTAT00000025832	ENSBTAG00000005146	-	23	28330539	28334072	-	CODING	-0.46087	0.005643
ENSBTAT00000002946	ENSBTAG00000002283	FZD7	2	90987008	90989214	+	CODING	-0.73122	0.00566
ENSBTAT00000031069	ENSBTAG00000007784	NAA50	1	58847733	58879203	-	CODING	0.231495	0.005669
ENSBTAT00000028141	ENSBTAG00000021120	SMYD1	11	47799217	47848713	-	CODING	0.333538	0.005678
ENSBTAT00000009643	ENSBTAG00000007332	ATP5L	15	29521980	29530109	+	CODING	-0.26946	0.005692
ENSBTAT00000008517	ENSBTAG00000006499	PIP4K2B	19	39995184	40017502	-	CODING	0.37042	0.005754
ENSBTAT00000015345	ENSBTAG00000011547	KBTBD12	22	60249057	60288275	-	CODING	0.457284	0.005755
ENSBTAT00000017600	ENSBTAG00000013227	SNAI2	14	21577309	21580910	-	CODING	-1.2384	0.005764
ENSBTAT00000065783	ENSBTAG00000001141	ADAM17	11	87898074	87940943	+	CODING	6.059675	0.005837
ENSBTAT00000053957	ENSBTAG00000039764	IER5	16	63689680	63690654	+	CODING	-0.96651	0.005854
ENSBTAT00000017503	ENSBTAG00000003418	MSN	X	100070406	100162454	-	CODING	-0.40664	0.005861
ENSBTAT00000015210	ENSBTAG00000011446	SEMA7A	21	34626181	34651240	+	CODING	-0.9823	0.005922
ENSBTAT00000004594	ENSBTAG00000023823	CYCS	11	98339924	98340241	-	CODING	-0.16739	0.00593

ENSBTAT00000027511	ENSBTAG00000020645	GNAI2	22	50670852	50691007	-	CODING	-0.51792	0.005971
ENSBTAT00000020254	ENSBTAG00000015222	RSRP1	2	128302962	128306994	+	CODING	-0.99069	0.006104
ENSBTAT00000008327	ENSBTAG00000006346	DAP	20	62630506	62697437	+	CODING	-0.74941	0.006181
ENSBTAT00000013990	ENSBTAG00000010587	SH3BGRL	X	70399116	70527059	-	CODING	-0.64602	0.006204
ENSBTAT00000044557	ENSBTAG00000031441	FXYD5	18	46067593	46078935	+	CODING	-1.02049	0.006254
ENSBTAT00000018428	ENSBTAG00000013881	GJA4	3	111481702	111482589	-	CODING	-0.52414	0.006333
ENSBTAT00000024703	ENSBTAG00000018562	TMEM159	25	19185676	19201990	+	CODING	-0.4076	0.006341
ENSBTAT00000006383	ENSBTAG00000004855	PRDX6	16	56389804	56399714	+	CODING	-0.22594	0.006352
ENSBTAT00000026818	ENSBTAG00000020133	PPP3CC	8	70262316	70341783	+	CODING	-0.43859	0.006359
ENSBTAT00000024848	ENSBTAG00000018671	DCUN1D2	12	90680190	90695973	-	CODING	0.303922	0.006361
ENSBTAT00000029015	ENSBTAG00000021766	HBEGF	7	53216665	53227848	-	CODING	-0.65878	0.006395
ENSBTAT00000008357	ENSBTAG00000006367	CTGF	9	70873221	70876455	-	CODING	-0.44957	0.006396
ENSBTAT00000018500	ENSBTAG00000013926	FCGRT	18	56415700	56421855	+	CODING	-0.53408	0.006446
ENSBTAT00000000943	ENSBTAG00000000705	COX7A2L	11	24824245	24836596	-	CODING	-0.51879	0.006522
ENSBTAT00000045859	ENSBTAG00000000199	PDP1	14	72678594	72686964	-	CODING	0.472666	0.006562
ENSBTAT00000063333	ENSBTAG00000048119	-	6	108909400	108910432	-	CODING	-0.75662	0.006645
ENSBTAT00000006954	ENSBTAG00000005285	MRPS33	4	105101880	105110529	-	CODING	-0.30777	0.006663
ENSBTAT00000008467	ENSBTAG00000006457	AHSP	25	27801696	27805181	+	CODING	1.623105	0.00669
ENSBTAT00000043764	ENSBTAG00000002094	ATP5J2	25	37494182	37499276	+	CODING	-0.11306	0.006712
ENSBTAT00000020877	ENSBTAG00000015727	IFI47	7	41823772	41840054	-	CODING	-0.96167	0.006756
ENSBTAT00000064314	ENSBTAG00000011351	TMEM259	7	45141210	45144158	-	CODING	-0.73883	0.006854
ENSBTAT00000024516	ENSBTAG00000018426	MRRF	11	93149379	93158576	-	CODING	-0.77446	0.006856
ENSBTAT00000000389	ENSBTAG00000000305	LMO4	3	56832933	56846708	-	CODING	-0.36995	0.006868
ENSBTAT00000053533	ENSBTAG00000004136	NFE2L3	4	70211380	70214288	-	CODING	1.154405	0.006886
ENSBTAT00000015763	ENSBTAG00000024091	MALL	11	1747909	1780103	+	CODING	-1.11426	0.006975
ENSBTAT00000006285	ENSBTAG00000004787	METRNL	19	50243829	50258586	-	CODING	0.846902	0.007009
ENSBTAT00000030828	ENSBTAG00000003120	MGC139239	2	17084655	17448607	+	CODING	-1.38769	0.007071
ENSBTAT00000005102	ENSBTAG00000003907	TSPAN14	28	36077428	36109014	+	CODING	-0.52849	0.00709
ENSBTAT00000005060	ENSBTAG00000003877	ZCCHC24	28	35189622	35251005	-	CODING	0.433085	0.00719
ENSBTAT00000017759	ENSBTAG00000013347	DMPK	18	53760689	53769611	-	CODING	-0.36283	0.007299
ENSBTAT00000034553	ENSBTAG00000024815	ANKRD28	1	154443133	154568336	-	CODING	0.700262	0.00742
ENSBTAT00000025454	ENSBTAG00000019124	EIF4EBP2	28	26764785	26786028	+	CODING	0.266545	0.007551
ENSBTAT00000018584	ENSBTAG00000013982	UACA	10	17516736	17559258	-	CODING	1.740276	0.007564
ENSBTAT00000061244	ENSBTAG00000008077	SLC37A4	15	30122001	30127150	-	CODING	0.495317	0.007566
ENSBTAT00000002398	ENSBTAG00000001835	GJA1	9	30127786	30140793	-	CODING	-1.14884	0.007608
ENSBTAT00000028499	ENSBTAG00000021378	S100A13	3	16818414	16824125	+	CODING	-0.53755	0.007616
ENSBTAT00000014871	ENSBTAG00000011196	C1QB	2	130769172	130775911	-	CODING	-1.21719	0.007697
ENSBTAT00000012340	ENSBTAG00000009376	DPH1	19	23636131	23646338	+	CODING	0.828543	0.007721
ENSBTAT00000039259	ENSBTAG00000007718	TGIF1	24	37940878	37948732	+	CODING	-1.40783	0.007725
ENSBTAT00000063041	ENSBTAG00000047199	-	5	29600737	29601755	+	CODING	0.445358	0.007836
ENSBTAT00000025152	ENSBTAG00000018897	PSMD11	19	18035610	18064853	-	CODING	0.35429	0.007977
ENSBTAT00000023373	ENSBTAG00000017574	LMNA	3	14696144	14715262	-	CODING	-0.45119	0.008006
ENSBTAT00000028482	ENSBTAG00000021364	TNK2	1	71207395	71230157	-	CODING	0.803314	0.008017
ENSBTAT00000056799	ENSBTAG00000038889	RILP	19	23320981	23323996	-	CODING	0.663616	0.00804
ENSBTAT00000064362	ENSBTAG00000046319	NDUFA7	7	18191587	18198867	-	CODING	-0.31949	0.008158
ENSBTAT00000003716	ENSBTAG00000002863	ACAA2	24	49908126	49942827	-	CODING	-0.31306	0.008166
ENSBTAT00000007026	ENSBTAG00000005339	-	23	17255947	17269998	+	CODING	-0.99511	0.008174
ENSBTAT00000061638	ENSBTAG00000015361	CNTFR	8	77260361	77298847	-	CODING	0.729635	0.008194
ENSBTAT00000001839	ENSBTAG00000001400	AKT2	18	49904012	49950072	-	CODING	0.488529	0.008266
ENSBTAT00000024307	ENSBTAG00000018261	PDHX	15	66231837	66307403	+	CODING	0.292915	0.008317
ENSBTAT00000010926	ENSBTAG00000008300	FN1	2	103881402	103950562	-	CODING	-0.84923	0.008413
ENSBTAT00000005445	ENSBTAG00000004159	SIX2	11	27260475	27263699	-	CODING	-1.40896	0.008461
ENSBTAT00000052667	ENSBTAG00000010571	PPAT	6	73448407	73487539	-	CODING	0.530278	0.008702
ENSBTAT00000019149	ENSBTAG00000014401	SORBS3	8	70354893	70384759	+	CODING	-0.85894	0.0088
ENSBTAT00000025421	ENSBTAG00000019093	AMY2B	3	39926298	39948945	-	CODING	0.208337	0.008854
ENSBTAT00000006405	ENSBTAG00000004871	NDUFV2	24	41855938	41874838	+	CODING	-0.32166	0.008912
ENSBTAT00000011585	ENSBTAG00000008792	RNASE6	10	26402507	26404000	-	CODING	-1.00993	0.008954
ENSBTAT00000006307	ENSBTAG00000004803	NTAN1	25	13809385	13823824	+	CODING	-0.35582	0.009009
ENSBTAT00000064578	ENSBTAG00000048287	-	X	140308768	140315911	+	CODING	1.728456	0.009019
ENSBTAT00000004179	ENSBTAG00000003220	ACKR1	3	10647427	10648940	-	CODING	-1.1131	0.009117
ENSBTAT00000026323	ENSBTAG00000019754	PRKCDBP	15	47329716	47331334	+	CODING	-0.50646	0.009172
ENSBTAT00000023255	ENSBTAG00000017496	ATP5I	6	108851271	108852813	-	CODING	-0.27131	0.009187

ENSBTAT00000010568	ENSBTAG00000008033	PPP6C	11	96044189	96085315	-	CODING	0.362759	0.009339
ENSBTAT00000047947	ENSBTAG00000001212	SSR2	3	14808605	14815178	+	CODING	-0.33312	0.009446
ENSBTAT00000002556	ENSBTAG00000039682	MTUS1	27	18632852	18698056	+	CODING	-0.53245	0.009457
ENSBTAT00000065365	ENSBTAG00000003100	SMTN	17	72053880	72064312	+	CODING	-0.69618	0.009588
ENSBTAT00000019407	ENSBTAG00000014581	MLIP	23	6157657	6283325	-	CODING	0.288178	0.009591
ENSBTAT00000017904	ENSBTAG00000013463	DISP1	16	27161602	27209886	+	CODING	0.626234	0.009599
ENSBTAT00000063786	ENSBTAG00000002663	BCAS3	19	12346635	12392695	-	CODING	-4.41077	0.009616
ENSBTAT00000064442	ENSBTAG00000045582	ARPC5	16	66150415	66159807	-	CODING	-0.57921	0.009682
ENSBTAT00000025026	ENSBTAG00000018801	SNX3	9	42370448	42417660	+	CODING	-0.18855	0.009766
ENSBTAT00000021123	ENSBTAG00000015892	NDUFB4	1	65922482	65928617	+	CODING	-0.20423	0.009812
ENSBTAT00000033522	ENSBTAG00000024269	TGFBR3	3	51661004	51866717	+	CODING	-1.02946	0.009921
ENSBTAT00000023976	ENSBTAG00000018013	EMP3	18	55482512	55486850	+	CODING	-0.65436	0.009979
ENSBTAT00000010913	ENSBTAG00000008293	GPCPD1	13	48086530	48150177	-	CODING	-0.6306	0.010004
ENSBTAT00000011390	ENSBTAG00000008636	PDE4B	3	79286815	79734224	-	CODING	0.959787	0.010203
ENSBTAT00000009534	ENSBTAG00000007246	TMEM204	25	1194760	1207350	+	CODING	-0.90896	0.010228
ENSBTAT00000011800	ENSBTAG00000008964	DTD1	13	38909550	38989758	+	CODING	-0.93936	0.010274
ENSBTAT00000013685	ENSBTAG00000010368	TPST2	17	68445522	68499477	-	CODING	-0.74212	0.010286
ENSBTAT00000018261	ENSBTAG00000013745	ITGA5	5	25778012	25799053	+	CODING	-0.84881	0.010337
ENSBTAT00000034054	ENSBTAG00000024503	FCER1G	3	8305544	8308776	-	CODING	-1.08951	0.01035
ENSBTAT00000023313	ENSBTAG00000017537	PTGIS	13	78319020	78368752	-	CODING	-0.95149	0.010377
ENSBTAT00000022841	ENSBTAG00000017187	MGC142908	X	4995337	5043066	+	CODING	1.460733	0.010377
ENSBTAT00000022720	ENSBTAG00000017086	GRB10	4	5104899	5244348	+	CODING	0.425855	0.010429
ENSBTAT00000002867	ENSBTAG00000002215	GFPT2	7	772145	817756	+	CODING	-1.38758	0.010434
ENSBTAT00000007761	ENSBTAG00000005907	NDUFB6	8	11372938	11389199	+	CODING	-0.2187	0.010435
ENSBTAT00000065583	ENSBTAG00000047495	CD81	29	49842969	49848639	-	CODING	0.264902	0.010457
ENSBTAT00000044775	ENSBTAG00000031583	NUPR1L	25	28027757	28028658	-	CODING	-1.5192	0.010492
ENSBTAT00000052383	ENSBTAG00000001478	PPPDE1	16	33290070	33331173	-	CODING	-9.33293	0.010526
ENSBTAT00000015797	ENSBTAG00000011909	ACVR1	2	39287889	39361498	+	CODING	-0.59885	0.010539
ENSBTAT00000000233	ENSBTAG00000000199	PDP1	14	72678594	72686976	-	CODING	-6.32549	0.010575
ENSBTAT00000005522	ENSBTAG00000004211	TNFRSF1A	5	104402771	104415588	+	CODING	-0.72371	0.010756
ENSBTAT00000006666	ENSBTAG00000005057	FAM204A	26	38923063	38945878	-	CODING	-0.39285	0.01077
ENSBTAT00000023784	ENSBTAG00000017896	ST8SIA2	21	14879467	14949980	-	CODING	-1.69791	0.01094
ENSBTAT00000056227	ENSBTAG00000002964	TXLNB	9	77937305	77996890	-	CODING	0.291263	0.011091
ENSBTAT00000002506	ENSBTAG00000001926	RAD23B	8	98681864	98726100	+	CODING	0.156088	0.011149
ENSBTAT00000049002	ENSBTAG00000007732	ARPP21	22	9674589	9681167	+	CODING	1.217553	0.011221
ENSBTAT00000050296	ENSBTAG00000010442	PANK1	26	11280207	11335233	-	CODING	-1.17536	0.011343
ENSBTAT00000029167	ENSBTAG00000021880	ADCK3	16	30656001	30703396	+	CODING	0.164171	0.011358
ENSBTAT00000026548	ENSBTAG00000019927	CYB5R1	16	55553510	55558337	+	CODING	0.203854	0.011461
ENSBTAT00000003022	ENSBTAG00000002341	ETS1	29	32358407	32430222	-	CODING	-0.67515	0.011468
ENSBTAT00000065983	ENSBTAG00000046979	LGMN	21	57965316	58004541	-	CODING	0.51744	0.011543
ENSBTAT00000000259	ENSBTAG00000000215	GNB1	16	52158465	52182743	+	CODING	-0.92568	0.011598
ENSBTAT00000010606	ENSBTAG00000008063	PPARA	5	117151549	117233112	+	CODING	-0.7427	0.011608
ENSBTAT00000029301	ENSBTAG00000021976	NDUFS1	2	94893227	94922667	-	CODING	0.240198	0.011804
ENSBTAT00000063221	ENSBTAG00000047196	C9orf3	18	50611276	50611827	+	CODING	-0.54499	0.011886
ENSBTAT00000047200	ENSBTAG00000033221	CCDC152	20	31793082	31834092	-	CODING	-0.59406	0.01189
ENSBTAT00000043983	ENSBTAG00000031069	SNX24	7	32140926	32313012	-	CODING	-0.94093	0.011916
ENSBTAT00000009803	ENSBTAG00000007454	RPL10	X	40364802	40367164	+	CODING	-0.12252	0.01193
ENSBTAT00000021848	ENSBTAG00000016429	TMEM205	7	16971974	16974656	-	CODING	0.679986	0.011947
ENSBTAT00000037914	ENSBTAG00000026624	LYRM5	5	85210770	85217660	-	CODING	-0.35407	0.011964
ENSBTAT00000026769	ENSBTAG00000020093	CUL4A	12	90562966	90589009	+	CODING	0.257671	0.011999
ENSBTAT00000012886	ENSBTAG00000009770	GPD2	2	39765206	39912916	-	CODING	0.673916	0.01206
ENSBTAT00000027409	ENSBTAG00000020569	CACNA2D1	4	38712468	38856748	+	CODING	0.393874	0.012069
ENSBTAT00000011377	ENSBTAG00000024450	MAP2K2	7	21132389	21153974	+	CODING	-0.30927	0.012198
ENSBTAT00000024312	ENSBTAG00000018267	TRIM54	11	72327351	72348528	-	CODING	-0.14709	0.012264
ENSBTAT00000014324	ENSBTAG00000010813	-	14	17228479	17229057	-	CODING	-0.65348	0.012281
ENSBTAT00000016194	ENSBTAG00000012208	-	23	27689369	27692407	+	CODING	-1.11928	0.012413
ENSBTAT00000021425	ENSBTAG00000016093	PLP2	X	92294752	92297791	+	CODING	-0.3936	0.012425
ENSBTAT00000027115	ENSBTAG00000020345	CNN3	3	48763975	48794136	+	CODING	-0.63254	0.012479
ENSBTAT00000063482	ENSBTAG00000021048	ADM	15	42911789	42913325	-	CODING	-1.09374	0.01248
ENSBTAT00000045376	ENSBTAG00000011588	RNF123	22	51054914	51078183	-	CODING	0.452586	0.012493
ENSBTAT00000018239	ENSBTAG00000013724	ATG4A	X	60973684	61051097	+	CODING	-0.48086	0.012587
ENSBTAT00000006653	ENSBTAG00000005043	TIMP1	X	91232235	91236073	+	CODING	-0.73467	0.012587

ENSBTAT00000052306	ENSBTAG00000004271	UBAP2L	3	16318128	16358192	-	CODING	0.784782	0.012673
ENSBTAT00000034186	ENSBTAG00000024561	H3F3A	16	29832591	29840787	+	CODING	-0.21181	0.012753
ENSBTAT00000042645	ENSBTAG00000030209	ARHGDIA	19	51633694	51637708	+	CODING	-0.36096	0.012837
ENSBTAT00000006694	ENSBTAG00000030340	-	2	134294882	134340689	+	CODING	1.640094	0.012917
ENSBTAT00000017090	ENSBTAG00000012858	NT5C2	26	23983206	24080557	-	CODING	-0.58519	0.012964
ENSBTAT00000045685	ENSBTAG00000012112	SLC7A6	18	35763843	35794542	+	CODING	1.259738	0.013038
ENSBTAT00000035225	ENSBTAG00000004910	SLC25A11	19	27089299	27092026	+	CODING	0.217529	0.013075
ENSBTAT00000005786	ENSBTAG00000004413	RHOBTB3	7	97350408	97406000	+	CODING	-0.48197	0.013222
ENSBTAT00000008749	ENSBTAG00000006661	RALA	4	81919125	81929351	-	CODING	-0.54352	0.013242
ENSBTAT00000023810	ENSBTAG00000031752	TMEM256	19	27714471	27715619	-	CODING	-0.566	0.013305
ENSBTAT00000010372	ENSBTAG00000007884	SREBF1	19	35234637	35250672	+	CODING	-0.43308	0.013307
ENSBTAT00000044159	ENSBTAG00000031184	CDKN1C	29	49368787	49370785	+	CODING	-0.68419	0.01334
ENSBTAT00000017002	ENSBTAG00000012797	DCUN1D1	1	84656319	84689038	+	CODING	0.252326	0.013404
ENSBTAT00000046984	ENSBTAG00000005163	S100A1	3	16812602	16816588	-	CODING	-2.2421	0.013424
ENSBTAT00000003595	ENSBTAG00000002770	TCN2	17	71711883	71727449	+	CODING	-0.47882	0.013502
ENSBTAT00000007943	ENSBTAG00000006045	P4HB	19	51643765	51653754	+	CODING	-0.2787	0.013518
ENSBTAT00000001780	ENSBTAG00000001349	KIF1C	19	27014446	27043780	-	CODING	0.169777	0.013527
ENSBTAT00000052354	ENSBTAG00000008635	SULT1A1	25	26381342	26383753	-	CODING	-0.64624	0.013537
ENSBTAT00000016153	ENSBTAG00000012177	SNRPD2	18	53681070	53683928	-	CODING	-0.31448	0.013564
ENSBTAT00000015460	ENSBTAG00000011640	PPAP2B	3	90185369	90272863	+	CODING	-0.59772	0.013626
ENSBTAT00000000453	ENSBTAG00000000347	RHOG	15	52061683	52075146	+	CODING	-0.57568	0.013681
ENSBTAT00000002532	ENSBTAG00000001948	VTI1B	10	80113761	80131964	-	CODING	-0.42719	0.013752
ENSBTAT00000021860	ENSBTAG00000016441	ZNF622	20	56767512	56781229	+	CODING	-0.63649	0.013871
ENSBTAT00000024194	ENSBTAG00000018181	CCDC8	18	54124479	54127099	+	CODING	0.941166	0.013964
ENSBTAT00000044474	ENSBTAG00000003908	MAPRE1	13	62728053	62754549	+	CODING	-0.42056	0.014162
ENSBTAT00000046225	ENSBTAG00000023891	RBM20	26	31465604	31678528	+	CODING	0.797573	0.014436
ENSBTAT00000063958	ENSBTAG00000045729	SMIM20	16	68078643	68078846	+	CODING	-0.5855	0.014518
ENSBTAT00000001069	ENSBTAG00000000808	COX14	5	29952087	29956382	-	CODING	-0.26245	0.01454
ENSBTAT00000040082	ENSBTAG00000016648	BSG	7	44816940	44823914	+	CODING	-0.17398	0.014562
ENSBTAT00000065986	ENSBTAG00000046794	-	X	104334442	104334810	-	CODING	-0.51306	0.014675
ENSBTAT00000007861	ENSBTAG00000005990	S1PR1	3	42184097	42188752	-	CODING	-0.36075	0.014751
ENSBTAT00000001253	ENSBTAG00000000948	RAB2A	14	27864735	27937015	+	CODING	0.181378	0.014763
ENSBTAT00000014091	ENSBTAG00000010661	DPYSL3	7	60588659	60703487	-	CODING	-0.73248	0.014976
ENSBTAT00000007519	ENSBTAG00000005718	PLIN2	8	25129168	25137851	+	CODING	-1.97302	0.014997
ENSBTAT00000003826	ENSBTAG00000002939	FURIN	21	22206517	22213761	-	CODING	0.500272	0.015238
ENSBTAT00000015485	ENSBTAG00000011662	SOX18	13	54295399	54296819	+	CODING	-0.97629	0.015518
ENSBTAT00000016130	ENSBTAG00000012159	CNBP	22	59738963	59740707	-	CODING	0.110119	0.015596
ENSBTAT00000054165	ENSBTAG00000034373	CDH13	18	9512739	10162782	+	CODING	-0.35523	0.015605
ENSBTAT00000004922	ENSBTAG00000006876	PMPCB	4	44755331	44769491	+	CODING	0.387672	0.01562
ENSBTAT00000064445	ENSBTAG00000046838	-	X	115892940	116081425	+	CODING	0.640887	0.015708
ENSBTAT00000014749	ENSBTAG00000011105	SLC38A2	5	34028553	34042996	+	CODING	0.237638	0.015744
ENSBTAT00000014865	ENSBTAG00000011193	C1QC	2	130783987	130788357	-	CODING	-1.66768	0.015806
ENSBTAT00000047359	ENSBTAG00000010526	PPAP2A	20	23658802	23793336	+	CODING	-0.24632	0.015911
ENSBTAT00000029271	ENSBTAG00000021955	NPC2	10	86170653	86179237	-	CODING	-0.41648	0.015934
ENSBTAT00000009303	ENSBTAG00000007077	ABHD1	11	72497319	72504279	-	CODING	-1.62141	0.016039
ENSBTAT00000015117	ENSBTAG00000011381	SLC30A3	11	72364682	72373190	+	CODING	-2.75353	0.016094
ENSBTAT00000000179	ENSBTAG00000000156	LGALSL	11	62812719	62817829	+	CODING	-0.47948	0.016118
ENSBTAT00000022411	ENSBTAG00000016846	YWHAB	13	74018886	74040819	+	CODING	-0.40313	0.016176
ENSBTAT00000023247	ENSBTAG00000017492	PCMTD1	14	22669363	22717576	-	CODING	0.2417	0.016197
ENSBTAT00000000648	ENSBTAG00000000507	NR4A1	5	27977007	27992869	-	CODING	0.176186	0.016215
ENSBTAT00000019242	ENSBTAG00000014471	-	8	101361501	101406330	+	CODING	-0.49663	0.016215
ENSBTAT00000002878	ENSBTAG00000002226	FBXO31	18	12974880	13009289	-	CODING	0.4015	0.016286
ENSBTAT00000018869	ENSBTAG00000014191	QSOX1	16	62804447	62845817	+	CODING	-0.93763	0.016323
ENSBTAT00000014894	ENSBTAG00000011215	ACTN4	18	48668482	48741185	+	CODING	-0.40499	0.016357
ENSBTAT00000033359	ENSBTAG00000031723	RPL6	17	64115683	64120590	+	CODING	-0.293	0.016363
ENSBTAT00000001770	ENSBTAG00000001344	METTL21E	12	83099334	83115415	+	CODING	-0.6712	0.016411
ENSBTAT00000007391	ENSBTAG00000005622	LITAF	25	10288891	10325021	-	CODING	-0.75919	0.01656
ENSBTAT00000066025	ENSBTAG00000046323	-	18	25717808	25721329	-	CODING	-0.91692	0.016631
ENSBTAT00000042718	ENSBTAG00000014906	VCAN	7	85666058	85782896	+	CODING	-1.56793	0.016717
ENSBTAT00000027632	ENSBTAG00000020735	-	25	16556655	16625702	-	CODING	0.544563	0.016775
ENSBTAT00000026460	ENSBTAG00000034366	RGS2	16	13041921	13045220	-	CODING	0.652877	0.016822
ENSBTAT00000017023	ENSBTAG00000012816	-	2	43381652	43382632	-	CODING	-0.27736	0.016905

ENSBTAT00000027670	ENSBTAG00000020764	CNN2	7	45159981	45162941	+	CODING	-0.82385	0.016956
ENSBTAT00000053057	ENSBTAG00000032021	RALB	2	72372371	72430777	+	CODING	-0.50325	0.016993
ENSBTAT00000032213	ENSBTAG00000012881	EIF4G1	1	83466847	83484338	-	CODING	0.261435	0.017091
ENSBTAT00000017780	ENSBTAG00000013367	CLN1	3	106622151	106638462	+	CODING	-0.7433	0.017095
ENSBTAT00000024105	ENSBTAG00000018112	TBL1X	X	144430889	144489539	-	CODING	-0.5441	0.017256
ENSBTAT00000012170	ENSBTAG00000009233	RAC1	25	38832485	38841319	+	CODING	-0.20352	0.017262
ENSBTAT00000004198	ENSBTAG00000003238	MEOX2	4	23943520	24019359	-	CODING	-0.48566	0.017325
ENSBTAT00000029020	ENSBTAG00000021771	PTTG1IP	1	145057828	145075459	-	CODING	-0.54665	0.01735
ENSBTAT00000023734	ENSBTAG00000017855	ITM2C	2	119476496	119490380	+	CODING	-0.79255	0.017379
ENSBTAT00000003190	ENSBTAG00000002457	SEC61B	8	64722966	64731269	+	CODING	-0.54929	0.017515
ENSBTAT00000046362	ENSBTAG00000002608	42980	3	120968024	120988257	+	CODING	-0.47201	0.017658
ENSBTAT00000043544	ENSBTAG00000030805	SHISA4	16	49618802	49620917	+	CODING	0.139423	0.01768
ENSBTAT00000038491	ENSBTAG00000000754	PPP2R5A	16	73077108	73151753	-	CODING	-0.44226	0.017706
ENSBTAT00000064052	ENSBTAG00000027676	IL18BP	15	52417129	52418796	+	CODING	-10.1074	0.017766
ENSBTAT00000023407	ENSBTAG00000017604	RAB13	3	16511100	16515182	+	CODING	-0.54061	0.017814
ENSBTAT00000025878	ENSBTAG00000019427	KIAA0232	6	118129452	118178266	+	CODING	0.48011	0.017875
ENSBTAT00000029257	ENSBTAG00000021945	NID2	10	44894657	44986659	-	CODING	-0.73459	0.017911
ENSBTAT00000014571	ENSBTAG00000010976	KANK1	8	44046426	44076904	-	CODING	0.545625	0.017917
ENSBTAT00000003415	ENSBTAG00000002633	42987	19	55118592	55153750	-	CODING	-0.57267	0.01799
ENSBTAT00000021570	ENSBTAG00000016206	MAOA	X	105380194	105462564	-	CODING	-0.79222	0.017997
ENSBTAT00000034695	ENSBTAG00000024909	H3F3A	19	56453856	56455637	+	CODING	-0.30472	0.018146
ENSBTAT00000065275	ENSBTAG00000000895	TAF9B	X	79269814	79277533	+	CODING	0.816635	0.018168
ENSBTAT00000046197	ENSBTAG00000000223	PPM1B	11	26402682	26426120	+	CODING	0.290999	0.01833
ENSBTAT00000032460	ENSBTAG00000001852	BREH1	18	24827700	24854528	+	CODING	-0.83807	0.018591
ENSBTAT00000036029	ENSBTAG00000010106	CCND3	23	15707556	15714372	-	CODING	-0.69924	0.018607
ENSBTAT00000056395	ENSBTAG00000038630	KLHL34	X	128884989	128886938	+	CODING	-0.71402	0.018622
ENSBTAT00000060994	ENSBTAG00000013108	HK2	11	9723269	9766920	-	CODING	0.624188	0.01868
ENSBTAT00000026999	ENSBTAG00000020263	TIMP4	22	57602210	57609085	+	CODING	-0.44392	0.018698
ENSBTAT00000063543	ENSBTAG00000045925	CITED1	X	83497735	83501893	+	CODING	2.336507	0.01877
ENSBTAT00000028640	ENSBTAG00000004288	GSTA4	23	24963227	24977317	-	CODING	0.437682	0.01877
ENSBTAT00000027662	ENSBTAG00000020757	PCBP2	5	26702879	26723947	-	CODING	-0.18937	0.018841
ENSBTAT00000018391	ENSBTAG00000013834	-	7	18960639	18964880	+	CODING	-0.34292	0.018872
ENSBTAT00000023284	ENSBTAG00000017512	MAPT	19	46524375	46643750	+	CODING	-1.42244	0.018954
ENSBTAT00000007150	ENSBTAG00000005432	CAPG	11	49423731	49438680	+	CODING	-1.40319	0.019043
ENSBTAT00000007933	ENSBTAG00000006039	ARHGDIB	5	95376923	95395815	+	CODING	-0.44787	0.019117
ENSBTAT00000012704	ENSBTAG00000009641	MTHFD1	10	76858872	76919406	+	CODING	0.430208	0.019151
ENSBTAT00000050390	ENSBTAG00000006242	USP9X	X	107783133	107862518	-	CODING	0.382111	0.0192
ENSBTAT00000025870	ENSBTAG00000019419	NDUFS7	7	45427304	45433438	+	CODING	0.203179	0.019219
ENSBTAT00000017461	ENSBTAG00000013136	EFCAB11	10	102666363	102692145	-	CODING	-1.05352	0.019286
ENSBTAT00000049099	ENSBTAG00000034662	-	9	18473883	18475244	+	CODING	0.267269	0.01931
ENSBTAT00000025522	ENSBTAG00000019177	BIN1	2	5350654	5407851	+	CODING	0.140769	0.019344
ENSBTAT00000016242	ENSBTAG00000012244	TUBA1B	5	30864529	30868119	+	CODING	-0.20786	0.019582
ENSBTAT00000022146	ENSBTAG00000016650	TIGAR	5	106223071	106238040	-	CODING	-1.33244	0.01969
ENSBTAT00000021451	ENSBTAG00000019386	BOLA-NC	23	28355361	28358332	-	CODING	-0.55483	0.019719
ENSBTAT00000011605	ENSBTAG00000008812	PFDN4	13	82399773	82402817	+	CODING	-0.25175	0.01992
ENSBTAT00000052919	ENSBTAG00000018732	HSPA12B	13	51901885	51918849	-	CODING	-0.66799	0.019923
ENSBTAT00000015381	ENSBTAG00000011578	CD44	15	66454331	66541790	+	CODING	-0.95988	0.019956
ENSBTAT00000019527	ENSBTAG00000014667	PAIP2B	11	13393546	13397285	+	CODING	-0.36099	0.019963
ENSBTAT00000015092	ENSBTAG00000011358	IER3	23	28088456	28089722	+	CODING	-0.47203	0.019969
ENSBTAT00000035874	ENSBTAG00000022590	BOLA	23	27863067	27867532	+	CODING	-0.46867	0.02005
ENSBTAT00000004338	ENSBTAG00000003342	AMD1	9	39903108	39924416	-	CODING	-0.51546	0.020075
ENSBTAT00000055460	ENSBTAG00000039684	PTRF	19	43154126	43162165	-	CODING	0.456324	0.020305
ENSBTAT00000024587	ENSBTAG00000018474	IL6R	3	16179345	16231747	-	CODING	0.536453	0.020346
ENSBTAT00000037230	ENSBTAG00000026249	-	5	35174130	35174846	-	CODING	1.284541	0.020395
ENSBTAT00000025002	ENSBTAG00000040398	MGC148692	6	73368358	73396166	+	CODING	1.17615	0.020413
ENSBTAT00000005626	ENSBTAG00000021372	42989	6	93528980	93628589	+	CODING	0.539916	0.020423
ENSBTAT00000014593	ENSBTAG00000010992	CTSH	21	25675908	25696632	-	CODING	-0.74145	0.020535
ENSBTAT00000002648	ENSBTAG00000002044	SIRT3	11	107167131	107189741	-	CODING	-0.87705	0.020538
ENSBTAT00000030192	ENSBTAG00000022314	EMD	X	40341311	40343613	+	CODING	-0.51268	0.020538
ENSBTAT00000046544	ENSBTAG00000032774	C28H10ORF1	28	41912322	41914565	+	CODING	-0.3148	0.020681
ENSBTAT00000031716	ENSBTAG00000021204	TES	4	52477241	52532254	-	CODING	-0.79324	0.020738
ENSBTAT00000014028	ENSBTAG00000010611	OCIAD1	6	69144344	69167282	+	CODING	0.553459	0.02074

ENSBTAT00000017179	ENSBTAG00000012929	CEP85L	9	32779814	32900894	+	CODING	0.649019	0.020774
ENSBTAT00000022154	ENSBTAG00000016658	ABHD4	10	22045427	22056366	-	CODING	-0.45738	0.020979
ENSBTAT00000032574	ENSBTAG00000030274	CMC2	7	81396101	81396804	+	CODING	-0.3653	0.02105
ENSBTAT00000038641	ENSBTAG00000026994	C2H2orf88	2	6038403	6113008	-	CODING	-2.52745	0.021254
ENSBTAT00000064356	ENSBTAG00000048237	FGF9	12	35630411	35647612	-	CODING	-1.56736	0.021352
ENSBTAT00000021337	ENSBTAG00000016032	-	11	44707028	44711889	-	CODING	0.607993	0.021408
ENSBTAT00000009523	ENSBTAG00000007239	TSG-6	2	44850892	44867293	-	CODING	-1.50729	0.021505
ENSBTAT00000001440	ENSBTAG00000001086	FHL2	11	9253234	9300671	-	CODING	-1.85624	0.021658
ENSBTAT00000038517	ENSBTAG00000047248	-	3	63013693	63014043	-	CODING	2.399051	0.021679
ENSBTAT00000025696	ENSBTAG00000019298	STRADB	2	90432216	90456178	+	CODING	-0.27299	0.021686
ENSBTAT00000022774	ENSBTAG00000017135	CTSS	3	20024302	20047228	+	CODING	-0.65695	0.021712
ENSBTAT00000063435	ENSBTAG00000045772	-	9	95165926	95166855	-	CODING	-0.32019	0.021713
ENSBTAT00000065587	ENSBTAG00000047374	-	5	56217962	56220534	+	CODING	-0.88424	0.021742
ENSBTAT00000049295	ENSBTAG00000034824	-	20	2985749	2986486	-	CODING	-0.63917	0.021771
ENSBTAT00000027001	ENSBTAG00000020262	PSME4	11	36424173	36524445	-	CODING	0.281962	0.021811
ENSBTAT00000007442	ENSBTAG00000005664	YWHAE	19	23086308	23121103	-	CODING	0.11709	0.021813
ENSBTAT00000015348	ENSBTAG00000011548	AMPD1	3	28756908	28768496	+	CODING	0.185767	0.021842
ENSBTAT00000010674	ENSBTAG00000008114	CD99	X	140188320	140215657	-	CODING	-0.36172	0.021888
ENSBTAT00000008325	ENSBTAG00000006345	-	15	52184590	52213125	-	CODING	-0.99527	0.02189
ENSBTAT00000001034	ENSBTAG00000000778	-	23	17867543	17873174	+	CODING	-0.15331	0.021901
ENSBTAT00000006754	ENSBTAG00000005119	PSMD1	2	119675723	119756976	+	CODING	0.30682	0.021912
ENSBTAT00000021532	ENSBTAG00000037640	PID1	2	117836412	118104185	-	CODING	-0.66825	0.021923
ENSBTAT00000005377	ENSBTAG00000004115	MYLIP	23	40770315	40790973	-	CODING	-0.56953	0.021947
ENSBTAT00000009992	ENSBTAG00000007596	GEM	14	72386751	72399660	+	CODING	-1.01737	0.022039
ENSBTAT00000018092	ENSBTAG00000013607	WDR1	6	110136880	110178474	-	CODING	-0.41647	0.022141
ENSBTAT00000056243	ENSBTAG00000040602	-	2	131410548	131414758	-	CODING	-0.23188	0.022194
ENSBTAT00000064839	ENSBTAG00000045822	-	15	74915626	74916613	+	CODING	-0.99621	0.022246
ENSBTAT00000064054	ENSBTAG00000006027	USP34	11	59791217	59987854	-	CODING	-7.91213	0.022278
ENSBTAT00000026218	ENSBTAG00000019672	-	23	40609760	40611238	+	CODING	0.621962	0.022391
ENSBTAT00000032674	ENSBTAG00000021306	CHRDL2	15	54785596	54819153	-	CODING	-1.77403	0.022413
ENSBTAT00000065236	ENSBTAG00000046309	-	10	73129829	73133979	-	CODING	0.459833	0.022529
ENSBTAT00000009715	ENSBTAG00000007390	VAT1	19	43687403	43694870	-	CODING	-0.55064	0.02257
ENSBTAT00000003789	ENSBTAG00000002917	PRKAG2	4	114885501	114925565	-	CODING	-0.80864	0.022714
ENSBTAT00000008607	ENSBTAG00000006550	GABARAPL2	18	2907580	2917649	+	CODING	-0.25769	0.022732
ENSBTAT00000009897	ENSBTAG00000007520	MAPRE2	24	22193149	22306884	-	CODING	0.195049	0.022803
ENSBTAT00000049975	ENSBTAG00000033389	-	25	1167351	1168312	+	CODING	-0.85496	0.022834
ENSBTAT00000020501	ENSBTAG00000015426	PDLIM4	7	23443528	23457942	-	CODING	-1.29344	0.022897
ENSBTAT00000001061	ENSBTAG00000000802	LYVE1	15	42678184	42692464	+	CODING	-1.08004	0.022904
ENSBTAT00000000795	ENSBTAG00000000603	JAM2	1	10083035	10165613	-	CODING	-0.49841	0.022969
ENSBTAT00000002033	ENSBTAG00000001553	HNRNPA1	5	25939737	25944246	-	CODING	-0.24348	0.022991
ENSBTAT00000025350	ENSBTAG00000019044	BAIAP2	19	52198617	52263263	-	CODING	1.001104	0.023007
ENSBTAT00000016768	ENSBTAG00000012634	NDUFB7	7	12325362	12329750	+	CODING	-0.19795	0.023032
ENSBTAT00000061860	ENSBTAG00000044427	RNaseP_nuc	10	26814256	26814588	+	CODING	-0.68352	0.023035
ENSBTAT00000011643	ENSBTAG00000008842	JPH1	14	39633189	39725607	-	CODING	0.170263	0.023055
ENSBTAT00000005327	ENSBTAG00000004077	YWHAG	25	34884283	34906639	-	CODING	0.148632	0.023058
ENSBTAT00000012357	ENSBTAG00000009389	HNRNPH1	7	1593463	1602463	+	CODING	0.148176	0.023095
ENSBTAT00000064621	ENSBTAG00000046307	-	14	20738814	20740407	+	CODING	-0.3536	0.023326
ENSBTAT00000011200	ENSBTAG00000008497	RGS14	7	40214182	40227956	+	CODING	0.95574	0.023347
ENSBTAT00000052079	ENSBTAG00000000484	HYAL2	22	50593627	50597453	+	CODING	-1.22086	0.023396
ENSBTAT00000022939	ENSBTAG00000017258	ACSL3	2	111797170	111887224	+	CODING	-0.56679	0.023543
ENSBTAT00000028277	ENSBTAG00000021223	CRY2	15	76753525	76790450	+	CODING	0.712001	0.023681
ENSBTAT00000064420	ENSBTAG00000047747	NDFIP1	7	55061328	55085013	+	CODING	-0.38584	0.023875
ENSBTAT00000065901	ENSBTAG00000047491	CACNA1S	16	81472038	81527931	-	CODING	0.27752	0.024048
ENSBTAT00000029307	ENSBTAG00000021978	PARVB	5	115517820	115572329	+	CODING	-0.24861	0.024085
ENSBTAT00000036271	ENSBTAG00000012632	TECR	7	12329820	12357348	-	CODING	0.210176	0.024106
ENSBTAT00000031313	ENSBTAG00000023039	-	28	31044242	31057065	-	CODING	0.984166	0.024162
ENSBTAT00000024019	ENSBTAG00000018045	SLC12A4	18	35547822	35569646	-	CODING	-0.59621	0.024165
ENSBTAT00000009604	ENSBTAG00000007300	FHL3	3	108547857	108555472	+	CODING	-0.12473	0.024341
ENSBTAT00000048749	ENSBTAG00000034411	SHISA3	6	62877287	62880849	+	CODING	-1.64133	0.024426
ENSBTAT00000002891	ENSBTAG00000046814	ACOT2	10	85358158	85365337	+	CODING	-1.11947	0.024488
ENSBTAT00000005375	ENSBTAG00000004112	GAMT	7	45433859	45436865	-	CODING	0.301473	0.024624
ENSBTAT00000025633	ENSBTAG00000019253	RPL36A	X	55210108	55213506	+	CODING	-0.32049	0.024719

ENSBTAT00000022858	ENSBTAG00000017200	RHOJ	10	75783882	75877395	+	CODING	-0.54716	0.024788
ENSBTAT00000016850	ENSBTAG00000012684	CCL19	8	77394838	77396746	-	CODING	-2.75587	0.024797
ENSBTAT00000035455	ENSBTAG00000015988	MYH11	25	14218281	14343745	-	CODING	-0.57093	0.02493
ENSBTAT00000013707	ENSBTAG00000010386	ARPC2	2	106978526	107006143	+	CODING	-0.2621	0.024956
ENSBTAT00000004897	ENSBTAG00000003764	AKIRIN2	9	63127339	63150180	+	CODING	-0.26906	0.025032
ENSBTAT00000004739	ENSBTAG00000003634	CISD1	26	742617	760794	-	CODING	0.292276	0.025135
ENSBTAT00000049219	ENSBTAG00000004861	EIF3F	15	45130568	45138822	-	CODING	-0.25538	0.025202
ENSBTAT00000025388	ENSBTAG00000019069	SYNGR2	19	54611056	54614829	-	CODING	0.204112	0.025267
ENSBTAT00000053197	ENSBTAG00000018749	NUDT16	1	140044681	140046241	-	CODING	-2.16758	0.02534
ENSBTAT00000002948	ENSBTAG00000002282	EIF1	19	42545777	42548334	+	CODING	-0.091	0.025478
ENSBTAT00000022510	ENSBTAG00000016924	CAP2	23	39646543	39781557	-	CODING	0.162532	0.025592
ENSBTAT00000000662	ENSBTAG00000000512	SNTA1	13	63408786	63490256	-	CODING	0.274155	0.025631
ENSBTAT00000007399	ENSBTAG00000005628	CD52	2	127396373	127397972	-	CODING	-1.30994	0.025643
ENSBTAT00000008177	ENSBTAG00000006227	IDH3A	21	31063699	31079936	+	CODING	0.232063	0.025674
ENSBTAT00000063294	ENSBTAG00000046484	AIF1L	11	101335452	101343787	+	CODING	-0.80905	0.025778
ENSBTAT00000057439	ENSBTAG00000024608	-	13	59957517	59958130	+	CODING	-0.87758	0.025905
ENSBTAT00000039835	ENSBTAG00000014463	CAMK2D	6	12965129	13272298	+	CODING	0.314102	0.025965
ENSBTAT00000047798	ENSBTAG00000017189	POLE4	11	9683454	9694986	-	CODING	-1.10246	0.026115
ENSBTAT00000038684	ENSBTAG00000027020	COL5A2	2	7139738	7298551	+	CODING	-0.82401	0.026161
ENSBTAT00000002084	ENSBTAG00000001592	INSIG1	4	117906798	117920021	+	CODING	-1.02014	0.026284
ENSBTAT00000022232	ENSBTAG00000016724	NPEPL1	13	58207245	58222951	-	CODING	-0.36886	0.026539
ENSBTAT00000031148	ENSBTAG00000022920	RNF128	X	56840833	56905852	-	CODING	0.432786	0.02692
ENSBTAT00000061385	ENSBTAG00000039682	MTUS1	27	18574170	18698056	+	CODING	0.236706	0.027024
ENSBTAT00000013247	ENSBTAG00000027787	-	18	65621142	65621509	+	CODING	-0.24639	0.027039
ENSBTAT00000032427	ENSBTAG00000000605	ATP5J	1	10069680	10076995	+	CODING	-0.27615	0.027086
ENSBTAT00000052022	ENSBTAG00000040116	H1FX	22	59841218	59841862	-	CODING	-0.33805	0.027169
ENSBTAT00000019091	ENSBTAG00000014358	EVA1B	3	110118545	110120286	+	CODING	-1.46519	0.0272
ENSBTAT00000023720	ENSBTAG00000017845	BAG1	8	76352438	76363634	-	CODING	-0.38924	0.02731
ENSBTAT00000012183	ENSBTAG00000009246	DPY30	11	14683785	14696398	-	CODING	-0.22806	0.027413
ENSBTAT00000014476	ENSBTAG00000010899	TIMP2	19	54079297	54131052	+	CODING	-0.3069	0.027552
ENSBTAT00000022302	ENSBTAG00000016768	SCN3B	15	34769329	34791462	-	CODING	-2.41292	0.027639
ENSBTAT00000025198	ENSBTAG00000018921	USP19	22	51470661	51482027	+	CODING	0.433402	0.027653
ENSBTAT00000002341	ENSBTAG00000001788	BOVAGGRUS	16	55407771	55442826	-	CODING	-1.05592	0.027654
ENSBTAT00000011732	ENSBTAG00000008913	TMEM98	19	17622149	17634850	-	CODING	-1.32091	0.027821
ENSBTAT00000023684	ENSBTAG00000017804	BNIP3	26	51353458	51357472	-	CODING	0.174935	0.027841
ENSBTAT00000005137	ENSBTAG00000003935	RECS1	2	107023827	107041372	-	CODING	-0.27036	0.027865
ENSBTAT00000002986	ENSBTAG00000002315	RNF34	17	56021830	56042534	-	CODING	-0.38508	0.027934
ENSBTAT00000015277	ENSBTAG00000011494	PYGL	10	43800152	43840994	-	CODING	-0.83497	0.027959
ENSBTAT00000018505	ENSBTAG00000013930	ATP5C1	13	16171111	16187450	-	CODING	-0.17647	0.028041
ENSBTAT00000007922	ENSBTAG00000006029	OGDH	4	77458516	77512436	-	CODING	0.221428	0.028093
ENSBTAT00000004145	ENSBTAG00000003191	FSCN1	25	39292721	39302192	-	CODING	-0.88278	0.028098
ENSBTAT00000001575	ENSBTAG00000038238	-	16	60651006	60651572	+	CODING	-2.49125	0.028128
ENSBTAT00000015882	ENSBTAG00000011970	FNIP2	17	41065498	41144357	-	CODING	-1.26754	0.028203
ENSBTAT00000013104	ENSBTAG00000009928	MLST8	25	1738280	1742107	+	CODING	-6.2493	0.02862
ENSBTAT00000061487	ENSBTAG00000044073	CD248	29	45016284	45018614	-	CODING	-0.87542	0.028654
ENSBTAT00000038564	ENSBTAG00000015604	ZNF385A	5	25807926	25823618	+	CODING	-0.94586	0.028714
ENSBTAT00000011106	ENSBTAG00000008441	SOCS3	19	54458856	54459555	+	CODING	-0.92854	0.028837
ENSBTAT00000009395	ENSBTAG00000007139	WSB2	17	59422963	59437371	+	CODING	-0.29799	0.02886
ENSBTAT00000045695	ENSBTAG00000011823	CLSTN1	16	44633323	44671659	+	CODING	-0.5991	0.028915
ENSBTAT00000014061	ENSBTAG00000010637	ARSG	19	62368464	62401915	+	CODING	0.739386	0.028979
ENSBTAT00000032142	ENSBTAG00000038131	ABCC5	1	83736248	83764147	+	CODING	-0.47539	0.029014
ENSBTAT00000031330	ENSBTAG00000008827	SPOCK2	28	28304694	28329730	-	CODING	0.52392	0.029021
ENSBTAT00000024292	ENSBTAG00000018249	KCNN3	3	15781068	15948350	+	CODING	0.427731	0.02948
ENSBTAT00000009864	ENSBTAG00000007499	HNRPH2	X	55234948	55241418	+	CODING	0.334178	0.029586
ENSBTAT00000044340	ENSBTAG00000031295	HSDL2	8	103452039	103512716	+	CODING	-0.2777	0.029656
ENSBTAT00000063184	ENSBTAG00000046547	-	24	43217455	43224463	+	CODING	-1.51073	0.029702
ENSBTAT00000030791	ENSBTAG00000022699	CAV3	22	17832937	17846997	-	CODING	-0.33994	0.029794
ENSBTAT00000061257	ENSBTAG00000010229	LAMA2	9	68129102	68665593	+	CODING	-0.5688	0.029827
ENSBTAT00000035930	ENSBTAG00000004347	GPR116	23	20052363	20128180	-	CODING	-0.32316	0.029972
ENSBTAT00000002626	ENSBTAG00000002026	RPL5	3	50787385	50795965	-	CODING	-0.2424	0.030057
ENSBTAT00000019328	ENSBTAG00000030424	CLEC1A	5	100300781	100321208	+	CODING	-0.85972	0.030137
ENSBTAT00000026826	ENSBTAG00000020139	RPL7	14	38741347	38744856	-	CODING	-0.24379	0.030151

ENSBTAT00000025007	ENSBTAG00000018784	CTSZ	13	57889707	57899205	+	CODING	-0.73624	0.030248
ENSBTAT00000029340	ENSBTAG00000039995	CFH	16	6052925	6122550	+	CODING	-0.53855	0.030449
ENSBTAT00000023111	ENSBTAG00000017380	MYO18A	19	20994380	21082308	-	CODING	0.27874	0.030617
ENSBTAT00000061513	ENSBTAG00000019625	EHHADH	1	82467817	82521151	+	CODING	1.013492	0.0307
ENSBTAT00000003236	ENSBTAG00000002490	CHPT1	5	65852754	65882216	+	CODING	-0.29744	0.030731
ENSBTAT00000010998	ENSBTAG00000008350	KLHL21	16	47645900	47659012	+	CODING	0.434116	0.031002
ENSBTAT00000027837	ENSBTAG00000020894	LAPTM4A	11	78862495	78880461	+	CODING	-0.15596	0.031265
ENSBTAT00000001799	ENSBTAG00000001365	C1QTNF9	12	34540961	34548854	+	CODING	-0.70594	0.031339
ENSBTAT00000053250	ENSBTAG00000001274	PPM1L	1	107241227	107576086	-	CODING	0.24122	0.031454
ENSBTAT00000061219	ENSBTAG00000043975	MYCT1	9	90836602	90852725	+	CODING	-0.50672	0.031494
ENSBTAT00000016013	ENSBTAG00000012072	NDUFS8	29	46205045	46208795	+	CODING	-0.25583	0.031501
ENSBTAT00000022212	ENSBTAG00000016710	PTRH2	19	10894828	10903658	-	CODING	0.293854	0.031643
ENSBTAT00000053246	ENSBTAG00000006670	ERGIC3	13	65435895	65448951	+	CODING	-0.46907	0.031745
ENSBTAT00000003640	ENSBTAG00000045857	FAM195A	25	578685	583777	+	CODING	-0.4383	0.03182
ENSBTAT00000047151	ENSBTAG00000033190	PGM5	8	44682699	44892128	+	CODING	-0.39837	0.031861
ENSBTAT00000026501	ENSBTAG00000019891	MRPL40	17	74708565	74711079	+	CODING	-0.3013	0.031889
ENSBTAT00000023709	ENSBTAG00000017834	PRELP	16	1041923	1055957	+	CODING	-0.45829	0.0321
ENSBTAT00000011359	ENSBTAG00000008609	42986	7	22862426	22881449	+	CODING	-0.8132	0.032223
ENSBTAT00000021256	ENSBTAG00000015978	ANXA1	8	49624473	49642916	+	CODING	-0.7053	0.032289
ENSBTAT00000008447	ENSBTAG00000006441	ATP5F1	3	32003756	32020661	-	CODING	-0.14393	0.032316
ENSBTAT00000047532	ENSBTAG00000012778	CAPN2	16	27781671	27840009	+	CODING	-0.45849	0.032351
ENSBTAT00000024175	ENSBTAG00000018167	KLHL31	23	6771146	6785241	+	CODING	0.292216	0.032378
ENSBTAT00000015695	ENSBTAG00000011825	C7H19orf43	7	13896310	13899366	+	CODING	-0.3146	0.032594
ENSBTAT00000036144	ENSBTAG00000011104	RTN4	11	37575270	37649696	-	CODING	-1.00704	0.032681
ENSBTAT00000005780	ENSBTAG00000004406	MSI2	19	8651763	8720657	+	CODING	-0.75273	0.032763
ENSBTAT00000010126	ENSBTAG00000007700	PHYH	13	28254814	28275573	-	CODING	0.107138	0.032786
ENSBTAT00000002045	ENSBTAG00000001565	MESDC2	21	27322045	27330423	-	CODING	-0.46896	0.032984
ENSBTAT00000046448	ENSBTAG00000032705	CALM	20	8066341	8067501	+	CODING	0.348321	0.033203
ENSBTAT00000026386	ENSBTAG00000019803	POLR3K	25	110168	112894	-	CODING	-0.19719	0.033302
ENSBTAT00000028662	ENSBTAG00000021508	LMOD3	22	32534126	32550296	+	CODING	-0.10487	0.033422
ENSBTAT00000012110	ENSBTAG00000009191	TUSC3	27	20419472	20636849	-	CODING	-1.51406	0.033647
ENSBTAT00000020427	ENSBTAG00000015369	MLLT11	3	19762896	19767967	-	CODING	-0.55266	0.033705
ENSBTAT00000023618	ENSBTAG00000017763	NFIL3	8	87891326	87893182	+	CODING	-0.44764	0.033709
ENSBTAT00000047463	ENSBTAG00000003728	NDUFS4	20	25407689	25523382	-	CODING	0.206534	0.034191
ENSBTAT00000003129	ENSBTAG00000002412	CYB5B	18	36674604	36696376	+	CODING	-0.50568	0.034223
ENSBTAT00000025501	ENSBTAG00000019160	GLOD4	19	22633515	22653270	+	CODING	0.431852	0.034261
ENSBTAT00000018334	ENSBTAG00000005412	NEDD4L	24	57965974	58053921	+	CODING	0.399536	0.034295
ENSBTAT00000013117	ENSBTAG00000009942	PLCL2	1	155618262	155833445	+	CODING	0.383769	0.034301
ENSBTAT00000009357	ENSBTAG00000007112	C3H1ORF41	3	92831380	92864702	+	CODING	-0.61334	0.034379
ENSBTAT00000051493	ENSBTAG00000036993	U2	2	48352291	48352468	-	CODING	-2.66978	0.034452
ENSBTAT00000008676	ENSBTAG00000006614	TMCC1	22	56700645	56742873	+	CODING	0.237004	0.034505
ENSBTAT00000000930	ENSBTAG00000000695	UBAP2	8	76668494	76759843	-	CODING	0.442749	0.034524
ENSBTAT00000007374	ENSBTAG00000005614	UXS1	11	45583095	45639673	+	CODING	0.269214	0.034615
ENSBTAT00000008137	ENSBTAG00000006195	C29H11orf68	29	44691682	44693901	-	CODING	0.540616	0.034659
ENSBTAT00000055373	ENSBTAG00000038112	ULBP3	9	85948859	85961158	-	CODING	-0.92412	0.034769
ENSBTAT00000008734	ENSBTAG00000006646	HRMT1L2	18	56534833	56545053	+	CODING	-0.26376	0.034783
ENSBTAT00000020886	ENSBTAG00000015732	PSMC6	10	11401913	11425951	-	CODING	0.344749	0.034801
ENSBTAT00000027565	ENSBTAG00000020685	-	4	14000446	14001033	+	CODING	-0.47105	0.03488
ENSBTAT00000000284	ENSBTAG00000000233	OR51E1	15	51007543	51008496	-	CODING	-0.72398	0.035222
ENSBTAT00000052841	ENSBTAG00000016625	KIAA0922	17	3991592	4076484	-	CODING	0.643496	0.03523
ENSBTAT00000031877	ENSBTAG00000021799	RCN3	18	56422333	56430760	+	CODING	-0.98166	0.035265
ENSBTAT00000017775	ENSBTAG00000013363	CAP1	3	106638795	106667878	-	CODING	-0.50113	0.035301
ENSBTAT00000004017	ENSBTAG00000003084	MGC166084	10	88467535	88522547	+	CODING	-1.76605	0.035338
ENSBTAT00000002508	ENSBTAG00000001928	PDIA6	11	86834898	86857648	+	CODING	-0.43103	0.035348
ENSBTAT00000029488	ENSBTAG00000022039	CMLKR1	17	66827051	66828139	+	CODING	-1.21463	0.035421
ENSBTAT00000053824	ENSBTAG00000008285	OXR1	14	60000394	60025510	-	CODING	0.640537	0.035691
ENSBTAT00000027722	ENSBTAG00000020803	-	X	141098691	141104299	-	CODING	-2.41523	0.035801
ENSBTAT00000036093	ENSBTAG00000025595	-	22	7111728	7112287	+	CODING	-0.77798	0.035824
ENSBTAT00000008508	ENSBTAG00000006495	GNB2	25	36446227	36451406	-	CODING	-0.42303	0.035857
ENSBTAT00000001208	ENSBTAG00000000913	UQCR10	17	71038498	71040693	+	CODING	-0.10905	0.035907
ENSBTAT00000018774	ENSBTAG00000014127	PGHS-2	16	69263776	69271399	-	CODING	-1.14851	0.035975
ENSBTAT00000063548	ENSBTAG00000046763	MGC143103	22	43336993	43357321	+	CODING	-0.58024	0.035977

ENSBTAT00000024122	ENSBTAG00000018123	FBLN5	21	57153110	57246389	-	CODING	-0.49879	0.036162
ENSBTAT00000010698	ENSBTAG00000008137	-	5	75519790	75520044	+	CODING	-0.14062	0.03617
ENSBTAT00000001825	ENSBTAG00000001388	NMB	21	22741477	22744781	-	CODING	-3.45991	0.036206
ENSBTAT00000027838	ENSBTAG00000020895	LOXL4	26	19201799	19214350	-	CODING	-1.91132	0.036237
ENSBTAT00000035307	ENSBTAG00000019964	GAS6	12	90821803	90850163	-	CODING	-0.48391	0.036309
ENSBTAT00000019790	ENSBTAG00000014861	SLC20A2	27	36938406	37017424	-	CODING	-0.48156	0.036325
ENSBTAT00000011001	ENSBTAG00000008353	CDKN1A	23	10560499	10568780	+	CODING	-0.30168	0.036424
ENSBTAT00000003884	ENSBTAG00000002983	NT5C1A	3	106993389	107008708	+	CODING	0.547181	0.036485
ENSBTAT00000030532	ENSBTAG00000018313	MBNL2	12	78283460	78445751	+	CODING	0.238556	0.036493
ENSBTAT00000063711	ENSBTAG00000047896	-	17	74924020	74924274	+	CODING	0.704605	0.036845
ENSBTAT00000010173	ENSBTAG00000007732	ARPP21	22	9683024	9791554	+	CODING	0.593861	0.036983
ENSBTAT00000055244	ENSBTAG00000010336	TALDO1	29	50856122	50863474	-	CODING	-0.44319	0.037094
ENSBTAT00000004959	ENSBTAG00000003806	ECM1	3	20228528	20233933	-	CODING	-0.94333	0.03722
ENSBTAT00000065880	ENSBTAG00000021232	TPD52L2	13	54411455	54427367	-	CODING	0.467067	0.037439
ENSBTAT00000011383	ENSBTAG00000008632	-	25	26389490	26390167	+	CODING	-0.58521	0.037457
ENSBTAT00000047142	ENSBTAG00000033186	OXCT1	20	32683996	32848723	+	CODING	0.255194	0.037479
ENSBTAT00000032097	ENSBTAG00000023513	DNAJC19	1	86666813	86672081	+	CODING	-0.38482	0.037574
ENSBTAT00000030619	ENSBTAG00000015839	MAP4	22	52369974	52462511	+	CODING	0.281409	0.037684
ENSBTAT00000066309	ENSBTAG00000045628	C15orf61	10	14329838	14334934	+	CODING	-1.3302	0.037834
ENSBTAT00000017583	ENSBTAG00000013210	ADAMTS4	3	8322938	8331146	+	CODING	-1.22905	0.037847
ENSBTAT00000024660	ENSBTAG00000018527	HDGFRP3	21	25317912	25392905	+	CODING	-0.76641	0.037889
ENSBTAT00000049743	ENSBTAG00000002487	UBE3A	21	2346814	2410193	-	CODING	0.22549	0.037909
ENSBTAT00000061240	ENSBTAG00000012882	CUL5	15	17857169	17983781	+	CODING	0.346615	0.038027
ENSBTAT00000044865	ENSBTAG00000002014	SNX1	10	45899489	45936333	-	CODING	0.761299	0.038072
ENSBTAT00000009621	ENSBTAG00000007312	CD209	7	17811222	17814125	-	CODING	-2.04024	0.038118
ENSBTAT00000008170	ENSBTAG00000006222	TFDP2	1	127977930	128027258	+	CODING	0.420201	0.03815
ENSBTAT00000019734	ENSBTAG00000014818	MYLK3	18	15089441	15131888	-	CODING	2.207694	0.038243
ENSBTAT00000020550	ENSBTAG00000015457	FGFR1	27	33250534	33291989	-	CODING	-0.67151	0.038423
ENSBTAT00000027628	ENSBTAG00000020734	ARL6IP1	25	16543863	16550325	-	CODING	-0.31063	0.038435
ENSBTAT00000044261	ENSBTAG00000046337	TUBB6	24	43249500	43250564	+	CODING	-0.47877	0.03848
ENSBTAT00000034126	ENSBTAG00000024539	SPSB1	16	44994737	45006429	-	CODING	-0.784	0.038489
ENSBTAT00000043412	ENSBTAG00000019269	COL6A2	1	147542825	147570736	+	CODING	-0.66682	0.038525
ENSBTAT00000019298	ENSBTAG00000014518	RPL9	6	60210361	60215120	-	CODING	-0.225	0.038542
ENSBTAT00000000109	ENSBTAG00000000099	CERS2	3	19835017	19843638	+	CODING	-0.43977	0.038671
ENSBTAT00000064986	ENSBTAG00000046033	UBFD1	25	21476163	21488221	+	CODING	0.328646	0.038887
ENSBTAT00000004154	ENSBTAG00000003197	-	5	102100648	102101142	-	CODING	-0.45273	0.039137
ENSBTAT00000024448	ENSBTAG00000018373	DPYSL2	8	75089346	75168436	+	CODING	-0.43094	0.039241
ENSBTAT00000002376	ENSBTAG00000001817	TMEM161A	7	4032541	4046719	+	CODING	0.500161	0.039366
ENSBTAT00000065575	ENSBTAG00000046140	NPDC1	11	106192006	106194241	-	CODING	-1.0675	0.039522
ENSBTAT00000055485	ENSBTAG00000018086	TBC1D10A	17	71459718	71470126	-	CODING	-1.43947	0.039804
ENSBTAT00000000576	ENSBTAG00000000451	TMEM126A	29	9962193	9970745	-	CODING	-0.21951	0.039963
ENSBTAT00000026183	ENSBTAG00000019648	GNB2L1	7	41761805	41766598	-	CODING	-0.08693	0.039988
ENSBTAT00000009806	ENSBTAG00000007455	DNASE1L1	X	40368988	40371659	-	CODING	0.392285	0.040209
ENSBTAT00000022524	ENSBTAG00000016933	IL27RA	7	12743318	12767182	-	CODING	-1.05368	0.040352
ENSBTAT00000014101	ENSBTAG00000010663	ADAM15	3	15593459	15603312	-	CODING	-0.61641	0.040419
ENSBTAT00000027885	ENSBTAG00000020935	HIF1A	10	74095881	74139364	+	CODING	-0.50344	0.040425
ENSBTAT00000038099	ENSBTAG00000034531	RNF146B	9	24273357	24300611	-	CODING	-0.27747	0.040442
ENSBTAT00000001519	ENSBTAG00000001141	ADAM17	11	87898074	87940943	+	CODING	-1.22478	0.040508
ENSBTAT00000010403	ENSBTAG00000007913	CWC15	15	15705460	15713952	+	CODING	-0.18431	0.040618
ENSBTAT00000021144	ENSBTAG00000015904	RORA	10	48949618	49750993	+	CODING	0.502233	0.040843
ENSBTAT00000014193	ENSBTAG00000010719	ANGPTL1	16	61657545	61681733	-	CODING	-0.76091	0.040941
ENSBTAT00000027604	ENSBTAG00000003570	DDX19A	18	1772754	1791255	-	CODING	-3.6185	0.040941
ENSBTAT00000063911	ENSBTAG00000047424	TRIP10	7	18967189	18970399	-	CODING	-0.29132	0.040962
ENSBTAT00000006213	ENSBTAG00000004732	SPTB	10	77201411	77273590	-	CODING	0.490517	0.041203
ENSBTAT00000039717	ENSBTAG00000001027	SERPINH1	15	55514945	55525175	+	CODING	-0.34022	0.041274
ENSBTAT00000009178	ENSBTAG00000006984	CD55	16	5105577	5124229	-	CODING	-1.66979	0.041444
ENSBTAT00000018284	ENSBTAG00000013761	STMN1	2	127773927	127779431	+	CODING	-0.68728	0.041703
ENSBTAT00000008744	ENSBTAG00000006656	RANBP1	17	75036894	75038127	-	CODING	-0.60621	0.041729
ENSBTAT00000013737	ENSBTAG00000010402	MYH9	5	75094866	75179909	-	CODING	-0.32905	0.041949
ENSBTAT00000026119	ENSBTAG00000019604	VASP	18	53531933	53544919	+	CODING	-0.67982	0.042108
ENSBTAT00000016261	ENSBTAG00000012260	MTRF1	12	11452445	11494158	-	CODING	0.667638	0.042121
ENSBTAT00000049046	ENSBTAG00000038228	FAM127A	X	18687103	18688288	+	CODING	-0.55165	0.042223

ENSBTAT00000063984	ENSBTAG00000019832	TGFBR2	22	5176650	5233083	+	CODING	-0.83068	0.042275
ENSBTAT00000053822	ENSBTAG00000015786	LRRC39	3	43251251	43272503	+	CODING	-0.1513	0.042347
ENSBTAT00000053816	ENSBTAG00000013113	VDAC1	7	47247744	47262600	-	CODING	-0.4583	0.042457
ENSBTAT00000002983	ENSBTAG00000002317	PTN	4	101685424	101795483	-	CODING	-1.21695	0.042709
ENSBTAT00000022970	ENSBTAG00000017279	VAPA	24	42332694	42368659	+	CODING	0.142406	0.042716
ENSBTAT00000004182	ENSBTAG00000003221	ATF7IP	5	95800988	95858963	-	CODING	0.477973	0.04281
ENSBTAT00000015839	ENSBTAG00000011932	PRG4	16	68870182	68886786	+	CODING	-2.41856	0.042829
ENSBTAT00000008431	ENSBTAG00000006429	ACO2	5	113089139	113138185	+	CODING	0.122093	0.042904
ENSBTAT00000063730	ENSBTAG00000048058	-	10	4608189	4608558	+	CODING	-2.20397	0.042913
ENSBTAT00000007527	ENSBTAG00000005726	HNRNPA2B1	4	70198053	70207085	+	CODING	0.111708	0.042932
ENSBTAT00000009441	ENSBTAG00000007173	PDGFRA	6	71373513	71421283	+	CODING	-0.6754	0.042961
ENSBTAT00000015049	ENSBTAG00000011324	EMILIN1	11	72539296	72547260	-	CODING	-0.96903	0.042986
ENSBTAT00000014607	ENSBTAG00000010998	FLIP	2	90139395	90172951	+	CODING	-1.02783	0.042995
ENSBTAT00000007563	ENSBTAG00000005754	PPM1K	6	37876470	37898522	+	CODING	-1.39156	0.043025
ENSBTAT00000005011	ENSBTAG00000003842	RABGEF1	25	28509094	28534880	+	CODING	-0.438	0.043083
ENSBTAT00000053498	ENSBTAG00000037457	MBD2	24	54044363	54093578	-	CODING	-0.33477	0.043177
ENSBTAT00000049039	ENSBTAG00000005742	CYYR1	1	9242710	9250037	+	CODING	-0.31489	0.043206
ENSBTAT00000046579	ENSBTAG00000032812	-	14	45039060	45039371	-	CODING	-0.79241	0.043362
ENSBTAT00000061126	ENSBTAG00000010392	ESRRG	16	20595768	20789340	-	CODING	-1.14534	0.043723
ENSBTAT00000023028	ENSBTAG00000017318	TMEM178	11	21988495	22046009	+	CODING	-1.25489	0.043802
ENSBTAT00000019865	ENSBTAG00000014921	IL6	4	31578311	31582667	+	CODING	-2.59906	0.044111
ENSBTAT00000029261	ENSBTAG00000024086	ZMYM6NB	3	111277768	111280979	+	CODING	-1.77175	0.044118
ENSBTAT00000055170	ENSBTAG00000038286	C14H8orf22	14	21617777	21622267	+	CODING	-0.13693	0.044121
ENSBTAT00000002051	ENSBTAG00000001568	PPIC	7	32114445	32128245	+	CODING	-0.69496	0.044131
ENSBTAT00000024793	ENSBTAG00000018633	RABAC1	18	51607794	51610670	+	CODING	-0.2674	0.044512
ENSBTAT00000064232	ENSBTAG00000045692	-	18	34196399	34215940	-	CODING	-0.48417	0.044522
ENSBTAT00000000926	ENSBTAG00000000694	TAF10	15	47073272	47074686	+	CODING	-0.22018	0.044532
ENSBTAT00000017366	ENSBTAG00000013060	IQGAP1	21	22530902	22614701	-	CODING	-0.44838	0.044697
ENSBTAT00000019547	ENSBTAG00000014685	HPRT1	X	18120061	18151262	+	CODING	-0.37448	0.044905
ENSBTAT00000022129	ENSBTAG00000016637	WBP4	12	11306594	11329117	+	CODING	-0.46015	0.045104
ENSBTAT00000004892	ENSBTAG00000003758	TKT	22	48264511	48288059	+	CODING	-0.65105	0.045146
ENSBTAT00000063570	ENSBTAG00000045702	ZBED6	16	1400457	1403399	+	CODING	0.513061	0.045158
ENSBTAT00000012128	ENSBTAG00000009206	FOXS1	13	61924322	61925629	-	CODING	-0.74784	0.045247
ENSBTAT00000004943	ENSBTAG00000003800	LRRC27	26	51618947	51644391	+	CODING	2.699115	0.045286
ENSBTAT00000005025	ENSBTAG00000003851	CCNL1	1	111318106	111331103	+	CODING	-0.40131	0.045346
ENSBTAT00000007762	ENSBTAG00000005909	LEPROT	3	80243543	80254246	-	CODING	-0.61508	0.045721
ENSBTAT00000005523	ENSBTAG00000004215	RARRES2	4	113564407	113567496	-	CODING	-0.55833	0.045754
ENSBTAT00000024034	ENSBTAG00000025848	RBM3	X	91768033	91770790	+	CODING	-0.51428	0.045762
ENSBTAT00000011321	ENSBTAG00000023970	-	19	19919500	19921026	+	CODING	-1.75139	0.045855
ENSBTAT00000002784	ENSBTAG00000002147	PPP4R2	22	28775667	28823253	-	CODING	0.399338	0.046173
ENSBTAT00000052073	ENSBTAG00000037726	PLCD1	22	11497086	11518465	-	CODING	-0.95518	0.046191
ENSBTAT00000047310	ENSBTAG00000033290	TMEM60	4	43684551	43688401	+	CODING	-0.71957	0.046513
ENSBTAT00000028556	ENSBTAG00000021421	SSR4	X	39925628	39929382	+	CODING	-0.33401	0.046586
ENSBTAT00000000700	ENSBTAG00000000539	LRRC28	21	7411027	7600816	-	CODING	-0.256	0.046588
ENSBTAT00000023068	ENSBTAG00000017352	ZC3H11A	16	1404592	1440704	+	CODING	0.283625	0.046595
ENSBTAT00000000433	ENSBTAG00000000328	TPPP2	10	26128308	26130477	-	CODING	1.026461	0.046644
ENSBTAT00000026841	ENSBTAG00000020152	PFDN2	3	8395605	8407551	+	CODING	-0.29903	0.0467
ENSBTAT00000020215	ENSBTAG00000015193	BOLA3	11	10489732	10497683	+	CODING	-0.44304	0.046779
ENSBTAT00000029363	ENSBTAG00000022013	CBR4	8	943475	968096	-	CODING	0.381604	0.046797
ENSBTAT00000055617	ENSBTAG00000005225	RAD52	5	108209684	108235391	-	CODING	0.341071	0.047033
ENSBTAT00000044349	ENSBTAG00000018164	FNDC4	11	72181230	72183230	+	CODING	-2.24385	0.047077
ENSBTAT00000016321	ENSBTAG00000014269	SCARB1	17	53180927	53273723	+	CODING	-0.99084	0.047085
ENSBTAT00000028498	ENSBTAG00000021377	S100A14	3	16827337	16829392	+	CODING	-0.30997	0.047097
ENSBTAT00000008038	ENSBTAG00000006121	SBK2	18	62435402	62438857	+	CODING	-3.56	0.047141
ENSBTAT00000006074	ENSBTAG00000004630	COMP	7	4354380	4361950	+	CODING	-2.28969	0.047318
ENSBTAT00000012182	ENSBTAG00000009245	PPP2CB	27	26041259	26064654	-	CODING	-0.41282	0.047398
ENSBTAT00000005575	ENSBTAG00000004256	ODC1	11	87178098	87182503	+	CODING	0.274674	0.047406
ENSBTAT00000025341	ENSBTAG00000019037	AQP4	24	30362738	30377272	+	CODING	-1.53614	0.047882
ENSBTAT00000065097	ENSBTAG00000045497	-	21	66764061	66764393	+	CODING	-0.2556	0.047884
ENSBTAT00000019445	ENSBTAG00000011400	DBN1	7	40312121	40325975	-	CODING	-0.68836	0.047886
ENSBTAT00000020233	ENSBTAG00000015209	MXD4	6	108490181	108501580	+	CODING	-1.0037	0.047938
ENSBTAT00000023483	ENSBTAG00000017660	-	X	91260328	91261416	+	CODING	-0.68549	0.048124

ENSBTAT00000021643	ENSBTAG00000016271	SNRPF	5	60556051	60564297	+	CODING	-0.52897	0.048176
ENSBTAT00000001642	ENSBTAG00000001244	PLAT	27	36738833	36762837	-	CODING	-0.89027	0.048445
ENSBTAT00000024209	ENSBTAG00000018188	NDUFC2	29	18074821	18083006	+	CODING	-0.19681	0.048479
ENSBTAT00000005016	ENSBTAG00000003845	CSRNP1	22	12520393	12532806	-	CODING	-0.60194	0.048513
ENSBTAT00000018681	ENSBTAG00000014060	LSM6	17	12322226	12340189	-	CODING	-0.22839	0.048622
ENSBTAT00000003636	ENSBTAG00000002808	PSMA3	10	70797328	70822594	+	CODING	0.166468	0.048634
ENSBTAT00000055450	ENSBTAG00000003585	CD47	1	53103996	53169038	-	CODING	-0.35134	0.048659
ENSBTAT00000038056	ENSBTAG00000012946	HSF2	9	29122564	29161606	-	CODING	-0.46732	0.048788
ENSBTAT00000049047	ENSBTAG00000039875	-	9	88307676	88310265	-	CODING	-2.77502	0.048938
ENSBTAT00000029017	ENSBTAG00000021768	CCNG2	6	93746807	93758039	+	CODING	-0.58487	0.049008
ENSBTAT00000046528	ENSBTAG00000032763	RANBP10	18	35386426	35452124	-	CODING	0.439814	0.04909
ENSBTAT00000005722	ENSBTAG00000004367	COQ10A	5	57390328	57393708	-	CODING	0.138495	0.049253
ENSBTAT00000033670	ENSBTAG00000010161	CCL21	8	77421389	77422532	-	CODING	-0.86103	0.049366
ENSBTAT00000020651	ENSBTAG00000015543	ARHGAP35	18	54425390	54497692	+	CODING	-0.43454	0.049832
ENSBTAT00000016614	ENSBTAG00000012514	PODN	3	93728705	93751305	-	CODING	-0.45621	0.049861
ENSBTAT00000005615	ENSBTAG00000004283	PPFIBP1	5	82542192	82609403	-	CODING	-0.45171	0.049864
ENSBTAT00000008382	ENSBTAG00000006391	NDUFAB1	25	21498279	21509168	-	CODING	0.145143	0.049889
ENSBTAT00000025834	ENSBTAG00000019388	KIAA2013	16	42648166	42653791	-	CODING	0.325826	0.049897
ENSBTAT00000043916	ENSBTAG00000001700	CDC42	2	131341266	131359925	-	CODING	-0.17696	0.049954
